# Supplementary material for: Hepatitis C Virus Enhances the Invasiveness of Hepatocellular Carcinoma via EGFR-Mediated Invadopodia Formation and Activation
Source: Cells. 2019 Nov 5;8(11):1395. doi: 10.3390/cells8111395 (PMC6912298; doi:10.3390/cells8111395)
Supplement: Supplementary file 1 [file cells-08-01395-s001.zip › supplementry/Supp Table 1-min.pdf]

Supplementary Table 1. Quantitative real-time PCR primers.

SYBR Green Primers

Target gene	Forward	Reverse
Human β-actin	5'-CTGTTTTGTGGCTTGTTCAG-3'	5'-AGGAAACCTTCCCTCCTCTA-3'
Mouse β-2-microglobin b2m	5'-TTGGTTGAGAAGCAGAAACA-3'	5'-CACACAGTCAAGTTCCCAAA-3'
GAPDH	5'-GAAGGTGAAGGTCGGAGTC-3'	5'-GAAGATGGTGATGGGATTTC-3'
SLC9A1	5'-CCCAGGATTGTGCAATAGTC-3'	5'-GTACGTGGTTGTCGATGTCA-3'
ROCK	5'-CTGCTGGTGCTGGTAAGAGG-3'	5'-ATCCATCCAGCAAACAATCCGA-3'
Hepatitis C 3' UTR	5'-GCCATGGCGTTAGTATGAGTGT-3'	5'-CCCTATCAGGCAGTACCACAA-3'
N-WASP	5'-AAGAGATTAACCAAGGGAGATATAGGAA-3'	5'-CCAACCAACATGTCCAATGTG-3'
Arp2/3	5'-CCAGGTTGTGGAACGACACA-3'	5'-GCTGATGGTCACAGGTTGTAACA-3'
CTTN	5'-GGATGGATAAGAATGCGTCAAC-3'	5'-GTTACTTGTTTTGCTGGTCACAG-3'
Tks5	5'-CCACATCCGGGACGTAGCT-3'	5'-CCTCGAAGAACCGGAAGACTT-3'
MT1-MMP	5'-GCCCATTGGCCAGTTCTGGCCGG-3'	5'-CCTCGTCCACCTCAATGATGATC-3'
SRC	5'-CCTCCCGCACCCAGTTC-3'	5'-CATCGGCGTGTTTGGAGTAGT-3'

TaqMan Primers

Target gene	Assay Name	Ref. Seq. Number
MMP14	*(Hs.PT.58.39918453)*	NM_004995
SRC	*(Hs.PT.58.19132577)*	NM_198291
EGR1	*(Hs.PT.58.40805543.g)*	NM_001964
SYNJ2	*(Hs.PT.58.39211281)*	NM_003898
RHOC	*(Hs.PT.56a.21416046)*	NM_175744
SLC9A1	*(Hs.PT.58.15072523)*	NM_003047
SVIL	*(Hs.PT.58.3928007)*	NM_003174
CTTN	*(Hs.PT.58.707045)*	NM_138565
GAPDH	*(Hs.PT.39a.22214836)*	NM_002046
