# Supplementary material for: Hepatitis C Virus Enhances the Invasiveness of Hepatocellular Carcinoma via EGFR-Mediated Invadopodia Formation and Activation
Source: Cells. 2019 Nov 5;8(11):1395. doi: 10.3390/cells8111395 (PMC6912298; doi:10.3390/cells8111395)
Supplement: Supplementary file 1 [file cells-08-01395-s001.zip › supplementry/Supp Table 2-min.pdf]

Supplementary Table 2. Literature mining of invasion- and invadopodia-related genes.

List of invasion-associated proteins identified using ALS

**Protein name**	**Aliases**	**Specie**
ptk2	ptk2|fak|pp125fak|fadk|fak1	Homo sapiens
myo1c	mmi-beta|myo1c|mmib|flj23903|myr2	Homo sapiens
rac1	p21-rac1|tc-25|mgc111543|rac1|mig5	Homo sapiens
src	c-src|asv|src|src1|p60-src	Homo sapiens
akr1b10	arl-1|mgc14103|hsi|akr1b11|akr1b10|akr1b12|aldrln	Homo sapiens
itk	mgc126257|mgc126258|emt|itk|lyk	Homo sapiens
hcc	hcc	Homo sapiens
hsp90aa1	flj31884|hsp90aa1|hsp89a|hsp90a|hsp90n|hspn|hspcal4|hspcal1|lap2|hsp89|hsp86|hsp90|hspca|hspc1	Homo sapiens
krit1	krit1|cam|ccm1	Homo sapiens
aurka	mgc34538|aik|ark1|aurka|aura|stk6|stk7|btak|aurora2|stk15	Homo sapiens
mtdh	3d3|aeg1|aeg-1|lyric|mtdh	Homo sapiens
akt1	pkb-alpha|rac-alpha|akt|akt1|rac|mgc99656|prkba	Homo sapiens
pik3ca	pi3k|mgc142161|mgc142163|pik3ca|p110-alpha	Homo sapiens
sox3	ghdx|phpx|sox3|soxb|mrgh	Homo sapiens
nras	alps4|nras1|nras	Homo sapiens
mmp14	mt1-mmp|mtmmp1|mmp-14|mmp-x1|mmp14	Homo sapiens
alpl	hops|mgc167935|flj40094|alpl|flj93059|mgc161443|tnsalp|ap-tnap	Homo sapiens
vps39	hvam6p|flj46546|flj21681|vam6|vps39|kiaa0770	Homo sapiens
rab2a	rab2a|rab2	Homo sapiens
agfg1	hrb|rab|rip|mgc116940|mgc116938|dkfzp686i15205|agfg1	Homo sapiens
hspa5	flj26106|grp78|bip|hspa5	Homo sapiens
birc3	ciap2|aip1|malt2|api2|rnf49|birc3|hiap1|mihc|haip1	Homo sapiens
frap1	frap|raft1|rapt1|frap1|frap2|mtor|flj44809	Homo sapiens
cdkn2b	p15|p15ink4b|tp15|ink4b|mts2|cdkn2b	Homo sapiens
met	hgfr|rccp2|c-met|met|auts9	Homo sapiens

sardh	dmgdhl1|sar|sdh|sard|sardh|flj36475	Homo sapiens
mzf1	mzf1b|mzf-1|zscan6|znf42|zfp98|mzf1	Homo sapiens
snai2	snai2|slugh1|slug|mgc10182|ws2d	Homo sapiens
nono	nmt55|p54nrb|nrb54|nono	Homo sapiens
igfbp7	igfbp7|igfbp-7v|igfbp-7|mac25|igfbprp1|psf|fstl2	Homo sapiens
ptbp1	ptb-t|ptb-1|ptbp1|pptb|ptb2|ptb3|ptb4|hnrpi|ptb|mgc10830|hnrnpi|hnrnp-i|mgc8461	Homo sapiens
il8	monap|gcp1|il-8|il8|naf|gcp-1|lynap|lect|luct|cxcl8|mdncf|nap1|nap-1	Homo sapiens
usp21	mgc3394|usp23|usp21	Homo sapiens
xrcc1	xrcc1|rcc	Homo sapiens
vegfa	vpf|vegfa|vegf|mgc70609|vegf-a	Homo sapiens
hk2	hkii|hxk2|hk2|dkfzp686m1669	Homo sapiens
ptpn6	hptp1c|ptpn6|hcph|shp-1|ptp-1c|shp1|shp-1l|sh-ptp1	Homo sapiens
jak2	jtk10|jak2	Homo sapiens
jak1	jak1|jtk3|jak1b|jak1a	Homo sapiens
stat3	hies|mgc16063|flj20882|aprf|stat3	Homo sapiens
ephb2	pcbc|mgc87492|hek5|drt|erk|tyro5|epht3|ephb2	Homo sapiens
gpr160	gpcr150|gpr160|gpcr1	Homo sapiens
mapk3	mgc20180|p44mapk|hs44kdap|mapk3|p44erk1|humker1a|erk1|prkm3	Homo sapiens
gli2	gli2|hpe9|thp2|thp1|thp-1	Homo sapiens
adam10	adam10|cd156c|hst18717|kuz|madm	Homo sapiens
mmp2	tbe-1|clg4a|clg4|mmp-ii|mmp2|mona|mmp-2	Homo sapiens
timp1	flj90373|timp1|epa|hci|timp-1|timp|clgi	Homo sapiens
pebp4	pebp4|cork-1|mgc22776|cork1|gwtm1933|pro4408	Homo sapiens
pebp1	pebp|hcnp|rkip|pebp1	Homo sapiens
ldha	ldh-m|ldha|ldh1|pig19	Homo sapiens
myc	c-myc|bhlhe39|myc	Homo sapiens
cd44	in|hcell|ecmr-iii|pgp1|cspg8|lhr|cdw44|cd44|mgc10468|mc56|mdu3|mdu2|mic4|mutch-i	Homo sapiens

itga6	cd49f|vla-6|itga6|flj18737|dkfzp686j01244|itga6b	Homo sapiens
twist2	twist2|bhlha39|dermo1|mgc117334	Homo sapiens
mmp9	gelb|clg4b|mmp9|mmp-9	Homo sapiens
ervk2	herv-k|ervk2|polymerase	Homo sapiens
foxo1	foxo1|foxo1a|fkh1|fkhr	Homo sapiens
mapk8	jnk21b1/2|jnk1a2|jnk|mapk8|jnk1|sapk1|prkm8	Homo sapiens
jun	c-jun|ap1|jun	Homo sapiens
phpt1	cgi-202|hspc141|ba216l13.10|dkfzp564m173|php14|phpt1	Homo sapiens
suv39h1	suv39h|suv39h1|mg44|kmt1a	Homo sapiens
myst2	hbo1|hboa|myst2|kat7	Homo sapiens
ptpn11	mgc14433|ptp2c|ptpn11|shp-2|cfc|ns1|bptp3|ptp-1d|shp2|sh-ptp3|sh-ptp2	Homo sapiens
ifna1	ifl|ifn|mgc138507|mgc138505|mgc138207|ifna@|ifna1	Homo sapiens
tspan8	co-029|tm4sf3|tspan8	Homo sapiens
tp53	trp53|flj92943|p53|tp53|lfs1	Homo sapiens
jmjd1a	jmjd1|dkfzp686a24246|tsga|kdm3a|jhdm2a|jhmd2a|dkfzp686p07111|kiaa0742|jmjd1a	Homo sapiens
prmt5	jbp1|ibp72|skb1|hrmt1l5|skb1hs|prmt5	Homo sapiens
wdr5	swd3|big-3|wdr5	Homo sapiens
wdr77	wdr77|mgc2722|hkmt1069|rp11-552m11.3|nbla10071|mep50	Homo sapiens
map3k14	hs|map3k14|ftdcr1b|nik|hsnik	Homo sapiens
mirn373	mir373|mirna373|mirn373|hsa-mir-373|mir-373	Homo sapiens
pou3f2	pou3f2|otf7|brn2|pouf3|oct7	Homo sapiens
enox2	apk1|tnox|enox2|cova1	Homo sapiens
cldn7	cpetrl2|cldn7|hs.84359|ceptrl2|claudin-1	Homo sapiens
msi2	mgc3245|msi2h|flj36569|msi2	Homo sapiens
mapk14	rk|sapk2a|csbp1|csbp2|cspb1|p38|p38alpha|mapk14|prkm14|prkm15|mxi2|p-38|exip	Homo sapiens
fzd8	fz-8|fzd8|hfz8	Homo sapiens
mirn375	mir375|mirna375|mirn375|hsa-mir-375|mir-375	Homo sapiens
eif4e	eif4el1|eif4e|eif4e1|mgc111573	Homo sapiens

eif2c2	eif2c2|mgc3183|ago2|q10	Homo sapiens
uts2r	gpr14|utr|utr2|uts2r	Homo sapiens
pdcd4	h731|mgc33046|mgc33047|pdcd4	Homo sapiens
hotair	hoxas|flj41747|hotair|ncrna00072	Homo sapiens
aof2	aof2|kdm1|bhc110|lsd1	Homo sapiens
ezh2	mgc9169|kmt6|enx-1|ezh2	Homo sapiens
art1	cd296|rt6|art2|art1|mgc133217	Homo sapiens
rhoa	arh12|rhoa|arha|rhoh12|rho12	Homo sapiens
snx9	sh3pxd3a|snx9|mst155|mstp155|sh3px1|wisp	Homo sapiens
ros1	c-ros-1|ros1|ros|mcf3	Homo sapiens
slc2a1	glut|slc2a1|dyt17|mgc141896|mgc141895|glut1|glut-1	Homo sapiens
plau	plau|atf|upa|urk|u-pa|urokinase	Homo sapiens
na	na|nac	Homo sapiens
axpc1	pcarp|pca|axpc1	Homo sapiens
loxl2	loxl2|lor2|ws9-14	Homo sapiens
chek1	chk1|chek1	Homo sapiens
bad	bad|bbc2|bcl2l8	Homo sapiens
twist1	twist1|bpes3|bpes2|bhlha38|scs|twist	Homo sapiens
foxm1	mphosph2|mpp-2|hnf-3|trident|hfh11|foxm1|hfh-11|foxm1b|tgt3|ins-1|pig29|fkhl16	Homo sapiens
smad4	madh4|jip|dpc4|smad4	Homo sapiens
mapk7	mapk7|bmk1|erk5|prkm7	Homo sapiens
rela	mgc131774|rela|p65|nfkb3	Homo sapiens
tlr4	cd284|tlr4|toll|armd10|htoll	Homo sapiens
muc4	hsa276359|muc4	Homo sapiens
muc1	h23ag|cd227|pem|pum|mam6|muc1	Homo sapiens
ca9	mn|ca9|caix	Homo sapiens
aicda	aicda|aid|cda2|higm2	Homo sapiens
3.5.1.4	deaminase|acylamidase|3.5.1.4|fatty acylamidase|acylase|amidase|n-acetylaminohydrolase|amidohydrolase	Homo sapiens

eno3	mse|eno3	Homo sapiens
mlxip	bhlhe36|mir|mlxip|mondoa|kiaa0867	Homo sapiens
klf5	iklf|klf5|bteb2	Homo sapiens
hulc	hulc|ncrna00078	Homo sapiens
zeb1	areb6|zeb1|nil-2-a|zfhx1a|bzp|zeb|mgc133261|delta-ef1|tcf8|zeb-1|nil-2a|zfhep|nil2a	Homo sapiens
pbx3	pbx3	Homo sapiens
dcun1d1	rp42|scro|dcun1d1|dcun1l1|tes3|sccro	Homo sapiens
sfmbt1	dkfzp434l243|sfmbt|sfmbt1|ru1	Homo sapiens
prtn3	pr-3|acpa|mbt|agp7|p29|c-anca|prtn3	Homo sapiens
erbb2	erbb2|her2|her-2|cd340|neu|ngl|tkr1|her-2/neu	Homo sapiens
tjp1	dkfzp686m05161|zo-1|mgc133289|tjp1	Homo sapiens
cox8a	viii-l|cox|cox8|viii|cox8a|cox8l|cox8-2	Homo sapiens
egfr	erbb1|her1|mena|egfr|pig61|erbb	Homo sapiens
egf	egf|homg4|urg	Homo sapiens
cd34	cd34	Homo sapiens
prom1	ac133|cd133|rp41|proml1|mstp061|prom1	Homo sapiens
kdr	flk1|cd309|kdr|vegfr|vegfr2	Homo sapiens
parp1	parp1|parp|ppol|padprt-1|adprt|adprt1|parp-1	Homo sapiens
3.6.1.8	adenosine triphosphate pyrophosphatase|atpase|atp diphosphohydrolase [ambiguous]|atp pyrophosphatase|3.6.1.8|atp diphosphatase	Homo sapiens
stim1	gok|d11s4896e|stim1	Homo sapiens
orai1	tmem142a|flj14466|orai1|orat1|cracm1	Homo sapiens
rhd	mgc165007|cd240d|rhdva(tt)|rh30|rhii|rhd|diiic|rhxiii|rhk562-ii|rhced|rhdel|rhdcw|rhpii	Homo sapiens
actin	actin	Homo sapiens
trpv4	trp12|trpv4|vrl-2|vroac|vr-oac|otrpc4|vrl2	Homo sapiens
smad2	mgc34440|hmad-2|madh2|madr2|mgc22139|jv18|jv18-1|smad2	Homo sapiens
kitlg	kitlg|shep7|dkfzp686f2250|mgf|scf|kitl|kl-1	Homo sapiens

tnfsf13	tall2|unq383/pro715|cd256|trdl-1|april|tnfsf13|ligand	Homo sapiens
soat1	soat1|rp11-215i23.2|acact|soat|stat	Homo sapiens
myst1	myst1|hmof|flj14040|mof|kat8	Homo sapiens
yap1	yap1|yap2|yap|yki|yap65	Homo sapiens
rassf1	rda32|rassf1|123f2|nore2a|reh3p21|rassf1a	Homo sapiens
itch	itch|aif4|aip4|dj468o1.1|napp1	Homo sapiens
pxn	pxn|flj16691	Homo sapiens
bcar1	crkas|cass1|p130cas|cas1|bcar1	Homo sapiens
ddr1	ntrk4|ptk3a|ptk3|cd167|cak|ddr|rtk6|nep|eddr1|trke|mck10|ddr1	Homo sapiens
abl1	v-abl|c-abl|abl|abl1|jtk7|bcr/abl|p150	Homo sapiens
sox2	mgc2413|sox2|anop3|mcops3	Homo sapiens
setd2	flj45883|setd2|flj46217|kiaa1732|hypb|hspc069|set2|flj16420|p231hbp|flj22472|hif-1|kmt3a|flj23184	Homo sapiens
dap	dap|mgc99796	Homo sapiens
rara	rara|rar|nr1b1	Homo sapiens
ccl21	scya21|mgc34555|ecl|ccl21|slc|tca4|ckb9|6ckine	Homo sapiens
ptger4	mgc126583|ptger4|ep4|ep4r	Homo sapiens
ptgs2	ptgs2|gripghs|phs-2|cox-2|hcox-2|pghs-2	Homo sapiens
dbt	e2|mgc9061|e-2|e2b|dbt|bcate2	Homo sapiens
akt2	pkbb|akt2|rac-beta|pkbbeta|prkbb	Homo sapiens
eed	heed|eed|wait1	Homo sapiens
suz12	kiaa0160|jjaz1|suz12|chet9	Homo sapiens
vip	mgc13587|vip|phm27	Homo sapiens
adcyap1	mgc126852|adcyap1|pacap	Homo sapiens
vdac1	mgc111064|porin-31-hl|vdac1|porin	Homo sapiens
atp8a2	ib|atp8a2|atpib|dkfzp434b1913|atp|ml-1	Homo sapiens
postn	pn|pdlpostn|periostin|osf-2|rp11-412k4.1|postn|mgc119511|mgc119510	Homo sapiens
mirn200b	mir-200b|mirn200b|mir200b	Homo sapiens
cdc42	cdc42hs|cdc42|g25k	Homo sapiens

arhgap1	cdc42gap|arhgap1|rhogap1|rhogap|p50rhogap	Homo sapiens
il6	il-6|hsf|il6|bsf2|ifnb2	Homo sapiens
spp1	eta-1|opn|spp1|bspi|mgc110940	Homo sapiens
sirt1	sir2l1|sirt1	Homo sapiens
dicer1	herna|dicer|dicer1|kiaa0928	Homo sapiens
afp	afp|hpafp|feta	Homo sapiens
cxcr4	hsy3rr|hm89|npyy3r|cd184|d2s201e|lestr|lcr1|npy3r|npyrl|cxcr4|whim|fb22	Homo sapiens
apc	gs|dp2.5|apc|dp3|dp2|btps2	Homo sapiens
ifi27	isg12a|isg12|p27|fam14d|ifi27	Homo sapiens
abce1	rnaseli|rnasel1|abc38|abce1|rli|rns4i|oabp	Homo sapiens
decr1	decr1|decr|nadph	Homo sapiens
cav1	vip21|cav-1|cav1|mstp085	Homo sapiens
h2afx	h2ax|h2afx|h2a/x|h2a.x	Homo sapiens
sparc	on|sparc	Homo sapiens
kras	c-k-ras|rask2|ki-ras|kras2|kras1|k-ras4b|k-ras4a|k-ras2a|k-ras2b|ns3|kras	Homo sapiens
etv1	mgc120534|mgc120533|dkfzp781l0674|mgc104699|er81|etv1	Homo sapiens
has2	mgc126242|mgc126241|has2	Homo sapiens
pc-3	pc-3|psmp	Homo sapiens
arf6	arf6|dkfzp564m0264	Homo sapiens
snord87	u87|hbii-276|snord87	Homo sapiens
psip1	psip1|psip2|paip|mgc74712|dfs70|ledgf	Homo sapiens
sfrs1	sfrs1|sf2|mgc5228|srp30a|sf2p33	Homo sapiens
ifrd1	pc4|tis7|ifrd1	Homo sapiens
hla-e	hla-e|ea2.1|ea1.2|mhc|qa1|dkfzp686p19218|hla-6.2	Homo sapiens
il10	mgc126451|mgc126450|il10|il-10|il10a|tgif|csif	Homo sapiens
fbxl20	scrapper|mgc15482|scr|fbl20|fbxl20	Homo sapiens
fpr1	fmlp|fpr1|fpr	Homo sapiens
plaur	plaur|cd87|upar|urkr	Homo sapiens

tug1	flj20618|ti-227h|tug1|ncrna00080|mgc46067	Homo sapiens
tes	mgc1146|tes|tess|tess-2|dkfzp586b2022	Homo sapiens
pak7	pak7|kiaa1264|mgc26232	Homo sapiens
cdkn1a	cap20|p21cip1|p21|sdi1|cdkn1|cip1|mda-6|waf1|cdkn1a	Homo sapiens
il8rb	cmkar2|cdw128b|il8rb|il8r2|cxcr2	Homo sapiens
hdac1	dkfzp686h12203|hdac1|rpd3|hd1|rpd3l1|gon-10	Homo sapiens
1.4.1.20	phd|l-phenylalanine dehydrogenase|phenylalanine dehydrogenase|1.4.1.20	Homo sapiens
bcl9	mgc131591|lgs|bcl9	Homo sapiens
pygo2	flj33226|pygo2|1190004m21rik	Homo sapiens
lats1	warts|lats1	Homo sapiens
hla-g	hla-g|mhc-g	Homo sapiens
ctbs	ctb|ctbs	Homo sapiens
cd8a	cd8alpha|cd8|cd8-alpha|cd8a|cd8 alpha|leu2	Homo sapiens
cd4	cd4|cd4mut	Homo sapiens
rhob	rhob|arh6|arhb|mst081|mstp081|rhoh6	Homo sapiens
mirn19a	mir19a|mirna19a|mirn19a|hsa-mir-19a|mir-19a	Homo sapiens
mirn187	mirn187|mir-187	Homo sapiens
cd276	cd276|b7h3|b7-h3	Homo sapiens
slc16a4	slc16a4|mct4	Homo sapiens
cma1	chymase|cyh|cma1|mct1|mgc119891|mgc119890	Homo sapiens
cltc	hc|clh-17|chc|cltc|cltcl2|kiaa0034|chc17	Homo sapiens
cr1	kn|cr1|c3br|cd35	Homo sapiens
lamc2	lamnb2|b2t|csf|lamb2t|mgc141938|mgc138491|ebr2|lamc2	Homo sapiens
cdx2	cdx-3|cdx3|cdx2	Homo sapiens
lbx1	lbx1h|hpx6|homeobox|hpx-6|lbx1	Homo sapiens
ccr5	cc-ckr-5|cmkbr5|cd195|ckr-5|ccckr5|ccr5|ckr5	Homo sapiens
ccl5	tcp228|d17s136e|mgc17164|sisd|ccl5|scya5|rantes	Homo sapiens
nupr1	nupr1|com1	Homo sapiens

runx2	osf2|pebp2a1|pebp2aa|pebp2a2|cbfa1|runx2|mgc120023|mgc120022|ccd|aml3|pea2aa|ccd1|pebp2aa1	Homo sapiens
ier3	ier3|iex1|gly96|dif-2|dif2|iex-1|iex-1l	Homo sapiens
relb	relb|irel|i-rel	Homo sapiens
hm13	psl3|dj324o17.1|hm13|h13|spp|psenl3|mstp086|impas	Homo sapiens
pten	pten1|pten|bzs|mmac1|mgc11227|mham	Homo sapiens
mirn214	mir214|mirna214|mirn214|mir-214	Homo sapiens
eif3h	eif3-p40|eif3-gamma|eif3h|eif3s3|mgc102958	Homo sapiens
pou5f1	otf3|otf4|pou5f1|mgc22487|oct3|oct4	Homo sapiens
vcp	mgc131997|mgc148092|ibmpfd|vcp|tera|mgc8560	Homo sapiens
gabpa	e4tf1a|gabpa|e4tf1-60|gapbalpha|nrf2a|nft2|nrf2|gabp-alpha	Homo sapiens
keap1	mgc9454|mgc1114|mgc20887|mgc4407|klhl19|mgc10630|keap1|kiaa0132|inrf2	Homo sapiens
cct4	mgc126165|mgc126164|cct-delta|srb|cct4|cctd	Homo sapiens
slc2a3	flj90380|slc2a3|glut3	Homo sapiens
sp1	sp1|sp-1	Homo sapiens
zeb2	zeb2|zfhx1b|smadip1|kiaa0569	Homo sapiens
ogt	ogt|mgc22921|o-glcnac|hrnt1|flj23071	Homo sapiens
mgea5	mgea5|flj11229|ncoat|oga|mea5|kiaa0679|flj23355	Homo sapiens
mirn26b	mir26b|mirn26b|hsa-mir-26b|mir-26b	Homo sapiens
traf5	rnf84|mgc:39780|traf5	Homo sapiens
mirn26a1	mirn26a1|mir26a|mir26a1|mir-26a	Homo sapiens
atm	mgc74674|ata|atc|ate|atm|atdc|telo1|tel1|dkfzp781a0353	Homo sapiens
s100a4	fsp1|42a|pel98|capl|s100a4|18a2|p9ka	Homo sapiens
rassf10	rassf10	Homo sapiens
rrm2	rr2m|rrm2	Homo sapiens
commd7	mgc33315|flj14987|dj1085f17.3|c20orf92|commd7	Homo sapiens
bax	bax|bcl2l4	Homo sapiens
bcl2	bcl2|bcl-2	Homo sapiens
prkch	pkc-l|pkcl|npkc-eta|mgc5363|mgc26269|prkcl|prkch	Homo sapiens

ednra	eta|ednra|etra	Homo sapiens
tfpi	epi|tfpi|laci|tfpi1	Homo sapiens
il17a	il17|il-17|ctla8|il17a|il-17a	Homo sapiens
hspb1	hmn2b|dkfzp586p1322|srp27|hs.76067|cmt2f|hsp28|hsp27|hsp25|hspb1	Homo sapiens
ccr4	cc-ckr-4|hgcn:14099|mgc88293|cmkbr4|cd194|k5-5|ccr4|ckr4|chemr13	Homo sapiens
mmp13	clg3|mmp-13|mmp13	Homo sapiens
jag1	cd339|ags|ahd|aws|hj1|jag1|mgc104644|jagl1	Homo sapiens
opcml	opcm|opcml|obcam	Homo sapiens
timp3	timp3|k222|sfd|timp-3|hsmrk222|k222ta2	Homo sapiens
adamts5	adamts11|adamts-5|flj36738|admp-2|adamts5	Homo sapiens
fut1	hh|fut1|hsc	Homo sapiens
abl2	flj31718|arg|abl2|abll|flj41441|flj22224	Homo sapiens
arg2	arg2	Homo sapiens
rab27a	rab27|rab27a|hst18676|gs2|ram|mgc117246	Homo sapiens
mmp11	sl-3|stmy3|mmp11	Homo sapiens
mirn145	mir145|mirn145|mirna145|mir-145	Homo sapiens
ccnd1	d11s287e|ccnd1|bcl1|prad1|u21b31	Homo sapiens
bid	fp497|mgc15319|bid|mgc42355	Homo sapiens
dnmbp	dnmbp|tuba	Homo sapiens
tpt1	flj27337|hrf|p02|tctp|tpt1	Homo sapiens
scaf1	sr-a1|flj00034|scaf1	Homo sapiens
hspa4	ry|hs24/p52|mgc131852|hsp70ry|apg-2|hsp70|hspa4	Homo sapiens
stat6	d12s1644|stat6c|stat6b|il-4-stat|stat6	Homo sapiens
tam	tam	Homo sapiens
tlr2	cd282|til4|tlr2	Homo sapiens
tlr1	mgc126312|mgc126311|cd281|til|dkfzp547i0610|rsc786|tlr1|mgc104956|kiaa0012|dkfzp564i0682	Homo sapiens
mmp15	mt2-mmp|mtmmp2|smcp-2|mmp15	Homo sapiens
msc	abf-1|abf1|bhlha22|msc|myor	Homo sapiens

il8ra	c-c-ckr-1|il8rba|cmkar1|cd181|cd128|cdw128a|c-c|il8ra|il8r1|cxcr1	Homo sapiens
igf1r	igfir|jtk13|cd221|mgc18216|mgc142172|mgc142170|igf-1r|igf1r	Homo sapiens
insr	hhf5|cd220|insr	Homo sapiens
erbb4	erbb4|her4|p180erbb4|mgc138404	Homo sapiens
pdgfb	flj12858|pdgfb|pdgf2|c-sis|sis|ssv	Homo sapiens
pdgfrb	jtk12|pdgfrb|pdgfr1|pdgf-r-beta|cd140b|pdgfr	Homo sapiens
col1a1	col1a1|oi4	Homo sapiens
agrp	asip2|art|agrt|agrp|mgc118963	Homo sapiens
art3	flj26404|art3	Homo sapiens
nkd2	naked2|nkd2	Homo sapiens
fut4	fct3a|fut4|cd15|fuc-tiv|elft	Homo sapiens
ceacam3	cd66d|cgm1|w264|w282|mgc119875|ceacam3	Homo sapiens
ceacam5	dkfzp781m2392|cd66e|cea|ceacam5	Homo sapiens
ptx3	ptx3|tnfaip5|tsg-14	Homo sapiens
cdc25c	cdc25|cdc25c	Homo sapiens
cdc2	mgc111195|dkfzp686l20222|cdc28a|cdc2|cdk1	Homo sapiens
scrib	crib1|vartul|scrib1|scrb1|scrib	Homo sapiens
zdhhc7	znf370|zdhhc7|flj20279|flj10792	Homo sapiens
hras	n-ras|rash1|c-bas/has|hras|c-ha-ras1|k-ras|c-h-ras|ctlo|hras1|hamsv|h-rasidx	Homo sapiens
paep	gd|paeg|paep|pp14|gdf|gds|pep|mgc142288|mgc138509	Homo sapiens
bmp1	pcolc|pcp-2|pcp|tld|bmp1|flj44432	Homo sapiens
sohlh2	flj20449|teb1|sohlh2|ba121n13.2	Homo sapiens
axl	jtk11|axl|ufo	Homo sapiens
fos	c-fos|fos|ap-1	Homo sapiens
atf2	hb16|mgc111558|atf-2|atf2|cre-bp1|treb7	Homo sapiens
tmprss4	mt-sp2|tmprss4	Homo sapiens
pcsk1	pcsk1|pc1|pc3|spc3|nec1	Homo sapiens
nampt	vf|pbef1|pbef|1110035o14rik|visfatin|mgc117256|	Homo sapiens

	dkfzp666b131|nampt	
naprt1	naprt1|pp3856	Homo sapiens
pak4	pak4	Homo sapiens
ctgf	hcs24|igfbp8|mgc102839|ccn2|ctgf|nov2	Homo sapiens
ccl2	mgc9434|hsmcr30|hc11|gdcf-2|ccl2|mcp-1|mcaf|mcp1|scya2|smc-cf	Homo sapiens
mmp1	clg|mmp1|mmp-1	Homo sapiens
prame	oip4|mape|prame	Homo sapiens
nf-kappab	nf-kappab|nfkappab|nf-kappa b|nfkb|nfkappa b	Homo sapiens
dusp4	dusp4|hvh2|typ|mkp-2|mkp2	Homo sapiens
notch1	notch-1|tan1|notch1	Homo sapiens
mirn143	mir143|mirn143|mir-143	Homo sapiens
nostrin	mgc20702|nostrin|daip2	Homo sapiens
mirn221	mir221|mirna221|mirn221|mir-221	Homo sapiens
cyld	flj31664|hspc057|eac|mft|sbs|tem|flj20180|cdmt|uspl2|cyld|mft1|kiaa0849|flj78684|cyldi|cyld1	Homo sapiens
irf6	ofc6|irf6|lps|pit|pps|vws	Homo sapiens
ly96	md2|ly96|md-2	Homo sapiens
nr0b1	ahc|ahx|dss|gtd|ahch|nr0b1|dax1|dax-1|nrob1	Homo sapiens
col2a1	mgc131516|col2a1|aom|anfh|sedc|col11a3	Homo sapiens
creb1	mgc9284|creb1|creb	Homo sapiens
cd47	cd47|mer6	Homo sapiens
cops5	mgc3149|jab1|sgn5|mov-34|csn5|cops5	Homo sapiens
wwtr1	wwtr1|dkfzp586i1419	Homo sapiens
taz	flj27390|xap-2|lvncx|efe|taz|cmd3a|taz1|bths|efe2|g4.5	Homo sapiens
epha2	eck|epha2	Homo sapiens
mirn141	mir141|mirn141|mir-141	Homo sapiens
fas	alps1a|fas|apt1|tnfrsf6|cd95|fastm|apo-1|fas1	Homo sapiens
bcl2l1	bcl-xl/s|bclx|dkfzp781p2092|bcl2l1|bcl-xs|bcl-xl|bcl2l|bcl-x	Homo sapiens
prkaa1	mgc33776|ampk|ampka1|mgc57364|prkaa1	Homo sapiens

aprt	mgc125856|mgc125857|dkfzp686d13177|amp|aprt|mgc129961	Homo sapiens
dnmt1	dnmt1|flj16293|mcmt|dnmt|mgc104992|cxxc9	Homo sapiens
hdac9	hdac9|hdac|hdrp|dkfzp779k1053|hdac9fl|hd7|hdac7b|hdac9b|mitr|kiaa0744	Homo sapiens
prex1	kiaa1415|p-rex1|prex1	Homo sapiens
cic	cic|kiaa0306	Homo sapiens
tsc1	mgc86987|lam|tsc1|kiaa0243	Homo sapiens
slc12a3	tsc|slc12a3|flj96318|ncct	Homo sapiens
ing4	p29ing4|ing4|ing-4|mgc12557|my036	Homo sapiens
il13	il13|il-13|bhr1|mgc116788|mgc116789|mgc116786|p600	Homo sapiens
zfp36	tis11|nup475|ttp|g0s24|zfp36|rnf162a|gos24	Homo sapiens
uca1	uca1	Homo sapiens
bcatenin	bcatenin|beta catenin|beta-catenin	Homo sapiens
hmgb1	hmgb1|hmg3|hmg1|dkfzp686a04236|sbp-1	Homo sapiens
tmprss11d	mgc150587|mgc150588|hat|tmprss11d	Homo sapiens
hdac8	hdac8|hdacl1	Homo sapiens
hdac3	rpd3-2|hdac3|hd3	Homo sapiens
hdac2	yaf1|hdac2	Homo sapiens
mirn223	mir223|mirna223|mirn223|mir-223	Homo sapiens
chrna4	ebn|bfnc|chrna4|nacra4|flj95812|ebn1|nachr|nachra4	Homo sapiens
trim29	trim29|flj36085	Homo sapiens
eif2ak3	p-erk|perk|hri|pek|dkfzp781h1925|eif2ak3	Homo sapiens
fgfr2	flj98662|bfr-1|cd332|fgfr-2|bek|jws|fgfr2|k-sam|kgfr|tk25|tk14|cek3|cfd1|ect1	Homo sapiens
nudt6	fgf2as|fgf-as|nudt6|gfg|fgf-2|asfgf2|gfg-1	Homo sapiens
sfn	ywhas|sfn	Homo sapiens
pak1	pak1|pakalpha|mgc130001|mgc130000	Homo sapiens
macc1	7a5|macc1|sh3bp4l	Homo sapiens
ang	rnase5|ang|hel168|als9|mgc22466|mgc71966	Homo sapiens
mas1	mas|mas1|mgc119966	Homo sapiens

ace	cd143|ace|dcp|ace1|dcp1|mgc26566	Homo sapiens
mlc1	vl|lvm|mlc|mlc1|kiaa0027	Homo sapiens
myh14	kiaa2034|myh14|mhc16|dfna4|myosin|flj13881|fp17425|dkfzp667a1311|nmhc-ii-c|flj43092	Homo sapiens
pax3	pax3|hup2|mgc120384|mgc120383|mgc120382|mgc120381|mgc134778|ws1|cdhs	Homo sapiens
gfap	flj45472|gfap	Homo sapiens
pcna	pcna|mgc8367	Homo sapiens
cap1	cap1-pen|cap1	Homo sapiens
twf1	a6|ptk9|mgc41876|mgc23788|twf1	Homo sapiens
mirn206	mir206|mirna206|mirn206|mir-206	Homo sapiens
il11	il11|il-11|agif	Homo sapiens
srf	srf|mcm1	Homo sapiens
mkl1	mrtf-a|bsac|mkl1	Homo sapiens
akr1b1	mgc1804|adr|aldr1|alr2|akr1b1	Homo sapiens
rps19	dba|rps19	Homo sapiens
nmt1	nmt|nmt1	Homo sapiens
mtm1	cnm|xlmtm|mtm1|mtmx	Homo sapiens
bpp	pmgx|bpp|cbps	Homo sapiens
serpinb3	scca-pd|serpinb3|t4-a|scc|scca-1|scca1|hst1196	Homo sapiens
lct	lac|lct|lph|lph1	Homo sapiens
mtss1	mim|mtss1|dkfzp781p2223|mimb|mima|kiaa0429|flj44694	Homo sapiens
map3k5	ask1|mapkkk5|map3k5|mekk5	Homo sapiens
myd88	myd88|myd88d	Homo sapiens
prex2	dep.2|depdc2|p-rex2|prex2	Homo sapiens
dgcr14	es2el|es2|dgs-h|dgs-i|dgcr13|dgcr14|dgsi|dgsh|es-2	Homo sapiens
dlc1	arhgap7|flj21120|stard12|dlc1|p122-rhogap	Homo sapiens
slurp1	mdm|anup|ly6ls|slurp1	Homo sapiens
cdkn2a	p16ink4a|ink4|arf|mlm|p14|p16|p19|p16ink4|cdk4i|cdkn2|tp16|p14arf|cmm2|ink4a|mts1|cdkn2a	Homo sapiens

braf	mgc126806|rafb1|braf|braf1|flj95109|b-raf1|mgc138284	Homo sapiens
usp14	tgt|usp14	Homo sapiens
hccs	dkfzp779i1858|hccs|cchl|mcops7	Homo sapiens
c9orf140	p42.3|c9orf140	Homo sapiens
klf4	gklf|ezf|klf4	Homo sapiens
vav3	flj40431|vav3	Homo sapiens
wnk1	prkwnk1|kdp|pha2c|kiaa0344|wnk1	Homo sapiens
mirn34a	mir34a|mirna34a|mirn34a|mir-34a	Homo sapiens
tgfb	tgf-beta|tgfbeta|tgfb	Homo sapiens
sox9	sox9|cmd1	Homo sapiens
hcfc1r1	hpip|flj20568|hcfc1r1|mgc70711|mgc99622	Homo sapiens
pftk1	pftaire1|pftk1|kiaa0834	Homo sapiens
tsc2	tsc4|tsc2|flj43106	Homo sapiens
capza1	capz|caz1|capza1|cappa1	Homo sapiens
sfrp1	frza|sfrp1|sarp2|frp-1	Homo sapiens
id1	id|id-1|id1|bhlhb24	Homo sapiens
nqo1	nmori|nmor1|dtd|qr1|dhqu|dia4|nqo1	Homo sapiens
hbe1	hbe1|hbe	Homo sapiens
hmox1	hmox1|ho-1|bk286b10|hsp32	Homo sapiens
dld	gcsl|phe3|dld|lad|dldh	Homo sapiens
rplp0p2	rplp0p2|rplp0l2	Homo sapiens
mirn33a	mir33a|mir-33|mir33|mirn33a|hsa-mir-33a|mirn33| hsa-mir-33|mir-33a	Homo sapiens
gde1	363e6.2|gde1|mir-16|mir16	Homo sapiens
glipr1	rtvp1|glipr|glipr1|crisp7	Homo sapiens
mecp2	ppmx|dkfzp686a24160|mrx16|mrx79|rts|rtt|autsx3| mrxs13|mecp2	Homo sapiens
cldn6	cldn6	Homo sapiens
prdx2	nkefb|prx2|tsa|tdpx1|mgc4104|prdx2|prxii	Homo sapiens
msmb	psp57|psp94|pn44|prps|igbf|msp|psp|hpc13|psp-94| msmb|mspb	Homo sapiens

mirn451	mir451|mirn451|hsa-mir-451|mir-451	Homo sapiens
atp2c1	hspca1|pmr1|atp2c1|kiaa1347|spc-a-1|spca1|hhd|atp2c1a|bcpm	Homo sapiens
wwp2	wwp2-like|aip2|wwp2	Homo sapiens
s100a8	p8|ma387|nif|l1ag|caga|cfag|cgla|s100a8|cp-10|mrp8|60b8ag	Homo sapiens
socs2	socs2|cish2|socs-2|stati2|ssi2|ssi-2|cis2	Homo sapiens
sgcb	lgmd2e|a3b|sgc|sgcb	Homo sapiens
rln2	h2|ba12d24.1.1|ba12d24.1.2|rlxh2|rln2|h-2	Homo sapiens
tdgf1	cripto-1|tdgf1|crgf|cripto	Homo sapiens
itgam	cd11b|mac-1|mac1a|itgam|mgc117044|cr3a|mo1a	Homo sapiens
itgb2	lcamb|itgb2|cd18|mf17|mfi7	Homo sapiens
itgal	cd11a|lfa-1|lfa1a|itgal	Homo sapiens
nhs	nhs|dkfzp781l0254|dkfzp781f2016	Homo sapiens
plg	dkfzp779m0222|plg	Homo sapiens
mirn433	mir433|mirn433|mirna433|hsa-mir-433|mir-433	Homo sapiens
mirn182	mir182|mirn182|mirna182|mir-182	Homo sapiens
nrn1	nrn|dj380b8.2|mgc44811|nrn1	Homo sapiens
grb2	grb2|egfrbp-grb2|ash|grb3-3|mst084|mstp084	Homo sapiens
cbl	c-cbl|cbl|rnf55|cbl2	Homo sapiens
mirn122	mirna122a|mirn122a|mir122|mirn122|mirna122|mir122a|hsa-mir-122|mir-122	Homo sapiens
gsc	gsc	Homo sapiens
rbpj	rbp-j|rbpjk|rbpj|suh|mgc61669|kbf2|igkjrb|cbf1|rbpsuh|igkjrb1	Homo sapiens
dll1	dl1|delta1|dll1	Homo sapiens
sdcbp	st1|tacip18|sycl|mda-9|sdcbp	Homo sapiens
nanog	nanog	Homo sapiens
smad7	crcs3|madh8|flj16482|smad7	Homo sapiens
ski	ski|skv	Homo sapiens
2.6.1.77	tpa|2.6.1.77|taurine-pyruvate aminotransferase	Homo sapiens

tesc	flj20607|tesc|chp3	Homo sapiens
nr0b2	shp|flj17090|nr0b2	Homo sapiens
spock1	spock|tic1|testican|spock1|flj37170	Homo sapiens
mertk	c-mer|rp38|mer|mgc133349|mertk	Homo sapiens
tyro3	brt|byk|dtk|rse|sky|tif|tyro3	Homo sapiens
ppp3ca	ppp3ca|calna|calna1|caln|ppp2b	Homo sapiens
c16orf74	c16orf74|mgc17624	Homo sapiens
ctcf	ctcf	Homo sapiens
igf2	c11orf43|insigf|igf2|flj22066|pp9974|flj44734	Homo sapiens
iqgap1	iqgap1|sar1|humorfa01|kiaa0051|p195	Homo sapiens
npc1	npc|npca1|npc1	Homo sapiens
pla2g7	ldl-pla2|pla2g7	Homo sapiens
slc6a4	5-htt|5htt|sert|slc6a4|hsert|ocd1	Homo sapiens
tlr10	mgc126398|mgc126399|cd290|tlr10|mgc104967	Homo sapiens
nek3	mgc29949|hspk36|nek3	Homo sapiens
prl	prl	Homo sapiens
cd38	t10|cd38	Homo sapiens
serpine1	pai1|serpine1|pai|planh1|pai-1	Homo sapiens
anxa4	zap36|mgc75105|anx4|dkfzp686h02120|anxa4|pig28	Homo sapiens
mmp8	hnc|clg1|pmnl-cl|mmp8|mmp-8	Homo sapiens
cyr61	igfbp10|cyr61	Homo sapiens
pfn1	pfn1	Homo sapiens
cd24	cd24a|mgc75043|cd24|flj22950|flj43543	Homo sapiens
nf1	nf1|wss|dkfzp686j1293|flj21220|vrnf|nfns	Homo sapiens
rhoc	h9|mgc1448|rhoc|mgc61427|arhc|arh9|rhoh9	Homo sapiens
mirn10b	mir10b|mirn10b|mirna10b|hsa-mir-10b|mir-10b	Homo sapiens
glp-1	glp-1|flj39703|flj39511	Homo sapiens
rcbtb1	mgc33184|rcbtb1|glp|clll7|clld7|rp11-185c18.1	Homo sapiens
adam9	adam9|mltng|mdc9|kiaa0021	Homo sapiens

mirn140	mir140|mirn140|mirna140|mir-140	Homo sapiens
mirn186	mirn186|mir-186	Homo sapiens
map3k2	mekk2b|map3k2|mekk2	Homo sapiens
mirn107	mirn107|mir-107	Homo sapiens
bdnf	mgc34632|bdnf	Homo sapiens
mdm2	hdm2|hdmx|mdm2|mgc71221	Homo sapiens
wee1	wee1a|dkfzp686i18166|flj16446|wee1hu|wee1	Homo sapiens
tbx1	tbx1c|tga|tbx1|cafs|cthm|vcfs|dorv	Homo sapiens
pdgfd	pdgf-d|iegf|pdgfd|scdgf-b|mstp036|mgc26867	Homo sapiens
muc13	flj20063|drcc1|muc13	Homo sapiens
muc3a	muc3a|mucin|muc3	Homo sapiens
lect2	mgc126628|lect2|chm2|chm-ii	Homo sapiens
vegfc	flt4-l|vrp|vegfc	Homo sapiens
ppa1	pp|ppa1|mgc111556|pp1|sid6-8061|ioppp	Homo sapiens
nek2	nek2a|hspk21|nek2|nlk1	Homo sapiens
il6r	cd126|il6r|il-6r|il-6r-1|il-6r-alpha|mgc104991	Homo sapiens
foxp4	foxp4|flj40908|hfkhla|flj44184	Homo sapiens
mfi2	cd228|map97|mgc4856|mfi2|flj38863	Homo sapiens
vcan	pg-m|cspg2|wgn|vcan|ervr|wgn1|dkfzp686k06110	Homo sapiens
cd80	cd28lg|cd28lg1|cd80|lab7	Homo sapiens
il1b	il-1|il1b|il1f2|il1beta|il1-beta|il1 beta|il-1 beta|il-1beta	Homo sapiens
mirn126	mir126|mirn126|mirna126|mir-126	Homo sapiens
smad3	dkfzp586n0721|hspc193|dkfzp686j10186| mgc60396|madh3|hst17436|jv15-2|smad3	Homo sapiens
ahr	ahr	Homo sapiens
cyp1a1	cyp1a1|p450-c|p450dx|ahh|p1-450|cp11|cyp1	Homo sapiens
tek	cd202b|tek|vmcm1|tie2|vmcm|tie-2	Homo sapiens
mirnlet7b	let-7b|mirlet7b|let7b|hsa-let-7b|mirnlet7b	Homo sapiens
e2f2	e2f2|e2f-2	Homo sapiens
dclk1	dcamkl1|dcdc3a|dclk|kiaa0369|dclk1	Homo sapiens

ttk	esk|pyt|ttk|flj38280|mps1l1	Homo sapiens
mirn132	mir132|mirn132|mirna132|mir-132	Homo sapiens
hlf	mgc33822|hlf	Homo sapiens
foxa1	mgc33105|hnf3a|tcf3a|foxa1	Homo sapiens
akap13	flj11952|ha-3|ht31|proto-lb|c-lbc|brx|lbc|proto-lbc|akap13|akap-lbc|flj43341	Homo sapiens
hpd	hpd|ppd|4hppd|glod3|4-hppd	Homo sapiens
cda	cda|cdd	Homo sapiens
slc9a1	apnh|slc9a1|flj42224|nhe1	Homo sapiens
hgf	hgfb|hpta|hgf|f-tcf	Homo sapiens
angptl1	angpt3|angptl1|angy|unq162|dj595c2.2|kiaa0351	Homo sapiens
egr1	at225|ngfi-a|krox-24|tis8|g0s30|zif-268|egr1|egr-1	Homo sapiens
eya1	bor|mgc141875|eya1	Homo sapiens
kcnn4	hsk4|hikca1|ik1|sk4|ikca1|kca4|kcnn4|kca3.1|hkca4	Homo sapiens
tyr	shep3|tyr|oca1a|ocaia	Homo sapiens
dyrk2	dyrk2|flj21365|flj21217	Homo sapiens
mirn146b	mirna146b|mir-146b|mirn146b|mir146b	Homo sapiens
f9	f9|hemb|fix|ptc|mgc129641|mgc129642	Homo sapiens
irak1	irak1|irak|pelle	Homo sapiens
gli1	gli1|gli	Homo sapiens
smo	smo|smoh	Homo sapiens
shh	hhg1|hlp3|hpe3|shh|tpt|tptps|mcopcb5|smmci	Homo sapiens
alcam	cd166|alcam|memd|flj38514|mgc71733	Homo sapiens
fut8	fut8|mgc26465	Homo sapiens
magea4	mgc21336|mage4|mage-x2|mage-41|magea4|mage4a|mage4b	Homo sapiens
spry1	spry1|hspry1	Homo sapiens
mirn21	mir-21|mir21|mirna21|mirn21|hsa-mir-21	Homo sapiens
nfatc2	nfatc2|nfat1|nfatp|kiaa0611	Homo sapiens
mirn144	mir144|mirn144|mir-144	Homo sapiens

vhl	rca1|vhl|hrca1|vhl1	Homo sapiens
fat	hfat1|fat|me5|cdhf7|fat1	Homo sapiens
st7l	flj11657|flj20284|st7l|st7r|stlr|fam4b	Homo sapiens
mirn23a	mir23a|mirna23a|mirn23a|hsa-mir-23a|mir-23a	Homo sapiens
etv4	pea3|peas3|e1af|e1a-f|etv4	Homo sapiens
cmtm3	flj31762|bnas2|cklfsf3|cmtm3|mgc51956	Homo sapiens
cdk2	cdk-2|p33(cdk2)|cdk2	Homo sapiens
adamts6	adam-ts6|adamts6	Homo sapiens
stk11	pjs|lkb1|stk11	Homo sapiens
sdhd	pgl1|pgl|sdh4|sdhd|cbt1|pgl-1	Homo sapiens
pgls	pgls|6pgl	Homo sapiens
rbp2	crbp2|rbpc2|crbpii|rbp2	Homo sapiens
cdkn1c	bws|p57|wbs|kip2|bwcr|cdkn1c	Homo sapiens
cox7a2l	cox7a2l|eb1|sig81|cox7rp|cox7ar	Homo sapiens
bap1	dkfzp686n04275|hucep-6|hucep-13|bap1|flj35406|kiaa0272|flj37180	Homo sapiens
tbx3	tbx3-iso|ums|xhl|tbx3	Homo sapiens
bcs1l	flnms|bcs|bjs|ptd|bcs1|hs.6719|h-bcs|bcs1l|gracile	Homo sapiens
stat1	stat91|isgf-3|stat-1|stat1|dkfzp686b04100	Homo sapiens
ephb4	tyro11|htk|myk1|ephb4	Homo sapiens
icam1	icam-1|bb2|icam1|p3.58|cd54	Homo sapiens
epha1	eph|mgc163163|epht|epht1|epha1	Homo sapiens
dbp	dbp|dabp	Homo sapiens
ebna1bp2	ebna1bp2|ebp2|nobp	Homo sapiens
darc	fy|gpfy|cd234|dfy|gpd|ccbp1|darc|wbcq1	Homo sapiens
tfrc	tfr|tfr1|tfrc|trfr|cd71	Homo sapiens
icam2	cd102|icam2	Homo sapiens
ets2	ets2it1|ets2	Homo sapiens
mirnlet7e	let-7e|mirlet7e|hsa-let-7e|mirnlet7e	Homo sapiens
ncrna00084	neat1|tncrna|ncrna00084	Homo sapiens

tnfaip8l2	tipe2|tnfaip8l2|flj23467	Homo sapiens
rhoh	rhoh|ttf|arhh	Homo sapiens
6-Sep	sept2|mgc20339|mgc16619|sep2|6-sep|kiaa0128	Homo sapiens
cdk5	pssalre|cdk5	Homo sapiens
ervwe1	herv-w|herv-w-env|hervw|env|herv-7q|syncytin|env-w|ervwe1	Homo sapiens
max	mgc34679|max|mgc36767|mgc18164|mgc10775|mgc11225|bhlhd4	Homo sapiens
adsl	asase|adsl|amps	Homo sapiens
defb103a	defb3|hbd3|hbp3|defb103a|defb103|hbd-3|hbp-3	Homo sapiens
ccl20	scya20|ccl20|st38|larc|ckb4|mip-3a	Homo sapiens
cxcl11	h174|scyb11|scyb9b|b-r1|ip-9|ip9|cxcl11|i-tac|mgc102770	Homo sapiens
foxc2	ld|foxc2|mfh1|fkhl14|mfh-1	Homo sapiens
nanogp8	nanogp8|mgc119250	Homo sapiens
mirn22	mir-22|mir22|mirn22|hsa-mir-22	Homo sapiens
malat1	malat1|malat-1|neat2|pro2853|ncrna00047	Homo sapiens
map2k1	map2k1|mapkk1|mek1|mkk1|prkmk1	Homo sapiens
tgfb1	ced|tgfb1|dpd1	Homo sapiens
recql	recq1|recql|recql1	Homo sapiens
rhag	cd241|rh50|rhag|rh2|rh50gp|rh50a|slc42a1	Homo sapiens
dyrk1a	hp86|mnb|dyrk1|mnbh|dyrk|dyrk1a	Homo sapiens
tbx5	hos|tbx5	Homo sapiens
cd40	mgc9013|p50|cdw40|tnfrsf5|cd40	Homo sapiens
nfkb1	mgc54151|dkfzp686c01211|ebp-1|nfkb1|kbf1|nfkb-p50|nf-kappa-b|p105|nfkb-p105	Homo sapiens
phgdh	pgad|mgc3017|3-pgdh|pdg|sera|phgdh|3pgdh	Homo sapiens
mt1jp	mt1jp|mt1np|mtb|mt1j	Homo sapiens
fbn2	cca|da9|fbn2	Homo sapiens
xiap	xiap|xlp2|ilp1|api3|birc4|miha	Homo sapiens
tacc3	tacc3|eric1|mgc133242|mgc117382	Homo sapiens

zhx2	zhx2|afr1|raf|kiaa0854	Homo sapiens
larp6	flj11196|achn|larp6	Homo sapiens
mirn195	mir195|mirn195|mirna195|mir-195	Homo sapiens
me3	me3|flj34862	Homo sapiens
setdb2	dkfzp761j1217|clll8|clld8|dkfzp586i0123|c13orf4| setdb2|kmt1f	Homo sapiens
ceacam1	bgpi|bgp1|ceacam1	Homo sapiens
bat2	g2|g-2|bat2|d6s51e|dkfzp686d09175|d6s51	Homo sapiens
gsn	gsn|dkfzp313l0718	Homo sapiens
mirn27b	mir27b|mirna27b|mirn27b|mir-27b	Homo sapiens
mirn23b	mir23b|mirna23b|mirn23b|hsa-mir-23b|mir-23b	Homo sapiens
cdk6	plstire|stqtl11|cdk6|mgc59692	Homo sapiens
spi1	of|sfpi1|pu.1|spi-a|spi-1|spi1	Homo sapiens
cpi1	spi-2.3|serine protease inhibitor 2|spi-2| growth hormone-regulated proteinase inhibitor| thyroid hormone-regulated protein|kbp|ghr-p63| cpi1|cpi-21|kallikrein-binding protein|contrapsin-like<br>protease inhibitor 1	Homo sapiens
sall4	znf797|dj1112f19.1|mgc133050|sall4|drrs|hsal4	Homo sapiens
sox1	sox1	Homo sapiens
angpt2	angpt2|ang2|agpt2	Homo sapiens
mirn100	mirn100|mir-100	Homo sapiens
rfxank	f14150_1|rfxank|bls|ankra1|rfx-b|mgc138628	Homo sapiens
pik3r2	p85-beta|pik3r2|p85b	Homo sapiens
mirn216a	mirn216a|mir216|mirna216|mirn216|mir-216	Homo sapiens
tcf4	pths|tcf-4|itf2|bhlhb19|sef2|tcf4|mgc149723| mgc149724|sef2-1b|sef2-1a|e2-2|sef2-1	Homo sapiens
id2	gig8|id2h|id2a|id2|bhlhb26|mgc26389	Homo sapiens
sox12	sox12|sox22	Homo sapiens
mirn25	mir-25|mir25|mirn25|hsa-mir-25	Homo sapiens
rbm24	flj26355|rbm24|dj259a10.1|rnpc6|flj37697|flj30829	Homo sapiens

loc553103	loc553103	Homo sapiens
camp	cramp|cap18|fall-39|camp|ll37|hsd26|fall39|ll-37	Homo sapiens
csda	csda1|csda|dbpa|zonab	Homo sapiens
mirn204	mir204|mirna204|mirn204|mir-204	Homo sapiens
lepr	cd295|obr|lepr	Homo sapiens
st6gal1	st6gal1|st6gali|st6gal-i|mgc48859|siat1|st6n|cd75	Homo sapiens
timp2	timp2|timp-2|csc-21k	Homo sapiens
cdkn1b	p27kip1|cdkn4|kip1|men4|men1b|cdkn1b	Homo sapiens
il34	c16orf77|mgc34647|il34|il-34	Homo sapiens
mrc1	cd206|clec13d|mmr|mrc1	Homo sapiens
csf1r	csf1r|c-fms|cd115|fms|csfr|fim2	Homo sapiens
cd68	cd68|scard1|dkfzp686m18236|gp110	Homo sapiens
lef1	tcf1alpha|lef1|dkfzp586h0919	Homo sapiens
hpse	hpse|hse1|hpa|hpse1	Homo sapiens
slamf7	slamf7|cracc|19a|cd319	Homo sapiens
gata4	mgc126629|gata4	Homo sapiens
ppm1h	kiaa1157|flj13253|arhcl1|urcc2|ppm1h	Homo sapiens
znf395	dkfzp434k1210|hdbp2|znf395|prf-1|si-1-8-14	Homo sapiens
app	abeta|aaa|ad1|app|abpp|pn2|appi|ctfgamma| cvap	Homo sapiens
tarbp2	trbp|tarbp2|loqs|trbp2|trbp1	Homo sapiens
tar	tar	Homo sapiens
hoxa7	antp|hox1a|hoxa7|hox1.1	Homo sapiens
mitf	mi|bhlhe32|mitf	Homo sapiens
hnf4a	hnf4|hnf4a|tcf14|tcf|hnf4a9|hnf4a8|hnf4a7| nr2a1|mody|nr2a21|flj39654|mody1	Homo sapiens
phb2	pnas-141|phb2|bap|rea|mgc117268|bap37| bcap37	Homo sapiens
wnt5a	hwnt5a|wnt5a	Homo sapiens
ulbp2	n2dl2|ulbp2|raet1h	Homo sapiens
mia	mia|cd-rap	Homo sapiens

hmga1	hmga1|hmg-r|hmgiy|hmga1a|mgc4854|mgc4242|mgc12816	Homo sapiens
il24	il24|il-24|il10b|c49a|st16|mda-7|mda7|mob-5|fisp	Homo sapiens
mirn222	mir222|mirna222|mirn222|mir-222	Homo sapiens
ppp2r2a	mgc52248|flj26613|b55a|b55-alpha|pr55a|pr52a|ppp2r2a	Homo sapiens
fosl2	fra2|fosl2|flj23306	Homo sapiens
fosl1	fra1|fosl1|fra|fra-1	Homo sapiens
fosb	fosb|gosb|gos3|mgc42291|dkfzp686c0818|g0s3	Homo sapiens
jund	jund	Homo sapiens
junb	junb	Homo sapiens
stat5a	stat5a|stat5	Homo sapiens
4.1.2.13	4.1.2.13|ketose 1-phosphate aldolase|phosphofructoaldolase|1,6-diphosphofructose aldolase|diphosphofructose aldolase|fructose 1,6-diphosphate aldolase|fructoaldolase|aldolase|fructose 1-phosphate aldolase|fructose-bisphosphate aldolase|fructose-1,6-bisphosphate triosephosphate-lyase|zymohexase|fructose diphosphate aldolase|smaldo|fructose 1-monophosphate aldolase	Homo sapiens
ctd	ctd	Homo sapiens
rhce	rh|rhce|rhpi|rhvi|rh4|rhe|rhviii|rh30a|rhivb(j)|cd240ce|rhixb|mgc103977	Homo sapiens
trap	trap	Homo sapiens
nos2	inos|hep-nos|nos2a|nos2	Homo sapiens
phactr4	flj13171|mgc20618|dkfzp686l07205|mgc34186|phactr4	Homo sapiens
ddx6	hlr2|p54|rck|ddx6|flj36338	Homo sapiens
anxa1	anx1|anxa1|lpc1	Homo sapiens
smug1	hmudg|fdg|ung3|mgc104370|smug1	Homo sapiens
mamld1	f18|cxorf6|mamld1|f-18	Homo sapiens
dym	flj90130|dmc|dym|smc|flj20071	Homo sapiens

List of invasion-related publications identified using ALS

Source	Target	PubMed entry
ptk2	zeb1	http://www.ncbi.nlm.nih.gov/entrez/query.fcgi?cmd=Retrieve&db=pubmed&dopt=Abstract&list_uids=27286256|6|Oncotarget|||Jun|2016|JOURNAL ARTICLE
ptk2	tjp1	http://www.ncbi.nlm.nih.gov/entrez/query.fcgi?cmd=Retrieve&db=pubmed&dopt=Abstract&list_uids=27286256|6|Oncotarget|||Jun|2016|JOURNAL ARTICLE
ptk2	cldn7	http://www.ncbi.nlm.nih.gov/entrez/query.fcgi?cmd=Retrieve&db=pubmed&dopt=Abstract&list_uids=27286256|6|Oncotarget|||Jun|2016|JOURNAL ARTICLE
ptk2	ddr1	http://www.ncbi.nlm.nih.gov/entrez/query.fcgi?cmd=Retrieve&db=pubmed&dopt=Abstract&list_uids=27293031|6|Sci Rep|6|||2016|Journal Article
ptk2	abl1	http://www.ncbi.nlm.nih.gov/entrez/query.fcgi?cmd=Retrieve&db=pubmed&dopt=Abstract&list_uids=27293031|6|Sci Rep|6|||2016|Journal Article
ptk2	pxn	http://www.ncbi.nlm.nih.gov/entrez/query.fcgi?cmd=Retrieve&db=pubmed&dopt=Abstract&list_uids=27293031|6|Sci Rep|6|||2016|Journal Article
ptk2	kdr	http://www.ncbi.nlm.nih.gov/entrez/query.fcgi?cmd=Retrieve&db=pubmed&dopt=Abstract&list_uids=27297262|6|Clin Exp Pharmacol Physiol|43|10|Oct|2016|Journal Article
ptk2	fbxl20	http://www.ncbi.nlm.nih.gov/entrez/query.fcgi?cmd=Retrieve&db=pubmed&dopt=Abstract&list_uids=27322511|6|Environ Toxicol|||Jun|2016|JOURNAL ARTICLE
ptk2	ephb2	http://www.ncbi.nlm.nih.gov/entrez/query.fcgi?cmd=Retrieve&db=pubmed&dopt=Abstract&list_uids=27322511|6|Environ Toxicol|||Jun|2016|JOURNAL ARTICLE
ptk2	rhoa	http://www.ncbi.nlm.nih.gov/entrez/query.fcgi?cmd=Retrieve&db=pubmed&dopt=Abstract&list_uids=27330411|6|Cancer Cell Int|16|||2016|Journal Article
ptk2	rac1	http://www.ncbi.nlm.nih.gov/entrez/query.fcgi?cmd=Retrieve&db=pubmed&dopt=Abstract&list_uids=27330411|6|Cancer Cell Int|16|||2016|Journal Article|http://www.ncbi.nlm.nih.gov/entrez/query.fcgi?cmd=Retrieve&db=pubmed&dopt=Abstract&list_uids=27248472|6|Oncotarget|||May|2016|JOURNAL ARTICLE
ptk2	pdgfb	http://www.ncbi.nlm.nih.gov/entrez/query.fcgi?cmd=Retrieve&db=pubmed&dopt=Abstract&list_uids=27374103|6|Oncotarget|||Jun|2016|JOURNAL ARTICLE
ptk2	grb2	http://www.ncbi.nlm.nih.gov/entrez/query.fcgi?cmd=Retrieve&db=pubmed&dopt=Abstract&list_uids=27472394|6|Oncotarget|||Jul|2016|JOURNAL ARTICLE
ptk2	src	http://www.ncbi.nlm.nih.gov/entrez/query.fcgi?cmd=Retrieve&db=pubmed&dopt=Abstract&list_uids=27322511|6|Environ Toxicol|||Jun|2016|JOURNAL ARTICLE|http://www.ncbi.nlm.nih.gov/entrez/query.fcgi?cmd=Retrieve&db=pubmed&dopt=Abstract&list_uids=27472394|6|Oncotarget|||Jul|2016|JOURNAL ARTICLE|http://www.ncbi.nlm.nih.gov/entrez/query.fcgi?cmd=Retrieve&db=pubmed&dopt=Abstract&list_uids=27297262|

		6|Clin Exp Pharmacol Physiol|43|10|Oct|2016|Journal Article|http://www.ncbi.nlm.nih.gov/entrez/query.fcgi?cmd=Retrieve&db=pubmed&dopt=Abstract&list_uids=27248472|6|Oncotarget|||May|2016|JOURNAL ARTICLE|http://www.ncbi.nlm.nih.gov/entrez/query.fcgi?cmd=Retrieve&db=pubmed&dopt=Abstract&list_uids=27422337|6|Bioorg Med Chem Lett|26|16|Aug|2016|Journal Article
ptk2	frap1	http://www.ncbi.nlm.nih.gov/entrez/query.fcgi?cmd=Retrieve&db=pubmed&dopt=Abstract&list_uids=27491275|6|Matrix Biol|||Aug|2016|REVIEW,JOURNAL ARTICLE
ptk2	pik3ca	http://www.ncbi.nlm.nih.gov/entrez/query.fcgi?cmd=Retrieve&db=pubmed&dopt=Abstract&list_uids=27491275|6|Matrix Biol|||Aug|2016|REVIEW,JOURNAL ARTICLE
ptk2	akt1	http://www.ncbi.nlm.nih.gov/entrez/query.fcgi?cmd=Retrieve&db=pubmed&dopt=Abstract&list_uids=27297262|6|Clin Exp Pharmacol Physiol|43|10|Oct|2016|Journal Article|http://www.ncbi.nlm.nih.gov/entrez/query.fcgi?cmd=Retrieve&db=pubmed&dopt=Abstract&list_uids=27491275|6|Matrix Biol|||Aug|2016|REVIEW,JOURNAL ARTICLE|http://www.ncbi.nlm.nih.gov/entrez/query.fcgi?cmd=Retrieve&db=pubmed&dopt=Abstract&list_uids=27605043|6|Sci Rep|6|||2016|Journal Article
ptk2	stat5a	http://www.ncbi.nlm.nih.gov/entrez/query.fcgi?cmd=Retrieve&db=pubmed&dopt=Abstract&list_uids=27605043|6|Sci Rep|6|||2016|Journal Article
myo1c	ptk2	http://www.ncbi.nlm.nih.gov/entrez/query.fcgi?cmd=Retrieve&db=pubmed&dopt=Abstract&list_uids=27246739|6|Biochem Biophys Res Commun|476|4|Aug|2016|Journal Article
rac1	arf6	http://www.ncbi.nlm.nih.gov/entrez/query.fcgi?cmd=Retrieve&db=pubmed&dopt=Abstract&list_uids=27320871|6|Nan Fang Yi Ke Da Xue Xue Bao|36|6|Jun|2016|English Abstract,Journal Article,Research Support, Non-U.S. Gov't
rac1	cdc42	http://www.ncbi.nlm.nih.gov/entrez/query.fcgi?cmd=Retrieve&db=pubmed&dopt=Abstract&list_uids=27418645|6|Mol Cancer Res|||Jul|2016|JOURNAL ARTICLE|http://www.ncbi.nlm.nih.gov/entrez/query.fcgi?cmd=Retrieve&db=pubmed&dopt=Abstract&list_uids=27340868|6|Oncotarget|||Jun|2016|JOURNAL ARTICLE
rac1	akt1	http://www.ncbi.nlm.nih.gov/entrez/query.fcgi?cmd=Retrieve&db=pubmed&dopt=Abstract&list_uids=27320871|6|Nan Fang Yi Ke Da Xue Xue Bao|36|6|Jun|2016|English Abstract,Journal Article,Research Support, Non-U.S. Gov't|http://www.ncbi.nlm.nih.gov/entrez/query.fcgi?cmd=Retrieve&db=pubmed&dopt=Abstract&list_uids=27453460|6|Pancreatology|||Jul|2016|JOURNAL ARTICLE
rac1	vegfa	http://www.ncbi.nlm.nih.gov/entrez/query.fcgi?cmd=Retrieve&db=pubmed&dopt=Abstract&list_uids=27556698|6|Oncotarget|||Aug|2016|JOURNAL ARTICLE
src	rac1	http://www.ncbi.nlm.nih.gov/entrez/query.fcgi?cmd=Retrieve&db=pubmed&dopt=Abstract&list_uids=27248472|6|Oncotarget|||May|2016|JOURNAL ARTICLE
src	egfr	http://www.ncbi.nlm.nih.gov/entrez/query.fcgi?cmd=Retrieve&db=pubmed&dopt=Abstract&list_uids=27286795|6|Neuro Oncol|||Jun|2016|JOURNAL ARTICLE
src	kdr	http://www.ncbi.nlm.nih.gov/entrez/query.fcgi?cmd=Retrieve&db=pubmed&dopt=Abstract&list_uids=27297262|6|Clin Exp

		Pharmacol Physiol|43|10|Oct|2016|Journal Article
src	akt1	http://www.ncbi.nlm.nih.gov/entrez/query.fcgi?cmd=Retrieve&db=pubmed&dopt=Abstract&list_uids=27297262|6|Clin Exp Pharmacol Physiol|43|10|Oct|2016|Journal Article
src	ephb2	http://www.ncbi.nlm.nih.gov/entrez/query.fcgi?cmd=Retrieve&db=pubmed&dopt=Abstract&list_uids=27322511|6|Environ Toxicol|||Jun|2016|JOURNAL ARTICLE
src	grb2	http://www.ncbi.nlm.nih.gov/entrez/query.fcgi?cmd=Retrieve&db=pubmed&dopt=Abstract&list_uids=27472394|6|Oncotarget|||Jul|2016|JOURNAL ARTICLE
src	smad3	http://www.ncbi.nlm.nih.gov/entrez/query.fcgi?cmd=Retrieve&db=pubmed&dopt=Abstract&list_uids=27519288|6|Toxicology|366-367||Aug|2016|JOURNAL ARTICLE
src	ahr	http://www.ncbi.nlm.nih.gov/entrez/query.fcgi?cmd=Retrieve&db=pubmed&dopt=Abstract&list_uids=27519288|6|Toxicology|366-367||Aug|2016|JOURNAL ARTICLE
src	pik3ca	http://www.ncbi.nlm.nih.gov/entrez/query.fcgi?cmd=Retrieve&db=pubmed&dopt=Abstract&list_uids=27609774|6|FASEB J|||Sep|2016|JOURNAL ARTICLE
akr1b10	rac1	http://www.ncbi.nlm.nih.gov/entrez/query.fcgi?cmd=Retrieve&db=pubmed&dopt=Abstract&list_uids=27248472|6|Oncotarget|||May|2016|JOURNAL ARTICLE
akr1b10	src	http://www.ncbi.nlm.nih.gov/entrez/query.fcgi?cmd=Retrieve&db=pubmed&dopt=Abstract&list_uids=27248472|6|Oncotarget|||May|2016|JOURNAL ARTICLE
akr1b10	ptk2	http://www.ncbi.nlm.nih.gov/entrez/query.fcgi?cmd=Retrieve&db=pubmed&dopt=Abstract&list_uids=27248472|6|Oncotarget|||May|2016|JOURNAL ARTICLE
itk	foxm1	http://www.ncbi.nlm.nih.gov/entrez/query.fcgi?cmd=Retrieve&db=pubmed&dopt=Abstract&list_uids=27279287|6|Oncol Rep|36|2|Aug|2016|Journal Article
itk	dcun1d1	http://www.ncbi.nlm.nih.gov/entrez/query.fcgi?cmd=Retrieve&db=pubmed&dopt=Abstract&list_uids=27285984|6|Oncotarget|||Jun|2016|JOURNAL ARTICLE
itk	sfmbt1	http://www.ncbi.nlm.nih.gov/entrez/query.fcgi?cmd=Retrieve&db=pubmed&dopt=Abstract&list_uids=27285984|6|Oncotarget|||Jun|2016|JOURNAL ARTICLE
itk	vegfa	http://www.ncbi.nlm.nih.gov/entrez/query.fcgi?cmd=Retrieve&db=pubmed&dopt=Abstract&list_uids=27307601|6|Mol Cancer Ther|15|9|Sep|2016|Journal Article
itk	pcsk1	http://www.ncbi.nlm.nih.gov/entrez/query.fcgi?cmd=Retrieve&db=pubmed&dopt=Abstract&list_uids=27385093|6|Oncotarget|||Jul|2016|JOURNAL ARTICLE
itk	plau	http://www.ncbi.nlm.nih.gov/entrez/query.fcgi?cmd=Retrieve&db=pubmed&dopt=Abstract&list_uids=27385093|6|Oncotarget|||Jul|2016|JOURNAL ARTICLE
itk	jun	http://www.ncbi.nlm.nih.gov/entrez/query.fcgi?cmd=Retrieve&db=pubmed&dopt=Abstract&list_uids=27385093|6|Oncotarget|||Jul|2016|JOURNAL ARTICLE
itk	fos	http://www.ncbi.nlm.nih.gov/entrez/query.fcgi?cmd=Retrieve&db=pubmed&dopt=Abstract&list_uids=27385093|6|Oncotarget|||Jul|2016|JOURNAL ARTICLE

itk	bcatenin	http://www.ncbi.nlm.nih.gov/entrez/query.fcgi?cmd=Retrieve&db=pubmed&dopt=Abstract&list_uids=27424981|6|Eur Rev Med Pharmacol Sci|20|13|Jul|2016|Journal Article
itk	rela	http://www.ncbi.nlm.nih.gov/entrez/query.fcgi?cmd=Retrieve&db=pubmed&dopt=Abstract&list_uids=27430890|6|Oncol Rep|36|3|Sep|2016|Journal Article
itk	tgfb	http://www.ncbi.nlm.nih.gov/entrez/query.fcgi?cmd=Retrieve&db=pubmed&dopt=Abstract&list_uids=27457505|6|Sci Rep|6|||2016|Journal Article
itk	zeb2	http://www.ncbi.nlm.nih.gov/entrez/query.fcgi?cmd=Retrieve&db=pubmed&dopt=Abstract&list_uids=27470974|6|Nat Commun|7|||2016|Journal Article|http://www.ncbi.nlm.nih.gov/entrez/query.fcgi?cmd=Retrieve&db=pubmed&dopt=Abstract&list_uids=27347142|6|Oncol Lett|12|1|Jul|2016|JOURNAL ARTICLE|http://www.ncbi.nlm.nih.gov/entrez/query.fcgi?cmd=Retrieve&db=pubmed&dopt=Abstract&list_uids=27453416|6|Indian J Cancer|52 Suppl 3||Dec|2015|Journal Article
itk	ros1	http://www.ncbi.nlm.nih.gov/entrez/query.fcgi?cmd=Retrieve&db=pubmed&dopt=Abstract&list_uids=27481258|6|Lasers Med Sci|||Aug|2016|JOURNAL ARTICLE
itk	mmp9	http://www.ncbi.nlm.nih.gov/entrez/query.fcgi?cmd=Retrieve&db=pubmed&dopt=Abstract&list_uids=27481258|6|Lasers Med Sci|||Aug|2016|JOURNAL ARTICLE
itk	mmp2	http://www.ncbi.nlm.nih.gov/entrez/query.fcgi?cmd=Retrieve&db=pubmed&dopt=Abstract&list_uids=27481258|6|Lasers Med Sci|||Aug|2016|JOURNAL ARTICLE
itk	tbx1	http://www.ncbi.nlm.nih.gov/entrez/query.fcgi?cmd=Retrieve&db=pubmed&dopt=Abstract&list_uids=27503928|6|Cancer Res|||Aug|2016|JOURNAL ARTICLE
itk	met	http://www.ncbi.nlm.nih.gov/entrez/query.fcgi?cmd=Retrieve&db=pubmed&dopt=Abstract&list_uids=27530187|6|Hepatology|||Aug|2016|JOURNAL ARTICLE
itk	zeb1	http://www.ncbi.nlm.nih.gov/entrez/query.fcgi?cmd=Retrieve&db=pubmed&dopt=Abstract&list_uids=27533463|6|Oncotarget|||Aug|2016|JOURNAL ARTICLE|http://www.ncbi.nlm.nih.gov/entrez/query.fcgi?cmd=Retrieve&db=pubmed&dopt=Abstract&list_uids=27315462|6|Cell Cycle|15|18|Sep|2016|Journal Article|http://www.ncbi.nlm.nih.gov/entrez/query.fcgi?cmd=Retrieve&db=pubmed&dopt=Abstract&list_uids=27500490|6|J Clin Invest|126|9|Sep|2016|Journal Article|http://www.ncbi.nlm.nih.gov/entrez/query.fcgi?cmd=Retrieve&db=pubmed&dopt=Abstract&list_uids=27285757|6|Oncotarget|||Jun|2016|JOURNAL ARTICLE|http://www.ncbi.nlm.nih.gov/entrez/query.fcgi?cmd=Retrieve&db=pubmed&dopt=Abstract&list_uids=27520310|6|J Cancer Res Clin Oncol|142|10|Oct|2016|Journal Article
hcc	mmp2	http://www.ncbi.nlm.nih.gov/entrez/query.fcgi?cmd=Retrieve&db=pubmed&dopt=Abstract&list_uids=27348267|6|Oncogenesis|5|6||2016|Journal Article|http://www.ncbi.nlm.nih.gov/entrez/query.fcgi?cmd=Retrieve&db=pubmed&dopt=Abstract&list_uids=27266355|6|Zhonghua Yi Xue Za Zhi|96|18|May|2016|English Abstract,Journal Article,Research Support, Non-U.S. Gov't

hcc	mapk14	http://www.ncbi.nlm.nih.gov/entrez/query.fcgi?cmd=Retrieve&db=pubmed&dopt=Abstract&list_uids=27348267|6|Oncogenesis|5|6||2016|Journal Article
hcc	sp1	http://www.ncbi.nlm.nih.gov/entrez/query.fcgi?cmd=Retrieve&db=pubmed&dopt=Abstract&list_uids=27416558|6|Biomed Pharmacother|83||Jul|2016|JOURNAL ARTICLE
hcc	prkaa1	http://www.ncbi.nlm.nih.gov/entrez/query.fcgi?cmd=Retrieve&db=pubmed&dopt=Abstract&list_uids=27416558|6|Biomed Pharmacother|83||Jul|2016|JOURNAL ARTICLE
hcc	aprt	http://www.ncbi.nlm.nih.gov/entrez/query.fcgi?cmd=Retrieve&db=pubmed&dopt=Abstract&list_uids=27416558|6|Biomed Pharmacother|83||Jul|2016|JOURNAL ARTICLE
hcc	ros1	http://www.ncbi.nlm.nih.gov/entrez/query.fcgi?cmd=Retrieve&db=pubmed&dopt=Abstract&list_uids=27416558|6|Biomed Pharmacother|83||Jul|2016|JOURNAL ARTICLE
hcc	dnmt1	http://www.ncbi.nlm.nih.gov/entrez/query.fcgi?cmd=Retrieve&db=pubmed&dopt=Abstract&list_uids=27416558|6|Biomed Pharmacother|83||Jul|2016|JOURNAL ARTICLE
hcc	sox9	http://www.ncbi.nlm.nih.gov/entrez/query.fcgi?cmd=Retrieve&db=pubmed&dopt=Abstract&list_uids=27457505|6|Sci Rep|6|||2016|Journal Article
hcc	ccl2	http://www.ncbi.nlm.nih.gov/entrez/query.fcgi?cmd=Retrieve&db=pubmed&dopt=Abstract&list_uids=27486225|6|Mol Cancer Ther|||Aug|2016|JOURNAL ARTICLE
hcc	vegfa	http://www.ncbi.nlm.nih.gov/entrez/query.fcgi?cmd=Retrieve&db=pubmed&dopt=Abstract&list_uids=27461244|6|Oncol Rep|36|3|Sep|2016|Journal Article|http://www.ncbi.nlm.nih.gov/entrez/query.fcgi?cmd=Retrieve&db=pubmed&dopt=Abstract&list_uids=27507763|6|Sci Rep|6|||2016|Journal Article
hcc	kdr	http://www.ncbi.nlm.nih.gov/entrez/query.fcgi?cmd=Retrieve&db=pubmed&dopt=Abstract&list_uids=27507763|6|Sci Rep|6|||2016|Journal Article
hcc	met	http://www.ncbi.nlm.nih.gov/entrez/query.fcgi?cmd=Retrieve&db=pubmed&dopt=Abstract&list_uids=27530187|6|Hepatology|||Aug|2016|JOURNAL ARTICLE
hcc	itk	http://www.ncbi.nlm.nih.gov/entrez/query.fcgi?cmd=Retrieve&db=pubmed&dopt=Abstract&list_uids=27391793|6|Hepatol Res|||Feb|2016|JOURNAL ARTICLE|http://www.ncbi.nlm.nih.gov/entrez/query.fcgi?cmd=Retrieve&db=pubmed&dopt=Abstract&list_uids=27248828|6|Oncotarget|||May|2016|JOURNAL ARTICLE|http://www.ncbi.nlm.nih.gov/entrez/query.fcgi?cmd=Retrieve&db=pubmed&dopt=Abstract&list_uids=27285757|6|Oncotarget|||Jun|2016|JOURNAL ARTICLE|http://www.ncbi.nlm.nih.gov/entrez/query.fcgi?cmd=Retrieve&db=pubmed&dopt=Abstract&list_uids=27530187|6|Hepatology|||Aug|2016|JOURNAL ARTICLE
hsp90aa1	itk	http://www.ncbi.nlm.nih.gov/entrez/query.fcgi?cmd=Retrieve&db=pubmed&dopt=Abstract&list_uids=27248828|6|Oncotarget|||May|2016|JOURNAL ARTICLE
hsp90aa1	vegfa	http://www.ncbi.nlm.nih.gov/entrez/query.fcgi?cmd=Retrieve&db=pubmed&dopt=Abstract&list_uids=27494878|6|Oncotarget|||Aug|2016|JOURNAL ARTICLE

hsp90aa1	stat3	http://www.ncbi.nlm.nih.gov/entrez/query.fcgi?cmd=Retrieve&db=pubmed&dopt=Abstract&list_uids=27494878|6|Oncotarget|||Aug|2016|JOURNAL ARTICLE
hsp90aa1	rac1	http://www.ncbi.nlm.nih.gov/entrez/query.fcgi?cmd=Retrieve&db=pubmed&dopt=Abstract&list_uids=27575453|6|Peptides|85||Aug|2016|JOURNAL ARTICLE
aurka	krit1	http://www.ncbi.nlm.nih.gov/entrez/query.fcgi?cmd=Retrieve&db=pubmed&dopt=Abstract&list_uids=27250762|6|Target Oncol|||Jun|2016|JOURNAL ARTICLE
mtdh	akt2	http://www.ncbi.nlm.nih.gov/entrez/query.fcgi?cmd=Retrieve&db=pubmed&dopt=Abstract&list_uids=27308476|6|Mol Cell Oncol|2|3||2015|Journal Article
mtdh	akt1	http://www.ncbi.nlm.nih.gov/entrez/query.fcgi?cmd=Retrieve&db=pubmed&dopt=Abstract&list_uids=27251589|6|Sci Rep|6|||2016|Journal Article|http://www.ncbi.nlm.nih.gov/entrez/query.fcgi?cmd=Retrieve&db=pubmed&dopt=Abstract&list_uids=27308476|6|Mol Cell Oncol|2|3||2015|Journal Article
mtdh	itk	http://www.ncbi.nlm.nih.gov/entrez/query.fcgi?cmd=Retrieve&db=pubmed&dopt=Abstract&list_uids=27571703|6|Oncol Rep|||Aug|2016|JOURNAL ARTICLE
akt1	nras	http://www.ncbi.nlm.nih.gov/entrez/query.fcgi?cmd=Retrieve&db=pubmed&dopt=Abstract&list_uids=27251789|6|J Invest Dermatol|||May|2016|JOURNAL ARTICLE
akt1	akt2	http://www.ncbi.nlm.nih.gov/entrez/query.fcgi?cmd=Retrieve&db=pubmed&dopt=Abstract&list_uids=27302072|6|J Cell Biochem|||Jun|2016|JOURNAL ARTICLE|http://www.ncbi.nlm.nih.gov/entrez/query.fcgi?cmd=Retrieve&db=pubmed&dopt=Abstract&list_uids=27308476|6|Mol Cell Oncol|2|3||2015|Journal Article
akt1	arf6	http://www.ncbi.nlm.nih.gov/entrez/query.fcgi?cmd=Retrieve&db=pubmed&dopt=Abstract&list_uids=27320871|6|Nan Fang Yi Ke Da Xue Xue Bao|36|6|Jun|2016|English Abstract,Journal Article,Research Support, Non-U.S. Gov't
akt1	pak7	http://www.ncbi.nlm.nih.gov/entrez/query.fcgi?cmd=Retrieve&db=pubmed&dopt=Abstract&list_uids=27323857|6|Oncotarget|||Jun|2016|JOURNAL ARTICLE
akt1	snord87	http://www.ncbi.nlm.nih.gov/entrez/query.fcgi?cmd=Retrieve&db=pubmed&dopt=Abstract&list_uids=27394418|6|Neurochem Res|||Jul|2016|JOURNAL ARTICLE
akt1	il13	http://www.ncbi.nlm.nih.gov/entrez/query.fcgi?cmd=Retrieve&db=pubmed&dopt=Abstract&list_uids=27424080|6|Int Immunopharmacol|39||Oct|2016|Journal Article
akt1	prex2	http://www.ncbi.nlm.nih.gov/entrez/query.fcgi?cmd=Retrieve&db=pubmed&dopt=Abstract&list_uids=27446408|6|Oncol Lett|12|2|Aug|2016|JOURNAL ARTICLE
akt1	egf	http://www.ncbi.nlm.nih.gov/entrez/query.fcgi?cmd=Retrieve&db=pubmed&dopt=Abstract&list_uids=27470348|6|Biomed Pharmacother|82||Aug|2016|Journal Article
akt1	tp53	http://www.ncbi.nlm.nih.gov/entrez/query.fcgi?cmd=Retrieve&db=pubmed&dopt=Abstract&list_uids=27499479|6|Int J Oncol|||Aug|2016|JOURNAL ARTICLE
akt1	mdm2	http://www.ncbi.nlm.nih.gov/entrez/query.fcgi?cmd=Retrieve&db=pubmed&dopt=Abstract&list_uids=27499479|6|Int J Oncol|||Aug|2016|JOURNAL ARTICLE

akt1	hcc	http://www.ncbi.nlm.nih.gov/entrez/query.fcgi?cmd=Retrieve&db=pubmed&dopt=Abstract&list_uids=27509921|6|Oncol Rep|||Aug|2016|JOURNAL ARTICLE
akt1	plau	http://www.ncbi.nlm.nih.gov/entrez/query.fcgi?cmd=Retrieve&db=pubmed&dopt=Abstract&list_uids=27527140|6|Molecules|21|8||2016|Journal Article
akt1	mmp2	http://www.ncbi.nlm.nih.gov/entrez/query.fcgi?cmd=Retrieve&db=pubmed&dopt=Abstract&list_uids=27511626|6|Int J Oncol|||Aug|2016|JOURNAL ARTICLE|http://www.ncbi.nlm.nih.gov/entrez/query.fcgi?cmd=Retrieve&db=pubmed&dopt=Abstract&list_uids=27527140|6|Molecules|21|8||2016|Journal Article|http://www.ncbi.nlm.nih.gov/entrez/query.fcgi?cmd=Retrieve&db=pubmed&dopt=Abstract&list_uids=27470348|6|Biomed Pharmacother|82||Aug|2016|Journal Article|http://www.ncbi.nlm.nih.gov/entrez/query.fcgi?cmd=Retrieve&db=pubmed&dopt=Abstract&list_uids=27324095|6|Placenta|43||Jul|2016|Journal Article
akt1	egr1	http://www.ncbi.nlm.nih.gov/entrez/query.fcgi?cmd=Retrieve&db=pubmed&dopt=Abstract&list_uids=27530187|6|Hepatology|||Aug|2016|JOURNAL ARTICLE
akt1	mapk3	http://www.ncbi.nlm.nih.gov/entrez/query.fcgi?cmd=Retrieve&db=pubmed&dopt=Abstract&list_uids=27320871|6|Nan Fang Yi Ke Da Xue Xue Bao|36|6|Jun|2016|English Abstract,Journal Article,Research Support, Non-U.S. Gov't|http://www.ncbi.nlm.nih.gov/entrez/query.fcgi?cmd=Retrieve&db=pubmed&dopt=Abstract&list_uids=27297262|6|Clin Exp Pharmacol Physiol|43|10|Oct|2016|Journal Article|http://www.ncbi.nlm.nih.gov/entrez/query.fcgi?cmd=Retrieve&db=pubmed&dopt=Abstract&list_uids=27527140|6|Molecules|21|8||2016|Journal Article|http://www.ncbi.nlm.nih.gov/entrez/query.fcgi?cmd=Retrieve&db=pubmed&dopt=Abstract&list_uids=27551764|6|Biomed Pharmacother|83||Aug|2016|JOURNAL ARTICLE|http://www.ncbi.nlm.nih.gov/entrez/query.fcgi?cmd=Retrieve&db=pubmed&dopt=Abstract&list_uids=27470348|6|Biomed Pharmacother|82||Aug|2016|Journal Article
akt1	6-Sep	http://www.ncbi.nlm.nih.gov/entrez/query.fcgi?cmd=Retrieve&db=pubmed&dopt=Abstract&list_uids=27557506|6|Oncotarget|||Aug|2016|JOURNAL ARTICLE
akt1	itk	http://www.ncbi.nlm.nih.gov/entrez/query.fcgi?cmd=Retrieve&db=pubmed&dopt=Abstract&list_uids=27562816|6|Cell Physiol Biochem|39|3||2016|Journal Article|http://www.ncbi.nlm.nih.gov/entrez/query.fcgi?cmd=Retrieve&db=pubmed&dopt=Abstract&list_uids=27507215|6|Biochem Biophys Res Commun|478|2|Sep|2016|Journal Article
akt1	foxc2	http://www.ncbi.nlm.nih.gov/entrez/query.fcgi?cmd=Retrieve&db=pubmed&dopt=Abstract&list_uids=27562816|6|Cell Physiol Biochem|39|3||2016|Journal Article
akt1	zhx2	http://www.ncbi.nlm.nih.gov/entrez/query.fcgi?cmd=Retrieve&db=pubmed&dopt=Abstract&list_uids=27572273|6|Int J Oncol|49|3|Sep|2016|Journal Article
akt1	il17a	http://www.ncbi.nlm.nih.gov/entrez/query.fcgi?cmd=Retrieve&db=pubmed&dopt=Abstract&list_uids=27572304|6|Sci Rep|6|||2016|Journal Article

akt1	mmp9	http://www.ncbi.nlm.nih.gov/entrez/query.fcgi?cmd=Retrieve&db=pubmed&dopt=Abstract&list_uids=27573026|6|Int J Oncol|49|3|Sep|2016|Journal Article|http://www.ncbi.nlm.nih.gov/entrez/query.fcgi?cmd=Retrieve&db=pubmed&dopt=Abstract&list_uids=27511626|6|Int J Oncol|||Aug|2016|JOURNAL ARTICLE|http://www.ncbi.nlm.nih.gov/entrez/query.fcgi?cmd=Retrieve&db=pubmed&dopt=Abstract&list_uids=27527140|6|Molecules|21|8||2016|Journal Article|http://www.ncbi.nlm.nih.gov/entrez/query.fcgi?cmd=Retrieve&db=pubmed&dopt=Abstract&list_uids=27470348|6|Biomed Pharmacother|82||Aug|2016|Journal Article|http://www.ncbi.nlm.nih.gov/entrez/query.fcgi?cmd=Retrieve&db=pubmed&dopt=Abstract&list_uids=27324095|6|Placenta|43||Jul|2016|Journal Article
akt1	frap1	http://www.ncbi.nlm.nih.gov/entrez/query.fcgi?cmd=Retrieve&db=pubmed&dopt=Abstract&list_uids=27499479|6|Int J Oncol|||Aug|2016|JOURNAL ARTICLE|http://www.ncbi.nlm.nih.gov/entrez/query.fcgi?cmd=Retrieve&db=pubmed&dopt=Abstract&list_uids=27498786|6|Oncol Rep|||Aug|2016|JOURNAL ARTICLE|http://www.ncbi.nlm.nih.gov/entrez/query.fcgi?cmd=Retrieve&db=pubmed&dopt=Abstract&list_uids=27394418|6|Neurochem Res|||Jul|2016|JOURNAL ARTICLE|http://www.ncbi.nlm.nih.gov/entrez/query.fcgi?cmd=Retrieve&db=pubmed&dopt=Abstract&list_uids=27572296|6|Oncol Rep|||Aug|2016|JOURNAL ARTICLE|http://www.ncbi.nlm.nih.gov/entrez/query.fcgi?cmd=Retrieve&db=pubmed&dopt=Abstract&list_uids=27424080|6|Int Immunopharmacol|39||Oct|2016|Journal Article|http://www.ncbi.nlm.nih.gov/entrez/query.fcgi?cmd=Retrieve&db=pubmed&dopt=Abstract&list_uids=27491275|6|Matrix Biol|||Aug|2016|REVIEW,JOURNAL ARTICLE|http://www.ncbi.nlm.nih.gov/entrez/query.fcgi?cmd=Retrieve&db=pubmed&dopt=Abstract&list_uids=27563247|6|Onco Targets Ther|9|||2016|Journal Article|http://www.ncbi.nlm.nih.gov/entrez/query.fcgi?cmd=Retrieve&db=pubmed&dopt=Abstract&list_uids=27551764|6|Biomed Pharmacother|83||Aug|2016|JOURNAL ARTICLE|http://www.ncbi.nlm.nih.gov/entrez/query.fcgi?cmd=Retrieve&db=pubmed&dopt=Abstract&list_uids=27573547|6|Int J Oncol|49|3|Sep|2016|Journal Article
akt1	mirn216a	http://www.ncbi.nlm.nih.gov/entrez/query.fcgi?cmd=Retrieve&db=pubmed&dopt=Abstract&list_uids=27578985|6|Onco Targets Ther|9|||2016|Journal Article
akt1	pc-3	http://www.ncbi.nlm.nih.gov/entrez/query.fcgi?cmd=Retrieve&db=pubmed&dopt=Abstract&list_uids=27588482|6|Oncotarget|||Aug|2016|JOURNAL ARTICLE
akt1	mirn222	http://www.ncbi.nlm.nih.gov/entrez/query.fcgi?cmd=Retrieve&db=pubmed&dopt=Abstract&list_uids=27602961|6|Oncotarget|||Sep|2016|JOURNAL ARTICLE
akt1	ppp2r2a	http://www.ncbi.nlm.nih.gov/entrez/query.fcgi?cmd=Retrieve&db=pubmed&dopt=Abstract&list_uids=27602961|6|Oncotarget|||Sep|2016|JOURNAL ARTICLE

pik3ca	mtdh	http://www.ncbi.nlm.nih.gov/entrez/query.fcgi?cmd=Retrieve&db=pubmed&dopt=Abstract&list_uids=27251589|6|Sci Rep|6|||2016|Journal Article
pik3ca	nras	http://www.ncbi.nlm.nih.gov/entrez/query.fcgi?cmd=Retrieve&db=pubmed&dopt=Abstract&list_uids=27251789|6|J Invest Dermatol|||May|2016|JOURNAL ARTICLE
pik3ca	egfr	http://www.ncbi.nlm.nih.gov/entrez/query.fcgi?cmd=Retrieve&db=pubmed&dopt=Abstract&list_uids=27317099|6|J Exp Clin Cancer Res|35|1||2016|Journal Article
pik3ca	il13	http://www.ncbi.nlm.nih.gov/entrez/query.fcgi?cmd=Retrieve&db=pubmed&dopt=Abstract&list_uids=27424080|6|Int Immunopharmacol|39||Oct|2016|Journal Article
pik3ca	chrna4	http://www.ncbi.nlm.nih.gov/entrez/query.fcgi?cmd=Retrieve&db=pubmed&dopt=Abstract&list_uids=27424921|6|Biochim Biophys Acta|1860|11 Pt A|Nov|2016|Journal Article
pik3ca	prdx2	http://www.ncbi.nlm.nih.gov/entrez/query.fcgi?cmd=Retrieve&db=pubmed&dopt=Abstract&list_uids=27507059|6|Oncotarget|||Aug|2016|JOURNAL ARTICLE
pik3ca	hdac9	http://www.ncbi.nlm.nih.gov/entrez/query.fcgi?cmd=Retrieve&db=pubmed&dopt=Abstract&list_uids=27507059|6|Oncotarget|||Aug|2016|JOURNAL ARTICLE
pik3ca	zhx2	http://www.ncbi.nlm.nih.gov/entrez/query.fcgi?cmd=Retrieve&db=pubmed&dopt=Abstract&list_uids=27572273|6|Int J Oncol|49|3|Sep|2016|Journal Article
pik3ca	frap1	http://www.ncbi.nlm.nih.gov/entrez/query.fcgi?cmd=Retrieve&db=pubmed&dopt=Abstract&list_uids=27498786|6|Oncol Rep|||Aug|2016|JOURNAL ARTICLE|http://www.ncbi.nlm.nih.gov/entrez/query.fcgi?cmd=Retrieve&db=pubmed&dopt=Abstract&list_uids=27394418|6|Neurochem Res|||Jul|2016|JOURNAL ARTICLE|http://www.ncbi.nlm.nih.gov/entrez/query.fcgi?cmd=Retrieve&db=pubmed&dopt=Abstract&list_uids=27424080|6|Int Immunopharmacol|39||Oct|2016|Journal Article|http://www.ncbi.nlm.nih.gov/entrez/query.fcgi?cmd=Retrieve&db=pubmed&dopt=Abstract&list_uids=27491275|6|Matrix Biol|||Aug|2016|REVIEW,JOURNAL ARTICLE|http://www.ncbi.nlm.nih.gov/entrez/query.fcgi?cmd=Retrieve&db=pubmed&dopt=Abstract&list_uids=27551764|6|Biomed Pharmacother|83||Aug|2016|JOURNAL ARTICLE|http://www.ncbi.nlm.nih.gov/entrez/query.fcgi?cmd=Retrieve&db=pubmed&dopt=Abstract&list_uids=27507059|6|Oncotarget|||Aug|2016|JOURNAL ARTICLE|http://www.ncbi.nlm.nih.gov/entrez/query.fcgi?cmd=Retrieve&db=pubmed&dopt=Abstract&list_uids=27573547|6|Int J Oncol|49|3|Sep|2016|Journal Article
pik3ca	mirn216a	http://www.ncbi.nlm.nih.gov/entrez/query.fcgi?cmd=Retrieve&db=pubmed&dopt=Abstract&list_uids=27578985|6|Onco Targets Ther|9|||2016|Journal Article
pik3ca	pc-3	http://www.ncbi.nlm.nih.gov/entrez/query.fcgi?cmd=Retrieve&db=pubmed&dopt=Abstract&list_uids=27588482|6|Oncotarget|||Aug|2016|JOURNAL ARTICLE
pik3ca	egf	http://www.ncbi.nlm.nih.gov/entrez/query.fcgi?cmd=Retrieve&db=pubmed&dopt=Abstract&list_uids=27598237|6|Eur J Med Chem|124||Aug|2016|JOURNAL

		ARTICLE|http://www.ncbi.nlm.nih.gov/entrez/query.fcgi?cmd=Retrieve&db=pubmed&dopt=Abstract&list_uids=27470348|6|Biomed Pharmacother|82||Aug|2016|Journal Article
pik3ca	slamf7	http://www.ncbi.nlm.nih.gov/entrez/query.fcgi?cmd=Retrieve&db=pubmed&dopt=Abstract&list_uids=27598237|6|Eur J Med Chem|124||Aug|2016|JOURNAL ARTICLE
pik3ca	akt1	http://www.ncbi.nlm.nih.gov/entrez/query.fcgi?cmd=Retrieve&db=pubmed&dopt=Abstract&list_uids=27496641|6|Mol Cell Endocrinol|436||Aug|2016|JOURNAL ARTICLE|http://www.ncbi.nlm.nih.gov/entrez/query.fcgi?cmd=Retrieve&db=pubmed&dopt=Abstract&list_uids=27498786|6|Oncol Rep|||Aug|2016|JOURNAL ARTICLE|http://www.ncbi.nlm.nih.gov/entrez/query.fcgi?cmd=Retrieve&db=pubmed&dopt=Abstract&list_uids=27394418|6|Neurochem Res|||Jul|2016|JOURNAL ARTICLE|http://www.ncbi.nlm.nih.gov/entrez/query.fcgi?cmd=Retrieve&db=pubmed&dopt=Abstract&list_uids=27569589|6|Life Sci|||Aug|2016|JOURNAL ARTICLE|http://www.ncbi.nlm.nih.gov/entrez/query.fcgi?cmd=Retrieve&db=pubmed&dopt=Abstract&list_uids=27602956|6|Oncotarget|||Sep|2016|JOURNAL ARTICLE|http://www.ncbi.nlm.nih.gov/entrez/query.fcgi?cmd=Retrieve&db=pubmed&dopt=Abstract&list_uids=27489353|6|Oncotarget|||Jul|2016|JOURNAL ARTICLE|http://www.ncbi.nlm.nih.gov/entrez/query.fcgi?cmd=Retrieve&db=pubmed&dopt=Abstract&list_uids=27458096|6|Oncol Res|24|3||2016|Journal Article|http://www.ncbi.nlm.nih.gov/entrez/query.fcgi?cmd=Retrieve&db=pubmed&dopt=Abstract&list_uids=27600360|6|Cell Biol Int|||Sep|2016|JOURNAL ARTICLE|http://www.ncbi.nlm.nih.gov/entrez/query.fcgi?cmd=Retrieve&db=pubmed&dopt=Abstract&list_uids=27572273|6|Int J Oncol|49|3|Sep|2016|Journal Article|http://www.ncbi.nlm.nih.gov/entrez/query.fcgi?cmd=Retrieve&db=pubmed&dopt=Abstract&list_uids=27424921|6|Biochim Biophys Acta|1860|11 Pt A|Nov|2016|Journal Article|http://www.ncbi.nlm.nih.gov/entrez/query.fcgi?cmd=Retrieve&db=pubmed&dopt=Abstract&list_uids=27530117|6|Biomol Ther (Seoul)|||Aug|2016|JOURNAL ARTICLE|http://www.ncbi.nlm.nih.gov/entrez/query.fcgi?cmd=Retrieve&db=pubmed&dopt=Abstract&list_uids=27301070|6|Stem Cells|34|9|Sep|2016|Journal Article|http://www.ncbi.nlm.nih.gov/entrez/query.fcgi?cmd=Retrieve&db=pubmed&dopt=Abstract&list_uids=27573547|6|Int J Oncol|49|3|Sep|2016|Journal Article|http://www.ncbi.nlm.nih.gov/entrez/query.fcgi?cmd=Retrieve&db=pubmed&dopt=Abstract&list_uids=27344565|6|Cell Biol Toxicol|32|5|Oct|2016|Journal Article|http://www.ncbi.nlm.nih.gov/entrez/query.fcgi?cmd=Retrieve&db=pubmed&dopt=Abstract&list_uids=27424080|6|Int Immunopharmacol|39||Oct|2016|Journal Article|http://www.ncbi.nlm.nih.gov/entrez/query.fcgi?cmd=Retrieve&db=pubmed&dopt=Abstract&list_uids=27498977|6|Int J Oncol|||Jul|2016|JOURNAL

		ARTICLE|http://www.ncbi.nlm.nih.gov/entrez/query.fcgi?cmd=Retrieve&db=pubmed&dopt=Abstract&list_uids=27572091|6|Mol Med Rep|||Aug|2016|JOURNAL ARTICLE|http://www.ncbi.nlm.nih.gov/entrez/query.fcgi?cmd=Retrieve&db=pubmed&dopt=Abstract&list_uids=27507059|6|Oncotarget|||Aug|2016|JOURNAL ARTICLE|http://www.ncbi.nlm.nih.gov/entrez/query.fcgi?cmd=Retrieve&db=pubmed&dopt=Abstract&list_uids=27470348|6|Biomed Pharmacother|82||Aug|2016|Journal Article|http://www.ncbi.nlm.nih.gov/entrez/query.fcgi?cmd=Retrieve&db=pubmed&dopt=Abstract&list_uids=27598237|6|Eur J Med Chem|124||Aug|2016|JOURNAL ARTICLE|http://www.ncbi.nlm.nih.gov/entrez/query.fcgi?cmd=Retrieve&db=pubmed&dopt=Abstract&list_uids=27446408|6|Oncol Lett|12|2|Aug|2016|JOURNAL ARTICLE|http://www.ncbi.nlm.nih.gov/entrez/query.fcgi?cmd=Retrieve&db=pubmed&dopt=Abstract&list_uids=27251589|6|Sci Rep|6|||2016|Journal Article|http://www.ncbi.nlm.nih.gov/entrez/query.fcgi?cmd=Retrieve&db=pubmed&dopt=Abstract&list_uids=27303300|6|Front Pharmacol|7|||2016|Journal Article|http://www.ncbi.nlm.nih.gov/entrez/query.fcgi?cmd=Retrieve&db=pubmed&dopt=Abstract&list_uids=27383307|6|Eur Rev Med Pharmacol Sci|20|12|Jun|2016|Journal Article|http://www.ncbi.nlm.nih.gov/entrez/query.fcgi?cmd=Retrieve&db=pubmed&dopt=Abstract&list_uids=27289010|6|Exp Mol Pathol|101|1|Aug|2016|Journal Article|http://www.ncbi.nlm.nih.gov/entrez/query.fcgi?cmd=Retrieve&db=pubmed&dopt=Abstract&list_uids=27251789|6|J Invest Dermatol|||May|2016|JOURNAL ARTICLE|http://www.ncbi.nlm.nih.gov/entrez/query.fcgi?cmd=Retrieve&db=pubmed&dopt=Abstract&list_uids=27578985|6|Onco Targets Ther|9|||2016|Journal Article|http://www.ncbi.nlm.nih.gov/entrez/query.fcgi?cmd=Retrieve&db=pubmed&dopt=Abstract&list_uids=27491275|6|Matrix Biol|||Aug|2016|REVIEW,JOURNAL ARTICLE|http://www.ncbi.nlm.nih.gov/entrez/query.fcgi?cmd=Retrieve&db=pubmed&dopt=Abstract&list_uids=27588482|6|Oncotarget|||Aug|2016|JOURNAL ARTICLE|http://www.ncbi.nlm.nih.gov/entrez/query.fcgi?cmd=Retrieve&db=pubmed&dopt=Abstract&list_uids=27551764|6|Biomed Pharmacother|83||Aug|2016|JOURNAL ARTICLE
sox3	src	http://www.ncbi.nlm.nih.gov/entrez/query.fcgi?cmd=Retrieve&db=pubmed&dopt=Abstract&list_uids=27251670|6|Tumour Biol|||Jun|2016|JOURNAL ARTICLE
nras	mirnlet7e	http://www.ncbi.nlm.nih.gov/entrez/query.fcgi?cmd=Retrieve&db=pubmed&dopt=Abstract&list_uids=27556696|6|Oncotarget|||Aug|2016|JOURNAL ARTICLE
mmp14	vegfa	http://www.ncbi.nlm.nih.gov/entrez/query.fcgi?cmd=Retrieve&db=pubmed&dopt=Abstract&list_uids=27491275|6|Matrix Biol|||Aug|2016|REVIEW,JOURNAL ARTICLE
mmp14	mmp13	http://www.ncbi.nlm.nih.gov/entrez/query.fcgi?cmd=Retrieve&db=pubmed&dopt=Abstract&list_uids=27600927|6|J Dermatol Sci|||Jun|2016|JOURNAL ARTICLE

mmp14	mmp2	http://www.ncbi.nlm.nih.gov/entrez/query.fcgi?cmd=Retrieve&db=pubmed&dopt=Abstract&list_uids=27600927|6|J Dermatol Sci|||Jun|2016|JOURNAL ARTICLE|http://www.ncbi.nlm.nih.gov/entrez/query.fcgi?cmd=Retrieve&db=pubmed&dopt=Abstract&list_uids=27613828|6|Oncotarget|||Aug|2016|JOURNAL ARTICLE
alpl	mmp14	http://www.ncbi.nlm.nih.gov/entrez/query.fcgi?cmd=Retrieve&db=pubmed&dopt=Abstract&list_uids=27255086|6|EMBO Rep|17|7|Jul|2016|Journal Article
vps39	mmp14	http://www.ncbi.nlm.nih.gov/entrez/query.fcgi?cmd=Retrieve&db=pubmed&dopt=Abstract&list_uids=27255086|6|EMBO Rep|17|7|Jul|2016|Journal Article
vps39	alpl	http://www.ncbi.nlm.nih.gov/entrez/query.fcgi?cmd=Retrieve&db=pubmed&dopt=Abstract&list_uids=27255086|6|EMBO Rep|17|7|Jul|2016|Journal Article
rab2a	mmp14	http://www.ncbi.nlm.nih.gov/entrez/query.fcgi?cmd=Retrieve&db=pubmed&dopt=Abstract&list_uids=27255086|6|EMBO Rep|17|7|Jul|2016|Journal Article
agfg1	rab2a	http://www.ncbi.nlm.nih.gov/entrez/query.fcgi?cmd=Retrieve&db=pubmed&dopt=Abstract&list_uids=27255086|6|EMBO Rep|17|7|Jul|2016|Journal Article
birc3	hspa5	http://www.ncbi.nlm.nih.gov/entrez/query.fcgi?cmd=Retrieve&db=pubmed&dopt=Abstract&list_uids=27256049|6|Zhonghua Bing Li Xue Za Zhi|45|6|Jun|2016|English Abstract,Journal Article
frap1	hspa5	http://www.ncbi.nlm.nih.gov/entrez/query.fcgi?cmd=Retrieve&db=pubmed&dopt=Abstract&list_uids=27256049|6|Zhonghua Bing Li Xue Za Zhi|45|6|Jun|2016|English Abstract,Journal Article
frap1	birc3	http://www.ncbi.nlm.nih.gov/entrez/query.fcgi?cmd=Retrieve&db=pubmed&dopt=Abstract&list_uids=27256049|6|Zhonghua Bing Li Xue Za Zhi|45|6|Jun|2016|English Abstract,Journal Article
frap1	atp8a2	http://www.ncbi.nlm.nih.gov/entrez/query.fcgi?cmd=Retrieve&db=pubmed&dopt=Abstract&list_uids=27335323|6|Open Biol|6|6|Jun|2016|Journal Article
frap1	ccr5	http://www.ncbi.nlm.nih.gov/entrez/query.fcgi?cmd=Retrieve&db=pubmed&dopt=Abstract&list_uids=27335323|6|Open Biol|6|6|Jun|2016|Journal Article
frap1	snord87	http://www.ncbi.nlm.nih.gov/entrez/query.fcgi?cmd=Retrieve&db=pubmed&dopt=Abstract&list_uids=27394418|6|Neurochem Res|||Jul|2016|JOURNAL ARTICLE
frap1	mapk14	http://www.ncbi.nlm.nih.gov/entrez/query.fcgi?cmd=Retrieve&db=pubmed&dopt=Abstract&list_uids=27394418|6|Neurochem Res|||Jul|2016|JOURNAL ARTICLE
frap1	il13	http://www.ncbi.nlm.nih.gov/entrez/query.fcgi?cmd=Retrieve&db=pubmed&dopt=Abstract&list_uids=27424080|6|Int Immunopharmacol|39||Oct|2016|Journal Article
frap1	tp53	http://www.ncbi.nlm.nih.gov/entrez/query.fcgi?cmd=Retrieve&db=pubmed&dopt=Abstract&list_uids=27499479|6|Int J Oncol|||Aug|2016|JOURNAL ARTICLE
frap1	prdx2	http://www.ncbi.nlm.nih.gov/entrez/query.fcgi?cmd=Retrieve&db=pubmed&dopt=Abstract&list_uids=27507059|6|Oncotarget|||Aug|2016|JOURNAL ARTICLE
frap1	hdac9	http://www.ncbi.nlm.nih.gov/entrez/query.fcgi?cmd=Retrieve&db=pubmed&dopt=Abstract&list_uids=27507059|6|Oncotarget|||Aug|2016|JOURNAL ARTICLE

cdkn2b	hspa5	http://www.ncbi.nlm.nih.gov/entrez/query.fcgi?cmd=Retrieve&db=pubmed&dopt=Abstract&list_uids=27256049|6|Zhonghua Bing Li Xue Za Zhi|45|6|Jun|2016|English Abstract,Journal Article
cdkn2b	birc3	http://www.ncbi.nlm.nih.gov/entrez/query.fcgi?cmd=Retrieve&db=pubmed&dopt=Abstract&list_uids=27256049|6|Zhonghua Bing Li Xue Za Zhi|45|6|Jun|2016|English Abstract,Journal Article
cdkn2b	frap1	http://www.ncbi.nlm.nih.gov/entrez/query.fcgi?cmd=Retrieve&db=pubmed&dopt=Abstract&list_uids=27256049|6|Zhonghua Bing Li Xue Za Zhi|45|6|Jun|2016|English Abstract,Journal Article
met	egr1	http://www.ncbi.nlm.nih.gov/entrez/query.fcgi?cmd=Retrieve&db=pubmed&dopt=Abstract&list_uids=27530187|6|Hepatology|||Aug|2016|JOURNAL ARTICLE
met	akt1	http://www.ncbi.nlm.nih.gov/entrez/query.fcgi?cmd=Retrieve&db=pubmed&dopt=Abstract&list_uids=27530187|6|Hepatology|||Aug|2016|JOURNAL ARTICLE
met	ephb2	http://www.ncbi.nlm.nih.gov/entrez/query.fcgi?cmd=Retrieve&db=pubmed&dopt=Abstract&list_uids=27530187|6|Hepatology|||Aug|2016|JOURNAL ARTICLE
sardh	met	http://www.ncbi.nlm.nih.gov/entrez/query.fcgi?cmd=Retrieve&db=pubmed&dopt=Abstract&list_uids=27258622|6|Eur J Med Chem|118||Apr|2016|JOURNAL ARTICLE
mzf1	mmp14	http://www.ncbi.nlm.nih.gov/entrez/query.fcgi?cmd=Retrieve&db=pubmed&dopt=Abstract&list_uids=27259238|6|Oncotarget|||May|2016|JOURNAL ARTICLE
snai2	nono	http://www.ncbi.nlm.nih.gov/entrez/query.fcgi?cmd=Retrieve&db=pubmed&dopt=Abstract&list_uids=27259250|6|Oncotarget|||May|2016|JOURNAL ARTICLE
snai2	igfbp7	http://www.ncbi.nlm.nih.gov/entrez/query.fcgi?cmd=Retrieve&db=pubmed&dopt=Abstract&list_uids=27259250|6|Oncotarget|||May|2016|JOURNAL ARTICLE
igfbp7	nono	http://www.ncbi.nlm.nih.gov/entrez/query.fcgi?cmd=Retrieve&db=pubmed&dopt=Abstract&list_uids=27259250|6|Oncotarget|||May|2016|JOURNAL ARTICLE
ptbp1	nono	http://www.ncbi.nlm.nih.gov/entrez/query.fcgi?cmd=Retrieve&db=pubmed&dopt=Abstract&list_uids=27259250|6|Oncotarget|||May|2016|JOURNAL ARTICLE
ptbp1	igfbp7	http://www.ncbi.nlm.nih.gov/entrez/query.fcgi?cmd=Retrieve&db=pubmed&dopt=Abstract&list_uids=27259250|6|Oncotarget|||May|2016|JOURNAL ARTICLE
il8	hm13	http://www.ncbi.nlm.nih.gov/entrez/query.fcgi?cmd=Retrieve&db=pubmed&dopt=Abstract&list_uids=27337492|6|J Appl Microbiol|121|3|Sep|2016|Journal Article
il8	stat3	http://www.ncbi.nlm.nih.gov/entrez/query.fcgi?cmd=Retrieve&db=pubmed&dopt=Abstract&list_uids=27391430|6|Oncotarget|||Jul|2016|JOURNAL ARTICLE
il8	mmp9	http://www.ncbi.nlm.nih.gov/entrez/query.fcgi?cmd=Retrieve&db=pubmed&dopt=Abstract&list_uids=27398151|6|Am J Transl Res|8|6||2016|Journal Article
il8	notch1	http://www.ncbi.nlm.nih.gov/entrez/query.fcgi?cmd=Retrieve&db=pubmed&dopt=Abstract&list_uids=27398151|6|Am J Transl Res|8|6||2016|Journal Article
il8	vegfa	http://www.ncbi.nlm.nih.gov/entrez/query.fcgi?cmd=Retrieve&db=pubmed&dopt=Abstract&list_uids=27391430|6|Oncotarget|||Jul|2016|JOURNAL

		ARTICLE|http://www.ncbi.nlm.nih.gov/entrez/query.fcgi?cmd=Retrieve&db=pubmed&dopt=Abstract&list_uids=27398151|6|Am J Transl Res|8|6||2016|Journal Article
il8	il10	http://www.ncbi.nlm.nih.gov/entrez/query.fcgi?cmd=Retrieve&db=pubmed&dopt=Abstract&list_uids=27513864|6|PLoS One|11|8||2016|Journal Article
il8	vcan	http://www.ncbi.nlm.nih.gov/entrez/query.fcgi?cmd=Retrieve&db=pubmed&dopt=Abstract&list_uids=27513864|6|PLoS One|11|8||2016|Journal Article
il8	cd80	http://www.ncbi.nlm.nih.gov/entrez/query.fcgi?cmd=Retrieve&db=pubmed&dopt=Abstract&list_uids=27513864|6|PLoS One|11|8||2016|Journal Article
il8	il6	http://www.ncbi.nlm.nih.gov/entrez/query.fcgi?cmd=Retrieve&db=pubmed&dopt=Abstract&list_uids=27558278|6|J Periodontal Res|||Aug|2016|JOURNAL ARTICLE|http://www.ncbi.nlm.nih.gov/entrez/query.fcgi?cmd=Retrieve&db=pubmed&dopt=Abstract&list_uids=27312487|6|Tumour Biol|||Jun|2016|JOURNAL ARTICLE
il8	lepr	http://www.ncbi.nlm.nih.gov/entrez/query.fcgi?cmd=Retrieve&db=pubmed&dopt=Abstract&list_uids=27588409|6|Oncotarget|||Aug|2016|JOURNAL ARTICLE
il8	mapk14	http://www.ncbi.nlm.nih.gov/entrez/query.fcgi?cmd=Retrieve&db=pubmed&dopt=Abstract&list_uids=27588409|6|Oncotarget|||Aug|2016|JOURNAL ARTICLE
il8	ephb2	http://www.ncbi.nlm.nih.gov/entrez/query.fcgi?cmd=Retrieve&db=pubmed&dopt=Abstract&list_uids=27588409|6|Oncotarget|||Aug|2016|JOURNAL ARTICLE
usp21	il8	http://www.ncbi.nlm.nih.gov/entrez/query.fcgi?cmd=Retrieve&db=pubmed&dopt=Abstract&list_uids=27259257|6|Oncotarget|||May|2016|JOURNAL ARTICLE
usp21	xrcc1	http://www.ncbi.nlm.nih.gov/entrez/query.fcgi?cmd=Retrieve&db=pubmed&dopt=Abstract&list_uids=27259257|6|Oncotarget|||May|2016|JOURNAL ARTICLE
xrcc1	mirn182	http://www.ncbi.nlm.nih.gov/entrez/query.fcgi?cmd=Retrieve&db=pubmed&dopt=Abstract&list_uids=27468875|6|Neoplasma|63|5|Jul|2016|JOURNAL ARTICLE
vegfa	egfr	http://www.ncbi.nlm.nih.gov/entrez/query.fcgi?cmd=Retrieve&db=pubmed&dopt=Abstract&list_uids=27286795|6|Neuro Oncol|||Jun|2016|JOURNAL ARTICLE
vegfa	mapk3	http://www.ncbi.nlm.nih.gov/entrez/query.fcgi?cmd=Retrieve&db=pubmed&dopt=Abstract&list_uids=27297262|6|Clin Exp Pharmacol Physiol|43|10|Oct|2016|Journal Article
vegfa	ptk2	http://www.ncbi.nlm.nih.gov/entrez/query.fcgi?cmd=Retrieve&db=pubmed&dopt=Abstract&list_uids=27297262|6|Clin Exp Pharmacol Physiol|43|10|Oct|2016|Journal Article
vegfa	src	http://www.ncbi.nlm.nih.gov/entrez/query.fcgi?cmd=Retrieve&db=pubmed&dopt=Abstract&list_uids=27297262|6|Clin Exp Pharmacol Physiol|43|10|Oct|2016|Journal Article|http://www.ncbi.nlm.nih.gov/entrez/query.fcgi?cmd=Retrieve&db=pubmed&dopt=Abstract&list_uids=27286795|6|Neuro Oncol|||Jun|2016|JOURNAL ARTICLE
vegfa	il17a	http://www.ncbi.nlm.nih.gov/entrez/query.fcgi?cmd=Retrieve&db=pubmed&dopt=Abstract&list_uids=27353107|6|Zhonghua Wei Chang Wai Ke Za Zhi|19|6|Jun|2016|English Abstract,Journal Article

vegfa	mmp9	http://www.ncbi.nlm.nih.gov/entrez/query.fcgi?cmd=Retrieve&db=pubmed&dopt=Abstract&list_uids=27398151|6|Am J Transl Res|8|6||2016|Journal Article|http://www.ncbi.nlm.nih.gov/entrez/query.fcgi?cmd=Retrieve&db=pubmed&dopt=Abstract&list_uids=27353107|6|Zhonghua Wei Chang Wai Ke Za Zhi|19|6|Jun|2016|English Abstract,Journal Article
vegfa	notch1	http://www.ncbi.nlm.nih.gov/entrez/query.fcgi?cmd=Retrieve&db=pubmed&dopt=Abstract&list_uids=27398151|6|Am J Transl Res|8|6||2016|Journal Article
vegfa	hccs	http://www.ncbi.nlm.nih.gov/entrez/query.fcgi?cmd=Retrieve&db=pubmed&dopt=Abstract&list_uids=27449032|6|Tumour Biol|||Jul|2016|JOURNAL ARTICLE
vegfa	akt1	http://www.ncbi.nlm.nih.gov/entrez/query.fcgi?cmd=Retrieve&db=pubmed&dopt=Abstract&list_uids=27297262|6|Clin Exp Pharmacol Physiol|43|10|Oct|2016|Journal Article|http://www.ncbi.nlm.nih.gov/entrez/query.fcgi?cmd=Retrieve&db=pubmed&dopt=Abstract&list_uids=27507763|6|Sci Rep|6|||2016|Journal Article
vegfa	ephb2	http://www.ncbi.nlm.nih.gov/entrez/query.fcgi?cmd=Retrieve&db=pubmed&dopt=Abstract&list_uids=27569654|6|Cancer Lett|382|1|Aug|2016|JOURNAL ARTICLE
hk2	vegfa	http://www.ncbi.nlm.nih.gov/entrez/query.fcgi?cmd=Retrieve&db=pubmed&dopt=Abstract&list_uids=27259263|6|Oncotarget|||Jun|2016|JOURNAL ARTICLE
jak2	ptpn6	http://www.ncbi.nlm.nih.gov/entrez/query.fcgi?cmd=Retrieve&db=pubmed&dopt=Abstract&list_uids=27259268|6|Oncotarget|||Jun|2016|JOURNAL ARTICLE
jak2	trim29	http://www.ncbi.nlm.nih.gov/entrez/query.fcgi?cmd=Retrieve&db=pubmed&dopt=Abstract&list_uids=27430345|6|Oncol Rep|36|3|Sep|2016|Journal Article
jak2	stat3	http://www.ncbi.nlm.nih.gov/entrez/query.fcgi?cmd=Retrieve&db=pubmed&dopt=Abstract&list_uids=27471397|6|Onco Targets Ther|9|||2016|Journal Article|http://www.ncbi.nlm.nih.gov/entrez/query.fcgi?cmd=Retrieve&db=pubmed&dopt=Abstract&list_uids=27430345|6|Oncol Rep|36|3|Sep|2016|Journal Article|http://www.ncbi.nlm.nih.gov/entrez/query.fcgi?cmd=Retrieve&db=pubmed&dopt=Abstract&list_uids=27259268|6|Oncotarget|||Jun|2016|JOURNAL ARTICLE
jak1	ptpn6	http://www.ncbi.nlm.nih.gov/entrez/query.fcgi?cmd=Retrieve&db=pubmed&dopt=Abstract&list_uids=27259268|6|Oncotarget|||Jun|2016|JOURNAL ARTICLE
jak1	jak2	http://www.ncbi.nlm.nih.gov/entrez/query.fcgi?cmd=Retrieve&db=pubmed&dopt=Abstract&list_uids=27259268|6|Oncotarget|||Jun|2016|JOURNAL ARTICLE
jak1	stat3	http://www.ncbi.nlm.nih.gov/entrez/query.fcgi?cmd=Retrieve&db=pubmed&dopt=Abstract&list_uids=27292026|6|Neoplasia|18|6|Jun|2016|Journal Article|http://www.ncbi.nlm.nih.gov/entrez/query.fcgi?cmd=Retrieve&db=pubmed&dopt=Abstract&list_uids=27259268|6|Oncotarget|||Jun|2016|JOURNAL ARTICLE
jak1	src	http://www.ncbi.nlm.nih.gov/entrez/query.fcgi?cmd=Retrieve&db=pubmed&dopt=Abstract&list_uids=27458171|6|Oncotarget|||Jul|2016|JOURNAL ARTICLE

stat3	ptpn6	http://www.ncbi.nlm.nih.gov/entrez/query.fcgi?cmd=Retrieve&db=pubmed&dopt=Abstract&list_uids=27259268|6|Oncotarget|||Jun|2016|JOURNAL ARTICLE
stat3	stat6	http://www.ncbi.nlm.nih.gov/entrez/query.fcgi?cmd=Retrieve&db=pubmed&dopt=Abstract&list_uids=27367025|6|Oncotarget|||Jun|2016|JOURNAL ARTICLE
stat3	il10	http://www.ncbi.nlm.nih.gov/entrez/query.fcgi?cmd=Retrieve&db=pubmed&dopt=Abstract&list_uids=27384401|6|Sci Rep|6|||2016|Journal Article
stat3	bcl2	http://www.ncbi.nlm.nih.gov/entrez/query.fcgi?cmd=Retrieve&db=pubmed&dopt=Abstract&list_uids=27415751|6|Cancer Biol Ther|||Jul|2016|JOURNAL ARTICLE
stat3	bcl2l1	http://www.ncbi.nlm.nih.gov/entrez/query.fcgi?cmd=Retrieve&db=pubmed&dopt=Abstract&list_uids=27415751|6|Cancer Biol Ther|||Jul|2016|JOURNAL ARTICLE
stat3	myc	http://www.ncbi.nlm.nih.gov/entrez/query.fcgi?cmd=Retrieve&db=pubmed&dopt=Abstract&list_uids=27415751|6|Cancer Biol Ther|||Jul|2016|JOURNAL ARTICLE
stat3	trim29	http://www.ncbi.nlm.nih.gov/entrez/query.fcgi?cmd=Retrieve&db=pubmed&dopt=Abstract&list_uids=27430345|6|Oncol Rep|36|3|Sep|2016|Journal Article
stat3	ddr1	http://www.ncbi.nlm.nih.gov/entrez/query.fcgi?cmd=Retrieve&db=pubmed&dopt=Abstract&list_uids=27458446|6|Front Microbiol|7|||2016|Journal Article,Review
stat3	vegfa	http://www.ncbi.nlm.nih.gov/entrez/query.fcgi?cmd=Retrieve&db=pubmed&dopt=Abstract&list_uids=27391430|6|Oncotarget|||Jul|2016|JOURNAL ARTICLE|http://www.ncbi.nlm.nih.gov/entrez/query.fcgi?cmd=Retrieve&db=pubmed&dopt=Abstract&list_uids=27494878|6|Oncotarget|||Aug|2016|JOURNAL ARTICLE|http://www.ncbi.nlm.nih.gov/entrez/query.fcgi?cmd=Retrieve&db=pubmed&dopt=Abstract&list_uids=27415751|6|Cancer Biol Ther|||Jul|2016|JOURNAL ARTICLE|http://www.ncbi.nlm.nih.gov/entrez/query.fcgi?cmd=Retrieve&db=pubmed&dopt=Abstract&list_uids=27307601|6|Mol Cancer Ther|15|9|Sep|2016|Journal Article|http://www.ncbi.nlm.nih.gov/entrez/query.fcgi?cmd=Retrieve&db=pubmed&dopt=Abstract&list_uids=27461244|6|Oncol Rep|36|3|Sep|2016|Journal Article|http://www.ncbi.nlm.nih.gov/entrez/query.fcgi?cmd=Retrieve&db=pubmed&dopt=Abstract&list_uids=27353107|6|Zhonghua Wei Chang Wai Ke Za Zhi|19|6|Jun|2016|English Abstract,Journal Article
stat3	mmp1	http://www.ncbi.nlm.nih.gov/entrez/query.fcgi?cmd=Retrieve&db=pubmed&dopt=Abstract&list_uids=27566200|6|J Ethnopharmacol|||Aug|2016|JOURNAL ARTICLE
stat3	itk	http://www.ncbi.nlm.nih.gov/entrez/query.fcgi?cmd=Retrieve&db=pubmed&dopt=Abstract&list_uids=27307601|6|Mol Cancer Ther|15|9|Sep|2016|Journal Article|http://www.ncbi.nlm.nih.gov/entrez/query.fcgi?cmd=Retrieve&db=pubmed&dopt=Abstract&list_uids=27576314|6|J Exp Clin Cancer Res|35|1||2016|Journal Article
stat3	akt1	http://www.ncbi.nlm.nih.gov/entrez/query.fcgi?cmd=Retrieve&db=pubmed&dopt=Abstract&list_uids=27599909|6|Mol Med Rep|||Sep|2016|JOURNAL ARTICLE

stat3	hcc	http://www.ncbi.nlm.nih.gov/entrez/query.fcgi?cmd=Retrieve&db=pubmed&dopt=Abstract&list_uids=27608898|6|Eur Rev Med Pharmacol Sci|20|16|Aug|2016|Journal Article|http://www.ncbi.nlm.nih.gov/entrez/query.fcgi?cmd=Retrieve&db=pubmed&dopt=Abstract&list_uids=27461244|6|Oncol Rep|36|3|Sep|2016|Journal Article|http://www.ncbi.nlm.nih.gov/entrez/query.fcgi?cmd=Retrieve&db=pubmed&dopt=Abstract&list_uids=27292026|6|Neoplasia|18|6|Jun|2016|Journal Article
ephb2	mmp13	http://www.ncbi.nlm.nih.gov/entrez/query.fcgi?cmd=Retrieve&db=pubmed&dopt=Abstract&list_uids=27356745|6|Oncotarget|||Jun|2016|JOURNAL ARTICLE
ephb2	hdac9	http://www.ncbi.nlm.nih.gov/entrez/query.fcgi?cmd=Retrieve&db=pubmed&dopt=Abstract&list_uids=27418645|6|Mol Cancer Res|||Jul|2016|JOURNAL ARTICLE
ephb2	egr1	http://www.ncbi.nlm.nih.gov/entrez/query.fcgi?cmd=Retrieve&db=pubmed&dopt=Abstract&list_uids=27530187|6|Hepatology|||Aug|2016|JOURNAL ARTICLE
ephb2	rela	http://www.ncbi.nlm.nih.gov/entrez/query.fcgi?cmd=Retrieve&db=pubmed&dopt=Abstract&list_uids=27533779|6|Mol Carcinog|||Aug|2016|JOURNAL ARTICLE
ephb2	jag1	http://www.ncbi.nlm.nih.gov/entrez/query.fcgi?cmd=Retrieve&db=pubmed&dopt=Abstract&list_uids=27542217|6|Oncotarget|||Aug|2016|JOURNAL ARTICLE
ephb2	sgcb	http://www.ncbi.nlm.nih.gov/entrez/query.fcgi?cmd=Retrieve&db=pubmed&dopt=Abstract&list_uids=27542217|6|Oncotarget|||Aug|2016|JOURNAL ARTICLE
ephb2	adamts6	http://www.ncbi.nlm.nih.gov/entrez/query.fcgi?cmd=Retrieve&db=pubmed&dopt=Abstract&list_uids=27542224|6|Oncotarget|||Aug|2016|JOURNAL ARTICLE
ephb2	itk	http://www.ncbi.nlm.nih.gov/entrez/query.fcgi?cmd=Retrieve&db=pubmed&dopt=Abstract&list_uids=27481258|6|Lasers Med Sci|||Aug|2016|JOURNAL ARTICLE|http://www.ncbi.nlm.nih.gov/entrez/query.fcgi?cmd=Retrieve&db=pubmed&dopt=Abstract&list_uids=27562816|6|Cell Physiol Biochem|39|3||2016|Journal Article|http://www.ncbi.nlm.nih.gov/entrez/query.fcgi?cmd=Retrieve&db=pubmed&dopt=Abstract&list_uids=27409670|6|Oncotarget|||Jul|2016|JOURNAL ARTICLE|http://www.ncbi.nlm.nih.gov/entrez/query.fcgi?cmd=Retrieve&db=pubmed&dopt=Abstract&list_uids=27507215|6|Biochem Biophys Res Commun|478|2|Sep|2016|Journal Article
ephb2	foxc2	http://www.ncbi.nlm.nih.gov/entrez/query.fcgi?cmd=Retrieve&db=pubmed&dopt=Abstract&list_uids=27562816|6|Cell Physiol Biochem|39|3||2016|Journal Article
ephb2	akt1	http://www.ncbi.nlm.nih.gov/entrez/query.fcgi?cmd=Retrieve&db=pubmed&dopt=Abstract&list_uids=27573026|6|Int J Oncol|49|3|Sep|2016|Journal Article|http://www.ncbi.nlm.nih.gov/entrez/query.fcgi?cmd=Retrieve&db=pubmed&dopt=Abstract&list_uids=27303300|6|Front Pharmacol|7|||2016|Journal Article|http://www.ncbi.nlm.nih.gov/entrez/query.fcgi?cmd=Retrieve&db=pubmed&dopt=Abstract&list_uids=27572273|6|Int J Oncol|49|3|Sep|2016|Journal

		Article|http://www.ncbi.nlm.nih.gov/entrez/query.fcgi?cmd=Retrieve&db=pubmed&dopt=Abstract&list_uids=27562816|6|Cell Physiol Biochem|39|3||2016|Journal Article|http://www.ncbi.nlm.nih.gov/entrez/query.fcgi?cmd=Retrieve&db=pubmed&dopt=Abstract&list_uids=27507215|6|Biochem Biophys Res Commun|478|2|Sep|2016|Journal Article|http://www.ncbi.nlm.nih.gov/entrez/query.fcgi?cmd=Retrieve&db=pubmed&dopt=Abstract&list_uids=27557506|6|Oncotarget|||Aug|2016|JOURNAL ARTICLE|http://www.ncbi.nlm.nih.gov/entrez/query.fcgi?cmd=Retrieve&db=pubmed&dopt=Abstract&list_uids=27530187|6|Hepatology|||Aug|2016|JOURNAL ARTICLE|http://www.ncbi.nlm.nih.gov/entrez/query.fcgi?cmd=Retrieve&db=pubmed&dopt=Abstract&list_uids=27374177|6|Oncotarget|||Jun|2016|JOURNAL ARTICLE|http://www.ncbi.nlm.nih.gov/entrez/query.fcgi?cmd=Retrieve&db=pubmed&dopt=Abstract&list_uids=27573547|6|Int J Oncol|49|3|Sep|2016|Journal Article
ephb2	pik3ca	http://www.ncbi.nlm.nih.gov/entrez/query.fcgi?cmd=Retrieve&db=pubmed&dopt=Abstract&list_uids=27303300|6|Front Pharmacol|7|||2016|Journal Article|http://www.ncbi.nlm.nih.gov/entrez/query.fcgi?cmd=Retrieve&db=pubmed&dopt=Abstract&list_uids=27572273|6|Int J Oncol|49|3|Sep|2016|Journal Article|http://www.ncbi.nlm.nih.gov/entrez/query.fcgi?cmd=Retrieve&db=pubmed&dopt=Abstract&list_uids=27573547|6|Int J Oncol|49|3|Sep|2016|Journal Article
ephb2	mapk14	http://www.ncbi.nlm.nih.gov/entrez/query.fcgi?cmd=Retrieve&db=pubmed&dopt=Abstract&list_uids=27278736|6|Int J Oncol|49|2|Aug|2016|Journal Article|http://www.ncbi.nlm.nih.gov/entrez/query.fcgi?cmd=Retrieve&db=pubmed&dopt=Abstract&list_uids=27507215|6|Biochem Biophys Res Commun|478|2|Sep|2016|Journal Article|http://www.ncbi.nlm.nih.gov/entrez/query.fcgi?cmd=Retrieve&db=pubmed&dopt=Abstract&list_uids=27588409|6|Oncotarget|||Aug|2016|JOURNAL ARTICLE|http://www.ncbi.nlm.nih.gov/entrez/query.fcgi?cmd=Retrieve&db=pubmed&dopt=Abstract&list_uids=27476935|6|Biol Pharm Bull|39|8||2016|Journal Article|http://www.ncbi.nlm.nih.gov/entrez/query.fcgi?cmd=Retrieve&db=pubmed&dopt=Abstract&list_uids=27274278|6|Onco Targets Ther|9|||2016|Journal Article
ephb2	frap1	http://www.ncbi.nlm.nih.gov/entrez/query.fcgi?cmd=Retrieve&db=pubmed&dopt=Abstract&list_uids=27589685|6|Oncotarget|||Aug|2016|JOURNAL ARTICLE|http://www.ncbi.nlm.nih.gov/entrez/query.fcgi?cmd=Retrieve&db=pubmed&dopt=Abstract&list_uids=27573547|6|Int J Oncol|49|3|Sep|2016|Journal Article
ephb2	myc	http://www.ncbi.nlm.nih.gov/entrez/query.fcgi?cmd=Retrieve&db=pubmed&dopt=Abstract&list_uids=27601158|6|Int J Oncol|||Aug|2016|JOURNAL ARTICLE
ephb2	mmp9	http://www.ncbi.nlm.nih.gov/entrez/query.fcgi?cmd=Retrieve&db=pubmed&dopt=Abstract&list_uids=27573026|6|Int J Oncol|49|3|Sep|2016|Journal

		Article|http://www.ncbi.nlm.nih.gov/entrez/query.fcgi?cmd=Retrieve&db=pubmed&dopt=Abstract&list_uids=27481258|6|Lasers Med Sci|||Aug|2016|JOURNAL ARTICLE|http://www.ncbi.nlm.nih.gov/entrez/query.fcgi?cmd=Retrieve&db=pubmed&dopt=Abstract&list_uids=27601158|6|Int J Oncol|||Aug|2016|JOURNAL ARTICLE
gpr160	ephb2	http://www.ncbi.nlm.nih.gov/entrez/query.fcgi?cmd=Retrieve&db=pubmed&dopt=Abstract&list_uids=27259691|6|Cell Signal|28|9|Sep|2016|Journal Article
gpr160	gli2	http://www.ncbi.nlm.nih.gov/entrez/query.fcgi?cmd=Retrieve&db=pubmed&dopt=Abstract&list_uids=27259691|6|Cell Signal|28|9|Sep|2016|Journal Article
mapk3	ephb2	http://www.ncbi.nlm.nih.gov/entrez/query.fcgi?cmd=Retrieve&db=pubmed&dopt=Abstract&list_uids=27259691|6|Cell Signal|28|9|Sep|2016|Journal Article
mapk3	gpr160	http://www.ncbi.nlm.nih.gov/entrez/query.fcgi?cmd=Retrieve&db=pubmed&dopt=Abstract&list_uids=27259691|6|Cell Signal|28|9|Sep|2016|Journal Article
mapk3	mapk7	http://www.ncbi.nlm.nih.gov/entrez/query.fcgi?cmd=Retrieve&db=pubmed&dopt=Abstract&list_uids=27279438|6|Oncol Rep|36|2|Aug|2016|Journal Article
mapk3	kdr	http://www.ncbi.nlm.nih.gov/entrez/query.fcgi?cmd=Retrieve&db=pubmed&dopt=Abstract&list_uids=27297262|6|Clin Exp Pharmacol Physiol|43|10|Oct|2016|Journal Article
mapk3	arf6	http://www.ncbi.nlm.nih.gov/entrez/query.fcgi?cmd=Retrieve&db=pubmed&dopt=Abstract&list_uids=27320871|6|Nan Fang Yi Ke Da Xue Xue Bao|36|6|Jun|2016|English Abstract,Journal Article,Research Support, Non-U.S. Gov't
mapk3	rac1	http://www.ncbi.nlm.nih.gov/entrez/query.fcgi?cmd=Retrieve&db=pubmed&dopt=Abstract&list_uids=27320871|6|Nan Fang Yi Ke Da Xue Xue Bao|36|6|Jun|2016|English Abstract,Journal Article,Research Support, Non-U.S. Gov't
mapk3	commd7	http://www.ncbi.nlm.nih.gov/entrez/query.fcgi?cmd=Retrieve&db=pubmed&dopt=Abstract&list_uids=27350032|6|Mol Carcinog|||Jun|2016|JOURNAL ARTICLE
mapk3	rela	http://www.ncbi.nlm.nih.gov/entrez/query.fcgi?cmd=Retrieve&db=pubmed&dopt=Abstract&list_uids=27393618|6|Breast Cancer Res Treat|158|3|Aug|2016|Journal Article
mapk3	nf-kappab	http://www.ncbi.nlm.nih.gov/entrez/query.fcgi?cmd=Retrieve&db=pubmed&dopt=Abstract&list_uids=27393618|6|Breast Cancer Res Treat|158|3|Aug|2016|Journal Article
mapk3	ptk2	http://www.ncbi.nlm.nih.gov/entrez/query.fcgi?cmd=Retrieve&db=pubmed&dopt=Abstract&list_uids=27297262|6|Clin Exp Pharmacol Physiol|43|10|Oct|2016|Journal Article|http://www.ncbi.nlm.nih.gov/entrez/query.fcgi?cmd=Retrieve&db=pubmed&dopt=Abstract&list_uids=27422337|6|Bioorg Med Chem Lett|26|16|Aug|2016|Journal Article
mapk3	src	http://www.ncbi.nlm.nih.gov/entrez/query.fcgi?cmd=Retrieve&db=pubmed&dopt=Abstract&list_uids=27297262|6|Clin Exp Pharmacol Physiol|43|10|Oct|2016|Journal Article|http://www.ncbi.nlm.nih.gov/entrez/query.fcgi?cmd=Retrieve&db=pubmed&dopt=Abstract&list_uids=27422337|6|Bioorg Med Chem Lett|26|16|Aug|2016|Journal Article
mapk3	egf	http://www.ncbi.nlm.nih.gov/entrez/query.fcgi?cmd=Retrieve&db=pubmed&dopt=Abstract&list_uids=27470348|6|Biomed Pharmacother|82||Aug|2016|Journal Article

mapk3	pik3ca	http://www.ncbi.nlm.nih.gov/entrez/query.fcgi?cmd=Retrieve&db=pubmed&dopt=Abstract&list_uids=27470348|6|Biomed Pharmacother|82||Aug|2016|Journal Article
mapk3	plau	http://www.ncbi.nlm.nih.gov/entrez/query.fcgi?cmd=Retrieve&db=pubmed&dopt=Abstract&list_uids=27527140|6|Molecules|21|8||2016|Journal Article
mapk3	mmp2	http://www.ncbi.nlm.nih.gov/entrez/query.fcgi?cmd=Retrieve&db=pubmed&dopt=Abstract&list_uids=27322511|6|Environ Toxicol|||Jun|2016|JOURNAL ARTICLE|http://www.ncbi.nlm.nih.gov/entrez/query.fcgi?cmd=Retrieve&db=pubmed&dopt=Abstract&list_uids=27527140|6|Molecules|21|8||2016|Journal Article|http://www.ncbi.nlm.nih.gov/entrez/query.fcgi?cmd=Retrieve&db=pubmed&dopt=Abstract&list_uids=27470348|6|Biomed Pharmacother|82||Aug|2016|Journal Article|http://www.ncbi.nlm.nih.gov/entrez/query.fcgi?cmd=Retrieve&db=pubmed&dopt=Abstract&list_uids=27422337|6|Bioorg Med Chem Lett|26|16|Aug|2016|Journal Article
mapk3	mmp9	http://www.ncbi.nlm.nih.gov/entrez/query.fcgi?cmd=Retrieve&db=pubmed&dopt=Abstract&list_uids=27527140|6|Molecules|21|8||2016|Journal Article|http://www.ncbi.nlm.nih.gov/entrez/query.fcgi?cmd=Retrieve&db=pubmed&dopt=Abstract&list_uids=27470348|6|Biomed Pharmacother|82||Aug|2016|Journal Article|http://www.ncbi.nlm.nih.gov/entrez/query.fcgi?cmd=Retrieve&db=pubmed&dopt=Abstract&list_uids=27476935|6|Biol Pharm Bull|39|8||2016|Journal Article|http://www.ncbi.nlm.nih.gov/entrez/query.fcgi?cmd=Retrieve&db=pubmed&dopt=Abstract&list_uids=27422337|6|Bioorg Med Chem Lett|26|16|Aug|2016|Journal Article
mapk3	mapk8	http://www.ncbi.nlm.nih.gov/entrez/query.fcgi?cmd=Retrieve&db=pubmed&dopt=Abstract&list_uids=27366113|6|Cancer Cell Int|16|||2016|Journal Article|http://www.ncbi.nlm.nih.gov/entrez/query.fcgi?cmd=Retrieve&db=pubmed&dopt=Abstract&list_uids=27527140|6|Molecules|21|8||2016|Journal Article|http://www.ncbi.nlm.nih.gov/entrez/query.fcgi?cmd=Retrieve&db=pubmed&dopt=Abstract&list_uids=27565324|6|Neoplasma||6|Aug|2016|JOURNAL ARTICLE|http://www.ncbi.nlm.nih.gov/entrez/query.fcgi?cmd=Retrieve&db=pubmed&dopt=Abstract&list_uids=27393618|6|Breast Cancer Res Treat|158|3|Aug|2016|Journal Article|http://www.ncbi.nlm.nih.gov/entrez/query.fcgi?cmd=Retrieve&db=pubmed&dopt=Abstract&list_uids=27279438|6|Oncol Rep|36|2|Aug|2016|Journal Article
mapk3	jun	http://www.ncbi.nlm.nih.gov/entrez/query.fcgi?cmd=Retrieve&db=pubmed&dopt=Abstract&list_uids=27565324|6|Neoplasma||6|Aug|2016|JOURNAL ARTICLE
mapk3	frap1	http://www.ncbi.nlm.nih.gov/entrez/query.fcgi?cmd=Retrieve&db=pubmed&dopt=Abstract&list_uids=27589685|6|Oncotarget|||Aug|2016|JOURNAL ARTICLE|http://www.ncbi.nlm.nih.gov/entrez/query.fcgi?cmd=Retrieve&db=pubmed&dopt=Abstract&list_uids=27551764|6|Biomed Pharmacother|83||Aug|2016|JOURNAL ARTICLE

adam10	hcc	http://www.ncbi.nlm.nih.gov/entrez/query.fcgi?cmd=Retrieve&db=pubmed&dopt=Abstract&list_uids=27259866|6|J Exp Clin Cancer Res|35|1||2016|Journal Article
mmp2	ervk2	http://www.ncbi.nlm.nih.gov/entrez/query.fcgi?cmd=Retrieve&db=pubmed&dopt=Abstract&list_uids=27266355|6|Zhonghua Yi Xue Za Zhi|96|18|May|2016|English Abstract,Journal Article,Research Support, Non-U.S. Gov't
mmp2	rela	http://www.ncbi.nlm.nih.gov/entrez/query.fcgi?cmd=Retrieve&db=pubmed&dopt=Abstract&list_uids=27279714|6|Pharmacogn Mag|12|Suppl 2|May|2016|Journal Article
mmp2	tlr4	http://www.ncbi.nlm.nih.gov/entrez/query.fcgi?cmd=Retrieve&db=pubmed&dopt=Abstract&list_uids=27279714|6|Pharmacogn Mag|12|Suppl 2|May|2016|Journal Article
mmp2	fbxl20	http://www.ncbi.nlm.nih.gov/entrez/query.fcgi?cmd=Retrieve&db=pubmed&dopt=Abstract&list_uids=27322511|6|Environ Toxicol|||Jun|2016|JOURNAL ARTICLE
mmp2	vegfa	http://www.ncbi.nlm.nih.gov/entrez/query.fcgi?cmd=Retrieve&db=pubmed&dopt=Abstract&list_uids=27363017|6|Oncotarget|||May|2016|JOURNAL ARTICLE
mmp2	ptk2	http://www.ncbi.nlm.nih.gov/entrez/query.fcgi?cmd=Retrieve&db=pubmed&dopt=Abstract&list_uids=27322511|6|Environ Toxicol|||Jun|2016|JOURNAL ARTICLE|http://www.ncbi.nlm.nih.gov/entrez/query.fcgi?cmd=Retrieve&db=pubmed&dopt=Abstract&list_uids=27422337|6|Bioorg Med Chem Lett|26|16|Aug|2016|Journal Article
mmp2	src	http://www.ncbi.nlm.nih.gov/entrez/query.fcgi?cmd=Retrieve&db=pubmed&dopt=Abstract&list_uids=27422337|6|Bioorg Med Chem Lett|26|16|Aug|2016|Journal Article
mmp2	mapk14	http://www.ncbi.nlm.nih.gov/entrez/query.fcgi?cmd=Retrieve&db=pubmed&dopt=Abstract&list_uids=27461575|6|Oncol Rep|36|3|Sep|2016|Journal Article|http://www.ncbi.nlm.nih.gov/entrez/query.fcgi?cmd=Retrieve&db=pubmed&dopt=Abstract&list_uids=27348267|6|Oncogenesis|5|6||2016|Journal Article
mmp2	ephb2	http://www.ncbi.nlm.nih.gov/entrez/query.fcgi?cmd=Retrieve&db=pubmed&dopt=Abstract&list_uids=27322511|6|Environ Toxicol|||Jun|2016|JOURNAL ARTICLE|http://www.ncbi.nlm.nih.gov/entrez/query.fcgi?cmd=Retrieve&db=pubmed&dopt=Abstract&list_uids=27481258|6|Lasers Med Sci|||Aug|2016|JOURNAL ARTICLE
mmp2	mmp8	http://www.ncbi.nlm.nih.gov/entrez/query.fcgi?cmd=Retrieve&db=pubmed&dopt=Abstract&list_uids=27491937|6|Anticancer Agents Med Chem|||Aug|2016|JOURNAL ARTICLE
mmp2	mmp1	http://www.ncbi.nlm.nih.gov/entrez/query.fcgi?cmd=Retrieve&db=pubmed&dopt=Abstract&list_uids=27491937|6|Anticancer Agents Med Chem|||Aug|2016|JOURNAL ARTICLE
mmp2	plau	http://www.ncbi.nlm.nih.gov/entrez/query.fcgi?cmd=Retrieve&db=pubmed&dopt=Abstract&list_uids=27377307|6|Sci Rep|6|||2016|Journal Article|http://www.ncbi.nlm.nih.gov/entrez/query.fcgi?cmd=Retrieve&db=pubmed&dopt=Abstract&list_uids=27527140|6|Molecules|21|8||2016|Journal Article
mmp2	timp2	http://www.ncbi.nlm.nih.gov/entrez/query.fcgi?cmd=Retrieve&db=pubmed&dopt=Abstract&list_uids=27589705|6|Molecules|21|9||2016|Journal Article

mmp2	timp1	http://www.ncbi.nlm.nih.gov/entrez/query.fcgi?cmd=Retrieve&db=pubmed&dopt=Abstract&list_uids=27589705|6|Molecules|21|9||2016|Journal Article|http://www.ncbi.nlm.nih.gov/entrez/query.fcgi?cmd=Retrieve&db=pubmed&dopt=Abstract&list_uids=27260469|6|J Nutr Biochem|33||Jul|2016|Journal Article
mmp2	pik3ca	http://www.ncbi.nlm.nih.gov/entrez/query.fcgi?cmd=Retrieve&db=pubmed&dopt=Abstract&list_uids=27600360|6|Cell Biol Int|||Sep|2016|JOURNAL ARTICLE|http://www.ncbi.nlm.nih.gov/entrez/query.fcgi?cmd=Retrieve&db=pubmed&dopt=Abstract&list_uids=27470348|6|Biomed Pharmacother|82||Aug|2016|Journal Article
mmp2	pten	http://www.ncbi.nlm.nih.gov/entrez/query.fcgi?cmd=Retrieve&db=pubmed&dopt=Abstract&list_uids=27600360|6|Cell Biol Int|||Sep|2016|JOURNAL ARTICLE
mmp2	mmp9	http://www.ncbi.nlm.nih.gov/entrez/query.fcgi?cmd=Retrieve&db=pubmed&dopt=Abstract&list_uids=27589705|6|Molecules|21|9||2016|Journal Article|http://www.ncbi.nlm.nih.gov/entrez/query.fcgi?cmd=Retrieve&db=pubmed&dopt=Abstract&list_uids=27348727|6|Environ Toxicol Pharmacol|45||Jul|2016|Journal Article|http://www.ncbi.nlm.nih.gov/entrez/query.fcgi?cmd=Retrieve&db=pubmed&dopt=Abstract&list_uids=27481258|6|Lasers Med Sci|||Aug|2016|JOURNAL ARTICLE|http://www.ncbi.nlm.nih.gov/entrez/query.fcgi?cmd=Retrieve&db=pubmed&dopt=Abstract&list_uids=27511626|6|Int J Oncol|||Aug|2016|JOURNAL ARTICLE|http://www.ncbi.nlm.nih.gov/entrez/query.fcgi?cmd=Retrieve&db=pubmed&dopt=Abstract&list_uids=27377307|6|Sci Rep|6|||2016|Journal Article|http://www.ncbi.nlm.nih.gov/entrez/query.fcgi?cmd=Retrieve&db=pubmed&dopt=Abstract&list_uids=27527140|6|Molecules|21|8||2016|Journal Article|http://www.ncbi.nlm.nih.gov/entrez/query.fcgi?cmd=Retrieve&db=pubmed&dopt=Abstract&list_uids=27510965|6|Biomedicine (Taipei)|6|3|Jun|2016|Journal Article|http://www.ncbi.nlm.nih.gov/entrez/query.fcgi?cmd=Retrieve&db=pubmed&dopt=Abstract&list_uids=27470348|6|Biomed Pharmacother|82||Aug|2016|Journal Article|http://www.ncbi.nlm.nih.gov/entrez/query.fcgi?cmd=Retrieve&db=pubmed&dopt=Abstract&list_uids=27491937|6|Anticancer Agents Med Chem|||Aug|2016|JOURNAL ARTICLE|http://www.ncbi.nlm.nih.gov/entrez/query.fcgi?cmd=Retrieve&db=pubmed&dopt=Abstract&list_uids=27266355|6|Zhonghua Yi Xue Za Zhi|96|18|May|2016|English Abstract,Journal Article,Research Support, Non-U.S. Gov't|http://www.ncbi.nlm.nih.gov/entrez/query.fcgi?cmd=Retrieve&db=pubmed&dopt=Abstract&list_uids=27600360|6|Cell Biol Int|||Sep|2016|JOURNAL ARTICLE|http://www.ncbi.nlm.nih.gov/entrez/query.fcgi?cmd=Retrieve&db=pubmed&dopt=Abstract&list_uids=27279714|6|Pharmacogn Mag|12|Suppl 2|May|2016|Journal Article|http://www.ncbi.nlm.nih.gov/entrez/query.fcgi?cmd=Retrieve&db=pubmed&dopt=Abstract&list_uids=27461575|6|Oncol Rep|36|3|Sep|2016|Journal

		Article|http://www.ncbi.nlm.nih.gov/entrez/query.fcgi?cmd=Retrieve&db=pubmed&dopt=Abstract&list_uids=27327035|6|J Agric Food Chem|64|27|Jul|2016|Journal Article|http://www.ncbi.nlm.nih.gov/entrez/query.fcgi?cmd=Retrieve&db=pubmed&dopt=Abstract&list_uids=27579534|6|Oncotarget|||Aug|2016|JOURNAL ARTICLE|http://www.ncbi.nlm.nih.gov/entrez/query.fcgi?cmd=Retrieve&db=pubmed&dopt=Abstract&list_uids=27452283|6|Bioorg Med Chem|24|18|Sep|2016|Journal Article|http://www.ncbi.nlm.nih.gov/entrez/query.fcgi?cmd=Retrieve&db=pubmed&dopt=Abstract&list_uids=27572503|6|Oncol Rep|||Aug|2016|JOURNAL ARTICLE|http://www.ncbi.nlm.nih.gov/entrez/query.fcgi?cmd=Retrieve&db=pubmed&dopt=Abstract&list_uids=27422337|6|Bioorg Med Chem Lett|26|16|Aug|2016|Journal Article|http://www.ncbi.nlm.nih.gov/entrez/query.fcgi?cmd=Retrieve&db=pubmed&dopt=Abstract&list_uids=27324095|6|Placenta|43||Jul|2016|Journal Article
mmp2	mmp13	http://www.ncbi.nlm.nih.gov/entrez/query.fcgi?cmd=Retrieve&db=pubmed&dopt=Abstract&list_uids=27600927|6|J Dermatol Sci|||Jun|2016|JOURNAL ARTICLE|http://www.ncbi.nlm.nih.gov/entrez/query.fcgi?cmd=Retrieve&db=pubmed&dopt=Abstract&list_uids=27491937|6|Anticancer Agents Med Chem|||Aug|2016|JOURNAL ARTICLE
mmp2	mapk8	http://www.ncbi.nlm.nih.gov/entrez/query.fcgi?cmd=Retrieve&db=pubmed&dopt=Abstract&list_uids=27602114|6|Oncol Lett|12|3|Sep|2016|JOURNAL ARTICLE|http://www.ncbi.nlm.nih.gov/entrez/query.fcgi?cmd=Retrieve&db=pubmed&dopt=Abstract&list_uids=27527140|6|Molecules|21|8||2016|Journal Article
timp1	ephb2	http://www.ncbi.nlm.nih.gov/entrez/query.fcgi?cmd=Retrieve&db=pubmed&dopt=Abstract&list_uids=27362846|6|PLoS One|11|6||2016|Journal Article
timp1	mapk14	http://www.ncbi.nlm.nih.gov/entrez/query.fcgi?cmd=Retrieve&db=pubmed&dopt=Abstract&list_uids=27476935|6|Biol Pharm Bull|39|8||2016|Journal Article
timp1	mapk3	http://www.ncbi.nlm.nih.gov/entrez/query.fcgi?cmd=Retrieve&db=pubmed&dopt=Abstract&list_uids=27476935|6|Biol Pharm Bull|39|8||2016|Journal Article
timp1	2.6.1.77	http://www.ncbi.nlm.nih.gov/entrez/query.fcgi?cmd=Retrieve&db=pubmed&dopt=Abstract&list_uids=27476935|6|Biol Pharm Bull|39|8||2016|Journal Article
timp1	akt1	http://www.ncbi.nlm.nih.gov/entrez/query.fcgi?cmd=Retrieve&db=pubmed&dopt=Abstract&list_uids=27572091|6|Mol Med Rep|||Aug|2016|JOURNAL ARTICLE
timp1	pik3ca	http://www.ncbi.nlm.nih.gov/entrez/query.fcgi?cmd=Retrieve&db=pubmed&dopt=Abstract&list_uids=27572091|6|Mol Med Rep|||Aug|2016|JOURNAL ARTICLE
timp1	timp2	http://www.ncbi.nlm.nih.gov/entrez/query.fcgi?cmd=Retrieve&db=pubmed&dopt=Abstract&list_uids=27589705|6|Molecules|21|9||2016|Journal Article
pebp4	itk	http://www.ncbi.nlm.nih.gov/entrez/query.fcgi?cmd=Retrieve&db=pubmed&dopt=Abstract&list_uids=27261570|6|Biomed Pharmacother|81||Jul|2016|Journal Article

pebp4	pebp1	http://www.ncbi.nlm.nih.gov/entrez/query.fcgi?cmd=Retrieve&db=pubmed&dopt=Abstract&list_uids=27261570|6|Biomed Pharmacother|81||Jul|2016|Journal Article
myc	ldha	http://www.ncbi.nlm.nih.gov/entrez/query.fcgi?cmd=Retrieve&db=pubmed&dopt=Abstract&list_uids=27261617|6|Biomed Pharmacother|81||Jul|2016|Journal Article
myc	vegfa	http://www.ncbi.nlm.nih.gov/entrez/query.fcgi?cmd=Retrieve&db=pubmed&dopt=Abstract&list_uids=27415751|6|Cancer Biol Ther|||Jul|2016|JOURNAL ARTICLE
myc	bcl2	http://www.ncbi.nlm.nih.gov/entrez/query.fcgi?cmd=Retrieve&db=pubmed&dopt=Abstract&list_uids=27415751|6|Cancer Biol Ther|||Jul|2016|JOURNAL ARTICLE
myc	bcl2l1	http://www.ncbi.nlm.nih.gov/entrez/query.fcgi?cmd=Retrieve&db=pubmed&dopt=Abstract&list_uids=27415751|6|Cancer Biol Ther|||Jul|2016|JOURNAL ARTICLE
myc	hcc	http://www.ncbi.nlm.nih.gov/entrez/query.fcgi?cmd=Retrieve&db=pubmed&dopt=Abstract&list_uids=27446476|6|Oncol Lett|12|2|Aug|2016|JOURNAL ARTICLE
myc	rhoa	http://www.ncbi.nlm.nih.gov/entrez/query.fcgi?cmd=Retrieve&db=pubmed&dopt=Abstract&list_uids=27446476|6|Oncol Lett|12|2|Aug|2016|JOURNAL ARTICLE
myc	dlc1	http://www.ncbi.nlm.nih.gov/entrez/query.fcgi?cmd=Retrieve&db=pubmed&dopt=Abstract&list_uids=27446476|6|Oncol Lett|12|2|Aug|2016|JOURNAL ARTICLE
myc	gsc	http://www.ncbi.nlm.nih.gov/entrez/query.fcgi?cmd=Retrieve&db=pubmed&dopt=Abstract&list_uids=27472461|6|Oncotarget|||Jul|2016|JOURNAL ARTICLE
myc	rbpj	http://www.ncbi.nlm.nih.gov/entrez/query.fcgi?cmd=Retrieve&db=pubmed&dopt=Abstract&list_uids=27472461|6|Oncotarget|||Jul|2016|JOURNAL ARTICLE
myc	tp53	http://www.ncbi.nlm.nih.gov/entrez/query.fcgi?cmd=Retrieve&db=pubmed&dopt=Abstract&list_uids=27557535|6|Recent Results Cancer Res|207|||2016|Journal Article
myc	ahr	http://www.ncbi.nlm.nih.gov/entrez/query.fcgi?cmd=Retrieve&db=pubmed&dopt=Abstract&list_uids=27601158|6|Int J Oncol|||Aug|2016|JOURNAL ARTICLE
cd44	tspan8	http://www.ncbi.nlm.nih.gov/entrez/query.fcgi?cmd=Retrieve&db=pubmed&dopt=Abstract&list_uids=27419629|6|Oncotarget|||Jul|2016|JOURNAL ARTICLE
cd44	stat6	http://www.ncbi.nlm.nih.gov/entrez/query.fcgi?cmd=Retrieve&db=pubmed&dopt=Abstract&list_uids=27533463|6|Oncotarget|||Aug|2016|JOURNAL ARTICLE
cd44	alcam	http://www.ncbi.nlm.nih.gov/entrez/query.fcgi?cmd=Retrieve&db=pubmed&dopt=Abstract&list_uids=27533463|6|Oncotarget|||Aug|2016|JOURNAL ARTICLE
cd44	prom1	http://www.ncbi.nlm.nih.gov/entrez/query.fcgi?cmd=Retrieve&db=pubmed&dopt=Abstract&list_uids=27533463|6|Oncotarget|||Aug|2016|JOURNAL ARTICLE
cd44	pcsk1	http://www.ncbi.nlm.nih.gov/entrez/query.fcgi?cmd=Retrieve&db=pubmed&dopt=Abstract&list_uids=27569589|6|Life Sci|||Aug|2016|JOURNAL ARTICLE
cd44	axpc1	http://www.ncbi.nlm.nih.gov/entrez/query.fcgi?cmd=Retrieve&db=pubmed&dopt=Abstract&list_uids=27569589|6|Life Sci|||Aug|2016|JOURNAL ARTICLE

itga6	cd44	http://www.ncbi.nlm.nih.gov/entrez/query.fcgi?cmd=Retrieve&db=pubmed&dopt=Abstract&list_uids=27261625|6|Biomed Pharmacother|81||Jul|2016|Journal Article
twist2	cd44	http://www.ncbi.nlm.nih.gov/entrez/query.fcgi?cmd=Retrieve&db=pubmed&dopt=Abstract&list_uids=27261625|6|Biomed Pharmacother|81||Jul|2016|Journal Article
twist2	itga6	http://www.ncbi.nlm.nih.gov/entrez/query.fcgi?cmd=Retrieve&db=pubmed&dopt=Abstract&list_uids=27261625|6|Biomed Pharmacother|81||Jul|2016|Journal Article
mmp9	hcc	http://www.ncbi.nlm.nih.gov/entrez/query.fcgi?cmd=Retrieve&db=pubmed&dopt=Abstract&list_uids=27266355|6|Zhonghua Yi Xue Za Zhi|96|18|May|2016|English Abstract,Journal Article,Research Support, Non-U.S. Gov't
mmp9	ervk2	http://www.ncbi.nlm.nih.gov/entrez/query.fcgi?cmd=Retrieve&db=pubmed&dopt=Abstract&list_uids=27266355|6|Zhonghua Yi Xue Za Zhi|96|18|May|2016|English Abstract,Journal Article,Research Support, Non-U.S. Gov't
mmp9	phpt1	http://www.ncbi.nlm.nih.gov/entrez/query.fcgi?cmd=Retrieve&db=pubmed&dopt=Abstract&list_uids=27268917|6|Neoplasma|63|4||2016|Journal Article
mmp9	rela	http://www.ncbi.nlm.nih.gov/entrez/query.fcgi?cmd=Retrieve&db=pubmed&dopt=Abstract&list_uids=27279714|6|Pharmacogn Mag|12|Suppl 2|May|2016|Journal Article
mmp9	tlr4	http://www.ncbi.nlm.nih.gov/entrez/query.fcgi?cmd=Retrieve&db=pubmed&dopt=Abstract&list_uids=27279714|6|Pharmacogn Mag|12|Suppl 2|May|2016|Journal Article
mmp9	stat3	http://www.ncbi.nlm.nih.gov/entrez/query.fcgi?cmd=Retrieve&db=pubmed&dopt=Abstract&list_uids=27353107|6|Zhonghua Wei Chang Wai Ke Za Zhi|19|6|Jun|2016|English Abstract,Journal Article
mmp9	il17a	http://www.ncbi.nlm.nih.gov/entrez/query.fcgi?cmd=Retrieve&db=pubmed&dopt=Abstract&list_uids=27353107|6|Zhonghua Wei Chang Wai Ke Za Zhi|19|6|Jun|2016|English Abstract,Journal Article
mmp9	src	http://www.ncbi.nlm.nih.gov/entrez/query.fcgi?cmd=Retrieve&db=pubmed&dopt=Abstract&list_uids=27422337|6|Bioorg Med Chem Lett|26|16|Aug|2016|Journal Article
mmp9	mapk14	http://www.ncbi.nlm.nih.gov/entrez/query.fcgi?cmd=Retrieve&db=pubmed&dopt=Abstract&list_uids=27320884|6|Nan Fang Yi Ke Da Xue Xue Bao|36|6|Jun|2016|English Abstract,Journal Article,Research Support, Non-U.S. Gov't|http://www.ncbi.nlm.nih.gov/entrez/query.fcgi?cmd=Retrieve&db=pubmed&dopt=Abstract&list_uids=27461575|6|Oncol Rep|36|3|Sep|2016|Journal Article|http://www.ncbi.nlm.nih.gov/entrez/query.fcgi?cmd=Retrieve&db=pubmed&dopt=Abstract&list_uids=27476935|6|Biol Pharm Bull|39|8||2016|Journal Article
mmp9	2.6.1.77	http://www.ncbi.nlm.nih.gov/entrez/query.fcgi?cmd=Retrieve&db=pubmed&dopt=Abstract&list_uids=27476935|6|Biol Pharm Bull|39|8||2016|Journal Article
mmp9	mmp13	http://www.ncbi.nlm.nih.gov/entrez/query.fcgi?cmd=Retrieve&db=pubmed&dopt=Abstract&list_uids=27491937|6|Anticancer Agents Med Chem|||Aug|2016|JOURNAL ARTICLE
mmp9	mmp8	http://www.ncbi.nlm.nih.gov/entrez/query.fcgi?cmd=Retrieve&db=pubmed&dopt=Abstract&list_uids=27491937|6|Anticancer Agents Med Chem|||Aug|2016|JOURNAL ARTICLE
mmp9	mmp1	http://www.ncbi.nlm.nih.gov/entrez/query.fcgi?cmd=Retrieve&db=pubmed&dopt=Abstract&list_uids=27491937|6|Anticancer Agents Med Chem|||Aug|2016|JOURNAL ARTICLE

mmp9	plau	http://www.ncbi.nlm.nih.gov/entrez/query.fcgi?cmd=Retrieve&db=pubmed&dopt=Abstract&list_uids=27377307|6|Sci Rep|6|||2016|Journal Article|http://www.ncbi.nlm.nih.gov/entrez/query.fcgi?cmd=Retrieve&db=pubmed&dopt=Abstract&list_uids=27527140|6|Molecules|21|8||2016|Journal Article
mmp9	ptk2	http://www.ncbi.nlm.nih.gov/entrez/query.fcgi?cmd=Retrieve&db=pubmed&dopt=Abstract&list_uids=27573547|6|Int J Oncol|49|3|Sep|2016|Journal Article|http://www.ncbi.nlm.nih.gov/entrez/query.fcgi?cmd=Retrieve&db=pubmed&dopt=Abstract&list_uids=27422337|6|Bioorg Med Chem Lett|26|16|Aug|2016|Journal Article
mmp9	timp2	http://www.ncbi.nlm.nih.gov/entrez/query.fcgi?cmd=Retrieve&db=pubmed&dopt=Abstract&list_uids=27589705|6|Molecules|21|9||2016|Journal Article
mmp9	timp1	http://www.ncbi.nlm.nih.gov/entrez/query.fcgi?cmd=Retrieve&db=pubmed&dopt=Abstract&list_uids=27589705|6|Molecules|21|9||2016|Journal Article|http://www.ncbi.nlm.nih.gov/entrez/query.fcgi?cmd=Retrieve&db=pubmed&dopt=Abstract&list_uids=27596524|6|Toxicol In Vitro|||Sep|2016|JOURNAL ARTICLE|http://www.ncbi.nlm.nih.gov/entrez/query.fcgi?cmd=Retrieve&db=pubmed&dopt=Abstract&list_uids=27476935|6|Biol Pharm Bull|39|8||2016|Journal Article
mmp9	pik3ca	http://www.ncbi.nlm.nih.gov/entrez/query.fcgi?cmd=Retrieve&db=pubmed&dopt=Abstract&list_uids=27600360|6|Cell Biol Int|||Sep|2016|JOURNAL ARTICLE|http://www.ncbi.nlm.nih.gov/entrez/query.fcgi?cmd=Retrieve&db=pubmed&dopt=Abstract&list_uids=27470348|6|Biomed Pharmacother|82||Aug|2016|Journal Article
mmp9	pten	http://www.ncbi.nlm.nih.gov/entrez/query.fcgi?cmd=Retrieve&db=pubmed&dopt=Abstract&list_uids=27600360|6|Cell Biol Int|||Sep|2016|JOURNAL ARTICLE
mmp9	ahr	http://www.ncbi.nlm.nih.gov/entrez/query.fcgi?cmd=Retrieve&db=pubmed&dopt=Abstract&list_uids=27601158|6|Int J Oncol|||Aug|2016|JOURNAL ARTICLE
mmp9	myc	http://www.ncbi.nlm.nih.gov/entrez/query.fcgi?cmd=Retrieve&db=pubmed&dopt=Abstract&list_uids=27601158|6|Int J Oncol|||Aug|2016|JOURNAL ARTICLE
ervk2	yap1	http://www.ncbi.nlm.nih.gov/entrez/query.fcgi?cmd=Retrieve&db=pubmed&dopt=Abstract&list_uids=27399496|6|Neurosurgery|63 Suppl 1||Aug|2016|Journal Article
ervk2	ervwe1	http://www.ncbi.nlm.nih.gov/entrez/query.fcgi?cmd=Retrieve&db=pubmed&dopt=Abstract&list_uids=27557521|6|Oncotarget|||Aug|2016|JOURNAL ARTICLE
ervk2	rfxank	http://www.ncbi.nlm.nih.gov/entrez/query.fcgi?cmd=Retrieve&db=pubmed&dopt=Abstract&list_uids=27578985|6|Onco Targets Ther|9|||2016|Journal Article
ervk2	akt1	http://www.ncbi.nlm.nih.gov/entrez/query.fcgi?cmd=Retrieve&db=pubmed&dopt=Abstract&list_uids=27578985|6|Onco Targets Ther|9|||2016|Journal Article
ervk2	pik3ca	http://www.ncbi.nlm.nih.gov/entrez/query.fcgi?cmd=Retrieve&db=pubmed&dopt=Abstract&list_uids=27578985|6|Onco Targets Ther|9|||2016|Journal Article

ervk2	pik3r2	http://www.ncbi.nlm.nih.gov/entrez/query.fcgi?cmd=Retrieve&db=pubmed&dopt=Abstract&list_uids=27578985|6|Onco Targets Ther|9|||2016|Journal Article
ervk2	mirn126	http://www.ncbi.nlm.nih.gov/entrez/query.fcgi?cmd=Retrieve&db=pubmed&dopt=Abstract&list_uids=27578985|6|Onco Targets Ther|9|||2016|Journal Article
mapk8	foxo1	http://www.ncbi.nlm.nih.gov/entrez/query.fcgi?cmd=Retrieve&db=pubmed&dopt=Abstract&list_uids=27268017|6|BMC Gastroenterol|16|1||2016|Journal Article
mapk8	rela	http://www.ncbi.nlm.nih.gov/entrez/query.fcgi?cmd=Retrieve&db=pubmed&dopt=Abstract&list_uids=27393618|6|Breast Cancer Res Treat|158|3|Aug|2016|Journal Article
mapk8	nf-kappab	http://www.ncbi.nlm.nih.gov/entrez/query.fcgi?cmd=Retrieve&db=pubmed&dopt=Abstract&list_uids=27393618|6|Breast Cancer Res Treat|158|3|Aug|2016|Journal Article
mapk8	plau	http://www.ncbi.nlm.nih.gov/entrez/query.fcgi?cmd=Retrieve&db=pubmed&dopt=Abstract&list_uids=27527140|6|Molecules|21|8||2016|Journal Article
mapk8	mmp9	http://www.ncbi.nlm.nih.gov/entrez/query.fcgi?cmd=Retrieve&db=pubmed&dopt=Abstract&list_uids=27527140|6|Molecules|21|8||2016|Journal Article|http://www.ncbi.nlm.nih.gov/entrez/query.fcgi?cmd=Retrieve&db=pubmed&dopt=Abstract&list_uids=27367023|6|Oncotarget|||Jun|2016|JOURNAL ARTICLE
mapk8	akt1	http://www.ncbi.nlm.nih.gov/entrez/query.fcgi?cmd=Retrieve&db=pubmed&dopt=Abstract&list_uids=27527140|6|Molecules|21|8||2016|Journal Article|http://www.ncbi.nlm.nih.gov/entrez/query.fcgi?cmd=Retrieve&db=pubmed&dopt=Abstract&list_uids=27599909|6|Mol Med Rep|||Sep|2016|JOURNAL ARTICLE
mapk8	stat3	http://www.ncbi.nlm.nih.gov/entrez/query.fcgi?cmd=Retrieve&db=pubmed&dopt=Abstract&list_uids=27599909|6|Mol Med Rep|||Sep|2016|JOURNAL ARTICLE
jun	foxo1	http://www.ncbi.nlm.nih.gov/entrez/query.fcgi?cmd=Retrieve&db=pubmed&dopt=Abstract&list_uids=27268017|6|BMC Gastroenterol|16|1||2016|Journal Article
jun	axl	http://www.ncbi.nlm.nih.gov/entrez/query.fcgi?cmd=Retrieve&db=pubmed&dopt=Abstract&list_uids=27385093|6|Oncotarget|||Jul|2016|JOURNAL ARTICLE
jun	atf2	http://www.ncbi.nlm.nih.gov/entrez/query.fcgi?cmd=Retrieve&db=pubmed&dopt=Abstract&list_uids=27385093|6|Oncotarget|||Jul|2016|JOURNAL ARTICLE
jun	pcsk1	http://www.ncbi.nlm.nih.gov/entrez/query.fcgi?cmd=Retrieve&db=pubmed&dopt=Abstract&list_uids=27385093|6|Oncotarget|||Jul|2016|JOURNAL ARTICLE
jun	plau	http://www.ncbi.nlm.nih.gov/entrez/query.fcgi?cmd=Retrieve&db=pubmed&dopt=Abstract&list_uids=27385093|6|Oncotarget|||Jul|2016|JOURNAL ARTICLE
jun	cops5	http://www.ncbi.nlm.nih.gov/entrez/query.fcgi?cmd=Retrieve&db=pubmed&dopt=Abstract&list_uids=27412572|6|Med Oncol|33|8|Aug|2016|Journal Article,Review
jun	nf1	http://www.ncbi.nlm.nih.gov/entrez/query.fcgi?cmd=Retrieve&db=pubmed&dopt=Abstract&list_uids=27494896|6|Oncotarget|||Aug|2016|JOURNAL ARTICLE

jun	rhoc	http://www.ncbi.nlm.nih.gov/entrez/query.fcgi?cmd=Retrieve&db=pubmed&dopt=Abstract&list_uids=27494896|6|Oncotarget|||Aug|2016|JOURNAL ARTICLE
jun	tnfsf13	http://www.ncbi.nlm.nih.gov/entrez/query.fcgi?cmd=Retrieve&db=pubmed&dopt=Abstract&list_uids=27529686|6|Int J Cancer|||Aug|2016|JOURNAL ARTICLE
jun	hgf	http://www.ncbi.nlm.nih.gov/entrez/query.fcgi?cmd=Retrieve&db=pubmed&dopt=Abstract&list_uids=27529686|6|Int J Cancer|||Aug|2016|JOURNAL ARTICLE
jun	slc9a1	http://www.ncbi.nlm.nih.gov/entrez/query.fcgi?cmd=Retrieve&db=pubmed&dopt=Abstract&list_uids=27529686|6|Int J Cancer|||Aug|2016|JOURNAL ARTICLE
jun	mapk8	http://www.ncbi.nlm.nih.gov/entrez/query.fcgi?cmd=Retrieve&db=pubmed&dopt=Abstract&list_uids=27392951|6|Target Oncol|||Jul|2016|JOURNAL ARTICLE|http://www.ncbi.nlm.nih.gov/entrez/query.fcgi?cmd=Retrieve&db=pubmed&dopt=Abstract&list_uids=27268017|6|BMC Gastroenterol|16|1||2016|Journal Article|http://www.ncbi.nlm.nih.gov/entrez/query.fcgi?cmd=Retrieve&db=pubmed&dopt=Abstract&list_uids=27565324|6|Neoplasma||6|Aug|2016|JOURNAL ARTICLE
myst2	suv39h1	http://www.ncbi.nlm.nih.gov/entrez/query.fcgi?cmd=Retrieve&db=pubmed&dopt=Abstract&list_uids=27270040|6|Dev Cell|37|5|Jun|2016|Journal Article
ptpn11	ifna1	http://www.ncbi.nlm.nih.gov/entrez/query.fcgi?cmd=Retrieve&db=pubmed&dopt=Abstract&list_uids=27270128|6|Virus Res|222||Aug|2016|Journal Article
ptpn11	ceacam1	http://www.ncbi.nlm.nih.gov/entrez/query.fcgi?cmd=Retrieve&db=pubmed&dopt=Abstract&list_uids=27572314|6|Oncotarget|||Aug|2016|JOURNAL ARTICLE
tspan8	hcc	http://www.ncbi.nlm.nih.gov/entrez/query.fcgi?cmd=Retrieve&db=pubmed&dopt=Abstract&list_uids=27339400|6|FEBS Lett|590|16|Aug|2016|Letter|http://www.ncbi.nlm.nih.gov/entrez/query.fcgi?cmd=Retrieve&db=pubmed&dopt=Abstract&list_uids=27270327|6|Oncotarget|||Jun|2016|JOURNAL ARTICLE
tp53	chek1	http://www.ncbi.nlm.nih.gov/entrez/query.fcgi?cmd=Retrieve&db=pubmed&dopt=Abstract&list_uids=27279256|6|Int J Oncol|49|2|Aug|2016|Journal Article
tp53	sparc	http://www.ncbi.nlm.nih.gov/entrez/query.fcgi?cmd=Retrieve&db=pubmed&dopt=Abstract&list_uids=27318148|6|Gastroenterology|151|3|Sep|2016|Journal Article
tp53	prkch	http://www.ncbi.nlm.nih.gov/entrez/query.fcgi?cmd=Retrieve&db=pubmed&dopt=Abstract&list_uids=27351203|6|PLoS One|11|6||2016|Journal Article
tp53	s100a4	http://www.ncbi.nlm.nih.gov/entrez/query.fcgi?cmd=Retrieve&db=pubmed&dopt=Abstract&list_uids=27382312|6|Onco Targets Ther|9|||2016|Journal Article
tp53	bax	http://www.ncbi.nlm.nih.gov/entrez/query.fcgi?cmd=Retrieve&db=pubmed&dopt=Abstract&list_uids=27421660|6|Acta Biochim Biophys Sin (Shanghai)|48|8|Aug|2016|Journal Article
tp53	bcl2l1	http://www.ncbi.nlm.nih.gov/entrez/query.fcgi?cmd=Retrieve&db=pubmed&dopt=Abstract&list_uids=27421660|6|Acta Biochim Biophys Sin (Shanghai)|48|8|Aug|2016|Journal Article
tp53	bcl2	http://www.ncbi.nlm.nih.gov/entrez/query.fcgi?cmd=Retrieve&db=pubmed&dopt=Abstract&list_uids=27421660|6|Acta

		Biochim Biophys Sin (Shanghai)|48|8|Aug|2016|Journal Article
tp53	cdkn1a	http://www.ncbi.nlm.nih.gov/entrez/query.fcgi?cmd=Retrieve&db=pubmed&dopt=Abstract&list_uids=27421660|6|Acta Biochim Biophys Sin (Shanghai)|48|8|Aug|2016|Journal Article|http://www.ncbi.nlm.nih.gov/entrez/query.fcgi?cmd=Retrieve&db=pubmed&dopt=Abstract&list_uids=27351203|6|PLoS One|11|6||2016|Journal Article
tp53	ervk2	http://www.ncbi.nlm.nih.gov/entrez/query.fcgi?cmd=Retrieve&db=pubmed&dopt=Abstract&list_uids=27557521|6|Oncotarget|||Aug|2016|JOURNAL ARTICLE
jmjd1a	tp53	http://www.ncbi.nlm.nih.gov/entrez/query.fcgi?cmd=Retrieve&db=pubmed&dopt=Abstract&list_uids=27270439|6|Oncogene|||Jun|2016|JOURNAL ARTICLE
prmt5	wdr5	http://www.ncbi.nlm.nih.gov/entrez/query.fcgi?cmd=Retrieve&db=pubmed&dopt=Abstract&list_uids=27270440|6|Oncogene|||Jun|2016|JOURNAL ARTICLE
prmt5	wdr77	http://www.ncbi.nlm.nih.gov/entrez/query.fcgi?cmd=Retrieve&db=pubmed&dopt=Abstract&list_uids=27270440|6|Oncogene|||Jun|2016|JOURNAL ARTICLE
map3k14	mmp14	http://www.ncbi.nlm.nih.gov/entrez/query.fcgi?cmd=Retrieve&db=pubmed&dopt=Abstract&list_uids=27270613|6|Oncogenesis|5|6||2016|Journal Article
mirn373	stat3	http://www.ncbi.nlm.nih.gov/entrez/query.fcgi?cmd=Retrieve&db=pubmed&dopt=Abstract&list_uids=27271572|6|PLoS One|11|6||2016|Journal Article
enox2	pou3f2	http://www.ncbi.nlm.nih.gov/entrez/query.fcgi?cmd=Retrieve&db=pubmed&dopt=Abstract&list_uids=27271588|6|Molecules|21|6||2016|Journal Article
cldn7	zeb1	http://www.ncbi.nlm.nih.gov/entrez/query.fcgi?cmd=Retrieve&db=pubmed&dopt=Abstract&list_uids=27286256|6|Oncotarget|||Jun|2016|JOURNAL ARTICLE
cldn7	tjp1	http://www.ncbi.nlm.nih.gov/entrez/query.fcgi?cmd=Retrieve&db=pubmed&dopt=Abstract&list_uids=27286256|6|Oncotarget|||Jun|2016|JOURNAL ARTICLE
msi2	cldn7	http://www.ncbi.nlm.nih.gov/entrez/query.fcgi?cmd=Retrieve&db=pubmed&dopt=Abstract&list_uids=27274057|6|Proc Natl Acad Sci U S A|113|25|Jun|2016|Journal Article
msi2	klf4	http://www.ncbi.nlm.nih.gov/entrez/query.fcgi?cmd=Retrieve&db=pubmed&dopt=Abstract&list_uids=27449499|6|Clin Cancer Res|||Jul|2016|JOURNAL ARTICLE
mapk14	nf-kappab	http://www.ncbi.nlm.nih.gov/entrez/query.fcgi?cmd=Retrieve&db=pubmed&dopt=Abstract&list_uids=27393618|6|Breast Cancer Res Treat|158|3|Aug|2016|Journal Article
mapk14	pik3ca	http://www.ncbi.nlm.nih.gov/entrez/query.fcgi?cmd=Retrieve&db=pubmed&dopt=Abstract&list_uids=27394418|6|Neurochem Res|||Jul|2016|JOURNAL ARTICLE
mapk14	snord87	http://www.ncbi.nlm.nih.gov/entrez/query.fcgi?cmd=Retrieve&db=pubmed&dopt=Abstract&list_uids=27394418|6|Neurochem Res|||Jul|2016|JOURNAL ARTICLE
mapk14	itk	http://www.ncbi.nlm.nih.gov/entrez/query.fcgi?cmd=Retrieve&db=pubmed&dopt=Abstract&list_uids=27507215|6|Biochem Biophys Res Commun|478|2|Sep|2016|Journal Article

mapk14	akt1	http://www.ncbi.nlm.nih.gov/entrez/query.fcgi?cmd=Retrieve&db=pubmed&dopt=Abstract&list_uids=27394418|6|Neurochem Res|||Jul|2016|JOURNAL ARTICLE|http://www.ncbi.nlm.nih.gov/entrez/query.fcgi?cmd=Retrieve&db=pubmed&dopt=Abstract&list_uids=27507215|6|Biochem Biophys Res Commun|478|2|Sep|2016|Journal Article
mapk14	macc1	http://www.ncbi.nlm.nih.gov/entrez/query.fcgi?cmd=Retrieve&db=pubmed&dopt=Abstract&list_uids=27529686|6|Int J Cancer|||Aug|2016|JOURNAL ARTICLE
mapk14	mapk8	http://www.ncbi.nlm.nih.gov/entrez/query.fcgi?cmd=Retrieve&db=pubmed&dopt=Abstract&list_uids=27519288|6|Toxicology|366-367||Aug|2016|JOURNAL ARTICLE|http://www.ncbi.nlm.nih.gov/entrez/query.fcgi?cmd=Retrieve&db=pubmed&dopt=Abstract&list_uids=27366113|6|Cancer Cell Int|16|||2016|Journal Article|http://www.ncbi.nlm.nih.gov/entrez/query.fcgi?cmd=Retrieve&db=pubmed&dopt=Abstract&list_uids=27565324|6|Neoplasma||6|Aug|2016|JOURNAL ARTICLE|http://www.ncbi.nlm.nih.gov/entrez/query.fcgi?cmd=Retrieve&db=pubmed&dopt=Abstract&list_uids=27393618|6|Breast Cancer Res Treat|158|3|Aug|2016|Journal Article
mapk14	jun	http://www.ncbi.nlm.nih.gov/entrez/query.fcgi?cmd=Retrieve&db=pubmed&dopt=Abstract&list_uids=27565324|6|Neoplasma||6|Aug|2016|JOURNAL ARTICLE
mapk14	mapk3	http://www.ncbi.nlm.nih.gov/entrez/query.fcgi?cmd=Retrieve&db=pubmed&dopt=Abstract&list_uids=27366113|6|Cancer Cell Int|16|||2016|Journal Article|http://www.ncbi.nlm.nih.gov/entrez/query.fcgi?cmd=Retrieve&db=pubmed&dopt=Abstract&list_uids=27565324|6|Neoplasma||6|Aug|2016|JOURNAL ARTICLE|http://www.ncbi.nlm.nih.gov/entrez/query.fcgi?cmd=Retrieve&db=pubmed&dopt=Abstract&list_uids=27393618|6|Breast Cancer Res Treat|158|3|Aug|2016|Journal Article|http://www.ncbi.nlm.nih.gov/entrez/query.fcgi?cmd=Retrieve&db=pubmed&dopt=Abstract&list_uids=27588409|6|Oncotarget|||Aug|2016|JOURNAL ARTICLE|http://www.ncbi.nlm.nih.gov/entrez/query.fcgi?cmd=Retrieve&db=pubmed&dopt=Abstract&list_uids=27476935|6|Biol Pharm Bull|39|8||2016|Journal Article
mirn375	fzd8	http://www.ncbi.nlm.nih.gov/entrez/query.fcgi?cmd=Retrieve&db=pubmed&dopt=Abstract&list_uids=27276676|6|Oncotarget|||Jun|2016|JOURNAL ARTICLE
eif2c2	eif4e	http://www.ncbi.nlm.nih.gov/entrez/query.fcgi?cmd=Retrieve&db=pubmed&dopt=Abstract&list_uids=27276678|6|Oncotarget|||Jun|2016|JOURNAL ARTICLE
uts2r	eif4e	http://www.ncbi.nlm.nih.gov/entrez/query.fcgi?cmd=Retrieve&db=pubmed&dopt=Abstract&list_uids=27276678|6|Oncotarget|||Jun|2016|JOURNAL ARTICLE
uts2r	eif2c2	http://www.ncbi.nlm.nih.gov/entrez/query.fcgi?cmd=Retrieve&db=pubmed&dopt=Abstract&list_uids=27276678|6|Oncotarget|||Jun|2016|JOURNAL ARTICLE
uts2r	pdcd4	http://www.ncbi.nlm.nih.gov/entrez/query.fcgi?cmd=Retrieve&db=pubmed&dopt=Abstract&list_uids=27279541|6|Sci Rep|6|||2016|Journal Article

uts2r	fut4	http://www.ncbi.nlm.nih.gov/entrez/query.fcgi?cmd=Retrieve&db=pubmed&dopt=Abstract&list_uids=27375041|6|Oncol Rep|36|2|Aug|2016|Journal Article
uts2r	nostrin	http://www.ncbi.nlm.nih.gov/entrez/query.fcgi?cmd=Retrieve&db=pubmed&dopt=Abstract&list_uids=27401251|6|Clin Cancer Res|||Jul|2016|JOURNAL ARTICLE
uts2r	sfrp1	http://www.ncbi.nlm.nih.gov/entrez/query.fcgi?cmd=Retrieve&db=pubmed&dopt=Abstract&list_uids=27459115|6|Biomed Pharmacother|83||Jul|2016|JOURNAL ARTICLE
uts2r	nrn1	http://www.ncbi.nlm.nih.gov/entrez/query.fcgi?cmd=Retrieve&db=pubmed&dopt=Abstract&list_uids=27471565|6|J Cancer|7|11||2016|Journal Article
pdcd4	rela	http://www.ncbi.nlm.nih.gov/entrez/query.fcgi?cmd=Retrieve&db=pubmed&dopt=Abstract&list_uids=27533779|6|Mol Carcinog|||Aug|2016|JOURNAL ARTICLE
pdcd4	eif2ak3	http://www.ncbi.nlm.nih.gov/entrez/query.fcgi?cmd=Retrieve&db=pubmed&dopt=Abstract&list_uids=27533779|6|Mol Carcinog|||Aug|2016|JOURNAL ARTICLE
pdcd4	ephb2	http://www.ncbi.nlm.nih.gov/entrez/query.fcgi?cmd=Retrieve&db=pubmed&dopt=Abstract&list_uids=27533779|6|Mol Carcinog|||Aug|2016|JOURNAL ARTICLE
pdcd4	pten	http://www.ncbi.nlm.nih.gov/entrez/query.fcgi?cmd=Retrieve&db=pubmed&dopt=Abstract&list_uids=27533779|6|Mol Carcinog|||Aug|2016|JOURNAL ARTICLE
pdcd4	bcl2	http://www.ncbi.nlm.nih.gov/entrez/query.fcgi?cmd=Retrieve&db=pubmed&dopt=Abstract&list_uids=27600360|6|Cell Biol Int|||Sep|2016|JOURNAL ARTICLE
pdcd4	bax	http://www.ncbi.nlm.nih.gov/entrez/query.fcgi?cmd=Retrieve&db=pubmed&dopt=Abstract&list_uids=27600360|6|Cell Biol Int|||Sep|2016|JOURNAL ARTICLE
hotair	pdcd4	http://www.ncbi.nlm.nih.gov/entrez/query.fcgi?cmd=Retrieve&db=pubmed&dopt=Abstract&list_uids=27277755|6|Int J Oncol|49|2|Aug|2016|Journal Article
hotair	aof2	http://www.ncbi.nlm.nih.gov/entrez/query.fcgi?cmd=Retrieve&db=pubmed&dopt=Abstract&list_uids=27277755|6|Int J Oncol|49|2|Aug|2016|Journal Article
hotair	ezh2	http://www.ncbi.nlm.nih.gov/entrez/query.fcgi?cmd=Retrieve&db=pubmed&dopt=Abstract&list_uids=27277755|6|Int J Oncol|49|2|Aug|2016|Journal Article
hotair	hla-g	http://www.ncbi.nlm.nih.gov/entrez/query.fcgi?cmd=Retrieve&db=pubmed&dopt=Abstract&list_uids=27574106|6|Int J Oncol|49|3|Sep|2016|Journal Article
aof2	pdcd4	http://www.ncbi.nlm.nih.gov/entrez/query.fcgi?cmd=Retrieve&db=pubmed&dopt=Abstract&list_uids=27277755|6|Int J Oncol|49|2|Aug|2016|Journal Article
aof2	itk	http://www.ncbi.nlm.nih.gov/entrez/query.fcgi?cmd=Retrieve&db=pubmed&dopt=Abstract&list_uids=27292636|6|Cell Rep|15|12|Jun|2016|Journal Article
ezh2	pdcd4	http://www.ncbi.nlm.nih.gov/entrez/query.fcgi?cmd=Retrieve&db=pubmed&dopt=Abstract&list_uids=27277755|6|Int J Oncol|49|2|Aug|2016|Journal Article
ezh2	aof2	http://www.ncbi.nlm.nih.gov/entrez/query.fcgi?cmd=Retrieve&db=pubmed&dopt=Abstract&list_uids=27277755|6|Int J Oncol|49|2|Aug|2016|Journal Article

ezh2	foxm1	http://www.ncbi.nlm.nih.gov/entrez/query.fcgi?cmd=Retrieve&db=pubmed&dopt=Abstract&list_uids=27303043|6|Proc Natl Acad Sci U S A|113|26|Jun|2016|Journal Article
ezh2	tam	http://www.ncbi.nlm.nih.gov/entrez/query.fcgi?cmd=Retrieve&db=pubmed&dopt=Abstract&list_uids=27436059|6|Cell Biol Int|||Jul|2016|JOURNAL ARTICLE
ezh2	akr1b1	http://www.ncbi.nlm.nih.gov/entrez/query.fcgi?cmd=Retrieve&db=pubmed&dopt=Abstract&list_uids=27436059|6|Cell Biol Int|||Jul|2016|JOURNAL ARTICLE
art1	itk	http://www.ncbi.nlm.nih.gov/entrez/query.fcgi?cmd=Retrieve&db=pubmed&dopt=Abstract&list_uids=27277835|6|Int J Oncol|49|2|Aug|2016|Journal Article
rhoa	akt1	http://www.ncbi.nlm.nih.gov/entrez/query.fcgi?cmd=Retrieve&db=pubmed&dopt=Abstract&list_uids=27277835|6|Int J Oncol|49|2|Aug|2016|Journal Article
rhoa	cdc42	http://www.ncbi.nlm.nih.gov/entrez/query.fcgi?cmd=Retrieve&db=pubmed&dopt=Abstract&list_uids=27340868|6|Oncotarget|||Jun|2016|JOURNAL ARTICLE
rhoa	rac1	http://www.ncbi.nlm.nih.gov/entrez/query.fcgi?cmd=Retrieve&db=pubmed&dopt=Abstract&list_uids=27330411|6|Cancer Cell Int|16|||2016|Journal Article|http://www.ncbi.nlm.nih.gov/entrez/query.fcgi?cmd=Retrieve&db=pubmed&dopt=Abstract&list_uids=27340868|6|Oncotarget|||Jun|2016|JOURNAL ARTICLE
rhoa	ephb2	http://www.ncbi.nlm.nih.gov/entrez/query.fcgi?cmd=Retrieve&db=pubmed&dopt=Abstract&list_uids=27391444|6|Oncotarget|||Jul|2016|JOURNAL ARTICLE
rhoa	mlc1	http://www.ncbi.nlm.nih.gov/entrez/query.fcgi?cmd=Retrieve&db=pubmed&dopt=Abstract&list_uids=27432222|6|Mol Med Rep|14|3|Sep|2016|Journal Article
rhoa	myh14	http://www.ncbi.nlm.nih.gov/entrez/query.fcgi?cmd=Retrieve&db=pubmed&dopt=Abstract&list_uids=27432222|6|Mol Med Rep|14|3|Sep|2016|Journal Article
rhoa	cxcr4	http://www.ncbi.nlm.nih.gov/entrez/query.fcgi?cmd=Retrieve&db=pubmed&dopt=Abstract&list_uids=27517626|6|Oncotarget|||Aug|2016|JOURNAL ARTICLE
snx9	src	http://www.ncbi.nlm.nih.gov/entrez/query.fcgi?cmd=Retrieve&db=pubmed&dopt=Abstract&list_uids=27278018|6|J Cell Sci|129|14|Jul|2016|Journal Article
ros1	decr1	http://www.ncbi.nlm.nih.gov/entrez/query.fcgi?cmd=Retrieve&db=pubmed&dopt=Abstract&list_uids=27315395|6|Mol Oral Microbiol|||Jun|2016|JOURNAL ARTICLE
ros1	mapk14	http://www.ncbi.nlm.nih.gov/entrez/query.fcgi?cmd=Retrieve&db=pubmed&dopt=Abstract&list_uids=27366113|6|Cancer Cell Int|16|||2016|Journal Article|http://www.ncbi.nlm.nih.gov/entrez/query.fcgi?cmd=Retrieve&db=pubmed&dopt=Abstract&list_uids=27278736|6|Int J Oncol|49|2|Aug|2016|Journal Article
ros1	mapk8	http://www.ncbi.nlm.nih.gov/entrez/query.fcgi?cmd=Retrieve&db=pubmed&dopt=Abstract&list_uids=27366113|6|Cancer Cell Int|16|||2016|Journal Article
ros1	mapk3	http://www.ncbi.nlm.nih.gov/entrez/query.fcgi?cmd=Retrieve&db=pubmed&dopt=Abstract&list_uids=27366113|6|Cancer Cell Int|16|||2016|Journal Article

ros1	sp1	http://www.ncbi.nlm.nih.gov/entrez/query.fcgi?cmd=Retrieve&db=pubmed&dopt=Abstract&list_uids=27416558|6|Biomed Pharmacother|83||Jul|2016|JOURNAL ARTICLE
ros1	prkaa1	http://www.ncbi.nlm.nih.gov/entrez/query.fcgi?cmd=Retrieve&db=pubmed&dopt=Abstract&list_uids=27416558|6|Biomed Pharmacother|83||Jul|2016|JOURNAL ARTICLE
ros1	aprt	http://www.ncbi.nlm.nih.gov/entrez/query.fcgi?cmd=Retrieve&db=pubmed&dopt=Abstract&list_uids=27416558|6|Biomed Pharmacother|83||Jul|2016|JOURNAL ARTICLE
ros1	vegfa	http://www.ncbi.nlm.nih.gov/entrez/query.fcgi?cmd=Retrieve&db=pubmed&dopt=Abstract&list_uids=27498749|6|Int J Oncol|||Aug|2016|JOURNAL ARTICLE
ros1	ephb2	http://www.ncbi.nlm.nih.gov/entrez/query.fcgi?cmd=Retrieve&db=pubmed&dopt=Abstract&list_uids=27278736|6|Int J Oncol|49|2|Aug|2016|Journal Article|http://www.ncbi.nlm.nih.gov/entrez/query.fcgi?cmd=Retrieve&db=pubmed&dopt=Abstract&list_uids=27481258|6|Lasers Med Sci|||Aug|2016|JOURNAL ARTICLE|http://www.ncbi.nlm.nih.gov/entrez/query.fcgi?cmd=Retrieve&db=pubmed&dopt=Abstract&list_uids=27572503|6|Oncol Rep|||Aug|2016|JOURNAL ARTICLE
ros1	mmp9	http://www.ncbi.nlm.nih.gov/entrez/query.fcgi?cmd=Retrieve&db=pubmed&dopt=Abstract&list_uids=27481258|6|Lasers Med Sci|||Aug|2016|JOURNAL ARTICLE|http://www.ncbi.nlm.nih.gov/entrez/query.fcgi?cmd=Retrieve&db=pubmed&dopt=Abstract&list_uids=27572503|6|Oncol Rep|||Aug|2016|JOURNAL ARTICLE
ros1	mmp2	http://www.ncbi.nlm.nih.gov/entrez/query.fcgi?cmd=Retrieve&db=pubmed&dopt=Abstract&list_uids=27481258|6|Lasers Med Sci|||Aug|2016|JOURNAL ARTICLE|http://www.ncbi.nlm.nih.gov/entrez/query.fcgi?cmd=Retrieve&db=pubmed&dopt=Abstract&list_uids=27572503|6|Oncol Rep|||Aug|2016|JOURNAL ARTICLE
slc2a1	vegfa	http://www.ncbi.nlm.nih.gov/entrez/query.fcgi?cmd=Retrieve&db=pubmed&dopt=Abstract&list_uids=27409833|6|Oncotarget|||Jul|2016|JOURNAL ARTICLE
plau	slc2a1	http://www.ncbi.nlm.nih.gov/entrez/query.fcgi?cmd=Retrieve&db=pubmed&dopt=Abstract&list_uids=27278736|6|Int J Oncol|49|2|Aug|2016|Journal Article
plau	fpr1	http://www.ncbi.nlm.nih.gov/entrez/query.fcgi?cmd=Retrieve&db=pubmed&dopt=Abstract&list_uids=27323409|6|Oncotarget|||Jun|2016|JOURNAL ARTICLE
plau	pcsk1	http://www.ncbi.nlm.nih.gov/entrez/query.fcgi?cmd=Retrieve&db=pubmed&dopt=Abstract&list_uids=27385093|6|Oncotarget|||Jul|2016|JOURNAL ARTICLE
plau	igf1r	http://www.ncbi.nlm.nih.gov/entrez/query.fcgi?cmd=Retrieve&db=pubmed&dopt=Abstract&list_uids=27502396|6|BMC Cancer|16|||2016|Journal Article
plau	serpine1	http://www.ncbi.nlm.nih.gov/entrez/query.fcgi?cmd=Retrieve&db=pubmed&dopt=Abstract&list_uids=27490118|6|PLoS One|11|8||2016|Journal Article|http://www.ncbi.nlm.nih.gov/entrez/query.fcgi?cmd=Retrieve&db=pubmed&dopt=Abstract&list_uids=27558262|6|Contrast Media Mol Imaging|||Aug|2016|JOURNAL

		ARTICLE|http://www.ncbi.nlm.nih.gov/entrez/query.fcgi?cmd=Retrieve&db=pubmed&dopt=Abstract&list_uids=27502396|6|BMC Cancer|16|||2016|Journal Article
plau	plaur	http://www.ncbi.nlm.nih.gov/entrez/query.fcgi?cmd=Retrieve&db=pubmed&dopt=Abstract&list_uids=27323409|6|Oncotarget|||Jun|2016|JOURNAL ARTICLE|http://www.ncbi.nlm.nih.gov/entrez/query.fcgi?cmd=Retrieve&db=pubmed&dopt=Abstract&list_uids=27602956|6|Oncotarget|||Sep|2016|JOURNAL ARTICLE|http://www.ncbi.nlm.nih.gov/entrez/query.fcgi?cmd=Retrieve&db=pubmed&dopt=Abstract&list_uids=27502396|6|BMC Cancer|16|||2016|Journal Article
na	mapk14	http://www.ncbi.nlm.nih.gov/entrez/query.fcgi?cmd=Retrieve&db=pubmed&dopt=Abstract&list_uids=27278736|6|Int J Oncol|49|2|Aug|2016|Journal Article
na	ephb2	http://www.ncbi.nlm.nih.gov/entrez/query.fcgi?cmd=Retrieve&db=pubmed&dopt=Abstract&list_uids=27278736|6|Int J Oncol|49|2|Aug|2016|Journal Article|http://www.ncbi.nlm.nih.gov/entrez/query.fcgi?cmd=Retrieve&db=pubmed&dopt=Abstract&list_uids=27572503|6|Oncol Rep|||Aug|2016|JOURNAL ARTICLE
na	mmp9	http://www.ncbi.nlm.nih.gov/entrez/query.fcgi?cmd=Retrieve&db=pubmed&dopt=Abstract&list_uids=27572503|6|Oncol Rep|||Aug|2016|JOURNAL ARTICLE
na	mmp2	http://www.ncbi.nlm.nih.gov/entrez/query.fcgi?cmd=Retrieve&db=pubmed&dopt=Abstract&list_uids=27572503|6|Oncol Rep|||Aug|2016|JOURNAL ARTICLE
na	ros1	http://www.ncbi.nlm.nih.gov/entrez/query.fcgi?cmd=Retrieve&db=pubmed&dopt=Abstract&list_uids=27572503|6|Oncol Rep|||Aug|2016|JOURNAL ARTICLE
axpc1	pcsk1	http://www.ncbi.nlm.nih.gov/entrez/query.fcgi?cmd=Retrieve&db=pubmed&dopt=Abstract&list_uids=27569589|6|Life Sci|||Aug|2016|JOURNAL ARTICLE
loxl2	axpc1	http://www.ncbi.nlm.nih.gov/entrez/query.fcgi?cmd=Retrieve&db=pubmed&dopt=Abstract&list_uids=27278788|6|J Hum Genet|||Jun|2016|JOURNAL ARTICLE
bad	chek1	http://www.ncbi.nlm.nih.gov/entrez/query.fcgi?cmd=Retrieve&db=pubmed&dopt=Abstract&list_uids=27279256|6|Int J Oncol|49|2|Aug|2016|Journal Article
bad	tp53	http://www.ncbi.nlm.nih.gov/entrez/query.fcgi?cmd=Retrieve&db=pubmed&dopt=Abstract&list_uids=27279256|6|Int J Oncol|49|2|Aug|2016|Journal Article
twist1	h2afx	http://www.ncbi.nlm.nih.gov/entrez/query.fcgi?cmd=Retrieve&db=pubmed&dopt=Abstract&list_uids=27315462|6|Cell Cycle|15|18|Sep|2016|Journal Article
twist1	uts2r	http://www.ncbi.nlm.nih.gov/entrez/query.fcgi?cmd=Retrieve&db=pubmed&dopt=Abstract&list_uids=27460728|6|Eur Rev Med Pharmacol Sci|20|14|Jul|2016|Journal Article
twist1	fos	http://www.ncbi.nlm.nih.gov/entrez/query.fcgi?cmd=Retrieve&db=pubmed&dopt=Abstract&list_uids=27524420|6|Oncogene|||Aug|2016|JOURNAL ARTICLE
twist1	ervk2	http://www.ncbi.nlm.nih.gov/entrez/query.fcgi?cmd=Retrieve&db=pubmed&dopt=Abstract&list_uids=27524420|6|Oncogene|||Aug|2016|JOURNAL ARTICLE

twist1	itk	http://www.ncbi.nlm.nih.gov/entrez/query.fcgi?cmd=Retrieve&db=pubmed&dopt=Abstract&list_uids=27279287|6|Oncol Rep|36|2|Aug|2016|Journal Article|http://www.ncbi.nlm.nih.gov/entrez/query.fcgi?cmd=Retrieve&db=pubmed&dopt=Abstract&list_uids=27524420|6|Oncogene|||Aug|2016|JOURNAL ARTICLE
twist1	foxa1	http://www.ncbi.nlm.nih.gov/entrez/query.fcgi?cmd=Retrieve&db=pubmed&dopt=Abstract&list_uids=27524420|6|Oncogene|||Aug|2016|JOURNAL ARTICLE
twist1	magea4	http://www.ncbi.nlm.nih.gov/entrez/query.fcgi?cmd=Retrieve&db=pubmed&dopt=Abstract&list_uids=27533647|6|Mol Carcinog|||Aug|2016|JOURNAL ARTICLE
foxm1	twist1	http://www.ncbi.nlm.nih.gov/entrez/query.fcgi?cmd=Retrieve&db=pubmed&dopt=Abstract&list_uids=27279287|6|Oncol Rep|36|2|Aug|2016|Journal Article
smad4	itk	http://www.ncbi.nlm.nih.gov/entrez/query.fcgi?cmd=Retrieve&db=pubmed&dopt=Abstract&list_uids=27279345|6|Int J Oncol|49|2|Aug|2016|Journal Article
smad4	uts2r	http://www.ncbi.nlm.nih.gov/entrez/query.fcgi?cmd=Retrieve&db=pubmed&dopt=Abstract&list_uids=27279345|6|Int J Oncol|49|2|Aug|2016|Journal Article|http://www.ncbi.nlm.nih.gov/entrez/query.fcgi?cmd=Retrieve&db=pubmed&dopt=Abstract&list_uids=27286257|6|Oncotarget|||Jun|2016|JOURNAL ARTICLE
smad4	spp1	http://www.ncbi.nlm.nih.gov/entrez/query.fcgi?cmd=Retrieve&db=pubmed&dopt=Abstract&list_uids=27498705|6|Oncol Rep|||Aug|2016|JOURNAL ARTICLE
smad4	lef1	http://www.ncbi.nlm.nih.gov/entrez/query.fcgi?cmd=Retrieve&db=pubmed&dopt=Abstract&list_uids=27595937|6|Sci Rep|6|||2016|Journal Article
smad4	hpse	http://www.ncbi.nlm.nih.gov/entrez/query.fcgi?cmd=Retrieve&db=pubmed&dopt=Abstract&list_uids=27595937|6|Sci Rep|6|||2016|Journal Article
mapk7	mapk8	http://www.ncbi.nlm.nih.gov/entrez/query.fcgi?cmd=Retrieve&db=pubmed&dopt=Abstract&list_uids=27279438|6|Oncol Rep|36|2|Aug|2016|Journal Article
rela	hcc	http://www.ncbi.nlm.nih.gov/entrez/query.fcgi?cmd=Retrieve&db=pubmed&dopt=Abstract&list_uids=27486225|6|Mol Cancer Ther|||Aug|2016|JOURNAL ARTICLE
rela	macc1	http://www.ncbi.nlm.nih.gov/entrez/query.fcgi?cmd=Retrieve&db=pubmed&dopt=Abstract&list_uids=27529686|6|Int J Cancer|||Aug|2016|JOURNAL ARTICLE
rela	mapk14	http://www.ncbi.nlm.nih.gov/entrez/query.fcgi?cmd=Retrieve&db=pubmed&dopt=Abstract&list_uids=27529686|6|Int J Cancer|||Aug|2016|JOURNAL ARTICLE|http://www.ncbi.nlm.nih.gov/entrez/query.fcgi?cmd=Retrieve&db=pubmed&dopt=Abstract&list_uids=27393618|6|Breast Cancer Res Treat|158|3|Aug|2016|Journal Article
tlr4	rela	http://www.ncbi.nlm.nih.gov/entrez/query.fcgi?cmd=Retrieve&db=pubmed&dopt=Abstract&list_uids=27279714|6|Pharmacogn Mag|12|Suppl 2|May|2016|Journal Article
tlr4	irf6	http://www.ncbi.nlm.nih.gov/entrez/query.fcgi?cmd=Retrieve&db=pubmed&dopt=Abstract&list_uids=27409669|6|Oncotarget|||Jul|2016|JOURNAL ARTICLE

tlr4	ly96	http://www.ncbi.nlm.nih.gov/entrez/query.fcgi?cmd=Retrieve&db=pubmed&dopt=Abstract&list_uids=27409669|6|Oncotarget|||Jul|2016|JOURNAL ARTICLE
tlr4	mapk14	http://www.ncbi.nlm.nih.gov/entrez/query.fcgi?cmd=Retrieve&db=pubmed&dopt=Abstract&list_uids=27446390|6|Oncol Lett|12|2|Aug|2016|JOURNAL ARTICLE
tlr4	map3k5	http://www.ncbi.nlm.nih.gov/entrez/query.fcgi?cmd=Retrieve&db=pubmed&dopt=Abstract&list_uids=27446390|6|Oncol Lett|12|2|Aug|2016|JOURNAL ARTICLE
tlr4	myd88	http://www.ncbi.nlm.nih.gov/entrez/query.fcgi?cmd=Retrieve&db=pubmed&dopt=Abstract&list_uids=27446390|6|Oncol Lett|12|2|Aug|2016|JOURNAL ARTICLE
muc4	ca9	http://www.ncbi.nlm.nih.gov/entrez/query.fcgi?cmd=Retrieve&db=pubmed&dopt=Abstract&list_uids=27283771|6|Oncotarget|||Jun|2016|JOURNAL ARTICLE
muc4	aicda	http://www.ncbi.nlm.nih.gov/entrez/query.fcgi?cmd=Retrieve&db=pubmed&dopt=Abstract&list_uids=27283771|6|Oncotarget|||Jun|2016|JOURNAL ARTICLE
muc4	3.5.1.4	http://www.ncbi.nlm.nih.gov/entrez/query.fcgi?cmd=Retrieve&db=pubmed&dopt=Abstract&list_uids=27283771|6|Oncotarget|||Jun|2016|JOURNAL ARTICLE
muc4	eno3	http://www.ncbi.nlm.nih.gov/entrez/query.fcgi?cmd=Retrieve&db=pubmed&dopt=Abstract&list_uids=27283771|6|Oncotarget|||Jun|2016|JOURNAL ARTICLE
muc1	ca9	http://www.ncbi.nlm.nih.gov/entrez/query.fcgi?cmd=Retrieve&db=pubmed&dopt=Abstract&list_uids=27283771|6|Oncotarget|||Jun|2016|JOURNAL ARTICLE
muc1	aicda	http://www.ncbi.nlm.nih.gov/entrez/query.fcgi?cmd=Retrieve&db=pubmed&dopt=Abstract&list_uids=27283771|6|Oncotarget|||Jun|2016|JOURNAL ARTICLE
muc1	3.5.1.4	http://www.ncbi.nlm.nih.gov/entrez/query.fcgi?cmd=Retrieve&db=pubmed&dopt=Abstract&list_uids=27283771|6|Oncotarget|||Jun|2016|JOURNAL ARTICLE
muc1	eno3	http://www.ncbi.nlm.nih.gov/entrez/query.fcgi?cmd=Retrieve&db=pubmed&dopt=Abstract&list_uids=27283771|6|Oncotarget|||Jun|2016|JOURNAL ARTICLE
muc1	muc4	http://www.ncbi.nlm.nih.gov/entrez/query.fcgi?cmd=Retrieve&db=pubmed&dopt=Abstract&list_uids=27283771|6|Oncotarget|||Jun|2016|JOURNAL ARTICLE
aicda	ca9	http://www.ncbi.nlm.nih.gov/entrez/query.fcgi?cmd=Retrieve&db=pubmed&dopt=Abstract&list_uids=27283771|6|Oncotarget|||Jun|2016|JOURNAL ARTICLE
3.5.1.4	ca9	http://www.ncbi.nlm.nih.gov/entrez/query.fcgi?cmd=Retrieve&db=pubmed&dopt=Abstract&list_uids=27283771|6|Oncotarget|||Jun|2016|JOURNAL ARTICLE
3.5.1.4	aicda	http://www.ncbi.nlm.nih.gov/entrez/query.fcgi?cmd=Retrieve&db=pubmed&dopt=Abstract&list_uids=27283771|6|Oncotarget|||Jun|2016|JOURNAL ARTICLE
mlxip	snord87	http://www.ncbi.nlm.nih.gov/entrez/query.fcgi?cmd=Retrieve&db=pubmed&dopt=Abstract&list_uids=27323092|6|Genet Mol Res|15|2||2016|Journal Article
mlxip	tp53	http://www.ncbi.nlm.nih.gov/entrez/query.fcgi?cmd=Retrieve&db=pubmed&dopt=Abstract&list_uids=27472451|6|Med Sci Monit|22|||2016|Journal Article

mlxip	mirn132	http://www.ncbi.nlm.nih.gov/entrez/query.fcgi?cmd=Retrieve&db=pubmed&dopt=Abstract&list_uids=27522003|6|Biomed Pharmacother|83||Aug|2016|JOURNAL ARTICLE
klf5	mlxip	http://www.ncbi.nlm.nih.gov/entrez/query.fcgi?cmd=Retrieve&db=pubmed&dopt=Abstract&list_uids=27284339|6|Exp Ther Med|11|6|Jun|2016|JOURNAL ARTICLE
hulc	itk	http://www.ncbi.nlm.nih.gov/entrez/query.fcgi?cmd=Retrieve&db=pubmed&dopt=Abstract&list_uids=27285757|6|Oncotarget|||Jun|2016|JOURNAL ARTICLE
hulc	hcc	http://www.ncbi.nlm.nih.gov/entrez/query.fcgi?cmd=Retrieve&db=pubmed&dopt=Abstract&list_uids=27285757|6|Oncotarget|||Jun|2016|JOURNAL ARTICLE
hulc	zeb1	http://www.ncbi.nlm.nih.gov/entrez/query.fcgi?cmd=Retrieve&db=pubmed&dopt=Abstract&list_uids=27285757|6|Oncotarget|||Jun|2016|JOURNAL ARTICLE
hulc	nkd2	http://www.ncbi.nlm.nih.gov/entrez/query.fcgi?cmd=Retrieve&db=pubmed&dopt=Abstract&list_uids=27496341|6|Gene|592|1|Oct|2016|Journal Article
zeb1	mirn200b	http://www.ncbi.nlm.nih.gov/entrez/query.fcgi?cmd=Retrieve&db=pubmed&dopt=Abstract&list_uids=27307751|6|Onco Targets Ther|9|||2016|Journal Article
zeb1	stat3	http://www.ncbi.nlm.nih.gov/entrez/query.fcgi?cmd=Retrieve&db=pubmed&dopt=Abstract&list_uids=27471397|6|Onco Targets Ther|9|||2016|Journal Article
zeb1	jak2	http://www.ncbi.nlm.nih.gov/entrez/query.fcgi?cmd=Retrieve&db=pubmed&dopt=Abstract&list_uids=27471397|6|Onco Targets Ther|9|||2016|Journal Article
pbx3	hcc	http://www.ncbi.nlm.nih.gov/entrez/query.fcgi?cmd=Retrieve&db=pubmed&dopt=Abstract&list_uids=27285759|6|Oncotarget|||Jun|2016|JOURNAL ARTICLE
sfmbt1	dcun1d1	http://www.ncbi.nlm.nih.gov/entrez/query.fcgi?cmd=Retrieve&db=pubmed&dopt=Abstract&list_uids=27285984|6|Oncotarget|||Jun|2016|JOURNAL ARTICLE
prtn3	dcun1d1	http://www.ncbi.nlm.nih.gov/entrez/query.fcgi?cmd=Retrieve&db=pubmed&dopt=Abstract&list_uids=27285984|6|Oncotarget|||Jun|2016|JOURNAL ARTICLE
erbb2	zeb1	http://www.ncbi.nlm.nih.gov/entrez/query.fcgi?cmd=Retrieve&db=pubmed&dopt=Abstract&list_uids=27286256|6|Oncotarget|||Jun|2016|JOURNAL ARTICLE
erbb2	tjp1	http://www.ncbi.nlm.nih.gov/entrez/query.fcgi?cmd=Retrieve&db=pubmed&dopt=Abstract&list_uids=27286256|6|Oncotarget|||Jun|2016|JOURNAL ARTICLE
erbb2	cldn7	http://www.ncbi.nlm.nih.gov/entrez/query.fcgi?cmd=Retrieve&db=pubmed&dopt=Abstract&list_uids=27286256|6|Oncotarget|||Jun|2016|JOURNAL ARTICLE
erbb2	ptk2	http://www.ncbi.nlm.nih.gov/entrez/query.fcgi?cmd=Retrieve&db=pubmed&dopt=Abstract&list_uids=27286256|6|Oncotarget|||Jun|2016|JOURNAL ARTICLE
erbb2	creb1	http://www.ncbi.nlm.nih.gov/entrez/query.fcgi?cmd=Retrieve&db=pubmed&dopt=Abstract&list_uids=27409833|6|Oncotarget|||Jul|2016|JOURNAL ARTICLE
tjp1	zeb1	http://www.ncbi.nlm.nih.gov/entrez/query.fcgi?cmd=Retrieve&db=pubmed&dopt=Abstract&list_uids=27286256|6|Oncotarget|||Jun|2016|JOURNAL ARTICLE

cox8a	smad4	http://www.ncbi.nlm.nih.gov/entrez/query.fcgi?cmd=Retrieve&db=pubmed&dopt=Abstract&list_uids=27286257|6|Oncotarget|||Jun|2016|JOURNAL ARTICLE
cox8a	ptger4	http://www.ncbi.nlm.nih.gov/entrez/query.fcgi?cmd=Retrieve&db=pubmed&dopt=Abstract&list_uids=27301070|6|Stem Cells|34|9|Sep|2016|Journal Article
cox8a	dbt	http://www.ncbi.nlm.nih.gov/entrez/query.fcgi?cmd=Retrieve&db=pubmed&dopt=Abstract&list_uids=27301070|6|Stem Cells|34|9|Sep|2016|Journal Article
egfr	ccnd1	http://www.ncbi.nlm.nih.gov/entrez/query.fcgi?cmd=Retrieve&db=pubmed&dopt=Abstract&list_uids=27364926|6|Sci Rep|6|||2016|Journal Article
egfr	akt2	http://www.ncbi.nlm.nih.gov/entrez/query.fcgi?cmd=Retrieve&db=pubmed&dopt=Abstract&list_uids=27364926|6|Sci Rep|6|||2016|Journal Article
egfr	igf1r	http://www.ncbi.nlm.nih.gov/entrez/query.fcgi?cmd=Retrieve&db=pubmed&dopt=Abstract&list_uids=27374103|6|Oncotarget|||Jun|2016|JOURNAL ARTICLE
egfr	insr	http://www.ncbi.nlm.nih.gov/entrez/query.fcgi?cmd=Retrieve&db=pubmed&dopt=Abstract&list_uids=27374103|6|Oncotarget|||Jun|2016|JOURNAL ARTICLE
egfr	akt1	http://www.ncbi.nlm.nih.gov/entrez/query.fcgi?cmd=Retrieve&db=pubmed&dopt=Abstract&list_uids=27317099|6|J Exp Clin Cancer Res|35|1||2016|Journal Article|http://www.ncbi.nlm.nih.gov/entrez/query.fcgi?cmd=Retrieve&db=pubmed&dopt=Abstract&list_uids=27510965|6|Biomedicine (Taipei)|6|3|Jun|2016|Journal Article
egfr	mmp9	http://www.ncbi.nlm.nih.gov/entrez/query.fcgi?cmd=Retrieve&db=pubmed&dopt=Abstract&list_uids=27573547|6|Int J Oncol|49|3|Sep|2016|Journal Article
egfr	ddx6	http://www.ncbi.nlm.nih.gov/entrez/query.fcgi?cmd=Retrieve&db=pubmed&dopt=Abstract&list_uids=27609774|6|FASEB J|||Sep|2016|JOURNAL ARTICLE
egf	rela	http://www.ncbi.nlm.nih.gov/entrez/query.fcgi?cmd=Retrieve&db=pubmed&dopt=Abstract&list_uids=27470348|6|Biomed Pharmacother|82||Aug|2016|Journal Article
egf	mmp2	http://www.ncbi.nlm.nih.gov/entrez/query.fcgi?cmd=Retrieve&db=pubmed&dopt=Abstract&list_uids=27470348|6|Biomed Pharmacother|82||Aug|2016|Journal Article
egf	pcna	http://www.ncbi.nlm.nih.gov/entrez/query.fcgi?cmd=Retrieve&db=pubmed&dopt=Abstract&list_uids=27470348|6|Biomed Pharmacother|82||Aug|2016|Journal Article
egf	bcl2	http://www.ncbi.nlm.nih.gov/entrez/query.fcgi?cmd=Retrieve&db=pubmed&dopt=Abstract&list_uids=27470348|6|Biomed Pharmacother|82||Aug|2016|Journal Article
egf	ptk2	http://www.ncbi.nlm.nih.gov/entrez/query.fcgi?cmd=Retrieve&db=pubmed&dopt=Abstract&list_uids=27573547|6|Int J Oncol|49|3|Sep|2016|Journal Article
egf	mmp9	http://www.ncbi.nlm.nih.gov/entrez/query.fcgi?cmd=Retrieve&db=pubmed&dopt=Abstract&list_uids=27470348|6|Biomed Pharmacother|82||Aug|2016|Journal Article|http://www.ncbi.nlm.nih.gov/entrez/query.fcgi?cmd=Retrieve&db=pubmed&dopt=Abstract&list_uids=27573547|6|Int J Oncol|49|3|Sep|2016|Journal Article

egf	egfr	http://www.ncbi.nlm.nih.gov/entrez/query.fcgi?cmd=Retrieve&db=pubmed&dopt=Abstract&list_uids=27609774|6|FASEB J|||Sep|2016|JOURNAL ARTICLE|http://www.ncbi.nlm.nih.gov/entrez/query.fcgi?cmd=Retrieve&db=pubmed&dopt=Abstract&list_uids=27286795|6|Neuro Oncol|||Jun|2016|JOURNAL ARTICLE|http://www.ncbi.nlm.nih.gov/entrez/query.fcgi?cmd=Retrieve&db=pubmed&dopt=Abstract&list_uids=27452449|6|Cell Transplant|||Jul|2016|JOURNAL ARTICLE
cd34	tfpi	http://www.ncbi.nlm.nih.gov/entrez/query.fcgi?cmd=Retrieve&db=pubmed&dopt=Abstract&list_uids=27352134|6|PLoS One|11|6||2016|Journal Article
prom1	cd34	http://www.ncbi.nlm.nih.gov/entrez/query.fcgi?cmd=Retrieve&db=pubmed&dopt=Abstract&list_uids=27288104|6|J Vasc Surg|||Jun|2016|JOURNAL ARTICLE
prom1	stat6	http://www.ncbi.nlm.nih.gov/entrez/query.fcgi?cmd=Retrieve&db=pubmed&dopt=Abstract&list_uids=27533463|6|Oncotarget|||Aug|2016|JOURNAL ARTICLE
prom1	alcam	http://www.ncbi.nlm.nih.gov/entrez/query.fcgi?cmd=Retrieve&db=pubmed&dopt=Abstract&list_uids=27533463|6|Oncotarget|||Aug|2016|JOURNAL ARTICLE
kdr	cd34	http://www.ncbi.nlm.nih.gov/entrez/query.fcgi?cmd=Retrieve&db=pubmed&dopt=Abstract&list_uids=27288104|6|J Vasc Surg|||Jun|2016|JOURNAL ARTICLE
kdr	prom1	http://www.ncbi.nlm.nih.gov/entrez/query.fcgi?cmd=Retrieve&db=pubmed&dopt=Abstract&list_uids=27288104|6|J Vasc Surg|||Jun|2016|JOURNAL ARTICLE
kdr	ros1	http://www.ncbi.nlm.nih.gov/entrez/query.fcgi?cmd=Retrieve&db=pubmed&dopt=Abstract&list_uids=27498749|6|Int J Oncol|||Aug|2016|JOURNAL ARTICLE
kdr	lect2	http://www.ncbi.nlm.nih.gov/entrez/query.fcgi?cmd=Retrieve&db=pubmed&dopt=Abstract&list_uids=27507763|6|Sci Rep|6|||2016|Journal Article
kdr	vegfa	http://www.ncbi.nlm.nih.gov/entrez/query.fcgi?cmd=Retrieve&db=pubmed&dopt=Abstract&list_uids=27569654|6|Cancer Lett|382|1|Aug|2016|JOURNAL ARTICLE|http://www.ncbi.nlm.nih.gov/entrez/query.fcgi?cmd=Retrieve&db=pubmed&dopt=Abstract&list_uids=27297262|6|Clin Exp Pharmacol Physiol|43|10|Oct|2016|Journal Article|http://www.ncbi.nlm.nih.gov/entrez/query.fcgi?cmd=Retrieve&db=pubmed&dopt=Abstract&list_uids=27498749|6|Int J Oncol|||Aug|2016|JOURNAL ARTICLE|http://www.ncbi.nlm.nih.gov/entrez/query.fcgi?cmd=Retrieve&db=pubmed&dopt=Abstract&list_uids=27507763|6|Sci Rep|6|||2016|Journal Article
kdr	ephb2	http://www.ncbi.nlm.nih.gov/entrez/query.fcgi?cmd=Retrieve&db=pubmed&dopt=Abstract&list_uids=27569654|6|Cancer Lett|382|1|Aug|2016|JOURNAL ARTICLE|http://www.ncbi.nlm.nih.gov/entrez/query.fcgi?cmd=Retrieve&db=pubmed&dopt=Abstract&list_uids=27572273|6|Int J Oncol|49|3|Sep|2016|Journal Article
kdr	zhx2	http://www.ncbi.nlm.nih.gov/entrez/query.fcgi?cmd=Retrieve&db=pubmed&dopt=Abstract&list_uids=27572273|6|Int J Oncol|49|3|Sep|2016|Journal Article

kdr	akt1	http://www.ncbi.nlm.nih.gov/entrez/query.fcgi?cmd=Retrieve&db=pubmed&dopt=Abstract&list_uids=27572273|6|Int J Oncol|49|3|Sep|2016|Journal Article|http://www.ncbi.nlm.nih.gov/entrez/query.fcgi?cmd=Retrieve&db=pubmed&dopt=Abstract&list_uids=27297262|6|Clin Exp Pharmacol Physiol|43|10|Oct|2016|Journal Article
kdr	pik3ca	http://www.ncbi.nlm.nih.gov/entrez/query.fcgi?cmd=Retrieve&db=pubmed&dopt=Abstract&list_uids=27572273|6|Int J Oncol|49|3|Sep|2016|Journal Article
parp1	cd34	http://www.ncbi.nlm.nih.gov/entrez/query.fcgi?cmd=Retrieve&db=pubmed&dopt=Abstract&list_uids=27288104|6|J Vasc Surg|||Jun|2016|JOURNAL ARTICLE
parp1	prom1	http://www.ncbi.nlm.nih.gov/entrez/query.fcgi?cmd=Retrieve&db=pubmed&dopt=Abstract&list_uids=27288104|6|J Vasc Surg|||Jun|2016|JOURNAL ARTICLE
parp1	kdr	http://www.ncbi.nlm.nih.gov/entrez/query.fcgi?cmd=Retrieve&db=pubmed&dopt=Abstract&list_uids=27288104|6|J Vasc Surg|||Jun|2016|JOURNAL ARTICLE
parp1	bcl2l1	http://www.ncbi.nlm.nih.gov/entrez/query.fcgi?cmd=Retrieve&db=pubmed&dopt=Abstract&list_uids=27415751|6|Cancer Biol Ther|||Jul|2016|JOURNAL ARTICLE
parp1	dym	http://www.ncbi.nlm.nih.gov/entrez/query.fcgi?cmd=Retrieve&db=pubmed&dopt=Abstract&list_uids=27614381|6|Med Sci Monit|22||||JOURNAL ARTICLE
stim1	3.6.1.8	http://www.ncbi.nlm.nih.gov/entrez/query.fcgi?cmd=Retrieve&db=pubmed&dopt=Abstract&list_uids=27291153|6|Biochem Biophys Res Commun|477|1|Aug|2016|Journal Article
stim1	macc1	http://www.ncbi.nlm.nih.gov/entrez/query.fcgi?cmd=Retrieve&db=pubmed&dopt=Abstract&list_uids=27431311|6|Cancer Lett|381|1|Oct|2016|Journal Article
orai1	3.6.1.8	http://www.ncbi.nlm.nih.gov/entrez/query.fcgi?cmd=Retrieve&db=pubmed&dopt=Abstract&list_uids=27291153|6|Biochem Biophys Res Commun|477|1|Aug|2016|Journal Article
orai1	macc1	http://www.ncbi.nlm.nih.gov/entrez/query.fcgi?cmd=Retrieve&db=pubmed&dopt=Abstract&list_uids=27431311|6|Cancer Lett|381|1|Oct|2016|Journal Article
orai1	stim1	http://www.ncbi.nlm.nih.gov/entrez/query.fcgi?cmd=Retrieve&db=pubmed&dopt=Abstract&list_uids=27291153|6|Biochem Biophys Res Commun|477|1|Aug|2016|Journal Article|http://www.ncbi.nlm.nih.gov/entrez/query.fcgi?cmd=Retrieve&db=pubmed&dopt=Abstract&list_uids=27431311|6|Cancer Lett|381|1|Oct|2016|Journal Article
rhd	3.6.1.8	http://www.ncbi.nlm.nih.gov/entrez/query.fcgi?cmd=Retrieve&db=pubmed&dopt=Abstract&list_uids=27291153|6|Biochem Biophys Res Commun|477|1|Aug|2016|Journal Article
rhd	stim1	http://www.ncbi.nlm.nih.gov/entrez/query.fcgi?cmd=Retrieve&db=pubmed&dopt=Abstract&list_uids=27291153|6|Biochem Biophys Res Commun|477|1|Aug|2016|Journal Article
rhd	orai1	http://www.ncbi.nlm.nih.gov/entrez/query.fcgi?cmd=Retrieve&db=pubmed&dopt=Abstract&list_uids=27291153|6|Biochem Biophys Res Commun|477|1|Aug|2016|Journal Article
actin	hcc	http://www.ncbi.nlm.nih.gov/entrez/query.fcgi?cmd=Retrieve&db=pubmed&dopt=Abstract&list_uids=27459117|6|Biomed Pharmacother|83||Jul|2016|JOURNAL ARTICLE

actin	id1	http://www.ncbi.nlm.nih.gov/entrez/query.fcgi?cmd=Retrieve&db=pubmed&dopt=Abstract&list_uids=27459117|6|Biomed Pharmacother|83||Jul|2016|JOURNAL ARTICLE
actin	pfn1	http://www.ncbi.nlm.nih.gov/entrez/query.fcgi?cmd=Retrieve&db=pubmed&dopt=Abstract&list_uids=27494863|6|Oncotarget|||Aug|2016|JOURNAL ARTICLE
actin	ppa1	http://www.ncbi.nlm.nih.gov/entrez/query.fcgi?cmd=Retrieve&db=pubmed&dopt=Abstract&list_uids=27608898|6|Eur Rev Med Pharmacol Sci|20|16|Aug|2016|Journal Article
actin	phactr4	http://www.ncbi.nlm.nih.gov/entrez/query.fcgi?cmd=Retrieve&db=pubmed&dopt=Abstract&list_uids=27608898|6|Eur Rev Med Pharmacol Sci|20|16|Aug|2016|Journal Article
trpv4	actin	http://www.ncbi.nlm.nih.gov/entrez/query.fcgi?cmd=Retrieve&db=pubmed&dopt=Abstract&list_uids=27291497|6|Sci Rep|6|||2016|Journal Article
smad2	hcc	http://www.ncbi.nlm.nih.gov/entrez/query.fcgi?cmd=Retrieve&db=pubmed&dopt=Abstract&list_uids=27292026|6|Neoplasia|18|6|Jun|2016|Journal Article
smad2	stat3	http://www.ncbi.nlm.nih.gov/entrez/query.fcgi?cmd=Retrieve&db=pubmed&dopt=Abstract&list_uids=27292026|6|Neoplasia|18|6|Jun|2016|Journal Article
smad2	jak1	http://www.ncbi.nlm.nih.gov/entrez/query.fcgi?cmd=Retrieve&db=pubmed&dopt=Abstract&list_uids=27292026|6|Neoplasia|18|6|Jun|2016|Journal Article
smad2	kitlg	http://www.ncbi.nlm.nih.gov/entrez/query.fcgi?cmd=Retrieve&db=pubmed&dopt=Abstract&list_uids=27292026|6|Neoplasia|18|6|Jun|2016|Journal Article
smad2	tnfsf13	http://www.ncbi.nlm.nih.gov/entrez/query.fcgi?cmd=Retrieve&db=pubmed&dopt=Abstract&list_uids=27292026|6|Neoplasia|18|6|Jun|2016|Journal Article
smad2	timp3	http://www.ncbi.nlm.nih.gov/entrez/query.fcgi?cmd=Retrieve&db=pubmed&dopt=Abstract&list_uids=27383203|6|Cell Physiol Biochem|39|2||2016|Journal Article
smad2	ski	http://www.ncbi.nlm.nih.gov/entrez/query.fcgi?cmd=Retrieve&db=pubmed&dopt=Abstract&list_uids=27473823|6|Cancer Lett|381|1|Oct|2016|Journal Article
smad2	snord87	http://www.ncbi.nlm.nih.gov/entrez/query.fcgi?cmd=Retrieve&db=pubmed&dopt=Abstract&list_uids=27574009|6|Oncol Rep|||Aug|2016|JOURNAL ARTICLE
smad2	smad7	http://www.ncbi.nlm.nih.gov/entrez/query.fcgi?cmd=Retrieve&db=pubmed&dopt=Abstract&list_uids=27473823|6|Cancer Lett|381|1|Oct|2016|Journal Article|http://www.ncbi.nlm.nih.gov/entrez/query.fcgi?cmd=Retrieve&db=pubmed&dopt=Abstract&list_uids=27574009|6|Oncol Rep|||Aug|2016|JOURNAL ARTICLE
kitlg	hcc	http://www.ncbi.nlm.nih.gov/entrez/query.fcgi?cmd=Retrieve&db=pubmed&dopt=Abstract&list_uids=27292026|6|Neoplasia|18|6|Jun|2016|Journal Article
kitlg	stat3	http://www.ncbi.nlm.nih.gov/entrez/query.fcgi?cmd=Retrieve&db=pubmed&dopt=Abstract&list_uids=27292026|6|Neoplasia|18|6|Jun|2016|Journal Article
kitlg	jak1	http://www.ncbi.nlm.nih.gov/entrez/query.fcgi?cmd=Retrieve&db=pubmed&dopt=Abstract&list_uids=27292026|6|Neoplasia|18|6|Jun|2016|Journal Article

tnfsf13	stat3	http://www.ncbi.nlm.nih.gov/entrez/query.fcgi?cmd=Retrieve&db=pubmed&dopt=Abstract&list_uids=27292026|6|Neoplasia|18|6|Jun|2016|Journal Article
tnfsf13	jak1	http://www.ncbi.nlm.nih.gov/entrez/query.fcgi?cmd=Retrieve&db=pubmed&dopt=Abstract&list_uids=27292026|6|Neoplasia|18|6|Jun|2016|Journal Article
tnfsf13	kitlg	http://www.ncbi.nlm.nih.gov/entrez/query.fcgi?cmd=Retrieve&db=pubmed&dopt=Abstract&list_uids=27292026|6|Neoplasia|18|6|Jun|2016|Journal Article
tnfsf13	scaf1	http://www.ncbi.nlm.nih.gov/entrez/query.fcgi?cmd=Retrieve&db=pubmed&dopt=Abstract&list_uids=27367025|6|Oncotarget|||Jun|2016|JOURNAL ARTICLE
tnfsf13	mmp9	http://www.ncbi.nlm.nih.gov/entrez/query.fcgi?cmd=Retrieve&db=pubmed&dopt=Abstract&list_uids=27452283|6|Bioorg Med Chem|24|18|Sep|2016|Journal Article
tnfsf13	mmp2	http://www.ncbi.nlm.nih.gov/entrez/query.fcgi?cmd=Retrieve&db=pubmed&dopt=Abstract&list_uids=27452283|6|Bioorg Med Chem|24|18|Sep|2016|Journal Article
tnfsf13	tek	http://www.ncbi.nlm.nih.gov/entrez/query.fcgi?cmd=Retrieve&db=pubmed&dopt=Abstract&list_uids=27519652|6|J Invest Dermatol|||Aug|2016|JOURNAL ARTICLE
tnfsf13	darc	http://www.ncbi.nlm.nih.gov/entrez/query.fcgi?cmd=Retrieve&db=pubmed&dopt=Abstract&list_uids=27555313|6|MBio|7|4||2016|Journal Article
tnfsf13	il6	http://www.ncbi.nlm.nih.gov/entrez/query.fcgi?cmd=Retrieve&db=pubmed&dopt=Abstract&list_uids=27558278|6|J Periodontal Res|||Aug|2016|JOURNAL ARTICLE
tnfsf13	il8	http://www.ncbi.nlm.nih.gov/entrez/query.fcgi?cmd=Retrieve&db=pubmed&dopt=Abstract&list_uids=27558278|6|J Periodontal Res|||Aug|2016|JOURNAL ARTICLE
tnfsf13	ccl20	http://www.ncbi.nlm.nih.gov/entrez/query.fcgi?cmd=Retrieve&db=pubmed&dopt=Abstract&list_uids=27558278|6|J Periodontal Res|||Aug|2016|JOURNAL ARTICLE
soat1	stat3	http://www.ncbi.nlm.nih.gov/entrez/query.fcgi?cmd=Retrieve&db=pubmed&dopt=Abstract&list_uids=27292260|6|Oncogene|||Jun|2016|JOURNAL ARTICLE
myst1	itk	http://www.ncbi.nlm.nih.gov/entrez/query.fcgi?cmd=Retrieve&db=pubmed&dopt=Abstract&list_uids=27292636|6|Cell Rep|15|12|Jun|2016|Journal Article
myst1	aof2	http://www.ncbi.nlm.nih.gov/entrez/query.fcgi?cmd=Retrieve&db=pubmed&dopt=Abstract&list_uids=27292636|6|Cell Rep|15|12|Jun|2016|Journal Article
yap1	smad2	http://www.ncbi.nlm.nih.gov/entrez/query.fcgi?cmd=Retrieve&db=pubmed&dopt=Abstract&list_uids=27292796|6|Mol Cell|63|1|Jul|2016|Journal Article
yap1	hras	http://www.ncbi.nlm.nih.gov/entrez/query.fcgi?cmd=Retrieve&db=pubmed&dopt=Abstract&list_uids=27380321|6|Nat Chem Biol|12|9|Sep|2016|Journal Article
yap1	itk	http://www.ncbi.nlm.nih.gov/entrez/query.fcgi?cmd=Retrieve&db=pubmed&dopt=Abstract&list_uids=27432214|6|Oncol Rep|36|3|Sep|2016|Journal Article
rassf1	smad2	http://www.ncbi.nlm.nih.gov/entrez/query.fcgi?cmd=Retrieve&db=pubmed&dopt=Abstract&list_uids=27292796|6|Mol Cell|63|1|Jul|2016|Journal Article

rassf1	yap1	http://www.ncbi.nlm.nih.gov/entrez/query.fcgi?cmd=Retrieve&db=pubmed&dopt=Abstract&list_uids=27292796|6|Mol Cell|63|1|Jul|2016|Journal Article
rassf1	itch	http://www.ncbi.nlm.nih.gov/entrez/query.fcgi?cmd=Retrieve&db=pubmed&dopt=Abstract&list_uids=27292796|6|Mol Cell|63|1|Jul|2016|Journal Article
rassf1	uts2r	http://www.ncbi.nlm.nih.gov/entrez/query.fcgi?cmd=Retrieve&db=pubmed&dopt=Abstract&list_uids=27600004|6|Zhong Nan Da Xue Xue Bao Yi Xue Ban|41|8|Aug|2016|English Abstract,Journal Article
bcar1	ddr1	http://www.ncbi.nlm.nih.gov/entrez/query.fcgi?cmd=Retrieve&db=pubmed&dopt=Abstract&list_uids=27293031|6|Sci Rep|6|||2016|Journal Article
bcar1	abl1	http://www.ncbi.nlm.nih.gov/entrez/query.fcgi?cmd=Retrieve&db=pubmed&dopt=Abstract&list_uids=27293031|6|Sci Rep|6|||2016|Journal Article
bcar1	pxn	http://www.ncbi.nlm.nih.gov/entrez/query.fcgi?cmd=Retrieve&db=pubmed&dopt=Abstract&list_uids=27293031|6|Sci Rep|6|||2016|Journal Article
bcar1	ptk2	http://www.ncbi.nlm.nih.gov/entrez/query.fcgi?cmd=Retrieve&db=pubmed&dopt=Abstract&list_uids=27293031|6|Sci Rep|6|||2016|Journal Article
ddr1	ephb2	http://www.ncbi.nlm.nih.gov/entrez/query.fcgi?cmd=Retrieve&db=pubmed&dopt=Abstract&list_uids=27391444|6|Oncotarget|||Jul|2016|JOURNAL ARTICLE
ddr1	rhoa	http://www.ncbi.nlm.nih.gov/entrez/query.fcgi?cmd=Retrieve&db=pubmed&dopt=Abstract&list_uids=27391444|6|Oncotarget|||Jul|2016|JOURNAL ARTICLE
abl1	ddr1	http://www.ncbi.nlm.nih.gov/entrez/query.fcgi?cmd=Retrieve&db=pubmed&dopt=Abstract&list_uids=27293031|6|Sci Rep|6|||2016|Journal Article
sox2	setd2	http://www.ncbi.nlm.nih.gov/entrez/query.fcgi?cmd=Retrieve&db=pubmed&dopt=Abstract&list_uids=27294000|6|Am J Cancer Res|6|5||2016|Journal Article
sox2	stat3	http://www.ncbi.nlm.nih.gov/entrez/query.fcgi?cmd=Retrieve&db=pubmed&dopt=Abstract&list_uids=27472461|6|Oncotarget|||Jul|2016|JOURNAL ARTICLE
dap	kdr	http://www.ncbi.nlm.nih.gov/entrez/query.fcgi?cmd=Retrieve&db=pubmed&dopt=Abstract&list_uids=27297262|6|Clin Exp Pharmacol Physiol|43|10|Oct|2016|Journal Article
dap	akt1	http://www.ncbi.nlm.nih.gov/entrez/query.fcgi?cmd=Retrieve&db=pubmed&dopt=Abstract&list_uids=27297262|6|Clin Exp Pharmacol Physiol|43|10|Oct|2016|Journal Article
dap	mapk3	http://www.ncbi.nlm.nih.gov/entrez/query.fcgi?cmd=Retrieve&db=pubmed&dopt=Abstract&list_uids=27297262|6|Clin Exp Pharmacol Physiol|43|10|Oct|2016|Journal Article
dap	ptk2	http://www.ncbi.nlm.nih.gov/entrez/query.fcgi?cmd=Retrieve&db=pubmed&dopt=Abstract&list_uids=27297262|6|Clin Exp Pharmacol Physiol|43|10|Oct|2016|Journal Article
dap	src	http://www.ncbi.nlm.nih.gov/entrez/query.fcgi?cmd=Retrieve&db=pubmed&dopt=Abstract&list_uids=27297262|6|Clin Exp Pharmacol Physiol|43|10|Oct|2016|Journal Article
dap	vegfa	http://www.ncbi.nlm.nih.gov/entrez/query.fcgi?cmd=Retrieve&db=pubmed&dopt=Abstract&list_uids=27297262|6|Clin Exp Pharmacol Physiol|43|10|Oct|2016|Journal Article

dap	krit1	http://www.ncbi.nlm.nih.gov/entrez/query.fcgi?cmd=Retrieve&db=pubmed&dopt=Abstract&list_uids=27297262|6|Clin Exp Pharmacol Physiol|43|10|Oct|2016|Journal Article
dap	rara	http://www.ncbi.nlm.nih.gov/entrez/query.fcgi?cmd=Retrieve&db=pubmed&dopt=Abstract&list_uids=27297262|6|Clin Exp Pharmacol Physiol|43|10|Oct|2016|Journal Article
dap	fas	http://www.ncbi.nlm.nih.gov/entrez/query.fcgi?cmd=Retrieve&db=pubmed&dopt=Abstract&list_uids=27415751|6|Cancer Biol Ther|||Jul|2016|JOURNAL ARTICLE
dap	stat3	http://www.ncbi.nlm.nih.gov/entrez/query.fcgi?cmd=Retrieve&db=pubmed&dopt=Abstract&list_uids=27415751|6|Cancer Biol Ther|||Jul|2016|JOURNAL ARTICLE
rara	krit1	http://www.ncbi.nlm.nih.gov/entrez/query.fcgi?cmd=Retrieve&db=pubmed&dopt=Abstract&list_uids=27297262|6|Clin Exp Pharmacol Physiol|43|10|Oct|2016|Journal Article
ptger4	ccl21	http://www.ncbi.nlm.nih.gov/entrez/query.fcgi?cmd=Retrieve&db=pubmed&dopt=Abstract&list_uids=27301070|6|Stem Cells|34|9|Sep|2016|Journal Article
ptgs2	ccl21	http://www.ncbi.nlm.nih.gov/entrez/query.fcgi?cmd=Retrieve&db=pubmed&dopt=Abstract&list_uids=27301070|6|Stem Cells|34|9|Sep|2016|Journal Article
ptgs2	akt1	http://www.ncbi.nlm.nih.gov/entrez/query.fcgi?cmd=Retrieve&db=pubmed&dopt=Abstract&list_uids=27301070|6|Stem Cells|34|9|Sep|2016|Journal Article
ptgs2	pik3ca	http://www.ncbi.nlm.nih.gov/entrez/query.fcgi?cmd=Retrieve&db=pubmed&dopt=Abstract&list_uids=27301070|6|Stem Cells|34|9|Sep|2016|Journal Article
ptgs2	ptger4	http://www.ncbi.nlm.nih.gov/entrez/query.fcgi?cmd=Retrieve&db=pubmed&dopt=Abstract&list_uids=27301070|6|Stem Cells|34|9|Sep|2016|Journal Article
ptgs2	tp53	http://www.ncbi.nlm.nih.gov/entrez/query.fcgi?cmd=Retrieve&db=pubmed&dopt=Abstract&list_uids=27314294|6|Cancer Biomark|17|1|Jun|2016|Journal Article
ptgs2	apc	http://www.ncbi.nlm.nih.gov/entrez/query.fcgi?cmd=Retrieve&db=pubmed&dopt=Abstract&list_uids=27314294|6|Cancer Biomark|17|1|Jun|2016|Journal Article
ptgs2	fos	http://www.ncbi.nlm.nih.gov/entrez/query.fcgi?cmd=Retrieve&db=pubmed&dopt=Abstract&list_uids=27530354|6|J Nat Med|||Aug|2016|JOURNAL ARTICLE
dbt	ptger4	http://www.ncbi.nlm.nih.gov/entrez/query.fcgi?cmd=Retrieve&db=pubmed&dopt=Abstract&list_uids=27301070|6|Stem Cells|34|9|Sep|2016|Journal Article
akt2	ccnd1	http://www.ncbi.nlm.nih.gov/entrez/query.fcgi?cmd=Retrieve&db=pubmed&dopt=Abstract&list_uids=27364926|6|Sci Rep|6|||2016|Journal Article
eed	foxm1	http://www.ncbi.nlm.nih.gov/entrez/query.fcgi?cmd=Retrieve&db=pubmed&dopt=Abstract&list_uids=27303043|6|Proc Natl Acad Sci U S A|113|26|Jun|2016|Journal Article
eed	ezh2	http://www.ncbi.nlm.nih.gov/entrez/query.fcgi?cmd=Retrieve&db=pubmed&dopt=Abstract&list_uids=27303043|6|Proc Natl Acad Sci U S A|113|26|Jun|2016|Journal Article
suz12	foxm1	http://www.ncbi.nlm.nih.gov/entrez/query.fcgi?cmd=Retrieve&db=pubmed&dopt=Abstract&list_uids=27303043|6|Proc Natl Acad Sci U S A|113|26|Jun|2016|Journal Article

suz12	ezh2	http://www.ncbi.nlm.nih.gov/entrez/query.fcgi?cmd=Retrieve&db=pubmed&dopt=Abstract&list_uids=27303043|6|Proc Natl Acad Sci U S A|113|26|Jun|2016|Journal Article
suz12	eed	http://www.ncbi.nlm.nih.gov/entrez/query.fcgi?cmd=Retrieve&db=pubmed&dopt=Abstract&list_uids=27303043|6|Proc Natl Acad Sci U S A|113|26|Jun|2016|Journal Article
vip	egfr	http://www.ncbi.nlm.nih.gov/entrez/query.fcgi?cmd=Retrieve&db=pubmed&dopt=Abstract&list_uids=27303300|6|Front Pharmacol|7|||2016|Journal Article
adcyap1	egfr	http://www.ncbi.nlm.nih.gov/entrez/query.fcgi?cmd=Retrieve&db=pubmed&dopt=Abstract&list_uids=27303300|6|Front Pharmacol|7|||2016|Journal Article
adcyap1	vip	http://www.ncbi.nlm.nih.gov/entrez/query.fcgi?cmd=Retrieve&db=pubmed&dopt=Abstract&list_uids=27303300|6|Front Pharmacol|7|||2016|Journal Article
vdac1	tlr2	http://www.ncbi.nlm.nih.gov/entrez/query.fcgi?cmd=Retrieve&db=pubmed&dopt=Abstract&list_uids=27373686|6|Microbes Infect|||Jul|2016|JOURNAL ARTICLE
atp8a2	vdac1	http://www.ncbi.nlm.nih.gov/entrez/query.fcgi?cmd=Retrieve&db=pubmed&dopt=Abstract&list_uids=27304056|6|Oncotarget|||Jun|2016|JOURNAL ARTICLE
atp8a2	abce1	http://www.ncbi.nlm.nih.gov/entrez/query.fcgi?cmd=Retrieve&db=pubmed&dopt=Abstract&list_uids=27314749|6|Mol Med Rep|14|2|Aug|2016|Journal Article
postn	stat3	http://www.ncbi.nlm.nih.gov/entrez/query.fcgi?cmd=Retrieve&db=pubmed&dopt=Abstract&list_uids=27307601|6|Mol Cancer Ther|15|9|Sep|2016|Journal Article
postn	vegfa	http://www.ncbi.nlm.nih.gov/entrez/query.fcgi?cmd=Retrieve&db=pubmed&dopt=Abstract&list_uids=27307601|6|Mol Cancer Ther|15|9|Sep|2016|Journal Article
postn	itk	http://www.ncbi.nlm.nih.gov/entrez/query.fcgi?cmd=Retrieve&db=pubmed&dopt=Abstract&list_uids=27307601|6|Mol Cancer Ther|15|9|Sep|2016|Journal Article
mirn200b	ldha	http://www.ncbi.nlm.nih.gov/entrez/query.fcgi?cmd=Retrieve&db=pubmed&dopt=Abstract&list_uids=27374173|6|Oncotarget|||Jun|2016|JOURNAL ARTICLE
cdc42	pak7	http://www.ncbi.nlm.nih.gov/entrez/query.fcgi?cmd=Retrieve&db=pubmed&dopt=Abstract&list_uids=27323857|6|Oncotarget|||Jun|2016|JOURNAL ARTICLE
cdc42	akt1	http://www.ncbi.nlm.nih.gov/entrez/query.fcgi?cmd=Retrieve&db=pubmed&dopt=Abstract&list_uids=27323857|6|Oncotarget|||Jun|2016|JOURNAL ARTICLE
cdc42	mmp9	http://www.ncbi.nlm.nih.gov/entrez/query.fcgi?cmd=Retrieve&db=pubmed&dopt=Abstract&list_uids=27367023|6|Oncotarget|||Jun|2016|JOURNAL ARTICLE
cdc42	mapk8	http://www.ncbi.nlm.nih.gov/entrez/query.fcgi?cmd=Retrieve&db=pubmed&dopt=Abstract&list_uids=27367023|6|Oncotarget|||Jun|2016|JOURNAL ARTICLE
cdc42	cd47	http://www.ncbi.nlm.nih.gov/entrez/query.fcgi?cmd=Retrieve&db=pubmed&dopt=Abstract&list_uids=27411490|6|Sci Rep|6|||2016|Journal Article
cdc42	ptk2	http://www.ncbi.nlm.nih.gov/entrez/query.fcgi?cmd=Retrieve&db=pubmed&dopt=Abstract&list_uids=27486972|6|Oncotarget|||Jul|2016|JOURNAL ARTICLE

cdc42	iqgap1	http://www.ncbi.nlm.nih.gov/entrez/query.fcgi?cmd=Retrieve&db=pubmed&dopt=Abstract&list_uids=27486972|6|Oncotarget|||Jul|2016|JOURNAL ARTICLE
arhgap1	cdc42	http://www.ncbi.nlm.nih.gov/entrez/query.fcgi?cmd=Retrieve&db=pubmed&dopt=Abstract&list_uids=27311480|6|J Cell Sci|129|15|Aug|2016|Journal Article
il6	jak2	http://www.ncbi.nlm.nih.gov/entrez/query.fcgi?cmd=Retrieve&db=pubmed&dopt=Abstract&list_uids=27471397|6|Onco Targets Ther|9|||2016|Journal Article
il6	mmp1	http://www.ncbi.nlm.nih.gov/entrez/query.fcgi?cmd=Retrieve&db=pubmed&dopt=Abstract&list_uids=27566200|6|J Ethnopharmacol|||Aug|2016|JOURNAL ARTICLE
il6	hcc	http://www.ncbi.nlm.nih.gov/entrez/query.fcgi?cmd=Retrieve&db=pubmed&dopt=Abstract&list_uids=27608898|6|Eur Rev Med Pharmacol Sci|20|16|Aug|2016|Journal Article
il6	stat3	http://www.ncbi.nlm.nih.gov/entrez/query.fcgi?cmd=Retrieve&db=pubmed&dopt=Abstract&list_uids=27608898|6|Eur Rev Med Pharmacol Sci|20|16|Aug|2016|Journal Article|http://www.ncbi.nlm.nih.gov/entrez/query.fcgi?cmd=Retrieve&db=pubmed&dopt=Abstract&list_uids=27471397|6|Onco Targets Ther|9|||2016|Journal Article|http://www.ncbi.nlm.nih.gov/entrez/query.fcgi?cmd=Retrieve&db=pubmed&dopt=Abstract&list_uids=27384401|6|Sci Rep|6|||2016|Journal Article|http://www.ncbi.nlm.nih.gov/entrez/query.fcgi?cmd=Retrieve&db=pubmed&dopt=Abstract&list_uids=27566200|6|J Ethnopharmacol|||Aug|2016|JOURNAL ARTICLE|http://www.ncbi.nlm.nih.gov/entrez/query.fcgi?cmd=Retrieve&db=pubmed&dopt=Abstract&list_uids=27510094|6|Oncol Rep|||Aug|2016|JOURNAL ARTICLE|http://www.ncbi.nlm.nih.gov/entrez/query.fcgi?cmd=Retrieve&db=pubmed&dopt=Abstract&list_uids=27602757|6|Oncotarget|||Sep|2016|JOURNAL ARTICLE
il6	mmp2	http://www.ncbi.nlm.nih.gov/entrez/query.fcgi?cmd=Retrieve&db=pubmed&dopt=Abstract&list_uids=27613828|6|Oncotarget|||Aug|2016|JOURNAL ARTICLE
il6	mmp14	http://www.ncbi.nlm.nih.gov/entrez/query.fcgi?cmd=Retrieve&db=pubmed&dopt=Abstract&list_uids=27613828|6|Oncotarget|||Aug|2016|JOURNAL ARTICLE
spp1	il8	http://www.ncbi.nlm.nih.gov/entrez/query.fcgi?cmd=Retrieve&db=pubmed&dopt=Abstract&list_uids=27312487|6|Tumour Biol|||Jun|2016|JOURNAL ARTICLE
spp1	il6	http://www.ncbi.nlm.nih.gov/entrez/query.fcgi?cmd=Retrieve&db=pubmed&dopt=Abstract&list_uids=27312487|6|Tumour Biol|||Jun|2016|JOURNAL ARTICLE
spp1	cd34	http://www.ncbi.nlm.nih.gov/entrez/query.fcgi?cmd=Retrieve&db=pubmed&dopt=Abstract&list_uids=27489587|6|Int J Hematol Oncol Stem Cell Res|10|3|Jul|2016|Journal Article
spp1	cd38	http://www.ncbi.nlm.nih.gov/entrez/query.fcgi?cmd=Retrieve&db=pubmed&dopt=Abstract&list_uids=27489587|6|Int J Hematol Oncol Stem Cell Res|10|3|Jul|2016|Journal Article
sirt1	sox2	http://www.ncbi.nlm.nih.gov/entrez/query.fcgi?cmd=Retrieve&db=pubmed&dopt=Abstract&list_uids=27312708|6|Hepatology|64|3|Sep|2016|Journal Article

dicer1	hcc	http://www.ncbi.nlm.nih.gov/entrez/query.fcgi?cmd=Retrieve&db=pubmed&dopt=Abstract&list_uids=27313724|6|Oncol Lett|11|6|Jun|2016|JOURNAL ARTICLE
afp	hcc	http://www.ncbi.nlm.nih.gov/entrez/query.fcgi?cmd=Retrieve&db=pubmed&dopt=Abstract&list_uids=27313784|6|J Cancer|7|8||2016|Journal Article
cxcr4	hcc	http://www.ncbi.nlm.nih.gov/entrez/query.fcgi?cmd=Retrieve&db=pubmed&dopt=Abstract&list_uids=27313784|6|J Cancer|7|8||2016|Journal Article
cxcr4	afp	http://www.ncbi.nlm.nih.gov/entrez/query.fcgi?cmd=Retrieve&db=pubmed&dopt=Abstract&list_uids=27313784|6|J Cancer|7|8||2016|Journal Article
apc	tp53	http://www.ncbi.nlm.nih.gov/entrez/query.fcgi?cmd=Retrieve&db=pubmed&dopt=Abstract&list_uids=27314294|6|Cancer Biomark|17|1|Jun|2016|Journal Article
ifi27	cdkn1b	http://www.ncbi.nlm.nih.gov/entrez/query.fcgi?cmd=Retrieve&db=pubmed&dopt=Abstract&list_uids=27591936|6|Cancer Lett|||Aug|2016|JOURNAL ARTICLE
abce1	ifi27	http://www.ncbi.nlm.nih.gov/entrez/query.fcgi?cmd=Retrieve&db=pubmed&dopt=Abstract&list_uids=27314749|6|Mol Med Rep|14|2|Aug|2016|Journal Article
decr1	ephb2	http://www.ncbi.nlm.nih.gov/entrez/query.fcgi?cmd=Retrieve&db=pubmed&dopt=Abstract&list_uids=27315395|6|Mol Oral Microbiol|||Jun|2016|JOURNAL ARTICLE
cav1	ephb2	http://www.ncbi.nlm.nih.gov/entrez/query.fcgi?cmd=Retrieve&db=pubmed&dopt=Abstract&list_uids=27315395|6|Mol Oral Microbiol|||Jun|2016|JOURNAL ARTICLE
cav1	decr1	http://www.ncbi.nlm.nih.gov/entrez/query.fcgi?cmd=Retrieve&db=pubmed&dopt=Abstract&list_uids=27315395|6|Mol Oral Microbiol|||Jun|2016|JOURNAL ARTICLE
h2afx	zeb1	http://www.ncbi.nlm.nih.gov/entrez/query.fcgi?cmd=Retrieve&db=pubmed&dopt=Abstract&list_uids=27315462|6|Cell Cycle|15|18|Sep|2016|Journal Article
h2afx	itk	http://www.ncbi.nlm.nih.gov/entrez/query.fcgi?cmd=Retrieve&db=pubmed&dopt=Abstract&list_uids=27315462|6|Cell Cycle|15|18|Sep|2016|Journal Article
kras	sparc	http://www.ncbi.nlm.nih.gov/entrez/query.fcgi?cmd=Retrieve&db=pubmed&dopt=Abstract&list_uids=27318148|6|Gastroenterology|151|3|Sep|2016|Journal Article
kras	tp53	http://www.ncbi.nlm.nih.gov/entrez/query.fcgi?cmd=Retrieve&db=pubmed&dopt=Abstract&list_uids=27318148|6|Gastroenterology|151|3|Sep|2016|Journal Article
etv1	sparc	http://www.ncbi.nlm.nih.gov/entrez/query.fcgi?cmd=Retrieve&db=pubmed&dopt=Abstract&list_uids=27318148|6|Gastroenterology|151|3|Sep|2016|Journal Article
etv1	tp53	http://www.ncbi.nlm.nih.gov/entrez/query.fcgi?cmd=Retrieve&db=pubmed&dopt=Abstract&list_uids=27318148|6|Gastroenterology|151|3|Sep|2016|Journal Article
etv1	kras	http://www.ncbi.nlm.nih.gov/entrez/query.fcgi?cmd=Retrieve&db=pubmed&dopt=Abstract&list_uids=27318148|6|Gastroenterology|151|3|Sep|2016|Journal Article
has2	sparc	http://www.ncbi.nlm.nih.gov/entrez/query.fcgi?cmd=Retrieve&db=pubmed&dopt=Abstract&list_uids=27318148|6|Gastroenterology|151|3|Sep|2016|Journal Article

has2	tp53	http://www.ncbi.nlm.nih.gov/entrez/query.fcgi?cmd=Retrieve&db=pubmed&dopt=Abstract&list_uids=27318148|6|Gastroenterology|151|3|Sep|2016|Journal Article
has2	kras	http://www.ncbi.nlm.nih.gov/entrez/query.fcgi?cmd=Retrieve&db=pubmed&dopt=Abstract&list_uids=27318148|6|Gastroenterology|151|3|Sep|2016|Journal Article
has2	etv1	http://www.ncbi.nlm.nih.gov/entrez/query.fcgi?cmd=Retrieve&db=pubmed&dopt=Abstract&list_uids=27318148|6|Gastroenterology|151|3|Sep|2016|Journal Article
pc-3	rac1	http://www.ncbi.nlm.nih.gov/entrez/query.fcgi?cmd=Retrieve&db=pubmed&dopt=Abstract&list_uids=27320871|6|Nan Fang Yi Ke Da Xue Xue Bao|36|6|Jun|2016|English Abstract,Journal Article,Research Support, Non-U.S. Gov't
pc-3	dgcr14	http://www.ncbi.nlm.nih.gov/entrez/query.fcgi?cmd=Retrieve&db=pubmed&dopt=Abstract&list_uids=27446413|6|Oncol Lett|12|2|Aug|2016|JOURNAL ARTICLE
arf6	pc-3	http://www.ncbi.nlm.nih.gov/entrez/query.fcgi?cmd=Retrieve&db=pubmed&dopt=Abstract&list_uids=27320871|6|Nan Fang Yi Ke Da Xue Xue Bao|36|6|Jun|2016|English Abstract,Journal Article,Research Support, Non-U.S. Gov't
snord87	pik3ca	http://www.ncbi.nlm.nih.gov/entrez/query.fcgi?cmd=Retrieve&db=pubmed&dopt=Abstract&list_uids=27394418|6|Neurochem Res|||Jul|2016|JOURNAL ARTICLE
snord87	mirn195	http://www.ncbi.nlm.nih.gov/entrez/query.fcgi?cmd=Retrieve&db=pubmed&dopt=Abstract&list_uids=27574009|6|Oncol Rep|||Aug|2016|JOURNAL ARTICLE
snord87	mirn204	http://www.ncbi.nlm.nih.gov/entrez/query.fcgi?cmd=Retrieve&db=pubmed&dopt=Abstract&list_uids=27588402|6|Oncotarget|||Aug|2016|JOURNAL ARTICLE
psip1	snord87	http://www.ncbi.nlm.nih.gov/entrez/query.fcgi?cmd=Retrieve&db=pubmed&dopt=Abstract&list_uids=27320882|6|Nan Fang Yi Ke Da Xue Xue Bao|36|6|Jun|2016|English Abstract,Journal Article,Research Support, Non-U.S. Gov't
sfrs1	snord87	http://www.ncbi.nlm.nih.gov/entrez/query.fcgi?cmd=Retrieve&db=pubmed&dopt=Abstract&list_uids=27320882|6|Nan Fang Yi Ke Da Xue Xue Bao|36|6|Jun|2016|English Abstract,Journal Article,Research Support, Non-U.S. Gov't
sfrs1	psip1	http://www.ncbi.nlm.nih.gov/entrez/query.fcgi?cmd=Retrieve&db=pubmed&dopt=Abstract&list_uids=27320882|6|Nan Fang Yi Ke Da Xue Xue Bao|36|6|Jun|2016|English Abstract,Journal Article,Research Support, Non-U.S. Gov't
ifrd1	snord87	http://www.ncbi.nlm.nih.gov/entrez/query.fcgi?cmd=Retrieve&db=pubmed&dopt=Abstract&list_uids=27320882|6|Nan Fang Yi Ke Da Xue Xue Bao|36|6|Jun|2016|English Abstract,Journal Article,Research Support, Non-U.S. Gov't
ifrd1	psip1	http://www.ncbi.nlm.nih.gov/entrez/query.fcgi?cmd=Retrieve&db=pubmed&dopt=Abstract&list_uids=27320882|6|Nan Fang Yi Ke Da Xue Xue Bao|36|6|Jun|2016|English Abstract,Journal Article,Research Support, Non-U.S. Gov't
ifrd1	sfrs1	http://www.ncbi.nlm.nih.gov/entrez/query.fcgi?cmd=Retrieve&db=pubmed&dopt=Abstract&list_uids=27320882|6|Nan Fang Yi Ke Da Xue Xue Bao|36|6|Jun|2016|English Abstract,Journal Article,Research Support, Non-U.S. Gov't
il10	hla-e	http://www.ncbi.nlm.nih.gov/entrez/query.fcgi?cmd=Retrieve&db=pubmed&dopt=Abstract&list_uids=27322426|6|Oncotarget|||Jun|2016|JOURNAL ARTICLE
fbxl20	ephb2	http://www.ncbi.nlm.nih.gov/entrez/query.fcgi?cmd=Retrieve&db=pubmed&dopt=Abstract&list_uids=27322511|6|Environ Toxicol|||Jun|2016|JOURNAL ARTICLE
plaur	fpr1	http://www.ncbi.nlm.nih.gov/entrez/query.fcgi?cmd=Retrieve&db=pubmed&dopt=Abstract&list_uids=27323409|6|Oncotarget|||Jun|2016|JOURNAL ARTICLE

plaur	igf1r	http://www.ncbi.nlm.nih.gov/entrez/query.fcgi?cmd=Retrieve&db=pubmed&dopt=Abstract&list_uids=27502396|6|BMC Cancer|16|||2016|Journal Article
plaur	serpine 1	http://www.ncbi.nlm.nih.gov/entrez/query.fcgi?cmd=Retrieve&db=pubmed&dopt=Abstract&list_uids=27502396|6|BMC Cancer|16|||2016|Journal Article
plaur	akt1	http://www.ncbi.nlm.nih.gov/entrez/query.fcgi?cmd=Retrieve&db=pubmed&dopt=Abstract&list_uids=27602956|6|Oncotarget|||Sep|2016|JOURNAL ARTICLE
plaur	pik3ca	http://www.ncbi.nlm.nih.gov/entrez/query.fcgi?cmd=Retrieve&db=pubmed&dopt=Abstract&list_uids=27602956|6|Oncotarget|||Sep|2016|JOURNAL ARTICLE
tug1	xrcc1	http://www.ncbi.nlm.nih.gov/entrez/query.fcgi?cmd=Retrieve&db=pubmed&dopt=Abstract&list_uids=27323757|6|J Mol Histol|47|4|Aug|2016|Journal Article
tes	mapk1 4	http://www.ncbi.nlm.nih.gov/entrez/query.fcgi?cmd=Retrieve&db=pubmed&dopt=Abstract&list_uids=27323777|6|Oncotarget|||Jun|2016|JOURNAL ARTICLE
pak7	itk	http://www.ncbi.nlm.nih.gov/entrez/query.fcgi?cmd=Retrieve&db=pubmed&dopt=Abstract&list_uids=27323857|6|Oncotarget|||Jun|2016|JOURNAL ARTICLE
cdkn1a	pak7	http://www.ncbi.nlm.nih.gov/entrez/query.fcgi?cmd=Retrieve&db=pubmed&dopt=Abstract&list_uids=27323857|6|Oncotarget|||Jun|2016|JOURNAL ARTICLE
cdkn1a	akt1	http://www.ncbi.nlm.nih.gov/entrez/query.fcgi?cmd=Retrieve&db=pubmed&dopt=Abstract&list_uids=27323857|6|Oncotarget|||Jun|2016|JOURNAL ARTICLE
cdkn1a	cdc42	http://www.ncbi.nlm.nih.gov/entrez/query.fcgi?cmd=Retrieve&db=pubmed&dopt=Abstract&list_uids=27323857|6|Oncotarget|||Jun|2016|JOURNAL ARTICLE
cdkn1a	prkch	http://www.ncbi.nlm.nih.gov/entrez/query.fcgi?cmd=Retrieve&db=pubmed&dopt=Abstract&list_uids=27351203|6|PLoS One|11|6||2016|Journal Article
cdkn1a	bax	http://www.ncbi.nlm.nih.gov/entrez/query.fcgi?cmd=Retrieve&db=pubmed&dopt=Abstract&list_uids=27421660|6|Acta Biochim Biophys Sin (Shanghai)|48|8|Aug|2016|Journal Article
cdkn1a	bcl2l1	http://www.ncbi.nlm.nih.gov/entrez/query.fcgi?cmd=Retrieve&db=pubmed&dopt=Abstract&list_uids=27421660|6|Acta Biochim Biophys Sin (Shanghai)|48|8|Aug|2016|Journal Article
cdkn1a	bcl2	http://www.ncbi.nlm.nih.gov/entrez/query.fcgi?cmd=Retrieve&db=pubmed&dopt=Abstract&list_uids=27421660|6|Acta Biochim Biophys Sin (Shanghai)|48|8|Aug|2016|Journal Article
cdkn1a	pak1	http://www.ncbi.nlm.nih.gov/entrez/query.fcgi?cmd=Retrieve&db=pubmed&dopt=Abstract&list_uids=27430434|6|Mol Med Rep|14|3|Sep|2016|Journal Article
cdkn1a	rbp2	http://www.ncbi.nlm.nih.gov/entrez/query.fcgi?cmd=Retrieve&db=pubmed&dopt=Abstract&list_uids=27548814|6|Oncogenesis|5|8||2016|Journal Article
cdkn1a	cdkn1c	http://www.ncbi.nlm.nih.gov/entrez/query.fcgi?cmd=Retrieve&db=pubmed&dopt=Abstract&list_uids=27548814|6|Oncogenesis|5|8||2016|Journal Article
cdkn1a	ifi27	http://www.ncbi.nlm.nih.gov/entrez/query.fcgi?cmd=Retrieve&db=pubmed&dopt=Abstract&list_uids=27377307|6|Sci Rep|6|||2016|Journal

		Article|http://www.ncbi.nlm.nih.gov/entrez/query.fcgi?cmd=Retrieve&db=pubmed&dopt=Abstract&list_uids=27569589|6|Life Sci|||Aug|2016|JOURNAL ARTICLE
il8rb	mmp9	http://www.ncbi.nlm.nih.gov/entrez/query.fcgi?cmd=Retrieve&db=pubmed&dopt=Abstract&list_uids=27324095|6|Placenta|43||Jul|2016|Journal Article
il8rb	mmp2	http://www.ncbi.nlm.nih.gov/entrez/query.fcgi?cmd=Retrieve&db=pubmed&dopt=Abstract&list_uids=27324095|6|Placenta|43||Jul|2016|Journal Article
il8rb	akt1	http://www.ncbi.nlm.nih.gov/entrez/query.fcgi?cmd=Retrieve&db=pubmed&dopt=Abstract&list_uids=27324095|6|Placenta|43||Jul|2016|Journal Article
il8rb	msc	http://www.ncbi.nlm.nih.gov/entrez/query.fcgi?cmd=Retrieve&db=pubmed&dopt=Abstract&list_uids=27374099|6|Oncotarget|||Jun|2016|JOURNAL ARTICLE
hdac1	bcl9	http://www.ncbi.nlm.nih.gov/entrez/query.fcgi?cmd=Retrieve&db=pubmed&dopt=Abstract&list_uids=27324116|6|Cancer Sci|||Jun|2016|JOURNAL ARTICLE
hdac1	tmprss11d	http://www.ncbi.nlm.nih.gov/entrez/query.fcgi?cmd=Retrieve&db=pubmed&dopt=Abstract&list_uids=27429844|6|Am J Cancer Res|6|6||2016|Journal Article
hdac1	hdac8	http://www.ncbi.nlm.nih.gov/entrez/query.fcgi?cmd=Retrieve&db=pubmed&dopt=Abstract&list_uids=27429844|6|Am J Cancer Res|6|6||2016|Journal Article
hdac1	hdac3	http://www.ncbi.nlm.nih.gov/entrez/query.fcgi?cmd=Retrieve&db=pubmed&dopt=Abstract&list_uids=27429844|6|Am J Cancer Res|6|6||2016|Journal Article
hdac1	hdac2	http://www.ncbi.nlm.nih.gov/entrez/query.fcgi?cmd=Retrieve&db=pubmed&dopt=Abstract&list_uids=27429844|6|Am J Cancer Res|6|6||2016|Journal Article
1.4.1.20	bcl9	http://www.ncbi.nlm.nih.gov/entrez/query.fcgi?cmd=Retrieve&db=pubmed&dopt=Abstract&list_uids=27324116|6|Cancer Sci|||Jun|2016|JOURNAL ARTICLE
1.4.1.20	hdac1	http://www.ncbi.nlm.nih.gov/entrez/query.fcgi?cmd=Retrieve&db=pubmed&dopt=Abstract&list_uids=27324116|6|Cancer Sci|||Jun|2016|JOURNAL ARTICLE
1.4.1.20	pygo2	http://www.ncbi.nlm.nih.gov/entrez/query.fcgi?cmd=Retrieve&db=pubmed&dopt=Abstract&list_uids=27324116|6|Cancer Sci|||Jun|2016|JOURNAL ARTICLE
pygo2	bcl9	http://www.ncbi.nlm.nih.gov/entrez/query.fcgi?cmd=Retrieve&db=pubmed&dopt=Abstract&list_uids=27324116|6|Cancer Sci|||Jun|2016|JOURNAL ARTICLE
pygo2	hdac1	http://www.ncbi.nlm.nih.gov/entrez/query.fcgi?cmd=Retrieve&db=pubmed&dopt=Abstract&list_uids=27324116|6|Cancer Sci|||Jun|2016|JOURNAL ARTICLE
lats1	yap1	http://www.ncbi.nlm.nih.gov/entrez/query.fcgi?cmd=Retrieve&db=pubmed&dopt=Abstract&list_uids=27325643|6|Cancer Res|76|13|Jul|2016|Journal Article
ctbs	hla-g	http://www.ncbi.nlm.nih.gov/entrez/query.fcgi?cmd=Retrieve&db=pubmed&dopt=Abstract&list_uids=27325764|6|Proc Natl Acad Sci U S A|113|27|Jul|2016|Journal Article
cd8a	rln2	http://www.ncbi.nlm.nih.gov/entrez/query.fcgi?cmd=Retrieve&db=pubmed&dopt=Abstract&list_uids=27467944|6|Oncoimmunology|5|5|May|2016|Journal Article

cd4	cd8a	http://www.ncbi.nlm.nih.gov/entrez/query.fcgi?cmd=Retrieve&db=pubmed&dopt=Abstract&list_uids=27328524|6|Roum Arch Microbiol Immunol|74|3-4||2015|Journal Article,Research Support, Non-U.S. Gov't,Review
mirn19a	rhob	http://www.ncbi.nlm.nih.gov/entrez/query.fcgi?cmd=Retrieve&db=pubmed&dopt=Abstract&list_uids=27329239|6|Neurosci Lett|628||Aug|2016|Journal Article
cd276	mirn187	http://www.ncbi.nlm.nih.gov/entrez/query.fcgi?cmd=Retrieve&db=pubmed&dopt=Abstract&list_uids=27329595|6|Oncotarget|||Jun|2016|JOURNAL ARTICLE
slc16a4	cma1	http://www.ncbi.nlm.nih.gov/entrez/query.fcgi?cmd=Retrieve&db=pubmed&dopt=Abstract&list_uids=27331625|6|Oncotarget|||Jun|2016|JOURNAL ARTICLE
cma1	cltc	http://www.ncbi.nlm.nih.gov/entrez/query.fcgi?cmd=Retrieve&db=pubmed&dopt=Abstract&list_uids=27331625|6|Oncotarget|||Jun|2016|JOURNAL ARTICLE
cr1	tnfsf13	http://www.ncbi.nlm.nih.gov/entrez/query.fcgi?cmd=Retrieve&db=pubmed&dopt=Abstract&list_uids=27333049|6|EBioMedicine|9||Jul|2016|Journal Article
lamc2	cdx2	http://www.ncbi.nlm.nih.gov/entrez/query.fcgi?cmd=Retrieve&db=pubmed&dopt=Abstract&list_uids=27333824|6|J Cell Biochem|||Jun|2016|JOURNAL ARTICLE
lbx1	cdx2	http://www.ncbi.nlm.nih.gov/entrez/query.fcgi?cmd=Retrieve&db=pubmed&dopt=Abstract&list_uids=27333824|6|J Cell Biochem|||Jun|2016|JOURNAL ARTICLE
lbx1	zeb2	http://www.ncbi.nlm.nih.gov/entrez/query.fcgi?cmd=Retrieve&db=pubmed&dopt=Abstract&list_uids=27347142|6|Oncol Lett|12|1|Jul|2016|JOURNAL ARTICLE
lbx1	itk	http://www.ncbi.nlm.nih.gov/entrez/query.fcgi?cmd=Retrieve&db=pubmed&dopt=Abstract&list_uids=27500490|6|J Clin Invest|126|9|Sep|2016|Journal Article
lbx1	zeb1	http://www.ncbi.nlm.nih.gov/entrez/query.fcgi?cmd=Retrieve&db=pubmed&dopt=Abstract&list_uids=27500490|6|J Clin Invest|126|9|Sep|2016|Journal Article
ccr5	atp8a2	http://www.ncbi.nlm.nih.gov/entrez/query.fcgi?cmd=Retrieve&db=pubmed&dopt=Abstract&list_uids=27335323|6|Open Biol|6|6|Jun|2016|Journal Article
ccl5	atp8a2	http://www.ncbi.nlm.nih.gov/entrez/query.fcgi?cmd=Retrieve&db=pubmed&dopt=Abstract&list_uids=27335323|6|Open Biol|6|6|Jun|2016|Journal Article
ccl5	frap1	http://www.ncbi.nlm.nih.gov/entrez/query.fcgi?cmd=Retrieve&db=pubmed&dopt=Abstract&list_uids=27335323|6|Open Biol|6|6|Jun|2016|Journal Article
ccl5	ccr5	http://www.ncbi.nlm.nih.gov/entrez/query.fcgi?cmd=Retrieve&db=pubmed&dopt=Abstract&list_uids=27335323|6|Open Biol|6|6|Jun|2016|Journal Article
nupr1	hcc	http://www.ncbi.nlm.nih.gov/entrez/query.fcgi?cmd=Retrieve&db=pubmed&dopt=Abstract&list_uids=27336713|6|Cell Death Dis|7|6||2016|Journal Article
runx2	hcc	http://www.ncbi.nlm.nih.gov/entrez/query.fcgi?cmd=Retrieve&db=pubmed&dopt=Abstract&list_uids=27336713|6|Cell Death Dis|7|6||2016|Journal Article
runx2	nupr1	http://www.ncbi.nlm.nih.gov/entrez/query.fcgi?cmd=Retrieve&db=pubmed&dopt=Abstract&list_uids=27336713|6|Cell Death Dis|7|6||2016|Journal Article

ier3	hcc	http://www.ncbi.nlm.nih.gov/entrez/query.fcgi?cmd=Retrieve&db=pubmed&dopt=Abstract&list_uids=27336713|6|Cell Death Dis|7|6||2016|Journal Article
relb	hcc	http://www.ncbi.nlm.nih.gov/entrez/query.fcgi?cmd=Retrieve&db=pubmed&dopt=Abstract&list_uids=27336713|6|Cell Death Dis|7|6||2016|Journal Article
relb	ier3	http://www.ncbi.nlm.nih.gov/entrez/query.fcgi?cmd=Retrieve&db=pubmed&dopt=Abstract&list_uids=27336713|6|Cell Death Dis|7|6||2016|Journal Article
pten	bax	http://www.ncbi.nlm.nih.gov/entrez/query.fcgi?cmd=Retrieve&db=pubmed&dopt=Abstract&list_uids=27421660|6|Acta Biochim Biophys Sin (Shanghai)|48|8|Aug|2016|Journal Article
pten	bcl2l1	http://www.ncbi.nlm.nih.gov/entrez/query.fcgi?cmd=Retrieve&db=pubmed&dopt=Abstract&list_uids=27421660|6|Acta Biochim Biophys Sin (Shanghai)|48|8|Aug|2016|Journal Article
pten	bcl2	http://www.ncbi.nlm.nih.gov/entrez/query.fcgi?cmd=Retrieve&db=pubmed&dopt=Abstract&list_uids=27421660|6|Acta Biochim Biophys Sin (Shanghai)|48|8|Aug|2016|Journal Article
pten	cdkn1a	http://www.ncbi.nlm.nih.gov/entrez/query.fcgi?cmd=Retrieve&db=pubmed&dopt=Abstract&list_uids=27421660|6|Acta Biochim Biophys Sin (Shanghai)|48|8|Aug|2016|Journal Article
pten	tp53	http://www.ncbi.nlm.nih.gov/entrez/query.fcgi?cmd=Retrieve&db=pubmed&dopt=Abstract&list_uids=27421660|6|Acta Biochim Biophys Sin (Shanghai)|48|8|Aug|2016|Journal Article|http://www.ncbi.nlm.nih.gov/entrez/query.fcgi?cmd=Retrieve&db=pubmed&dopt=Abstract&list_uids=27499479|6|Int J Oncol|||Aug|2016|JOURNAL ARTICLE
pten	frap1	http://www.ncbi.nlm.nih.gov/entrez/query.fcgi?cmd=Retrieve&db=pubmed&dopt=Abstract&list_uids=27499479|6|Int J Oncol|||Aug|2016|JOURNAL ARTICLE
pten	mdm2	http://www.ncbi.nlm.nih.gov/entrez/query.fcgi?cmd=Retrieve&db=pubmed&dopt=Abstract&list_uids=27499479|6|Int J Oncol|||Aug|2016|JOURNAL ARTICLE
pten	rela	http://www.ncbi.nlm.nih.gov/entrez/query.fcgi?cmd=Retrieve&db=pubmed&dopt=Abstract&list_uids=27533779|6|Mol Carcinog|||Aug|2016|JOURNAL ARTICLE
pten	eif2ak3	http://www.ncbi.nlm.nih.gov/entrez/query.fcgi?cmd=Retrieve&db=pubmed&dopt=Abstract&list_uids=27533779|6|Mol Carcinog|||Aug|2016|JOURNAL ARTICLE
pten	ephb2	http://www.ncbi.nlm.nih.gov/entrez/query.fcgi?cmd=Retrieve&db=pubmed&dopt=Abstract&list_uids=27533779|6|Mol Carcinog|||Aug|2016|JOURNAL ARTICLE
pten	akt1	http://www.ncbi.nlm.nih.gov/entrez/query.fcgi?cmd=Retrieve&db=pubmed&dopt=Abstract&list_uids=27600360|6|Cell Biol Int|||Sep|2016|JOURNAL ARTICLE|http://www.ncbi.nlm.nih.gov/entrez/query.fcgi?cmd=Retrieve&db=pubmed&dopt=Abstract&list_uids=27446408|6|Oncol Lett|12|2|Aug|2016|JOURNAL ARTICLE|http://www.ncbi.nlm.nih.gov/entrez/query.fcgi?cmd=Retrieve&db=pubmed&dopt=Abstract&list_uids=27499479|6|Int J Oncol|||Aug|2016|JOURNAL ARTICLE|http://www.ncbi.nlm.nih.gov/entrez/query.fcgi?cmd=Retrieve&db=pubmed&dopt=Abstract&list_uids=27383307|6|Eur Rev Med Pharmacol Sci|20|12|Jun|2016|Journal Article

pten	pik3ca	http://www.ncbi.nlm.nih.gov/entrez/query.fcgi?cmd=Retrieve&db=pubmed&dopt=Abstract&list_uids=27600360|6|Cell Biol Int|||Sep|2016|JOURNAL ARTICLE|http://www.ncbi.nlm.nih.gov/entrez/query.fcgi?cmd=Retrieve&db=pubmed&dopt=Abstract&list_uids=27446408|6|Oncol Lett|12|2|Aug|2016|JOURNAL ARTICLE|http://www.ncbi.nlm.nih.gov/entrez/query.fcgi?cmd=Retrieve&db=pubmed&dopt=Abstract&list_uids=27383307|6|Eur Rev Med Pharmacol Sci|20|12|Jun|2016|Journal Article
mirn214	pten	http://www.ncbi.nlm.nih.gov/entrez/query.fcgi?cmd=Retrieve&db=pubmed&dopt=Abstract&list_uids=27339596|6|Clin Res Hepatol Gastroenterol|||Jun|2016|JOURNAL ARTICLE
eif3h	hcc	http://www.ncbi.nlm.nih.gov/entrez/query.fcgi?cmd=Retrieve&db=pubmed&dopt=Abstract&list_uids=27340783|6|Oncotarget|||Jun|2016|JOURNAL ARTICLE
pou5f1	jun	http://www.ncbi.nlm.nih.gov/entrez/query.fcgi?cmd=Retrieve&db=pubmed&dopt=Abstract&list_uids=27341307|6|Stem Cells|||Jun|2016|JOURNAL ARTICLE
pou5f1	stat3	http://www.ncbi.nlm.nih.gov/entrez/query.fcgi?cmd=Retrieve&db=pubmed&dopt=Abstract&list_uids=27472461|6|Oncotarget|||Jul|2016|JOURNAL ARTICLE
pou5f1	sox2	http://www.ncbi.nlm.nih.gov/entrez/query.fcgi?cmd=Retrieve&db=pubmed&dopt=Abstract&list_uids=27472461|6|Oncotarget|||Jul|2016|JOURNAL ARTICLE
vcp	stat3	http://www.ncbi.nlm.nih.gov/entrez/query.fcgi?cmd=Retrieve&db=pubmed&dopt=Abstract&list_uids=27344168|6|Mol Cell Biochem|418|1-2|Jul|2016|Journal Article
gabpa	nqo1	http://www.ncbi.nlm.nih.gov/entrez/query.fcgi?cmd=Retrieve&db=pubmed&dopt=Abstract&list_uids=27459794|6|Wei Sheng Yan Jiu|45|3|May|2016|English Abstract,Journal Article
gabpa	hbe1	http://www.ncbi.nlm.nih.gov/entrez/query.fcgi?cmd=Retrieve&db=pubmed&dopt=Abstract&list_uids=27459794|6|Wei Sheng Yan Jiu|45|3|May|2016|English Abstract,Journal Article
keap1	gabpa	http://www.ncbi.nlm.nih.gov/entrez/query.fcgi?cmd=Retrieve&db=pubmed&dopt=Abstract&list_uids=27346396|6|Zhonghua Zhong Liu Za Zhi|38|6|Jun|2016|English Abstract,Journal Article
cct4	gabpa	http://www.ncbi.nlm.nih.gov/entrez/query.fcgi?cmd=Retrieve&db=pubmed&dopt=Abstract&list_uids=27346396|6|Zhonghua Zhong Liu Za Zhi|38|6|Jun|2016|English Abstract,Journal Article
cct4	keap1	http://www.ncbi.nlm.nih.gov/entrez/query.fcgi?cmd=Retrieve&db=pubmed&dopt=Abstract&list_uids=27346396|6|Zhonghua Zhong Liu Za Zhi|38|6|Jun|2016|English Abstract,Journal Article
sp1	slc2a3	http://www.ncbi.nlm.nih.gov/entrez/query.fcgi?cmd=Retrieve&db=pubmed&dopt=Abstract&list_uids=27347112|6|Oncol Lett|12|1|Jul|2016|JOURNAL ARTICLE
sp1	mmp9	http://www.ncbi.nlm.nih.gov/entrez/query.fcgi?cmd=Retrieve&db=pubmed&dopt=Abstract&list_uids=27573859|6|Oncol Rep|||Aug|2016|JOURNAL ARTICLE
ogt	mgea5	http://www.ncbi.nlm.nih.gov/entrez/query.fcgi?cmd=Retrieve&db=pubmed&dopt=Abstract&list_uids=27347182|6|Oncol Lett|12|1|Jul|2016|JOURNAL ARTICLE
ogt	ephb2	http://www.ncbi.nlm.nih.gov/entrez/query.fcgi?cmd=Retrieve&db=pubmed&dopt=Abstract&list_uids=27542217|6|Oncotarget|||Aug|2016|JOURNAL ARTICLE

ogt	cdk2	http://www.ncbi.nlm.nih.gov/entrez/query.fcgi?cmd=Retrieve&db=pubmed&dopt=Abstract&list_uids=27542217|6|Oncotarget|||Aug|2016|JOURNAL ARTICLE
mirn26b	mirn26a1	http://www.ncbi.nlm.nih.gov/entrez/query.fcgi?cmd=Retrieve&db=pubmed&dopt=Abstract&list_uids=27347306|6|Am J Transl Res|8|5||2016|Journal Article
traf5	mirn26a1	http://www.ncbi.nlm.nih.gov/entrez/query.fcgi?cmd=Retrieve&db=pubmed&dopt=Abstract&list_uids=27347306|6|Am J Transl Res|8|5||2016|Journal Article
traf5	mirn26b	http://www.ncbi.nlm.nih.gov/entrez/query.fcgi?cmd=Retrieve&db=pubmed&dopt=Abstract&list_uids=27347306|6|Am J Transl Res|8|5||2016|Journal Article
atm	smad2	http://www.ncbi.nlm.nih.gov/entrez/query.fcgi?cmd=Retrieve&db=pubmed&dopt=Abstract&list_uids=27347327|6|Am J Transl Res|8|5||2016|Journal Article
s100a4	mmp2	http://www.ncbi.nlm.nih.gov/entrez/query.fcgi?cmd=Retrieve&db=pubmed&dopt=Abstract&list_uids=27347327|6|Am J Transl Res|8|5||2016|Journal Article
rassf10	mmp2	http://www.ncbi.nlm.nih.gov/entrez/query.fcgi?cmd=Retrieve&db=pubmed&dopt=Abstract&list_uids=27348267|6|Oncogenesis|5|6||2016|Journal Article
rassf10	mapk14	http://www.ncbi.nlm.nih.gov/entrez/query.fcgi?cmd=Retrieve&db=pubmed&dopt=Abstract&list_uids=27348267|6|Oncogenesis|5|6||2016|Journal Article
rassf10	hcc	http://www.ncbi.nlm.nih.gov/entrez/query.fcgi?cmd=Retrieve&db=pubmed&dopt=Abstract&list_uids=27348267|6|Oncogenesis|5|6||2016|Journal Article
rrm2	akt1	http://www.ncbi.nlm.nih.gov/entrez/query.fcgi?cmd=Retrieve&db=pubmed&dopt=Abstract&list_uids=27348973|6|Pharmazie|71|5|May|2016|Journal Article,Research Support, Non-U.S. Gov't
commd7	mmp2	http://www.ncbi.nlm.nih.gov/entrez/query.fcgi?cmd=Retrieve&db=pubmed&dopt=Abstract&list_uids=27350032|6|Mol Carcinog|||Jun|2016|JOURNAL ARTICLE
commd7	bax	http://www.ncbi.nlm.nih.gov/entrez/query.fcgi?cmd=Retrieve&db=pubmed&dopt=Abstract&list_uids=27350032|6|Mol Carcinog|||Jun|2016|JOURNAL ARTICLE
commd7	bcl2	http://www.ncbi.nlm.nih.gov/entrez/query.fcgi?cmd=Retrieve&db=pubmed&dopt=Abstract&list_uids=27350032|6|Mol Carcinog|||Jun|2016|JOURNAL ARTICLE
bax	bid	http://www.ncbi.nlm.nih.gov/entrez/query.fcgi?cmd=Retrieve&db=pubmed&dopt=Abstract&list_uids=27366113|6|Cancer Cell Int|16|||2016|Journal Article
bax	eya1	http://www.ncbi.nlm.nih.gov/entrez/query.fcgi?cmd=Retrieve&db=pubmed&dopt=Abstract&list_uids=27531778|6|Zhongguo Shi Yan Xue Ye Xue Za Zhi|24|4|Aug|2016|English Abstract,Journal Article
bax	parp1	http://www.ncbi.nlm.nih.gov/entrez/query.fcgi?cmd=Retrieve&db=pubmed&dopt=Abstract&list_uids=27580587|6|Biofactors|||Sep|2016|JOURNAL ARTICLE
bax	bcl2	http://www.ncbi.nlm.nih.gov/entrez/query.fcgi?cmd=Retrieve&db=pubmed&dopt=Abstract&list_uids=27429843|6|Am J Cancer Res|6|6||2016|Journal Article|http://www.ncbi.nlm.nih.gov/entrez/query.fcgi?cmd=Retrieve&db=pubmed&dopt=Abstract&list_uids=27600360|6|Cell Biol Int|||Sep|2016|JOURNAL

		ARTICLE|http://www.ncbi.nlm.nih.gov/entrez/query.fcgi?cmd=Retrieve&db=pubmed&dopt=Abstract&list_uids=27565846|6|Biomed Pharmacother|83||Aug|2016|JOURNAL ARTICLE|http://www.ncbi.nlm.nih.gov/entrez/query.fcgi?cmd=Retrieve&db=pubmed&dopt=Abstract&list_uids=27366113|6|Cancer Cell Int|16|||2016|Journal Article|http://www.ncbi.nlm.nih.gov/entrez/query.fcgi?cmd=Retrieve&db=pubmed&dopt=Abstract&list_uids=27350032|6|Mol Carcinog|||Jun|2016|JOURNAL ARTICLE|http://www.ncbi.nlm.nih.gov/entrez/query.fcgi?cmd=Retrieve&db=pubmed&dopt=Abstract&list_uids=27421660|6|Acta Biochim Biophys Sin (Shanghai)|48|8|Aug|2016|Journal Article|http://www.ncbi.nlm.nih.gov/entrez/query.fcgi?cmd=Retrieve&db=pubmed&dopt=Abstract&list_uids=27531778|6|Zhongguo Shi Yan Xue Ye Xue Za Zhi|24|4|Aug|2016|English Abstract,Journal Article|http://www.ncbi.nlm.nih.gov/entrez/query.fcgi?cmd=Retrieve&db=pubmed&dopt=Abstract&list_uids=27470369|6|Biomed Pharmacother|82||Aug|2016|Journal Article|http://www.ncbi.nlm.nih.gov/entrez/query.fcgi?cmd=Retrieve&db=pubmed&dopt=Abstract&list_uids=27470358|6|Biomed Pharmacother|82||Aug|2016|Journal Article|http://www.ncbi.nlm.nih.gov/entrez/query.fcgi?cmd=Retrieve&db=pubmed&dopt=Abstract&list_uids=27580587|6|Biofactors|||Sep|2016|JOURNAL ARTICLE
bcl2	bid	http://www.ncbi.nlm.nih.gov/entrez/query.fcgi?cmd=Retrieve&db=pubmed&dopt=Abstract&list_uids=27366113|6|Cancer Cell Int|16|||2016|Journal Article
bcl2	vegfa	http://www.ncbi.nlm.nih.gov/entrez/query.fcgi?cmd=Retrieve&db=pubmed&dopt=Abstract&list_uids=27415751|6|Cancer Biol Ther|||Jul|2016|JOURNAL ARTICLE
bcl2	bcl2l1	http://www.ncbi.nlm.nih.gov/entrez/query.fcgi?cmd=Retrieve&db=pubmed&dopt=Abstract&list_uids=27415751|6|Cancer Biol Ther|||Jul|2016|JOURNAL ARTICLE|http://www.ncbi.nlm.nih.gov/entrez/query.fcgi?cmd=Retrieve&db=pubmed&dopt=Abstract&list_uids=27421660|6|Acta Biochim Biophys Sin (Shanghai)|48|8|Aug|2016|Journal Article
bcl2	mmp9	http://www.ncbi.nlm.nih.gov/entrez/query.fcgi?cmd=Retrieve&db=pubmed&dopt=Abstract&list_uids=27470348|6|Biomed Pharmacother|82||Aug|2016|Journal Article
bcl2	mmp2	http://www.ncbi.nlm.nih.gov/entrez/query.fcgi?cmd=Retrieve&db=pubmed&dopt=Abstract&list_uids=27470348|6|Biomed Pharmacother|82||Aug|2016|Journal Article
bcl2	pcna	http://www.ncbi.nlm.nih.gov/entrez/query.fcgi?cmd=Retrieve&db=pubmed&dopt=Abstract&list_uids=27470348|6|Biomed Pharmacother|82||Aug|2016|Journal Article
bcl2	parp1	http://www.ncbi.nlm.nih.gov/entrez/query.fcgi?cmd=Retrieve&db=pubmed&dopt=Abstract&list_uids=27580587|6|Biofactors|||Sep|2016|JOURNAL ARTICLE
prkch	uts2r	http://www.ncbi.nlm.nih.gov/entrez/query.fcgi?cmd=Retrieve&db=pubmed&dopt=Abstract&list_uids=27351203|6|PLoS One|11|6||2016|Journal Article
ednra	uts2r	http://www.ncbi.nlm.nih.gov/entrez/query.fcgi?cmd=Retrieve&db=pubmed&dopt=Abstract&list_uids=27351203|6|PLoS One|11|6||2016|Journal Article

ednra	prkch	http://www.ncbi.nlm.nih.gov/entrez/query.fcgi?cmd=Retrieve&db=pubmed&dopt=Abstract&list_uids=27351203|6|PLoS One|11|6||2016|Journal Article
il17a	stat3	http://www.ncbi.nlm.nih.gov/entrez/query.fcgi?cmd=Retrieve&db=pubmed&dopt=Abstract&list_uids=27353107|6|Zhonghua Wei Chang Wai Ke Za Zhi|19|6|Jun|2016|English Abstract,Journal Article
hspb1	hcc	http://www.ncbi.nlm.nih.gov/entrez/query.fcgi?cmd=Retrieve&db=pubmed&dopt=Abstract&list_uids=27354654|6|Anticancer Res|36|7|Jul|2016|Journal Article
ccr4	mmp13	http://www.ncbi.nlm.nih.gov/entrez/query.fcgi?cmd=Retrieve&db=pubmed&dopt=Abstract&list_uids=27356745|6|Oncotarget|||Jun|2016|JOURNAL ARTICLE
ccr4	ephb2	http://www.ncbi.nlm.nih.gov/entrez/query.fcgi?cmd=Retrieve&db=pubmed&dopt=Abstract&list_uids=27356745|6|Oncotarget|||Jun|2016|JOURNAL ARTICLE
opcml	jag1	http://www.ncbi.nlm.nih.gov/entrez/query.fcgi?cmd=Retrieve&db=pubmed&dopt=Abstract&list_uids=27358143|6|J Biol Regul Homeost Agents|30|2||2016|Journal Article
adamts5	timp3	http://www.ncbi.nlm.nih.gov/entrez/query.fcgi?cmd=Retrieve&db=pubmed&dopt=Abstract&list_uids=27359117|6|Pathobiology|83|6||2016|Journal Article
fut1	cxcr4	http://www.ncbi.nlm.nih.gov/entrez/query.fcgi?cmd=Retrieve&db=pubmed&dopt=Abstract&list_uids=27362846|6|PLoS One|11|6||2016|Journal Article
abl2	vegfa	http://www.ncbi.nlm.nih.gov/entrez/query.fcgi?cmd=Retrieve&db=pubmed&dopt=Abstract&list_uids=27363017|6|Oncotarget|||May|2016|JOURNAL ARTICLE
abl2	mmp2	http://www.ncbi.nlm.nih.gov/entrez/query.fcgi?cmd=Retrieve&db=pubmed&dopt=Abstract&list_uids=27363017|6|Oncotarget|||May|2016|JOURNAL ARTICLE
arg2	vegfa	http://www.ncbi.nlm.nih.gov/entrez/query.fcgi?cmd=Retrieve&db=pubmed&dopt=Abstract&list_uids=27363017|6|Oncotarget|||May|2016|JOURNAL ARTICLE
arg2	abl2	http://www.ncbi.nlm.nih.gov/entrez/query.fcgi?cmd=Retrieve&db=pubmed&dopt=Abstract&list_uids=27363017|6|Oncotarget|||May|2016|JOURNAL ARTICLE
arg2	mmp2	http://www.ncbi.nlm.nih.gov/entrez/query.fcgi?cmd=Retrieve&db=pubmed&dopt=Abstract&list_uids=27363017|6|Oncotarget|||May|2016|JOURNAL ARTICLE
mmp11	rab27a	http://www.ncbi.nlm.nih.gov/entrez/query.fcgi?cmd=Retrieve&db=pubmed&dopt=Abstract&list_uids=27364572|6|Cancer Gene Ther|23|8|Aug|2016|Journal Article
mirn145	rab27a	http://www.ncbi.nlm.nih.gov/entrez/query.fcgi?cmd=Retrieve&db=pubmed&dopt=Abstract&list_uids=27364572|6|Cancer Gene Ther|23|8|Aug|2016|Journal Article
mirn145	mmp11	http://www.ncbi.nlm.nih.gov/entrez/query.fcgi?cmd=Retrieve&db=pubmed&dopt=Abstract&list_uids=27364572|6|Cancer Gene Ther|23|8|Aug|2016|Journal Article
mirn145	muc13	http://www.ncbi.nlm.nih.gov/entrez/query.fcgi?cmd=Retrieve&db=pubmed&dopt=Abstract&list_uids=27507554|6|J Gastrointest Surg|||Aug|2016|JOURNAL ARTICLE
mirn145	muc3a	http://www.ncbi.nlm.nih.gov/entrez/query.fcgi?cmd=Retrieve&db=pubmed&dopt=Abstract&list_uids=27507554|6|J Gastrointest Surg|||Aug|2016|JOURNAL ARTICLE

dnmbp	ros1	http://www.ncbi.nlm.nih.gov/entrez/query.fcgi?cmd=Retrieve&db=pubmed&dopt=Abstract&list_uids=27366113|6|Cancer Cell Int|16|||2016|Journal Article
tpt1	mmp9	http://www.ncbi.nlm.nih.gov/entrez/query.fcgi?cmd=Retrieve&db=pubmed&dopt=Abstract&list_uids=27367023|6|Oncotarget|||Jun|2016|JOURNAL ARTICLE
tpt1	mapk8	http://www.ncbi.nlm.nih.gov/entrez/query.fcgi?cmd=Retrieve&db=pubmed&dopt=Abstract&list_uids=27367023|6|Oncotarget|||Jun|2016|JOURNAL ARTICLE
tpt1	cdc42	http://www.ncbi.nlm.nih.gov/entrez/query.fcgi?cmd=Retrieve&db=pubmed&dopt=Abstract&list_uids=27367023|6|Oncotarget|||Jun|2016|JOURNAL ARTICLE
scaf1	stat6	http://www.ncbi.nlm.nih.gov/entrez/query.fcgi?cmd=Retrieve&db=pubmed&dopt=Abstract&list_uids=27367025|6|Oncotarget|||Jun|2016|JOURNAL ARTICLE
scaf1	stat3	http://www.ncbi.nlm.nih.gov/entrez/query.fcgi?cmd=Retrieve&db=pubmed&dopt=Abstract&list_uids=27367025|6|Oncotarget|||Jun|2016|JOURNAL ARTICLE
scaf1	tam	http://www.ncbi.nlm.nih.gov/entrez/query.fcgi?cmd=Retrieve&db=pubmed&dopt=Abstract&list_uids=27367025|6|Oncotarget|||Jun|2016|JOURNAL ARTICLE
hspa4	scaf1	http://www.ncbi.nlm.nih.gov/entrez/query.fcgi?cmd=Retrieve&db=pubmed&dopt=Abstract&list_uids=27367025|6|Oncotarget|||Jun|2016|JOURNAL ARTICLE
hspa4	tnfsf13	http://www.ncbi.nlm.nih.gov/entrez/query.fcgi?cmd=Retrieve&db=pubmed&dopt=Abstract&list_uids=27367025|6|Oncotarget|||Jun|2016|JOURNAL ARTICLE
stat6	zeb1	http://www.ncbi.nlm.nih.gov/entrez/query.fcgi?cmd=Retrieve&db=pubmed&dopt=Abstract&list_uids=27533463|6|Oncotarget|||Aug|2016|JOURNAL ARTICLE
stat6	itk	http://www.ncbi.nlm.nih.gov/entrez/query.fcgi?cmd=Retrieve&db=pubmed&dopt=Abstract&list_uids=27533463|6|Oncotarget|||Aug|2016|JOURNAL ARTICLE
stat6	il13	http://www.ncbi.nlm.nih.gov/entrez/query.fcgi?cmd=Retrieve&db=pubmed&dopt=Abstract&list_uids=27533463|6|Oncotarget|||Aug|2016|JOURNAL ARTICLE
tam	stat6	http://www.ncbi.nlm.nih.gov/entrez/query.fcgi?cmd=Retrieve&db=pubmed&dopt=Abstract&list_uids=27367025|6|Oncotarget|||Jun|2016|JOURNAL ARTICLE
tam	stat3	http://www.ncbi.nlm.nih.gov/entrez/query.fcgi?cmd=Retrieve&db=pubmed&dopt=Abstract&list_uids=27367025|6|Oncotarget|||Jun|2016|JOURNAL ARTICLE
tlr2	tnfsf13	http://www.ncbi.nlm.nih.gov/entrez/query.fcgi?cmd=Retrieve&db=pubmed&dopt=Abstract&list_uids=27368535|6|Fish Shellfish Immunol|56||Sep|2016|Journal Article
tlr2	slc6a4	http://www.ncbi.nlm.nih.gov/entrez/query.fcgi?cmd=Retrieve&db=pubmed&dopt=Abstract&list_uids=27488594|6|Microb Ecol|||Aug|2016|JOURNAL ARTICLE
tlr2	tlr10	http://www.ncbi.nlm.nih.gov/entrez/query.fcgi?cmd=Retrieve&db=pubmed&dopt=Abstract&list_uids=27488594|6|Microb Ecol|||Aug|2016|JOURNAL ARTICLE
tlr1	tnfsf13	http://www.ncbi.nlm.nih.gov/entrez/query.fcgi?cmd=Retrieve&db=pubmed&dopt=Abstract&list_uids=27368535|6|Fish Shellfish Immunol|56||Sep|2016|Journal Article

tlr1	tlr2	http://www.ncbi.nlm.nih.gov/entrez/query.fcgi?cmd=Retrieve&db=pubmed&dopt=Abstract&list_uids=27368535|6|Fish Shellfish Immunol|56||Sep|2016|Journal Article
mmp15	itk	http://www.ncbi.nlm.nih.gov/entrez/query.fcgi?cmd=Retrieve&db=pubmed&dopt=Abstract&list_uids=27374080|6|Oncotarget|||Jun|2016|JOURNAL ARTICLE
il8ra	msc	http://www.ncbi.nlm.nih.gov/entrez/query.fcgi?cmd=Retrieve&db=pubmed&dopt=Abstract&list_uids=27374099|6|Oncotarget|||Jun|2016|JOURNAL ARTICLE
il8ra	il8rb	http://www.ncbi.nlm.nih.gov/entrez/query.fcgi?cmd=Retrieve&db=pubmed&dopt=Abstract&list_uids=27374099|6|Oncotarget|||Jun|2016|JOURNAL ARTICLE
il8ra	il6	http://www.ncbi.nlm.nih.gov/entrez/query.fcgi?cmd=Retrieve&db=pubmed&dopt=Abstract&list_uids=27558278|6|J Periodontal Res|||Aug|2016|JOURNAL ARTICLE
il8ra	il8	http://www.ncbi.nlm.nih.gov/entrez/query.fcgi?cmd=Retrieve&db=pubmed&dopt=Abstract&list_uids=27558278|6|J Periodontal Res|||Aug|2016|JOURNAL ARTICLE
il8ra	ccl20	http://www.ncbi.nlm.nih.gov/entrez/query.fcgi?cmd=Retrieve&db=pubmed&dopt=Abstract&list_uids=27558278|6|J Periodontal Res|||Aug|2016|JOURNAL ARTICLE
il8ra	tnfsf13	http://www.ncbi.nlm.nih.gov/entrez/query.fcgi?cmd=Retrieve&db=pubmed&dopt=Abstract&list_uids=27558278|6|J Periodontal Res|||Aug|2016|JOURNAL ARTICLE
igf1r	mirn182	http://www.ncbi.nlm.nih.gov/entrez/query.fcgi?cmd=Retrieve&db=pubmed&dopt=Abstract&list_uids=27468875|6|Neoplasma|63|5|Jul|2016|JOURNAL ARTICLE
igf1r	pik3ca	http://www.ncbi.nlm.nih.gov/entrez/query.fcgi?cmd=Retrieve&db=pubmed&dopt=Abstract&list_uids=27531475|6|Zhonghua Zhong Liu Za Zhi|38|8|Aug|2016|English Abstract,Journal Article
insr	igf1r	http://www.ncbi.nlm.nih.gov/entrez/query.fcgi?cmd=Retrieve&db=pubmed&dopt=Abstract&list_uids=27374103|6|Oncotarget|||Jun|2016|JOURNAL ARTICLE
erbb4	igf1r	http://www.ncbi.nlm.nih.gov/entrez/query.fcgi?cmd=Retrieve&db=pubmed&dopt=Abstract&list_uids=27374103|6|Oncotarget|||Jun|2016|JOURNAL ARTICLE
erbb4	insr	http://www.ncbi.nlm.nih.gov/entrez/query.fcgi?cmd=Retrieve&db=pubmed&dopt=Abstract&list_uids=27374103|6|Oncotarget|||Jun|2016|JOURNAL ARTICLE
erbb4	egfr	http://www.ncbi.nlm.nih.gov/entrez/query.fcgi?cmd=Retrieve&db=pubmed&dopt=Abstract&list_uids=27374103|6|Oncotarget|||Jun|2016|JOURNAL ARTICLE
pdgfrb	pdgfb	http://www.ncbi.nlm.nih.gov/entrez/query.fcgi?cmd=Retrieve&db=pubmed&dopt=Abstract&list_uids=27374103|6|Oncotarget|||Jun|2016|JOURNAL ARTICLE
pdgfrb	ptk2	http://www.ncbi.nlm.nih.gov/entrez/query.fcgi?cmd=Retrieve&db=pubmed&dopt=Abstract&list_uids=27374103|6|Oncotarget|||Jun|2016|JOURNAL ARTICLE
col1a1	pdgfb	http://www.ncbi.nlm.nih.gov/entrez/query.fcgi?cmd=Retrieve&db=pubmed&dopt=Abstract&list_uids=27374103|6|Oncotarget|||Jun|2016|JOURNAL ARTICLE
agrp	ephb2	http://www.ncbi.nlm.nih.gov/entrez/query.fcgi?cmd=Retrieve&db=pubmed&dopt=Abstract&list_uids=27374177|6|Oncotarget|||Jun|2016|JOURNAL ARTICLE

agrp	akt1	http://www.ncbi.nlm.nih.gov/entrez/query.fcgi?cmd=Retrieve&db=pubmed&dopt=Abstract&list_uids=27374177|6|Oncotarget|||Jun|2016|JOURNAL ARTICLE
art3	ephb2	http://www.ncbi.nlm.nih.gov/entrez/query.fcgi?cmd=Retrieve&db=pubmed&dopt=Abstract&list_uids=27374177|6|Oncotarget|||Jun|2016|JOURNAL ARTICLE
art3	akt1	http://www.ncbi.nlm.nih.gov/entrez/query.fcgi?cmd=Retrieve&db=pubmed&dopt=Abstract&list_uids=27374177|6|Oncotarget|||Jun|2016|JOURNAL ARTICLE
nkd2	ervk2	http://www.ncbi.nlm.nih.gov/entrez/query.fcgi?cmd=Retrieve&db=pubmed&dopt=Abstract&list_uids=27374455|6|J Thorac Oncol|||Jun|2016|JOURNAL ARTICLE
ceacam5	ceacam3	http://www.ncbi.nlm.nih.gov/entrez/query.fcgi?cmd=Retrieve&db=pubmed&dopt=Abstract&list_uids=27376801|6|J Huazhong Univ Sci Technolog Med Sci|36|3|Jun|2016|Journal Article
ptx3	plau	http://www.ncbi.nlm.nih.gov/entrez/query.fcgi?cmd=Retrieve&db=pubmed&dopt=Abstract&list_uids=27377307|6|Sci Rep|6|||2016|Journal Article
ptx3	mmp9	http://www.ncbi.nlm.nih.gov/entrez/query.fcgi?cmd=Retrieve&db=pubmed&dopt=Abstract&list_uids=27377307|6|Sci Rep|6|||2016|Journal Article
ptx3	mmp2	http://www.ncbi.nlm.nih.gov/entrez/query.fcgi?cmd=Retrieve&db=pubmed&dopt=Abstract&list_uids=27377307|6|Sci Rep|6|||2016|Journal Article
ptx3	ifi27	http://www.ncbi.nlm.nih.gov/entrez/query.fcgi?cmd=Retrieve&db=pubmed&dopt=Abstract&list_uids=27377307|6|Sci Rep|6|||2016|Journal Article
ptx3	cdkn1a	http://www.ncbi.nlm.nih.gov/entrez/query.fcgi?cmd=Retrieve&db=pubmed&dopt=Abstract&list_uids=27377307|6|Sci Rep|6|||2016|Journal Article
ptx3	cdc25c	http://www.ncbi.nlm.nih.gov/entrez/query.fcgi?cmd=Retrieve&db=pubmed&dopt=Abstract&list_uids=27377307|6|Sci Rep|6|||2016|Journal Article
ptx3	cdc2	http://www.ncbi.nlm.nih.gov/entrez/query.fcgi?cmd=Retrieve&db=pubmed&dopt=Abstract&list_uids=27377307|6|Sci Rep|6|||2016|Journal Article
cdc25c	ifi27	http://www.ncbi.nlm.nih.gov/entrez/query.fcgi?cmd=Retrieve&db=pubmed&dopt=Abstract&list_uids=27377307|6|Sci Rep|6|||2016|Journal Article
cdc25c	cdkn1a	http://www.ncbi.nlm.nih.gov/entrez/query.fcgi?cmd=Retrieve&db=pubmed&dopt=Abstract&list_uids=27377307|6|Sci Rep|6|||2016|Journal Article
cdc2	ifi27	http://www.ncbi.nlm.nih.gov/entrez/query.fcgi?cmd=Retrieve&db=pubmed&dopt=Abstract&list_uids=27377307|6|Sci Rep|6|||2016|Journal Article
cdc2	cdkn1a	http://www.ncbi.nlm.nih.gov/entrez/query.fcgi?cmd=Retrieve&db=pubmed&dopt=Abstract&list_uids=27377307|6|Sci Rep|6|||2016|Journal Article
cdc2	cdc25c	http://www.ncbi.nlm.nih.gov/entrez/query.fcgi?cmd=Retrieve&db=pubmed&dopt=Abstract&list_uids=27377307|6|Sci Rep|6|||2016|Journal Article
cdc2	wee1	http://www.ncbi.nlm.nih.gov/entrez/query.fcgi?cmd=Retrieve&db=pubmed&dopt=Abstract&list_uids=27501501|6|Biomed Pharmacother|83||Aug|2016|JOURNAL ARTICLE

scrib	hras	http://www.ncbi.nlm.nih.gov/entrez/query.fcgi?cmd=Retrieve&db=pubmed&dopt=Abstract&list_uids=27380321|6|Nat Chem Biol|12|9|Sep|2016|Journal Article
scrib	yap1	http://www.ncbi.nlm.nih.gov/entrez/query.fcgi?cmd=Retrieve&db=pubmed&dopt=Abstract&list_uids=27380321|6|Nat Chem Biol|12|9|Sep|2016|Journal Article
zdhhc7	hras	http://www.ncbi.nlm.nih.gov/entrez/query.fcgi?cmd=Retrieve&db=pubmed&dopt=Abstract&list_uids=27380321|6|Nat Chem Biol|12|9|Sep|2016|Journal Article
zdhhc7	yap1	http://www.ncbi.nlm.nih.gov/entrez/query.fcgi?cmd=Retrieve&db=pubmed&dopt=Abstract&list_uids=27380321|6|Nat Chem Biol|12|9|Sep|2016|Journal Article
zdhhc7	scrib	http://www.ncbi.nlm.nih.gov/entrez/query.fcgi?cmd=Retrieve&db=pubmed&dopt=Abstract&list_uids=27380321|6|Nat Chem Biol|12|9|Sep|2016|Journal Article
bmp1	paep	http://www.ncbi.nlm.nih.gov/entrez/query.fcgi?cmd=Retrieve&db=pubmed&dopt=Abstract&list_uids=27384474|6|Oncotarget|||Feb|2016|JOURNAL ARTICLE
sohlh2	il8	http://www.ncbi.nlm.nih.gov/entrez/query.fcgi?cmd=Retrieve&db=pubmed&dopt=Abstract&list_uids=27384482|6|Oncotarget|||Jun|2016|JOURNAL ARTICLE
sohlh2	itk	http://www.ncbi.nlm.nih.gov/entrez/query.fcgi?cmd=Retrieve&db=pubmed&dopt=Abstract&list_uids=27384482|6|Oncotarget|||Jun|2016|JOURNAL ARTICLE
fos	axl	http://www.ncbi.nlm.nih.gov/entrez/query.fcgi?cmd=Retrieve&db=pubmed&dopt=Abstract&list_uids=27385093|6|Oncotarget|||Jul|2016|JOURNAL ARTICLE
fos	pcsk1	http://www.ncbi.nlm.nih.gov/entrez/query.fcgi?cmd=Retrieve&db=pubmed&dopt=Abstract&list_uids=27385093|6|Oncotarget|||Jul|2016|JOURNAL ARTICLE
fos	plau	http://www.ncbi.nlm.nih.gov/entrez/query.fcgi?cmd=Retrieve&db=pubmed&dopt=Abstract&list_uids=27385093|6|Oncotarget|||Jul|2016|JOURNAL ARTICLE
fos	jun	http://www.ncbi.nlm.nih.gov/entrez/query.fcgi?cmd=Retrieve&db=pubmed&dopt=Abstract&list_uids=27385093|6|Oncotarget|||Jul|2016|JOURNAL ARTICLE
fos	fosl2	http://www.ncbi.nlm.nih.gov/entrez/query.fcgi?cmd=Retrieve&db=pubmed&dopt=Abstract&list_uids=27604827|6|Prostate|||Sep|2016|JOURNAL ARTICLE
fos	fosl1	http://www.ncbi.nlm.nih.gov/entrez/query.fcgi?cmd=Retrieve&db=pubmed&dopt=Abstract&list_uids=27604827|6|Prostate|||Sep|2016|JOURNAL ARTICLE
fos	fosb	http://www.ncbi.nlm.nih.gov/entrez/query.fcgi?cmd=Retrieve&db=pubmed&dopt=Abstract&list_uids=27604827|6|Prostate|||Sep|2016|JOURNAL ARTICLE
fos	jund	http://www.ncbi.nlm.nih.gov/entrez/query.fcgi?cmd=Retrieve&db=pubmed&dopt=Abstract&list_uids=27604827|6|Prostate|||Sep|2016|JOURNAL ARTICLE
fos	junb	http://www.ncbi.nlm.nih.gov/entrez/query.fcgi?cmd=Retrieve&db=pubmed&dopt=Abstract&list_uids=27604827|6|Prostate|||Sep|2016|JOURNAL ARTICLE
atf2	axl	http://www.ncbi.nlm.nih.gov/entrez/query.fcgi?cmd=Retrieve&db=pubmed&dopt=Abstract&list_uids=27385093|6|Oncotarget|||Jul|2016|JOURNAL ARTICLE

atf2	fos	http://www.ncbi.nlm.nih.gov/entrez/query.fcgi?cmd=Retrieve&db=pubmed&dopt=Abstract&list_uids=27385093|6|Oncotarget|||Jul|2016|JOURNAL ARTICLE
atf2	snord87	http://www.ncbi.nlm.nih.gov/entrez/query.fcgi?cmd=Retrieve&db=pubmed&dopt=Abstract&list_uids=27588402|6|Oncotarget|||Aug|2016|JOURNAL ARTICLE
tmprss4	pcsk1	http://www.ncbi.nlm.nih.gov/entrez/query.fcgi?cmd=Retrieve&db=pubmed&dopt=Abstract&list_uids=27385093|6|Oncotarget|||Jul|2016|JOURNAL ARTICLE
tmprss4	plau	http://www.ncbi.nlm.nih.gov/entrez/query.fcgi?cmd=Retrieve&db=pubmed&dopt=Abstract&list_uids=27385093|6|Oncotarget|||Jul|2016|JOURNAL ARTICLE
tmprss4	jun	http://www.ncbi.nlm.nih.gov/entrez/query.fcgi?cmd=Retrieve&db=pubmed&dopt=Abstract&list_uids=27385093|6|Oncotarget|||Jul|2016|JOURNAL ARTICLE
tmprss4	itk	http://www.ncbi.nlm.nih.gov/entrez/query.fcgi?cmd=Retrieve&db=pubmed&dopt=Abstract&list_uids=27385093|6|Oncotarget|||Jul|2016|JOURNAL ARTICLE
tmprss4	fos	http://www.ncbi.nlm.nih.gov/entrez/query.fcgi?cmd=Retrieve&db=pubmed&dopt=Abstract&list_uids=27385093|6|Oncotarget|||Jul|2016|JOURNAL ARTICLE
pcsk1	ifi27	http://www.ncbi.nlm.nih.gov/entrez/query.fcgi?cmd=Retrieve&db=pubmed&dopt=Abstract&list_uids=27569589|6|Life Sci|||Aug|2016|JOURNAL ARTICLE
pcsk1	cdkn1a	http://www.ncbi.nlm.nih.gov/entrez/query.fcgi?cmd=Retrieve&db=pubmed&dopt=Abstract&list_uids=27569589|6|Life Sci|||Aug|2016|JOURNAL ARTICLE
nampt	xrcc1	http://www.ncbi.nlm.nih.gov/entrez/query.fcgi?cmd=Retrieve&db=pubmed&dopt=Abstract&list_uids=27390344|6|Mol Cancer Ther|15|9|Sep|2016|Journal Article
nampt	pak4	http://www.ncbi.nlm.nih.gov/entrez/query.fcgi?cmd=Retrieve&db=pubmed&dopt=Abstract&list_uids=27390344|6|Mol Cancer Ther|15|9|Sep|2016|Journal Article
naprt1	nampt	http://www.ncbi.nlm.nih.gov/entrez/query.fcgi?cmd=Retrieve&db=pubmed&dopt=Abstract&list_uids=27390344|6|Mol Cancer Ther|15|9|Sep|2016|Journal Article
naprt1	xrcc1	http://www.ncbi.nlm.nih.gov/entrez/query.fcgi?cmd=Retrieve&db=pubmed&dopt=Abstract&list_uids=27390344|6|Mol Cancer Ther|15|9|Sep|2016|Journal Article
pak4	myc	http://www.ncbi.nlm.nih.gov/entrez/query.fcgi?cmd=Retrieve&db=pubmed&dopt=Abstract&list_uids=27390344|6|Mol Cancer Ther|15|9|Sep|2016|Journal Article
pak4	xrcc1	http://www.ncbi.nlm.nih.gov/entrez/query.fcgi?cmd=Retrieve&db=pubmed&dopt=Abstract&list_uids=27390344|6|Mol Cancer Ther|15|9|Sep|2016|Journal Article
ctgf	plau	http://www.ncbi.nlm.nih.gov/entrez/query.fcgi?cmd=Retrieve&db=pubmed&dopt=Abstract&list_uids=27391090|6|PLoS One|11|7||2016|Journal Article
ctgf	mapk14	http://www.ncbi.nlm.nih.gov/entrez/query.fcgi?cmd=Retrieve&db=pubmed&dopt=Abstract&list_uids=27403934|6|Cancer Sci|||Jul|2016|JOURNAL ARTICLE
ccl2	plau	http://www.ncbi.nlm.nih.gov/entrez/query.fcgi?cmd=Retrieve&db=pubmed&dopt=Abstract&list_uids=27391090|6|PLoS One|11|7||2016|Journal Article

ccl2	ctgf	http://www.ncbi.nlm.nih.gov/entrez/query.fcgi?cmd=Retrieve&db=pubmed&dopt=Abstract&list_uids=27391090|6|PLoS One|11|7||2016|Journal Article
ccl2	rela	http://www.ncbi.nlm.nih.gov/entrez/query.fcgi?cmd=Retrieve&db=pubmed&dopt=Abstract&list_uids=27486225|6|Mol Cancer Ther|||Aug|2016|JOURNAL ARTICLE
mmp1	plau	http://www.ncbi.nlm.nih.gov/entrez/query.fcgi?cmd=Retrieve&db=pubmed&dopt=Abstract&list_uids=27391090|6|PLoS One|11|7||2016|Journal Article
mmp1	ctgf	http://www.ncbi.nlm.nih.gov/entrez/query.fcgi?cmd=Retrieve&db=pubmed&dopt=Abstract&list_uids=27391090|6|PLoS One|11|7||2016|Journal Article
mmp1	ccl2	http://www.ncbi.nlm.nih.gov/entrez/query.fcgi?cmd=Retrieve&db=pubmed&dopt=Abstract&list_uids=27391090|6|PLoS One|11|7||2016|Journal Article
mmp1	mmp13	http://www.ncbi.nlm.nih.gov/entrez/query.fcgi?cmd=Retrieve&db=pubmed&dopt=Abstract&list_uids=27491937|6|Anticancer Agents Med Chem|||Aug|2016|JOURNAL ARTICLE
mmp1	mmp8	http://www.ncbi.nlm.nih.gov/entrez/query.fcgi?cmd=Retrieve&db=pubmed&dopt=Abstract&list_uids=27491937|6|Anticancer Agents Med Chem|||Aug|2016|JOURNAL ARTICLE
prame	plau	http://www.ncbi.nlm.nih.gov/entrez/query.fcgi?cmd=Retrieve&db=pubmed&dopt=Abstract&list_uids=27391090|6|PLoS One|11|7||2016|Journal Article
prame	ctgf	http://www.ncbi.nlm.nih.gov/entrez/query.fcgi?cmd=Retrieve&db=pubmed&dopt=Abstract&list_uids=27391090|6|PLoS One|11|7||2016|Journal Article
prame	ccl2	http://www.ncbi.nlm.nih.gov/entrez/query.fcgi?cmd=Retrieve&db=pubmed&dopt=Abstract&list_uids=27391090|6|PLoS One|11|7||2016|Journal Article
prame	mmp1	http://www.ncbi.nlm.nih.gov/entrez/query.fcgi?cmd=Retrieve&db=pubmed&dopt=Abstract&list_uids=27391090|6|PLoS One|11|7||2016|Journal Article
nf-kappab	rela	http://www.ncbi.nlm.nih.gov/entrez/query.fcgi?cmd=Retrieve&db=pubmed&dopt=Abstract&list_uids=27393618|6|Breast Cancer Res Treat|158|3|Aug|2016|Journal Article
dusp4	rela	http://www.ncbi.nlm.nih.gov/entrez/query.fcgi?cmd=Retrieve&db=pubmed&dopt=Abstract&list_uids=27393618|6|Breast Cancer Res Treat|158|3|Aug|2016|Journal Article
dusp4	nf-kappab	http://www.ncbi.nlm.nih.gov/entrez/query.fcgi?cmd=Retrieve&db=pubmed&dopt=Abstract&list_uids=27393618|6|Breast Cancer Res Treat|158|3|Aug|2016|Journal Article
dusp4	mapk8	http://www.ncbi.nlm.nih.gov/entrez/query.fcgi?cmd=Retrieve&db=pubmed&dopt=Abstract&list_uids=27393618|6|Breast Cancer Res Treat|158|3|Aug|2016|Journal Article
dusp4	mapk14	http://www.ncbi.nlm.nih.gov/entrez/query.fcgi?cmd=Retrieve&db=pubmed&dopt=Abstract&list_uids=27393618|6|Breast Cancer Res Treat|158|3|Aug|2016|Journal Article
dusp4	mapk3	http://www.ncbi.nlm.nih.gov/entrez/query.fcgi?cmd=Retrieve&db=pubmed&dopt=Abstract&list_uids=27393618|6|Breast Cancer Res Treat|158|3|Aug|2016|Journal Article
notch1	mmp9	http://www.ncbi.nlm.nih.gov/entrez/query.fcgi?cmd=Retrieve&db=pubmed&dopt=Abstract&list_uids=27398151|6|Am J Transl Res|8|6||2016|Journal Article

notch1	gsc	http://www.ncbi.nlm.nih.gov/entrez/query.fcgi?cmd=Retrieve&db=pubmed&dopt=Abstract&list_uids=27472461|6|Oncotarget|||Jul|2016|JOURNAL ARTICLE
notch1	rbpj	http://www.ncbi.nlm.nih.gov/entrez/query.fcgi?cmd=Retrieve&db=pubmed&dopt=Abstract&list_uids=27472461|6|Oncotarget|||Jul|2016|JOURNAL ARTICLE
notch1	myc	http://www.ncbi.nlm.nih.gov/entrez/query.fcgi?cmd=Retrieve&db=pubmed&dopt=Abstract&list_uids=27472461|6|Oncotarget|||Jul|2016|JOURNAL ARTICLE
notch1	dll1	http://www.ncbi.nlm.nih.gov/entrez/query.fcgi?cmd=Retrieve&db=pubmed&dopt=Abstract&list_uids=27472461|6|Oncotarget|||Jul|2016|JOURNAL ARTICLE
mirn143	ctgf	http://www.ncbi.nlm.nih.gov/entrez/query.fcgi?cmd=Retrieve&db=pubmed&dopt=Abstract&list_uids=27398154|6|Am J Transl Res|8|6||2016|Journal Article
mirn221	nostrin	http://www.ncbi.nlm.nih.gov/entrez/query.fcgi?cmd=Retrieve&db=pubmed&dopt=Abstract&list_uids=27401251|6|Clin Cancer Res|||Jul|2016|JOURNAL ARTICLE
mirn221	uts2r	http://www.ncbi.nlm.nih.gov/entrez/query.fcgi?cmd=Retrieve&db=pubmed&dopt=Abstract&list_uids=27401251|6|Clin Cancer Res|||Jul|2016|JOURNAL ARTICLE
cyld	na	http://www.ncbi.nlm.nih.gov/entrez/query.fcgi?cmd=Retrieve&db=pubmed&dopt=Abstract&list_uids=27405163|6|Yao Xue Xue Bao|51|1|Jan|2016|English Abstract,Journal Article
ly96	irf6	http://www.ncbi.nlm.nih.gov/entrez/query.fcgi?cmd=Retrieve&db=pubmed&dopt=Abstract&list_uids=27409669|6|Oncotarget|||Jul|2016|JOURNAL ARTICLE
nr0b1	ly96	http://www.ncbi.nlm.nih.gov/entrez/query.fcgi?cmd=Retrieve&db=pubmed&dopt=Abstract&list_uids=27409669|6|Oncotarget|||Jul|2016|JOURNAL ARTICLE
col2a1	ly96	http://www.ncbi.nlm.nih.gov/entrez/query.fcgi?cmd=Retrieve&db=pubmed&dopt=Abstract&list_uids=27409669|6|Oncotarget|||Jul|2016|JOURNAL ARTICLE
col2a1	nr0b1	http://www.ncbi.nlm.nih.gov/entrez/query.fcgi?cmd=Retrieve&db=pubmed&dopt=Abstract&list_uids=27409669|6|Oncotarget|||Jul|2016|JOURNAL ARTICLE
creb1	cdkn1b	http://www.ncbi.nlm.nih.gov/entrez/query.fcgi?cmd=Retrieve&db=pubmed&dopt=Abstract&list_uids=27591936|6|Cancer Lett|||Aug|2016|JOURNAL ARTICLE
creb1	ifi27	http://www.ncbi.nlm.nih.gov/entrez/query.fcgi?cmd=Retrieve&db=pubmed&dopt=Abstract&list_uids=27591936|6|Cancer Lett|||Aug|2016|JOURNAL ARTICLE
taz	wwtr1	http://www.ncbi.nlm.nih.gov/entrez/query.fcgi?cmd=Retrieve&db=pubmed&dopt=Abstract&list_uids=27412635|6|Protein Cell|7|8|Aug|2016|Journal Article,Review
epha2	hcc	http://www.ncbi.nlm.nih.gov/entrez/query.fcgi?cmd=Retrieve&db=pubmed&dopt=Abstract&list_uids=27412940|6|Xi Bao Yu Fen Zi Mian Yi Xue Za Zhi|32|8|Aug|2016|English Abstract,Journal Article
mirn141	hcc	http://www.ncbi.nlm.nih.gov/entrez/query.fcgi?cmd=Retrieve&db=pubmed&dopt=Abstract&list_uids=27412940|6|Xi Bao Yu Fen Zi Mian Yi Xue Za Zhi|32|8|Aug|2016|English Abstract,Journal Article
mirn141	epha2	http://www.ncbi.nlm.nih.gov/entrez/query.fcgi?cmd=Retrieve&db=pubmed&dopt=Abstract&list_uids=27412940|6|Xi Bao Yu Fen Zi Mian Yi Xue Za Zhi|32|8|Aug|2016|English Abstract,Journal Article

fas	vegfa	http://www.ncbi.nlm.nih.gov/entrez/query.fcgi?cmd=Retrieve&db=pubmed&dopt=Abstract&list_uids=27415751|6|Cancer Biol Ther|||Jul|2016|JOURNAL ARTICLE
fas	bcl2	http://www.ncbi.nlm.nih.gov/entrez/query.fcgi?cmd=Retrieve&db=pubmed&dopt=Abstract&list_uids=27415751|6|Cancer Biol Ther|||Jul|2016|JOURNAL ARTICLE
fas	bcl2l1	http://www.ncbi.nlm.nih.gov/entrez/query.fcgi?cmd=Retrieve&db=pubmed&dopt=Abstract&list_uids=27415751|6|Cancer Biol Ther|||Jul|2016|JOURNAL ARTICLE
fas	myc	http://www.ncbi.nlm.nih.gov/entrez/query.fcgi?cmd=Retrieve&db=pubmed&dopt=Abstract&list_uids=27415751|6|Cancer Biol Ther|||Jul|2016|JOURNAL ARTICLE
fas	stat3	http://www.ncbi.nlm.nih.gov/entrez/query.fcgi?cmd=Retrieve&db=pubmed&dopt=Abstract&list_uids=27415751|6|Cancer Biol Ther|||Jul|2016|JOURNAL ARTICLE
bcl2l1	vegfa	http://www.ncbi.nlm.nih.gov/entrez/query.fcgi?cmd=Retrieve&db=pubmed&dopt=Abstract&list_uids=27415751|6|Cancer Biol Ther|||Jul|2016|JOURNAL ARTICLE
bcl2l1	bax	http://www.ncbi.nlm.nih.gov/entrez/query.fcgi?cmd=Retrieve&db=pubmed&dopt=Abstract&list_uids=27421660|6|Acta Biochim Biophys Sin (Shanghai)|48|8|Aug|2016|Journal Article
prkaa1	sp1	http://www.ncbi.nlm.nih.gov/entrez/query.fcgi?cmd=Retrieve&db=pubmed&dopt=Abstract&list_uids=27416558|6|Biomed Pharmacother|83||Jul|2016|JOURNAL ARTICLE
aprt	sp1	http://www.ncbi.nlm.nih.gov/entrez/query.fcgi?cmd=Retrieve&db=pubmed&dopt=Abstract&list_uids=27416558|6|Biomed Pharmacother|83||Jul|2016|JOURNAL ARTICLE
aprt	prkaa1	http://www.ncbi.nlm.nih.gov/entrez/query.fcgi?cmd=Retrieve&db=pubmed&dopt=Abstract&list_uids=27416558|6|Biomed Pharmacother|83||Jul|2016|JOURNAL ARTICLE
dnmt1	sp1	http://www.ncbi.nlm.nih.gov/entrez/query.fcgi?cmd=Retrieve&db=pubmed&dopt=Abstract&list_uids=27416558|6|Biomed Pharmacother|83||Jul|2016|JOURNAL ARTICLE
dnmt1	prkaa1	http://www.ncbi.nlm.nih.gov/entrez/query.fcgi?cmd=Retrieve&db=pubmed&dopt=Abstract&list_uids=27416558|6|Biomed Pharmacother|83||Jul|2016|JOURNAL ARTICLE
dnmt1	aprt	http://www.ncbi.nlm.nih.gov/entrez/query.fcgi?cmd=Retrieve&db=pubmed&dopt=Abstract&list_uids=27416558|6|Biomed Pharmacother|83||Jul|2016|JOURNAL ARTICLE
dnmt1	ros1	http://www.ncbi.nlm.nih.gov/entrez/query.fcgi?cmd=Retrieve&db=pubmed&dopt=Abstract&list_uids=27416558|6|Biomed Pharmacother|83||Jul|2016|JOURNAL ARTICLE
hdac9	tmprss 11d	http://www.ncbi.nlm.nih.gov/entrez/query.fcgi?cmd=Retrieve&db=pubmed&dopt=Abstract&list_uids=27429844|6|Am J Cancer Res|6|6||2016|Journal Article
hdac9	hdac8	http://www.ncbi.nlm.nih.gov/entrez/query.fcgi?cmd=Retrieve&db=pubmed&dopt=Abstract&list_uids=27429844|6|Am J Cancer Res|6|6||2016|Journal Article
hdac9	hdac3	http://www.ncbi.nlm.nih.gov/entrez/query.fcgi?cmd=Retrieve&db=pubmed&dopt=Abstract&list_uids=27429844|6|Am J Cancer Res|6|6||2016|Journal Article
hdac9	hdac2	http://www.ncbi.nlm.nih.gov/entrez/query.fcgi?cmd=Retrieve&db=pubmed&dopt=Abstract&list_uids=27429844|6|Am J Cancer Res|6|6||2016|Journal Article

hdac9	hdac1	http://www.ncbi.nlm.nih.gov/entrez/query.fcgi?cmd=Retrieve&db=pubmed&dopt=Abstract&list_uids=27429844|6|Am J Cancer Res|6|6||2016|Journal Article
hdac9	prdx2	http://www.ncbi.nlm.nih.gov/entrez/query.fcgi?cmd=Retrieve&db=pubmed&dopt=Abstract&list_uids=27507059|6|Oncotarget|||Aug|2016|JOURNAL ARTICLE
prex1	hdac9	http://www.ncbi.nlm.nih.gov/entrez/query.fcgi?cmd=Retrieve&db=pubmed&dopt=Abstract&list_uids=27418645|6|Mol Cancer Res|||Jul|2016|JOURNAL ARTICLE
prex1	cdc42	http://www.ncbi.nlm.nih.gov/entrez/query.fcgi?cmd=Retrieve&db=pubmed&dopt=Abstract&list_uids=27418645|6|Mol Cancer Res|||Jul|2016|JOURNAL ARTICLE
prex1	rac1	http://www.ncbi.nlm.nih.gov/entrez/query.fcgi?cmd=Retrieve&db=pubmed&dopt=Abstract&list_uids=27418645|6|Mol Cancer Res|||Jul|2016|JOURNAL ARTICLE
prex1	ephb2	http://www.ncbi.nlm.nih.gov/entrez/query.fcgi?cmd=Retrieve&db=pubmed&dopt=Abstract&list_uids=27418645|6|Mol Cancer Res|||Jul|2016|JOURNAL ARTICLE
cic	tspan8	http://www.ncbi.nlm.nih.gov/entrez/query.fcgi?cmd=Retrieve&db=pubmed&dopt=Abstract&list_uids=27419629|6|Oncotarget|||Jul|2016|JOURNAL ARTICLE
cic	cd44	http://www.ncbi.nlm.nih.gov/entrez/query.fcgi?cmd=Retrieve&db=pubmed&dopt=Abstract&list_uids=27419629|6|Oncotarget|||Jul|2016|JOURNAL ARTICLE
tsc1	frap1	http://www.ncbi.nlm.nih.gov/entrez/query.fcgi?cmd=Retrieve&db=pubmed&dopt=Abstract&list_uids=27421126|6|Wiad Lek|69|2 Pt 1||2016|English Abstract,Journal Article
tsc1	slc12a3	http://www.ncbi.nlm.nih.gov/entrez/query.fcgi?cmd=Retrieve&db=pubmed&dopt=Abstract&list_uids=27421126|6|Wiad Lek|69|2 Pt 1||2016|English Abstract,Journal Article
slc12a3	frap1	http://www.ncbi.nlm.nih.gov/entrez/query.fcgi?cmd=Retrieve&db=pubmed&dopt=Abstract&list_uids=27421126|6|Wiad Lek|69|2 Pt 1||2016|English Abstract,Journal Article
ing4	bax	http://www.ncbi.nlm.nih.gov/entrez/query.fcgi?cmd=Retrieve&db=pubmed&dopt=Abstract&list_uids=27421660|6|Acta Biochim Biophys Sin (Shanghai)|48|8|Aug|2016|Journal Article
ing4	bcl2l1	http://www.ncbi.nlm.nih.gov/entrez/query.fcgi?cmd=Retrieve&db=pubmed&dopt=Abstract&list_uids=27421660|6|Acta Biochim Biophys Sin (Shanghai)|48|8|Aug|2016|Journal Article
ing4	bcl2	http://www.ncbi.nlm.nih.gov/entrez/query.fcgi?cmd=Retrieve&db=pubmed&dopt=Abstract&list_uids=27421660|6|Acta Biochim Biophys Sin (Shanghai)|48|8|Aug|2016|Journal Article
ing4	cdkn1a	http://www.ncbi.nlm.nih.gov/entrez/query.fcgi?cmd=Retrieve&db=pubmed&dopt=Abstract&list_uids=27421660|6|Acta Biochim Biophys Sin (Shanghai)|48|8|Aug|2016|Journal Article
ing4	tp53	http://www.ncbi.nlm.nih.gov/entrez/query.fcgi?cmd=Retrieve&db=pubmed&dopt=Abstract&list_uids=27421660|6|Acta Biochim Biophys Sin (Shanghai)|48|8|Aug|2016|Journal Article
ing4	pten	http://www.ncbi.nlm.nih.gov/entrez/query.fcgi?cmd=Retrieve&db=pubmed&dopt=Abstract&list_uids=27421660|6|Acta Biochim Biophys Sin (Shanghai)|48|8|Aug|2016|Journal Article
il13	alcam	http://www.ncbi.nlm.nih.gov/entrez/query.fcgi?cmd=Retrieve&db=pubmed&dopt=Abstract&list_uids=27533463|6|Oncotarget|||Aug|2016|JOURNAL ARTICLE

il13	prom1	http://www.ncbi.nlm.nih.gov/entrez/query.fcgi?cmd=Retrieve&db=pubmed&dopt=Abstract&list_uids=27533463|6|Oncotarget|||Aug|2016|JOURNAL ARTICLE
il13	cd44	http://www.ncbi.nlm.nih.gov/entrez/query.fcgi?cmd=Retrieve&db=pubmed&dopt=Abstract&list_uids=27533463|6|Oncotarget|||Aug|2016|JOURNAL ARTICLE
il13	nanog	http://www.ncbi.nlm.nih.gov/entrez/query.fcgi?cmd=Retrieve&db=pubmed&dopt=Abstract&list_uids=27533463|6|Oncotarget|||Aug|2016|JOURNAL ARTICLE
il13	zeb1	http://www.ncbi.nlm.nih.gov/entrez/query.fcgi?cmd=Retrieve&db=pubmed&dopt=Abstract&list_uids=27533463|6|Oncotarget|||Aug|2016|JOURNAL ARTICLE
il13	itk	http://www.ncbi.nlm.nih.gov/entrez/query.fcgi?cmd=Retrieve&db=pubmed&dopt=Abstract&list_uids=27533463|6|Oncotarget|||Aug|2016|JOURNAL ARTICLE
il13	phgdh	http://www.ncbi.nlm.nih.gov/entrez/query.fcgi?cmd=Retrieve&db=pubmed&dopt=Abstract&list_uids=27593106|6|J Hepatol|||Sep|2016|JOURNAL ARTICLE
il13	fbn2	http://www.ncbi.nlm.nih.gov/entrez/query.fcgi?cmd=Retrieve&db=pubmed&dopt=Abstract&list_uids=27593106|6|J Hepatol|||Sep|2016|JOURNAL ARTICLE
il13	il34	http://www.ncbi.nlm.nih.gov/entrez/query.fcgi?cmd=Retrieve&db=pubmed&dopt=Abstract&list_uids=27593106|6|J Hepatol|||Sep|2016|JOURNAL ARTICLE
zfp36	il13	http://www.ncbi.nlm.nih.gov/entrez/query.fcgi?cmd=Retrieve&db=pubmed&dopt=Abstract&list_uids=27424080|6|Int Immunopharmacol|39||Oct|2016|Journal Article
zfp36	frap1	http://www.ncbi.nlm.nih.gov/entrez/query.fcgi?cmd=Retrieve&db=pubmed&dopt=Abstract&list_uids=27424080|6|Int Immunopharmacol|39||Oct|2016|Journal Article
zfp36	akt1	http://www.ncbi.nlm.nih.gov/entrez/query.fcgi?cmd=Retrieve&db=pubmed&dopt=Abstract&list_uids=27424080|6|Int Immunopharmacol|39||Oct|2016|Journal Article
zfp36	pik3ca	http://www.ncbi.nlm.nih.gov/entrez/query.fcgi?cmd=Retrieve&db=pubmed&dopt=Abstract&list_uids=27424080|6|Int Immunopharmacol|39||Oct|2016|Journal Article
uca1	bcatenin	http://www.ncbi.nlm.nih.gov/entrez/query.fcgi?cmd=Retrieve&db=pubmed&dopt=Abstract&list_uids=27424981|6|Eur Rev Med Pharmacol Sci|20|13|Jul|2016|Journal Article
uca1	itk	http://www.ncbi.nlm.nih.gov/entrez/query.fcgi?cmd=Retrieve&db=pubmed&dopt=Abstract&list_uids=27424981|6|Eur Rev Med Pharmacol Sci|20|13|Jul|2016|Journal Article
uca1	cdkn1b	http://www.ncbi.nlm.nih.gov/entrez/query.fcgi?cmd=Retrieve&db=pubmed&dopt=Abstract&list_uids=27591936|6|Cancer Lett|||Aug|2016|JOURNAL ARTICLE
uca1	ifi27	http://www.ncbi.nlm.nih.gov/entrez/query.fcgi?cmd=Retrieve&db=pubmed&dopt=Abstract&list_uids=27591936|6|Cancer Lett|||Aug|2016|JOURNAL ARTICLE
uca1	creb1	http://www.ncbi.nlm.nih.gov/entrez/query.fcgi?cmd=Retrieve&db=pubmed&dopt=Abstract&list_uids=27591936|6|Cancer Lett|||Aug|2016|JOURNAL ARTICLE
hmgb1	il10	http://www.ncbi.nlm.nih.gov/entrez/query.fcgi?cmd=Retrieve&db=pubmed&dopt=Abstract&list_uids=27426915|6|Sci Rep|6|||2016|Journal Article

hdac8	tmprss 11d	http://www.ncbi.nlm.nih.gov/entrez/query.fcgi?cmd=Retrieve&db=pubmed&dopt=Abstract&list_uids=27429844|6|Am J Cancer Res|6|6||2016|Journal Article
hdac3	tmprss 11d	http://www.ncbi.nlm.nih.gov/entrez/query.fcgi?cmd=Retrieve&db=pubmed&dopt=Abstract&list_uids=27429844|6|Am J Cancer Res|6|6||2016|Journal Article
hdac3	hdac8	http://www.ncbi.nlm.nih.gov/entrez/query.fcgi?cmd=Retrieve&db=pubmed&dopt=Abstract&list_uids=27429844|6|Am J Cancer Res|6|6||2016|Journal Article
hdac2	tmprss 11d	http://www.ncbi.nlm.nih.gov/entrez/query.fcgi?cmd=Retrieve&db=pubmed&dopt=Abstract&list_uids=27429844|6|Am J Cancer Res|6|6||2016|Journal Article
hdac2	hdac8	http://www.ncbi.nlm.nih.gov/entrez/query.fcgi?cmd=Retrieve&db=pubmed&dopt=Abstract&list_uids=27429844|6|Am J Cancer Res|6|6||2016|Journal Article
hdac2	hdac3	http://www.ncbi.nlm.nih.gov/entrez/query.fcgi?cmd=Retrieve&db=pubmed&dopt=Abstract&list_uids=27429844|6|Am J Cancer Res|6|6||2016|Journal Article
mirn223	itk	http://www.ncbi.nlm.nih.gov/entrez/query.fcgi?cmd=Retrieve&db=pubmed&dopt=Abstract&list_uids=27429851|6|Am J Cancer Res|6|6||2016|Journal Article
chrna4	akt1	http://www.ncbi.nlm.nih.gov/entrez/query.fcgi?cmd=Retrieve&db=pubmed&dopt=Abstract&list_uids=27424921|6|Biochim Biophys Acta|1860|11 Pt A|Nov|2016|Journal Article
eif2ak3	rela	http://www.ncbi.nlm.nih.gov/entrez/query.fcgi?cmd=Retrieve&db=pubmed&dopt=Abstract&list_uids=27533779|6|Mol Carcinog|||Aug|2016|JOURNAL ARTICLE
eif2ak3	ephb2	http://www.ncbi.nlm.nih.gov/entrez/query.fcgi?cmd=Retrieve&db=pubmed&dopt=Abstract&list_uids=27533779|6|Mol Carcinog|||Aug|2016|JOURNAL ARTICLE|http://www.ncbi.nlm.nih.gov/entrez/query.fcgi?cmd=Retrieve&db=pubmed&dopt=Abstract&list_uids=27572503|6|Oncol Rep|||Aug|2016|JOURNAL ARTICLE
eif2ak3	na	http://www.ncbi.nlm.nih.gov/entrez/query.fcgi?cmd=Retrieve&db=pubmed&dopt=Abstract&list_uids=27572503|6|Oncol Rep|||Aug|2016|JOURNAL ARTICLE
fgfr2	eif2ak3	http://www.ncbi.nlm.nih.gov/entrez/query.fcgi?cmd=Retrieve&db=pubmed&dopt=Abstract&list_uids=27430377|6|Oncol Rep|36|3|Sep|2016|Journal Article
nudt6	eif2ak3	http://www.ncbi.nlm.nih.gov/entrez/query.fcgi?cmd=Retrieve&db=pubmed&dopt=Abstract&list_uids=27430377|6|Oncol Rep|36|3|Sep|2016|Journal Article
nudt6	fgfr2	http://www.ncbi.nlm.nih.gov/entrez/query.fcgi?cmd=Retrieve&db=pubmed&dopt=Abstract&list_uids=27430377|6|Oncol Rep|36|3|Sep|2016|Journal Article
sfn	pcsk1	http://www.ncbi.nlm.nih.gov/entrez/query.fcgi?cmd=Retrieve&db=pubmed&dopt=Abstract&list_uids=27430422|6|Oncol Rep|36|3|Sep|2016|Journal Article
macc1	itk	http://www.ncbi.nlm.nih.gov/entrez/query.fcgi?cmd=Retrieve&db=pubmed&dopt=Abstract&list_uids=27492459|6|Tumour Biol|||Aug|2016|JOURNAL ARTICLE|http://www.ncbi.nlm.nih.gov/entrez/query.fcgi?cmd=Retrieve&db=pubmed&dopt=Abstract&list_uids=27488539|6|Biosci Trends|10|4|Sep|2016|Journal Article

macc1	cyr61	http://www.ncbi.nlm.nih.gov/entrez/query.fcgi?cmd=Retrieve&db=pubmed&dopt=Abstract&list_uids=27492459|6|Tumour Biol|||Aug|2016|JOURNAL ARTICLE
macc1	tnfsf13	http://www.ncbi.nlm.nih.gov/entrez/query.fcgi?cmd=Retrieve&db=pubmed&dopt=Abstract&list_uids=27529686|6|Int J Cancer|||Aug|2016|JOURNAL ARTICLE
macc1	hgf	http://www.ncbi.nlm.nih.gov/entrez/query.fcgi?cmd=Retrieve&db=pubmed&dopt=Abstract&list_uids=27529686|6|Int J Cancer|||Aug|2016|JOURNAL ARTICLE
macc1	jun	http://www.ncbi.nlm.nih.gov/entrez/query.fcgi?cmd=Retrieve&db=pubmed&dopt=Abstract&list_uids=27529686|6|Int J Cancer|||Aug|2016|JOURNAL ARTICLE
macc1	slc9a1	http://www.ncbi.nlm.nih.gov/entrez/query.fcgi?cmd=Retrieve&db=pubmed&dopt=Abstract&list_uids=27529686|6|Int J Cancer|||Aug|2016|JOURNAL ARTICLE
ang	ace	http://www.ncbi.nlm.nih.gov/entrez/query.fcgi?cmd=Retrieve&db=pubmed&dopt=Abstract&list_uids=27431603|6|Biochim Biophys Acta|1860|11 Pt A|Nov|2016|Journal Article
ang	mas1	http://www.ncbi.nlm.nih.gov/entrez/query.fcgi?cmd=Retrieve&db=pubmed&dopt=Abstract&list_uids=27431603|6|Biochim Biophys Acta|1860|11 Pt A|Nov|2016|Journal Article
myh14	mlc1	http://www.ncbi.nlm.nih.gov/entrez/query.fcgi?cmd=Retrieve&db=pubmed&dopt=Abstract&list_uids=27432222|6|Mol Med Rep|14|3|Sep|2016|Journal Article
gfap	pax3	http://www.ncbi.nlm.nih.gov/entrez/query.fcgi?cmd=Retrieve&db=pubmed&dopt=Abstract&list_uids=27432276|6|Oncol Rep|36|3|Sep|2016|Journal Article
pcna	mmp9	http://www.ncbi.nlm.nih.gov/entrez/query.fcgi?cmd=Retrieve&db=pubmed&dopt=Abstract&list_uids=27470348|6|Biomed Pharmacother|82||Aug|2016|Journal Article
pcna	mmp2	http://www.ncbi.nlm.nih.gov/entrez/query.fcgi?cmd=Retrieve&db=pubmed&dopt=Abstract&list_uids=27470348|6|Biomed Pharmacother|82||Aug|2016|Journal Article
pcna	cdk2	http://www.ncbi.nlm.nih.gov/entrez/query.fcgi?cmd=Retrieve&db=pubmed&dopt=Abstract&list_uids=27582508|6|Biosci Rep|||Aug|2016|JOURNAL ARTICLE
cap1	pcna	http://www.ncbi.nlm.nih.gov/entrez/query.fcgi?cmd=Retrieve&db=pubmed&dopt=Abstract&list_uids=27432289|6|Oncol Rep|36|3|Sep|2016|Journal Article
mirn206	twf1	http://www.ncbi.nlm.nih.gov/entrez/query.fcgi?cmd=Retrieve&db=pubmed&dopt=Abstract&list_uids=27435395|6|Clin Cancer Res|||Jul|2016|JOURNAL ARTICLE
il11	twf1	http://www.ncbi.nlm.nih.gov/entrez/query.fcgi?cmd=Retrieve&db=pubmed&dopt=Abstract&list_uids=27435395|6|Clin Cancer Res|||Jul|2016|JOURNAL ARTICLE
il11	mirn206	http://www.ncbi.nlm.nih.gov/entrez/query.fcgi?cmd=Retrieve&db=pubmed&dopt=Abstract&list_uids=27435395|6|Clin Cancer Res|||Jul|2016|JOURNAL ARTICLE
srf	twf1	http://www.ncbi.nlm.nih.gov/entrez/query.fcgi?cmd=Retrieve&db=pubmed&dopt=Abstract&list_uids=27435395|6|Clin Cancer Res|||Jul|2016|JOURNAL ARTICLE
srf	mirn206	http://www.ncbi.nlm.nih.gov/entrez/query.fcgi?cmd=Retrieve&db=pubmed&dopt=Abstract&list_uids=27435395|6|Clin Cancer Res|||Jul|2016|JOURNAL ARTICLE

srf	il11	http://www.ncbi.nlm.nih.gov/entrez/query.fcgi?cmd=Retrieve&db=pubmed&dopt=Abstract&list_uids=27435395|6|Clin Cancer Res|||Jul|2016|JOURNAL ARTICLE
mkl1	twf1	http://www.ncbi.nlm.nih.gov/entrez/query.fcgi?cmd=Retrieve&db=pubmed&dopt=Abstract&list_uids=27435395|6|Clin Cancer Res|||Jul|2016|JOURNAL ARTICLE
mkl1	mirn206	http://www.ncbi.nlm.nih.gov/entrez/query.fcgi?cmd=Retrieve&db=pubmed&dopt=Abstract&list_uids=27435395|6|Clin Cancer Res|||Jul|2016|JOURNAL ARTICLE
mkl1	srf	http://www.ncbi.nlm.nih.gov/entrez/query.fcgi?cmd=Retrieve&db=pubmed&dopt=Abstract&list_uids=27435395|6|Clin Cancer Res|||Jul|2016|JOURNAL ARTICLE
mkl1	il11	http://www.ncbi.nlm.nih.gov/entrez/query.fcgi?cmd=Retrieve&db=pubmed&dopt=Abstract&list_uids=27435395|6|Clin Cancer Res|||Jul|2016|JOURNAL ARTICLE
akr1b1	tam	http://www.ncbi.nlm.nih.gov/entrez/query.fcgi?cmd=Retrieve&db=pubmed&dopt=Abstract&list_uids=27436059|6|Cell Biol Int|||Jul|2016|JOURNAL ARTICLE
rps19	nmt1	http://www.ncbi.nlm.nih.gov/entrez/query.fcgi?cmd=Retrieve&db=pubmed&dopt=Abstract&list_uids=27438140|6|Oncotarget|||Jul|2016|JOURNAL ARTICLE
bpp	mtm1	http://www.ncbi.nlm.nih.gov/entrez/query.fcgi?cmd=Retrieve&db=pubmed&dopt=Abstract&list_uids=27442266|6|PLoS One|11|7||2016|Journal Article
serpinb3	camp	http://www.ncbi.nlm.nih.gov/entrez/query.fcgi?cmd=Retrieve&db=pubmed&dopt=Abstract&list_uids=27588122|6|Oncol Lett|12|3|Sep|2016|JOURNAL ARTICLE
lct	serpinb3	http://www.ncbi.nlm.nih.gov/entrez/query.fcgi?cmd=Retrieve&db=pubmed&dopt=Abstract&list_uids=27446348|6|Exp Ther Med|12|2|Aug|2016|JOURNAL ARTICLE
mtss1	serpinb3	http://www.ncbi.nlm.nih.gov/entrez/query.fcgi?cmd=Retrieve&db=pubmed&dopt=Abstract&list_uids=27446348|6|Exp Ther Med|12|2|Aug|2016|JOURNAL ARTICLE
mtss1	lct	http://www.ncbi.nlm.nih.gov/entrez/query.fcgi?cmd=Retrieve&db=pubmed&dopt=Abstract&list_uids=27446348|6|Exp Ther Med|12|2|Aug|2016|JOURNAL ARTICLE
mtss1	ptk2	http://www.ncbi.nlm.nih.gov/entrez/query.fcgi?cmd=Retrieve&db=pubmed&dopt=Abstract&list_uids=27446348|6|Exp Ther Med|12|2|Aug|2016|JOURNAL ARTICLE
map3k5	mapk14	http://www.ncbi.nlm.nih.gov/entrez/query.fcgi?cmd=Retrieve&db=pubmed&dopt=Abstract&list_uids=27446390|6|Oncol Lett|12|2|Aug|2016|JOURNAL ARTICLE
myd88	mapk14	http://www.ncbi.nlm.nih.gov/entrez/query.fcgi?cmd=Retrieve&db=pubmed&dopt=Abstract&list_uids=27446390|6|Oncol Lett|12|2|Aug|2016|JOURNAL ARTICLE
myd88	map3k5	http://www.ncbi.nlm.nih.gov/entrez/query.fcgi?cmd=Retrieve&db=pubmed&dopt=Abstract&list_uids=27446390|6|Oncol Lett|12|2|Aug|2016|JOURNAL ARTICLE
prex2	pten	http://www.ncbi.nlm.nih.gov/entrez/query.fcgi?cmd=Retrieve&db=pubmed&dopt=Abstract&list_uids=27446408|6|Oncol Lett|12|2|Aug|2016|JOURNAL ARTICLE
prex2	pik3ca	http://www.ncbi.nlm.nih.gov/entrez/query.fcgi?cmd=Retrieve&db=pubmed&dopt=Abstract&list_uids=27446408|6|Oncol Lett|12|2|Aug|2016|JOURNAL ARTICLE

dlc1	hcc	http://www.ncbi.nlm.nih.gov/entrez/query.fcgi?cmd=Retrieve&db=pubmed&dopt=Abstract&list_uids=27446476|6|Oncol Lett|12|2|Aug|2016|JOURNAL ARTICLE
dlc1	rhoa	http://www.ncbi.nlm.nih.gov/entrez/query.fcgi?cmd=Retrieve&db=pubmed&dopt=Abstract&list_uids=27446476|6|Oncol Lett|12|2|Aug|2016|JOURNAL ARTICLE
slurp1	tp53	http://www.ncbi.nlm.nih.gov/entrez/query.fcgi?cmd=Retrieve&db=pubmed&dopt=Abstract&list_uids=27447121|6|Biomed Pharmacother|83||Jul|2016|JOURNAL ARTICLE
cdkn2a	tp53	http://www.ncbi.nlm.nih.gov/entrez/query.fcgi?cmd=Retrieve&db=pubmed&dopt=Abstract&list_uids=27447534|6|J Pathol|||Jul|2016|JOURNAL ARTICLE
braf	pik3ca	http://www.ncbi.nlm.nih.gov/entrez/query.fcgi?cmd=Retrieve&db=pubmed&dopt=Abstract&list_uids=27448973|6|Oncotarget|||Jul|2016|JOURNAL ARTICLE
usp14	uts2r	http://www.ncbi.nlm.nih.gov/entrez/query.fcgi?cmd=Retrieve&db=pubmed&dopt=Abstract&list_uids=27448976|6|Oncotarget|||Jul|2016|JOURNAL ARTICLE
c9orf140	eif2ak3	http://www.ncbi.nlm.nih.gov/entrez/query.fcgi?cmd=Retrieve&db=pubmed&dopt=Abstract&list_uids=27449033|6|Tumour Biol|||Jul|2016|JOURNAL ARTICLE
vav3	wnk1	http://www.ncbi.nlm.nih.gov/entrez/query.fcgi?cmd=Retrieve&db=pubmed&dopt=Abstract&list_uids=27453460|6|Pancreatology|||Jul|2016|JOURNAL ARTICLE
vav3	akt1	http://www.ncbi.nlm.nih.gov/entrez/query.fcgi?cmd=Retrieve&db=pubmed&dopt=Abstract&list_uids=27453460|6|Pancreatology|||Jul|2016|JOURNAL ARTICLE
vav3	rac1	http://www.ncbi.nlm.nih.gov/entrez/query.fcgi?cmd=Retrieve&db=pubmed&dopt=Abstract&list_uids=27453460|6|Pancreatology|||Jul|2016|JOURNAL ARTICLE
mirn34a	hmgb1	http://www.ncbi.nlm.nih.gov/entrez/query.fcgi?cmd=Retrieve&db=pubmed&dopt=Abstract&list_uids=27456356|6|Tumour Biol|||Jul|2016|JOURNAL ARTICLE
sox9	tgfb	http://www.ncbi.nlm.nih.gov/entrez/query.fcgi?cmd=Retrieve&db=pubmed&dopt=Abstract&list_uids=27457505|6|Sci Rep|6|||2016|Journal Article
sox9	itk	http://www.ncbi.nlm.nih.gov/entrez/query.fcgi?cmd=Retrieve&db=pubmed&dopt=Abstract&list_uids=27457505|6|Sci Rep|6|||2016|Journal Article
hcfc1r1	akt1	http://www.ncbi.nlm.nih.gov/entrez/query.fcgi?cmd=Retrieve&db=pubmed&dopt=Abstract&list_uids=27458096|6|Oncol Res|24|3||2016|Journal Article
hcfc1r1	pik3ca	http://www.ncbi.nlm.nih.gov/entrez/query.fcgi?cmd=Retrieve&db=pubmed&dopt=Abstract&list_uids=27458096|6|Oncol Res|24|3||2016|Journal Article
pftk1	myc	http://www.ncbi.nlm.nih.gov/entrez/query.fcgi?cmd=Retrieve&db=pubmed&dopt=Abstract&list_uids=27458099|6|Oncol Res|24|3||2016|Journal Article
tsc2	ephb2	http://www.ncbi.nlm.nih.gov/entrez/query.fcgi?cmd=Retrieve&db=pubmed&dopt=Abstract&list_uids=27458154|6|Oncotarget|||Jul|2016|JOURNAL ARTICLE
capza1	src	http://www.ncbi.nlm.nih.gov/entrez/query.fcgi?cmd=Retrieve&db=pubmed&dopt=Abstract&list_uids=27458171|6|Oncotarget|||Jul|2016|JOURNAL ARTICLE

capza1	jak1	http://www.ncbi.nlm.nih.gov/entrez/query.fcgi?cmd=Retrieve&db=pubmed&dopt=Abstract&list_uids=27458171|6|Oncotarget|||Jul|2016|JOURNAL ARTICLE
capza1	stat3	http://www.ncbi.nlm.nih.gov/entrez/query.fcgi?cmd=Retrieve&db=pubmed&dopt=Abstract&list_uids=27458171|6|Oncotarget|||Jul|2016|JOURNAL ARTICLE
id1	hcc	http://www.ncbi.nlm.nih.gov/entrez/query.fcgi?cmd=Retrieve&db=pubmed&dopt=Abstract&list_uids=27459117|6|Biomed Pharmacother|83||Jul|2016|JOURNAL ARTICLE
id1	sgcb	http://www.ncbi.nlm.nih.gov/entrez/query.fcgi?cmd=Retrieve&db=pubmed&dopt=Abstract&list_uids=27466488|6|Anticancer Res|36|8|Aug|2016|Journal Article
hbe1	nqo1	http://www.ncbi.nlm.nih.gov/entrez/query.fcgi?cmd=Retrieve&db=pubmed&dopt=Abstract&list_uids=27459794|6|Wei Sheng Yan Jiu|45|3|May|2016|English Abstract,Journal Article
hmox1	nqo1	http://www.ncbi.nlm.nih.gov/entrez/query.fcgi?cmd=Retrieve&db=pubmed&dopt=Abstract&list_uids=27459794|6|Wei Sheng Yan Jiu|45|3|May|2016|English Abstract,Journal Article
hmox1	hbe1	http://www.ncbi.nlm.nih.gov/entrez/query.fcgi?cmd=Retrieve&db=pubmed&dopt=Abstract&list_uids=27459794|6|Wei Sheng Yan Jiu|45|3|May|2016|English Abstract,Journal Article
hmox1	gabpa	http://www.ncbi.nlm.nih.gov/entrez/query.fcgi?cmd=Retrieve&db=pubmed&dopt=Abstract&list_uids=27459794|6|Wei Sheng Yan Jiu|45|3|May|2016|English Abstract,Journal Article
rplp0p2	dld	http://www.ncbi.nlm.nih.gov/entrez/query.fcgi?cmd=Retrieve&db=pubmed&dopt=Abstract&list_uids=27460542|6|Oncol Rep|36|3|Sep|2016|Journal Article
mirn33a	uts2r	http://www.ncbi.nlm.nih.gov/entrez/query.fcgi?cmd=Retrieve&db=pubmed&dopt=Abstract&list_uids=27460728|6|Eur Rev Med Pharmacol Sci|20|14|Jul|2016|Journal Article
mirn33a	twist1	http://www.ncbi.nlm.nih.gov/entrez/query.fcgi?cmd=Retrieve&db=pubmed&dopt=Abstract&list_uids=27460728|6|Eur Rev Med Pharmacol Sci|20|14|Jul|2016|Journal Article
glipr1	gde1	http://www.ncbi.nlm.nih.gov/entrez/query.fcgi?cmd=Retrieve&db=pubmed&dopt=Abstract&list_uids=27460987|6|Oncol Rep|36|3|Sep|2016|Journal Article
mecp2	cldn6	http://www.ncbi.nlm.nih.gov/entrez/query.fcgi?cmd=Retrieve&db=pubmed&dopt=Abstract&list_uids=27461117|6|J Exp Clin Cancer Res|35|1||2016|Journal Article
cldn6	prdx2	http://www.ncbi.nlm.nih.gov/entrez/query.fcgi?cmd=Retrieve&db=pubmed&dopt=Abstract&list_uids=27461117|6|J Exp Clin Cancer Res|35|1||2016|Journal Article
prdx2	mecp2	http://www.ncbi.nlm.nih.gov/entrez/query.fcgi?cmd=Retrieve&db=pubmed&dopt=Abstract&list_uids=27461117|6|J Exp Clin Cancer Res|35|1||2016|Journal Article
msmb	cldn6	http://www.ncbi.nlm.nih.gov/entrez/query.fcgi?cmd=Retrieve&db=pubmed&dopt=Abstract&list_uids=27461117|6|J Exp Clin Cancer Res|35|1||2016|Journal Article
mirn451	hcc	http://www.ncbi.nlm.nih.gov/entrez/query.fcgi?cmd=Retrieve&db=pubmed&dopt=Abstract&list_uids=27461244|6|Oncol Rep|36|3|Sep|2016|Journal Article
wwp2	atp2c1	http://www.ncbi.nlm.nih.gov/entrez/query.fcgi?cmd=Retrieve&db=pubmed&dopt=Abstract&list_uids=27462019|6|Biochem Biophys Res Commun|||Jul|2016|JOURNAL ARTICLE

s100a8	hcc	http://www.ncbi.nlm.nih.gov/entrez/query.fcgi?cmd=Retrieve&db=pubmed&dopt=Abstract&list_uids=27462864|6|Oncotarget|||Jul|2016|JOURNAL ARTICLE
socs2	hcc	http://www.ncbi.nlm.nih.gov/entrez/query.fcgi?cmd=Retrieve&db=pubmed&dopt=Abstract&list_uids=27465557|6|Tumour Biol|||Jul|2016|JOURNAL ARTICLE
sgcb	jag1	http://www.ncbi.nlm.nih.gov/entrez/query.fcgi?cmd=Retrieve&db=pubmed&dopt=Abstract&list_uids=27542217|6|Oncotarget|||Aug|2016|JOURNAL ARTICLE
tdgf1	rln2	http://www.ncbi.nlm.nih.gov/entrez/query.fcgi?cmd=Retrieve&db=pubmed&dopt=Abstract&list_uids=27467944|6|Oncoimmunology|5|5|May|2016|Journal Article
tdgf1	cd8a	http://www.ncbi.nlm.nih.gov/entrez/query.fcgi?cmd=Retrieve&db=pubmed&dopt=Abstract&list_uids=27467944|6|Oncoimmunology|5|5|May|2016|Journal Article
itgb2	itgam	http://www.ncbi.nlm.nih.gov/entrez/query.fcgi?cmd=Retrieve&db=pubmed&dopt=Abstract&list_uids=27468683|6|Microbiol Immunol|||Jul|2016|JOURNAL ARTICLE
itgal	itgam	http://www.ncbi.nlm.nih.gov/entrez/query.fcgi?cmd=Retrieve&db=pubmed&dopt=Abstract&list_uids=27468683|6|Microbiol Immunol|||Jul|2016|JOURNAL ARTICLE
itgal	itgb2	http://www.ncbi.nlm.nih.gov/entrez/query.fcgi?cmd=Retrieve&db=pubmed&dopt=Abstract&list_uids=27468683|6|Microbiol Immunol|||Jul|2016|JOURNAL ARTICLE
plg	nhs	http://www.ncbi.nlm.nih.gov/entrez/query.fcgi?cmd=Retrieve&db=pubmed&dopt=Abstract&list_uids=27468683|6|Microbiol Immunol|||Jul|2016|JOURNAL ARTICLE
mirn433	notch1	http://www.ncbi.nlm.nih.gov/entrez/query.fcgi?cmd=Retrieve&db=pubmed&dopt=Abstract&list_uids=27468873|6|Neoplasma|63|5|Jul|2016|JOURNAL ARTICLE
cbl	grb2	http://www.ncbi.nlm.nih.gov/entrez/query.fcgi?cmd=Retrieve&db=pubmed&dopt=Abstract&list_uids=27472394|6|Oncotarget|||Jul|2016|JOURNAL ARTICLE
cbl	src	http://www.ncbi.nlm.nih.gov/entrez/query.fcgi?cmd=Retrieve&db=pubmed&dopt=Abstract&list_uids=27472394|6|Oncotarget|||Jul|2016|JOURNAL ARTICLE
cbl	ptk2	http://www.ncbi.nlm.nih.gov/entrez/query.fcgi?cmd=Retrieve&db=pubmed&dopt=Abstract&list_uids=27472394|6|Oncotarget|||Jul|2016|JOURNAL ARTICLE
mirn122	tp53	http://www.ncbi.nlm.nih.gov/entrez/query.fcgi?cmd=Retrieve&db=pubmed&dopt=Abstract&list_uids=27472451|6|Med Sci Monit|22|||2016|Journal Article
mirn122	uts2r	http://www.ncbi.nlm.nih.gov/entrez/query.fcgi?cmd=Retrieve&db=pubmed&dopt=Abstract&list_uids=27508026|6|Am J Transl Res|8|7||2016|Journal Article
mirn122	vegfc	http://www.ncbi.nlm.nih.gov/entrez/query.fcgi?cmd=Retrieve&db=pubmed&dopt=Abstract&list_uids=27508026|6|Am J Transl Res|8|7||2016|Journal Article
rbpj	gsc	http://www.ncbi.nlm.nih.gov/entrez/query.fcgi?cmd=Retrieve&db=pubmed&dopt=Abstract&list_uids=27472461|6|Oncotarget|||Jul|2016|JOURNAL ARTICLE
sdcbp	dll1	http://www.ncbi.nlm.nih.gov/entrez/query.fcgi?cmd=Retrieve&db=pubmed&dopt=Abstract&list_uids=27472461|6|Oncotarget|||Jul|2016|JOURNAL ARTICLE

sdcbp	notch1	http://www.ncbi.nlm.nih.gov/entrez/query.fcgi?cmd=Retrieve&db=pubmed&dopt=Abstract&list_uids=27472461|6|Oncotarget|||Jul|2016|JOURNAL ARTICLE
sdcbp	stat3	http://www.ncbi.nlm.nih.gov/entrez/query.fcgi?cmd=Retrieve&db=pubmed&dopt=Abstract&list_uids=27472461|6|Oncotarget|||Jul|2016|JOURNAL ARTICLE
sdcbp	sox2	http://www.ncbi.nlm.nih.gov/entrez/query.fcgi?cmd=Retrieve&db=pubmed&dopt=Abstract&list_uids=27472461|6|Oncotarget|||Jul|2016|JOURNAL ARTICLE
sdcbp	pou5f1	http://www.ncbi.nlm.nih.gov/entrez/query.fcgi?cmd=Retrieve&db=pubmed&dopt=Abstract&list_uids=27472461|6|Oncotarget|||Jul|2016|JOURNAL ARTICLE
sdcbp	nanog	http://www.ncbi.nlm.nih.gov/entrez/query.fcgi?cmd=Retrieve&db=pubmed&dopt=Abstract&list_uids=27472461|6|Oncotarget|||Jul|2016|JOURNAL ARTICLE
nanog	stat3	http://www.ncbi.nlm.nih.gov/entrez/query.fcgi?cmd=Retrieve&db=pubmed&dopt=Abstract&list_uids=27472461|6|Oncotarget|||Jul|2016|JOURNAL ARTICLE
nanog	sox2	http://www.ncbi.nlm.nih.gov/entrez/query.fcgi?cmd=Retrieve&db=pubmed&dopt=Abstract&list_uids=27472461|6|Oncotarget|||Jul|2016|JOURNAL ARTICLE
nanog	pou5f1	http://www.ncbi.nlm.nih.gov/entrez/query.fcgi?cmd=Retrieve&db=pubmed&dopt=Abstract&list_uids=27472461|6|Oncotarget|||Jul|2016|JOURNAL ARTICLE
nanog	stat6	http://www.ncbi.nlm.nih.gov/entrez/query.fcgi?cmd=Retrieve&db=pubmed&dopt=Abstract&list_uids=27533463|6|Oncotarget|||Aug|2016|JOURNAL ARTICLE
nanog	alcam	http://www.ncbi.nlm.nih.gov/entrez/query.fcgi?cmd=Retrieve&db=pubmed&dopt=Abstract&list_uids=27533463|6|Oncotarget|||Aug|2016|JOURNAL ARTICLE
nanog	prom1	http://www.ncbi.nlm.nih.gov/entrez/query.fcgi?cmd=Retrieve&db=pubmed&dopt=Abstract&list_uids=27533463|6|Oncotarget|||Aug|2016|JOURNAL ARTICLE
nanog	cd44	http://www.ncbi.nlm.nih.gov/entrez/query.fcgi?cmd=Retrieve&db=pubmed&dopt=Abstract&list_uids=27533463|6|Oncotarget|||Aug|2016|JOURNAL ARTICLE
smad7	ski	http://www.ncbi.nlm.nih.gov/entrez/query.fcgi?cmd=Retrieve&db=pubmed&dopt=Abstract&list_uids=27473823|6|Cancer Lett|381|1|Oct|2016|Journal Article
smad7	snord87	http://www.ncbi.nlm.nih.gov/entrez/query.fcgi?cmd=Retrieve&db=pubmed&dopt=Abstract&list_uids=27574009|6|Oncol Rep|||Aug|2016|JOURNAL ARTICLE
2.6.1.77	mapk14	http://www.ncbi.nlm.nih.gov/entrez/query.fcgi?cmd=Retrieve&db=pubmed&dopt=Abstract&list_uids=27476935|6|Biol Pharm Bull|39|8||2016|Journal Article
2.6.1.77	ephb2	http://www.ncbi.nlm.nih.gov/entrez/query.fcgi?cmd=Retrieve&db=pubmed&dopt=Abstract&list_uids=27476935|6|Biol Pharm Bull|39|8||2016|Journal Article
tesc	itk	http://www.ncbi.nlm.nih.gov/entrez/query.fcgi?cmd=Retrieve&db=pubmed&dopt=Abstract&list_uids=27485112|6|Tumour Biol|||Aug|2016|JOURNAL ARTICLE
nr0b2	rela	http://www.ncbi.nlm.nih.gov/entrez/query.fcgi?cmd=Retrieve&db=pubmed&dopt=Abstract&list_uids=27486225|6|Mol Cancer Ther|||Aug|2016|JOURNAL ARTICLE

nr0b2	ccl2	http://www.ncbi.nlm.nih.gov/entrez/query.fcgi?cmd=Retrieve&db=pubmed&dopt=Abstract&list_uids=27486225|6|Mol Cancer Ther|||Aug|2016|JOURNAL ARTICLE
nr0b2	hcc	http://www.ncbi.nlm.nih.gov/entrez/query.fcgi?cmd=Retrieve&db=pubmed&dopt=Abstract&list_uids=27486225|6|Mol Cancer Ther|||Aug|2016|JOURNAL ARTICLE
spock1	pcsk1	http://www.ncbi.nlm.nih.gov/entrez/query.fcgi?cmd=Retrieve&db=pubmed&dopt=Abstract&list_uids=27486308|6|Drug Des Devel Ther|10|||2016|Journal Article
tyro3	mertk	http://www.ncbi.nlm.nih.gov/entrez/query.fcgi?cmd=Retrieve&db=pubmed&dopt=Abstract&list_uids=27486820|6|Oncotarget|||Jul|2016|JOURNAL ARTICLE
c16orf74	ppp3ca	http://www.ncbi.nlm.nih.gov/entrez/query.fcgi?cmd=Retrieve&db=pubmed&dopt=Abstract&list_uids=27486884|6|Oncotarget|||Jul|2016|JOURNAL ARTICLE
ctcf	suz12	http://www.ncbi.nlm.nih.gov/entrez/query.fcgi?cmd=Retrieve&db=pubmed&dopt=Abstract&list_uids=27486969|6|Oncotarget|||Jul|2016|JOURNAL ARTICLE
igf2	suz12	http://www.ncbi.nlm.nih.gov/entrez/query.fcgi?cmd=Retrieve&db=pubmed&dopt=Abstract&list_uids=27486969|6|Oncotarget|||Jul|2016|JOURNAL ARTICLE
igf2	ctcf	http://www.ncbi.nlm.nih.gov/entrez/query.fcgi?cmd=Retrieve&db=pubmed&dopt=Abstract&list_uids=27486969|6|Oncotarget|||Jul|2016|JOURNAL ARTICLE
iqgap1	ptk2	http://www.ncbi.nlm.nih.gov/entrez/query.fcgi?cmd=Retrieve&db=pubmed&dopt=Abstract&list_uids=27486972|6|Oncotarget|||Jul|2016|JOURNAL ARTICLE
npc1	itk	http://www.ncbi.nlm.nih.gov/entrez/query.fcgi?cmd=Retrieve&db=pubmed&dopt=Abstract&list_uids=27584792|6|Cell Death Dis|7|9||2016|Journal Article
pla2g7	npc1	http://www.ncbi.nlm.nih.gov/entrez/query.fcgi?cmd=Retrieve&db=pubmed&dopt=Abstract&list_uids=27487154|6|Oncotarget|||Aug|2016|JOURNAL ARTICLE
tlr10	slc6a4	http://www.ncbi.nlm.nih.gov/entrez/query.fcgi?cmd=Retrieve&db=pubmed&dopt=Abstract&list_uids=27488594|6|Microb Ecol|||Aug|2016|JOURNAL ARTICLE
nek3	mapk3	http://www.ncbi.nlm.nih.gov/entrez/query.fcgi?cmd=Retrieve&db=pubmed&dopt=Abstract&list_uids=27489110|6|J Biol Chem|||Aug|2016|JOURNAL ARTICLE
nek3	actin	http://www.ncbi.nlm.nih.gov/entrez/query.fcgi?cmd=Retrieve&db=pubmed&dopt=Abstract&list_uids=27489110|6|J Biol Chem|||Aug|2016|JOURNAL ARTICLE
prl	actin	http://www.ncbi.nlm.nih.gov/entrez/query.fcgi?cmd=Retrieve&db=pubmed&dopt=Abstract&list_uids=27489110|6|J Biol Chem|||Aug|2016|JOURNAL ARTICLE
prl	nek3	http://www.ncbi.nlm.nih.gov/entrez/query.fcgi?cmd=Retrieve&db=pubmed&dopt=Abstract&list_uids=27489110|6|J Biol Chem|||Aug|2016|JOURNAL ARTICLE
cd38	cd34	http://www.ncbi.nlm.nih.gov/entrez/query.fcgi?cmd=Retrieve&db=pubmed&dopt=Abstract&list_uids=27489587|6|Int J Hematol Oncol Stem Cell Res|10|3|Jul|2016|Journal Article
anxa4	rela	http://www.ncbi.nlm.nih.gov/entrez/query.fcgi?cmd=Retrieve&db=pubmed&dopt=Abstract&list_uids=27491820|6|Sci Rep|6|||2016|Journal Article

mmp8	mmp13	http://www.ncbi.nlm.nih.gov/entrez/query.fcgi?cmd=Retrieve&db=pubmed&dopt=Abstract&list_uids=27491937|6|Anticancer Agents Med Chem|||Aug|2016|JOURNAL ARTICLE
cyr61	itk	http://www.ncbi.nlm.nih.gov/entrez/query.fcgi?cmd=Retrieve&db=pubmed&dopt=Abstract&list_uids=27492459|6|Tumour Biol|||Aug|2016|JOURNAL ARTICLE
cd24	vegfa	http://www.ncbi.nlm.nih.gov/entrez/query.fcgi?cmd=Retrieve&db=pubmed&dopt=Abstract&list_uids=27494878|6|Oncotarget|||Aug|2016|JOURNAL ARTICLE
cd24	stat3	http://www.ncbi.nlm.nih.gov/entrez/query.fcgi?cmd=Retrieve&db=pubmed&dopt=Abstract&list_uids=27494878|6|Oncotarget|||Aug|2016|JOURNAL ARTICLE
cd24	hsp90aa1	http://www.ncbi.nlm.nih.gov/entrez/query.fcgi?cmd=Retrieve&db=pubmed&dopt=Abstract&list_uids=27494878|6|Oncotarget|||Aug|2016|JOURNAL ARTICLE
nf1	mirn10b	http://www.ncbi.nlm.nih.gov/entrez/query.fcgi?cmd=Retrieve&db=pubmed&dopt=Abstract&list_uids=27494896|6|Oncotarget|||Aug|2016|JOURNAL ARTICLE
nf1	rhoc	http://www.ncbi.nlm.nih.gov/entrez/query.fcgi?cmd=Retrieve&db=pubmed&dopt=Abstract&list_uids=27494896|6|Oncotarget|||Aug|2016|JOURNAL ARTICLE
rhoc	mirn10b	http://www.ncbi.nlm.nih.gov/entrez/query.fcgi?cmd=Retrieve&db=pubmed&dopt=Abstract&list_uids=27494896|6|Oncotarget|||Aug|2016|JOURNAL ARTICLE
mirn10b	jun	http://www.ncbi.nlm.nih.gov/entrez/query.fcgi?cmd=Retrieve&db=pubmed&dopt=Abstract&list_uids=27494896|6|Oncotarget|||Aug|2016|JOURNAL ARTICLE
mirn10b	pten	http://www.ncbi.nlm.nih.gov/entrez/query.fcgi?cmd=Retrieve&db=pubmed&dopt=Abstract&list_uids=27569213|6|Cancer Res|||Aug|2016|JOURNAL ARTICLE
mirn10b	dyrk1a	http://www.ncbi.nlm.nih.gov/entrez/query.fcgi?cmd=Retrieve&db=pubmed&dopt=Abstract&list_uids=27569213|6|Cancer Res|||Aug|2016|JOURNAL ARTICLE
mirn10b	tbx5	http://www.ncbi.nlm.nih.gov/entrez/query.fcgi?cmd=Retrieve&db=pubmed&dopt=Abstract&list_uids=27569213|6|Cancer Res|||Aug|2016|JOURNAL ARTICLE
rcbtb1	glp-1	http://www.ncbi.nlm.nih.gov/entrez/query.fcgi?cmd=Retrieve&db=pubmed&dopt=Abstract&list_uids=27496641|6|Mol Cell Endocrinol|436||Aug|2016|JOURNAL ARTICLE
mirn140	adam9	http://www.ncbi.nlm.nih.gov/entrez/query.fcgi?cmd=Retrieve&db=pubmed&dopt=Abstract&list_uids=27498787|6|Oncol Rep|||Aug|2016|JOURNAL ARTICLE
map3k2	mirn186	http://www.ncbi.nlm.nih.gov/entrez/query.fcgi?cmd=Retrieve&db=pubmed&dopt=Abstract&list_uids=27498924|6|Int J Oncol|||Jul|2016|JOURNAL ARTICLE
mirn107	akt1	http://www.ncbi.nlm.nih.gov/entrez/query.fcgi?cmd=Retrieve&db=pubmed&dopt=Abstract&list_uids=27498977|6|Int J Oncol|||Jul|2016|JOURNAL ARTICLE
mirn107	pik3ca	http://www.ncbi.nlm.nih.gov/entrez/query.fcgi?cmd=Retrieve&db=pubmed&dopt=Abstract&list_uids=27498977|6|Int J Oncol|||Jul|2016|JOURNAL ARTICLE
bdnf	mirn107	http://www.ncbi.nlm.nih.gov/entrez/query.fcgi?cmd=Retrieve&db=pubmed&dopt=Abstract&list_uids=27498977|6|Int J Oncol|||Jul|2016|JOURNAL ARTICLE

bdnf	akt1	http://www.ncbi.nlm.nih.gov/entrez/query.fcgi?cmd=Retrieve&db=pubmed&dopt=Abstract&list_uids=27498977|6|Int J Oncol|||Jul|2016|JOURNAL ARTICLE
bdnf	pik3ca	http://www.ncbi.nlm.nih.gov/entrez/query.fcgi?cmd=Retrieve&db=pubmed&dopt=Abstract&list_uids=27498977|6|Int J Oncol|||Jul|2016|JOURNAL ARTICLE
mdm2	tp53	http://www.ncbi.nlm.nih.gov/entrez/query.fcgi?cmd=Retrieve&db=pubmed&dopt=Abstract&list_uids=27499479|6|Int J Oncol|||Aug|2016|JOURNAL ARTICLE
mdm2	frap1	http://www.ncbi.nlm.nih.gov/entrez/query.fcgi?cmd=Retrieve&db=pubmed&dopt=Abstract&list_uids=27499479|6|Int J Oncol|||Aug|2016|JOURNAL ARTICLE
pdgfd	itk	http://www.ncbi.nlm.nih.gov/entrez/query.fcgi?cmd=Retrieve&db=pubmed&dopt=Abstract&list_uids=27507215|6|Biochem Biophys Res Commun|478|2|Sep|2016|Journal Article
pdgfd	ephb2	http://www.ncbi.nlm.nih.gov/entrez/query.fcgi?cmd=Retrieve&db=pubmed&dopt=Abstract&list_uids=27507215|6|Biochem Biophys Res Commun|478|2|Sep|2016|Journal Article
pdgfd	akt1	http://www.ncbi.nlm.nih.gov/entrez/query.fcgi?cmd=Retrieve&db=pubmed&dopt=Abstract&list_uids=27507215|6|Biochem Biophys Res Commun|478|2|Sep|2016|Journal Article
pdgfd	mapk14	http://www.ncbi.nlm.nih.gov/entrez/query.fcgi?cmd=Retrieve&db=pubmed&dopt=Abstract&list_uids=27507215|6|Biochem Biophys Res Commun|478|2|Sep|2016|Journal Article
muc3a	muc13	http://www.ncbi.nlm.nih.gov/entrez/query.fcgi?cmd=Retrieve&db=pubmed&dopt=Abstract&list_uids=27507554|6|J Gastrointest Surg|||Aug|2016|JOURNAL ARTICLE
lect2	akt1	http://www.ncbi.nlm.nih.gov/entrez/query.fcgi?cmd=Retrieve&db=pubmed&dopt=Abstract&list_uids=27507763|6|Sci Rep|6|||2016|Journal Article
lect2	vegfa	http://www.ncbi.nlm.nih.gov/entrez/query.fcgi?cmd=Retrieve&db=pubmed&dopt=Abstract&list_uids=27507763|6|Sci Rep|6|||2016|Journal Article
lect2	hcc	http://www.ncbi.nlm.nih.gov/entrez/query.fcgi?cmd=Retrieve&db=pubmed&dopt=Abstract&list_uids=27507763|6|Sci Rep|6|||2016|Journal Article
vegfc	uts2r	http://www.ncbi.nlm.nih.gov/entrez/query.fcgi?cmd=Retrieve&db=pubmed&dopt=Abstract&list_uids=27508026|6|Am J Transl Res|8|7||2016|Journal Article
ppa1	hcc	http://www.ncbi.nlm.nih.gov/entrez/query.fcgi?cmd=Retrieve&db=pubmed&dopt=Abstract&list_uids=27509921|6|Oncol Rep|||Aug|2016|JOURNAL ARTICLE
ppa1	akt1	http://www.ncbi.nlm.nih.gov/entrez/query.fcgi?cmd=Retrieve&db=pubmed&dopt=Abstract&list_uids=27509921|6|Oncol Rep|||Aug|2016|JOURNAL ARTICLE
nek2	hcc	http://www.ncbi.nlm.nih.gov/entrez/query.fcgi?cmd=Retrieve&db=pubmed&dopt=Abstract&list_uids=27509921|6|Oncol Rep|||Aug|2016|JOURNAL ARTICLE
nek2	akt1	http://www.ncbi.nlm.nih.gov/entrez/query.fcgi?cmd=Retrieve&db=pubmed&dopt=Abstract&list_uids=27509921|6|Oncol Rep|||Aug|2016|JOURNAL ARTICLE
nek2	ppa1	http://www.ncbi.nlm.nih.gov/entrez/query.fcgi?cmd=Retrieve&db=pubmed&dopt=Abstract&list_uids=27509921|6|Oncol Rep|||Aug|2016|JOURNAL ARTICLE

il6r	il6	http://www.ncbi.nlm.nih.gov/entrez/query.fcgi?cmd=Retrieve&db=pubmed&dopt=Abstract&list_uids=27510094|6|Oncol Rep|||Aug|2016|JOURNAL ARTICLE
mfi2	foxp4	http://www.ncbi.nlm.nih.gov/entrez/query.fcgi?cmd=Retrieve&db=pubmed&dopt=Abstract&list_uids=27513470|6|Oncol Rep|||Aug|2016|JOURNAL ARTICLE
vcan	il10	http://www.ncbi.nlm.nih.gov/entrez/query.fcgi?cmd=Retrieve&db=pubmed&dopt=Abstract&list_uids=27513864|6|PLoS One|11|8||2016|Journal Article
cd80	il10	http://www.ncbi.nlm.nih.gov/entrez/query.fcgi?cmd=Retrieve&db=pubmed&dopt=Abstract&list_uids=27513864|6|PLoS One|11|8||2016|Journal Article
cd80	vcan	http://www.ncbi.nlm.nih.gov/entrez/query.fcgi?cmd=Retrieve&db=pubmed&dopt=Abstract&list_uids=27513864|6|PLoS One|11|8||2016|Journal Article
il1b	il10	http://www.ncbi.nlm.nih.gov/entrez/query.fcgi?cmd=Retrieve&db=pubmed&dopt=Abstract&list_uids=27513864|6|PLoS One|11|8||2016|Journal Article
il1b	vcan	http://www.ncbi.nlm.nih.gov/entrez/query.fcgi?cmd=Retrieve&db=pubmed&dopt=Abstract&list_uids=27513864|6|PLoS One|11|8||2016|Journal Article
il1b	cd80	http://www.ncbi.nlm.nih.gov/entrez/query.fcgi?cmd=Retrieve&db=pubmed&dopt=Abstract&list_uids=27513864|6|PLoS One|11|8||2016|Journal Article
il1b	il8	http://www.ncbi.nlm.nih.gov/entrez/query.fcgi?cmd=Retrieve&db=pubmed&dopt=Abstract&list_uids=27513864|6|PLoS One|11|8||2016|Journal Article
mirn126	cxcr4	http://www.ncbi.nlm.nih.gov/entrez/query.fcgi?cmd=Retrieve&db=pubmed&dopt=Abstract&list_uids=27517626|6|Oncotarget|||Aug|2016|JOURNAL ARTICLE
mirn126	rhoa	http://www.ncbi.nlm.nih.gov/entrez/query.fcgi?cmd=Retrieve&db=pubmed&dopt=Abstract&list_uids=27517626|6|Oncotarget|||Aug|2016|JOURNAL ARTICLE
mirn126	uts2r	http://www.ncbi.nlm.nih.gov/entrez/query.fcgi?cmd=Retrieve&db=pubmed&dopt=Abstract&list_uids=27578985|6|Onco Targets Ther|9|||2016|Journal Article
mirn126	mirn216a	http://www.ncbi.nlm.nih.gov/entrez/query.fcgi?cmd=Retrieve&db=pubmed&dopt=Abstract&list_uids=27578985|6|Onco Targets Ther|9|||2016|Journal Article
mirn126	akt1	http://www.ncbi.nlm.nih.gov/entrez/query.fcgi?cmd=Retrieve&db=pubmed&dopt=Abstract&list_uids=27578985|6|Onco Targets Ther|9|||2016|Journal Article
mirn126	pik3ca	http://www.ncbi.nlm.nih.gov/entrez/query.fcgi?cmd=Retrieve&db=pubmed&dopt=Abstract&list_uids=27578985|6|Onco Targets Ther|9|||2016|Journal Article
mirn126	pik3r2	http://www.ncbi.nlm.nih.gov/entrez/query.fcgi?cmd=Retrieve&db=pubmed&dopt=Abstract&list_uids=27578985|6|Onco Targets Ther|9|||2016|Journal Article
mirn126	rfxank	http://www.ncbi.nlm.nih.gov/entrez/query.fcgi?cmd=Retrieve&db=pubmed&dopt=Abstract&list_uids=27578985|6|Onco Targets Ther|9|||2016|Journal Article
mirn126	gata4	http://www.ncbi.nlm.nih.gov/entrez/query.fcgi?cmd=Retrieve&db=pubmed&dopt=Abstract&list_uids=27598297|6|Artif Cells Nanomed Biotechnol|||Sep|2016|JOURNAL ARTICLE

smad3	mapk14	http://www.ncbi.nlm.nih.gov/entrez/query.fcgi?cmd=Retrieve&db=pubmed&dopt=Abstract&list_uids=27519288|6|Toxicology|366-367||Aug|2016|JOURNAL ARTICLE
smad3	mapk8	http://www.ncbi.nlm.nih.gov/entrez/query.fcgi?cmd=Retrieve&db=pubmed&dopt=Abstract&list_uids=27519288|6|Toxicology|366-367||Aug|2016|JOURNAL ARTICLE
ahr	smad3	http://www.ncbi.nlm.nih.gov/entrez/query.fcgi?cmd=Retrieve&db=pubmed&dopt=Abstract&list_uids=27519288|6|Toxicology|366-367||Aug|2016|JOURNAL ARTICLE
ahr	cyp1a1	http://www.ncbi.nlm.nih.gov/entrez/query.fcgi?cmd=Retrieve&db=pubmed&dopt=Abstract&list_uids=27519288|6|Toxicology|366-367||Aug|2016|JOURNAL ARTICLE
ahr	ephb2	http://www.ncbi.nlm.nih.gov/entrez/query.fcgi?cmd=Retrieve&db=pubmed&dopt=Abstract&list_uids=27601158|6|Int J Oncol|||Aug|2016|JOURNAL ARTICLE
mirnlet7b	e2f2	http://www.ncbi.nlm.nih.gov/entrez/query.fcgi?cmd=Retrieve&db=pubmed&dopt=Abstract&list_uids=27520092|6|J Physiol Biochem|||Aug|2016|JOURNAL ARTICLE
dclk1	zeb1	http://www.ncbi.nlm.nih.gov/entrez/query.fcgi?cmd=Retrieve&db=pubmed&dopt=Abstract&list_uids=27520310|6|J Cancer Res Clin Oncol|142|10|Oct|2016|Journal Article
dclk1	itk	http://www.ncbi.nlm.nih.gov/entrez/query.fcgi?cmd=Retrieve&db=pubmed&dopt=Abstract&list_uids=27520310|6|J Cancer Res Clin Oncol|142|10|Oct|2016|Journal Article
mirn132	ttk	http://www.ncbi.nlm.nih.gov/entrez/query.fcgi?cmd=Retrieve&db=pubmed&dopt=Abstract&list_uids=27522003|6|Biomed Pharmacother|83||Aug|2016|JOURNAL ARTICLE
mirn132	itk	http://www.ncbi.nlm.nih.gov/entrez/query.fcgi?cmd=Retrieve&db=pubmed&dopt=Abstract&list_uids=27527117|6|Prostate|||Aug|2016|JOURNAL ARTICLE
hlf	ttk	http://www.ncbi.nlm.nih.gov/entrez/query.fcgi?cmd=Retrieve&db=pubmed&dopt=Abstract&list_uids=27522003|6|Biomed Pharmacother|83||Aug|2016|JOURNAL ARTICLE
hlf	mirn132	http://www.ncbi.nlm.nih.gov/entrez/query.fcgi?cmd=Retrieve&db=pubmed&dopt=Abstract&list_uids=27522003|6|Biomed Pharmacother|83||Aug|2016|JOURNAL ARTICLE
foxa1	fos	http://www.ncbi.nlm.nih.gov/entrez/query.fcgi?cmd=Retrieve&db=pubmed&dopt=Abstract&list_uids=27524420|6|Oncogene|||Aug|2016|JOURNAL ARTICLE
foxa1	ervk2	http://www.ncbi.nlm.nih.gov/entrez/query.fcgi?cmd=Retrieve&db=pubmed&dopt=Abstract&list_uids=27524420|6|Oncogene|||Aug|2016|JOURNAL ARTICLE
foxa1	itk	http://www.ncbi.nlm.nih.gov/entrez/query.fcgi?cmd=Retrieve&db=pubmed&dopt=Abstract&list_uids=27524420|6|Oncogene|||Aug|2016|JOURNAL ARTICLE
foxa1	akap13	http://www.ncbi.nlm.nih.gov/entrez/query.fcgi?cmd=Retrieve&db=pubmed&dopt=Abstract&list_uids=27524420|6|Oncogene|||Aug|2016|JOURNAL ARTICLE
akap13	itk	http://www.ncbi.nlm.nih.gov/entrez/query.fcgi?cmd=Retrieve&db=pubmed&dopt=Abstract&list_uids=27524420|6|Oncogene|||Aug|2016|JOURNAL ARTICLE
akap13	twist1	http://www.ncbi.nlm.nih.gov/entrez/query.fcgi?cmd=Retrieve&db=pubmed&dopt=Abstract&list_uids=27524420|6|Oncogene|||Aug|2016|JOURNAL ARTICLE

cda	hpd	http://www.ncbi.nlm.nih.gov/entrez/query.fcgi?cmd=Retrieve&db=pubmed&dopt=Abstract&list_uids=27527502|6|BMC Genomics|17|1||2016|Journal Article
slc9a1	tnfsf13	http://www.ncbi.nlm.nih.gov/entrez/query.fcgi?cmd=Retrieve&db=pubmed&dopt=Abstract&list_uids=27529686|6|Int J Cancer|||Aug|2016|JOURNAL ARTICLE
slc9a1	hgf	http://www.ncbi.nlm.nih.gov/entrez/query.fcgi?cmd=Retrieve&db=pubmed&dopt=Abstract&list_uids=27529686|6|Int J Cancer|||Aug|2016|JOURNAL ARTICLE
hgf	tnfsf13	http://www.ncbi.nlm.nih.gov/entrez/query.fcgi?cmd=Retrieve&db=pubmed&dopt=Abstract&list_uids=27529686|6|Int J Cancer|||Aug|2016|JOURNAL ARTICLE
angptl1	itk	http://www.ncbi.nlm.nih.gov/entrez/query.fcgi?cmd=Retrieve&db=pubmed&dopt=Abstract&list_uids=27530187|6|Hepatology|||Aug|2016|JOURNAL ARTICLE
angptl1	hcc	http://www.ncbi.nlm.nih.gov/entrez/query.fcgi?cmd=Retrieve&db=pubmed&dopt=Abstract&list_uids=27530187|6|Hepatology|||Aug|2016|JOURNAL ARTICLE
angptl1	egr1	http://www.ncbi.nlm.nih.gov/entrez/query.fcgi?cmd=Retrieve&db=pubmed&dopt=Abstract&list_uids=27530187|6|Hepatology|||Aug|2016|JOURNAL ARTICLE
angptl1	akt1	http://www.ncbi.nlm.nih.gov/entrez/query.fcgi?cmd=Retrieve&db=pubmed&dopt=Abstract&list_uids=27530187|6|Hepatology|||Aug|2016|JOURNAL ARTICLE
angptl1	ephb2	http://www.ncbi.nlm.nih.gov/entrez/query.fcgi?cmd=Retrieve&db=pubmed&dopt=Abstract&list_uids=27530187|6|Hepatology|||Aug|2016|JOURNAL ARTICLE
angptl1	met	http://www.ncbi.nlm.nih.gov/entrez/query.fcgi?cmd=Retrieve&db=pubmed&dopt=Abstract&list_uids=27530187|6|Hepatology|||Aug|2016|JOURNAL ARTICLE
eya1	bcl2	http://www.ncbi.nlm.nih.gov/entrez/query.fcgi?cmd=Retrieve&db=pubmed&dopt=Abstract&list_uids=27531778|6|Zhongguo Shi Yan Xue Ye Xue Za Zhi|24|4|Aug|2016|English Abstract,Journal Article
kcnn4	tp53	http://www.ncbi.nlm.nih.gov/entrez/query.fcgi?cmd=Retrieve&db=pubmed&dopt=Abstract&list_uids=27531900|6|Oncotarget|||Aug|2016|JOURNAL ARTICLE
dyrk2	tyr	http://www.ncbi.nlm.nih.gov/entrez/query.fcgi?cmd=Retrieve&db=pubmed&dopt=Abstract&list_uids=27532268|6|PLoS One|11|8||2016|Journal Article
mirn146b	irak1	http://www.ncbi.nlm.nih.gov/entrez/query.fcgi?cmd=Retrieve&db=pubmed&dopt=Abstract&list_uids=27533309|6|J Clin Endocrinol Metab|||Aug|2016|JOURNAL ARTICLE
mirn146b	f9	http://www.ncbi.nlm.nih.gov/entrez/query.fcgi?cmd=Retrieve&db=pubmed&dopt=Abstract&list_uids=27533309|6|J Clin Endocrinol Metab|||Aug|2016|JOURNAL ARTICLE
f9	irak1	http://www.ncbi.nlm.nih.gov/entrez/query.fcgi?cmd=Retrieve&db=pubmed&dopt=Abstract&list_uids=27533309|6|J Clin Endocrinol Metab|||Aug|2016|JOURNAL ARTICLE
gli1	itk	http://www.ncbi.nlm.nih.gov/entrez/query.fcgi?cmd=Retrieve&db=pubmed&dopt=Abstract&list_uids=27533453|6|Oncotarget|||Aug|2016|JOURNAL ARTICLE
smo	gli1	http://www.ncbi.nlm.nih.gov/entrez/query.fcgi?cmd=Retrieve&db=pubmed&dopt=Abstract&list_uids=27533453|6|Oncotarget|||Aug|2016|JOURNAL ARTICLE

shh	gli1	http://www.ncbi.nlm.nih.gov/entrez/query.fcgi?cmd=Retrieve&db=pubmed&dopt=Abstract&list_uids=27533453|6|Oncotarget|||Aug|2016|JOURNAL ARTICLE
shh	smo	http://www.ncbi.nlm.nih.gov/entrez/query.fcgi?cmd=Retrieve&db=pubmed&dopt=Abstract&list_uids=27533453|6|Oncotarget|||Aug|2016|JOURNAL ARTICLE
alcam	stat6	http://www.ncbi.nlm.nih.gov/entrez/query.fcgi?cmd=Retrieve&db=pubmed&dopt=Abstract&list_uids=27533463|6|Oncotarget|||Aug|2016|JOURNAL ARTICLE
fut8	hcc	http://www.ncbi.nlm.nih.gov/entrez/query.fcgi?cmd=Retrieve&db=pubmed&dopt=Abstract&list_uids=27533464|6|Oncotarget|||Aug|2016|JOURNAL ARTICLE
spry1	rela	http://www.ncbi.nlm.nih.gov/entrez/query.fcgi?cmd=Retrieve&db=pubmed&dopt=Abstract&list_uids=27533779|6|Mol Carcinog|||Aug|2016|JOURNAL ARTICLE
spry1	eif2ak3	http://www.ncbi.nlm.nih.gov/entrez/query.fcgi?cmd=Retrieve&db=pubmed&dopt=Abstract&list_uids=27533779|6|Mol Carcinog|||Aug|2016|JOURNAL ARTICLE
spry1	ephb2	http://www.ncbi.nlm.nih.gov/entrez/query.fcgi?cmd=Retrieve&db=pubmed&dopt=Abstract&list_uids=27533779|6|Mol Carcinog|||Aug|2016|JOURNAL ARTICLE
spry1	pten	http://www.ncbi.nlm.nih.gov/entrez/query.fcgi?cmd=Retrieve&db=pubmed&dopt=Abstract&list_uids=27533779|6|Mol Carcinog|||Aug|2016|JOURNAL ARTICLE
spry1	pdcd4	http://www.ncbi.nlm.nih.gov/entrez/query.fcgi?cmd=Retrieve&db=pubmed&dopt=Abstract&list_uids=27533779|6|Mol Carcinog|||Aug|2016|JOURNAL ARTICLE
mirn21	rela	http://www.ncbi.nlm.nih.gov/entrez/query.fcgi?cmd=Retrieve&db=pubmed&dopt=Abstract&list_uids=27533779|6|Mol Carcinog|||Aug|2016|JOURNAL ARTICLE
mirn21	eif2ak3	http://www.ncbi.nlm.nih.gov/entrez/query.fcgi?cmd=Retrieve&db=pubmed&dopt=Abstract&list_uids=27533779|6|Mol Carcinog|||Aug|2016|JOURNAL ARTICLE
mirn21	ephb2	http://www.ncbi.nlm.nih.gov/entrez/query.fcgi?cmd=Retrieve&db=pubmed&dopt=Abstract&list_uids=27533779|6|Mol Carcinog|||Aug|2016|JOURNAL ARTICLE
mirn21	spry1	http://www.ncbi.nlm.nih.gov/entrez/query.fcgi?cmd=Retrieve&db=pubmed&dopt=Abstract&list_uids=27533779|6|Mol Carcinog|||Aug|2016|JOURNAL ARTICLE
mirn21	mmp9	http://www.ncbi.nlm.nih.gov/entrez/query.fcgi?cmd=Retrieve&db=pubmed&dopt=Abstract&list_uids=27600360|6|Cell Biol Int|||Sep|2016|JOURNAL ARTICLE
mirn21	mmp2	http://www.ncbi.nlm.nih.gov/entrez/query.fcgi?cmd=Retrieve&db=pubmed&dopt=Abstract&list_uids=27600360|6|Cell Biol Int|||Sep|2016|JOURNAL ARTICLE
mirn21	bcl2	http://www.ncbi.nlm.nih.gov/entrez/query.fcgi?cmd=Retrieve&db=pubmed&dopt=Abstract&list_uids=27600360|6|Cell Biol Int|||Sep|2016|JOURNAL ARTICLE
mirn21	bax	http://www.ncbi.nlm.nih.gov/entrez/query.fcgi?cmd=Retrieve&db=pubmed&dopt=Abstract&list_uids=27600360|6|Cell Biol Int|||Sep|2016|JOURNAL ARTICLE
mirn21	pdcd4	http://www.ncbi.nlm.nih.gov/entrez/query.fcgi?cmd=Retrieve&db=pubmed&dopt=Abstract&list_uids=27600360|6|Cell Biol Int|||Sep|2016|JOURNAL

		ARTICLE|http://www.ncbi.nlm.nih.gov/entrez/query.fcgi?cmd=Retrieve&db=pubmed&dopt=Abstract&list_uids=27533779|6|Mol Carcinog|||Aug|2016|JOURNAL ARTICLE
mirn21	akt1	http://www.ncbi.nlm.nih.gov/entrez/query.fcgi?cmd=Retrieve&db=pubmed&dopt=Abstract&list_uids=27600360|6|Cell Biol Int|||Sep|2016|JOURNAL ARTICLE
mirn21	pik3ca	http://www.ncbi.nlm.nih.gov/entrez/query.fcgi?cmd=Retrieve&db=pubmed&dopt=Abstract&list_uids=27600360|6|Cell Biol Int|||Sep|2016|JOURNAL ARTICLE
mirn21	pten	http://www.ncbi.nlm.nih.gov/entrez/query.fcgi?cmd=Retrieve&db=pubmed&dopt=Abstract&list_uids=27600360|6|Cell Biol Int|||Sep|2016|JOURNAL ARTICLE|http://www.ncbi.nlm.nih.gov/entrez/query.fcgi?cmd=Retrieve&db=pubmed&dopt=Abstract&list_uids=27533779|6|Mol Carcinog|||Aug|2016|JOURNAL ARTICLE
nfatc2	mdm2	http://www.ncbi.nlm.nih.gov/entrez/query.fcgi?cmd=Retrieve&db=pubmed&dopt=Abstract&list_uids=27533941|6|J Biomed Res|30|4|Jul|2016|Journal Article
mirn144	hcc	http://www.ncbi.nlm.nih.gov/entrez/query.fcgi?cmd=Retrieve&db=pubmed&dopt=Abstract&list_uids=27536132|6|Onco Targets Ther|9|||2016|Journal Article
fat	vhl	http://www.ncbi.nlm.nih.gov/entrez/query.fcgi?cmd=Retrieve&db=pubmed&dopt=Abstract&list_uids=27536856|6|Int J Cancer|||Aug|2016|JOURNAL ARTICLE
mirn23a	st7l	http://www.ncbi.nlm.nih.gov/entrez/query.fcgi?cmd=Retrieve&db=pubmed&dopt=Abstract&list_uids=27537390|6|Br J Cancer|115|6|Sep|2016|Journal Article
etv4	erbb2	http://www.ncbi.nlm.nih.gov/entrez/query.fcgi?cmd=Retrieve&db=pubmed&dopt=Abstract&list_uids=27537398|6|Anticancer Drugs|||Aug|2016|JOURNAL ARTICLE
cmtm3	pcsk1	http://www.ncbi.nlm.nih.gov/entrez/query.fcgi?cmd=Retrieve&db=pubmed&dopt=Abstract&list_uids=27538134|6|Beijing Da Xue Xue Bao|48|1|Feb|2016|English Abstract,Journal Article
cdk2	ephb2	http://www.ncbi.nlm.nih.gov/entrez/query.fcgi?cmd=Retrieve&db=pubmed&dopt=Abstract&list_uids=27542217|6|Oncotarget|||Aug|2016|JOURNAL ARTICLE
stk11	atp8a2	http://www.ncbi.nlm.nih.gov/entrez/query.fcgi?cmd=Retrieve&db=pubmed&dopt=Abstract&list_uids=27546620|6|Oncogene|||Aug|2016|JOURNAL ARTICLE
sdhd	tlr2	http://www.ncbi.nlm.nih.gov/entrez/query.fcgi?cmd=Retrieve&db=pubmed&dopt=Abstract&list_uids=27548027|6|ACS Chem Biol|||Sep|2016|JOURNAL ARTICLE
pgls	tlr2	http://www.ncbi.nlm.nih.gov/entrez/query.fcgi?cmd=Retrieve&db=pubmed&dopt=Abstract&list_uids=27548027|6|ACS Chem Biol|||Sep|2016|JOURNAL ARTICLE
cdkn1c	rbp2	http://www.ncbi.nlm.nih.gov/entrez/query.fcgi?cmd=Retrieve&db=pubmed&dopt=Abstract&list_uids=27548814|6|Oncogenesis|5|8||2016|Journal Article
cox7a2l	cox8a	http://www.ncbi.nlm.nih.gov/entrez/query.fcgi?cmd=Retrieve&db=pubmed&dopt=Abstract&list_uids=27550821|6|Sci Rep|6|||2016|Journal Article
bap1	lct	http://www.ncbi.nlm.nih.gov/entrez/query.fcgi?cmd=Retrieve&db=pubmed&dopt=Abstract&list_uids=27553041|6|BMC Cancer|16|1||2016|Journal Article

tbx3	itk	http://www.ncbi.nlm.nih.gov/entrez/query.fcgi?cmd=Retrieve&db=pubmed&dopt=Abstract&list_uids=27553355|6|Virchows Arch|||Aug|2016|JOURNAL ARTICLE
bcs1l	erbb2	http://www.ncbi.nlm.nih.gov/entrez/query.fcgi?cmd=Retrieve&db=pubmed&dopt=Abstract&list_uids=27553713|6|Int J Cancer|||Aug|2016|REVIEW,JOURNAL ARTICLE
ephb4	icam1	http://www.ncbi.nlm.nih.gov/entrez/query.fcgi?cmd=Retrieve&db=pubmed&dopt=Abstract&list_uids=27553867|6|Am J Reprod Immunol|||Aug|2016|JOURNAL ARTICLE
ephb4	stat1	http://www.ncbi.nlm.nih.gov/entrez/query.fcgi?cmd=Retrieve&db=pubmed&dopt=Abstract&list_uids=27553867|6|Am J Reprod Immunol|||Aug|2016|JOURNAL ARTICLE
epha1	ephb4	http://www.ncbi.nlm.nih.gov/entrez/query.fcgi?cmd=Retrieve&db=pubmed&dopt=Abstract&list_uids=27553867|6|Am J Reprod Immunol|||Aug|2016|JOURNAL ARTICLE
dbp	tnfsf13	http://www.ncbi.nlm.nih.gov/entrez/query.fcgi?cmd=Retrieve&db=pubmed&dopt=Abstract&list_uids=27555313|6|MBio|7|4||2016|Journal Article
dbp	darc	http://www.ncbi.nlm.nih.gov/entrez/query.fcgi?cmd=Retrieve&db=pubmed&dopt=Abstract&list_uids=27555313|6|MBio|7|4||2016|Journal Article
ebna1bp2	darc	http://www.ncbi.nlm.nih.gov/entrez/query.fcgi?cmd=Retrieve&db=pubmed&dopt=Abstract&list_uids=27555313|6|MBio|7|4||2016|Journal Article
ebna1bp2	tfrc	http://www.ncbi.nlm.nih.gov/entrez/query.fcgi?cmd=Retrieve&db=pubmed&dopt=Abstract&list_uids=27555313|6|MBio|7|4||2016|Journal Article
ebna1bp2	tnfsf13	http://www.ncbi.nlm.nih.gov/entrez/query.fcgi?cmd=Retrieve&db=pubmed&dopt=Abstract&list_uids=27555313|6|MBio|7|4||2016|Journal Article
ebna1bp2	dbp	http://www.ncbi.nlm.nih.gov/entrez/query.fcgi?cmd=Retrieve&db=pubmed&dopt=Abstract&list_uids=27555313|6|MBio|7|4||2016|Journal Article
icam2	ephb2	http://www.ncbi.nlm.nih.gov/entrez/query.fcgi?cmd=Retrieve&db=pubmed&dopt=Abstract&list_uids=27556181|6|Oncotarget|||Aug|2016|JOURNAL ARTICLE
icam2	tp53	http://www.ncbi.nlm.nih.gov/entrez/query.fcgi?cmd=Retrieve&db=pubmed&dopt=Abstract&list_uids=27556181|6|Oncotarget|||Aug|2016|JOURNAL ARTICLE
ets2	frap1	http://www.ncbi.nlm.nih.gov/entrez/query.fcgi?cmd=Retrieve&db=pubmed&dopt=Abstract&list_uids=27556183|6|Oncotarget|||Aug|2016|JOURNAL ARTICLE
ncrna00084	gsc	http://www.ncbi.nlm.nih.gov/entrez/query.fcgi?cmd=Retrieve&db=pubmed&dopt=Abstract&list_uids=27556696|6|Oncotarget|||Aug|2016|JOURNAL ARTICLE
ncrna00084	mirnlet7e	http://www.ncbi.nlm.nih.gov/entrez/query.fcgi?cmd=Retrieve&db=pubmed&dopt=Abstract&list_uids=27556696|6|Oncotarget|||Aug|2016|JOURNAL ARTICLE
tnfaip8l2	vegfa	http://www.ncbi.nlm.nih.gov/entrez/query.fcgi?cmd=Retrieve&db=pubmed&dopt=Abstract&list_uids=27556698|6|Oncotarget|||Aug|2016|JOURNAL ARTICLE
tnfaip8l2	rac1	http://www.ncbi.nlm.nih.gov/entrez/query.fcgi?cmd=Retrieve&db=pubmed&dopt=Abstract&list_uids=27556698|6|Oncotarget|||Aug|2016|JOURNAL ARTICLE

rhoh	mmp9	http://www.ncbi.nlm.nih.gov/entrez/query.fcgi?cmd=Retrieve&db=pubmed&dopt=Abstract&list_uids=27556699|6|Oncotarget|||Aug|2016|JOURNAL ARTICLE
6-Sep	ephb2	http://www.ncbi.nlm.nih.gov/entrez/query.fcgi?cmd=Retrieve&db=pubmed&dopt=Abstract&list_uids=27557506|6|Oncotarget|||Aug|2016|JOURNAL ARTICLE
cdk5	ervk2	http://www.ncbi.nlm.nih.gov/entrez/query.fcgi?cmd=Retrieve&db=pubmed&dopt=Abstract&list_uids=27557521|6|Oncotarget|||Aug|2016|JOURNAL ARTICLE
cdk5	tp53	http://www.ncbi.nlm.nih.gov/entrez/query.fcgi?cmd=Retrieve&db=pubmed&dopt=Abstract&list_uids=27557521|6|Oncotarget|||Aug|2016|JOURNAL ARTICLE
max	myc	http://www.ncbi.nlm.nih.gov/entrez/query.fcgi?cmd=Retrieve&db=pubmed&dopt=Abstract&list_uids=27557535|6|Recent Results Cancer Res|207|||2016|Journal Article
adsl	defb103a	http://www.ncbi.nlm.nih.gov/entrez/query.fcgi?cmd=Retrieve&db=pubmed&dopt=Abstract&list_uids=27558278|6|J Periodontal Res|||Aug|2016|JOURNAL ARTICLE
adsl	il6	http://www.ncbi.nlm.nih.gov/entrez/query.fcgi?cmd=Retrieve&db=pubmed&dopt=Abstract&list_uids=27558278|6|J Periodontal Res|||Aug|2016|JOURNAL ARTICLE
adsl	il8	http://www.ncbi.nlm.nih.gov/entrez/query.fcgi?cmd=Retrieve&db=pubmed&dopt=Abstract&list_uids=27558278|6|J Periodontal Res|||Aug|2016|JOURNAL ARTICLE
adsl	ccl20	http://www.ncbi.nlm.nih.gov/entrez/query.fcgi?cmd=Retrieve&db=pubmed&dopt=Abstract&list_uids=27558278|6|J Periodontal Res|||Aug|2016|JOURNAL ARTICLE
adsl	tnfsf13	http://www.ncbi.nlm.nih.gov/entrez/query.fcgi?cmd=Retrieve&db=pubmed&dopt=Abstract&list_uids=27558278|6|J Periodontal Res|||Aug|2016|JOURNAL ARTICLE
adsl	il8ra	http://www.ncbi.nlm.nih.gov/entrez/query.fcgi?cmd=Retrieve&db=pubmed&dopt=Abstract&list_uids=27558278|6|J Periodontal Res|||Aug|2016|JOURNAL ARTICLE
adsl	cxcl11	http://www.ncbi.nlm.nih.gov/entrez/query.fcgi?cmd=Retrieve&db=pubmed&dopt=Abstract&list_uids=27558278|6|J Periodontal Res|||Aug|2016|JOURNAL ARTICLE
ccl20	il6	http://www.ncbi.nlm.nih.gov/entrez/query.fcgi?cmd=Retrieve&db=pubmed&dopt=Abstract&list_uids=27558278|6|J Periodontal Res|||Aug|2016|JOURNAL ARTICLE
ccl20	il8	http://www.ncbi.nlm.nih.gov/entrez/query.fcgi?cmd=Retrieve&db=pubmed&dopt=Abstract&list_uids=27558278|6|J Periodontal Res|||Aug|2016|JOURNAL ARTICLE
cxcl11	il6	http://www.ncbi.nlm.nih.gov/entrez/query.fcgi?cmd=Retrieve&db=pubmed&dopt=Abstract&list_uids=27558278|6|J Periodontal Res|||Aug|2016|JOURNAL ARTICLE
cxcl11	il8	http://www.ncbi.nlm.nih.gov/entrez/query.fcgi?cmd=Retrieve&db=pubmed&dopt=Abstract&list_uids=27558278|6|J Periodontal Res|||Aug|2016|JOURNAL ARTICLE
cxcl11	ccl20	http://www.ncbi.nlm.nih.gov/entrez/query.fcgi?cmd=Retrieve&db=pubmed&dopt=Abstract&list_uids=27558278|6|J Periodontal Res|||Aug|2016|JOURNAL ARTICLE
cxcl11	tnfsf13	http://www.ncbi.nlm.nih.gov/entrez/query.fcgi?cmd=Retrieve&db=pubmed&dopt=Abstract&list_uids=27558278|6|J Periodontal Res|||Aug|2016|JOURNAL ARTICLE

cxcl11	il8ra	http://www.ncbi.nlm.nih.gov/entrez/query.fcgi?cmd=Retrieve&db=pubmed&dopt=Abstract&list_uids=27558278|6|J Periodontal Res|||Aug|2016|JOURNAL ARTICLE
foxc2	itk	http://www.ncbi.nlm.nih.gov/entrez/query.fcgi?cmd=Retrieve&db=pubmed&dopt=Abstract&list_uids=27562816|6|Cell Physiol Biochem|39|3||2016|Journal Article
nanogp8	frap1	http://www.ncbi.nlm.nih.gov/entrez/query.fcgi?cmd=Retrieve&db=pubmed&dopt=Abstract&list_uids=27563247|6|Onco Targets Ther|9|||2016|Journal Article
nanogp8	akt1	http://www.ncbi.nlm.nih.gov/entrez/query.fcgi?cmd=Retrieve&db=pubmed&dopt=Abstract&list_uids=27563247|6|Onco Targets Ther|9|||2016|Journal Article
malat1	mirn22	http://www.ncbi.nlm.nih.gov/entrez/query.fcgi?cmd=Retrieve&db=pubmed&dopt=Abstract&list_uids=27564100|6|Oncotarget|||Aug|2016|JOURNAL ARTICLE
malat1	mapk8	http://www.ncbi.nlm.nih.gov/entrez/query.fcgi?cmd=Retrieve&db=pubmed&dopt=Abstract&list_uids=27565324|6|Neoplasma||6|Aug|2016|JOURNAL ARTICLE
malat1	mapk14	http://www.ncbi.nlm.nih.gov/entrez/query.fcgi?cmd=Retrieve&db=pubmed&dopt=Abstract&list_uids=27565324|6|Neoplasma||6|Aug|2016|JOURNAL ARTICLE
malat1	mapk3	http://www.ncbi.nlm.nih.gov/entrez/query.fcgi?cmd=Retrieve&db=pubmed&dopt=Abstract&list_uids=27565324|6|Neoplasma||6|Aug|2016|JOURNAL ARTICLE
malat1	map2k1	http://www.ncbi.nlm.nih.gov/entrez/query.fcgi?cmd=Retrieve&db=pubmed&dopt=Abstract&list_uids=27565324|6|Neoplasma||6|Aug|2016|JOURNAL ARTICLE
malat1	tgfb1	http://www.ncbi.nlm.nih.gov/entrez/query.fcgi?cmd=Retrieve&db=pubmed&dopt=Abstract&list_uids=27565324|6|Neoplasma||6|Aug|2016|JOURNAL ARTICLE
map2k1	mapk8	http://www.ncbi.nlm.nih.gov/entrez/query.fcgi?cmd=Retrieve&db=pubmed&dopt=Abstract&list_uids=27565324|6|Neoplasma||6|Aug|2016|JOURNAL ARTICLE
map2k1	jun	http://www.ncbi.nlm.nih.gov/entrez/query.fcgi?cmd=Retrieve&db=pubmed&dopt=Abstract&list_uids=27565324|6|Neoplasma||6|Aug|2016|JOURNAL ARTICLE
map2k1	mapk14	http://www.ncbi.nlm.nih.gov/entrez/query.fcgi?cmd=Retrieve&db=pubmed&dopt=Abstract&list_uids=27565324|6|Neoplasma||6|Aug|2016|JOURNAL ARTICLE
map2k1	mapk3	http://www.ncbi.nlm.nih.gov/entrez/query.fcgi?cmd=Retrieve&db=pubmed&dopt=Abstract&list_uids=27565324|6|Neoplasma||6|Aug|2016|JOURNAL ARTICLE
recql	itk	http://www.ncbi.nlm.nih.gov/entrez/query.fcgi?cmd=Retrieve&db=pubmed&dopt=Abstract&list_uids=27565844|6|Biomed Pharmacother|83||Aug|2016|JOURNAL ARTICLE
rhag	mmp1	http://www.ncbi.nlm.nih.gov/entrez/query.fcgi?cmd=Retrieve&db=pubmed&dopt=Abstract&list_uids=27566200|6|J Ethnopharmacol|||Aug|2016|JOURNAL ARTICLE
rhag	stat3	http://www.ncbi.nlm.nih.gov/entrez/query.fcgi?cmd=Retrieve&db=pubmed&dopt=Abstract&list_uids=27566200|6|J Ethnopharmacol|||Aug|2016|JOURNAL ARTICLE
rhag	il6	http://www.ncbi.nlm.nih.gov/entrez/query.fcgi?cmd=Retrieve&db=pubmed&dopt=Abstract&list_uids=27566200|6|J Ethnopharmacol|||Aug|2016|JOURNAL ARTICLE

dyrk1a	pten	http://www.ncbi.nlm.nih.gov/entrez/query.fcgi?cmd=Retrieve&db=pubmed&dopt=Abstract&list_uids=27569213|6|Cancer Res|||Aug|2016|JOURNAL ARTICLE
tbx5	pten	http://www.ncbi.nlm.nih.gov/entrez/query.fcgi?cmd=Retrieve&db=pubmed&dopt=Abstract&list_uids=27569213|6|Cancer Res|||Aug|2016|JOURNAL ARTICLE
tbx5	dyrk1a	http://www.ncbi.nlm.nih.gov/entrez/query.fcgi?cmd=Retrieve&db=pubmed&dopt=Abstract&list_uids=27569213|6|Cancer Res|||Aug|2016|JOURNAL ARTICLE
cd40	akt1	http://www.ncbi.nlm.nih.gov/entrez/query.fcgi?cmd=Retrieve&db=pubmed&dopt=Abstract&list_uids=27569589|6|Life Sci|||Aug|2016|JOURNAL ARTICLE
cd40	pik3ca	http://www.ncbi.nlm.nih.gov/entrez/query.fcgi?cmd=Retrieve&db=pubmed&dopt=Abstract&list_uids=27569589|6|Life Sci|||Aug|2016|JOURNAL ARTICLE
nfkb1	akt1	http://www.ncbi.nlm.nih.gov/entrez/query.fcgi?cmd=Retrieve&db=pubmed&dopt=Abstract&list_uids=27569589|6|Life Sci|||Aug|2016|JOURNAL ARTICLE
nfkb1	pik3ca	http://www.ncbi.nlm.nih.gov/entrez/query.fcgi?cmd=Retrieve&db=pubmed&dopt=Abstract&list_uids=27569589|6|Life Sci|||Aug|2016|JOURNAL ARTICLE
nfkb1	cd40	http://www.ncbi.nlm.nih.gov/entrez/query.fcgi?cmd=Retrieve&db=pubmed&dopt=Abstract&list_uids=27569589|6|Life Sci|||Aug|2016|JOURNAL ARTICLE
mt1jp	phgdh	http://www.ncbi.nlm.nih.gov/entrez/query.fcgi?cmd=Retrieve&db=pubmed&dopt=Abstract&list_uids=27569900|6|Biochim Biophys Acta|||Aug|2016|JOURNAL ARTICLE
fbn2	phgdh	http://www.ncbi.nlm.nih.gov/entrez/query.fcgi?cmd=Retrieve&db=pubmed&dopt=Abstract&list_uids=27593106|6|J Hepatol|||Sep|2016|JOURNAL ARTICLE
xiap	fbn2	http://www.ncbi.nlm.nih.gov/entrez/query.fcgi?cmd=Retrieve&db=pubmed&dopt=Abstract&list_uids=27571770|6|Sci Rep|6|||2016|Journal Article
tacc3	timp1	http://www.ncbi.nlm.nih.gov/entrez/query.fcgi?cmd=Retrieve&db=pubmed&dopt=Abstract&list_uids=27572091|6|Mol Med Rep|||Aug|2016|JOURNAL ARTICLE
tacc3	akt1	http://www.ncbi.nlm.nih.gov/entrez/query.fcgi?cmd=Retrieve&db=pubmed&dopt=Abstract&list_uids=27572091|6|Mol Med Rep|||Aug|2016|JOURNAL ARTICLE
tacc3	pik3ca	http://www.ncbi.nlm.nih.gov/entrez/query.fcgi?cmd=Retrieve&db=pubmed&dopt=Abstract&list_uids=27572091|6|Mol Med Rep|||Aug|2016|JOURNAL ARTICLE
zhx2	ephb2	http://www.ncbi.nlm.nih.gov/entrez/query.fcgi?cmd=Retrieve&db=pubmed&dopt=Abstract&list_uids=27572273|6|Int J Oncol|49|3|Sep|2016|Journal Article
larp6	ephb2	http://www.ncbi.nlm.nih.gov/entrez/query.fcgi?cmd=Retrieve&db=pubmed&dopt=Abstract&list_uids=27572273|6|Int J Oncol|49|3|Sep|2016|Journal Article
larp6	zhx2	http://www.ncbi.nlm.nih.gov/entrez/query.fcgi?cmd=Retrieve&db=pubmed&dopt=Abstract&list_uids=27572273|6|Int J Oncol|49|3|Sep|2016|Journal Article
larp6	akt1	http://www.ncbi.nlm.nih.gov/entrez/query.fcgi?cmd=Retrieve&db=pubmed&dopt=Abstract&list_uids=27572273|6|Int J Oncol|49|3|Sep|2016|Journal Article

larp6	pik3ca	http://www.ncbi.nlm.nih.gov/entrez/query.fcgi?cmd=Retrieve&db=pubmed&dopt=Abstract&list_uids=27572273|6|Int J Oncol|49|3|Sep|2016|Journal Article
larp6	kdr	http://www.ncbi.nlm.nih.gov/entrez/query.fcgi?cmd=Retrieve&db=pubmed&dopt=Abstract&list_uids=27572273|6|Int J Oncol|49|3|Sep|2016|Journal Article
mirn195	ephb2	http://www.ncbi.nlm.nih.gov/entrez/query.fcgi?cmd=Retrieve&db=pubmed&dopt=Abstract&list_uids=27572273|6|Int J Oncol|49|3|Sep|2016|Journal Article
mirn195	zhx2	http://www.ncbi.nlm.nih.gov/entrez/query.fcgi?cmd=Retrieve&db=pubmed&dopt=Abstract&list_uids=27572273|6|Int J Oncol|49|3|Sep|2016|Journal Article
mirn195	akt1	http://www.ncbi.nlm.nih.gov/entrez/query.fcgi?cmd=Retrieve&db=pubmed&dopt=Abstract&list_uids=27572273|6|Int J Oncol|49|3|Sep|2016|Journal Article
mirn195	pik3ca	http://www.ncbi.nlm.nih.gov/entrez/query.fcgi?cmd=Retrieve&db=pubmed&dopt=Abstract&list_uids=27572273|6|Int J Oncol|49|3|Sep|2016|Journal Article
mirn195	kdr	http://www.ncbi.nlm.nih.gov/entrez/query.fcgi?cmd=Retrieve&db=pubmed&dopt=Abstract&list_uids=27572273|6|Int J Oncol|49|3|Sep|2016|Journal Article
mirn195	larp6	http://www.ncbi.nlm.nih.gov/entrez/query.fcgi?cmd=Retrieve&db=pubmed&dopt=Abstract&list_uids=27572273|6|Int J Oncol|49|3|Sep|2016|Journal Article
mirn195	smad7	http://www.ncbi.nlm.nih.gov/entrez/query.fcgi?cmd=Retrieve&db=pubmed&dopt=Abstract&list_uids=27574009|6|Oncol Rep|||Aug|2016|JOURNAL ARTICLE
mirn195	smad2	http://www.ncbi.nlm.nih.gov/entrez/query.fcgi?cmd=Retrieve&db=pubmed&dopt=Abstract&list_uids=27574009|6|Oncol Rep|||Aug|2016|JOURNAL ARTICLE
setdb2	me3	http://www.ncbi.nlm.nih.gov/entrez/query.fcgi?cmd=Retrieve&db=pubmed&dopt=Abstract&list_uids=27572307|6|Oncotarget|||Aug|2016|JOURNAL ARTICLE
ceacam1	bat2	http://www.ncbi.nlm.nih.gov/entrez/query.fcgi?cmd=Retrieve&db=pubmed&dopt=Abstract&list_uids=27572314|6|Oncotarget|||Aug|2016|JOURNAL ARTICLE
ceacam1	itk	http://www.ncbi.nlm.nih.gov/entrez/query.fcgi?cmd=Retrieve&db=pubmed&dopt=Abstract&list_uids=27572314|6|Oncotarget|||Aug|2016|JOURNAL ARTICLE
gsn	soat1	http://www.ncbi.nlm.nih.gov/entrez/query.fcgi?cmd=Retrieve&db=pubmed&dopt=Abstract&list_uids=27573444|6|Mol Med Rep|||Aug|2016|JOURNAL ARTICLE
mirn23b	mirn27b	http://www.ncbi.nlm.nih.gov/entrez/query.fcgi?cmd=Retrieve&db=pubmed&dopt=Abstract&list_uids=27573718|6|Int J Oncol|49|3|Sep|2016|Journal Article
cdk6	mmp9	http://www.ncbi.nlm.nih.gov/entrez/query.fcgi?cmd=Retrieve&db=pubmed&dopt=Abstract&list_uids=27573859|6|Oncol Rep|||Aug|2016|JOURNAL ARTICLE
cdk6	sp1	http://www.ncbi.nlm.nih.gov/entrez/query.fcgi?cmd=Retrieve&db=pubmed&dopt=Abstract&list_uids=27573859|6|Oncol Rep|||Aug|2016|JOURNAL ARTICLE
cpi1	spi1	http://www.ncbi.nlm.nih.gov/entrez/query.fcgi?cmd=Retrieve&db=pubmed&dopt=Abstract&list_uids=27573929|6|Curr Pharm Des|||Aug|2016|JOURNAL ARTICLE

sox1	sall4	http://www.ncbi.nlm.nih.gov/entrez/query.fcgi?cmd=Retrieve&db=pubmed&dopt=Abstract&list_uids=27576349|6|Gene|||Aug|2016|JOURNAL ARTICLE
angpt2	frap1	http://www.ncbi.nlm.nih.gov/entrez/query.fcgi?cmd=Retrieve&db=pubmed&dopt=Abstract&list_uids=27577856|6|J Pathol|||Aug|2016|JOURNAL ARTICLE
angpt2	mirn100	http://www.ncbi.nlm.nih.gov/entrez/query.fcgi?cmd=Retrieve&db=pubmed&dopt=Abstract&list_uids=27577856|6|J Pathol|||Aug|2016|JOURNAL ARTICLE
mirn100	frap1	http://www.ncbi.nlm.nih.gov/entrez/query.fcgi?cmd=Retrieve&db=pubmed&dopt=Abstract&list_uids=27577856|6|J Pathol|||Aug|2016|JOURNAL ARTICLE
rfxank	mirn216a	http://www.ncbi.nlm.nih.gov/entrez/query.fcgi?cmd=Retrieve&db=pubmed&dopt=Abstract&list_uids=27578985|6|Onco Targets Ther|9|||2016|Journal Article
rfxank	akt1	http://www.ncbi.nlm.nih.gov/entrez/query.fcgi?cmd=Retrieve&db=pubmed&dopt=Abstract&list_uids=27578985|6|Onco Targets Ther|9|||2016|Journal Article
rfxank	pik3ca	http://www.ncbi.nlm.nih.gov/entrez/query.fcgi?cmd=Retrieve&db=pubmed&dopt=Abstract&list_uids=27578985|6|Onco Targets Ther|9|||2016|Journal Article
rfxank	pik3r2	http://www.ncbi.nlm.nih.gov/entrez/query.fcgi?cmd=Retrieve&db=pubmed&dopt=Abstract&list_uids=27578985|6|Onco Targets Ther|9|||2016|Journal Article
pik3r2	uts2r	http://www.ncbi.nlm.nih.gov/entrez/query.fcgi?cmd=Retrieve&db=pubmed&dopt=Abstract&list_uids=27578985|6|Onco Targets Ther|9|||2016|Journal Article
pik3r2	mirn216a	http://www.ncbi.nlm.nih.gov/entrez/query.fcgi?cmd=Retrieve&db=pubmed&dopt=Abstract&list_uids=27578985|6|Onco Targets Ther|9|||2016|Journal Article
pik3r2	akt1	http://www.ncbi.nlm.nih.gov/entrez/query.fcgi?cmd=Retrieve&db=pubmed&dopt=Abstract&list_uids=27578985|6|Onco Targets Ther|9|||2016|Journal Article
pik3r2	pik3ca	http://www.ncbi.nlm.nih.gov/entrez/query.fcgi?cmd=Retrieve&db=pubmed&dopt=Abstract&list_uids=27578985|6|Onco Targets Ther|9|||2016|Journal Article
id2	tcf4	http://www.ncbi.nlm.nih.gov/entrez/query.fcgi?cmd=Retrieve&db=pubmed&dopt=Abstract&list_uids=27580028|6|Nature|||Aug|2016|JOURNAL ARTICLE
sox12	cdk2	http://www.ncbi.nlm.nih.gov/entrez/query.fcgi?cmd=Retrieve&db=pubmed&dopt=Abstract&list_uids=27582508|6|Biosci Rep|||Aug|2016|JOURNAL ARTICLE
sox12	pcna	http://www.ncbi.nlm.nih.gov/entrez/query.fcgi?cmd=Retrieve&db=pubmed&dopt=Abstract&list_uids=27582508|6|Biosci Rep|||Aug|2016|JOURNAL ARTICLE
mirn25	malat1	http://www.ncbi.nlm.nih.gov/entrez/query.fcgi?cmd=Retrieve&db=pubmed&dopt=Abstract&list_uids=27584791|6|Cell Death Dis|7|9||2016|Journal Article
rbm24	malat1	http://www.ncbi.nlm.nih.gov/entrez/query.fcgi?cmd=Retrieve&db=pubmed&dopt=Abstract&list_uids=27584791|6|Cell Death Dis|7|9||2016|Journal Article
rbm24	mirn25	http://www.ncbi.nlm.nih.gov/entrez/query.fcgi?cmd=Retrieve&db=pubmed&dopt=Abstract&list_uids=27584791|6|Cell Death Dis|7|9||2016|Journal Article

rbm24	npc1	http://www.ncbi.nlm.nih.gov/entrez/query.fcgi?cmd=Retrieve&db=pubmed&dopt=Abstract&list_uids=27584791|6|Cell Death Dis|7|9||2016|Journal Article
loc553103	itk	http://www.ncbi.nlm.nih.gov/entrez/query.fcgi?cmd=Retrieve&db=pubmed&dopt=Abstract&list_uids=27584792|6|Cell Death Dis|7|9||2016|Journal Article
camp	csda	http://www.ncbi.nlm.nih.gov/entrez/query.fcgi?cmd=Retrieve&db=pubmed&dopt=Abstract&list_uids=27588122|6|Oncol Lett|12|3|Sep|2016|JOURNAL ARTICLE
csda	serpinb3	http://www.ncbi.nlm.nih.gov/entrez/query.fcgi?cmd=Retrieve&db=pubmed&dopt=Abstract&list_uids=27588122|6|Oncol Lett|12|3|Sep|2016|JOURNAL ARTICLE
mirn204	atf2	http://www.ncbi.nlm.nih.gov/entrez/query.fcgi?cmd=Retrieve&db=pubmed&dopt=Abstract&list_uids=27588402|6|Oncotarget|||Aug|2016|JOURNAL ARTICLE
lepr	ephb2	http://www.ncbi.nlm.nih.gov/entrez/query.fcgi?cmd=Retrieve&db=pubmed&dopt=Abstract&list_uids=27588409|6|Oncotarget|||Aug|2016|JOURNAL ARTICLE
lepr	mapk14	http://www.ncbi.nlm.nih.gov/entrez/query.fcgi?cmd=Retrieve&db=pubmed&dopt=Abstract&list_uids=27588409|6|Oncotarget|||Aug|2016|JOURNAL ARTICLE
st6gal1	pc-3	http://www.ncbi.nlm.nih.gov/entrez/query.fcgi?cmd=Retrieve&db=pubmed&dopt=Abstract&list_uids=27588482|6|Oncotarget|||Aug|2016|JOURNAL ARTICLE
il34	phgdh	http://www.ncbi.nlm.nih.gov/entrez/query.fcgi?cmd=Retrieve&db=pubmed&dopt=Abstract&list_uids=27593106|6|J Hepatol|||Sep|2016|JOURNAL ARTICLE
il34	fbn2	http://www.ncbi.nlm.nih.gov/entrez/query.fcgi?cmd=Retrieve&db=pubmed&dopt=Abstract&list_uids=27593106|6|J Hepatol|||Sep|2016|JOURNAL ARTICLE
csf1r	mrc1	http://www.ncbi.nlm.nih.gov/entrez/query.fcgi?cmd=Retrieve&db=pubmed&dopt=Abstract&list_uids=27593106|6|J Hepatol|||Sep|2016|JOURNAL ARTICLE
cd68	mrc1	http://www.ncbi.nlm.nih.gov/entrez/query.fcgi?cmd=Retrieve&db=pubmed&dopt=Abstract&list_uids=27593106|6|J Hepatol|||Sep|2016|JOURNAL ARTICLE
cd68	csf1r	http://www.ncbi.nlm.nih.gov/entrez/query.fcgi?cmd=Retrieve&db=pubmed&dopt=Abstract&list_uids=27593106|6|J Hepatol|||Sep|2016|JOURNAL ARTICLE
hpse	lef1	http://www.ncbi.nlm.nih.gov/entrez/query.fcgi?cmd=Retrieve&db=pubmed&dopt=Abstract&list_uids=27595937|6|Sci Rep|6|||2016|Journal Article
slamf7	egf	http://www.ncbi.nlm.nih.gov/entrez/query.fcgi?cmd=Retrieve&db=pubmed&dopt=Abstract&list_uids=27598237|6|Eur J Med Chem|124||Aug|2016|JOURNAL ARTICLE
ppm1h	itk	http://www.ncbi.nlm.nih.gov/entrez/query.fcgi?cmd=Retrieve&db=pubmed&dopt=Abstract&list_uids=27599670|6|Oncol Rep|||Sep|2016|JOURNAL ARTICLE
znf395	akt1	http://www.ncbi.nlm.nih.gov/entrez/query.fcgi?cmd=Retrieve&db=pubmed&dopt=Abstract&list_uids=27599909|6|Mol Med Rep|||Sep|2016|JOURNAL ARTICLE
znf395	stat3	http://www.ncbi.nlm.nih.gov/entrez/query.fcgi?cmd=Retrieve&db=pubmed&dopt=Abstract&list_uids=27599909|6|Mol Med Rep|||Sep|2016|JOURNAL ARTICLE

znf395	mapk8	http://www.ncbi.nlm.nih.gov/entrez/query.fcgi?cmd=Retrieve&db=pubmed&dopt=Abstract&list_uids=27599909|6|Mol Med Rep|||Sep|2016|JOURNAL ARTICLE
app	akt1	http://www.ncbi.nlm.nih.gov/entrez/query.fcgi?cmd=Retrieve&db=pubmed&dopt=Abstract&list_uids=27599909|6|Mol Med Rep|||Sep|2016|JOURNAL ARTICLE
app	stat3	http://www.ncbi.nlm.nih.gov/entrez/query.fcgi?cmd=Retrieve&db=pubmed&dopt=Abstract&list_uids=27599909|6|Mol Med Rep|||Sep|2016|JOURNAL ARTICLE
app	mapk8	http://www.ncbi.nlm.nih.gov/entrez/query.fcgi?cmd=Retrieve&db=pubmed&dopt=Abstract&list_uids=27599909|6|Mol Med Rep|||Sep|2016|JOURNAL ARTICLE
app	znf395	http://www.ncbi.nlm.nih.gov/entrez/query.fcgi?cmd=Retrieve&db=pubmed&dopt=Abstract&list_uids=27599909|6|Mol Med Rep|||Sep|2016|JOURNAL ARTICLE
tarbp2	akt1	http://www.ncbi.nlm.nih.gov/entrez/query.fcgi?cmd=Retrieve&db=pubmed&dopt=Abstract&list_uids=27599909|6|Mol Med Rep|||Sep|2016|JOURNAL ARTICLE
tarbp2	stat3	http://www.ncbi.nlm.nih.gov/entrez/query.fcgi?cmd=Retrieve&db=pubmed&dopt=Abstract&list_uids=27599909|6|Mol Med Rep|||Sep|2016|JOURNAL ARTICLE
tarbp2	mapk8	http://www.ncbi.nlm.nih.gov/entrez/query.fcgi?cmd=Retrieve&db=pubmed&dopt=Abstract&list_uids=27599909|6|Mol Med Rep|||Sep|2016|JOURNAL ARTICLE
tarbp2	znf395	http://www.ncbi.nlm.nih.gov/entrez/query.fcgi?cmd=Retrieve&db=pubmed&dopt=Abstract&list_uids=27599909|6|Mol Med Rep|||Sep|2016|JOURNAL ARTICLE
tarbp2	app	http://www.ncbi.nlm.nih.gov/entrez/query.fcgi?cmd=Retrieve&db=pubmed&dopt=Abstract&list_uids=27599909|6|Mol Med Rep|||Sep|2016|JOURNAL ARTICLE
tar	tarbp2	http://www.ncbi.nlm.nih.gov/entrez/query.fcgi?cmd=Retrieve&db=pubmed&dopt=Abstract&list_uids=27599909|6|Mol Med Rep|||Sep|2016|JOURNAL ARTICLE
hoxa7	hcc	http://www.ncbi.nlm.nih.gov/entrez/query.fcgi?cmd=Retrieve&db=pubmed&dopt=Abstract&list_uids=27600149|6|Mol Cancer|15|1||2016|Journal Article
mitf	mmp13	http://www.ncbi.nlm.nih.gov/entrez/query.fcgi?cmd=Retrieve&db=pubmed&dopt=Abstract&list_uids=27600927|6|J Dermatol Sci|||Jun|2016|JOURNAL ARTICLE
mitf	mmp14	http://www.ncbi.nlm.nih.gov/entrez/query.fcgi?cmd=Retrieve&db=pubmed&dopt=Abstract&list_uids=27600927|6|J Dermatol Sci|||Jun|2016|JOURNAL ARTICLE
mitf	mmp2	http://www.ncbi.nlm.nih.gov/entrez/query.fcgi?cmd=Retrieve&db=pubmed&dopt=Abstract&list_uids=27600927|6|J Dermatol Sci|||Jun|2016|JOURNAL ARTICLE
mitf	hnf4a	http://www.ncbi.nlm.nih.gov/entrez/query.fcgi?cmd=Retrieve&db=pubmed&dopt=Abstract&list_uids=27600927|6|J Dermatol Sci|||Jun|2016|JOURNAL ARTICLE
phb2	ephb2	http://www.ncbi.nlm.nih.gov/entrez/query.fcgi?cmd=Retrieve&db=pubmed&dopt=Abstract&list_uids=27601158|6|Int J Oncol|||Aug|2016|JOURNAL ARTICLE
phb2	ahr	http://www.ncbi.nlm.nih.gov/entrez/query.fcgi?cmd=Retrieve&db=pubmed&dopt=Abstract&list_uids=27601158|6|Int J Oncol|||Aug|2016|JOURNAL ARTICLE

phb2	myc	http://www.ncbi.nlm.nih.gov/entrez/query.fcgi?cmd=Retrieve&db=pubmed&dopt=Abstract&list_uids=27601158|6|Int J Oncol|||Aug|2016|JOURNAL ARTICLE
phb2	mmp9	http://www.ncbi.nlm.nih.gov/entrez/query.fcgi?cmd=Retrieve&db=pubmed&dopt=Abstract&list_uids=27601158|6|Int J Oncol|||Aug|2016|JOURNAL ARTICLE
wnt5a	mapk8	http://www.ncbi.nlm.nih.gov/entrez/query.fcgi?cmd=Retrieve&db=pubmed&dopt=Abstract&list_uids=27602114|6|Oncol Lett|12|3|Sep|2016|JOURNAL ARTICLE
wnt5a	mmp2	http://www.ncbi.nlm.nih.gov/entrez/query.fcgi?cmd=Retrieve&db=pubmed&dopt=Abstract&list_uids=27602114|6|Oncol Lett|12|3|Sep|2016|JOURNAL ARTICLE
ulbp2	adam10	http://www.ncbi.nlm.nih.gov/entrez/query.fcgi?cmd=Retrieve&db=pubmed&dopt=Abstract&list_uids=27602753|6|Oncotarget|||Sep|2016|JOURNAL ARTICLE
mia	adam10	http://www.ncbi.nlm.nih.gov/entrez/query.fcgi?cmd=Retrieve&db=pubmed&dopt=Abstract&list_uids=27602753|6|Oncotarget|||Sep|2016|JOURNAL ARTICLE
hmga1	mirn222	http://www.ncbi.nlm.nih.gov/entrez/query.fcgi?cmd=Retrieve&db=pubmed&dopt=Abstract&list_uids=27602961|6|Oncotarget|||Sep|2016|JOURNAL ARTICLE
hmga1	ppp2r2a	http://www.ncbi.nlm.nih.gov/entrez/query.fcgi?cmd=Retrieve&db=pubmed&dopt=Abstract&list_uids=27602961|6|Oncotarget|||Sep|2016|JOURNAL ARTICLE
hmga1	akt1	http://www.ncbi.nlm.nih.gov/entrez/query.fcgi?cmd=Retrieve&db=pubmed&dopt=Abstract&list_uids=27602961|6|Oncotarget|||Sep|2016|JOURNAL ARTICLE
il24	ppp2r2a	http://www.ncbi.nlm.nih.gov/entrez/query.fcgi?cmd=Retrieve&db=pubmed&dopt=Abstract&list_uids=27602961|6|Oncotarget|||Sep|2016|JOURNAL ARTICLE
il24	mirn222	http://www.ncbi.nlm.nih.gov/entrez/query.fcgi?cmd=Retrieve&db=pubmed&dopt=Abstract&list_uids=27602961|6|Oncotarget|||Sep|2016|JOURNAL ARTICLE
il24	hmga1	http://www.ncbi.nlm.nih.gov/entrez/query.fcgi?cmd=Retrieve&db=pubmed&dopt=Abstract&list_uids=27602961|6|Oncotarget|||Sep|2016|JOURNAL ARTICLE
il24	akt1	http://www.ncbi.nlm.nih.gov/entrez/query.fcgi?cmd=Retrieve&db=pubmed&dopt=Abstract&list_uids=27602961|6|Oncotarget|||Sep|2016|JOURNAL ARTICLE
mirn222	ppp2r2a	http://www.ncbi.nlm.nih.gov/entrez/query.fcgi?cmd=Retrieve&db=pubmed&dopt=Abstract&list_uids=27602961|6|Oncotarget|||Sep|2016|JOURNAL ARTICLE
fosl1	fosl2	http://www.ncbi.nlm.nih.gov/entrez/query.fcgi?cmd=Retrieve&db=pubmed&dopt=Abstract&list_uids=27604827|6|Prostate|||Sep|2016|JOURNAL ARTICLE
fosb	fosl2	http://www.ncbi.nlm.nih.gov/entrez/query.fcgi?cmd=Retrieve&db=pubmed&dopt=Abstract&list_uids=27604827|6|Prostate|||Sep|2016|JOURNAL ARTICLE
fosb	fosl1	http://www.ncbi.nlm.nih.gov/entrez/query.fcgi?cmd=Retrieve&db=pubmed&dopt=Abstract&list_uids=27604827|6|Prostate|||Sep|2016|JOURNAL ARTICLE
jund	fosl2	http://www.ncbi.nlm.nih.gov/entrez/query.fcgi?cmd=Retrieve&db=pubmed&dopt=Abstract&list_uids=27604827|6|Prostate|||Sep|2016|JOURNAL ARTICLE

jund	fosl1	http://www.ncbi.nlm.nih.gov/entrez/query.fcgi?cmd=Retrieve&db=pubmed&dopt=Abstract&list_uids=27604827|6|Prostate|||Sep|2016|JOURNAL ARTICLE
jund	fosb	http://www.ncbi.nlm.nih.gov/entrez/query.fcgi?cmd=Retrieve&db=pubmed&dopt=Abstract&list_uids=27604827|6|Prostate|||Sep|2016|JOURNAL ARTICLE
junb	fosl2	http://www.ncbi.nlm.nih.gov/entrez/query.fcgi?cmd=Retrieve&db=pubmed&dopt=Abstract&list_uids=27604827|6|Prostate|||Sep|2016|JOURNAL ARTICLE
junb	fosl1	http://www.ncbi.nlm.nih.gov/entrez/query.fcgi?cmd=Retrieve&db=pubmed&dopt=Abstract&list_uids=27604827|6|Prostate|||Sep|2016|JOURNAL ARTICLE
junb	fosb	http://www.ncbi.nlm.nih.gov/entrez/query.fcgi?cmd=Retrieve&db=pubmed&dopt=Abstract&list_uids=27604827|6|Prostate|||Sep|2016|JOURNAL ARTICLE
junb	jund	http://www.ncbi.nlm.nih.gov/entrez/query.fcgi?cmd=Retrieve&db=pubmed&dopt=Abstract&list_uids=27604827|6|Prostate|||Sep|2016|JOURNAL ARTICLE
stat5a	akt1	http://www.ncbi.nlm.nih.gov/entrez/query.fcgi?cmd=Retrieve&db=pubmed&dopt=Abstract&list_uids=27605043|6|Sci Rep|6|||2016|Journal Article
ctd	4.1.2.13	http://www.ncbi.nlm.nih.gov/entrez/query.fcgi?cmd=Retrieve&db=pubmed&dopt=Abstract&list_uids=27607074|6|PLoS One|11|9||2016|Journal Article
ctd	trap	http://www.ncbi.nlm.nih.gov/entrez/query.fcgi?cmd=Retrieve&db=pubmed&dopt=Abstract&list_uids=27607074|6|PLoS One|11|9||2016|Journal Article
rhce	4.1.2.13	http://www.ncbi.nlm.nih.gov/entrez/query.fcgi?cmd=Retrieve&db=pubmed&dopt=Abstract&list_uids=27607074|6|PLoS One|11|9||2016|Journal Article
trap	4.1.2.13	http://www.ncbi.nlm.nih.gov/entrez/query.fcgi?cmd=Retrieve&db=pubmed&dopt=Abstract&list_uids=27607074|6|PLoS One|11|9||2016|Journal Article
nos2	s100a4	http://www.ncbi.nlm.nih.gov/entrez/query.fcgi?cmd=Retrieve&db=pubmed&dopt=Abstract&list_uids=27608331|6|Photochem Photobiol|||Sep|2016|JOURNAL ARTICLE
phactr4	hcc	http://www.ncbi.nlm.nih.gov/entrez/query.fcgi?cmd=Retrieve&db=pubmed&dopt=Abstract&list_uids=27608898|6|Eur Rev Med Pharmacol Sci|20|16|Aug|2016|Journal Article
phactr4	ppa1	http://www.ncbi.nlm.nih.gov/entrez/query.fcgi?cmd=Retrieve&db=pubmed&dopt=Abstract&list_uids=27608898|6|Eur Rev Med Pharmacol Sci|20|16|Aug|2016|Journal Article
phactr4	stat3	http://www.ncbi.nlm.nih.gov/entrez/query.fcgi?cmd=Retrieve&db=pubmed&dopt=Abstract&list_uids=27608898|6|Eur Rev Med Pharmacol Sci|20|16|Aug|2016|Journal Article
phactr4	il6	http://www.ncbi.nlm.nih.gov/entrez/query.fcgi?cmd=Retrieve&db=pubmed&dopt=Abstract&list_uids=27608898|6|Eur Rev Med Pharmacol Sci|20|16|Aug|2016|Journal Article
ddx6	egf	http://www.ncbi.nlm.nih.gov/entrez/query.fcgi?cmd=Retrieve&db=pubmed&dopt=Abstract&list_uids=27609774|6|FASEB J|||Sep|2016|JOURNAL ARTICLE
anxa1	itk	http://www.ncbi.nlm.nih.gov/entrez/query.fcgi?cmd=Retrieve&db=pubmed&dopt=Abstract&list_uids=27612479|6|Tumour Biol|||Sep|2016|JOURNAL ARTICLE

mamld1	smug1	http://www.ncbi.nlm.nih.gov/entrez/query.fcgi?cmd=Retrieve&db=pubmed&dopt=Abstract&list_uids=27613996|6|Br Med Bull|||Sep|2016|REVIEW,JOURNAL ARTICLE

List of invasion-associated proteins identified using GLAD4U

**Rank**	**Gene ID**	**Gene symbol**	**Specie**	**Score**
1	4318	MMP9	Homo sapiens	306.2129
2	4313	MMP2	Homo sapiens	259.7701
3	999	CDH1	Homo sapiens	189.38
4	4323	MMP14	Homo sapiens	121.2408
5	682	BSG	Homo sapiens	103.4729
6	4233	MET	Homo sapiens	97.85923
7	6615	SNAI1	Homo sapiens	81.93426
8	5328	PLAU	Homo sapiens	79.41798
9	5329	PLAUR	Homo sapiens	57.47993
10	6591	SNAI2	Homo sapiens	55.29275
11	5747	PTK2	Homo sapiens	54.31059
12	10855	HPSE	Homo sapiens	53.13603
13	960	CD44	Homo sapiens	52.70118
14	7074	TIAM1	Homo sapiens	52.15575
15	7291	TWIST1	Homo sapiens	50.8607

16	8434	RECK	Homo sapiens	47.77837
17	5268	SERPINB5	Homo sapiens	45.28517
18	4312	MMP1	Homo sapiens	45.05728
19	4316	MMP7	Homo sapiens	44.68947
20	6935	ZEB1	Homo sapiens	44.40258
21	3688	ITGB1	Homo sapiens	43.46699
22	1499	CTNNB1	Homo sapiens	42.20405
23	6275	S100A4	Homo sapiens	40.96154
24	406937	MIR145	Homo sapiens	38.99991
25	406991	MIR21	Homo sapiens	37.38722
26	5879	RAC1	Homo sapiens	36.44673
27	92140	MTDH	Homo sapiens	36.32073
28	7852	CXCR4	Homo sapiens	35.27336
29	3091	HIF1A	Homo sapiens	35.15325
30	7077	TIMP2	Homo sapiens	33.38051
31	389	RHOC	Homo sapiens	32.12575
32	6624	FSCN1	Homo sapiens	30.56068
33	10630	PDPN	Homo sapiens	30.48908
34	6696	SPP1	Homo sapiens	30.11507
35	7430	EZR	Homo sapiens	29.41737
36	387	RHOA	Homo sapiens	26.8353
37	11156	PTP4A3	Homo sapiens	26.36996
38	7424	VEGFC	Homo sapiens	26.35193
39	2305	FOXM1	Homo sapiens	26.09057
40	3082	HGF	Homo sapiens	26.00846
41	9839	ZEB2	Homo sapiens	25.61254
42	1000	CDH2	Homo sapiens	25.47979
43	3918	LAMC2	Homo sapiens	25.09343
44	2017	CTTN	Homo sapiens	23.94507
45	6774	STAT3	Homo sapiens	23.86445

46	406903	MIR10B	Homo sapiens	23.51674
47	9112	MTA1	Homo sapiens	22.86938
48	6387	CXCL12	Homo sapiens	22.62813
49	5743	PTGS2	Homo sapiens	21.97044
50	4830	NME1	Homo sapiens	21.95506
51	3685	ITGAV	Homo sapiens	21.94147
52	3611	ILK	Homo sapiens	21.79725
53	7076	TIMP1	Homo sapiens	21.58898
54	2191	FAP	Homo sapiens	21.08628
55	100124700	HOTAIR	Homo sapiens	20.63441
56	5037	PEBP1	Homo sapiens	20.23388
57	3814	KISS1	Homo sapiens	20.03252
58	6678	SPARC	Homo sapiens	19.49895
59	207	AKT1	Homo sapiens	19.3308
60	2146	EZH2	Homo sapiens	19.02944
61	5728	PTEN	Homo sapiens	18.98822
62	977	CD151	Homo sapiens	18.95758
63	2064	ERBB2	Homo sapiens	17.95825
64	3678	ITGA5	Homo sapiens	17.88653
65	558	AXL	Homo sapiens	17.80712
66	1956	EGFR	Homo sapiens	17.74661
67	406985	MIR200C	Homo sapiens	17.50817
68	6093	ROCK1	Homo sapiens	17.28745
69	4322	MMP13	Homo sapiens	17.27934
70	407000	MIR218-1	Homo sapiens	17.16913
71	407040	MIR34A	Homo sapiens	17.07468
72	3675	ITGA3	Homo sapiens	16.88695
73	407035	MIR31	Homo sapiens	16.75173
74	7980	TFPI2	Homo sapiens	15.92418
75	3691	ITGB4	Homo sapiens	15.33351

76	27250	PDCD4	Homo sapiens	14.97916
77	7422	VEGFA	Homo sapiens	14.68825
78	4486	MST1R	Homo sapiens	14.55795
79	6714	SRC	Homo sapiens	14.54304
80	7431	VIM	Homo sapiens	14.38527
81	346389	MACC1	Homo sapiens	14.18072
82	56547	MMP26	Homo sapiens	14.16503
83	2113	ETS1	Homo sapiens	14.09341
84	406984	MIR200B	Homo sapiens	14.00377
85	4739	NEDD9	Homo sapiens	14.00338
86	406983	MIR200A	Homo sapiens	13.92346
87	4851	NOTCH1	Homo sapiens	13.80669
88	2735	GLI1	Homo sapiens	13.75057
89	10631	POSTN	Homo sapiens	13.58019
90	100133941	CD24	Homo sapiens	13.56305
91	7474	WNT5A	Homo sapiens	13.51644
92	1500	CTNND1	Homo sapiens	13.39048
93	3655	ITGA6	Homo sapiens	13.32952
94	1969	EPHA2	Homo sapiens	13.27697
95	406913	MIR126	Homo sapiens	12.76673
96	4017	LOXL2	Homo sapiens	12.70852
97	3068	HDGF	Homo sapiens	12.69243
98	11279	KLF8	Homo sapiens	12.56066
99	1236	CCR7	Homo sapiens	12.55289
100	2118	ETV4	Homo sapiens	12.21893
101	100642175	SPRY4-IT1	Homo sapiens	12.08939
102	4582	MUC1	Homo sapiens	12.04041
103	407026	MIR29C	Homo sapiens	11.83173
104	857	CAV1	Homo sapiens	11.78358
105	6768	ST14	Homo sapiens	11.64284

106	3732	CD82	Homo sapiens	11.53079
107	4015	LOX	Homo sapiens	11.38205
108	406958	MIR182	Homo sapiens	11.35229
109	7040	TGFB1	Homo sapiens	11.15337
110	10397	NDRG1	Homo sapiens	11.06278
111	378938	MALAT1	Homo sapiens	10.86058
112	3480	IGF1R	Homo sapiens	10.83799
113	442904	MIR335	Homo sapiens	10.81866
114	10810	WASF3	Homo sapiens	10.81866
115	115908	CTHRC1	Homo sapiens	10.38023
116	1508	CTSB	Homo sapiens	10.37263
117	5829	PXN	Homo sapiens	10.34036
118	4314	MMP3	Homo sapiens	10.27504
119	3984	LIMK1	Homo sapiens	10.26033
120	56649	TMPRSS4	Homo sapiens	10.19158
121	407011	MIR23B	Homo sapiens	10.15155
122	5058	PAK1	Homo sapiens	10.07164
123	768	CA9	Homo sapiens	9.984308
124	406933	MIR141	Homo sapiens	9.969517
125	2149	F2R	Homo sapiens	9.866804
126	3897	L1CAM	Homo sapiens	9.780317
127	407009	MIR224	Homo sapiens	9.645644
128	574447	MIR146B	Homo sapiens	9.391292
129	406987	MIR204	Homo sapiens	9.252239
130	7078	TIMP3	Homo sapiens	9.241503
131	4288	MKI67	Homo sapiens	9.118286
132	4162	MCAM	Homo sapiens	9.081684
133	406893	MIR101-1	Homo sapiens	8.798243
134	57007	ACKR3	Homo sapiens	8.753263
135	976	ADGRE5	Homo sapiens	8.687883

136	118856	MMP21	Homo sapiens	8.492416
137	406907	MIR124-1	Homo sapiens	8.490562
138	673	BRAF	Homo sapiens	8.353496
139	3397	ID1	Homo sapiens	8.349009
140	998	CDC42	Homo sapiens	8.328811
141	406959	MIR183	Homo sapiens	8.325047
142	84634	KISS1R	Homo sapiens	8.319463
143	407006	MIR221	Homo sapiens	8.31054
144	9891	NUAK1	Homo sapiens	8.200702
145	9564	BCAR1	Homo sapiens	8.18904
146	9076	CLDN1	Homo sapiens	8.151938
147	406935	MIR143	Homo sapiens	8.023444
148	4325	MMP16	Homo sapiens	7.95668
149	9788	MTSS1	Homo sapiens	7.95668
150	6382	SDC1	Homo sapiens	7.893165
151	406922	MIR133A1	Homo sapiens	7.840345
152	406986	MIR203A	Homo sapiens	7.836954
153	4249	MGAT5	Homo sapiens	7.836181
154	4838	NODAL	Homo sapiens	7.79663
155	406936	MIR144	Homo sapiens	7.676461
156	1015	CDH17	Homo sapiens	7.540654
157	406996	MIR214	Homo sapiens	7.525747
158	8754	ADAM9	Homo sapiens	7.525747
159	4072	EPCAM	Homo sapiens	7.40065
160	1950	EGF	Homo sapiens	7.37619
161	442890	MIR133B	Homo sapiens	7.303364
162	648	BMI1	Homo sapiens	7.273169
163	6286	S100P	Homo sapiens	7.101097
164	407014	MIR25	Homo sapiens	7.079069
165	407007	MIR222	Homo sapiens	7.041421

166	55704	CCDC88A	Homo sapiens	7.006685
167	4790	NFKB1	Homo sapiens	6.968999
168	2115	ETV1	Homo sapiens	6.958557
169	4320	MMP11	Homo sapiens	6.912997
170	406931	MIR139	Homo sapiens	6.862791
171	57447	NDRG2	Homo sapiens	6.861141
172	407024	MIR29B1	Homo sapiens	6.821348
173	302	ANXA2	Homo sapiens	6.79961
174	406929	MIR138-1	Homo sapiens	6.725651
175	406988	MIR205	Homo sapiens	6.68752
176	8061	FOSL1	Homo sapiens	6.68752
177	574456	MIR497	Homo sapiens	6.634509
178	214	ALCAM	Homo sapiens	6.465377
179	390	RND3	Homo sapiens	6.38016
180	6868	ADAM17	Homo sapiens	6.376775
181	574411	MIR451A	Homo sapiens	6.289733
182	6304	SATB1	Homo sapiens	6.266575
183	780	DDR1	Homo sapiens	6.252663
184	406947	MIR155	Homo sapiens	6.250332
185	117581	TWIST2	Homo sapiens	6.226705
186	8614	STC2	Homo sapiens	6.127878
187	406999	MIR217	Homo sapiens	6.119768
188	406969	MIR194-1	Homo sapiens	6.119768
189	2050	EPHB4	Homo sapiens	6.013755
190	406989	MIR206	Homo sapiens	5.947932
191	50807	ASAP1	Homo sapiens	5.937972
192	94234	FOXQ1	Homo sapiens	5.927381
193	494324	MIR375	Homo sapiens	5.856482
194	3956	LGALS1	Homo sapiens	5.841407
195	8842	PROM1	Homo sapiens	5.837365

196	9314	KLF4	Homo sapiens	5.789629
197	4319	MMP10	Homo sapiens	5.756291
198	634	CEACAM1	Homo sapiens	5.693478
199	9922	IQSEC1	Homo sapiens	5.683466
200	1793	DOCK1	Homo sapiens	5.647817
201	174	AFP	Homo sapiens	5.55101
202	3589	IL11	Homo sapiens	5.497617
203	407015	MIR26A1	Homo sapiens	5.449901
204	406993	MIR211	Homo sapiens	5.439844
205	407050	MIR93	Homo sapiens	5.422545
206	10276	NET1	Homo sapiens	5.402262
207	406901	MIR107	Homo sapiens	5.402262
208	5584	PRKCI	Homo sapiens	5.391294
209	864	RUNX3	Homo sapiens	5.381679
210	2335	FN1	Homo sapiens	5.370334
211	10395	DLC1	Homo sapiens	5.331209
212	80381	CD276	Homo sapiens	5.323485
213	10763	NES	Homo sapiens	5.282806
214	128209	KLF17	Homo sapiens	5.277343
215	407010	MIR23A	Homo sapiens	5.266445
216	406900	MIR106B	Homo sapiens	5.266445
217	64866	CDCP1	Homo sapiens	5.266445
218	25803	SPDEF	Homo sapiens	5.225041
219	9475	ROCK2	Homo sapiens	5.14174
220	4089	SMAD4	Homo sapiens	5.086116
221	406902	MIR10A	Homo sapiens	5.050012
222	11080	DNAJB4	Homo sapiens	5.050012
223	1400	CRMP1	Homo sapiens	5.050012
224	3371	TNC	Homo sapiens	5.005495
225	27022	FOXD3	Homo sapiens	4.980562

226	2119	ETV5	Homo sapiens	4.974341
227	4915	NTRK2	Homo sapiens	4.963022
228	1001	CDH3	Homo sapiens	4.957899
229	407001	MIR218-2	Homo sapiens	4.844367
230	10413	YAP1	Homo sapiens	4.827238
231	8091	HMGA2	Homo sapiens	4.803443
232	407021	MIR29A	Homo sapiens	4.786356
233	406943	MIR152	Homo sapiens	4.777404
234	406976	MIR199A1	Homo sapiens	4.771018
235	23705	CADM1	Homo sapiens	4.765346
236	1364	CLDN4	Homo sapiens	4.7412
237	822	CAPG	Homo sapiens	4.691926
238	5595	MAPK3	Homo sapiens	4.650426
239	723788	MIG7	Homo sapiens	4.649835
240	442918	MIR373	Homo sapiens	4.60389
241	64083	GOLPH3	Homo sapiens	4.601471
242	6548	SLC9A1	Homo sapiens	4.59431
243	6659	SOX4	Homo sapiens	4.59385
244	10298	PAK4	Homo sapiens	4.59385
245	6623	SNCG	Homo sapiens	4.543499
246	407029	MIR30A	Homo sapiens	4.518419
247	102	ADAM10	Homo sapiens	4.512039
248	554210	MIR429	Homo sapiens	4.508486
249	9644	SH3PXD2A	Homo sapiens	4.508486
250	57650	KIAA1524	Homo sapiens	4.493878
251	9166	EBAG9	Homo sapiens	4.489103
252	406982	MIR20A	Homo sapiens	4.489103
253	406925	MIR135A1	Homo sapiens	4.475803
254	406971	MIR195	Homo sapiens	4.463543
255	397	ARHGDIB	Homo sapiens	4.458293

256	3236	HOXD10	Homo sapiens	4.44983
257	574434	MIR410	Homo sapiens	4.441607
258	1012	CDH13	Homo sapiens	4.438936
259	407055	MIR99A	Homo sapiens	4.416415
260	2185	PTK2B	Homo sapiens	4.398556
261	3880	KRT19	Homo sapiens	4.397005
262	114793	FMNL2	Homo sapiens	4.364401
263	6692	SPINT1	Homo sapiens	4.331723
264	51330	TNFRSF12A	Homo sapiens	4.330851
265	3921	RPSA	Homo sapiens	4.311075
266	388	RHOB	Homo sapiens	4.258058
267	9048	ARTN	Homo sapiens	4.257955
268	3694	ITGB6	Homo sapiens	4.229513
269	406940	MIR148A	Homo sapiens	4.228797
270	407043	MIR7-1	Homo sapiens	4.226964
271	6657	SOX2	Homo sapiens	4.212101
272	3373	HYAL1	Homo sapiens	4.205571
273	407042	MIR34C	Homo sapiens	4.205571
274	5168	ENPP2	Homo sapiens	4.174488
275	442891	MIR135B	Homo sapiens	4.156082
276	3958	LGALS3	Homo sapiens	4.147597
277	208	AKT2	Homo sapiens	4.136138
278	406979	MIR19A	Homo sapiens	4.087835
279	10553	HTATIP2	Homo sapiens	4.087835
280	57575	PCDH10	Homo sapiens	4.077736
281	494327	MIR378A	Homo sapiens	4.077736
282	25855	BRMS1	Homo sapiens	4.067349
283	3925	STMN1	Homo sapiens	4.049145
284	84299	MIEN1	Homo sapiens	4.042094
285	80310	PDGFD	Homo sapiens	4.020866

286	6997	TDGF1	Homo sapiens	4.015414
287	84740	AFAP1-AS1	Homo sapiens	4.005745
288	1748	DLX4	Homo sapiens	3.999665
289	406961	MIR185	Homo sapiens	3.999665
290	81839	VANGL1	Homo sapiens	3.96474
291	1474	CST6	Homo sapiens	3.923957
292	10643	IGF2BP3	Homo sapiens	3.923015
293	55740	ENAH	Homo sapiens	3.914209
294	7057	THBS1	Homo sapiens	3.895798
295	64754	SMYD3	Homo sapiens	3.891772
296	4904	YBX1	Homo sapiens	3.889852
297	7593	MZF1	Homo sapiens	3.874696
298	3914	LAMB3	Homo sapiens	3.864893
299	406910	MIR125A	Homo sapiens	3.8574
300	51162	EGFL7	Homo sapiens	3.850493
301	2797	GNRH2	Homo sapiens	3.829518
302	407004	MIR22	Homo sapiens	3.803362
303	574455	MIR193B	Homo sapiens	3.78807
304	407037	MIR320A	Homo sapiens	3.78807
305	694	BTG1	Homo sapiens	3.779162
306	9122	SLC16A4	Homo sapiens	3.709309
307	7033	TFF3	Homo sapiens	3.708979
308	5054	SERPINE1	Homo sapiens	3.700701
309	3037	HAS2	Homo sapiens	3.698391
310	9547	CXCL14	Homo sapiens	3.647394
311	8190	MIA	Homo sapiens	3.642492
312	9232	PTTG1	Homo sapiens	3.641644
313	5918	RARRES1	Homo sapiens	3.605704
314	332	BIRC5	Homo sapiens	3.602816
315	4921	DDR2	Homo sapiens	3.59735

316	11082	ESM1	Homo sapiens	3.576969
317	7764	ZNF217	Homo sapiens	3.576969
318	6386	SDCBP	Homo sapiens	3.56011
319	10232	MSLN	Homo sapiens	3.558373
320	693210	MIR625	Homo sapiens	3.553478
321	2048	EPHB2	Homo sapiens	3.539078
322	406955	MIR181B1	Homo sapiens	3.513208
323	27076	LYPD3	Homo sapiens	3.509775
324	442906	MIR338	Homo sapiens	3.509775
325	406909	MIR124-3	Homo sapiens	3.509775
326	3491	CYR61	Homo sapiens	3.478649
327	3324	HSP90AA2P	Homo sapiens	3.418244
328	1398	CRK	Homo sapiens	3.410513
329	407008	MIR223	Homo sapiens	3.404658
330	6469	SHH	Homo sapiens	3.403953
331	3696	ITGB8	Homo sapiens	3.399161
332	382	ARF6	Homo sapiens	3.397028
333	55272	IMP3	Homo sapiens	3.390667
334	664616	MIR487B	Homo sapiens	3.369587
335	407027	MIR301A	Homo sapiens	3.36702
336	2995	GYPC	Homo sapiens	3.329054
337	406972	MIR196A1	Homo sapiens	3.329054
338	29956	CERS2	Homo sapiens	3.329054
339	3934	LCN2	Homo sapiens	3.316045
340	51147	ING4	Homo sapiens	3.31562
341	5594	MAPK1	Homo sapiens	3.312473
342	2078	ERG	Homo sapiens	3.310772
343	406892	MIR100	Homo sapiens	3.278626
344	1045	CDX2	Homo sapiens	3.273383
345	406914	MIR127	Homo sapiens	3.254939

346	374	AREG	Homo sapiens	3.24908
347	406920	MIR130B	Homo sapiens	3.247044
348	3161	HMMR	Homo sapiens	3.228197
349	1366	CLDN7	Homo sapiens	3.228197
350	407053	MIR96	Homo sapiens	3.218286
351	1009	CDH11	Homo sapiens	3.218286
352	3249	HPN	Homo sapiens	3.218286
353	83998	REG4	Homo sapiens	3.218286
354	10979	FERMT2	Homo sapiens	3.218286
355	406954	MIR181A2	Homo sapiens	3.195422
356	7216	TRO	Homo sapiens	3.195422
357	8826	IQGAP1	Homo sapiens	3.189224
358	2277	FIGF	Homo sapiens	3.186696
359	25805	BAMBI	Homo sapiens	3.166797
360	407012	MIR24-1	Homo sapiens	3.16363
361	406941	MIR149	Homo sapiens	3.16363
362	406894	MIR101-2	Homo sapiens	3.149685
363	23213	SULF1	Homo sapiens	3.137625
364	2303	FOXC2	Homo sapiens	3.137625
365	22943	DKK1	Homo sapiens	3.127957
366	1839	HBEGF	Homo sapiens	3.123133
367	60672	MIIP	Homo sapiens	3.058553
368	406904	MIR1-1	Homo sapiens	3.0582
369	25794	FSCN2	Homo sapiens	3.050544
370	442917	MIR372	Homo sapiens	3.050544
371	8626	TP63	Homo sapiens	3.042269
372	360	AQP3	Homo sapiens	3.027719
373	7471	WNT1	Homo sapiens	3.015178
374	407019	MIR27B	Homo sapiens	3.009794
375	23566	LPAR3	Homo sapiens	3.009794

376	8838	WISP3	Homo sapiens	3.009794
377	2736	GLI2	Homo sapiens	2.992129
378	5587	PRKD1	Homo sapiens	2.988826
379	5290	PIK3CA	Homo sapiens	2.977318
380	1514	CTSL	Homo sapiens	2.97562
381	9043	SPAG9	Homo sapiens	2.957626
382	1902	LPAR1	Homo sapiens	2.956111
383	56287	GKN1	Homo sapiens	2.944613
384	574506	MIR503	Homo sapiens	2.944613
385	494326	MIR377	Homo sapiens	2.92478
386	100126333	MIR708	Homo sapiens	2.92478
387	2202	EFEMP1	Homo sapiens	2.922451
388	290	ANPEP	Homo sapiens	2.921374
389	406995	MIR181A1	Homo sapiens	2.90905
390	4585	MUC4	Homo sapiens	2.905419
391	101101692	HELLPAR	Homo sapiens	2.900425
392	3309	HSPA5	Homo sapiens	2.893508
393	2532	ACKR1	Homo sapiens	2.890059
394	5732	PTGER2	Homo sapiens	2.890059
395	3146	HMGB1	Homo sapiens	2.871603
396	406997	MIR215	Homo sapiens	2.868259
397	79444	BIRC7	Homo sapiens	2.854708
398	860	RUNX2	Homo sapiens	2.847517
399	5731	PTGER1	Homo sapiens	2.839745
400	3910	LAMA4	Homo sapiens	2.839745
401	2065	ERBB3	Homo sapiens	2.83248
402	7049	TGFBR3	Homo sapiens	2.828583
403	396	ARHGDIA	Homo sapiens	2.828583
404	5909	RAP1GAP	Homo sapiens	2.815119
405	574511	MIR506	Homo sapiens	2.80242

406	406944	MIR153-1	Homo sapiens	2.80242
407	3911	LAMA5	Homo sapiens	2.796483
408	1942	EFNA1	Homo sapiens	2.790416
409	6695	SPOCK1	Homo sapiens	2.784143
410	2022	ENG	Homo sapiens	2.766126
411	406928	MIR137	Homo sapiens	2.75087
412	406977	MIR199A2	Homo sapiens	2.746291
413	2852	GPER1	Homo sapiens	2.720425
414	4478	MSN	Homo sapiens	2.714684
415	3159	HMGA1	Homo sapiens	2.70453
416	406924	MIR134	Homo sapiens	2.704171
417	206338	LVRN	Homo sapiens	2.689804
418	574413	MIR409	Homo sapiens	2.689804
419	406930	MIR138-2	Homo sapiens	2.689804
420	7803	PTP4A1	Homo sapiens	2.68324
421	724025	MIR655	Homo sapiens	2.6608
422	5734	PTGER4	Homo sapiens	2.630793
423	51754	TMEM8B	Homo sapiens	2.627999
424	157310	PEBP4	Homo sapiens	2.627999
425	11178	LZTS1	Homo sapiens	2.627999
426	406899	MIR106A	Homo sapiens	2.625301
427	407018	MIR27A	Homo sapiens	2.601615
428	6241	RRM2	Homo sapiens	2.599595
429	4856	NOV	Homo sapiens	2.599595
430	407036	MIR32	Homo sapiens	2.585605
431	406946	MIR154	Homo sapiens	2.585605
432	29775	CARD10	Homo sapiens	2.567801
433	5294	PIK3CG	Homo sapiens	2.561318
434	8549	LGR5	Homo sapiens	2.558574
435	4311	MME	Homo sapiens	2.548481

436	9181	ARHGEF2	Homo sapiens	2.527854
437	1365	CLDN3	Homo sapiens	2.527854
438	51176	LEF1	Homo sapiens	2.510096
439	574030	MIR362	Homo sapiens	2.496305
440	84513	PLPP5	Homo sapiens	2.496305
441	26018	LRIG1	Homo sapiens	2.492804
442	7031	TFF1	Homo sapiens	2.489443
443	84651	SPINK7	Homo sapiens	2.48875
444	100033819	MIR675	Homo sapiens	2.48875
445	2012	EMP1	Homo sapiens	2.485881
446	10103	TSPAN1	Homo sapiens	2.485881
447	1890	TYMP	Homo sapiens	2.47975
448	4321	MMP12	Homo sapiens	2.47975
449	928	CD9	Homo sapiens	2.46684
450	27122	DKK3	Homo sapiens	2.464062
451	362	AQP5	Homo sapiens	2.458538
452	406912	MIR125B2	Homo sapiens	2.458269
453	8839	WISP2	Homo sapiens	2.458269
454	574452	MIR494	Homo sapiens	2.458269
455	102723099	GHET1	Homo sapiens	2.433694
456	10163	WASF2	Homo sapiens	2.424752
457	5768	QSOX1	Homo sapiens	2.406051
458	6493	SIM2	Homo sapiens	2.406051
459	9170	LPAR2	Homo sapiens	2.404497
460	6862	T	Homo sapiens	2.404497
461	442909	MIR342	Homo sapiens	2.39836
462	2150	F2RL1	Homo sapiens	2.388111
463	407041	MIR34B	Homo sapiens	2.361921
464	5284	PIGR	Homo sapiens	2.358851
465	56998	CTNNBIP1	Homo sapiens	2.358851

466	406968	MIR193A	Homo sapiens	2.355758
467	406890	MIRLET7G	Homo sapiens	2.355758
468	57111	RAB25	Homo sapiens	2.35542
469	9902	MRC2	Homo sapiens	2.35542
470	406970	MIR194-2	Homo sapiens	2.352978
471	693215	MIR630	Homo sapiens	2.352978
472	2296	FOXC1	Homo sapiens	2.344331
473	9219	MTA2	Homo sapiens	2.344331
474	406911	MIR125B1	Homo sapiens	2.326711
475	9353	SLIT2	Homo sapiens	2.320722
476	10653	SPINT2	Homo sapiens	2.320345
477	574453	MIR495	Homo sapiens	2.313706
478	56667	MUC13	Homo sapiens	2.313706
479	11149	BVES	Homo sapiens	2.313706
480	4092	SMAD7	Homo sapiens	2.289219
481	1803	DPP4	Homo sapiens	2.275142
482	79923	NANOG	Homo sapiens	2.261687
483	1495	CTNNA1	Homo sapiens	2.241021
484	5364	PLXNB1	Homo sapiens	2.24005
485	7481	WNT11	Homo sapiens	2.236009
486	4324	MMP15	Homo sapiens	2.236009
487	406966	MIR191	Homo sapiens	2.236009
488	407044	MIR7-2	Homo sapiens	2.234175
489	10551	AGR2	Homo sapiens	2.233378
490	374907	B3GNT8	Homo sapiens	2.226227
491	286205	SCAI	Homo sapiens	2.226227
492	51079	NDUFA13	Homo sapiens	2.223516
493	9077	DIRAS3	Homo sapiens	2.212309
494	5754	PTK7	Homo sapiens	2.212309
495	406992	MIR210	Homo sapiens	2.203982

496	1462	VCAN	Homo sapiens	2.194625
497	10006	ABI1	Homo sapiens	2.18995
498	599	BCL2L2	Homo sapiens	2.18995
499	6370	CCL25	Homo sapiens	2.179604
500	9423	NTN1	Homo sapiens	2.173615
501	8099	CDK2AP1	Homo sapiens	2.167311
502	23326	USP22	Homo sapiens	2.163347
503	652995	UCA1	Homo sapiens	2.159247
504	407045	MIR7-3	Homo sapiens	2.159247
505	101669767	SCHLAP1	Homo sapiens	2.143023
506	3673	ITGA2	Homo sapiens	2.134016
507	2034	EPAS1	Homo sapiens	2.132863
508	4583	MUC2	Homo sapiens	2.126938
509	406994	MIR212	Homo sapiens	2.12532
510	406884	MIRLET7B	Homo sapiens	2.123566
511	10803	CCR9	Homo sapiens	2.123566
512	5641	LGMN	Homo sapiens	2.123566
513	7113	TMPRSS2	Homo sapiens	2.12029
514	693175	MIR590	Homo sapiens	2.112819
515	101867536	PRNCR1	Homo sapiens	2.112819
516	5045	FURIN	Homo sapiens	2.109984
517	23568	ARL2BP	Homo sapiens	2.088478
518	26136	TES	Homo sapiens	2.08102
519	6667	SP1	Homo sapiens	2.080739
520	406915	MIR128-1	Homo sapiens	2.073028
521	56648	EIF5A2	Homo sapiens	2.073028
522	9037	SEMA5A	Homo sapiens	2.073028
523	60495	HPSE2	Homo sapiens	2.073028
524	2444	FRK	Homo sapiens	2.073028
525	6374	CXCL5	Homo sapiens	2.059598

526	2994	GYPB	Homo sapiens	2.039622
527	25937	WWTR1	Homo sapiens	2.031097
528	5460	POU5F1	Homo sapiens	2.030623
529	6525	SMTN	Homo sapiens	2.022612
530	3590	IL11RA	Homo sapiens	2.022612
531	27121	DKK4	Homo sapiens	2.021484
532	64101	LRRC4	Homo sapiens	2.021484
533	7162	TPBG	Homo sapiens	2.021484
534	406918	MIR129-2	Homo sapiens	2.021484
535	407047	MIR9-2	Homo sapiens	2.021484
536	1602	DACH1	Homo sapiens	2.019785
537	8840	WISP1	Homo sapiens	2.019785
538	2919	CXCL1	Homo sapiens	2.01948
539	3791	KDR	Homo sapiens	2.010623
540	50859	SPOCK3	Homo sapiens	2.010382
541	55200	PLEKHG6	Homo sapiens	2.010382
542	494330	MIR381	Homo sapiens	2.010382
543	946	SIGLEC6	Homo sapiens	2.010382
544	442907	MIR339	Homo sapiens	2.010382

List of invadopodia-associated proteins identified using ALS

Protein name	Aliases	Specie
snx9	sh3pxd3a|snx9|mst155|mstp155|sh3px1|wisp	Homo sapiens
wipf2	wipf2|wich|wire	Homo sapiens
pdgfb	pdgf-b|flj12858|pdgfb|pdgf2|c-sis|sis|ssv	Homo sapiens
erbb2	erbb2|her2|her-2|cd340|neu|ngl|tkr1|her-2/neu	Homo sapiens
glyat	acgnat|gat|glyat	Homo sapiens
tom1l1	tom1l1|ok/kns-cl.3|srcasm	Homo sapiens
tollip	flj33531|tollip|il-1racpip	Homo sapiens
gdi1	gdi1|gdil|ophn2|xap-4|mrx48|mrx41|rabgdia|rabgd1a|flj41411|gdi-1	Homo sapiens
agfg1	hrb|rab|rip|mgc116940|mgc116938|dkfzp686i15205|agfg1	Homo sapiens
kiss1r	gpr54|hot7t175|kiss1r|axor12	Homo sapiens
inf2	mgc13251|c14orf151|c14orf173|inf2|flj22056|pp9484|dkfzp762a0214	Homo sapiens
skap2	skap-hom|mgc33304|prap|ra70|skap55r|saps|skap2|mgc10411|scap2	Homo sapiens
s100b	nef|s100beta|s100b	Homo sapiens
fgr	c-fgr|fgr|mgc75096|src2|p58c-fgr|p55c-fgr|flj43153|c-src2	Homo sapiens
abi2	aip-1|ssh3bp2|abi2b|abi-2|ablbp3|abi2|argbpia|argbpib	Homo sapiens
abi3	ssh3bp3|abi-3|abi3|nesh	Homo sapiens
rela	mgc131774|rela|p65|nfkb3	Homo sapiens
foxa2	hnf3b|tcf3b|foxa2|mgc19807	Homo sapiens
cdx2	cdx-3|cdx3|cdx2	Homo sapiens
nkx2-1	titf1|nkx2a|ttf-1|nkx2-1|nkx2.1|bhc|tebp|nk-2	Homo sapiens
zeb1	areb6|zeb1|nil-2-a|zfhx1a|bzp|zeb|mgc133261|delta-ef1|tcf8|nil-2a|zfhep|nil2a	Homo sapiens
erlin2	mgc87072|spfh2|erlin-2|c8orf2|erlin2	Homo sapiens
3.5.1.4	deaminase|acylamidase|3.5.1.4|fatty acylamidase|acylase|amidase|n-acetylaminohydrolase|amidohydrolase	Homo sapiens
prl	prl	Homo sapiens
jak2	jtk10|jak2	Homo sapiens
cdk5rap1	hspc167|c42|cdk5rap1.4|cdk5rap1.3|cdk5rap1|c20orf34|cgi-05	Homo sapiens
cdk5r2	p39nck5ai|nck5ai|p39|cdk5r2	Homo sapiens

psmc6	psmc6|cadp44|p42|p44|sug2|mgc12520	Homo sapiens
cd40	mgc9013|p50|cdw40|tnfrsf5|cd40	Homo sapiens
cd80	cd28lg|cd28lg1|cd80|lab7	Homo sapiens
cd86	mgc34413|cd28lg2|b7-2|b70|cd86|lab72	Homo sapiens
tlr4	cd284|tlr-4|tlr4|toll|armd10|htoll	Homo sapiens
sp1	sp1|sp-1	Homo sapiens
rchy1	arnip|znf363|harnip|rchy1|dkfzp586c1620|pro1996|rnf199|pirh2|chimp	Homo sapiens
siah1	siah-1a|humsiah|hsiah1|siah1|siah-1	Homo sapiens
bmp1	pcolc|pcp-2|pcp|tld|bmp1|flj44432	Homo sapiens
synj1	synj1|inpp5g	Homo sapiens
akt3	pkbg|pkb-gamma|akt3|dkfzp434n0250|stk-2|rac-pk-gamma|rac-gamma|prkbg	Homo sapiens
akt2	pkbb|akt2|rac-beta|pkbbeta|prkbb	Homo sapiens
foxo3	af6q21|foxo3|foxo2|foxo3a|dkfzp781a0677|fkhrl1p2|fkhrl1|mgc31925|mgc12739	Homo sapiens
slc9a3r1	slc9a3r1|nphlop2|ebp50|nherf1|nherf	Homo sapiens
serpinb3	scca-pd|serpinb3|t4-a|scc|scca-1|scca1|hst1196	Homo sapiens
btk	at|mgc126261|mgc126262|imd1|atk|bpk|btk|xla|psctk1|agmx1	Homo sapiens
stim1	gok|d11s4896e|stim1	Homo sapiens
orai1	tmem142a|flj14466|orai1|orat1|cracm1	Homo sapiens
mylk	dkfzp686i10125|mylk1|smmlck|flj12216|krp|mstp083|mlck1|mlck|mlck108|mlck210|mylk	Homo sapiens
clic1	g6|ncc27|clic1	Homo sapiens
fut1	hh|fut1|hsc	Homo sapiens
ctns	ctns-lsb|ctns|pqlc4	Homo sapiens
mirn21	mir-21|mir21|mirna21|mirn21|hsa-mir-21	Homo sapiens
adamts4	adamts-4|adamts-2|admp-1|kiaa0688|adamts4	Homo sapiens
ca5a	ca5|cav|ca5a|cava	Homo sapiens
rap1b	ral1b|rap1b|k-rev|dkfzp586h0723	Homo sapiens
ptk7	ptk7|cck4	Homo sapiens
podxl	pclp|podxl|gp200|mgc138240	Homo sapiens
smad2	mgc34440|hmad-2|madh2|madr2|mgc22139|jv18|jv18-1|smad2	Homo sapiens
hcc	hcc	Homo sapiens

pak1	pak1|pakalpha|mgc130001|mgc130000	Homo sapiens
tp63	ofc8|shfm4|tp53cp|ket|lms|p51|rhs|tp53l|tp73l|tp63|b(p51a)|b(p51b)|p53cp|eec3|p73l|p73h	Homo sapiens
dmd	dxs164|dxs142|dxs230|dxs239|dxs270|dxs272|dxs268|dxs269|dxs206|bmd|dmd|cmd3b	Homo sapiens
bmpr1a	acvrlk3|10q23del|cd292|alk3|bmpr1a	Homo sapiens
bmpr1b	alk-6|alk6|bmpr1b|cdw293	Homo sapiens
bmpr2	bmpr2|bmpr3|t-alk|pph1|bmpr-ii|flj41585|bmr2|brk-3|flj76945	Homo sapiens
smad4	madh4|jip|dpc4|smad4	Homo sapiens
pdlim5	l9|enh|lim|pdlim5|enh1	Homo sapiens
serpinb2	pai2|serpinb2|planh2|hst1201|pai-2	Homo sapiens
nts	nn|nmn-125|nts|nt/n|nts1	Homo sapiens
akr1c4	3-alpha-hsd|c11|dd4|mgc22581|chdr|akr1c4|hakra	Homo sapiens
stx12	stx14|stx13|stx12|mgc51957	Homo sapiens
vav1	vav|vav1	Homo sapiens
mdm2	hdm2|hdmx|mdm2|mgc71221	Homo sapiens
skp2	mgc1366|skp2|fbxl1|fbl1|flb1	Homo sapiens
arap3	arap3|flj21065|centd3|drag1	Homo sapiens
ogt	ogt|mgc22921|o-glcnac|hrnt1|flj23071	Homo sapiens
mlc1	vl|lvm|mlc|mlc1|kiaa0027	Homo sapiens
gmpr	gmpr|gmpr1	Homo sapiens
fhod1	fhod1|fhos	Homo sapiens
muc1	h23ag|cd227|pem|pum|mam6|muc1	Homo sapiens
xrcc1	xrcc1|rcc	Homo sapiens
sts	es|arsc1|asc|sts|arsc|ssdd	Homo sapiens
btg2	tis21|mgc126063|mgc126064|btg2	Homo sapiens
scn5a	hbbd|iccd|hh1|ivf|sss1|cdcd2|cmd1e|pfhb1|cmpd2|nav1.5|lqt3|scn5a	Homo sapiens
bsg	m6|ok|5f7|cd147|bsg|tcsf|emmprin	Homo sapiens
comp	psach|mgc131819|mgc149768|comp|thbs5|edm1|epd1	Homo sapiens
nsmaf	nsmaf|fan	Homo sapiens
braf	mgc126806|rafb1|braf|braf1|flj95109|b-raf1|mgc138284	Homo sapiens
swap70	hspc321|swap70|swap-70|kiaa0640|flj39540	Homo sapiens

pstpip2	mgc34175|pstpip2|mayp	Homo sapiens
tbx1	tbx1c|tga|tbx1|cafs|cthm|vcfs|dorv	Homo sapiens
smad3	dkfzp586n0721|hspc193|dkfzp686j10186|mgc60396|madh3|hst17436|jv15-2|smad3	Homo sapiens
epgn	algv3072|epg|pro9904|epigen|flj75542|epgn	Homo sapiens
hmbs	pbg-d|pbgd|hmbs|ups	Homo sapiens
tnfsf11	opgl|rankl|optb2|cd254|trance|hrankl2|tnfsf11	Homo sapiens
nfatc1	nfatc1|nf-atc|nfat2|nfatc|mgc138448	Homo sapiens
adamts1	kiaa1346|adamts-1|c3-c5|meth1|adamts1	Homo sapiens
ptpn11	mgc14433|ptp2c|ptpn11|cfc|ns1|bptp3|ptp-1d|shp2|sh-ptp3|sh-ptp2	Homo sapiens
hmmr	ihabp|hmmr|cd168|rhamm|mgc119495|mgc119494	Homo sapiens
lsp1	pp52|lsp1|wp34	Homo sapiens
slit2	slit-2|flj14420|slil3|slit2	Homo sapiens
robo1	dutt1|mgc131599|sax3|mgc133277|flj21882|robo1	Homo sapiens
npl	npl|c112|mgc61869|mgc149582|c1orf13|npl1	Homo sapiens
vangl2	kiaa1215|stb1|stbm|vangl2|stbm1|ltap|mgc119404|mgc119403	Homo sapiens
tfap2a	tfap2a|ap-2|bofs|tfap2|ap2tf	Homo sapiens
ctsb	apps|cpsb|ctsb	Homo sapiens
inppl1	ship2|inppl1	Homo sapiens
rhog	mgc125836|mgc125835|rhog|arhg	Homo sapiens
hgf	hgfb|hpta|hgf|f-tcf	Homo sapiens
chrna4	ebn|bfnc|chrna4|nacra4|flj95812|ebn1|nachr|nachra4	Homo sapiens
il10	mgc126451|mgc126450|il10|il-10|il10a|tgif|csif	Homo sapiens
cdkn1a	cap20|p21cip1|p21|sdi1|cdkn1|cip1|mda-6|waf1|cdkn1a	Homo sapiens
tgm2	gnah|g-alpha-h|tg2|tgc|tgm2	Homo sapiens
tgm1	li|icr2|ktg|li1|tgk|tgm1|tgase	Homo sapiens
asf1a	asf1a|hspc146|dkfzp547e2110|cia|cgi-98	Homo sapiens
pik3r1	grb1|p85-alpha|p85|pik3r1	Homo sapiens
exoc7	flj46415|yjl085w|2-5-3p|dkfzp686j04253|flj40965|ex070|exo70p|exoc7|exo70	Homo sapiens
neurog1	neurod3|neurog1|a.k.a|aka|math4c|bhlha6|ngn1	Homo sapiens
tgfb1i1	tgfb1i1|ara55|tsc-5|hic5|hic-5	Homo sapiens

lasp1	lasp-1|mln50|lasp1	Homo sapiens
flna	flna|fln1|opd1|opd2|fln|fmd|abpx|mns|opd|dkfzp434p031|abp-280|nhbp	Homo sapiens
kras	c-k-ras|rask2|ki-ras|kras2|kras1|k-ras4b|k-ras4a|k-ras2a|k-ras2b|ns3|kras	Homo sapiens
ralb	ralb	Homo sapiens
ralbp1	ralbp1|rlip76	Homo sapiens
exoc2	flj11026|sec5|sec5l1|exoc2|sec5p	Homo sapiens
exoc8	exo84p|exoc8|exo84|sec84	Homo sapiens
fas	alps1a|fas|apt1|tnfrsf6|cd95|fastm|apo-1|fas1	Homo sapiens
tyr	shep3|tyr|oca1a|ocaia	Homo sapiens
mt1a	mt1|mtc|mt1a|mt1s|mgc32848	Homo sapiens
igf2	c11orf43|insigf|igf2|flj22066|pp9974|flj44734	Homo sapiens
actb	actb|ps1tp5bp1	Homo sapiens
pten	pten1|pten|bzs|mmac1|mgc11227|mham	Homo sapiens
mapk4	mapk4|p63mapk|erk4|erk3|prkm4	Homo sapiens
hspb1	hmn2b|dkfzp586p1322|srp27|hs.76067|cmt2f|hsp28|hsp27|hsp25|hspb1	Homo sapiens
tagln	tagln|tagln1|ws3-10|sm22|dkfzp686p11128	Homo sapiens
prkaa1	mgc33776|ampk|ampka1|mgc57364|prkaa1	Homo sapiens
acaca	acaca|acc|acac|acc1|acca	Homo sapiens
pc-3	pc-3|psmp	Homo sapiens
canx	flj26570|ip90|cnx|p90|canx	Homo sapiens
rps6ka1	mapkapk1a|rsk1|rsk|rps6ka1	Homo sapiens
hras	n-ras|rash1|c-bas/has|hras|c-ha-ras1|k-ras|c-h-ras|ctlo|hras1|hamsv|h-rasidx	Homo sapiens
myo10	mgc131988|myo10|flj21066|flj22268|flj43256|kiaa0799|flj10639	Homo sapiens
mbtps1	pcsk8|s1p|ski-1|mgc138712|mgc138711|mbtps1|kiaa0091	Homo sapiens
rock1	p160rock|mgc131603|pro0435|mgc43611|rock1	Homo sapiens
lpar1	gpcr26|rec.1.3|mrec1.3|edg-2|lpar1|lpa1|edg2|vzg1|vzg-1	Homo sapiens
dnajb7	dnajb7|hsc3|dj5|mgc138340|hsc-3	Homo sapiens
egfr	erbb1|her1|mena|egfr|pig61|erbb	Homo sapiens
tsg101	tsg10|tsg101|vps23	Homo sapiens
vps4a	flj22197|vps4|vps4a|vps4-1	Homo sapiens

cdk5	pssalre|cdk5	Homo sapiens
pdgfrb	jtk12|pdgfrb|pdgfr1|pdgf-r-beta|cd140b|pdgfr	Homo sapiens
dio3	d3|dioiii|d-3|5diii|txdi3|dio3	Homo sapiens
pdk1	pdk1	Homo sapiens
prkg1	dkfzp686k042|pgk|pkg|prkgr1b|cgki|flj36117|cgki-beta|prkg1b|cgki-alpha|prkg1|mgc71944	Homo sapiens
vasp	vasp	Homo sapiens
rln2	h2|ba12d24.1.1|ba12d24.1.2|rlxh2|rln2	Homo sapiens
timp4	timp4	Homo sapiens
twist1	twist1|bpes3|bpes2|bhlha38|scs|twist	Homo sapiens
itk	mgc126257|mgc126258|emt|itk|lyk	Homo sapiens
arhgef2	dkfzp547p1516|gef-h1|gef|lfp40|arhgef2|dkfzp547l106|gefh1|kiaa0651	Homo sapiens
afap1l2	xb130|kiaa1914|afap1l2	Homo sapiens
atp8a2	ib|atp8a2|atpib|dkfzp434b1913|atp|ml-1	Homo sapiens
mirn206	mir206|mirna206|mirn206|mir-206	Homo sapiens
map2k5	hst17454|map2k5|mapkk5|mek5|prkmk5	Homo sapiens
axpc1	pcarp|pca|axpc1	Homo sapiens
mapk7	mapk7|bmk1|erk5|prkm7	Homo sapiens
rgnef	kiaa1998|flj21817|rgnef|dkfzp686p12164	Homo sapiens
tp53	trp53|flj92943|p53|tp53|lfs1	Homo sapiens
etv5	erm|etv5	Homo sapiens
hdac9	hdac9|hdac|hdrp|dkfzp779k1053|hdac9fl|hd7|hdac7b|hdac9b|mitr|kiaa0744	Homo sapiens
prdx2	nkefb|prx2|tsa|tdpx1|mgc4104|prdx2|prxii	Homo sapiens
tjp1	dkfzp686m05161|zo-1|mgc133289|tjp1	Homo sapiens
creb1	mgc9284|creb1|creb	Homo sapiens
s100p	mig9|s100p	Homo sapiens
ptger4	mgc126583|ptger4|ep4|ep4r	Homo sapiens
limk1	limk|limk1	Homo sapiens
frap1	frap|raft1|rapt1|frap1|frap2|mtor|flj44809	Homo sapiens
grb2	grb2|egfrbp-grb2|ash|grb3-3|mst084|mstp084	Homo sapiens
nme1	gaad|ndpka|awd|ndpk-a|nm23-h1|nm23|nme1	Homo sapiens

sgsm3	dkfzp761d051|sgsm3|rusc3|map|rutbc3	Homo sapiens
decr1	decr1|decr|nadph	Homo sapiens
noxo1	snx28|sh3pxd5|p41noxb|p41noxc|p41noxa|mgc20258|noxo1|p41nox	Homo sapiens
pvrl1	ofc7|pvrr|higr|hvec|cd111|ed4|pvrr1|pvrl1|mgc16207|prr|sk-12|nectin-1|mgc142031|clped1	Homo sapiens
ros1	c-ros-1|ros1|ros|mcf3	Homo sapiens
itpr1	ip3r|itpr1|sca16|sca15|ip3r1|insp3r1	Homo sapiens
mrvi1	irag|mrvi1|jaw1l	Homo sapiens
kif5b	kif5b|kns|kinh|kns1|ukhc	Homo sapiens
kif3a	kif3a	Homo sapiens
kif3b	hh0048|kif3b|kiaa0359	Homo sapiens
nedd9	hef1|cas-l|cass2|nedd9|cas2|casl|dj49g10.2|dj761i2.1	Homo sapiens
enpp2	lysopld|autotaxin|flj26803|pdnp2|atx-x|atx|pd-ialpha|enpp2|npp2	Homo sapiens
rapgef3	bcm910|mgc21410|camp-gefi|rapgef3|hsu79275|epac|epac1	Homo sapiens
aprt	mgc125856|mgc125857|dkfzp686d13177|amp|aprt|mgc129961	Homo sapiens
hspg2	plc|sja|sjs|sjs1|hspg2|prcan	Homo sapiens
ccr7	cmkbr7|cd197|blr2|ccr7|cdw197|ebi1	Homo sapiens
mapk14	rk|sapk2a|csbp1|csbp2|cspb1|p38|p38alpha|mapk14|prkm14|prkm15|mxi2|exip	Homo sapiens
golga2	mgc20672|golga2|gm130	Homo sapiens
grasp	grasp|tamalin	Homo sapiens
cav1	vip21|cav-1|cav1|mstp085	Homo sapiens
abi1	ssh3bp1|abi-1|ssh3bp|abi1|e3b1|nap1bp	Homo sapiens
id1	id|id1|bhlhb24	Homo sapiens
nox3	gp91-3|nox3	Homo sapiens
cybb	gp91-1|cgd|gp91phox|p91-phox|cybb|gp91-phox|nox2	Homo sapiens
nox4	kox-1|kox|renox|nox4	Homo sapiens
actn4	fsgs|dkfzp686k23158|actinin-4|actn4|fsgs1	Homo sapiens
ifi27	isg12a|isg12|p27|fam14d|ifi27	Homo sapiens
cdkn1b	p27kip1|cdkn4|kip1|men4|men1b|cdkn1b	Homo sapiens
nck1	nck|nckalpha|nck1|mgc12668	Homo sapiens
nck2	grb4|nckbeta|nck2	Homo sapiens

ror2	bdb|ror2|ntrkr2|bdb1|mgc163394	Homo sapiens
ncf1	sh3pxd1a|ncf1a|p47phox|noxo2|ncf1	Homo sapiens
rbl2	flj26459|rbl2|rb2|p130	Homo sapiens
wnt1	wnt-1|int1|wnt1	Homo sapiens
il6st	gp130-raps|il6r-beta|cd130|il6st|gp130|cdw130	Homo sapiens
jun	c-jun|ap1|jun	Homo sapiens
stat3	hies|mgc16063|flj20882|aprf|stat3	Homo sapiens
3.6.1.8	adenosine triphosphate pyrophosphatase|atpase|atp diphosphohydrolase [ambiguous]|atp pyrophosphatase|3.6.1.8|atp diphosphatase	Homo sapiens
erbb3	p45-serbb3|lccs2|erbb3-s|erbb3|her3|mda-bf-1|mgc88033|erbb-3|p180-erbb3|c-erbb-3|p85-serbb3|c-erbb3	Homo sapiens
erbb4	erbb4|her4|p180erbb4|mgc138404	Homo sapiens
nrg1	ggf2|hrg1|hrga|ggf|hgl|ndf|aria|smdf|nrg-1|nrg1	Homo sapiens
mapk9	jnk2b|jnk2a|jnk-55|sapk|mapk9|jnk2|p54asapk|jnk2alpha|jnk2beta|p54a|prkm9	Homo sapiens
mapk8	jnk21b1/2|jnk1a2|jnk|mapk8|jnk1|sapk1|prkm8	Homo sapiens
wdtc1	wdtc1|rp11-4k3__a.1|kiaa1037|adp	Homo sapiens
dynamin-2	dynamin-2|dynamin2	Homo sapiens
fnbp1	mgc126804|fnbp1|fbp17	Homo sapiens
col9a1	col9a1|flj40263|med|edm6|dj149l1.1.2	Homo sapiens
gli2	gli2|hpe9|thp2|thp1|thp-1	Homo sapiens
scn8a	ceriii|pn4|nav1.6|scn8a|nach6	Homo sapiens
csf1	m-csf|csf1|mcsf|mgc31930	Homo sapiens
rhou	hg28k|cdc42l1|rhou|arhu|wrch1|fj646b12.2|dj646b12.2|flj10616	Homo sapiens
mif	glif|mif|mmif	Homo sapiens
slurp1	mdm|anup|ly6ls|slurp1	Homo sapiens
prkd1	pkcm|pkc-mu|pkd|prkcm|prkd1	Homo sapiens
egf	egf|homg4|urg	Homo sapiens
lpa	lp|lpa|ak38|apoa	Homo sapiens
3.1.4.3	3.1.4.3|clostridium oedematiens beta- and gamma-toxins|clostridium welchii alpha-toxin|lipophosphodiesterase i|lipophosphodiesterase c|lecithinase c|phospholipase c|heat-labile hemolysin|phosphatidase c|alpha-toxin	Homo sapiens
fzd4	fz-4|fzd4|fze4|gpcr|mgc34390|cd344|fzd4s	Homo sapiens

tktl1	tktl1|tkr|tkt2	Homo sapiens
npy6r	pp2|y2b|npy6r|npy1rl	Homo sapiens
git1	git1	Homo sapiens
vegfa	vpf|vegfa|vegf|mgc70609|vegf-a	Homo sapiens
bcar1	crkas|cass1|p130cas|cas1|bcar1	Homo sapiens
cse1l	xpo2|cse1l|cas|mgc117283|cse1|mgc130037|mgc130036	Homo sapiens
hsp90b2p	hspcp2|hsgrp94b|hsp90b2p|hsp|tra1p1|grp94b|grp94p1	Homo sapiens
hspa4	ry|hs24/p52|mgc131852|hsp70ry|apg-2|hsp70|hspa4	Homo sapiens
hsp90b1	gp96|grp94|hsp90b1|tra1|ecgp	Homo sapiens
iqgap1	iqgap1|sar1|humorfa01|kiaa0051|p195	Homo sapiens
arf1	arf1	Homo sapiens
arf5	arf5	Homo sapiens
rhoq	tc10a|rhoq|rasl7a|arhq|tc10	Homo sapiens
rhoj	tc10b|mgc34777|flj14445|rhoj|rasl7b|tcl|arhj	Homo sapiens
ptpro	glepp1|ptpu2|ptpro|ptp-u2	Homo sapiens
reg1a	psps1|psps|icrf|ptp|reg1a|mgc12447	Homo sapiens
ptpn6	hptp1c|ptpn6|hcph|shp-1|ptp-1c|shp1|shp-1l|sh-ptp1	Homo sapiens
spp1	eta-1|opn|spp1|bspi|mgc110940	Homo sapiens
pcsk1	pcsk1|pc1|pc3|spc3|nec1	Homo sapiens
mmp19	rasi-1|mmp-19|mmp18|mmp19	Homo sapiens
mmp1	clg|mmp1|mmp-1	Homo sapiens
mapk1	p42mapk|p41|mapk2|mapk1|erk2|ert1|prkm2|prkm1|p41mapk	Homo sapiens
mrc1	cd206|clec13d|mmr|mrc1	Homo sapiens
ly75	dec-205|cd205|clec13b|gp200-mr6|ly75	Homo sapiens
cdkn2a	p16ink4a|ink4|arf|mlm|p14|p16|p19|p16ink4|cdk4i|cdkn2|tp16|p14arf|cmm2|ink4a|mts1|cdkn2a	Homo sapiens
rasa1	rasa1|pkws|cm-avm|rasa|dkfzp434n071|gap|p120rasgap|rasgap|cmavm|p120gap	Homo sapiens
adrb2	adrb2r|b2ar|bar|adrbr|adrb2|beta2ar	Homo sapiens
clathrin	clathrin-hc|clathrin	Homo sapiens
cd2ap	cd2ap|cms|dkfzp586h0519	Homo sapiens
stat5a	stat5a|stat5	Homo sapiens

ifng	ifngamma|ifng|ifg|ifi|ifn-gamma|ifn gamma	Homo sapiens
bcr	bcr-abl1|d22s11|all|bcr|cml|phl|flj16453|bcr1|d22s662	Homo sapiens
actr3	actr3|arp3	Homo sapiens
bcatenin	bcatenin|beta catenin|beta-catenin	Homo sapiens
hnf4a	hnf4|hnf4a|tcf14|tcf|hnf4a9|hnf4a8|hnf4a7|nr2a1|mody|nr2a21|flj39654|mody1	Homo sapiens
cad	cad	Homo sapiens
arhgdia	arhgdia|mgc117248|gdia1|rhogdi|rhogdi-1	Homo sapiens
fermt2	unc112b|kind2|mig-2|flj34213|unc112|plekhc1|mig2|flj44462|fermt2|dkfzp686g11125	Homo sapiens
ilk	dkfzp686f1765|ilk|p59	Homo sapiens
sos1	ggf1|sos-1|gingf|ns4|sos1	Homo sapiens
eps8	eps8	Homo sapiens
irf6	ofc6|irf6|lps|pit|pps|vws	Homo sapiens
mrxs5	mrxs5|pgs	Homo sapiens
tnfa	tnfalpha|tumor-necrosis-factor-alpha|tnfa|tnf-alpha|tnf alpha	Homo sapiens
nf-kappab	nf-kappab|nfkappab|nf-kappa b|nfkb|nfkappa b	Homo sapiens
cxcl12	scyb12|pbsf|tlsf-a|tlsf-b|sdf-1b|cxcl12|tpar1|sdf1b|sdf1a	Homo sapiens
cxcr4	hsy3rr|hm89|npyy3r|cd184|d2s201e|lestr|lcr1|npy3r|npyrl|cxcr4|whim|fb22	Homo sapiens
mapk3	mgc20180|p44mapk|hs44kdap|mapk3|p44erk1|humker1a|erk1|prkm3	Homo sapiens
fos	c-fos|fos|ap-1	Homo sapiens
timp1	flj90373|timp1|epa|hci|timp-1|timp|clgi	Homo sapiens
tgfb	tgf-beta|tgfbeta|tgfb	Homo sapiens
vcam1	vcam-1|dkfzp779g2333|vcam1|cd106|mgc99561|incam-100	Homo sapiens
ptprd	ptpd|ptprd|hptp|hptpd|r-ptp-delta|hptp-delta|mgc119753|mgc119752|mgc119751|mgc119750	Homo sapiens
grlf1	grf-1|grlf1|kiaa1722|p190a|p190rhogap|mgc10745|p190-a	Homo sapiens
dym	flj90130|dmc|dym|smc|flj20071	Homo sapiens
uts2r	gpr14|utr|utr2|uts2r	Homo sapiens
cd44	in|hcell|ecmr-iii|pgp1|cspg8|lhr|cdw44|cd44|mgc10468|mc56|mdu3|mdu2|mic4|mutch-i	Homo sapiens
brap	imp|rnf52|brap|brap2	Homo sapiens
sdf1	sdf-1alpha|sdf-1a|sdf1|sdf1-alpha|sdf-1	Homo sapiens
mkks	hmcs|mks|bbs6|mkks	Homo sapiens

gp6	gpvi|gp6|mgc138168	Homo sapiens
tat	tat	Homo sapiens
3.6.4.1	actomyosin|myosin atpase|3.6.4.1	Homo sapiens
trpm7	trpm7|trp-plik|flj25718|flj20117|chak|ltrpc7|chak1	Homo sapiens
myh14	kiaa2034|myh14|mhc16|dfna4|myosin|flj13881|fp17425|dkfzp667a1311|nmhc-ii-c|flj43092	Homo sapiens
hsp90aa1	flj31884|hsp90aa1|hsp89a|hsp90a|hsp90n|hspn|hspcal4|hspcal1|lap2|hsp89|hsp86|hsp90|hspca|hspc1	Homo sapiens
bhlhb5	beta3|tnrc20|bhlhe22|bhlhb5|cagl85	Homo sapiens
actr2	actr2|arp2	Homo sapiens
afap1	afap|flj56849|afap1|afap-110	Homo sapiens
pkca	pkca|pkc alpha|pkc-alpha|pkcalpha	Homo sapiens
ephb2	pcbc|mgc87492|hek5|drt|erk|tyro5|epht3|ephb2	Homo sapiens
mtg1	rp11-108k14.2|gtp|gtpbp7|mtg1	Homo sapiens
pik3ca	pi3k|mgc142161|mgc142163|pi3-k|pik3ca|p110-alpha	Homo sapiens
akt1	pkb-alpha|rac-alpha|akt|akt1|rac|mgc99656|prkba	Homo sapiens
icam1	icam-1|bb2|icam1|p3.58|cd54	Homo sapiens
tnfsf13	tall2|unq383/pro715|cd256|trdl-1|april|tnfsf13|ligand	Homo sapiens
dynamin	dynamin|dynamin-1|dynamin1	Homo sapiens
plau	plau|atf|upa|urk|u-pa|urokinase	Homo sapiens
plaur	plaur|cd87|upar|urkr	Homo sapiens
slco6a1	oatp6a1|gst|oatpy|slco6a1|mgc26949	Homo sapiens
crkl	crkl	Homo sapiens
tnf	tnfsf2|dif|tnf	Homo sapiens
syk	syk|flj37489|flj25043|dkfzp313n1010	Homo sapiens
timp2	timp2|timp-2|csc-21k	Homo sapiens
lyn	flj26625|lyn|jtk8	Homo sapiens
arhgap1	cdc42gap|arhgap1|rhogap1|rhogap|p50rhogap	Homo sapiens
cntnap1	cntnap1|caspr|cntnap|p190|nrxn4	Homo sapiens
mmp2	tbe-1|clg4a|clg4|mmp-ii|mmp2|mona|mmp-2	Homo sapiens
il2ra	il-2ralpha|tcgfr|il2r|il-2r|il2ra|cd25	Homo sapiens
lox	lox|mgc105112	Homo sapiens

itgb2	lcamb|itgb2|cd18|mf17|mfi7	Homo sapiens
actin	actin	Homo sapiens
pkc	pkc|dyt10	Homo sapiens
rhoc	h9|mgc1448|rhoc|h-9|mgc61427|arhc|arh9|rhoh9	Homo sapiens
il2	lymphokine|il-2|il2|tcgf	Homo sapiens
il1b	il-1|il1b|il1f2|il1beta|il1-beta|il1 beta|il-1 beta|il-1beta	Homo sapiens
cyld	flj31664|hspc057|eac|mft|sbs|tem|flj20180|cdmt|uspl2|cyld|mft1|kiaa0849|flj78684|cyldi|cyld1	Homo sapiens
mmrn1	ecm|mmrn|mmrn1|emilin4|gpia*	Homo sapiens

List of invadopodia-related publications identified using ALS

Source	Target	PubMed entry
tyrp1	mmrn1	http://www.ncbi.nlm.nih.gov/entrez/query.fcgi?cmd=Retrieve&db=pubmed&dopt=Abstract&list_uids=27591737|6|J Cell Physiol|||Sep|2016|JOURNAL ARTICLE
tyrp1	actin	http://www.ncbi.nlm.nih.gov/entrez/query.fcgi?cmd=Retrieve&db=pubmed&dopt=Abstract&list_uids=27591737|6|J Cell Physiol|||Sep|2016|JOURNAL ARTICLE
mmrn1	actin	http://www.ncbi.nlm.nih.gov/entrez/query.fcgi?cmd=Retrieve&db=pubmed&dopt=Abstract&list_uids=27591737|6|J Cell Physiol|||Sep|2016|JOURNAL ARTICLE|http://www.ncbi.nlm.nih.gov/entrez/query.fcgi?cmd=Retrieve&db=pubmed&dopt=Abstract&list_uids=25081617|6|Prog Mol Biol Transl Sci|126|||2014|Journal Article,Research Support, Non-U.S. Gov't,Review|http://www.ncbi.nlm.nih.gov/entrez/query.fcgi?cmd=Retrieve&db=pubmed&dopt=Abstract&list_uids=25918420|6|Proc Natl Acad Sci U S A|112|19|May|2015|Journal Article,Research Support, N.I.H., Intramural|http://www.ncbi.nlm.nih.gov/entrez/query.fcgi?cmd=Retrieve&db=pubmed&dopt=Abstract&list_uids=25549589|6|Matrix Biol|42||Mar|2015|Journal Article,Research Support, N.I.H., Extramural
lims1	nck2	http://www.ncbi.nlm.nih.gov/entrez/query.fcgi?cmd=Retrieve&db=pubmed&dopt=Abstract&list_uids=27590440|6|Gene|||Aug|2016|REVIEW,JOURNAL ARTICLE
lims1	ilk	http://www.ncbi.nlm.nih.gov/entrez/query.fcgi?cmd=Retrieve&db=pubmed&dopt=Abstract&list_uids=27590440|6|Gene|||Aug|2016|REVIEW,JOURNAL ARTICLE|http://www.ncbi.nlm.nih.gov/entrez/query.fcgi?cmd=Retrieve&db=pubmed&dopt=Abstract&list_uids=23383235|6|PLoS One|8|1||2013|Journal Article,Research Support, N.I.H., Extramural|http://www.ncbi.nlm.nih.gov/entrez/query.fcgi?cmd=Retrieve&db=pubmed&dopt=Abstract&list_uids=25482629|6|Cell Adh Migr|9|3||2015|Journal Article,Research Support, U.S. Gov't, Non-P.H.S.
lims1	ipp	http://www.ncbi.nlm.nih.gov/entrez/query.fcgi?cmd=Retrieve&db=pubmed&dopt=Abstract&list_uids=27590440|6|Gene|||Aug|2016|REVIEW,JOURNAL ARTICLE|http://www.ncbi.nlm.nih.gov/entrez/query.fcgi?cmd=Retrieve&db=pubmed&dopt=Abstract&list_uids=23383235|6|PLoS One|8|1||2013|Journal Article,Research Support, N.I.H., Extramural|http://www.ncbi.nlm.nih.gov/entrez/query.fcgi?cmd=Retrieve&db=pubmed&dopt=Abstract&list_uids=25482629|6|Cell Adh Migr|9|3||2015|Journal Article,Research Support, U.S. Gov't, Non-P.H.S.
nck2	ilk	http://www.ncbi.nlm.nih.gov/entrez/query.fcgi?cmd=Retrieve&db=pubmed&dopt=Abstract&list_uids=27590440|6|Gene|||Aug|2016|REVIEW,JOURNAL ARTICLE
nck2	ipp	http://www.ncbi.nlm.nih.gov/entrez/query.fcgi?cmd=Retrieve&db=pubmed&dopt=Abstract&list_uids=27590440|6|Gene|||Aug|2016|REVIEW,JOURNAL ARTICLE
ilk	ipp	http://www.ncbi.nlm.nih.gov/entrez/query.fcgi?cmd=Retrieve&db=pubmed&dopt=Abstract&list_uids=27590440|6|Gene|||Aug|2016|REVIEW,JOURNAL ARTICLE|http://www.ncbi.nlm.nih.gov/entrez/query.fcgi?cmd=Retrieve&db=pubmed&dopt=Abstract&list_uids=23383235|

		6|PLoS One|8|1||2013|Journal Article,Research Support, N.I.H., Extramural|http://www.ncbi.nlm.nih.gov/entrez/query.fcgi?cmd=Retrieve&db=pubmed&dopt=Abstract&list_uids=25482629|6|Cell Adh Migr|9|3||2015|Journal Article,Research Support, U.S. Gov't, Non-P.H.S.
ipp	mmrn1	http://www.ncbi.nlm.nih.gov/entrez/query.fcgi?cmd=Retrieve&db=pubmed&dopt=Abstract&list_uids=27590440|6|Gene|||Aug|2016|REVIEW,JOURNAL ARTICLE
prl	nek3	http://www.ncbi.nlm.nih.gov/entrez/query.fcgi?cmd=Retrieve&db=pubmed&dopt=Abstract&list_uids=27489110|6|J Biol Chem|||Aug|2016|JOURNAL ARTICLE
prl	actin	http://www.ncbi.nlm.nih.gov/entrez/query.fcgi?cmd=Retrieve&db=pubmed&dopt=Abstract&list_uids=27489110|6|J Biol Chem|||Aug|2016|JOURNAL ARTICLE
nek3	actin	http://www.ncbi.nlm.nih.gov/entrez/query.fcgi?cmd=Retrieve&db=pubmed&dopt=Abstract&list_uids=27489110|6|J Biol Chem|||Aug|2016|JOURNAL ARTICLE
nek3	mapk3	http://www.ncbi.nlm.nih.gov/entrez/query.fcgi?cmd=Retrieve&db=pubmed&dopt=Abstract&list_uids=27489110|6|J Biol Chem|||Aug|2016|JOURNAL ARTICLE
plp2	ca4	http://www.ncbi.nlm.nih.gov/entrez/query.fcgi?cmd=Retrieve&db=pubmed&dopt=Abstract&list_uids=27292568|6|Sci Rep|6|||2016|Journal Article
rhoa	rac1	http://www.ncbi.nlm.nih.gov/entrez/query.fcgi?cmd=Retrieve&db=pubmed&dopt=Abstract&list_uids=27187066|6|PLoS One|11|5||2016|Journal Article|http://www.ncbi.nlm.nih.gov/entrez/query.fcgi?cmd=Retrieve&db=pubmed&dopt=Abstract&list_uids=23615439|6|Mol Biol Cell|24|12|Jun|2013|Journal Article,Research Support, N.I.H., Extramural|http://www.ncbi.nlm.nih.gov/entrez/query.fcgi?cmd=Retrieve&db=pubmed&dopt=Abstract&list_uids=22634185|6|Cell Signal|24|9|Sep|2012|Journal Article,Research Support, Non-U.S. Gov't|http://www.ncbi.nlm.nih.gov/entrez/query.fcgi?cmd=Retrieve&db=pubmed&dopt=Abstract&list_uids=23344455|6|Toxins (Basel)|5|1|Jan|2013|Comparative Study,Journal Article,Research Support, Non-U.S. Gov't|http://www.ncbi.nlm.nih.gov/entrez/query.fcgi?cmd=Retrieve&db=pubmed&dopt=Abstract&list_uids=24903274|6|FASEB J|28|9|Sep|2014|Journal Article,Research Support, Non-U.S. Gov't|http://www.ncbi.nlm.nih.gov/entrez/query.fcgi?cmd=Retrieve&db=pubmed&dopt=Abstract&list_uids=24675530|6|Carcinogenesis|35|9|Sep|2014|Journal Article,Research Support, Non-U.S. Gov't|http://www.ncbi.nlm.nih.gov/entrez/query.fcgi?cmd=Retrieve&db=pubmed&dopt=Abstract&list_uids=22879066|6|Adv Exp Med Biol|986|||2013|Journal Article,Review|http://www.ncbi.nlm.nih.gov/entrez/query.fcgi?cmd=Retrieve&db=pubmed&dopt=Abstract&list_uids=22721990|6|Cardiovasc Res|95|3|Aug|2012|Journal Article,Research Support, Non-U.S. Gov't
rhoa	mbtps1	http://www.ncbi.nlm.nih.gov/entrez/query.fcgi?cmd=Retrieve&db=pubmed&dopt=Abstract&list_uids=27187066|6|PLoS One|11|5||2016|Journal Article
rac1	mbtps1	http://www.ncbi.nlm.nih.gov/entrez/query.fcgi?cmd=Retrieve&db=pubmed&dopt=Abstract&list_uids=27187066|6|PLoS One|11|5||2016|Journal Article
rhoa	gpsn2	http://www.ncbi.nlm.nih.gov/entrez/query.fcgi?cmd=Retrieve&db=pubmed&dopt=Abstract&list_uids=27187066|6|PLoS

		One|11|5||2016|Journal Article
mbtps1	gpsn2	http://www.ncbi.nlm.nih.gov/entrez/query.fcgi?cmd=Retrieve&db=pubmed&dopt=Abstract&list_uids=27187066|6|PLoS One|11|5||2016|Journal Article
myh14	rhoa	http://www.ncbi.nlm.nih.gov/entrez/query.fcgi?cmd=Retrieve&db=pubmed&dopt=Abstract&list_uids=27115795|6|Am J Respir Cell Mol Biol|||Apr|2016|JOURNAL ARTICLE|http://www.ncbi.nlm.nih.gov/entrez/query.fcgi?cmd=Retrieve&db=pubmed&dopt=Abstract&list_uids=27187066|6|PLoS One|11|5||2016|Journal Article|http://www.ncbi.nlm.nih.gov/entrez/query.fcgi?cmd=Retrieve&db=pubmed&dopt=Abstract&list_uids=23762409|6|PLoS One|8|6||2013|Journal Article,Research Support, N.I.H., Extramural,Research Support, Non-U.S. Gov't|http://www.ncbi.nlm.nih.gov/entrez/query.fcgi?cmd=Retrieve&db=pubmed&dopt=Abstract&list_uids=23444376|6|J Cell Sci|126|Pt 7|Apr|2013|Journal Article,Research Support, Non-U.S. Gov't,Research Support, U.S. Gov't, Non-P.H.S.|http://www.ncbi.nlm.nih.gov/entrez/query.fcgi?cmd=Retrieve&db=pubmed&dopt=Abstract&list_uids=26906158|6|Invest Ophthalmol Vis Sci|57|2|Feb|2016|Journal Article,Research Support, Non-U.S. Gov't|http://www.ncbi.nlm.nih.gov/entrez/query.fcgi?cmd=Retrieve&db=pubmed&dopt=Abstract&list_uids=25471072|6|EMBO J|34|4|Feb|2015|Journal Article,Research Support, Non-U.S. Gov't|http://www.ncbi.nlm.nih.gov/entrez/query.fcgi?cmd=Retrieve&db=pubmed&dopt=Abstract&list_uids=26483365|6|Oncotarget|6|31|Oct|2015|Journal Article,Research Support, N.I.H., Extramural
mbtps1	actin	http://www.ncbi.nlm.nih.gov/entrez/query.fcgi?cmd=Retrieve&db=pubmed&dopt=Abstract&list_uids=27187066|6|PLoS One|11|5||2016|Journal Article
rhoa	actin	http://www.ncbi.nlm.nih.gov/entrez/query.fcgi?cmd=Retrieve&db=pubmed&dopt=Abstract&list_uids=27187066|6|PLoS One|11|5||2016|Journal Article|http://www.ncbi.nlm.nih.gov/entrez/query.fcgi?cmd=Retrieve&db=pubmed&dopt=Abstract&list_uids=23028669|6|PLoS One|7|9||2012|Journal Article,Research Support, N.I.H., Extramural|http://www.ncbi.nlm.nih.gov/entrez/query.fcgi?cmd=Retrieve&db=pubmed&dopt=Abstract&list_uids=26427649|6|Biochim Biophys Acta|1853|12|Dec|2015|Journal Article,Research Support, N.I.H., Extramural|http://www.ncbi.nlm.nih.gov/entrez/query.fcgi?cmd=Retrieve&db=pubmed&dopt=Abstract&list_uids=24126053|6|Mol Cell Biol|33|24|Dec|2013|Journal Article,Research Support, N.I.H., Extramural|http://www.ncbi.nlm.nih.gov/entrez/query.fcgi?cmd=Retrieve&db=pubmed&dopt=Abstract&list_uids=23716571|6|Hum Mol Genet|22|19|Oct|2013|Journal Article,Research Support, Non-U.S. Gov't|http://www.ncbi.nlm.nih.gov/entrez/query.fcgi?cmd=Retrieve&db=pubmed&dopt=Abstract&list_uids=24675530|6|Carcinogenesis|35|9|Sep|2014|Journal Article,Research Support, Non-U.S. Gov't|http://www.ncbi.nlm.nih.gov/entrez/query.fcgi?cmd=Retrieve&db=pubmed&dopt=Abstract&list_uids=23707396|6|Exp Cell Res|319|13|Aug|2013|Journal Article|http://www.ncbi.nlm.nih.gov/entrez/query.fcgi?cmd=Retrieve&db=pubmed&dopt=Abstract&list_uids=22879066|6|Adv Exp Med Biol|986|||2013|Journal Article,Review|http://www.ncbi.nlm.nih.gov/entrez/query.fcgi?cmd=Retrieve&db=pubmed&dopt=Abstract&list_uids=23836

		527|6|Stem Cells|31|11|Nov|2013|Journal Article,Research Support, N.I.H., Extramural|http://www.ncbi.nlm.nih.gov/entrez/query.fcgi?cmd=Retrieve&db=pubmed&dopt=Abstract&list_uids=23176180|6|BMC Cancer|12|||2012|Journal Article,Research Support, Non-U.S. Gov't
rab5a	rac1	http://www.ncbi.nlm.nih.gov/entrez/query.fcgi?cmd=Retrieve&db=pubmed&dopt=Abstract&list_uids=27121131|6|Oncotarget|7|20|May|2016|Journal Article
rab5a	ptk2	http://www.ncbi.nlm.nih.gov/entrez/query.fcgi?cmd=Retrieve&db=pubmed&dopt=Abstract&list_uids=23813952|6|J Cell Sci|126|Pt 17|Sep|2013|Journal Article,Research Support, Non-U.S. Gov't|http://www.ncbi.nlm.nih.gov/entrez/query.fcgi?cmd=Retrieve&db=pubmed&dopt=Abstract&list_uids=27121131|6|Oncotarget|7|20|May|2016|Journal Article
ptk2	rac1	http://www.ncbi.nlm.nih.gov/entrez/query.fcgi?cmd=Retrieve&db=pubmed&dopt=Abstract&list_uids=25820249|6|J Cell Physiol|230|11|Nov|2015|Journal Article,Research Support, Non-U.S. Gov't|http://www.ncbi.nlm.nih.gov/entrez/query.fcgi?cmd=Retrieve&db=pubmed&dopt=Abstract&list_uids=24858039|6|Oncogene|34|15|Apr|2015|Journal Article,Research Support, N.I.H., Extramural,Research Support, Non-U.S. Gov't|http://www.ncbi.nlm.nih.gov/entrez/query.fcgi?cmd=Retrieve&db=pubmed&dopt=Abstract&list_uids=24480309|6|Arch Biochem Biophys|545||Mar|2014|Journal Article,Research Support, Non-U.S. Gov't|http://www.ncbi.nlm.nih.gov/entrez/query.fcgi?cmd=Retrieve&db=pubmed&dopt=Abstract&list_uids=26299802|6|Eur J Cell Biol|94|11|Nov|2015|Journal Article,Research Support, Non-U.S. Gov't|http://www.ncbi.nlm.nih.gov/entrez/query.fcgi?cmd=Retrieve&db=pubmed&dopt=Abstract&list_uids=27121131|6|Oncotarget|7|20|May|2016|Journal Article|http://www.ncbi.nlm.nih.gov/entrez/query.fcgi?cmd=Retrieve&db=pubmed&dopt=Abstract&list_uids=22721990|6|Cardiovasc Res|95|3|Aug|2012|Journal Article,Research Support, Non-U.S. Gov't
f2	il6	http://www.ncbi.nlm.nih.gov/entrez/query.fcgi?cmd=Retrieve&db=pubmed&dopt=Abstract&list_uids=27115795|6|Am J Respir Cell Mol Biol|||Apr|2016|JOURNAL ARTICLE
f2	ptk2	http://www.ncbi.nlm.nih.gov/entrez/query.fcgi?cmd=Retrieve&db=pubmed&dopt=Abstract&list_uids=27115795|6|Am J Respir Cell Mol Biol|||Apr|2016|JOURNAL ARTICLE
f2	bcar1	http://www.ncbi.nlm.nih.gov/entrez/query.fcgi?cmd=Retrieve&db=pubmed&dopt=Abstract&list_uids=27115795|6|Am J Respir Cell Mol Biol|||Apr|2016|JOURNAL ARTICLE
il6	ptk2	http://www.ncbi.nlm.nih.gov/entrez/query.fcgi?cmd=Retrieve&db=pubmed&dopt=Abstract&list_uids=27115795|6|Am J Respir Cell Mol Biol|||Apr|2016|JOURNAL ARTICLE|http://www.ncbi.nlm.nih.gov/entrez/query.fcgi?cmd=Retrieve&db=pubmed&dopt=Abstract&list_uids=24912675|6|Mol Cell Biol|34|16|Aug|2014|Journal Article,Research Support, Non-U.S. Gov't
il6	bcar1	http://www.ncbi.nlm.nih.gov/entrez/query.fcgi?cmd=Retrieve&db=pubmed&dopt=Abstract&list_uids=27115795|6|Am J Respir Cell Mol Biol|||Apr|2016|JOURNAL ARTICLE
ptk2	bcar1	http://www.ncbi.nlm.nih.gov/entrez/query.fcgi?cmd=Retrieve&db=pubmed&dopt=Abstract&list_uids=27115795|6|Am J Respir Cell Mol Biol|||Apr|2016|JOURNAL ARTICLE|http://www.ncbi.nlm.nih.gov/entrez/query.fcgi?cmd=Retrieve&db=pubmed&dopt=Abstract&list_uids=24642576|

		6|PLoS One|9|3||2014|Journal Article,Research Support, Non-U.S. Gov't|http://www.ncbi.nlm.nih.gov/entrez/query.fcgi?cmd=Retrieve&db=pubmed&dopt=Abstract&list_uids=24480309|6|Arch Biochem Biophys|545||Mar|2014|Journal Article,Research Support, Non-U.S. Gov't
il6	rhoa	http://www.ncbi.nlm.nih.gov/entrez/query.fcgi?cmd=Retrieve&db=pubmed&dopt=Abstract&list_uids=27115795|6|Am J Respir Cell Mol Biol|||Apr|2016|JOURNAL ARTICLE
il6	myh14	http://www.ncbi.nlm.nih.gov/entrez/query.fcgi?cmd=Retrieve&db=pubmed&dopt=Abstract&list_uids=27115795|6|Am J Respir Cell Mol Biol|||Apr|2016|JOURNAL ARTICLE
il6	soat1	http://www.ncbi.nlm.nih.gov/entrez/query.fcgi?cmd=Retrieve&db=pubmed&dopt=Abstract&list_uids=27115795|6|Am J Respir Cell Mol Biol|||Apr|2016|JOURNAL ARTICLE
rhoa	soat1	http://www.ncbi.nlm.nih.gov/entrez/query.fcgi?cmd=Retrieve&db=pubmed&dopt=Abstract&list_uids=27115795|6|Am J Respir Cell Mol Biol|||Apr|2016|JOURNAL ARTICLE
myh14	soat1	http://www.ncbi.nlm.nih.gov/entrez/query.fcgi?cmd=Retrieve&db=pubmed&dopt=Abstract&list_uids=27115795|6|Am J Respir Cell Mol Biol|||Apr|2016|JOURNAL ARTICLE
dcn	lum	http://www.ncbi.nlm.nih.gov/entrez/query.fcgi?cmd=Retrieve&db=pubmed&dopt=Abstract&list_uids=27111714|6|Cell Adh Migr|||Apr|2016|JOURNAL ARTICLE
dcn	sdc1	http://www.ncbi.nlm.nih.gov/entrez/query.fcgi?cmd=Retrieve&db=pubmed&dopt=Abstract&list_uids=27111714|6|Cell Adh Migr|||Apr|2016|JOURNAL ARTICLE
dcn	mrxs5	http://www.ncbi.nlm.nih.gov/entrez/query.fcgi?cmd=Retrieve&db=pubmed&dopt=Abstract&list_uids=27111714|6|Cell Adh Migr|||Apr|2016|JOURNAL ARTICLE
lum	sdc1	http://www.ncbi.nlm.nih.gov/entrez/query.fcgi?cmd=Retrieve&db=pubmed&dopt=Abstract&list_uids=27111714|6|Cell Adh Migr|||Apr|2016|JOURNAL ARTICLE
lum	mrxs5	http://www.ncbi.nlm.nih.gov/entrez/query.fcgi?cmd=Retrieve&db=pubmed&dopt=Abstract&list_uids=27111714|6|Cell Adh Migr|||Apr|2016|JOURNAL ARTICLE
sdc1	mrxs5	http://www.ncbi.nlm.nih.gov/entrez/query.fcgi?cmd=Retrieve&db=pubmed&dopt=Abstract&list_uids=27111714|6|Cell Adh Migr|||Apr|2016|JOURNAL ARTICLE
cryab	actin	http://www.ncbi.nlm.nih.gov/entrez/query.fcgi?cmd=Retrieve&db=pubmed&dopt=Abstract&list_uids=27107034|6|Reproduction|152|1|Jul|2016|Journal Article
itk	ptk2	http://www.ncbi.nlm.nih.gov/entrez/query.fcgi?cmd=Retrieve&db=pubmed&dopt=Abstract&list_uids=26992741|6|Cancer Lett|375|2|Jun|2016|Journal Article,Research Support, Non-U.S. Gov't|http://www.ncbi.nlm.nih.gov/entrez/query.fcgi?cmd=Retrieve&db=pubmed&dopt=Abstract&list_uids=26318800|6|Acta Biomater|27||Nov|2015|Journal Article,Research Support, Non-U.S. Gov't
ptk2	akt1	http://www.ncbi.nlm.nih.gov/entrez/query.fcgi?cmd=Retrieve&db=pubmed&dopt=Abstract&list_uids=24635319|6|Biochem J|459|3|May|2014|Journal Article,Research Support, Non-U.S. Gov't|http://www.ncbi.nlm.nih.gov/entrez/query.fcgi?cmd=Retrieve&db=pubmed&dopt=Abstract&list_uids=24861878|6|Neuro Oncol|16|9|Sep|2014|Journal Article,Research Support, Non-U.S. Gov't|http://www.ncbi.nlm.nih.gov/entrez/query.fcgi?cmd=Retrieve&db=pubmed&dopt=Abstract&list_uids=24194567|6|Mo

		l Cancer Ther|13|1|Jan|2014|Journal Article,Research Support, Non-U.S. Gov't|http://www.ncbi.nlm.nih.gov/entrez/query.fcgi?cmd=Retrieve&db=pubmed&dopt=Abstract&list_uids=26988603|6|Sci Rep|6|||2016|Journal Article,Research Support, Non-U.S. Gov't|http://www.ncbi.nlm.nih.gov/entrez/query.fcgi?cmd=Retrieve&db=pubmed&dopt=Abstract&list_uids=22932395|6|FASEB J|26|12|Dec|2012|Journal Article,Research Support, Non-U.S. Gov't|http://www.ncbi.nlm.nih.gov/entrez/query.fcgi?cmd=Retrieve&db=pubmed&dopt=Abstract&list_uids=26194913|6|EMBO Mol Med|7|10|Oct|2015|Journal Article,Research Support, Non-U.S. Gov't|http://www.ncbi.nlm.nih.gov/entrez/query.fcgi?cmd=Retrieve&db=pubmed&dopt=Abstract&list_uids=23836527|6|Stem Cells|31|11|Nov|2013|Journal Article,Research Support, N.I.H., Extramural|http://www.ncbi.nlm.nih.gov/entrez/query.fcgi?cmd=Retrieve&db=pubmed&dopt=Abstract&list_uids=22575727|6|Gene|503|2|Jul|2012|Journal Article,Research Support, N.I.H., Extramural,Review
ppp2r4	pdlim5	http://www.ncbi.nlm.nih.gov/entrez/query.fcgi?cmd=Retrieve&db=pubmed&dopt=Abstract&list_uids=26945059|6|J Cell Sci|129|8|Apr|2016|Journal Article,Research Support, Non-U.S. Gov't
ppp2r4	ppp2r3a	http://www.ncbi.nlm.nih.gov/entrez/query.fcgi?cmd=Retrieve&db=pubmed&dopt=Abstract&list_uids=26945059|6|J Cell Sci|129|8|Apr|2016|Journal Article,Research Support, Non-U.S. Gov't
pdlim5	ppp2r3a	http://www.ncbi.nlm.nih.gov/entrez/query.fcgi?cmd=Retrieve&db=pubmed&dopt=Abstract&list_uids=26945059|6|J Cell Sci|129|8|Apr|2016|Journal Article,Research Support, Non-U.S. Gov't
pdlim5	lpp	http://www.ncbi.nlm.nih.gov/entrez/query.fcgi?cmd=Retrieve&db=pubmed&dopt=Abstract&list_uids=26945059|6|J Cell Sci|129|8|Apr|2016|Journal Article,Research Support, Non-U.S. Gov't
lpp	ppp2r4	http://www.ncbi.nlm.nih.gov/entrez/query.fcgi?cmd=Retrieve&db=pubmed&dopt=Abstract&list_uids=26945059|6|J Cell Sci|129|8|Apr|2016|Journal Article,Research Support, Non-U.S. Gov't
lpp	ppp2r3a	http://www.ncbi.nlm.nih.gov/entrez/query.fcgi?cmd=Retrieve&db=pubmed&dopt=Abstract&list_uids=26945059|6|J Cell Sci|129|8|Apr|2016|Journal Article,Research Support, Non-U.S. Gov't
psma5	3.6.4.1	http://www.ncbi.nlm.nih.gov/entrez/query.fcgi?cmd=Retrieve&db=pubmed&dopt=Abstract&list_uids=26906158|6|Invest Ophthalmol Vis Sci|57|2|Feb|2016|Journal Article,Research Support, Non-U.S. Gov't
psma5	rhoa	http://www.ncbi.nlm.nih.gov/entrez/query.fcgi?cmd=Retrieve&db=pubmed&dopt=Abstract&list_uids=26906158|6|Invest Ophthalmol Vis Sci|57|2|Feb|2016|Journal Article,Research Support, Non-U.S. Gov't
3.6.4.1	rhoa	http://www.ncbi.nlm.nih.gov/entrez/query.fcgi?cmd=Retrieve&db=pubmed&dopt=Abstract&list_uids=26906158|6|Invest Ophthalmol Vis Sci|57|2|Feb|2016|Journal Article,Research Support, Non-U.S. Gov't|http://www.ncbi.nlm.nih.gov/entrez/query.fcgi?cmd=Retrieve&db=pubmed&dopt=Abstract&list_uids=25526367|6|PLoS One|9|12||2014|Journal Article,Research Support, Non-U.S. Gov't
psma5	myh14	http://www.ncbi.nlm.nih.gov/entrez/query.fcgi?cmd=Retrieve&db=pubmed&dopt=Abstract&list_uids=26906158|6|Invest Ophthalmol Vis Sci|57|2|Feb|2016|Journal Article,Research Support, Non-U.S. Gov't
psma5	mlc1	http://www.ncbi.nlm.nih.gov/entrez/query.fcgi?cmd=Retrieve&db=pubmed&dopt=Abstract&list_uids=26906158|6|Invest Ophthalmol Vis Sci|57|2|Feb|2016|Journal Article,Research Support, Non-U.S. Gov't
rhoa	mlc1	http://www.ncbi.nlm.nih.gov/entrez/query.fcgi?cmd=Retrieve&db=pubmed&dopt=Abstract&list_uids=26906158|6|Invest

		Ophthalmol Vis Sci|57|2|Feb|2016|Journal Article,Research Support, Non-U.S. Gov't
myh14	mlc1	http://www.ncbi.nlm.nih.gov/entrez/query.fcgi?cmd=Retrieve&db=pubmed&dopt=Abstract&list_uids=22237407|6|Am J Physiol Cell Physiol|302|7|Apr|2012|Journal Article,Research Support, N.I.H., Extramural,Research Support, N.I.H., Intramural|http://www.ncbi.nlm.nih.gov/entrez/query.fcgi?cmd=Retrieve&db=pubmed&dopt=Abstract&list_uids=26906158|6|Invest Ophthalmol Vis Sci|57|2|Feb|2016|Journal Article,Research Support, Non-U.S. Gov't|http://www.ncbi.nlm.nih.gov/entrez/query.fcgi?cmd=Retrieve&db=pubmed&dopt=Abstract&list_uids=24610576|6|J Cell Biochem|115|9|Sep|2014|Journal Article,Research Support, Non-U.S. Gov't
psma5	mmrn1	http://www.ncbi.nlm.nih.gov/entrez/query.fcgi?cmd=Retrieve&db=pubmed&dopt=Abstract&list_uids=26906158|6|Invest Ophthalmol Vis Sci|57|2|Feb|2016|Journal Article,Research Support, Non-U.S. Gov't
mlc1	mmrn1	http://www.ncbi.nlm.nih.gov/entrez/query.fcgi?cmd=Retrieve&db=pubmed&dopt=Abstract&list_uids=26906158|6|Invest Ophthalmol Vis Sci|57|2|Feb|2016|Journal Article,Research Support, Non-U.S. Gov't
brca1	nbr1	http://www.ncbi.nlm.nih.gov/entrez/query.fcgi?cmd=Retrieve&db=pubmed&dopt=Abstract&list_uids=26903539|6|J Cell Biol|212|5|Feb|2016|Journal Article,Research Support, N.I.H., Extramural,Research Support, U.S. Gov't, Non-P.H.S.
ndrg1	ptk2	http://www.ncbi.nlm.nih.gov/entrez/query.fcgi?cmd=Retrieve&db=pubmed&dopt=Abstract&list_uids=26895766|6|Mol Pharmacol|89|5|May|2016|Journal Article,Research Support, Non-U.S. Gov't
cdkn2a	asap1	http://www.ncbi.nlm.nih.gov/entrez/query.fcgi?cmd=Retrieve&db=pubmed&dopt=Abstract&list_uids=26893376|6|J Biol Chem|291|14|Apr|2016|Journal Article,Research Support, N.I.H., Extramural
cdkn2a	myh14	http://www.ncbi.nlm.nih.gov/entrez/query.fcgi?cmd=Retrieve&db=pubmed&dopt=Abstract&list_uids=26893376|6|J Biol Chem|291|14|Apr|2016|Journal Article,Research Support, N.I.H., Extramural
cdkn2a	3.6.4.1	http://www.ncbi.nlm.nih.gov/entrez/query.fcgi?cmd=Retrieve&db=pubmed&dopt=Abstract&list_uids=26893376|6|J Biol Chem|291|14|Apr|2016|Journal Article,Research Support, N.I.H., Extramural
asap1	myh14	http://www.ncbi.nlm.nih.gov/entrez/query.fcgi?cmd=Retrieve&db=pubmed&dopt=Abstract&list_uids=26893376|6|J Biol Chem|291|14|Apr|2016|Journal Article,Research Support, N.I.H., Extramural
asap1	3.6.4.1	http://www.ncbi.nlm.nih.gov/entrez/query.fcgi?cmd=Retrieve&db=pubmed&dopt=Abstract&list_uids=26893376|6|J Biol Chem|291|14|Apr|2016|Journal Article,Research Support, N.I.H., Extramural
myh14	3.6.4.1	http://www.ncbi.nlm.nih.gov/entrez/query.fcgi?cmd=Retrieve&db=pubmed&dopt=Abstract&list_uids=25550404|6|EMBO Rep|16|2|Feb|2015|Journal Article,Research Support, Non-U.S. Gov't|http://www.ncbi.nlm.nih.gov/entrez/query.fcgi?cmd=Retrieve&db=pubmed&dopt=Abstract&list_uids=23378028|6|J Cell Sci|126|Pt 5|Mar|2013|Journal Article,Research Support, Non-U.S. Gov't|http://www.ncbi.nlm.nih.gov/entrez/query.fcgi?cmd=Retrieve&db=pubmed&dopt=Abstract&list_uids=26893376|6|J Biol Chem|291|14|Apr|2016|Journal Article,Research Support, N.I.H., Extramural
atf2	ucp1	http://www.ncbi.nlm.nih.gov/entrez/query.fcgi?cmd=Retrieve&db=pubmed&dopt=Abstract&list_uids=26883894|6|Sci Rep|6|||2016|Journal Article,Research Support, Non-U.S. Gov't
ptk2	fat	http://www.ncbi.nlm.nih.gov/entrez/query.fcgi?cmd=Retrieve&db=pubmed&dopt=Abstract&list_uids=25391654|6|J Biol Chem|290|1|Jan|2015|Journal Article,Research Support, Non-U.S. Gov't|http://www.ncbi.nlm.nih.gov/entrez/query.fcgi?cmd=Retrieve&db=pubmed&dopt=Abstract&list_uids=26866573|6|Bio

		chemistry|55|9|Mar|2016|Journal Article,Research Support, N.I.H., Extramural,Research Support, U.S. Gov't, Non-P.H.S.|http://www.ncbi.nlm.nih.gov/entrez/query.fcgi?cmd=Retrieve&db=pubmed&dopt=Abstract&list_uids=25226367|6|PLoS One|9|9||2014|Journal Article,Research Support, Non-U.S. Gov't
ptk2	ptk2b	http://www.ncbi.nlm.nih.gov/entrez/query.fcgi?cmd=Retrieve&db=pubmed&dopt=Abstract&list_uids=24290759|6|Cell Rep|5|5|Dec|2013|Journal Article,Research Support, Non-U.S. Gov't|http://www.ncbi.nlm.nih.gov/entrez/query.fcgi?cmd=Retrieve&db=pubmed&dopt=Abstract&list_uids=22859931|6|PLoS One|7|6||2012|Journal Article,Research Support, N.I.H., Extramural,Research Support, Non-U.S. Gov't|http://www.ncbi.nlm.nih.gov/entrez/query.fcgi?cmd=Retrieve&db=pubmed&dopt=Abstract&list_uids=26866573|6|Biochemistry|55|9|Mar|2016|Journal Article,Research Support, N.I.H., Extramural,Research Support, U.S. Gov't, Non-P.H.S.|http://www.ncbi.nlm.nih.gov/entrez/query.fcgi?cmd=Retrieve&db=pubmed&dopt=Abstract&list_uids=25475728|6|Biochem Biophys Res Commun|459|1|Mar|2015|Journal Article,Research Support, Non-U.S. Gov't
fat	ptk2b	http://www.ncbi.nlm.nih.gov/entrez/query.fcgi?cmd=Retrieve&db=pubmed&dopt=Abstract&list_uids=26866573|6|Biochemistry|55|9|Mar|2016|Journal Article,Research Support, N.I.H., Extramural,Research Support, U.S. Gov't, Non-P.H.S.
stat3	tns4	http://www.ncbi.nlm.nih.gov/entrez/query.fcgi?cmd=Retrieve&db=pubmed&dopt=Abstract&list_uids=26852686|6|Int J Exp Pathol|96|6|Dec|2015|Journal Article,Research Support, Non-U.S. Gov't
src	ptk2	http://www.ncbi.nlm.nih.gov/entrez/query.fcgi?cmd=Retrieve&db=pubmed&dopt=Abstract&list_uids=22665056|6|Oncogene|32|14|Apr|2013|Journal Article,Research Support, Non-U.S. Gov't|http://www.ncbi.nlm.nih.gov/entrez/query.fcgi?cmd=Retrieve&db=pubmed&dopt=Abstract&list_uids=25049397|6|Proc Natl Acad Sci U S A|111|31|Aug|2014|Journal Article,Research Support, Non-U.S. Gov't|http://www.ncbi.nlm.nih.gov/entrez/query.fcgi?cmd=Retrieve&db=pubmed&dopt=Abstract&list_uids=22488171|6|J Cancer Res Clin Oncol|138|8|Aug|2012|Journal Article|http://www.ncbi.nlm.nih.gov/entrez/query.fcgi?cmd=Retrieve&db=pubmed&dopt=Abstract&list_uids=26079101|6|Biochim Biophys Acta|1853|10 Pt A|Oct|2015|Journal Article,Research Support, Non-U.S. Gov't|http://www.ncbi.nlm.nih.gov/entrez/query.fcgi?cmd=Retrieve&db=pubmed&dopt=Abstract&list_uids=23754369|6|Proc Natl Acad Sci U S A|110|26|Jun|2013|Journal Article,Research Support, N.I.H., Intramural,Research Support, Non-U.S. Gov't|http://www.ncbi.nlm.nih.gov/entrez/query.fcgi?cmd=Retrieve&db=pubmed&dopt=Abstract&list_uids=22900044|6|PLoS One|7|8||2012|Journal Article,Research Support, N.I.H., Extramural,Research Support, Non-U.S. Gov't,Research Support, U.S. Gov't, Non-P.H.S.|http://www.ncbi.nlm.nih.gov/entrez/query.fcgi?cmd=Retrieve&db=pubmed&dopt=Abstract&list_uids=22287273|6|J Cell Physiol|227|11|Nov|2012|Journal Article,Research Support, N.I.H., Extramural|http://www.ncbi.nlm.nih.gov/entrez/query.fcgi?cmd=Retrieve&db=pubmed&dopt=Abstract&list_uids=25530216|6|Cell Signal|27|3|Mar|2015|Journal Article,Research Support, Non-U.S. Gov't|http://www.ncbi.nlm.nih.gov/entrez/query.fcgi?cmd=Retrieve&db=pubmed&dopt=Abstract&list_uids=22732841|6|EMBO Rep|13|8|Aug|2012|Journal Article,Research Support, Non-U.S. Gov't|http://www.ncbi.nlm.nih.gov/entrez/query.fcgi?cmd=Retrieve&db=pubmed&dopt=Abstract&list_uids=22302985|6|J Cell Sci|125|Pt 2|Jan|2012|Journal Article,Research Support, N.I.H.,

		Extramural|http://www.ncbi.nlm.nih.gov/entrez/query.fcgi?cmd=Retrieve&db=pubmed&dopt=Abstract&list_uids=26833789|6|J Cell Biol|212|3|Feb|2016|Journal Article,Research Support, Non-U.S. Gov't,Webcasts|http://www.ncbi.nlm.nih.gov/entrez/query.fcgi?cmd=Retrieve&db=pubmed&dopt=Abstract&list_uids=26378057|6|Oncotarget|6|31|Oct|2015|Journal Article,Research Support, Non-U.S. Gov't|http://www.ncbi.nlm.nih.gov/entrez/query.fcgi?cmd=Retrieve&db=pubmed&dopt=Abstract&list_uids=26172215|6|Exp Cell Res|336|2|Aug|2015|Journal Article,Research Support, Non-U.S. Gov't|http://www.ncbi.nlm.nih.gov/entrez/query.fcgi?cmd=Retrieve&db=pubmed&dopt=Abstract&list_uids=25766369|6|J Cell Mol Med|19|6|Jun|2015|Journal Article,Research Support, Non-U.S. Gov't|http://www.ncbi.nlm.nih.gov/entrez/query.fcgi?cmd=Retrieve&db=pubmed&dopt=Abstract&list_uids=23209645|6|PLoS One|7|11||2012|Journal Article,Research Support, Non-U.S. Gov't|http://www.ncbi.nlm.nih.gov/entrez/query.fcgi?cmd=Retrieve&db=pubmed&dopt=Abstract&list_uids=26119091|6|Gene|570|1|Oct|2015|Journal Article,Research Support, N.I.H., Extramural,Research Support, Non-U.S. Gov't,Review|http://www.ncbi.nlm.nih.gov/entrez/query.fcgi?cmd=Retrieve&db=pubmed&dopt=Abstract&list_uids=25359883|6|J Cell Sci|127|Pt 24|Dec|2014|Journal Article,Research Support, Non-U.S. Gov't|http://www.ncbi.nlm.nih.gov/entrez/query.fcgi?cmd=Retrieve&db=pubmed&dopt=Abstract&list_uids=23836527|6|Stem Cells|31|11|Nov|2013|Journal Article,Research Support, N.I.H., Extramural|http://www.ncbi.nlm.nih.gov/entrez/query.fcgi?cmd=Retrieve&db=pubmed&dopt=Abstract&list_uids=24266736|6|Biochem J|458|1|Feb|2014|Journal Article,Research Support, Non-U.S. Gov't
uvrag	tns4	http://www.ncbi.nlm.nih.gov/entrez/query.fcgi?cmd=Retrieve&db=pubmed&dopt=Abstract&list_uids=26784942|6|PLoS One|11|1||2016|Journal Article,Research Support, Non-U.S. Gov't
mmrn1	ptk2	http://www.ncbi.nlm.nih.gov/entrez/query.fcgi?cmd=Retrieve&db=pubmed&dopt=Abstract&list_uids=23689132|6|Mol Cell Biol|33|14|Jul|2013|Journal Article,Research Support, N.I.H., Extramural,Research Support, Non-U.S. Gov't|http://www.ncbi.nlm.nih.gov/entrez/query.fcgi?cmd=Retrieve&db=pubmed&dopt=Abstract&list_uids=26745022|6|Cancer Invest|34|1||2016|Journal Article,Research Support, Non-U.S. Gov't|http://www.ncbi.nlm.nih.gov/entrez/query.fcgi?cmd=Retrieve&db=pubmed&dopt=Abstract&list_uids=24289792|6|Arthritis Res Ther|15|5||2013|Journal Article,Research Support, Non-U.S. Gov't
hgf	mbtps1	http://www.ncbi.nlm.nih.gov/entrez/query.fcgi?cmd=Retrieve&db=pubmed&dopt=Abstract&list_uids=26697169|6|Pulm Circ|5|4|Dec|2015|Journal Article
hgf	abl1	http://www.ncbi.nlm.nih.gov/entrez/query.fcgi?cmd=Retrieve&db=pubmed&dopt=Abstract&list_uids=26697169|6|Pulm Circ|5|4|Dec|2015|Journal Article
mbtps1	abl1	http://www.ncbi.nlm.nih.gov/entrez/query.fcgi?cmd=Retrieve&db=pubmed&dopt=Abstract&list_uids=26697169|6|Pulm Circ|5|4|Dec|2015|Journal Article
hgf	ing1	http://www.ncbi.nlm.nih.gov/entrez/query.fcgi?cmd=Retrieve&db=pubmed&dopt=Abstract&list_uids=26697169|6|Pulm Circ|5|4|Dec|2015|Journal Article
mbtps1	ing1	http://www.ncbi.nlm.nih.gov/entrez/query.fcgi?cmd=Retrieve&db=pubmed&dopt=Abstract&list_uids=26697169|6|Pulm Circ|5|4|Dec|2015|Journal Article

abl1	ing1	http://www.ncbi.nlm.nih.gov/entrez/query.fcgi?cmd=Retrieve&db=pubmed&dopt=Abstract&list_uids=26697169|6|Pulm Circ|5|4|Dec|2015|Journal Article
abl1	ros1	http://www.ncbi.nlm.nih.gov/entrez/query.fcgi?cmd=Retrieve&db=pubmed&dopt=Abstract&list_uids=26697169|6|Pulm Circ|5|4|Dec|2015|Journal Article
hgf	ros1	http://www.ncbi.nlm.nih.gov/entrez/query.fcgi?cmd=Retrieve&db=pubmed&dopt=Abstract&list_uids=26697169|6|Pulm Circ|5|4|Dec|2015|Journal Article
mbtps1	ros1	http://www.ncbi.nlm.nih.gov/entrez/query.fcgi?cmd=Retrieve&db=pubmed&dopt=Abstract&list_uids=26697169|6|Pulm Circ|5|4|Dec|2015|Journal Article
actin	p2rx7	http://www.ncbi.nlm.nih.gov/entrez/query.fcgi?cmd=Retrieve&db=pubmed&dopt=Abstract&list_uids=26683661|6|Am J Pathol|186|2|Feb|2016|Journal Article,Research Support, N.I.H., Extramural,Research Support, Non-U.S. Gov't
cd70	rnd3	http://www.ncbi.nlm.nih.gov/entrez/query.fcgi?cmd=Retrieve&db=pubmed&dopt=Abstract&list_uids=26671750|6|Br J Cancer|114|1|Jan|2016|Journal Article,Research Support, Non-U.S. Gov't
cd70	rock1	http://www.ncbi.nlm.nih.gov/entrez/query.fcgi?cmd=Retrieve&db=pubmed&dopt=Abstract&list_uids=26671750|6|Br J Cancer|114|1|Jan|2016|Journal Article,Research Support, Non-U.S. Gov't
cd70	ppp1r12a	http://www.ncbi.nlm.nih.gov/entrez/query.fcgi?cmd=Retrieve&db=pubmed&dopt=Abstract&list_uids=26671750|6|Br J Cancer|114|1|Jan|2016|Journal Article,Research Support, Non-U.S. Gov't
rnd3	rock1	http://www.ncbi.nlm.nih.gov/entrez/query.fcgi?cmd=Retrieve&db=pubmed&dopt=Abstract&list_uids=26671750|6|Br J Cancer|114|1|Jan|2016|Journal Article,Research Support, Non-U.S. Gov't
rnd3	ppp1r12a	http://www.ncbi.nlm.nih.gov/entrez/query.fcgi?cmd=Retrieve&db=pubmed&dopt=Abstract&list_uids=26671750|6|Br J Cancer|114|1|Jan|2016|Journal Article,Research Support, Non-U.S. Gov't
rock1	ppp1r12a	http://www.ncbi.nlm.nih.gov/entrez/query.fcgi?cmd=Retrieve&db=pubmed&dopt=Abstract&list_uids=26671750|6|Br J Cancer|114|1|Jan|2016|Journal Article,Research Support, Non-U.S. Gov't
ephb2	fhl2	http://www.ncbi.nlm.nih.gov/entrez/query.fcgi?cmd=Retrieve&db=pubmed&dopt=Abstract&list_uids=26658111|6|PLoS One|10|12||2015|Journal Article,Research Support, Non-U.S. Gov't
ephb2	pdlim5	http://www.ncbi.nlm.nih.gov/entrez/query.fcgi?cmd=Retrieve&db=pubmed&dopt=Abstract&list_uids=26658111|6|PLoS One|10|12||2015|Journal Article,Research Support, Non-U.S. Gov't
fhl2	pdlim5	http://www.ncbi.nlm.nih.gov/entrez/query.fcgi?cmd=Retrieve&db=pubmed&dopt=Abstract&list_uids=26658111|6|PLoS One|10|12||2015|Journal Article,Research Support, Non-U.S. Gov't
myh14	actin	http://www.ncbi.nlm.nih.gov/entrez/query.fcgi?cmd=Retrieve&db=pubmed&dopt=Abstract&list_uids=22237407|6|Am J Physiol Cell Physiol|302|7|Apr|2012|Journal Article,Research Support, N.I.H., Extramural,Research Support, N.I.H., Intramural|http://www.ncbi.nlm.nih.gov/entrez/query.fcgi?cmd=Retrieve&db=pubmed&dopt=Abstract&list_uids=24121284|6|Exp Physiol|99|3|Mar|2014|Journal Article,Research Support, N.I.H., Extramural,Review|http://www.ncbi.nlm.nih.gov/entrez/query.fcgi?cmd=Retrieve&db=pubmed&dopt=Abstract&list_uids=26652273|6|Elife|4|||2015|Journal Article,Research Support, Non-U.S. Gov't|http://www.ncbi.nlm.nih.gov/entrez/query.fcgi?cmd=Retrieve&db=pubmed&dopt=Abstract&list_uids=22707566|6|FASEB J|26|10|Oct|2012|Journal Article,Research Support, N.I.H., Extramural,Research Support, Non-U.S.

		Gov't|http://www.ncbi.nlm.nih.gov/entrez/query.fcgi?cmd=Retrieve&db=pubmed&dopt=Abstract&list_uids=25883213|6|Oncotarget|6|13|May|2015|Journal Article,Research Support, Non-U.S. Gov't|http://www.ncbi.nlm.nih.gov/entrez/query.fcgi?cmd=Retrieve&db=pubmed&dopt=Abstract&list_uids=23782578|6|Open Biol|3|6|Jun|2013|Journal Article,Research Support, Non-U.S. Gov't,Review|http://www.ncbi.nlm.nih.gov/entrez/query.fcgi?cmd=Retrieve&db=pubmed&dopt=Abstract&list_uids=23361003|6|Nat Commun|4|||2013|Journal Article,Research Support, Non-U.S. Gov't|http://www.ncbi.nlm.nih.gov/entrez/query.fcgi?cmd=Retrieve&db=pubmed&dopt=Abstract&list_uids=23378028|6|J Cell Sci|126|Pt 5|Mar|2013|Journal Article,Research Support, Non-U.S. Gov't|http://www.ncbi.nlm.nih.gov/entrez/query.fcgi?cmd=Retrieve&db=pubmed&dopt=Abstract&list_uids=23923013|6|PLoS One|8|7||2013|Journal Article,Research Support, Non-U.S. Gov't
myh14	prkaa1	http://www.ncbi.nlm.nih.gov/entrez/query.fcgi?cmd=Retrieve&db=pubmed&dopt=Abstract&list_uids=26652273|6|Elife|4|||2015|Journal Article,Research Support, Non-U.S. Gov't
myh14	vasp	http://www.ncbi.nlm.nih.gov/entrez/query.fcgi?cmd=Retrieve&db=pubmed&dopt=Abstract&list_uids=26652273|6|Elife|4|||2015|Journal Article,Research Support, Non-U.S. Gov't
actin	prkaa1	http://www.ncbi.nlm.nih.gov/entrez/query.fcgi?cmd=Retrieve&db=pubmed&dopt=Abstract&list_uids=26652273|6|Elife|4|||2015|Journal Article,Research Support, Non-U.S. Gov't
actin	vasp	http://www.ncbi.nlm.nih.gov/entrez/query.fcgi?cmd=Retrieve&db=pubmed&dopt=Abstract&list_uids=26652273|6|Elife|4|||2015|Journal Article,Research Support, Non-U.S. Gov't|http://www.ncbi.nlm.nih.gov/entrez/query.fcgi?cmd=Retrieve&db=pubmed&dopt=Abstract&list_uids=26295568|6|Elife|4|||2015|Journal Article,Research Support, N.I.H., Extramural,Research Support, Non-U.S. Gov't
prkaa1	vasp	http://www.ncbi.nlm.nih.gov/entrez/query.fcgi?cmd=Retrieve&db=pubmed&dopt=Abstract&list_uids=26652273|6|Elife|4|||2015|Journal Article,Research Support, Non-U.S. Gov't
pik3ca	akt1	http://www.ncbi.nlm.nih.gov/entrez/query.fcgi?cmd=Retrieve&db=pubmed&dopt=Abstract&list_uids=26626726|6|Stem Cell Res Ther|6|||2015|Journal Article,Research Support, N.I.H., Extramural,Research Support, Non-U.S. Gov't|http://www.ncbi.nlm.nih.gov/entrez/query.fcgi?cmd=Retrieve&db=pubmed&dopt=Abstract&list_uids=25588838|6|J Cell Sci|128|5|Mar|2015|Journal Article,Research Support, Non-U.S. Gov't|http://www.ncbi.nlm.nih.gov/entrez/query.fcgi?cmd=Retrieve&db=pubmed&dopt=Abstract&list_uids=24825896|6|J Biol Chem|289|26|Jun|2014|Journal Article,Research Support, N.I.H., Extramural,Research Support, Non-U.S. Gov't|http://www.ncbi.nlm.nih.gov/entrez/query.fcgi?cmd=Retrieve&db=pubmed&dopt=Abstract&list_uids=22767580|6|Mol Biol Cell|23|17|Sep|2012|Journal Article,Research Support, Non-U.S. Gov't
pik3ca	msc	http://www.ncbi.nlm.nih.gov/entrez/query.fcgi?cmd=Retrieve&db=pubmed&dopt=Abstract&list_uids=26626726|6|Stem Cell Res Ther|6|||2015|Journal Article,Research Support, N.I.H., Extramural,Research Support, Non-U.S. Gov't
akt1	msc	http://www.ncbi.nlm.nih.gov/entrez/query.fcgi?cmd=Retrieve&db=pubmed&dopt=Abstract&list_uids=26626726|6|Stem Cell Res Ther|6|||2015|Journal Article,Research Support, N.I.H., Extramural,Research Support, Non-U.S. Gov't
1.1.3.6	cav1	http://www.ncbi.nlm.nih.gov/entrez/query.fcgi?cmd=Retrieve&db=pubmed&dopt=Abstract&list_uids=26626726|6|Stem Cell Res Ther|6|||2015|Journal Article,Research Support, N.I.H., Extramural,Research Support, Non-U.S. Gov't

1.1.3.6	pik3ca	http://www.ncbi.nlm.nih.gov/entrez/query.fcgi?cmd=Retrieve&db=pubmed&dopt=Abstract&list_uids=26626726|6|Stem Cell Res Ther|6|||2015|Journal Article,Research Support, N.I.H., Extramural,Research Support, Non-U.S. Gov't
1.1.3.6	akt1	http://www.ncbi.nlm.nih.gov/entrez/query.fcgi?cmd=Retrieve&db=pubmed&dopt=Abstract&list_uids=26626726|6|Stem Cell Res Ther|6|||2015|Journal Article,Research Support, N.I.H., Extramural,Research Support, Non-U.S. Gov't
1.1.3.6	msc	http://www.ncbi.nlm.nih.gov/entrez/query.fcgi?cmd=Retrieve&db=pubmed&dopt=Abstract&list_uids=26626726|6|Stem Cell Res Ther|6|||2015|Journal Article,Research Support, N.I.H., Extramural,Research Support, Non-U.S. Gov't
cav1	pik3ca	http://www.ncbi.nlm.nih.gov/entrez/query.fcgi?cmd=Retrieve&db=pubmed&dopt=Abstract&list_uids=26626726|6|Stem Cell Res Ther|6|||2015|Journal Article,Research Support, N.I.H., Extramural,Research Support, Non-U.S. Gov't
cav1	akt1	http://www.ncbi.nlm.nih.gov/entrez/query.fcgi?cmd=Retrieve&db=pubmed&dopt=Abstract&list_uids=26626726|6|Stem Cell Res Ther|6|||2015|Journal Article,Research Support, N.I.H., Extramural,Research Support, Non-U.S. Gov't
cav1	msc	http://www.ncbi.nlm.nih.gov/entrez/query.fcgi?cmd=Retrieve&db=pubmed&dopt=Abstract&list_uids=26626726|6|Stem Cell Res Ther|6|||2015|Journal Article,Research Support, N.I.H., Extramural,Research Support, Non-U.S. Gov't
lpa	ptk2	http://www.ncbi.nlm.nih.gov/entrez/query.fcgi?cmd=Retrieve&db=pubmed&dopt=Abstract&list_uids=26609804|6|Int Endod J|||Nov|2015|JOURNAL ARTICLE|http://www.ncbi.nlm.nih.gov/entrez/query.fcgi?cmd=Retrieve&db=pubmed&dopt=Abstract&list_uids=24061591|6|Dig Dis Sci|58|12|Dec|2013|Journal Article,Research Support, Non-U.S. Gov't
ptk2	pxn	http://www.ncbi.nlm.nih.gov/entrez/query.fcgi?cmd=Retrieve&db=pubmed&dopt=Abstract&list_uids=26546726|6|Exp Eye Res|145||Apr|2016|Journal Article
taf9	moxd1	http://www.ncbi.nlm.nih.gov/entrez/query.fcgi?cmd=Retrieve&db=pubmed&dopt=Abstract&list_uids=26546726|6|Exp Eye Res|145||Apr|2016|Journal Article
taf9	pxn	http://www.ncbi.nlm.nih.gov/entrez/query.fcgi?cmd=Retrieve&db=pubmed&dopt=Abstract&list_uids=26546726|6|Exp Eye Res|145||Apr|2016|Journal Article
moxd1	pxn	http://www.ncbi.nlm.nih.gov/entrez/query.fcgi?cmd=Retrieve&db=pubmed&dopt=Abstract&list_uids=26546726|6|Exp Eye Res|145||Apr|2016|Journal Article
taf9	ptk2	http://www.ncbi.nlm.nih.gov/entrez/query.fcgi?cmd=Retrieve&db=pubmed&dopt=Abstract&list_uids=26546726|6|Exp Eye Res|145||Apr|2016|Journal Article
moxd1	ptk2	http://www.ncbi.nlm.nih.gov/entrez/query.fcgi?cmd=Retrieve&db=pubmed&dopt=Abstract&list_uids=26546726|6|Exp Eye Res|145||Apr|2016|Journal Article
itgax	itgb2	http://www.ncbi.nlm.nih.gov/entrez/query.fcgi?cmd=Retrieve&db=pubmed&dopt=Abstract&list_uids=26519532|6|J Immunol|195|11|Dec|2015|Journal Article,Research Support, N.I.H., Extramural
itgax	vcam1	http://www.ncbi.nlm.nih.gov/entrez/query.fcgi?cmd=Retrieve&db=pubmed&dopt=Abstract&list_uids=26519532|6|J Immunol|195|11|Dec|2015|Journal Article,Research Support, N.I.H., Extramural
itgb2	vcam1	http://www.ncbi.nlm.nih.gov/entrez/query.fcgi?cmd=Retrieve&db=pubmed&dopt=Abstract&list_uids=26519532|6|J Immunol|195|11|Dec|2015|Journal Article,Research Support, N.I.H., Extramural
igf1	mmp2	http://www.ncbi.nlm.nih.gov/entrez/query.fcgi?cmd=Retrieve&db=pubmed&dopt=Abstract&list_uids=26513500|6|Invest Ophthalmol Vis Sci|56|11|Oct|2015|Journal Article,Research Support, Non-U.S. Gov't

actin	igf1	http://www.ncbi.nlm.nih.gov/entrez/query.fcgi?cmd=Retrieve&db=pubmed&dopt=Abstract&list_uids=26513500|6|Invest Ophthalmol Vis Sci|56|11|Oct|2015|Journal Article,Research Support, Non-U.S. Gov't|http://www.ncbi.nlm.nih.gov/entrez/query.fcgi?cmd=Retrieve&db=pubmed&dopt=Abstract&list_uids=23023326|6|Int J Oncol|41|6|Dec|2012|Journal Article,Research Support, Non-U.S. Gov't
actin	igf1r	http://www.ncbi.nlm.nih.gov/entrez/query.fcgi?cmd=Retrieve&db=pubmed&dopt=Abstract&list_uids=26513500|6|Invest Ophthalmol Vis Sci|56|11|Oct|2015|Journal Article,Research Support, Non-U.S. Gov't
igf1	igf1r	http://www.ncbi.nlm.nih.gov/entrez/query.fcgi?cmd=Retrieve&db=pubmed&dopt=Abstract&list_uids=26513500|6|Invest Ophthalmol Vis Sci|56|11|Oct|2015|Journal Article,Research Support, Non-U.S. Gov't|http://www.ncbi.nlm.nih.gov/entrez/query.fcgi?cmd=Retrieve&db=pubmed&dopt=Abstract&list_uids=22859931|6|PLoS One|7|6||2012|Journal Article,Research Support, N.I.H., Extramural,Research Support, Non-U.S. Gov't
mmp2	igf1r	http://www.ncbi.nlm.nih.gov/entrez/query.fcgi?cmd=Retrieve&db=pubmed&dopt=Abstract&list_uids=26513500|6|Invest Ophthalmol Vis Sci|56|11|Oct|2015|Journal Article,Research Support, Non-U.S. Gov't
igf1	pik3ca	http://www.ncbi.nlm.nih.gov/entrez/query.fcgi?cmd=Retrieve&db=pubmed&dopt=Abstract&list_uids=26513500|6|Invest Ophthalmol Vis Sci|56|11|Oct|2015|Journal Article,Research Support, Non-U.S. Gov't
mmp2	pik3ca	http://www.ncbi.nlm.nih.gov/entrez/query.fcgi?cmd=Retrieve&db=pubmed&dopt=Abstract&list_uids=26513500|6|Invest Ophthalmol Vis Sci|56|11|Oct|2015|Journal Article,Research Support, Non-U.S. Gov't
flnb	actin	http://www.ncbi.nlm.nih.gov/entrez/query.fcgi?cmd=Retrieve&db=pubmed&dopt=Abstract&list_uids=26491051|6|Am J Physiol Cell Physiol|310|1|Jan|2016|Journal Article,Research Support, N.I.H., Extramural,Research Support, Non-U.S. Gov't
myh14	cdkn1a	http://www.ncbi.nlm.nih.gov/entrez/query.fcgi?cmd=Retrieve&db=pubmed&dopt=Abstract&list_uids=26491051|6|Am J Physiol Cell Physiol|310|1|Jan|2016|Journal Article,Research Support, N.I.H., Extramural,Research Support, Non-U.S. Gov't
asprv1	rhoa	http://www.ncbi.nlm.nih.gov/entrez/query.fcgi?cmd=Retrieve&db=pubmed&dopt=Abstract&list_uids=26483365|6|Oncotarget|6|31|Oct|2015|Journal Article,Research Support, N.I.H., Extramural
pdlim7	itk	http://www.ncbi.nlm.nih.gov/entrez/query.fcgi?cmd=Retrieve&db=pubmed&dopt=Abstract&list_uids=26479443|6|Oncogenesis|4|||2015|Journal Article
pdlim7	src	http://www.ncbi.nlm.nih.gov/entrez/query.fcgi?cmd=Retrieve&db=pubmed&dopt=Abstract&list_uids=26479443|6|Oncogenesis|4|||2015|Journal Article
pdlim7	npy6r	http://www.ncbi.nlm.nih.gov/entrez/query.fcgi?cmd=Retrieve&db=pubmed&dopt=Abstract&list_uids=26479443|6|Oncogenesis|4|||2015|Journal Article
src	npy6r	http://www.ncbi.nlm.nih.gov/entrez/query.fcgi?cmd=Retrieve&db=pubmed&dopt=Abstract&list_uids=26479443|6|Oncogenesis|4|||2015|Journal Article|http://www.ncbi.nlm.nih.gov/entrez/query.fcgi?cmd=Retrieve&db=pubmed&dopt=Abstract&list_uids=22287273|6|J Cell Physiol|227|11|Nov|2012|Journal Article,Research Support, N.I.H., Extramural|http://www.ncbi.nlm.nih.gov/entrez/query.fcgi?cmd=Retrieve&db=pubmed&dopt=Abstract&list_uids=22505999|6|PLoS One|7|4||2012|Journal Article,Research Support, N.I.H., Extramural,Research Support, Non-U.S. Gov't|http://www.ncbi.nlm.nih.gov/entrez/query.fcgi?cmd=Retrieve&db=pubmed&dopt=Abstract&list_uids=25707853|6|Biochem Biophys Res Commun|458|4|Mar|2015|Journal Article,Research Support, Non-U.S. Gov't

pdlim7	npc1	http://www.ncbi.nlm.nih.gov/entrez/query.fcgi?cmd=Retrieve&db=pubmed&dopt=Abstract&list_uids=26479443|6|Oncogenesis|4|||2015|Journal Article
src	mirn27b	http://www.ncbi.nlm.nih.gov/entrez/query.fcgi?cmd=Retrieve&db=pubmed&dopt=Abstract&list_uids=26473412|6|Cancer Sci|107|1|Jan|2016|Journal Article,Research Support, Non-U.S. Gov't
src	hras	http://www.ncbi.nlm.nih.gov/entrez/query.fcgi?cmd=Retrieve&db=pubmed&dopt=Abstract&list_uids=26473412|6|Cancer Sci|107|1|Jan|2016|Journal Article,Research Support, Non-U.S. Gov't
mirn27b	hras	http://www.ncbi.nlm.nih.gov/entrez/query.fcgi?cmd=Retrieve&db=pubmed&dopt=Abstract&list_uids=26473412|6|Cancer Sci|107|1|Jan|2016|Journal Article,Research Support, Non-U.S. Gov't
arfgef1	mirn27b	http://www.ncbi.nlm.nih.gov/entrez/query.fcgi?cmd=Retrieve&db=pubmed&dopt=Abstract&list_uids=26473412|6|Cancer Sci|107|1|Jan|2016|Journal Article,Research Support, Non-U.S. Gov't
ptk2	rab21	http://www.ncbi.nlm.nih.gov/entrez/query.fcgi?cmd=Retrieve&db=pubmed&dopt=Abstract&list_uids=26436690|6|Nat Cell Biol|17|11|Nov|2015|Journal Article,Research Support, Non-U.S. Gov't
arhgap1	dlc1	http://www.ncbi.nlm.nih.gov/entrez/query.fcgi?cmd=Retrieve&db=pubmed&dopt=Abstract&list_uids=26427649|6|Biochim Biophys Acta|1853|12|Dec|2015|Journal Article,Research Support, N.I.H., Extramural
arhgap1	rhoa	http://www.ncbi.nlm.nih.gov/entrez/query.fcgi?cmd=Retrieve&db=pubmed&dopt=Abstract&list_uids=26427649|6|Biochim Biophys Acta|1853|12|Dec|2015|Journal Article,Research Support, N.I.H., Extramural
arhgap1	actin	http://www.ncbi.nlm.nih.gov/entrez/query.fcgi?cmd=Retrieve&db=pubmed&dopt=Abstract&list_uids=26427649|6|Biochim Biophys Acta|1853|12|Dec|2015|Journal Article,Research Support, N.I.H., Extramural
dlc1	rhoa	http://www.ncbi.nlm.nih.gov/entrez/query.fcgi?cmd=Retrieve&db=pubmed&dopt=Abstract&list_uids=26427649|6|Biochim Biophys Acta|1853|12|Dec|2015|Journal Article,Research Support, N.I.H., Extramural|http://www.ncbi.nlm.nih.gov/entrez/query.fcgi?cmd=Retrieve&db=pubmed&dopt=Abstract&list_uids=22307599|6|Proc Natl Acad Sci U S A|109|5|Jan|2012|Journal Article,Research Support, Non-U.S. Gov't
dlc1	actin	http://www.ncbi.nlm.nih.gov/entrez/query.fcgi?cmd=Retrieve&db=pubmed&dopt=Abstract&list_uids=26427649|6|Biochim Biophys Acta|1853|12|Dec|2015|Journal Article,Research Support, N.I.H., Extramural|http://www.ncbi.nlm.nih.gov/entrez/query.fcgi?cmd=Retrieve&db=pubmed&dopt=Abstract&list_uids=22307599|6|Proc Natl Acad Sci U S A|109|5|Jan|2012|Journal Article,Research Support, Non-U.S. Gov't
tns1	actin	http://www.ncbi.nlm.nih.gov/entrez/query.fcgi?cmd=Retrieve&db=pubmed&dopt=Abstract&list_uids=26427649|6|Biochim Biophys Acta|1853|12|Dec|2015|Journal Article,Research Support, N.I.H., Extramural
dlc1	tns1	http://www.ncbi.nlm.nih.gov/entrez/query.fcgi?cmd=Retrieve&db=pubmed&dopt=Abstract&list_uids=26427649|6|Biochim Biophys Acta|1853|12|Dec|2015|Journal Article,Research Support, N.I.H., Extramural
dlc1	ptbp1	http://www.ncbi.nlm.nih.gov/entrez/query.fcgi?cmd=Retrieve&db=pubmed&dopt=Abstract&list_uids=26427649|6|Biochim Biophys Acta|1853|12|Dec|2015|Journal Article,Research Support, N.I.H., Extramural
tns1	ptbp1	http://www.ncbi.nlm.nih.gov/entrez/query.fcgi?cmd=Retrieve&db=pubmed&dopt=Abstract&list_uids=26427649|6|Biochim Biophys Acta|1853|12|Dec|2015|Journal Article,Research Support, N.I.H., Extramural
tns1	arhgap1	http://www.ncbi.nlm.nih.gov/entrez/query.fcgi?cmd=Retrieve&db=pubmed&dopt=Abstract&list_uids=26427649|6|Biochim Biophys Acta|1853|12|Dec|2015|Journal Article,Research Support, N.I.H., Extramural

tns1	rhoa	http://www.ncbi.nlm.nih.gov/entrez/query.fcgi?cmd=Retrieve&db=pubmed&dopt=Abstract&list_uids=26427649|6|Biochim Biophys Acta|1853|12|Dec|2015|Journal Article,Research Support, N.I.H., Extramural
jak2	ghr	http://www.ncbi.nlm.nih.gov/entrez/query.fcgi?cmd=Retrieve&db=pubmed&dopt=Abstract&list_uids=26421979|6|Growth Horm IGF Res|28||Jun|2016|Journal Article
soat1	shc1	http://www.ncbi.nlm.nih.gov/entrez/query.fcgi?cmd=Retrieve&db=pubmed&dopt=Abstract&list_uids=26421979|6|Growth Horm IGF Res|28||Jun|2016|Journal Article
soat1	akt1	http://www.ncbi.nlm.nih.gov/entrez/query.fcgi?cmd=Retrieve&db=pubmed&dopt=Abstract&list_uids=26421979|6|Growth Horm IGF Res|28||Jun|2016|Journal Article
soat1	ptpn11	http://www.ncbi.nlm.nih.gov/entrez/query.fcgi?cmd=Retrieve&db=pubmed&dopt=Abstract&list_uids=26421979|6|Growth Horm IGF Res|28||Jun|2016|Journal Article
soat1	sh2b1	http://www.ncbi.nlm.nih.gov/entrez/query.fcgi?cmd=Retrieve&db=pubmed&dopt=Abstract&list_uids=26421979|6|Growth Horm IGF Res|28||Jun|2016|Journal Article
soat1	jak2	http://www.ncbi.nlm.nih.gov/entrez/query.fcgi?cmd=Retrieve&db=pubmed&dopt=Abstract&list_uids=26421979|6|Growth Horm IGF Res|28||Jun|2016|Journal Article
soat1	actin	http://www.ncbi.nlm.nih.gov/entrez/query.fcgi?cmd=Retrieve&db=pubmed&dopt=Abstract&list_uids=26421979|6|Growth Horm IGF Res|28||Jun|2016|Journal Article
shc1	akt1	http://www.ncbi.nlm.nih.gov/entrez/query.fcgi?cmd=Retrieve&db=pubmed&dopt=Abstract&list_uids=26421979|6|Growth Horm IGF Res|28||Jun|2016|Journal Article
shc1	ptpn11	http://www.ncbi.nlm.nih.gov/entrez/query.fcgi?cmd=Retrieve&db=pubmed&dopt=Abstract&list_uids=26421979|6|Growth Horm IGF Res|28||Jun|2016|Journal Article
shc1	sh2b1	http://www.ncbi.nlm.nih.gov/entrez/query.fcgi?cmd=Retrieve&db=pubmed&dopt=Abstract&list_uids=26421979|6|Growth Horm IGF Res|28||Jun|2016|Journal Article
shc1	jak2	http://www.ncbi.nlm.nih.gov/entrez/query.fcgi?cmd=Retrieve&db=pubmed&dopt=Abstract&list_uids=26421979|6|Growth Horm IGF Res|28||Jun|2016|Journal Article
shc1	actin	http://www.ncbi.nlm.nih.gov/entrez/query.fcgi?cmd=Retrieve&db=pubmed&dopt=Abstract&list_uids=25096985|6|Horm Cancer|5|6|Dec|2014|Journal Article,Research Support, N.I.H., Extramural,Research Support, Non-U.S. Gov't|http://www.ncbi.nlm.nih.gov/entrez/query.fcgi?cmd=Retrieve&db=pubmed&dopt=Abstract&list_uids=26421979|6|Growth Horm IGF Res|28||Jun|2016|Journal Article
akt1	ptpn11	http://www.ncbi.nlm.nih.gov/entrez/query.fcgi?cmd=Retrieve&db=pubmed&dopt=Abstract&list_uids=26421979|6|Growth Horm IGF Res|28||Jun|2016|Journal Article
akt1	sh2b1	http://www.ncbi.nlm.nih.gov/entrez/query.fcgi?cmd=Retrieve&db=pubmed&dopt=Abstract&list_uids=26421979|6|Growth Horm IGF Res|28||Jun|2016|Journal Article
akt1	jak2	http://www.ncbi.nlm.nih.gov/entrez/query.fcgi?cmd=Retrieve&db=pubmed&dopt=Abstract&list_uids=24861878|6|Neuro Oncol|16|9|Sep|2014|Journal Article,Research Support, Non-U.S. Gov't|http://www.ncbi.nlm.nih.gov/entrez/query.fcgi?cmd=Retrieve&db=pubmed&dopt=Abstract&list_uids=26421979|6|Growth Horm IGF Res|28||Jun|2016|Journal Article

akt1	actin	http://www.ncbi.nlm.nih.gov/entrez/query.fcgi?cmd=Retrieve&db=pubmed&dopt=Abstract&list_uids=24635319|6|Biochem J|459|3|May|2014|Journal Article,Research Support, Non-U.S. Gov't|http://www.ncbi.nlm.nih.gov/entrez/query.fcgi?cmd=Retrieve&db=pubmed&dopt=Abstract&list_uids=26421979|6|Growth Horm IGF Res|28||Jun|2016|Journal Article
ptpn11	sh2b1	http://www.ncbi.nlm.nih.gov/entrez/query.fcgi?cmd=Retrieve&db=pubmed&dopt=Abstract&list_uids=26421979|6|Growth Horm IGF Res|28||Jun|2016|Journal Article
ptpn11	jak2	http://www.ncbi.nlm.nih.gov/entrez/query.fcgi?cmd=Retrieve&db=pubmed&dopt=Abstract&list_uids=26421979|6|Growth Horm IGF Res|28||Jun|2016|Journal Article
ptpn11	actin	http://www.ncbi.nlm.nih.gov/entrez/query.fcgi?cmd=Retrieve&db=pubmed&dopt=Abstract&list_uids=26421979|6|Growth Horm IGF Res|28||Jun|2016|Journal Article
sh2b1	jak2	http://www.ncbi.nlm.nih.gov/entrez/query.fcgi?cmd=Retrieve&db=pubmed&dopt=Abstract&list_uids=26421979|6|Growth Horm IGF Res|28||Jun|2016|Journal Article
sh2b1	actin	http://www.ncbi.nlm.nih.gov/entrez/query.fcgi?cmd=Retrieve&db=pubmed&dopt=Abstract&list_uids=26421979|6|Growth Horm IGF Res|28||Jun|2016|Journal Article
jak2	actin	http://www.ncbi.nlm.nih.gov/entrez/query.fcgi?cmd=Retrieve&db=pubmed&dopt=Abstract&list_uids=26421979|6|Growth Horm IGF Res|28||Jun|2016|Journal Article
itga11	fgf2	http://www.ncbi.nlm.nih.gov/entrez/query.fcgi?cmd=Retrieve&db=pubmed&dopt=Abstract&list_uids=26403263|6|J Cell Biochem|117|4|Apr|2016|Journal Article,Research Support, N.I.H., Extramural
itga11	mapk3	http://www.ncbi.nlm.nih.gov/entrez/query.fcgi?cmd=Retrieve&db=pubmed&dopt=Abstract&list_uids=26403263|6|J Cell Biochem|117|4|Apr|2016|Journal Article,Research Support, N.I.H., Extramural
fgf2	mapk3	http://www.ncbi.nlm.nih.gov/entrez/query.fcgi?cmd=Retrieve&db=pubmed&dopt=Abstract&list_uids=26403263|6|J Cell Biochem|117|4|Apr|2016|Journal Article,Research Support, N.I.H., Extramural
cyth2	rras	http://www.ncbi.nlm.nih.gov/entrez/query.fcgi?cmd=Retrieve&db=pubmed&dopt=Abstract&list_uids=26378252|6|Mol Biol Cell|26|23|Nov|2015|Journal Article,Research Support, N.I.H., Extramural,Research Support, Non-U.S. Gov't
cyth2	cdkn2a	http://www.ncbi.nlm.nih.gov/entrez/query.fcgi?cmd=Retrieve&db=pubmed&dopt=Abstract&list_uids=26378252|6|Mol Biol Cell|26|23|Nov|2015|Journal Article,Research Support, N.I.H., Extramural,Research Support, Non-U.S. Gov't
rras	cdkn2a	http://www.ncbi.nlm.nih.gov/entrez/query.fcgi?cmd=Retrieve&db=pubmed&dopt=Abstract&list_uids=26378252|6|Mol Biol Cell|26|23|Nov|2015|Journal Article,Research Support, N.I.H., Extramural,Research Support, Non-U.S. Gov't
arhgef2	cyth2	http://www.ncbi.nlm.nih.gov/entrez/query.fcgi?cmd=Retrieve&db=pubmed&dopt=Abstract&list_uids=26378252|6|Mol Biol Cell|26|23|Nov|2015|Journal Article,Research Support, N.I.H., Extramural,Research Support, Non-U.S. Gov't
arhgef2	rras	http://www.ncbi.nlm.nih.gov/entrez/query.fcgi?cmd=Retrieve&db=pubmed&dopt=Abstract&list_uids=26378252|6|Mol Biol Cell|26|23|Nov|2015|Journal Article,Research Support, N.I.H., Extramural,Research Support, Non-U.S. Gov't
arhgef2	ehd1	http://www.ncbi.nlm.nih.gov/entrez/query.fcgi?cmd=Retrieve&db=pubmed&dopt=Abstract&list_uids=26378252|6|Mol Biol Cell|26|23|Nov|2015|Journal Article,Research Support, N.I.H., Extramural,Research Support, Non-U.S. Gov't
cyth2	ehd1	http://www.ncbi.nlm.nih.gov/entrez/query.fcgi?cmd=Retrieve&db=pubmed&dopt=Abstract&list_uids=26378252|6|Mol Biol Cell|26|23|Nov|2015|Journal Article,Research Support, N.I.H., Extramural,Research Support, Non-U.S. Gov't

rras	ehd1	http://www.ncbi.nlm.nih.gov/entrez/query.fcgi?cmd=Retrieve&db=pubmed&dopt=Abstract&list_uids=26378252|6|Mol Biol Cell|26|23|Nov|2015|Journal Article,Research Support, N.I.H., Extramural,Research Support, Non-U.S. Gov't
mapk14	sgsm3	http://www.ncbi.nlm.nih.gov/entrez/query.fcgi?cmd=Retrieve&db=pubmed&dopt=Abstract&list_uids=26373535|6|BMC Cancer|15|||2015|Journal Article,Research Support, Non-U.S. Gov't
itgb5	itgb6	http://www.ncbi.nlm.nih.gov/entrez/query.fcgi?cmd=Retrieve&db=pubmed&dopt=Abstract&list_uids=26359297|6|J Cell Sci|128|21|Nov|2015|Journal Article,Research Support, N.I.H., Extramural
vasp	enah	http://www.ncbi.nlm.nih.gov/entrez/query.fcgi?cmd=Retrieve&db=pubmed&dopt=Abstract&list_uids=26295568|6|Elife|4|||2015|Journal Article,Research Support, N.I.H., Extramural,Research Support, Non-U.S. Gov't|http://www.ncbi.nlm.nih.gov/entrez/query.fcgi?cmd=Retrieve&db=pubmed&dopt=Abstract&list_uids=25848013|6|Proc Natl Acad Sci U S A|112|16|Apr|2015|Journal Article,Research Support, Non-U.S. Gov't|http://www.ncbi.nlm.nih.gov/entrez/query.fcgi?cmd=Retrieve&db=pubmed&dopt=Abstract&list_uids=26358985|6|Microbiology|161|11|Nov|2015|Journal Article,Research Support, N.I.H., Extramural,Research Support, Non-U.S. Gov't|http://www.ncbi.nlm.nih.gov/entrez/query.fcgi?cmd=Retrieve&db=pubmed&dopt=Abstract&list_uids=22456508|6|Mol Biol Cell|23|10|May|2012|Journal Article,Research Support, N.I.H., Extramural,Research Support, Non-U.S. Gov't
vasp	evl	http://www.ncbi.nlm.nih.gov/entrez/query.fcgi?cmd=Retrieve&db=pubmed&dopt=Abstract&list_uids=26358985|6|Microbiology|161|11|Nov|2015|Journal Article,Research Support, N.I.H., Extramural,Research Support, Non-U.S. Gov't
ptk2b	ccr7	http://www.ncbi.nlm.nih.gov/entrez/query.fcgi?cmd=Retrieve&db=pubmed&dopt=Abstract&list_uids=26352169|6|Oncol Rep|34|6|Dec|2015|Journal Article,Research Support, Non-U.S. Gov't
braf	nras	http://www.ncbi.nlm.nih.gov/entrez/query.fcgi?cmd=Retrieve&db=pubmed&dopt=Abstract&list_uids=26339916|6|Magy Onkol|59|3|Sep|2015|English Abstract,Journal Article
mirn34b	mirn34c	http://www.ncbi.nlm.nih.gov/entrez/query.fcgi?cmd=Retrieve&db=pubmed&dopt=Abstract&list_uids=26330189|6|Oncol Rep|34|5|Nov|2015|Journal Article,Research Support, Non-U.S. Gov't
mirn34b	mirn18a	http://www.ncbi.nlm.nih.gov/entrez/query.fcgi?cmd=Retrieve&db=pubmed&dopt=Abstract&list_uids=26330189|6|Oncol Rep|34|5|Nov|2015|Journal Article,Research Support, Non-U.S. Gov't
mirn34b	mirn31	http://www.ncbi.nlm.nih.gov/entrez/query.fcgi?cmd=Retrieve&db=pubmed&dopt=Abstract&list_uids=26330189|6|Oncol Rep|34|5|Nov|2015|Journal Article,Research Support, Non-U.S. Gov't
mirn34b	tp53	http://www.ncbi.nlm.nih.gov/entrez/query.fcgi?cmd=Retrieve&db=pubmed&dopt=Abstract&list_uids=26330189|6|Oncol Rep|34|5|Nov|2015|Journal Article,Research Support, Non-U.S. Gov't
mirn34b	npc1	http://www.ncbi.nlm.nih.gov/entrez/query.fcgi?cmd=Retrieve&db=pubmed&dopt=Abstract&list_uids=26330189|6|Oncol Rep|34|5|Nov|2015|Journal Article,Research Support, Non-U.S. Gov't
mirn34c	mirn18a	http://www.ncbi.nlm.nih.gov/entrez/query.fcgi?cmd=Retrieve&db=pubmed&dopt=Abstract&list_uids=26330189|6|Oncol Rep|34|5|Nov|2015|Journal Article,Research Support, Non-U.S. Gov't
mirn34c	mirn31	http://www.ncbi.nlm.nih.gov/entrez/query.fcgi?cmd=Retrieve&db=pubmed&dopt=Abstract&list_uids=26330189|6|Oncol Rep|34|5|Nov|2015|Journal Article,Research Support, Non-U.S. Gov't
mirn34c	tp53	http://www.ncbi.nlm.nih.gov/entrez/query.fcgi?cmd=Retrieve&db=pubmed&dopt=Abstract&list_uids=26330189|6|Oncol Rep|34|5|Nov|2015|Journal Article,Research Support, Non-U.S. Gov't

mirn34c	npc1	http://www.ncbi.nlm.nih.gov/entrez/query.fcgi?cmd=Retrieve&db=pubmed&dopt=Abstract&list_uids=26330189|6|Oncol Rep|34|5|Nov|2015|Journal Article,Research Support, Non-U.S. Gov't
mirn18a	mirn31	http://www.ncbi.nlm.nih.gov/entrez/query.fcgi?cmd=Retrieve&db=pubmed&dopt=Abstract&list_uids=26330189|6|Oncol Rep|34|5|Nov|2015|Journal Article,Research Support, Non-U.S. Gov't
mirn18a	tp53	http://www.ncbi.nlm.nih.gov/entrez/query.fcgi?cmd=Retrieve&db=pubmed&dopt=Abstract&list_uids=26330189|6|Oncol Rep|34|5|Nov|2015|Journal Article,Research Support, Non-U.S. Gov't
mirn18a	npc1	http://www.ncbi.nlm.nih.gov/entrez/query.fcgi?cmd=Retrieve&db=pubmed&dopt=Abstract&list_uids=26330189|6|Oncol Rep|34|5|Nov|2015|Journal Article,Research Support, Non-U.S. Gov't
mirn31	tp53	http://www.ncbi.nlm.nih.gov/entrez/query.fcgi?cmd=Retrieve&db=pubmed&dopt=Abstract&list_uids=26330189|6|Oncol Rep|34|5|Nov|2015|Journal Article,Research Support, Non-U.S. Gov't
mirn31	npc1	http://www.ncbi.nlm.nih.gov/entrez/query.fcgi?cmd=Retrieve&db=pubmed&dopt=Abstract&list_uids=26330189|6|Oncol Rep|34|5|Nov|2015|Journal Article,Research Support, Non-U.S. Gov't
tp53	npc1	http://www.ncbi.nlm.nih.gov/entrez/query.fcgi?cmd=Retrieve&db=pubmed&dopt=Abstract&list_uids=26330189|6|Oncol Rep|34|5|Nov|2015|Journal Article,Research Support, Non-U.S. Gov't
rab11a	rac1	http://www.ncbi.nlm.nih.gov/entrez/query.fcgi?cmd=Retrieve&db=pubmed&dopt=Abstract&list_uids=26299802|6|Eur J Cell Biol|94|11|Nov|2015|Journal Article,Research Support, Non-U.S. Gov't
rab11a	ptk2	http://www.ncbi.nlm.nih.gov/entrez/query.fcgi?cmd=Retrieve&db=pubmed&dopt=Abstract&list_uids=26299802|6|Eur J Cell Biol|94|11|Nov|2015|Journal Article,Research Support, Non-U.S. Gov't
rab11a	fas	http://www.ncbi.nlm.nih.gov/entrez/query.fcgi?cmd=Retrieve&db=pubmed&dopt=Abstract&list_uids=26299802|6|Eur J Cell Biol|94|11|Nov|2015|Journal Article,Research Support, Non-U.S. Gov't
rac1	fas	http://www.ncbi.nlm.nih.gov/entrez/query.fcgi?cmd=Retrieve&db=pubmed&dopt=Abstract&list_uids=25820249|6|J Cell Physiol|230|11|Nov|2015|Journal Article,Research Support, Non-U.S. Gov't|http://www.ncbi.nlm.nih.gov/entrez/query.fcgi?cmd=Retrieve&db=pubmed&dopt=Abstract&list_uids=25544611|6|Curr Biol|25|2|Jan|2015|Journal Article,Research Support, N.I.H., Extramural,Research Support, N.I.H., Intramural|http://www.ncbi.nlm.nih.gov/entrez/query.fcgi?cmd=Retrieve&db=pubmed&dopt=Abstract&list_uids=23344455|6|Toxins (Basel)|5|1|Jan|2013|Comparative Study,Journal Article,Research Support, Non-U.S. Gov't|http://www.ncbi.nlm.nih.gov/entrez/query.fcgi?cmd=Retrieve&db=pubmed&dopt=Abstract&list_uids=23295182|6|J Biol Chem|288|8|Feb|2013|Journal Article,Research Support, N.I.H., Intramural|http://www.ncbi.nlm.nih.gov/entrez/query.fcgi?cmd=Retrieve&db=pubmed&dopt=Abstract&list_uids=26299802|6|Eur J Cell Biol|94|11|Nov|2015|Journal Article,Research Support, Non-U.S. Gov't
ptk2	fas	http://www.ncbi.nlm.nih.gov/entrez/query.fcgi?cmd=Retrieve&db=pubmed&dopt=Abstract&list_uids=25820249|6|J Cell Physiol|230|11|Nov|2015|Journal Article,Research Support, Non-U.S. Gov't|http://www.ncbi.nlm.nih.gov/entrez/query.fcgi?cmd=Retrieve&db=pubmed&dopt=Abstract&list_uids=22399300|6|J Biol Chem|287|19|May|2012|Journal Article,Research Support, Non-U.S. Gov't|http://www.ncbi.nlm.nih.gov/entrez/query.fcgi?cmd=Retrieve&db=pubmed&dopt=Abstract&list_uids=24642576|6|PLoS One|9|3||2014|Journal Article,Research Support, Non-U.S.

		Gov't|http://www.ncbi.nlm.nih.gov/entrez/query.fcgi?cmd=Retrieve&db=pubmed&dopt=Abstract&list_uids=23754369|6|Proc Natl Acad Sci U S A|110|26|Jun|2013|Journal Article,Research Support, N.I.H., Intramural,Research Support, Non-U.S. Gov't|http://www.ncbi.nlm.nih.gov/entrez/query.fcgi?cmd=Retrieve&db=pubmed&dopt=Abstract&list_uids=26172215|6|Exp Cell Res|336|2|Aug|2015|Journal Article,Research Support, Non-U.S. Gov't|http://www.ncbi.nlm.nih.gov/entrez/query.fcgi?cmd=Retrieve&db=pubmed&dopt=Abstract&list_uids=25391654|6|J Biol Chem|290|1|Jan|2015|Journal Article,Research Support, Non-U.S. Gov't|http://www.ncbi.nlm.nih.gov/entrez/query.fcgi?cmd=Retrieve&db=pubmed&dopt=Abstract&list_uids=26299802|6|Eur J Cell Biol|94|11|Nov|2015|Journal Article,Research Support, Non-U.S. Gov't|http://www.ncbi.nlm.nih.gov/entrez/query.fcgi?cmd=Retrieve&db=pubmed&dopt=Abstract&list_uids=25942420|6|PLoS One|10|5||2015|Journal Article,Research Support, Non-U.S. Gov't|http://www.ncbi.nlm.nih.gov/entrez/query.fcgi?cmd=Retrieve&db=pubmed&dopt=Abstract&list_uids=23836527|6|Stem Cells|31|11|Nov|2013|Journal Article,Research Support, N.I.H., Extramural|http://www.ncbi.nlm.nih.gov/entrez/query.fcgi?cmd=Retrieve&db=pubmed&dopt=Abstract&list_uids=24486488|6|Biochem Biophys Res Commun|444|4|Feb|2014|Journal Article,Research Support, N.I.H., Extramural,Research Support, Non-U.S. Gov't
actin	enah	http://www.ncbi.nlm.nih.gov/entrez/query.fcgi?cmd=Retrieve&db=pubmed&dopt=Abstract&list_uids=26295568|6|Elife|4|||2015|Journal Article,Research Support, N.I.H., Extramural,Research Support, Non-U.S. Gov't
enah	pgk1	http://www.ncbi.nlm.nih.gov/entrez/query.fcgi?cmd=Retrieve&db=pubmed&dopt=Abstract&list_uids=26295568|6|Elife|4|||2015|Journal Article,Research Support, N.I.H., Extramural,Research Support, Non-U.S. Gov't
enah	apbb1ip	http://www.ncbi.nlm.nih.gov/entrez/query.fcgi?cmd=Retrieve&db=pubmed&dopt=Abstract&list_uids=26295568|6|Elife|4|||2015|Journal Article,Research Support, N.I.H., Extramural,Research Support, Non-U.S. Gov't
vasp	pgk1	http://www.ncbi.nlm.nih.gov/entrez/query.fcgi?cmd=Retrieve&db=pubmed&dopt=Abstract&list_uids=26295568|6|Elife|4|||2015|Journal Article,Research Support, N.I.H., Extramural,Research Support, Non-U.S. Gov't
vasp	apbb1ip	http://www.ncbi.nlm.nih.gov/entrez/query.fcgi?cmd=Retrieve&db=pubmed&dopt=Abstract&list_uids=26295568|6|Elife|4|||2015|Journal Article,Research Support, N.I.H., Extramural,Research Support, Non-U.S. Gov't
pgk1	apbb1ip	http://www.ncbi.nlm.nih.gov/entrez/query.fcgi?cmd=Retrieve&db=pubmed&dopt=Abstract&list_uids=26295568|6|Elife|4|||2015|Journal Article,Research Support, N.I.H., Extramural,Research Support, Non-U.S. Gov't
actin	raph1	http://www.ncbi.nlm.nih.gov/entrez/query.fcgi?cmd=Retrieve&db=pubmed&dopt=Abstract&list_uids=26295568|6|Elife|4|||2015|Journal Article,Research Support, N.I.H., Extramural,Research Support, Non-U.S. Gov't
raph1	vasp	http://www.ncbi.nlm.nih.gov/entrez/query.fcgi?cmd=Retrieve&db=pubmed&dopt=Abstract&list_uids=26295568|6|Elife|4|||2015|Journal Article,Research Support, N.I.H., Extramural,Research Support, Non-U.S. Gov't
raph1	ervk2	http://www.ncbi.nlm.nih.gov/entrez/query.fcgi?cmd=Retrieve&db=pubmed&dopt=Abstract&list_uids=26295568|6|Elife|4|||2015|Journal Article,Research Support, N.I.H., Extramural,Research Support, Non-U.S. Gov't
actin	ervk2	http://www.ncbi.nlm.nih.gov/entrez/query.fcgi?cmd=Retrieve&db=pubmed&dopt=Abstract&list_uids=26295568|6|Elife|4|||2015|Journal Article,Research Support, N.I.H., Extramural,Research Support, Non-U.S. Gov't
vasp	ervk2	http://www.ncbi.nlm.nih.gov/entrez/query.fcgi?cmd=Retrieve&db=pubmed&dopt=Abstract&list_uids=26295568|6|Elife|4|||2

		015|Journal Article,Research Support, N.I.H., Extramural,Research Support, Non-U.S. Gov't
sparcl1	hdac9	http://www.ncbi.nlm.nih.gov/entrez/query.fcgi?cmd=Retrieve&db=pubmed&dopt=Abstract&list_uids=26294211|6|Cancer Res|75|20|Oct|2015|Journal Article,Research Support, N.I.H., Extramural,Research Support, Non-U.S. Gov't
sparcl1	mmrn1	http://www.ncbi.nlm.nih.gov/entrez/query.fcgi?cmd=Retrieve&db=pubmed&dopt=Abstract&list_uids=26294211|6|Cancer Res|75|20|Oct|2015|Journal Article,Research Support, N.I.H., Extramural,Research Support, Non-U.S. Gov't
jun	mapk8	http://www.ncbi.nlm.nih.gov/entrez/query.fcgi?cmd=Retrieve&db=pubmed&dopt=Abstract&list_uids=24894398|6|Invest Ophthalmol Vis Sci|55|7|Jul|2014|Journal Article,Research Support, Non-U.S. Gov't|http://www.ncbi.nlm.nih.gov/entrez/query.fcgi?cmd=Retrieve&db=pubmed&dopt=Abstract&list_uids=25586177|6|J Biol Chem|290|10|Mar|2015|Journal Article,Research Support, Non-U.S. Gov't|http://www.ncbi.nlm.nih.gov/entrez/query.fcgi?cmd=Retrieve&db=pubmed&dopt=Abstract&list_uids=25237887|6|PLoS One|9|9||2014|Journal Article,Research Support, Non-U.S. Gov't|http://www.ncbi.nlm.nih.gov/entrez/query.fcgi?cmd=Retrieve&db=pubmed&dopt=Abstract&list_uids=24193434|6|Cell Adh Migr|7|5||2013|Journal Article,Research Support, Non-U.S. Gov't|http://www.ncbi.nlm.nih.gov/entrez/query.fcgi?cmd=Retrieve&db=pubmed&dopt=Abstract&list_uids=25190348|6|Biochem J|464|1|Nov|2014|Journal Article,Research Support, N.I.H., Extramural,Research Support, Non-U.S. Gov't|http://www.ncbi.nlm.nih.gov/entrez/query.fcgi?cmd=Retrieve&db=pubmed&dopt=Abstract&list_uids=26255846|6|Curr Biol|25|17|Aug|2015|Journal Article,Research Support, N.I.H., Extramural,Research Support, Non-U.S. Gov't
jun	lims1	http://www.ncbi.nlm.nih.gov/entrez/query.fcgi?cmd=Retrieve&db=pubmed&dopt=Abstract&list_uids=26255846|6|Curr Biol|25|17|Aug|2015|Journal Article,Research Support, N.I.H., Extramural,Research Support, Non-U.S. Gov't
mapk8	lims1	http://www.ncbi.nlm.nih.gov/entrez/query.fcgi?cmd=Retrieve&db=pubmed&dopt=Abstract&list_uids=26255846|6|Curr Biol|25|17|Aug|2015|Journal Article,Research Support, N.I.H., Extramural,Research Support, Non-U.S. Gov't
arf6	arhgef2	http://www.ncbi.nlm.nih.gov/entrez/query.fcgi?cmd=Retrieve&db=pubmed&dopt=Abstract&list_uids=26239146|6|Nat Commun|6|||2015|Journal Article,Research Support, Non-U.S. Gov't
arf6	cyth3	http://www.ncbi.nlm.nih.gov/entrez/query.fcgi?cmd=Retrieve&db=pubmed&dopt=Abstract&list_uids=26239146|6|Nat Commun|6|||2015|Journal Article,Research Support, Non-U.S. Gov't
arhgef2	cyth3	http://www.ncbi.nlm.nih.gov/entrez/query.fcgi?cmd=Retrieve&db=pubmed&dopt=Abstract&list_uids=26239146|6|Nat Commun|6|||2015|Journal Article,Research Support, Non-U.S. Gov't
hgf	arf6	http://www.ncbi.nlm.nih.gov/entrez/query.fcgi?cmd=Retrieve&db=pubmed&dopt=Abstract&list_uids=26239146|6|Nat Commun|6|||2015|Journal Article,Research Support, Non-U.S. Gov't
eng	ptk2	http://www.ncbi.nlm.nih.gov/entrez/query.fcgi?cmd=Retrieve&db=pubmed&dopt=Abstract&list_uids=26230358|6|Stem Cells Dev|24|21|Nov|2015|Journal Article,Research Support, Non-U.S. Gov't
mapk3	eng	http://www.ncbi.nlm.nih.gov/entrez/query.fcgi?cmd=Retrieve&db=pubmed&dopt=Abstract&list_uids=26230358|6|Stem Cells Dev|24|21|Nov|2015|Journal Article,Research Support, Non-U.S. Gov't
mapk3	mapk14	http://www.ncbi.nlm.nih.gov/entrez/query.fcgi?cmd=Retrieve&db=pubmed&dopt=Abstract&list_uids=26230358|6|Stem Cells Dev|24|21|Nov|2015|Journal Article,Research Support, Non-U.S. Gov't
mapk3	ptk2	http://www.ncbi.nlm.nih.gov/entrez/query.fcgi?cmd=Retrieve&db=pubmed&dopt=Abstract&list_uids=26230358|6|Stem

		Cells Dev|24|21|Nov|2015|Journal Article,Research Support, Non-U.S. Gov't|http://www.ncbi.nlm.nih.gov/entrez/query.fcgi?cmd=Retrieve&db=pubmed&dopt=Abstract&list_uids=22287273|6|J Cell Physiol|227|11|Nov|2012|Journal Article,Research Support, N.I.H., Extramural
eng	mapk14	http://www.ncbi.nlm.nih.gov/entrez/query.fcgi?cmd=Retrieve&db=pubmed&dopt=Abstract&list_uids=26230358|6|Stem Cells Dev|24|21|Nov|2015|Journal Article,Research Support, Non-U.S. Gov't
mapk14	ptk2	http://www.ncbi.nlm.nih.gov/entrez/query.fcgi?cmd=Retrieve&db=pubmed&dopt=Abstract&list_uids=26230358|6|Stem Cells Dev|24|21|Nov|2015|Journal Article,Research Support, Non-U.S. Gov't
elmo1	src	http://www.ncbi.nlm.nih.gov/entrez/query.fcgi?cmd=Retrieve&db=pubmed&dopt=Abstract&list_uids=26205662|6|Cell Commun Signal|13|||2015|Journal Article,Research Support, Non-U.S. Gov't
elmo1	rac1	http://www.ncbi.nlm.nih.gov/entrez/query.fcgi?cmd=Retrieve&db=pubmed&dopt=Abstract&list_uids=26205662|6|Cell Commun Signal|13|||2015|Journal Article,Research Support, Non-U.S. Gov't
src	rac1	http://www.ncbi.nlm.nih.gov/entrez/query.fcgi?cmd=Retrieve&db=pubmed&dopt=Abstract&list_uids=26205662|6|Cell Commun Signal|13|||2015|Journal Article,Research Support, Non-U.S. Gov't
src	hck	http://www.ncbi.nlm.nih.gov/entrez/query.fcgi?cmd=Retrieve&db=pubmed&dopt=Abstract&list_uids=26205662|6|Cell Commun Signal|13|||2015|Journal Article,Research Support, Non-U.S. Gov't
hck	elmo1	http://www.ncbi.nlm.nih.gov/entrez/query.fcgi?cmd=Retrieve&db=pubmed&dopt=Abstract&list_uids=26205662|6|Cell Commun Signal|13|||2015|Journal Article,Research Support, Non-U.S. Gov't
hck	rac1	http://www.ncbi.nlm.nih.gov/entrez/query.fcgi?cmd=Retrieve&db=pubmed&dopt=Abstract&list_uids=26205662|6|Cell Commun Signal|13|||2015|Journal Article,Research Support, Non-U.S. Gov't
elmo1	mmrn1	http://www.ncbi.nlm.nih.gov/entrez/query.fcgi?cmd=Retrieve&db=pubmed&dopt=Abstract&list_uids=26205662|6|Cell Commun Signal|13|||2015|Journal Article,Research Support, Non-U.S. Gov't
elmo1	fyn	http://www.ncbi.nlm.nih.gov/entrez/query.fcgi?cmd=Retrieve&db=pubmed&dopt=Abstract&list_uids=26205662|6|Cell Commun Signal|13|||2015|Journal Article,Research Support, Non-U.S. Gov't
rac1	mmrn1	http://www.ncbi.nlm.nih.gov/entrez/query.fcgi?cmd=Retrieve&db=pubmed&dopt=Abstract&list_uids=25544611|6|Curr Biol|25|2|Jan|2015|Journal Article,Research Support, N.I.H., Extramural,Research Support, N.I.H., Intramural|http://www.ncbi.nlm.nih.gov/entrez/query.fcgi?cmd=Retrieve&db=pubmed&dopt=Abstract&list_uids=26205662|6|Cell Commun Signal|13|||2015|Journal Article,Research Support, Non-U.S. Gov't
rac1	fyn	http://www.ncbi.nlm.nih.gov/entrez/query.fcgi?cmd=Retrieve&db=pubmed&dopt=Abstract&list_uids=26205662|6|Cell Commun Signal|13|||2015|Journal Article,Research Support, Non-U.S. Gov't
mmrn1	src	http://www.ncbi.nlm.nih.gov/entrez/query.fcgi?cmd=Retrieve&db=pubmed&dopt=Abstract&list_uids=26205662|6|Cell Commun Signal|13|||2015|Journal Article,Research Support, Non-U.S. Gov't
mmrn1	fyn	http://www.ncbi.nlm.nih.gov/entrez/query.fcgi?cmd=Retrieve&db=pubmed&dopt=Abstract&list_uids=26205662|6|Cell Commun Signal|13|||2015|Journal Article,Research Support, Non-U.S. Gov't
src	fyn	http://www.ncbi.nlm.nih.gov/entrez/query.fcgi?cmd=Retrieve&db=pubmed&dopt=Abstract&list_uids=23836527|6|Stem Cells|31|11|Nov|2013|Journal Article,Research Support, N.I.H., Extramural|http://www.ncbi.nlm.nih.gov/entrez/query.fcgi?cmd=Retrieve&db=pubmed&dopt=Abstract&list_uids=26205662

		|6|Cell Commun Signal|13|||2015|Journal Article,Research Support, Non-U.S. Gov't|http://www.ncbi.nlm.nih.gov/entrez/query.fcgi?cmd=Retrieve&db=pubmed&dopt=Abstract&list_uids=25694432|6|J Biol Chem|290|15|Apr|2015|Journal Article,Research Support, Non-U.S. Gov't
elmo1	actin	http://www.ncbi.nlm.nih.gov/entrez/query.fcgi?cmd=Retrieve&db=pubmed&dopt=Abstract&list_uids=26205662|6|Cell Commun Signal|13|||2015|Journal Article,Research Support, Non-U.S. Gov't
sema3c	vegfa	http://www.ncbi.nlm.nih.gov/entrez/query.fcgi?cmd=Retrieve&db=pubmed&dopt=Abstract&list_uids=26194913|6|EMBO Mol Med|7|10|Oct|2015|Journal Article,Research Support, Non-U.S. Gov't
sema3c	akt1	http://www.ncbi.nlm.nih.gov/entrez/query.fcgi?cmd=Retrieve&db=pubmed&dopt=Abstract&list_uids=26194913|6|EMBO Mol Med|7|10|Oct|2015|Journal Article,Research Support, Non-U.S. Gov't
sema3c	ptk2	http://www.ncbi.nlm.nih.gov/entrez/query.fcgi?cmd=Retrieve&db=pubmed&dopt=Abstract&list_uids=26194913|6|EMBO Mol Med|7|10|Oct|2015|Journal Article,Research Support, Non-U.S. Gov't
vegfa	akt1	http://www.ncbi.nlm.nih.gov/entrez/query.fcgi?cmd=Retrieve&db=pubmed&dopt=Abstract&list_uids=26194913|6|EMBO Mol Med|7|10|Oct|2015|Journal Article,Research Support, Non-U.S. Gov't
vegfa	ptk2	http://www.ncbi.nlm.nih.gov/entrez/query.fcgi?cmd=Retrieve&db=pubmed&dopt=Abstract&list_uids=25820249|6|J Cell Physiol|230|11|Nov|2015|Journal Article,Research Support, Non-U.S. Gov't|http://www.ncbi.nlm.nih.gov/entrez/query.fcgi?cmd=Retrieve&db=pubmed&dopt=Abstract&list_uids=26194913|6|EMBO Mol Med|7|10|Oct|2015|Journal Article,Research Support, Non-U.S. Gov't
cd99	slc3a2	http://www.ncbi.nlm.nih.gov/entrez/query.fcgi?cmd=Retrieve&db=pubmed&dopt=Abstract&list_uids=26172215|6|Exp Cell Res|336|2|Aug|2015|Journal Article,Research Support, Non-U.S. Gov't
cd99	ptpn11	http://www.ncbi.nlm.nih.gov/entrez/query.fcgi?cmd=Retrieve&db=pubmed&dopt=Abstract&list_uids=26172215|6|Exp Cell Res|336|2|Aug|2015|Journal Article,Research Support, Non-U.S. Gov't
cd99	ptk2	http://www.ncbi.nlm.nih.gov/entrez/query.fcgi?cmd=Retrieve&db=pubmed&dopt=Abstract&list_uids=26172215|6|Exp Cell Res|336|2|Aug|2015|Journal Article,Research Support, Non-U.S. Gov't
slc3a2	ptpn11	http://www.ncbi.nlm.nih.gov/entrez/query.fcgi?cmd=Retrieve&db=pubmed&dopt=Abstract&list_uids=26172215|6|Exp Cell Res|336|2|Aug|2015|Journal Article,Research Support, Non-U.S. Gov't
slc3a2	ptk2	http://www.ncbi.nlm.nih.gov/entrez/query.fcgi?cmd=Retrieve&db=pubmed&dopt=Abstract&list_uids=26172215|6|Exp Cell Res|336|2|Aug|2015|Journal Article,Research Support, Non-U.S. Gov't
ptpn11	ptk2	http://www.ncbi.nlm.nih.gov/entrez/query.fcgi?cmd=Retrieve&db=pubmed&dopt=Abstract&list_uids=23512980|6|Mol Cancer Res|11|6|Jun|2013|Journal Article,Research Support, N.I.H., Extramural,Research Support, U.S. Gov't, Non-P.H.S.|http://www.ncbi.nlm.nih.gov/entrez/query.fcgi?cmd=Retrieve&db=pubmed&dopt=Abstract&list_uids=26172215|6|Exp Cell Res|336|2|Aug|2015|Journal Article,Research Support, Non-U.S. Gov't
slc3a2	fas	http://www.ncbi.nlm.nih.gov/entrez/query.fcgi?cmd=Retrieve&db=pubmed&dopt=Abstract&list_uids=26172215|6|Exp Cell Res|336|2|Aug|2015|Journal Article,Research Support, Non-U.S. Gov't
cd99	src	http://www.ncbi.nlm.nih.gov/entrez/query.fcgi?cmd=Retrieve&db=pubmed&dopt=Abstract&list_uids=26172215|6|Exp Cell Res|336|2|Aug|2015|Journal Article,Research Support, Non-U.S. Gov't
src	ptpn11	http://www.ncbi.nlm.nih.gov/entrez/query.fcgi?cmd=Retrieve&db=pubmed&dopt=Abstract&list_uids=26172215|6|Exp Cell

		Res|336|2|Aug|2015|Journal Article,Research Support, Non-U.S. Gov't
pip5k3	fas	http://www.ncbi.nlm.nih.gov/entrez/query.fcgi?cmd=Retrieve&db=pubmed&dopt=Abstract&list_uids=26149501|6|Biochim Biophys Acta|1853|10 Pt A|Oct|2015|Journal Article,Research Support, Non-U.S. Gov't
akt1	egf	http://www.ncbi.nlm.nih.gov/entrez/query.fcgi?cmd=Retrieve&db=pubmed&dopt=Abstract&list_uids=26149501|6|Biochim Biophys Acta|1853|10 Pt A|Oct|2015|Journal Article,Research Support, Non-U.S. Gov't
akt1	fas	http://www.ncbi.nlm.nih.gov/entrez/query.fcgi?cmd=Retrieve&db=pubmed&dopt=Abstract&list_uids=26149501|6|Biochim Biophys Acta|1853|10 Pt A|Oct|2015|Journal Article,Research Support, Non-U.S. Gov't
ptk2	actin	http://www.ncbi.nlm.nih.gov/entrez/query.fcgi?cmd=Retrieve&db=pubmed&dopt=Abstract&list_uids=24635319|6|Biochem J|459|3|May|2014|Journal Article,Research Support, Non-U.S. Gov't|http://www.ncbi.nlm.nih.gov/entrez/query.fcgi?cmd=Retrieve&db=pubmed&dopt=Abstract&list_uids=24550267|6|Proc Natl Acad Sci U S A|111|7|Feb|2014|Journal Article,Research Support, N.I.H., Extramural,Research Support, Non-U.S. Gov't,Research Support, U.S. Gov't, Non-P.H.S.|http://www.ncbi.nlm.nih.gov/entrez/query.fcgi?cmd=Retrieve&db=pubmed&dopt=Abstract&list_uids=23273897|6|Curr Biol|23|2|Jan|2013|Journal Article,Research Support, Non-U.S. Gov't|http://www.ncbi.nlm.nih.gov/entrez/query.fcgi?cmd=Retrieve&db=pubmed&dopt=Abstract&list_uids=26046934|6|PLoS One|10|6||2015|Journal Article,Research Support, Non-U.S. Gov't|http://www.ncbi.nlm.nih.gov/entrez/query.fcgi?cmd=Retrieve&db=pubmed&dopt=Abstract&list_uids=26119091|6|Gene|570|1|Oct|2015|Journal Article,Research Support, N.I.H., Extramural,Research Support, Non-U.S. Gov't,Review|http://www.ncbi.nlm.nih.gov/entrez/query.fcgi?cmd=Retrieve&db=pubmed&dopt=Abstract&list_uids=25359883|6|J Cell Sci|127|Pt 24|Dec|2014|Journal Article,Research Support, Non-U.S. Gov't|http://www.ncbi.nlm.nih.gov/entrez/query.fcgi?cmd=Retrieve&db=pubmed&dopt=Abstract&list_uids=24486488|6|Biochem Biophys Res Commun|444|4|Feb|2014|Journal Article,Research Support, N.I.H., Extramural,Research Support, Non-U.S. Gov't|http://www.ncbi.nlm.nih.gov/entrez/query.fcgi?cmd=Retrieve&db=pubmed&dopt=Abstract&list_uids=26078334|6|Oncotarget|6|18|Jun|2015|Journal Article,Research Support, Non-U.S. Gov't|http://www.ncbi.nlm.nih.gov/entrez/query.fcgi?cmd=Retrieve&db=pubmed&dopt=Abstract&list_uids=24463198|6|Chem Biol Interact|211||Mar|2014|Journal Article,Review|http://www.ncbi.nlm.nih.gov/entrez/query.fcgi?cmd=Retrieve&db=pubmed&dopt=Abstract&list_uids=22542712|6|Exp Mol Pathol|93|1|Aug|2012|Journal Article,Research Support, N.I.H., Extramural
src	actin	http://www.ncbi.nlm.nih.gov/entrez/query.fcgi?cmd=Retrieve&db=pubmed&dopt=Abstract&list_uids=23091071|6|J Cell Biol|199|3|Oct|2012|Journal Article,Research Support, Non-U.S. Gov't|http://www.ncbi.nlm.nih.gov/entrez/query.fcgi?cmd=Retrieve&db=pubmed&dopt=Abstract&list_uids=24587062|6|PLoS One|9|2||2014|Journal Article,Research Support, N.I.H., Extramural,Research Support, Non-U.S. Gov't|http://www.ncbi.nlm.nih.gov/entrez/query.fcgi?cmd=Retrieve&db=pubmed&dopt=Abstract&list_uids=24928898|6|J Cell Sci|127|Pt 16|Aug|2014|Journal Article,Research Support, Non-U.S. Gov't|http://www.ncbi.nlm.nih.gov/entrez/query.fcgi?cmd=Retrieve&db=pubmed&dopt=Abstract&list_uids=26119091|6|Ge

		ne|570|1|Oct|2015|Journal Article,Research Support, N.I.H., Extramural,Research Support, Non-U.S. Gov't,Review|http://www.ncbi.nlm.nih.gov/entrez/query.fcgi?cmd=Retrieve&db=pubmed&dopt=Abstract&list_uids=25359883|6|J Cell Sci|127|Pt 24|Dec|2014|Journal Article,Research Support, Non-U.S. Gov't|http://www.ncbi.nlm.nih.gov/entrez/query.fcgi?cmd=Retrieve&db=pubmed&dopt=Abstract&list_uids=23023326|6|Int J Oncol|41|6|Dec|2012|Journal Article,Research Support, Non-U.S. Gov't|http://www.ncbi.nlm.nih.gov/entrez/query.fcgi?cmd=Retrieve&db=pubmed&dopt=Abstract&list_uids=24036928|6|Cell Cycle|12|21|Nov|2013|Journal Article,Research Support, N.I.H., Extramural,Research Support, Non-U.S. Gov't
actin	wasf2	http://www.ncbi.nlm.nih.gov/entrez/query.fcgi?cmd=Retrieve&db=pubmed&dopt=Abstract&list_uids=26116771|6|Biochem Biophys Res Commun|464|1|Aug|2015|Journal Article,Research Support, Non-U.S. Gov't
igf2	h19	http://www.ncbi.nlm.nih.gov/entrez/query.fcgi?cmd=Retrieve&db=pubmed&dopt=Abstract&list_uids=26116569|6|J Cell Sci|128|16|Aug|2015|Journal Article,Research Support, Non-U.S. Gov't
evc2	sgcb	http://www.ncbi.nlm.nih.gov/entrez/query.fcgi?cmd=Retrieve&db=pubmed&dopt=Abstract&list_uids=26114488|6|Invest Ophthalmol Vis Sci|56|6|Jun|2015|Journal Article
evc2	myh14	http://www.ncbi.nlm.nih.gov/entrez/query.fcgi?cmd=Retrieve&db=pubmed&dopt=Abstract&list_uids=26114488|6|Invest Ophthalmol Vis Sci|56|6|Jun|2015|Journal Article
evc2	myl2	http://www.ncbi.nlm.nih.gov/entrez/query.fcgi?cmd=Retrieve&db=pubmed&dopt=Abstract&list_uids=26114488|6|Invest Ophthalmol Vis Sci|56|6|Jun|2015|Journal Article
evc2	edn1	http://www.ncbi.nlm.nih.gov/entrez/query.fcgi?cmd=Retrieve&db=pubmed&dopt=Abstract&list_uids=26114488|6|Invest Ophthalmol Vis Sci|56|6|Jun|2015|Journal Article
myh14	myl2	http://www.ncbi.nlm.nih.gov/entrez/query.fcgi?cmd=Retrieve&db=pubmed&dopt=Abstract&list_uids=26114488|6|Invest Ophthalmol Vis Sci|56|6|Jun|2015|Journal Article|http://www.ncbi.nlm.nih.gov/entrez/query.fcgi?cmd=Retrieve&db=pubmed&dopt=Abstract&list_uids=24699667|6|PLoS One|9|4||2014|Journal Article,Research Support, Non-U.S. Gov't
myh14	edn1	http://www.ncbi.nlm.nih.gov/entrez/query.fcgi?cmd=Retrieve&db=pubmed&dopt=Abstract&list_uids=26114488|6|Invest Ophthalmol Vis Sci|56|6|Jun|2015|Journal Article
myl2	edn1	http://www.ncbi.nlm.nih.gov/entrez/query.fcgi?cmd=Retrieve&db=pubmed&dopt=Abstract&list_uids=26114488|6|Invest Ophthalmol Vis Sci|56|6|Jun|2015|Journal Article
f2	nr3c2	http://www.ncbi.nlm.nih.gov/entrez/query.fcgi?cmd=Retrieve&db=pubmed&dopt=Abstract&list_uids=26085308|6|J Cell Biochem|116|11|Nov|2015|Journal Article,Research Support, Non-U.S. Gov't
egf	rapgef5	http://www.ncbi.nlm.nih.gov/entrez/query.fcgi?cmd=Retrieve&db=pubmed&dopt=Abstract&list_uids=26079101|6|Biochim Biophys Acta|1853|10 Pt A|Oct|2015|Journal Article,Research Support, Non-U.S. Gov't
egf	egfr	http://www.ncbi.nlm.nih.gov/entrez/query.fcgi?cmd=Retrieve&db=pubmed&dopt=Abstract&list_uids=24335959|6|Cancer Res|74|3|Feb|2014|Journal Article,Research Support, Non-U.S. Gov't|http://www.ncbi.nlm.nih.gov/entrez/query.fcgi?cmd=Retrieve&db=pubmed&dopt=Abstract&list_uids=24505297|6|PLoS One|9|2||2014|Journal Article,Research Support, Non-U.S. Gov't|http://www.ncbi.nlm.nih.gov/entrez/query.fcgi?cmd=Retrieve&db=pubmed&dopt=Abstract&list_uids=24091658|6|Cel

		l Death Dis|4|||2013|Journal Article,Research Support, Non-U.S. Gov't|http://www.ncbi.nlm.nih.gov/entrez/query.fcgi?cmd=Retrieve&db=pubmed&dopt=Abstract&list_uids=26079101|6|Biochim Biophys Acta|1853|10 Pt A|Oct|2015|Journal Article,Research Support, Non-U.S. Gov't
src	egf	http://www.ncbi.nlm.nih.gov/entrez/query.fcgi?cmd=Retrieve&db=pubmed&dopt=Abstract&list_uids=25530216|6|Cell Signal|27|3|Mar|2015|Journal Article,Research Support, Non-U.S. Gov't|http://www.ncbi.nlm.nih.gov/entrez/query.fcgi?cmd=Retrieve&db=pubmed&dopt=Abstract&list_uids=26079101|6|Biochim Biophys Acta|1853|10 Pt A|Oct|2015|Journal Article,Research Support, Non-U.S. Gov't|http://www.ncbi.nlm.nih.gov/entrez/query.fcgi?cmd=Retrieve&db=pubmed&dopt=Abstract&list_uids=23023326|6|Int J Oncol|41|6|Dec|2012|Journal Article,Research Support, Non-U.S. Gov't|http://www.ncbi.nlm.nih.gov/entrez/query.fcgi?cmd=Retrieve&db=pubmed&dopt=Abstract&list_uids=22357970|6|J Cell Sci|125|Pt 2|Jan|2012|Journal Article,Research Support, Non-U.S. Gov't
egf	ptk2	http://www.ncbi.nlm.nih.gov/entrez/query.fcgi?cmd=Retrieve&db=pubmed&dopt=Abstract&list_uids=23512980|6|Mol Cancer Res|11|6|Jun|2013|Journal Article,Research Support, N.I.H., Extramural,Research Support, U.S. Gov't, Non-P.H.S.|http://www.ncbi.nlm.nih.gov/entrez/query.fcgi?cmd=Retrieve&db=pubmed&dopt=Abstract&list_uids=25530216|6|Cell Signal|27|3|Mar|2015|Journal Article,Research Support, Non-U.S. Gov't|http://www.ncbi.nlm.nih.gov/entrez/query.fcgi?cmd=Retrieve&db=pubmed&dopt=Abstract&list_uids=26079101|6|Biochim Biophys Acta|1853|10 Pt A|Oct|2015|Journal Article,Research Support, Non-U.S. Gov't
egf	rhoa	http://www.ncbi.nlm.nih.gov/entrez/query.fcgi?cmd=Retrieve&db=pubmed&dopt=Abstract&list_uids=26079101|6|Biochim Biophys Acta|1853|10 Pt A|Oct|2015|Journal Article,Research Support, Non-U.S. Gov't
egf	grlf1	http://www.ncbi.nlm.nih.gov/entrez/query.fcgi?cmd=Retrieve&db=pubmed&dopt=Abstract&list_uids=26079101|6|Biochim Biophys Acta|1853|10 Pt A|Oct|2015|Journal Article,Research Support, Non-U.S. Gov't
rhoa	grlf1	http://www.ncbi.nlm.nih.gov/entrez/query.fcgi?cmd=Retrieve&db=pubmed&dopt=Abstract&list_uids=24403595|6|J Virol|88|7|Apr|2014|Journal Article,Research Support, Non-U.S. Gov't|http://www.ncbi.nlm.nih.gov/entrez/query.fcgi?cmd=Retrieve&db=pubmed&dopt=Abstract&list_uids=23707396|6|Exp Cell Res|319|13|Aug|2013|Journal Article|http://www.ncbi.nlm.nih.gov/entrez/query.fcgi?cmd=Retrieve&db=pubmed&dopt=Abstract&list_uids=26079101|6|Biochim Biophys Acta|1853|10 Pt A|Oct|2015|Journal Article,Research Support, Non-U.S. Gov't
ptk2	ilk	http://www.ncbi.nlm.nih.gov/entrez/query.fcgi?cmd=Retrieve&db=pubmed&dopt=Abstract&list_uids=26078334|6|Oncotarget|6|18|Jun|2015|Journal Article,Research Support, Non-U.S. Gov't
ilk	actin	http://www.ncbi.nlm.nih.gov/entrez/query.fcgi?cmd=Retrieve&db=pubmed&dopt=Abstract&list_uids=25482629|6|Cell Adh Migr|9|3||2015|Journal Article,Research Support, U.S. Gov't, Non-P.H.S.|http://www.ncbi.nlm.nih.gov/entrez/query.fcgi?cmd=Retrieve&db=pubmed&dopt=Abstract&list_uids=26078334|6|Oncotarget|6|18|Jun|2015|Journal Article,Research Support, Non-U.S. Gov't
fas	actin	http://www.ncbi.nlm.nih.gov/entrez/query.fcgi?cmd=Retrieve&db=pubmed&dopt=Abstract&list_uids=24501425|6|Mol Biol Cell|25|7|Apr|2014|Journal Article,Research Support, Non-U.S. Gov't|http://www.ncbi.nlm.nih.gov/entrez/query.fcgi?cmd=Retrieve&db=pubmed&dopt=Abstract&list_uids=24452377|6|Am

		J Physiol Cell Physiol|306|6|Mar|2014|Journal Article,Research Support, Non-U.S. Gov't|http://www.ncbi.nlm.nih.gov/entrez/query.fcgi?cmd=Retrieve&db=pubmed&dopt=Abstract&list_uids=22524451|6|Cell Biol Int|36|7|Jul|2012|Journal Article,Research Support, Non-U.S. Gov't|http://www.ncbi.nlm.nih.gov/entrez/query.fcgi?cmd=Retrieve&db=pubmed&dopt=Abstract&list_uids=25488920|6|J Cell Biol|207|5|Dec|2014|Journal Article,Research Support, N.I.H., Extramural,Research Support, Non-U.S. Gov't|http://www.ncbi.nlm.nih.gov/entrez/query.fcgi?cmd=Retrieve&db=pubmed&dopt=Abstract&list_uids=26053221|6|Nat Cell Biol|17|7|Jul|2015|Journal Article,Research Support, N.I.H., Extramural,Research Support, N.I.H., Intramural,Research Support, Non-U.S. Gov't|http://www.ncbi.nlm.nih.gov/entrez/query.fcgi?cmd=Retrieve&db=pubmed&dopt=Abstract&list_uids=23843624|6|J Cell Sci|126|Pt 18|Sep|2013|Journal Article,Research Support, Non-U.S. Gov't|http://www.ncbi.nlm.nih.gov/entrez/query.fcgi?cmd=Retrieve&db=pubmed&dopt=Abstract&list_uids=22496808|6|PLoS One|7|4||2012|Journal Article,Research Support, N.I.H., Extramural,Research Support, Non-U.S. Gov't|http://www.ncbi.nlm.nih.gov/entrez/query.fcgi?cmd=Retrieve&db=pubmed&dopt=Abstract&list_uids=25918420|6|Proc Natl Acad Sci U S A|112|19|May|2015|Journal Article,Research Support, N.I.H., Intramural|http://www.ncbi.nlm.nih.gov/entrez/query.fcgi?cmd=Retrieve&db=pubmed&dopt=Abstract&list_uids=24486488|6|Biochem Biophys Res Commun|444|4|Feb|2014|Journal Article,Research Support, N.I.H., Extramural,Research Support, Non-U.S. Gov't
spp1	actin	http://www.ncbi.nlm.nih.gov/entrez/query.fcgi?cmd=Retrieve&db=pubmed&dopt=Abstract&list_uids=26046934|6|PLoS One|10|6||2015|Journal Article,Research Support, Non-U.S. Gov't
spp1	runx2	http://www.ncbi.nlm.nih.gov/entrez/query.fcgi?cmd=Retrieve&db=pubmed&dopt=Abstract&list_uids=26046934|6|PLoS One|10|6||2015|Journal Article,Research Support, Non-U.S. Gov't
actin	runx2	http://www.ncbi.nlm.nih.gov/entrez/query.fcgi?cmd=Retrieve&db=pubmed&dopt=Abstract&list_uids=26046934|6|PLoS One|10|6||2015|Journal Article,Research Support, Non-U.S. Gov't
spp1	ptk2	http://www.ncbi.nlm.nih.gov/entrez/query.fcgi?cmd=Retrieve&db=pubmed&dopt=Abstract&list_uids=26046934|6|PLoS One|10|6||2015|Journal Article,Research Support, Non-U.S. Gov't
egf	mapk3	http://www.ncbi.nlm.nih.gov/entrez/query.fcgi?cmd=Retrieve&db=pubmed&dopt=Abstract&list_uids=26035355|6|Oncotarget|6|25|Aug|2015|Journal Article,Research Support, Non-U.S. Gov't|http://www.ncbi.nlm.nih.gov/entrez/query.fcgi?cmd=Retrieve&db=pubmed&dopt=Abstract&list_uids=24335959|6|Cancer Res|74|3|Feb|2014|Journal Article,Research Support, Non-U.S. Gov't
hcc	egf	http://www.ncbi.nlm.nih.gov/entrez/query.fcgi?cmd=Retrieve&db=pubmed&dopt=Abstract&list_uids=26035355|6|Oncotarget|6|25|Aug|2015|Journal Article,Research Support, Non-U.S. Gov't
hcc	map2k1	http://www.ncbi.nlm.nih.gov/entrez/query.fcgi?cmd=Retrieve&db=pubmed&dopt=Abstract&list_uids=26035355|6|Oncotarget|6|25|Aug|2015|Journal Article,Research Support, Non-U.S. Gov't
egf	map2k1	http://www.ncbi.nlm.nih.gov/entrez/query.fcgi?cmd=Retrieve&db=pubmed&dopt=Abstract&list_uids=26035355|6|Oncotarget|6|25|Aug|2015|Journal Article,Research Support, Non-U.S. Gov't
yap1	taz	http://www.ncbi.nlm.nih.gov/entrez/query.fcgi?cmd=Retrieve&db=pubmed&dopt=Abstract&list_uids=23969122|6|Differenti

		ation|86|3|Oct|2013|Journal Article,Review|http://www.ncbi.nlm.nih.gov/entrez/query.fcgi?cmd=Retrieve&db=pubmed&dopt=Abstract&list_uids=26026844|6|Biomaterials|61||Aug|2015|Journal Article,Research Support, Non-U.S. Gov't|http://www.ncbi.nlm.nih.gov/entrez/query.fcgi?cmd=Retrieve&db=pubmed&dopt=Abstract&list_uids=22215811|6|Genes Dev|26|1|Jan|2012|Journal Article,Research Support, N.I.H., Extramural,Research Support, U.S. Gov't, Non-P.H.S.|http://www.ncbi.nlm.nih.gov/entrez/query.fcgi?cmd=Retrieve&db=pubmed&dopt=Abstract&list_uids=24106267|6|J Biol Chem|288|47|Nov|2013|Journal Article,Research Support, Non-U.S. Gov't
parp1	mapk8	http://www.ncbi.nlm.nih.gov/entrez/query.fcgi?cmd=Retrieve&db=pubmed&dopt=Abstract&list_uids=26020630|6|PLoS Pathog|11|5|May|2015|Journal Article,Research Support, N.I.H., Extramural,Research Support, Non-U.S. Gov't
parp1	crk	http://www.ncbi.nlm.nih.gov/entrez/query.fcgi?cmd=Retrieve&db=pubmed&dopt=Abstract&list_uids=26020630|6|PLoS Pathog|11|5|May|2015|Journal Article,Research Support, N.I.H., Extramural,Research Support, Non-U.S. Gov't
krit1	a2bp1	http://www.ncbi.nlm.nih.gov/entrez/query.fcgi?cmd=Retrieve&db=pubmed&dopt=Abstract&list_uids=25988664|6|Ann N Y Acad Sci|1349||Sep|2015|Journal Article,Research Support, N.I.H., Intramural,Research Support, Non-U.S. Gov't,Review
akt3	bcl2	http://www.ncbi.nlm.nih.gov/entrez/query.fcgi?cmd=Retrieve&db=pubmed&dopt=Abstract&list_uids=25980492|6|Oncotarget|6|15|May|2015|Journal Article,Research Support, Non-U.S. Gov't
akt3	cyr61	http://www.ncbi.nlm.nih.gov/entrez/query.fcgi?cmd=Retrieve&db=pubmed&dopt=Abstract&list_uids=25980492|6|Oncotarget|6|15|May|2015|Journal Article,Research Support, Non-U.S. Gov't
bcl2	cyr61	http://www.ncbi.nlm.nih.gov/entrez/query.fcgi?cmd=Retrieve&db=pubmed&dopt=Abstract&list_uids=25980492|6|Oncotarget|6|15|May|2015|Journal Article,Research Support, Non-U.S. Gov't
src	bcar1	http://www.ncbi.nlm.nih.gov/entrez/query.fcgi?cmd=Retrieve&db=pubmed&dopt=Abstract&list_uids=25976166|6|J Cell Biochem|116|12|Dec|2015|Journal Article,Research Support, N.I.H., Extramural|http://www.ncbi.nlm.nih.gov/entrez/query.fcgi?cmd=Retrieve&db=pubmed&dopt=Abstract&list_uids=24497348|6|Cell Biol Int|38|3|Mar|2014|Journal Article,Research Support, Non-U.S. Gov't,Review
bcar1	cse1l	http://www.ncbi.nlm.nih.gov/entrez/query.fcgi?cmd=Retrieve&db=pubmed&dopt=Abstract&list_uids=24928898|6|J Cell Sci|127|Pt 16|Aug|2014|Journal Article,Research Support, Non-U.S. Gov't|http://www.ncbi.nlm.nih.gov/entrez/query.fcgi?cmd=Retrieve&db=pubmed&dopt=Abstract&list_uids=23874939|6|PLoS One|8|7||2013|Journal Article,Research Support, Non-U.S. Gov't|http://www.ncbi.nlm.nih.gov/entrez/query.fcgi?cmd=Retrieve&db=pubmed&dopt=Abstract&list_uids=25976166|6|J Cell Biochem|116|12|Dec|2015|Journal Article,Research Support, N.I.H., Extramural
cse1l	src	http://www.ncbi.nlm.nih.gov/entrez/query.fcgi?cmd=Retrieve&db=pubmed&dopt=Abstract&list_uids=22287273|6|J Cell Physiol|227|11|Nov|2012|Journal Article,Research Support, N.I.H., Extramural|http://www.ncbi.nlm.nih.gov/entrez/query.fcgi?cmd=Retrieve&db=pubmed&dopt=Abstract&list_uids=24928898|6|J Cell Sci|127|Pt 16|Aug|2014|Journal Article,Research Support, Non-U.S. Gov't|http://www.ncbi.nlm.nih.gov/entrez/query.fcgi?cmd=Retrieve&db=pubmed&dopt=Abstract&list_uids=25976166|6|J Cell Biochem|116|12|Dec|2015|Journal Article,Research Support, N.I.H., Extramural
cav1	lgals3	http://www.ncbi.nlm.nih.gov/entrez/query.fcgi?cmd=Retrieve&db=pubmed&dopt=Abstract&list_uids=22513979|6|J

		Pathol|228|1|Sep|2012|Journal Article,Research Support, Non-U.S. Gov't|http://www.ncbi.nlm.nih.gov/entrez/query.fcgi?cmd=Retrieve&db=pubmed&dopt=Abstract&list_uids=25942420|6|PLoS One|10|5||2015|Journal Article,Research Support, Non-U.S. Gov't
cav1	ptk2	http://www.ncbi.nlm.nih.gov/entrez/query.fcgi?cmd=Retrieve&db=pubmed&dopt=Abstract&list_uids=22513979|6|J Pathol|228|1|Sep|2012|Journal Article,Research Support, Non-U.S. Gov't|http://www.ncbi.nlm.nih.gov/entrez/query.fcgi?cmd=Retrieve&db=pubmed&dopt=Abstract&list_uids=25942420|6|PLoS One|10|5||2015|Journal Article,Research Support, Non-U.S. Gov't
cav1	fas	http://www.ncbi.nlm.nih.gov/entrez/query.fcgi?cmd=Retrieve&db=pubmed&dopt=Abstract&list_uids=25942420|6|PLoS One|10|5||2015|Journal Article,Research Support, Non-U.S. Gov't
lgals3	ptk2	http://www.ncbi.nlm.nih.gov/entrez/query.fcgi?cmd=Retrieve&db=pubmed&dopt=Abstract&list_uids=22513979|6|J Pathol|228|1|Sep|2012|Journal Article,Research Support, Non-U.S. Gov't|http://www.ncbi.nlm.nih.gov/entrez/query.fcgi?cmd=Retrieve&db=pubmed&dopt=Abstract&list_uids=25942420|6|PLoS One|10|5||2015|Journal Article,Research Support, Non-U.S. Gov't
lgals3	fas	http://www.ncbi.nlm.nih.gov/entrez/query.fcgi?cmd=Retrieve&db=pubmed&dopt=Abstract&list_uids=25942420|6|PLoS One|10|5||2015|Journal Article,Research Support, Non-U.S. Gov't
inf2	actin	http://www.ncbi.nlm.nih.gov/entrez/query.fcgi?cmd=Retrieve&db=pubmed&dopt=Abstract&list_uids=25918420|6|Proc Natl Acad Sci U S A|112|19|May|2015|Journal Article,Research Support, N.I.H., Intramural
inf2	fas	http://www.ncbi.nlm.nih.gov/entrez/query.fcgi?cmd=Retrieve&db=pubmed&dopt=Abstract&list_uids=25918420|6|Proc Natl Acad Sci U S A|112|19|May|2015|Journal Article,Research Support, N.I.H., Intramural
fas	mmrn1	http://www.ncbi.nlm.nih.gov/entrez/query.fcgi?cmd=Retrieve&db=pubmed&dopt=Abstract&list_uids=25544611|6|Curr Biol|25|2|Jan|2015|Journal Article,Research Support, N.I.H., Extramural,Research Support, N.I.H., Intramural|http://www.ncbi.nlm.nih.gov/entrez/query.fcgi?cmd=Retrieve&db=pubmed&dopt=Abstract&list_uids=25081612|6|Prog Mol Biol Transl Sci|126|||2014|Journal Article,Research Support, Non-U.S. Gov't,Review|http://www.ncbi.nlm.nih.gov/entrez/query.fcgi?cmd=Retrieve&db=pubmed&dopt=Abstract&list_uids=23830911|6|Curr Opin Cell Biol|25|5|Oct|2013|Journal Article,Research Support, N.I.H., Intramural,Review|http://www.ncbi.nlm.nih.gov/entrez/query.fcgi?cmd=Retrieve&db=pubmed&dopt=Abstract&list_uids=23754369|6|Proc Natl Acad Sci U S A|110|26|Jun|2013|Journal Article,Research Support, N.I.H., Intramural,Research Support, Non-U.S. Gov't|http://www.ncbi.nlm.nih.gov/entrez/query.fcgi?cmd=Retrieve&db=pubmed&dopt=Abstract&list_uids=25918420|6|Proc Natl Acad Sci U S A|112|19|May|2015|Journal Article,Research Support, N.I.H., Intramural|http://www.ncbi.nlm.nih.gov/entrez/query.fcgi?cmd=Retrieve&db=pubmed&dopt=Abstract&list_uids=22908306|6|J Cell Biol|198|4|Aug|2012|Journal Article,Research Support, N.I.H., Extramural,Research Support, Non-U.S. Gov't,Review
synpo2	actin	http://www.ncbi.nlm.nih.gov/entrez/query.fcgi?cmd=Retrieve&db=pubmed&dopt=Abstract&list_uids=25883213|6|Oncotarget|6|13|May|2015|Journal Article,Research Support, Non-U.S. Gov't
synpo2	pcsk1	http://www.ncbi.nlm.nih.gov/entrez/query.fcgi?cmd=Retrieve&db=pubmed&dopt=Abstract&list_uids=25883213|6|Oncotarget|6|13|May|2015|Journal Article,Research Support, Non-U.S. Gov't

actin	pcsk1	http://www.ncbi.nlm.nih.gov/entrez/query.fcgi?cmd=Retrieve&db=pubmed&dopt=Abstract&list_uids=25883213|6|Oncotarget|6|13|May|2015|Journal Article,Research Support, Non-U.S. Gov't
myh14	synpo2	http://www.ncbi.nlm.nih.gov/entrez/query.fcgi?cmd=Retrieve&db=pubmed&dopt=Abstract&list_uids=25883213|6|Oncotarget|6|13|May|2015|Journal Article,Research Support, Non-U.S. Gov't
ddr1	atm	http://www.ncbi.nlm.nih.gov/entrez/query.fcgi?cmd=Retrieve&db=pubmed&dopt=Abstract&list_uids=25874996|6|PLoS One|10|4||2015|Journal Article,Research Support, Non-U.S. Gov't
atm	actin	http://www.ncbi.nlm.nih.gov/entrez/query.fcgi?cmd=Retrieve&db=pubmed&dopt=Abstract&list_uids=25874996|6|PLoS One|10|4||2015|Journal Article,Research Support, Non-U.S. Gov't
atm	net1	http://www.ncbi.nlm.nih.gov/entrez/query.fcgi?cmd=Retrieve&db=pubmed&dopt=Abstract&list_uids=25874996|6|PLoS One|10|4||2015|Journal Article,Research Support, Non-U.S. Gov't
actin	net1	http://www.ncbi.nlm.nih.gov/entrez/query.fcgi?cmd=Retrieve&db=pubmed&dopt=Abstract&list_uids=25874996|6|PLoS One|10|4||2015|Journal Article,Research Support, Non-U.S. Gov't
pdcd6ip	tsg101	http://www.ncbi.nlm.nih.gov/entrez/query.fcgi?cmd=Retrieve&db=pubmed&dopt=Abstract&list_uids=25874996|6|PLoS One|10|4||2015|Journal Article,Research Support, Non-U.S. Gov't
fer	src	http://www.ncbi.nlm.nih.gov/entrez/query.fcgi?cmd=Retrieve&db=pubmed&dopt=Abstract&list_uids=25867068|6|Oncogene|35|4|Jan|2016|Journal Article,Research Support, Non-U.S. Gov't
anxa1	mapk3	http://www.ncbi.nlm.nih.gov/entrez/query.fcgi?cmd=Retrieve&db=pubmed&dopt=Abstract&list_uids=25866182|6|Biochem Biophys Res Commun|461|1|May|2015|Journal Article,Research Support, Non-U.S. Gov't
anxa1	rhoa	http://www.ncbi.nlm.nih.gov/entrez/query.fcgi?cmd=Retrieve&db=pubmed&dopt=Abstract&list_uids=25866182|6|Biochem Biophys Res Commun|461|1|May|2015|Journal Article,Research Support, Non-U.S. Gov't
anxa1	ephb2	http://www.ncbi.nlm.nih.gov/entrez/query.fcgi?cmd=Retrieve&db=pubmed&dopt=Abstract&list_uids=25866182|6|Biochem Biophys Res Commun|461|1|May|2015|Journal Article,Research Support, Non-U.S. Gov't
rhoa	ephb2	http://www.ncbi.nlm.nih.gov/entrez/query.fcgi?cmd=Retrieve&db=pubmed&dopt=Abstract&list_uids=25866182|6|Biochem Biophys Res Commun|461|1|May|2015|Journal Article,Research Support, Non-U.S. Gov't
rhoa	cxcl12	http://www.ncbi.nlm.nih.gov/entrez/query.fcgi?cmd=Retrieve&db=pubmed&dopt=Abstract&list_uids=25866182|6|Biochem Biophys Res Commun|461|1|May|2015|Journal Article,Research Support, Non-U.S. Gov't
rhoa	map3k1	http://www.ncbi.nlm.nih.gov/entrez/query.fcgi?cmd=Retrieve&db=pubmed&dopt=Abstract&list_uids=25866182|6|Biochem Biophys Res Commun|461|1|May|2015|Journal Article,Research Support, Non-U.S. Gov't
cxcl12	map3k1	http://www.ncbi.nlm.nih.gov/entrez/query.fcgi?cmd=Retrieve&db=pubmed&dopt=Abstract&list_uids=25866182|6|Biochem Biophys Res Commun|461|1|May|2015|Journal Article,Research Support, Non-U.S. Gov't
mapk3	rhoa	http://www.ncbi.nlm.nih.gov/entrez/query.fcgi?cmd=Retrieve&db=pubmed&dopt=Abstract&list_uids=22946047|6|J Cell Sci|125|Pt 22|Nov|2012|Journal Article,Research Support, Non-U.S. Gov't|http://www.ncbi.nlm.nih.gov/entrez/query.fcgi?cmd=Retrieve&db=pubmed&dopt=Abstract&list_uids=25866182|6|Biochem Biophys Res Commun|461|1|May|2015|Journal Article,Research Support, Non-U.S. Gov't
tys	s1pr1	http://www.ncbi.nlm.nih.gov/entrez/query.fcgi?cmd=Retrieve&db=pubmed&dopt=Abstract&list_uids=25862132|6|Microvasc Res|99||May|2015|Journal Article,Research Support, N.I.H., Extramural

tys	mbtps1	http://www.ncbi.nlm.nih.gov/entrez/query.fcgi?cmd=Retrieve&db=pubmed&dopt=Abstract&list_uids=25862132|6|Microvasc Res|99||May|2015|Journal Article,Research Support, N.I.H., Extramural
s1pr1	mbtps1	http://www.ncbi.nlm.nih.gov/entrez/query.fcgi?cmd=Retrieve&db=pubmed&dopt=Abstract&list_uids=25862132|6|Microvasc Res|99||May|2015|Journal Article,Research Support, N.I.H., Extramural
tys	tjp1	http://www.ncbi.nlm.nih.gov/entrez/query.fcgi?cmd=Retrieve&db=pubmed&dopt=Abstract&list_uids=25862132|6|Microvasc Res|99||May|2015|Journal Article,Research Support, N.I.H., Extramural
tys	gpsn2	http://www.ncbi.nlm.nih.gov/entrez/query.fcgi?cmd=Retrieve&db=pubmed&dopt=Abstract&list_uids=25862132|6|Microvasc Res|99||May|2015|Journal Article,Research Support, N.I.H., Extramural
gpsn2	tjp1	http://www.ncbi.nlm.nih.gov/entrez/query.fcgi?cmd=Retrieve&db=pubmed&dopt=Abstract&list_uids=25862132|6|Microvasc Res|99||May|2015|Journal Article,Research Support, N.I.H., Extramural
tys	ptk2	http://www.ncbi.nlm.nih.gov/entrez/query.fcgi?cmd=Retrieve&db=pubmed&dopt=Abstract&list_uids=25862132|6|Microvasc Res|99||May|2015|Journal Article,Research Support, N.I.H., Extramural
ptk2	gpsn2	http://www.ncbi.nlm.nih.gov/entrez/query.fcgi?cmd=Retrieve&db=pubmed&dopt=Abstract&list_uids=25862132|6|Microvasc Res|99||May|2015|Journal Article,Research Support, N.I.H., Extramural
tys	actin	http://www.ncbi.nlm.nih.gov/entrez/query.fcgi?cmd=Retrieve&db=pubmed&dopt=Abstract&list_uids=25862132|6|Microvasc Res|99||May|2015|Journal Article,Research Support, N.I.H., Extramural
actin	gpsn2	http://www.ncbi.nlm.nih.gov/entrez/query.fcgi?cmd=Retrieve&db=pubmed&dopt=Abstract&list_uids=25862132|6|Microvasc Res|99||May|2015|Journal Article,Research Support, N.I.H., Extramural
tys	akt1	http://www.ncbi.nlm.nih.gov/entrez/query.fcgi?cmd=Retrieve&db=pubmed&dopt=Abstract&list_uids=25862132|6|Microvasc Res|99||May|2015|Journal Article,Research Support, N.I.H., Extramural
tys	pi3	http://www.ncbi.nlm.nih.gov/entrez/query.fcgi?cmd=Retrieve&db=pubmed&dopt=Abstract&list_uids=25862132|6|Microvasc Res|99||May|2015|Journal Article,Research Support, N.I.H., Extramural
akt1	pi3	http://www.ncbi.nlm.nih.gov/entrez/query.fcgi?cmd=Retrieve&db=pubmed&dopt=Abstract&list_uids=25862132|6|Microvasc Res|99||May|2015|Journal Article,Research Support, N.I.H., Extramural|http://www.ncbi.nlm.nih.gov/entrez/query.fcgi?cmd=Retrieve&db=pubmed&dopt=Abstract&list_uids=25000176|6|PLoS One|9|7||2014|Journal Article,Research Support, N.I.H., Extramural,Research Support, U.S. Gov't, Non-P.H.S.
akt1	gpsn2	http://www.ncbi.nlm.nih.gov/entrez/query.fcgi?cmd=Retrieve&db=pubmed&dopt=Abstract&list_uids=25862132|6|Microvasc Res|99||May|2015|Journal Article,Research Support, N.I.H., Extramural
pi3	gpsn2	http://www.ncbi.nlm.nih.gov/entrez/query.fcgi?cmd=Retrieve&db=pubmed&dopt=Abstract&list_uids=25862132|6|Microvasc Res|99||May|2015|Journal Article,Research Support, N.I.H., Extramural
tys	rac1	http://www.ncbi.nlm.nih.gov/entrez/query.fcgi?cmd=Retrieve&db=pubmed&dopt=Abstract&list_uids=25862132|6|Microvasc Res|99||May|2015|Journal Article,Research Support, N.I.H., Extramural
rac1	gpsn2	http://www.ncbi.nlm.nih.gov/entrez/query.fcgi?cmd=Retrieve&db=pubmed&dopt=Abstract&list_uids=25862132|6|Microvasc Res|99||May|2015|Journal Article,Research Support, N.I.H., Extramural
actn1	zyx	http://www.ncbi.nlm.nih.gov/entrez/query.fcgi?cmd=Retrieve&db=pubmed&dopt=Abstract&list_uids=25860875|6|PLoS One|10|4||2015|Journal Article

actn1	actn4	http://www.ncbi.nlm.nih.gov/entrez/query.fcgi?cmd=Retrieve&db=pubmed&dopt=Abstract&list_uids=25860875|6|PLoS One|10|4||2015|Journal Article
zyx	actn4	http://www.ncbi.nlm.nih.gov/entrez/query.fcgi?cmd=Retrieve&db=pubmed&dopt=Abstract&list_uids=25860875|6|PLoS One|10|4||2015|Journal Article
vegfa	fas	http://www.ncbi.nlm.nih.gov/entrez/query.fcgi?cmd=Retrieve&db=pubmed&dopt=Abstract&list_uids=25820249|6|J Cell Physiol|230|11|Nov|2015|Journal Article,Research Support, Non-U.S. Gov't
rac1	vegfa	http://www.ncbi.nlm.nih.gov/entrez/query.fcgi?cmd=Retrieve&db=pubmed&dopt=Abstract&list_uids=25820249|6|J Cell Physiol|230|11|Nov|2015|Journal Article,Research Support, Non-U.S. Gov't|http://www.ncbi.nlm.nih.gov/entrez/query.fcgi?cmd=Retrieve&db=pubmed&dopt=Abstract&list_uids=24903274|6|FASEB J|28|9|Sep|2014|Journal Article,Research Support, Non-U.S. Gov't
tgfb1i1	nox4	http://www.ncbi.nlm.nih.gov/entrez/query.fcgi?cmd=Retrieve&db=pubmed&dopt=Abstract&list_uids=25814672|6|Arterioscler Thromb Vasc Biol|35|5|May|2015|Journal Article,Research Support, N.I.H., Extramural,Research Support, Non-U.S. Gov't
mdfic	hsp90b2p	http://www.ncbi.nlm.nih.gov/entrez/query.fcgi?cmd=Retrieve&db=pubmed&dopt=Abstract&list_uids=25814672|6|Arterioscler Thromb Vasc Biol|35|5|May|2015|Journal Article,Research Support, N.I.H., Extramural,Research Support, Non-U.S. Gov't
tgfb1i1	hspb1	http://www.ncbi.nlm.nih.gov/entrez/query.fcgi?cmd=Retrieve&db=pubmed&dopt=Abstract&list_uids=25814672|6|Arterioscler Thromb Vasc Biol|35|5|May|2015|Journal Article,Research Support, N.I.H., Extramural,Research Support, Non-U.S. Gov't
tgfb1i1	fas	http://www.ncbi.nlm.nih.gov/entrez/query.fcgi?cmd=Retrieve&db=pubmed&dopt=Abstract&list_uids=25814672|6|Arterioscler Thromb Vasc Biol|35|5|May|2015|Journal Article,Research Support, N.I.H., Extramural,Research Support, Non-U.S. Gov't
hspb1	nox4	http://www.ncbi.nlm.nih.gov/entrez/query.fcgi?cmd=Retrieve&db=pubmed&dopt=Abstract&list_uids=25814672|6|Arterioscler Thromb Vasc Biol|35|5|May|2015|Journal Article,Research Support, N.I.H., Extramural,Research Support, Non-U.S. Gov't
hspb1	fas	http://www.ncbi.nlm.nih.gov/entrez/query.fcgi?cmd=Retrieve&db=pubmed&dopt=Abstract&list_uids=25814672|6|Arterioscler Thromb Vasc Biol|35|5|May|2015|Journal Article,Research Support, N.I.H., Extramural,Research Support, Non-U.S. Gov't
nox4	fas	http://www.ncbi.nlm.nih.gov/entrez/query.fcgi?cmd=Retrieve&db=pubmed&dopt=Abstract&list_uids=25814672|6|Arterioscler Thromb Vasc Biol|35|5|May|2015|Journal Article,Research Support, N.I.H., Extramural,Research Support, Non-U.S. Gov't
itgb3bp	map3k8	http://www.ncbi.nlm.nih.gov/entrez/query.fcgi?cmd=Retrieve&db=pubmed&dopt=Abstract&list_uids=25774502|6|J Clin Invest|125|4|Apr|2015|Journal Article,Research Support, Non-U.S. Gov't,Video-Audio Media
itgb3bp	nek2	http://www.ncbi.nlm.nih.gov/entrez/query.fcgi?cmd=Retrieve&db=pubmed&dopt=Abstract&list_uids=25774502|6|J Clin Invest|125|4|Apr|2015|Journal Article,Research Support, Non-U.S. Gov't,Video-Audio Media
itgb3bp	shc1	http://www.ncbi.nlm.nih.gov/entrez/query.fcgi?cmd=Retrieve&db=pubmed&dopt=Abstract&list_uids=25774502|6|J Clin

		Invest|125|4|Apr|2015|Journal Article,Research Support, Non-U.S. Gov't,Video-Audio Media
map3k8	nek2	http://www.ncbi.nlm.nih.gov/entrez/query.fcgi?cmd=Retrieve&db=pubmed&dopt=Abstract&list_uids=25774502|6|J Clin Invest|125|4|Apr|2015|Journal Article,Research Support, Non-U.S. Gov't,Video-Audio Media
map3k8	shc1	http://www.ncbi.nlm.nih.gov/entrez/query.fcgi?cmd=Retrieve&db=pubmed&dopt=Abstract&list_uids=25774502|6|J Clin Invest|125|4|Apr|2015|Journal Article,Research Support, Non-U.S. Gov't,Video-Audio Media
nek2	shc1	http://www.ncbi.nlm.nih.gov/entrez/query.fcgi?cmd=Retrieve&db=pubmed&dopt=Abstract&list_uids=25774502|6|J Clin Invest|125|4|Apr|2015|Journal Article,Research Support, Non-U.S. Gov't,Video-Audio Media
src	ctgf	http://www.ncbi.nlm.nih.gov/entrez/query.fcgi?cmd=Retrieve&db=pubmed&dopt=Abstract&list_uids=25766369|6|J Cell Mol Med|19|6|Jun|2015|Journal Article,Research Support, Non-U.S. Gov't
ptk2	ctgf	http://www.ncbi.nlm.nih.gov/entrez/query.fcgi?cmd=Retrieve&db=pubmed&dopt=Abstract&list_uids=23280549|6|J Cell Biochem|114|6|Jun|2013|Journal Article,Research Support, Non-U.S. Gov't|http://www.ncbi.nlm.nih.gov/entrez/query.fcgi?cmd=Retrieve&db=pubmed&dopt=Abstract&list_uids=25766369|6|J Cell Mol Med|19|6|Jun|2015|Journal Article,Research Support, Non-U.S. Gov't
ptk2	sfrs2	http://www.ncbi.nlm.nih.gov/entrez/query.fcgi?cmd=Retrieve&db=pubmed&dopt=Abstract&list_uids=25765854|6|Integr Biol (Camb)|7|4|Apr|2015|Journal Article,Research Support, N.I.H., Extramural,Research Support, Non-U.S. Gov't
hrc	satb1	http://www.ncbi.nlm.nih.gov/entrez/query.fcgi?cmd=Retrieve&db=pubmed&dopt=Abstract&list_uids=25762622|6|Oncotarget|6|9|Mar|2015|Journal Article,Research Support, Non-U.S. Gov't
hrc	ptk2	http://www.ncbi.nlm.nih.gov/entrez/query.fcgi?cmd=Retrieve&db=pubmed&dopt=Abstract&list_uids=25762622|6|Oncotarget|6|9|Mar|2015|Journal Article,Research Support, Non-U.S. Gov't
hrc	atp2a2	http://www.ncbi.nlm.nih.gov/entrez/query.fcgi?cmd=Retrieve&db=pubmed&dopt=Abstract&list_uids=25762622|6|Oncotarget|6|9|Mar|2015|Journal Article,Research Support, Non-U.S. Gov't
hrc	hcc	http://www.ncbi.nlm.nih.gov/entrez/query.fcgi?cmd=Retrieve&db=pubmed&dopt=Abstract&list_uids=25762622|6|Oncotarget|6|9|Mar|2015|Journal Article,Research Support, Non-U.S. Gov't
hcc	satb1	http://www.ncbi.nlm.nih.gov/entrez/query.fcgi?cmd=Retrieve&db=pubmed&dopt=Abstract&list_uids=25762622|6|Oncotarget|6|9|Mar|2015|Journal Article,Research Support, Non-U.S. Gov't
satb1	fos	http://www.ncbi.nlm.nih.gov/entrez/query.fcgi?cmd=Retrieve&db=pubmed&dopt=Abstract&list_uids=25762622|6|Oncotarget|6|9|Mar|2015|Journal Article,Research Support, Non-U.S. Gov't
satb1	mapk8	http://www.ncbi.nlm.nih.gov/entrez/query.fcgi?cmd=Retrieve&db=pubmed&dopt=Abstract&list_uids=25762622|6|Oncotarget|6|9|Mar|2015|Journal Article,Research Support, Non-U.S. Gov't
hrc	fos	http://www.ncbi.nlm.nih.gov/entrez/query.fcgi?cmd=Retrieve&db=pubmed&dopt=Abstract&list_uids=25762622|6|Oncotarget|6|9|Mar|2015|Journal Article,Research Support, Non-U.S. Gov't
hrc	mapk8	http://www.ncbi.nlm.nih.gov/entrez/query.fcgi?cmd=Retrieve&db=pubmed&dopt=Abstract&list_uids=25762622|6|Oncotarget|6|9|Mar|2015|Journal Article,Research Support, Non-U.S. Gov't
fos	mapk8	http://www.ncbi.nlm.nih.gov/entrez/query.fcgi?cmd=Retrieve&db=pubmed&dopt=Abstract&list_uids=23399841|6|Cell Immunol|280|2|Dec|2012|Journal Article,Research Support, N.I.H., Extramural,Research Support, Non-U.S. Gov't|http://www.ncbi.nlm.nih.gov/entrez/query.fcgi?cmd=Retrieve&db=pubmed&dopt=Abstract&list_uids=25762622|6|On

		cotarget|6|9|Mar|2015|Journal Article,Research Support, Non-U.S. Gov't
gnb2l1	igf1	http://www.ncbi.nlm.nih.gov/entrez/query.fcgi?cmd=Retrieve&db=pubmed&dopt=Abstract&list_uids=25694432|6|J Biol Chem|290|15|Apr|2015|Journal Article,Research Support, Non-U.S. Gov't
gnb2l1	abl1	http://www.ncbi.nlm.nih.gov/entrez/query.fcgi?cmd=Retrieve&db=pubmed&dopt=Abstract&list_uids=25694432|6|J Biol Chem|290|15|Apr|2015|Journal Article,Research Support, Non-U.S. Gov't
igf1	abl1	http://www.ncbi.nlm.nih.gov/entrez/query.fcgi?cmd=Retrieve&db=pubmed&dopt=Abstract&list_uids=25694432|6|J Biol Chem|290|15|Apr|2015|Journal Article,Research Support, Non-U.S. Gov't
igf1	src	http://www.ncbi.nlm.nih.gov/entrez/query.fcgi?cmd=Retrieve&db=pubmed&dopt=Abstract&list_uids=23023326|6|Int J Oncol|41|6|Dec|2012|Journal Article,Research Support, Non-U.S. Gov't|http://www.ncbi.nlm.nih.gov/entrez/query.fcgi?cmd=Retrieve&db=pubmed&dopt=Abstract&list_uids=25694432|6|J Biol Chem|290|15|Apr|2015|Journal Article,Research Support, Non-U.S. Gov't
src	abl1	http://www.ncbi.nlm.nih.gov/entrez/query.fcgi?cmd=Retrieve&db=pubmed&dopt=Abstract&list_uids=25694432|6|J Biol Chem|290|15|Apr|2015|Journal Article,Research Support, Non-U.S. Gov't
fyn	igf1	http://www.ncbi.nlm.nih.gov/entrez/query.fcgi?cmd=Retrieve&db=pubmed&dopt=Abstract&list_uids=25694432|6|J Biol Chem|290|15|Apr|2015|Journal Article,Research Support, Non-U.S. Gov't
fyn	abl1	http://www.ncbi.nlm.nih.gov/entrez/query.fcgi?cmd=Retrieve&db=pubmed&dopt=Abstract&list_uids=25694432|6|J Biol Chem|290|15|Apr|2015|Journal Article,Research Support, Non-U.S. Gov't
cul3	kbtbd6	http://www.ncbi.nlm.nih.gov/entrez/query.fcgi?cmd=Retrieve&db=pubmed&dopt=Abstract&list_uids=25684205|6|Mol Cell|57|6|Mar|2015|Journal Article,Research Support, Non-U.S. Gov't
cul3	kbtbd7	http://www.ncbi.nlm.nih.gov/entrez/query.fcgi?cmd=Retrieve&db=pubmed&dopt=Abstract&list_uids=25684205|6|Mol Cell|57|6|Mar|2015|Journal Article,Research Support, Non-U.S. Gov't
cul3	tiam1	http://www.ncbi.nlm.nih.gov/entrez/query.fcgi?cmd=Retrieve&db=pubmed&dopt=Abstract&list_uids=25684205|6|Mol Cell|57|6|Mar|2015|Journal Article,Research Support, Non-U.S. Gov't
cul3	rac1	http://www.ncbi.nlm.nih.gov/entrez/query.fcgi?cmd=Retrieve&db=pubmed&dopt=Abstract&list_uids=25684205|6|Mol Cell|57|6|Mar|2015|Journal Article,Research Support, Non-U.S. Gov't
cul3	arhgef2	http://www.ncbi.nlm.nih.gov/entrez/query.fcgi?cmd=Retrieve&db=pubmed&dopt=Abstract&list_uids=25684205|6|Mol Cell|57|6|Mar|2015|Journal Article,Research Support, Non-U.S. Gov't
kbtbd6	kbtbd7	http://www.ncbi.nlm.nih.gov/entrez/query.fcgi?cmd=Retrieve&db=pubmed&dopt=Abstract&list_uids=25684205|6|Mol Cell|57|6|Mar|2015|Journal Article,Research Support, Non-U.S. Gov't
kbtbd6	tiam1	http://www.ncbi.nlm.nih.gov/entrez/query.fcgi?cmd=Retrieve&db=pubmed&dopt=Abstract&list_uids=25684205|6|Mol Cell|57|6|Mar|2015|Journal Article,Research Support, Non-U.S. Gov't
kbtbd6	rac1	http://www.ncbi.nlm.nih.gov/entrez/query.fcgi?cmd=Retrieve&db=pubmed&dopt=Abstract&list_uids=25684205|6|Mol Cell|57|6|Mar|2015|Journal Article,Research Support, Non-U.S. Gov't
kbtbd6	arhgef2	http://www.ncbi.nlm.nih.gov/entrez/query.fcgi?cmd=Retrieve&db=pubmed&dopt=Abstract&list_uids=25684205|6|Mol Cell|57|6|Mar|2015|Journal Article,Research Support, Non-U.S. Gov't

kbtbd7	tiam1	http://www.ncbi.nlm.nih.gov/entrez/query.fcgi?cmd=Retrieve&db=pubmed&dopt=Abstract&list_uids=25684205|6|Mol Cell|57|6|Mar|2015|Journal Article,Research Support, Non-U.S. Gov't
kbtbd7	rac1	http://www.ncbi.nlm.nih.gov/entrez/query.fcgi?cmd=Retrieve&db=pubmed&dopt=Abstract&list_uids=25684205|6|Mol Cell|57|6|Mar|2015|Journal Article,Research Support, Non-U.S. Gov't
kbtbd7	arhgef2	http://www.ncbi.nlm.nih.gov/entrez/query.fcgi?cmd=Retrieve&db=pubmed&dopt=Abstract&list_uids=25684205|6|Mol Cell|57|6|Mar|2015|Journal Article,Research Support, Non-U.S. Gov't
tiam1	rac1	http://www.ncbi.nlm.nih.gov/entrez/query.fcgi?cmd=Retrieve&db=pubmed&dopt=Abstract&list_uids=25684205|6|Mol Cell|57|6|Mar|2015|Journal Article,Research Support, Non-U.S. Gov't
tiam1	arhgef2	http://www.ncbi.nlm.nih.gov/entrez/query.fcgi?cmd=Retrieve&db=pubmed&dopt=Abstract&list_uids=25684205|6|Mol Cell|57|6|Mar|2015|Journal Article,Research Support, Non-U.S. Gov't
rac1	arhgef2	http://www.ncbi.nlm.nih.gov/entrez/query.fcgi?cmd=Retrieve&db=pubmed&dopt=Abstract&list_uids=23615439|6|Mol Biol Cell|24|12|Jun|2013|Journal Article,Research Support, N.I.H., Extramural|http://www.ncbi.nlm.nih.gov/entrez/query.fcgi?cmd=Retrieve&db=pubmed&dopt=Abstract&list_uids=25684205|6|Mol Cell|57|6|Mar|2015|Journal Article,Research Support, Non-U.S. Gov't
kbtbd6	gabarapl2	http://www.ncbi.nlm.nih.gov/entrez/query.fcgi?cmd=Retrieve&db=pubmed&dopt=Abstract&list_uids=25684205|6|Mol Cell|57|6|Mar|2015|Journal Article,Research Support, Non-U.S. Gov't
kbtbd6	gabarap	http://www.ncbi.nlm.nih.gov/entrez/query.fcgi?cmd=Retrieve&db=pubmed&dopt=Abstract&list_uids=25684205|6|Mol Cell|57|6|Mar|2015|Journal Article,Research Support, Non-U.S. Gov't
kbtbd7	gabarapl2	http://www.ncbi.nlm.nih.gov/entrez/query.fcgi?cmd=Retrieve&db=pubmed&dopt=Abstract&list_uids=25684205|6|Mol Cell|57|6|Mar|2015|Journal Article,Research Support, Non-U.S. Gov't
kbtbd7	gabarap	http://www.ncbi.nlm.nih.gov/entrez/query.fcgi?cmd=Retrieve&db=pubmed&dopt=Abstract&list_uids=25684205|6|Mol Cell|57|6|Mar|2015|Journal Article,Research Support, Non-U.S. Gov't
gabarapl2	gabarap	http://www.ncbi.nlm.nih.gov/entrez/query.fcgi?cmd=Retrieve&db=pubmed&dopt=Abstract&list_uids=25684205|6|Mol Cell|57|6|Mar|2015|Journal Article,Research Support, Non-U.S. Gov't
tiam1	gabarap	http://www.ncbi.nlm.nih.gov/entrez/query.fcgi?cmd=Retrieve&db=pubmed&dopt=Abstract&list_uids=25684205|6|Mol Cell|57|6|Mar|2015|Journal Article,Research Support, Non-U.S. Gov't
stard3	erbb2	http://www.ncbi.nlm.nih.gov/entrez/query.fcgi?cmd=Retrieve&db=pubmed&dopt=Abstract&list_uids=25681734|6|Am J Pathol|185|4|Apr|2015|Journal Article,Research Support, Non-U.S. Gov't
ca4	actin	http://www.ncbi.nlm.nih.gov/entrez/query.fcgi?cmd=Retrieve&db=pubmed&dopt=Abstract&list_uids=25678082|6|Invest New Drugs|33|3|Jun|2015|Journal Article
ilk	xrcc1	http://www.ncbi.nlm.nih.gov/entrez/query.fcgi?cmd=Retrieve&db=pubmed&dopt=Abstract&list_uids=25657336|6|Mol Cancer Ther|14|4|Apr|2015|Journal Article,Research Support, N.I.H., Extramural
dclk1	itk	http://www.ncbi.nlm.nih.gov/entrez/query.fcgi?cmd=Retrieve&db=pubmed&dopt=Abstract&list_uids=25605241|6|Oncotarget|6|4|Feb|2015|Journal Article
actin	gli2	http://www.ncbi.nlm.nih.gov/entrez/query.fcgi?cmd=Retrieve&db=pubmed&dopt=Abstract&list_uids=25601713|6|Biochim Biophys Acta|1853|5|May|2015|Journal Article,Research Support, Non-U.S. Gov't

flna	mmrn1	http://www.ncbi.nlm.nih.gov/entrez/query.fcgi?cmd=Retrieve&db=pubmed&dopt=Abstract&list_uids=25593085|6|Biochimie|110||Mar|2015|Journal Article,Research Support, Non-U.S. Gov't
flna	ptk2	http://www.ncbi.nlm.nih.gov/entrez/query.fcgi?cmd=Retrieve&db=pubmed&dopt=Abstract&list_uids=25593085|6|Biochimie|110||Mar|2015|Journal Article,Research Support, Non-U.S. Gov't
flna	p4hb	http://www.ncbi.nlm.nih.gov/entrez/query.fcgi?cmd=Retrieve&db=pubmed&dopt=Abstract&list_uids=25593085|6|Biochimie|110||Mar|2015|Journal Article,Research Support, Non-U.S. Gov't
pdk1	pik3ca	http://www.ncbi.nlm.nih.gov/entrez/query.fcgi?cmd=Retrieve&db=pubmed&dopt=Abstract&list_uids=25588838|6|J Cell Sci|128|5|Mar|2015|Journal Article,Research Support, Non-U.S. Gov't
pdk1	akt1	http://www.ncbi.nlm.nih.gov/entrez/query.fcgi?cmd=Retrieve&db=pubmed&dopt=Abstract&list_uids=25588838|6|J Cell Sci|128|5|Mar|2015|Journal Article,Research Support, Non-U.S. Gov't
foxo1	irf4	http://www.ncbi.nlm.nih.gov/entrez/query.fcgi?cmd=Retrieve&db=pubmed&dopt=Abstract&list_uids=25584414|6|Mol Endocrinol|29|3|Mar|2015|Journal Article,Research Support, N.I.H., Extramural,Research Support, Non-U.S. Gov't
irf4	igfbp1	http://www.ncbi.nlm.nih.gov/entrez/query.fcgi?cmd=Retrieve&db=pubmed&dopt=Abstract&list_uids=25584414|6|Mol Endocrinol|29|3|Mar|2015|Journal Article,Research Support, N.I.H., Extramural,Research Support, Non-U.S. Gov't
irf4	prl	http://www.ncbi.nlm.nih.gov/entrez/query.fcgi?cmd=Retrieve&db=pubmed&dopt=Abstract&list_uids=25584414|6|Mol Endocrinol|29|3|Mar|2015|Journal Article,Research Support, N.I.H., Extramural,Research Support, Non-U.S. Gov't
irf4	wnt4	http://www.ncbi.nlm.nih.gov/entrez/query.fcgi?cmd=Retrieve&db=pubmed&dopt=Abstract&list_uids=25584414|6|Mol Endocrinol|29|3|Mar|2015|Journal Article,Research Support, N.I.H., Extramural,Research Support, Non-U.S. Gov't
igfbp1	prl	http://www.ncbi.nlm.nih.gov/entrez/query.fcgi?cmd=Retrieve&db=pubmed&dopt=Abstract&list_uids=25584414|6|Mol Endocrinol|29|3|Mar|2015|Journal Article,Research Support, N.I.H., Extramural,Research Support, Non-U.S. Gov't
igfbp1	wnt4	http://www.ncbi.nlm.nih.gov/entrez/query.fcgi?cmd=Retrieve&db=pubmed&dopt=Abstract&list_uids=25584414|6|Mol Endocrinol|29|3|Mar|2015|Journal Article,Research Support, N.I.H., Extramural,Research Support, Non-U.S. Gov't
prl	wnt4	http://www.ncbi.nlm.nih.gov/entrez/query.fcgi?cmd=Retrieve&db=pubmed&dopt=Abstract&list_uids=25584414|6|Mol Endocrinol|29|3|Mar|2015|Journal Article,Research Support, N.I.H., Extramural,Research Support, Non-U.S. Gov't
3.6.4.1	ephb2	http://www.ncbi.nlm.nih.gov/entrez/query.fcgi?cmd=Retrieve&db=pubmed&dopt=Abstract&list_uids=25550404|6|EMBO Rep|16|2|Feb|2015|Journal Article,Research Support, Non-U.S. Gov't
sgsm3	ephb2	http://www.ncbi.nlm.nih.gov/entrez/query.fcgi?cmd=Retrieve&db=pubmed&dopt=Abstract&list_uids=24466271|6|PLoS One|9|1||2014|Journal Article,Research Support, N.I.H., Extramural,Research Support, Non-U.S. Gov't|http://www.ncbi.nlm.nih.gov/entrez/query.fcgi?cmd=Retrieve&db=pubmed&dopt=Abstract&list_uids=25550404|6|EMBO Rep|16|2|Feb|2015|Journal Article,Research Support, Non-U.S. Gov't
sgsm3	myh14	http://www.ncbi.nlm.nih.gov/entrez/query.fcgi?cmd=Retrieve&db=pubmed&dopt=Abstract&list_uids=25550404|6|EMBO Rep|16|2|Feb|2015|Journal Article,Research Support, Non-U.S. Gov't
ephb2	myh14	http://www.ncbi.nlm.nih.gov/entrez/query.fcgi?cmd=Retrieve&db=pubmed&dopt=Abstract&list_uids=25550404|6|EMBO Rep|16|2|Feb|2015|Journal Article,Research Support, Non-U.S. Gov't
mmrn1	has2	http://www.ncbi.nlm.nih.gov/entrez/query.fcgi?cmd=Retrieve&db=pubmed&dopt=Abstract&list_uids=25549589|6|Matrix Biol|42||Mar|2015|Journal Article,Research Support, N.I.H., Extramural

actin	has2	http://www.ncbi.nlm.nih.gov/entrez/query.fcgi?cmd=Retrieve&db=pubmed&dopt=Abstract&list_uids=25549589|6|Matrix Biol|42||Mar|2015|Journal Article,Research Support, N.I.H., Extramural
actin	cd44	http://www.ncbi.nlm.nih.gov/entrez/query.fcgi?cmd=Retrieve&db=pubmed&dopt=Abstract&list_uids=25549589|6|Matrix Biol|42||Mar|2015|Journal Article,Research Support, N.I.H., Extramural
rac1	myh14	http://www.ncbi.nlm.nih.gov/entrez/query.fcgi?cmd=Retrieve&db=pubmed&dopt=Abstract&list_uids=25544611|6|Curr Biol|25|2|Jan|2015|Journal Article,Research Support, N.I.H., Extramural,Research Support, N.I.H., Intramural|http://www.ncbi.nlm.nih.gov/entrez/query.fcgi?cmd=Retrieve&db=pubmed&dopt=Abstract&list_uids=24980436|6|Mol Cell Biol|34|18|Sep|2014|Journal Article,Research Support, Non-U.S. Gov't|http://www.ncbi.nlm.nih.gov/entrez/query.fcgi?cmd=Retrieve&db=pubmed&dopt=Abstract&list_uids=23979717|6|J Cell Biol|202|5|Sep|2013|Journal Article,Research Support, N.I.H., Extramural,Research Support, Non-U.S. Gov't,Research Support, U.S. Gov't, Non-P.H.S.
rac1	actin	http://www.ncbi.nlm.nih.gov/entrez/query.fcgi?cmd=Retrieve&db=pubmed&dopt=Abstract&list_uids=25544611|6|Curr Biol|25|2|Jan|2015|Journal Article,Research Support, N.I.H., Extramural,Research Support, N.I.H., Intramural|http://www.ncbi.nlm.nih.gov/entrez/query.fcgi?cmd=Retrieve&db=pubmed&dopt=Abstract&list_uids=23826261|6|PLoS One|8|6||2013|Journal Article,Research Support, Non-U.S. Gov't|http://www.ncbi.nlm.nih.gov/entrez/query.fcgi?cmd=Retrieve&db=pubmed&dopt=Abstract&list_uids=22879066|6|Adv Exp Med Biol|986|||2013|Journal Article,Review|http://www.ncbi.nlm.nih.gov/entrez/query.fcgi?cmd=Retrieve&db=pubmed&dopt=Abstract&list_uids=23132927|6|J Cell Sci|126|Pt 1|Jan|2013|Journal Article,Research Support, Non-U.S. Gov't
pkc	rac1	http://www.ncbi.nlm.nih.gov/entrez/query.fcgi?cmd=Retrieve&db=pubmed&dopt=Abstract&list_uids=25544611|6|Curr Biol|25|2|Jan|2015|Journal Article,Research Support, N.I.H., Extramural,Research Support, N.I.H., Intramural
fas	pkc	http://www.ncbi.nlm.nih.gov/entrez/query.fcgi?cmd=Retrieve&db=pubmed&dopt=Abstract&list_uids=25544611|6|Curr Biol|25|2|Jan|2015|Journal Article,Research Support, N.I.H., Extramural,Research Support, N.I.H., Intramural
mmrn1	pkc	http://www.ncbi.nlm.nih.gov/entrez/query.fcgi?cmd=Retrieve&db=pubmed&dopt=Abstract&list_uids=25544611|6|Curr Biol|25|2|Jan|2015|Journal Article,Research Support, N.I.H., Extramural,Research Support, N.I.H., Intramural
cd44	itgb1	http://www.ncbi.nlm.nih.gov/entrez/query.fcgi?cmd=Retrieve&db=pubmed&dopt=Abstract&list_uids=25534714|6|Biochim Biophys Acta|1848|3|Mar|2015|Journal Article,Research Support, Non-U.S. Gov't
cd44	vegfa	http://www.ncbi.nlm.nih.gov/entrez/query.fcgi?cmd=Retrieve&db=pubmed&dopt=Abstract&list_uids=25534714|6|Biochim Biophys Acta|1848|3|Mar|2015|Journal Article,Research Support, Non-U.S. Gov't
itgb1	vegfa	http://www.ncbi.nlm.nih.gov/entrez/query.fcgi?cmd=Retrieve&db=pubmed&dopt=Abstract&list_uids=25534714|6|Biochim Biophys Acta|1848|3|Mar|2015|Journal Article,Research Support, Non-U.S. Gov't
actin	mtg1	http://www.ncbi.nlm.nih.gov/entrez/query.fcgi?cmd=Retrieve&db=pubmed&dopt=Abstract&list_uids=25530216|6|Cell Signal|27|3|Mar|2015|Journal Article,Research Support, Non-U.S. Gov't
actin	cdkn2a	http://www.ncbi.nlm.nih.gov/entrez/query.fcgi?cmd=Retrieve&db=pubmed&dopt=Abstract&list_uids=25530216|6|Cell Signal|27|3|Mar|2015|Journal Article,Research Support, Non-U.S. Gov't
mtg1	cdkn2a	http://www.ncbi.nlm.nih.gov/entrez/query.fcgi?cmd=Retrieve&db=pubmed&dopt=Abstract&list_uids=25530216|6|Cell

		Signal|27|3|Mar|2015|Journal Article,Research Support, Non-U.S. Gov't|http://www.ncbi.nlm.nih.gov/entrez/query.fcgi?cmd=Retrieve&db=pubmed&dopt=Abstract&list_uids=23129116|6|Curr Protoc Cell Biol|Chapter 17||Jun|2012|Journal Article,Research Support, Non-U.S. Gov't,Review
arf1	egf	http://www.ncbi.nlm.nih.gov/entrez/query.fcgi?cmd=Retrieve&db=pubmed&dopt=Abstract&list_uids=25530216|6|Cell Signal|27|3|Mar|2015|Journal Article,Research Support, Non-U.S. Gov't
arf1	ptk2	http://www.ncbi.nlm.nih.gov/entrez/query.fcgi?cmd=Retrieve&db=pubmed&dopt=Abstract&list_uids=25530216|6|Cell Signal|27|3|Mar|2015|Journal Article,Research Support, Non-U.S. Gov't
arf1	src	http://www.ncbi.nlm.nih.gov/entrez/query.fcgi?cmd=Retrieve&db=pubmed&dopt=Abstract&list_uids=25530216|6|Cell Signal|27|3|Mar|2015|Journal Article,Research Support, Non-U.S. Gov't
dlc1	rho	http://www.ncbi.nlm.nih.gov/entrez/query.fcgi?cmd=Retrieve&db=pubmed&dopt=Abstract&list_uids=25525271|6|J Biol Chem|290|7|Feb|2015|Journal Article,Research Support, Non-U.S. Gov't
dlc1	rasa1	http://www.ncbi.nlm.nih.gov/entrez/query.fcgi?cmd=Retrieve&db=pubmed&dopt=Abstract&list_uids=25525271|6|J Biol Chem|290|7|Feb|2015|Journal Article,Research Support, Non-U.S. Gov't|http://www.ncbi.nlm.nih.gov/entrez/query.fcgi?cmd=Retrieve&db=pubmed&dopt=Abstract&list_uids=25448629|6|Cytoskeleton (Hoboken)|71|12|Dec|2014|Journal Article,Research Support, Non-U.S. Gov't
rho	rasa1	http://www.ncbi.nlm.nih.gov/entrez/query.fcgi?cmd=Retrieve&db=pubmed&dopt=Abstract&list_uids=25525271|6|J Biol Chem|290|7|Feb|2015|Journal Article,Research Support, Non-U.S. Gov't
egf	rasa1	http://www.ncbi.nlm.nih.gov/entrez/query.fcgi?cmd=Retrieve&db=pubmed&dopt=Abstract&list_uids=25525271|6|J Biol Chem|290|7|Feb|2015|Journal Article,Research Support, Non-U.S. Gov't
egf	dlc1	http://www.ncbi.nlm.nih.gov/entrez/query.fcgi?cmd=Retrieve&db=pubmed&dopt=Abstract&list_uids=25525271|6|J Biol Chem|290|7|Feb|2015|Journal Article,Research Support, Non-U.S. Gov't|http://www.ncbi.nlm.nih.gov/entrez/query.fcgi?cmd=Retrieve&db=pubmed&dopt=Abstract&list_uids=22307599|6|Proc Natl Acad Sci U S A|109|5|Jan|2012|Journal Article,Research Support, Non-U.S. Gov't
egf	ephb2	http://www.ncbi.nlm.nih.gov/entrez/query.fcgi?cmd=Retrieve&db=pubmed&dopt=Abstract&list_uids=25525271|6|J Biol Chem|290|7|Feb|2015|Journal Article,Research Support, Non-U.S. Gov't
egf	ppp2r4	http://www.ncbi.nlm.nih.gov/entrez/query.fcgi?cmd=Retrieve&db=pubmed&dopt=Abstract&list_uids=25525271|6|J Biol Chem|290|7|Feb|2015|Journal Article,Research Support, Non-U.S. Gov't
rasa1	ephb2	http://www.ncbi.nlm.nih.gov/entrez/query.fcgi?cmd=Retrieve&db=pubmed&dopt=Abstract&list_uids=25525271|6|J Biol Chem|290|7|Feb|2015|Journal Article,Research Support, Non-U.S. Gov't
rasa1	ppp2r4	http://www.ncbi.nlm.nih.gov/entrez/query.fcgi?cmd=Retrieve&db=pubmed&dopt=Abstract&list_uids=25525271|6|J Biol Chem|290|7|Feb|2015|Journal Article,Research Support, Non-U.S. Gov't
dlc1	ephb2	http://www.ncbi.nlm.nih.gov/entrez/query.fcgi?cmd=Retrieve&db=pubmed&dopt=Abstract&list_uids=25525271|6|J Biol Chem|290|7|Feb|2015|Journal Article,Research Support, Non-U.S. Gov't
dlc1	ppp2r4	http://www.ncbi.nlm.nih.gov/entrez/query.fcgi?cmd=Retrieve&db=pubmed&dopt=Abstract&list_uids=25525271|6|J Biol Chem|290|7|Feb|2015|Journal Article,Research Support, Non-U.S. Gov't
ephb2	ppp2r4	http://www.ncbi.nlm.nih.gov/entrez/query.fcgi?cmd=Retrieve&db=pubmed&dopt=Abstract&list_uids=25525271|6|J Biol

		Chem|290|7|Feb|2015|Journal Article,Research Support, Non-U.S. Gov't
dpp9	dpp8	http://www.ncbi.nlm.nih.gov/entrez/query.fcgi?cmd=Retrieve&db=pubmed&dopt=Abstract&list_uids=25486458|6|Biochim Biophys Acta|1853|2|Feb|2015|Journal Article,Research Support, Non-U.S. Gov't
ptk2	hcfc1r1	http://www.ncbi.nlm.nih.gov/entrez/query.fcgi?cmd=Retrieve&db=pubmed&dopt=Abstract&list_uids=25486428|6|Oncogene|34|35|Aug|2015|Journal Article,Research Support, Non-U.S. Gov't
actin	csk	http://www.ncbi.nlm.nih.gov/entrez/query.fcgi?cmd=Retrieve&db=pubmed&dopt=Abstract&list_uids=25482640|6|Cell Adh Migr|8|6||2014|Journal Article,Research Support, N.I.H., Extramural
lims1	actin	http://www.ncbi.nlm.nih.gov/entrez/query.fcgi?cmd=Retrieve&db=pubmed&dopt=Abstract&list_uids=25482629|6|Cell Adh Migr|9|3||2015|Journal Article,Research Support, U.S. Gov't, Non-P.H.S.
ipp	actin	http://www.ncbi.nlm.nih.gov/entrez/query.fcgi?cmd=Retrieve&db=pubmed&dopt=Abstract&list_uids=25482629|6|Cell Adh Migr|9|3||2015|Journal Article,Research Support, U.S. Gov't, Non-P.H.S.
ilk	rsu1	http://www.ncbi.nlm.nih.gov/entrez/query.fcgi?cmd=Retrieve&db=pubmed&dopt=Abstract&list_uids=25482629|6|Cell Adh Migr|9|3||2015|Journal Article,Research Support, U.S. Gov't, Non-P.H.S.
lims1	rsu1	http://www.ncbi.nlm.nih.gov/entrez/query.fcgi?cmd=Retrieve&db=pubmed&dopt=Abstract&list_uids=25482629|6|Cell Adh Migr|9|3||2015|Journal Article,Research Support, U.S. Gov't, Non-P.H.S.
rsu1	fas	http://www.ncbi.nlm.nih.gov/entrez/query.fcgi?cmd=Retrieve&db=pubmed&dopt=Abstract&list_uids=25482629|6|Cell Adh Migr|9|3||2015|Journal Article,Research Support, U.S. Gov't, Non-P.H.S.
lims1	fas	http://www.ncbi.nlm.nih.gov/entrez/query.fcgi?cmd=Retrieve&db=pubmed&dopt=Abstract&list_uids=25482629|6|Cell Adh Migr|9|3||2015|Journal Article,Research Support, U.S. Gov't, Non-P.H.S.
rsu1	egf	http://www.ncbi.nlm.nih.gov/entrez/query.fcgi?cmd=Retrieve&db=pubmed&dopt=Abstract&list_uids=25482629|6|Cell Adh Migr|9|3||2015|Journal Article,Research Support, U.S. Gov't, Non-P.H.S.
rsu1	mapk14	http://www.ncbi.nlm.nih.gov/entrez/query.fcgi?cmd=Retrieve&db=pubmed&dopt=Abstract&list_uids=25482629|6|Cell Adh Migr|9|3||2015|Journal Article,Research Support, U.S. Gov't, Non-P.H.S.
rsu1	atf2	http://www.ncbi.nlm.nih.gov/entrez/query.fcgi?cmd=Retrieve&db=pubmed&dopt=Abstract&list_uids=25482629|6|Cell Adh Migr|9|3||2015|Journal Article,Research Support, U.S. Gov't, Non-P.H.S.
rsu1	ipp	http://www.ncbi.nlm.nih.gov/entrez/query.fcgi?cmd=Retrieve&db=pubmed&dopt=Abstract&list_uids=25482629|6|Cell Adh Migr|9|3||2015|Journal Article,Research Support, U.S. Gov't, Non-P.H.S.
lims1	egf	http://www.ncbi.nlm.nih.gov/entrez/query.fcgi?cmd=Retrieve&db=pubmed&dopt=Abstract&list_uids=25482629|6|Cell Adh Migr|9|3||2015|Journal Article,Research Support, U.S. Gov't, Non-P.H.S.
lims1	mapk14	http://www.ncbi.nlm.nih.gov/entrez/query.fcgi?cmd=Retrieve&db=pubmed&dopt=Abstract&list_uids=25482629|6|Cell Adh Migr|9|3||2015|Journal Article,Research Support, U.S. Gov't, Non-P.H.S.
lims1	atf2	http://www.ncbi.nlm.nih.gov/entrez/query.fcgi?cmd=Retrieve&db=pubmed&dopt=Abstract&list_uids=25482629|6|Cell Adh Migr|9|3||2015|Journal Article,Research Support, U.S. Gov't, Non-P.H.S.
egf	mapk14	http://www.ncbi.nlm.nih.gov/entrez/query.fcgi?cmd=Retrieve&db=pubmed&dopt=Abstract&list_uids=25014166|6|Biochim Biophys Acta|1843|11|Nov|2014|Journal Article|http://www.ncbi.nlm.nih.gov/entrez/query.fcgi?cmd=Retrieve&db=pubmed&dopt=Abstract&list_uids=25482629|6|C

		ell Adh Migr|9|3||2015|Journal Article,Research Support, U.S. Gov't, Non-P.H.S.
egf	atf2	http://www.ncbi.nlm.nih.gov/entrez/query.fcgi?cmd=Retrieve&db=pubmed&dopt=Abstract&list_uids=25482629|6|Cell Adh Migr|9|3||2015|Journal Article,Research Support, U.S. Gov't, Non-P.H.S.
egf	ipp	http://www.ncbi.nlm.nih.gov/entrez/query.fcgi?cmd=Retrieve&db=pubmed&dopt=Abstract&list_uids=25482629|6|Cell Adh Migr|9|3||2015|Journal Article,Research Support, U.S. Gov't, Non-P.H.S.
mapk14	atf2	http://www.ncbi.nlm.nih.gov/entrez/query.fcgi?cmd=Retrieve&db=pubmed&dopt=Abstract&list_uids=25482629|6|Cell Adh Migr|9|3||2015|Journal Article,Research Support, U.S. Gov't, Non-P.H.S.
mapk14	ipp	http://www.ncbi.nlm.nih.gov/entrez/query.fcgi?cmd=Retrieve&db=pubmed&dopt=Abstract&list_uids=25482629|6|Cell Adh Migr|9|3||2015|Journal Article,Research Support, U.S. Gov't, Non-P.H.S.
atf2	ipp	http://www.ncbi.nlm.nih.gov/entrez/query.fcgi?cmd=Retrieve&db=pubmed&dopt=Abstract&list_uids=25482629|6|Cell Adh Migr|9|3||2015|Journal Article,Research Support, U.S. Gov't, Non-P.H.S.
fas	ipp	http://www.ncbi.nlm.nih.gov/entrez/query.fcgi?cmd=Retrieve&db=pubmed&dopt=Abstract&list_uids=25482629|6|Cell Adh Migr|9|3||2015|Journal Article,Research Support, U.S. Gov't, Non-P.H.S.
fas	egf	http://www.ncbi.nlm.nih.gov/entrez/query.fcgi?cmd=Retrieve&db=pubmed&dopt=Abstract&list_uids=25482629|6|Cell Adh Migr|9|3||2015|Journal Article,Research Support, U.S. Gov't, Non-P.H.S.
fas	mapk14	http://www.ncbi.nlm.nih.gov/entrez/query.fcgi?cmd=Retrieve&db=pubmed&dopt=Abstract&list_uids=25482629|6|Cell Adh Migr|9|3||2015|Journal Article,Research Support, U.S. Gov't, Non-P.H.S.
racgap1	rhoa	http://www.ncbi.nlm.nih.gov/entrez/query.fcgi?cmd=Retrieve&db=pubmed&dopt=Abstract&list_uids=25475728|6|Biochem Biophys Res Commun|459|1|Mar|2015|Journal Article,Research Support, Non-U.S. Gov't
racgap1	ptk2	http://www.ncbi.nlm.nih.gov/entrez/query.fcgi?cmd=Retrieve&db=pubmed&dopt=Abstract&list_uids=25475728|6|Biochem Biophys Res Commun|459|1|Mar|2015|Journal Article,Research Support, Non-U.S. Gov't
rhoa	ptk2	http://www.ncbi.nlm.nih.gov/entrez/query.fcgi?cmd=Retrieve&db=pubmed&dopt=Abstract&list_uids=22513979|6|J Pathol|228|1|Sep|2012|Journal Article,Research Support, Non-U.S. Gov't|http://www.ncbi.nlm.nih.gov/entrez/query.fcgi?cmd=Retrieve&db=pubmed&dopt=Abstract&list_uids=25471072|6|EMBO J|34|4|Feb|2015|Journal Article,Research Support, Non-U.S. Gov't|http://www.ncbi.nlm.nih.gov/entrez/query.fcgi?cmd=Retrieve&db=pubmed&dopt=Abstract&list_uids=23836527|6|Stem Cells|31|11|Nov|2013|Journal Article,Research Support, N.I.H., Extramural|http://www.ncbi.nlm.nih.gov/entrez/query.fcgi?cmd=Retrieve&db=pubmed&dopt=Abstract&list_uids=25475728|6|Biochem Biophys Res Commun|459|1|Mar|2015|Journal Article,Research Support, Non-U.S. Gov't|http://www.ncbi.nlm.nih.gov/entrez/query.fcgi?cmd=Retrieve&db=pubmed&dopt=Abstract&list_uids=22721990|6|Cardiovasc Res|95|3|Aug|2012|Journal Article,Research Support, Non-U.S. Gov't
ptk2b	racgap1	http://www.ncbi.nlm.nih.gov/entrez/query.fcgi?cmd=Retrieve&db=pubmed&dopt=Abstract&list_uids=25475728|6|Biochem Biophys Res Commun|459|1|Mar|2015|Journal Article,Research Support, Non-U.S. Gov't
crk	pik3r1	http://www.ncbi.nlm.nih.gov/entrez/query.fcgi?cmd=Retrieve&db=pubmed&dopt=Abstract&list_uids=25471072|6|EMBO J|34|4|Feb|2015|Journal Article,Research Support, Non-U.S. Gov't

ptk2	myh14	http://www.ncbi.nlm.nih.gov/entrez/query.fcgi?cmd=Retrieve&db=pubmed&dopt=Abstract&list_uids=22915102|6|J Neurosci|32|34|Aug|2012|Journal Article,Research Support, N.I.H., Extramural,Research Support, Non-U.S. Gov't,Validation Studies|http://www.ncbi.nlm.nih.gov/entrez/query.fcgi?cmd=Retrieve&db=pubmed&dopt=Abstract&list_uids=25471072|6|EMBO J|34|4|Feb|2015|Journal Article,Research Support, Non-U.S. Gov't|http://www.ncbi.nlm.nih.gov/entrez/query.fcgi?cmd=Retrieve&db=pubmed&dopt=Abstract&list_uids=23600470|6|Microcirculation|20|7|Oct|2013|Journal Article,Research Support, N.I.H., Extramural
depdc1b	rhoa	http://www.ncbi.nlm.nih.gov/entrez/query.fcgi?cmd=Retrieve&db=pubmed&dopt=Abstract&list_uids=25458010|6|Dev Cell|31|4|Nov|2014|Journal Article,Research Support, Non-U.S. Gov't
depdc1b	ptprf	http://www.ncbi.nlm.nih.gov/entrez/query.fcgi?cmd=Retrieve&db=pubmed&dopt=Abstract&list_uids=25458010|6|Dev Cell|31|4|Nov|2014|Journal Article,Research Support, Non-U.S. Gov't
depdc1b	fas	http://www.ncbi.nlm.nih.gov/entrez/query.fcgi?cmd=Retrieve&db=pubmed&dopt=Abstract&list_uids=25458010|6|Dev Cell|31|4|Nov|2014|Journal Article,Research Support, Non-U.S. Gov't
rhoa	ptprf	http://www.ncbi.nlm.nih.gov/entrez/query.fcgi?cmd=Retrieve&db=pubmed&dopt=Abstract&list_uids=25458010|6|Dev Cell|31|4|Nov|2014|Journal Article,Research Support, Non-U.S. Gov't
rhoa	fas	http://www.ncbi.nlm.nih.gov/entrez/query.fcgi?cmd=Retrieve&db=pubmed&dopt=Abstract&list_uids=25458010|6|Dev Cell|31|4|Nov|2014|Journal Article,Research Support, Non-U.S. Gov't|http://www.ncbi.nlm.nih.gov/entrez/query.fcgi?cmd=Retrieve&db=pubmed&dopt=Abstract&list_uids=23344455|6|Toxins (Basel)|5|1|Jan|2013|Comparative Study,Journal Article,Research Support, Non-U.S. Gov't
ptprf	fas	http://www.ncbi.nlm.nih.gov/entrez/query.fcgi?cmd=Retrieve&db=pubmed&dopt=Abstract&list_uids=25458010|6|Dev Cell|31|4|Nov|2014|Journal Article,Research Support, Non-U.S. Gov't
cdk5	dlc1	http://www.ncbi.nlm.nih.gov/entrez/query.fcgi?cmd=Retrieve&db=pubmed&dopt=Abstract&list_uids=25452387|6|J Cell Biol|207|5|Dec|2014|Journal Article,Research Support, N.I.H., Intramural
lpa	src	http://www.ncbi.nlm.nih.gov/entrez/query.fcgi?cmd=Retrieve&db=pubmed&dopt=Abstract&list_uids=25451317|6|Cancer Lett|356|2 Pt B|Jan|2015|Journal Article,Research Support, N.I.H., Extramural
rac1	bcar1	http://www.ncbi.nlm.nih.gov/entrez/query.fcgi?cmd=Retrieve&db=pubmed&dopt=Abstract&list_uids=25451317|6|Cancer Lett|356|2 Pt B|Jan|2015|Journal Article,Research Support, N.I.H., Extramural|http://www.ncbi.nlm.nih.gov/entrez/query.fcgi?cmd=Retrieve&db=pubmed&dopt=Abstract&list_uids=24480309|6|Arch Biochem Biophys|545||Mar|2014|Journal Article,Research Support, Non-U.S. Gov't
tmlhe	pry	http://www.ncbi.nlm.nih.gov/entrez/query.fcgi?cmd=Retrieve&db=pubmed&dopt=Abstract&list_uids=25450970|6|Biochim Biophys Acta|1853|2|Feb|2015|Journal Article,Research Support, Non-U.S. Gov't
tmlhe	trim15	http://www.ncbi.nlm.nih.gov/entrez/query.fcgi?cmd=Retrieve&db=pubmed&dopt=Abstract&list_uids=25450970|6|Biochim Biophys Acta|1853|2|Feb|2015|Journal Article,Research Support, Non-U.S. Gov't
pry	trim15	http://www.ncbi.nlm.nih.gov/entrez/query.fcgi?cmd=Retrieve&db=pubmed&dopt=Abstract&list_uids=25450970|6|Biochim Biophys Acta|1853|2|Feb|2015|Journal Article,Research Support, Non-U.S. Gov't
trim15	pdlim5	http://www.ncbi.nlm.nih.gov/entrez/query.fcgi?cmd=Retrieve&db=pubmed&dopt=Abstract&list_uids=25450970|6|Biochim Biophys Acta|1853|2|Feb|2015|Journal Article,Research Support, Non-U.S. Gov't

trim15	actin	http://www.ncbi.nlm.nih.gov/entrez/query.fcgi?cmd=Retrieve&db=pubmed&dopt=Abstract&list_uids=25450970|6|Biochim Biophys Acta|1853|2|Feb|2015|Journal Article,Research Support, Non-U.S. Gov't
pdlim5	actin	http://www.ncbi.nlm.nih.gov/entrez/query.fcgi?cmd=Retrieve&db=pubmed&dopt=Abstract&list_uids=24165133|6|J Biol Chem|288|49|Dec|2013|Journal Article,Research Support, N.I.H., Extramural,Research Support, Non-U.S. Gov't|http://www.ncbi.nlm.nih.gov/entrez/query.fcgi?cmd=Retrieve&db=pubmed&dopt=Abstract&list_uids=23974244|6|Cell Mol Life Sci|71|10|May|2014|Journal Article,Research Support, Non-U.S. Gov't|http://www.ncbi.nlm.nih.gov/entrez/query.fcgi?cmd=Retrieve&db=pubmed&dopt=Abstract&list_uids=25450970|6|Biochim Biophys Acta|1853|2|Feb|2015|Journal Article,Research Support, Non-U.S. Gov't
dlc1	fas	http://www.ncbi.nlm.nih.gov/entrez/query.fcgi?cmd=Retrieve&db=pubmed&dopt=Abstract&list_uids=25448629|6|Cytoskeleton (Hoboken)|71|12|Dec|2014|Journal Article,Research Support, Non-U.S. Gov't
fas	hgf	http://www.ncbi.nlm.nih.gov/entrez/query.fcgi?cmd=Retrieve&db=pubmed&dopt=Abstract&list_uids=25447308|6|Exp Cell Res|330|2|Jan|2015|Journal Article,Research Support, Non-U.S. Gov't
fas	rap1a	http://www.ncbi.nlm.nih.gov/entrez/query.fcgi?cmd=Retrieve&db=pubmed&dopt=Abstract&list_uids=25447308|6|Exp Cell Res|330|2|Jan|2015|Journal Article,Research Support, Non-U.S. Gov't
hgf	rap1a	http://www.ncbi.nlm.nih.gov/entrez/query.fcgi?cmd=Retrieve&db=pubmed&dopt=Abstract&list_uids=25447308|6|Exp Cell Res|330|2|Jan|2015|Journal Article,Research Support, Non-U.S. Gov't
fat	fas	http://www.ncbi.nlm.nih.gov/entrez/query.fcgi?cmd=Retrieve&db=pubmed&dopt=Abstract&list_uids=25391654|6|J Biol Chem|290|1|Jan|2015|Journal Article,Research Support, Non-U.S. Gov't
fat	tyr	http://www.ncbi.nlm.nih.gov/entrez/query.fcgi?cmd=Retrieve&db=pubmed&dopt=Abstract&list_uids=25391654|6|J Biol Chem|290|1|Jan|2015|Journal Article,Research Support, Non-U.S. Gov't
fat	grb2	http://www.ncbi.nlm.nih.gov/entrez/query.fcgi?cmd=Retrieve&db=pubmed&dopt=Abstract&list_uids=25391654|6|J Biol Chem|290|1|Jan|2015|Journal Article,Research Support, Non-U.S. Gov't
tyr	grb2	http://www.ncbi.nlm.nih.gov/entrez/query.fcgi?cmd=Retrieve&db=pubmed&dopt=Abstract&list_uids=25391654|6|J Biol Chem|290|1|Jan|2015|Journal Article,Research Support, Non-U.S. Gov't
ptk2	tyr	http://www.ncbi.nlm.nih.gov/entrez/query.fcgi?cmd=Retrieve&db=pubmed&dopt=Abstract&list_uids=25391654|6|J Biol Chem|290|1|Jan|2015|Journal Article,Research Support, Non-U.S. Gov't|http://www.ncbi.nlm.nih.gov/entrez/query.fcgi?cmd=Retrieve&db=pubmed&dopt=Abstract&list_uids=23637231|6|J Biol Chem|288|24|Jun|2013|Journal Article,Research Support, N.I.H., Extramural,Research Support, Non-U.S. Gov't|http://www.ncbi.nlm.nih.gov/entrez/query.fcgi?cmd=Retrieve&db=pubmed&dopt=Abstract&list_uids=24586773|6|PLoS One|9|2||2014|Journal Article,Research Support, N.I.H., Extramural,Research Support, Non-U.S. Gov't
ephb2	fat	http://www.ncbi.nlm.nih.gov/entrez/query.fcgi?cmd=Retrieve&db=pubmed&dopt=Abstract&list_uids=25391654|6|J Biol Chem|290|1|Jan|2015|Journal Article,Research Support, Non-U.S. Gov't
ate1	actin	http://www.ncbi.nlm.nih.gov/entrez/query.fcgi?cmd=Retrieve&db=pubmed&dopt=Abstract&list_uids=25381249|6|J Biol Chem|289|51|Dec|2014|Journal Article,Research Support, Non-U.S. Gov't
eps8	src	http://www.ncbi.nlm.nih.gov/entrez/query.fcgi?cmd=Retrieve&db=pubmed&dopt=Abstract&list_uids=25359883|6|J Cell Sci|127|Pt 24|Dec|2014|Journal Article,Research Support, Non-U.S. Gov't

eps8	ptk2	http://www.ncbi.nlm.nih.gov/entrez/query.fcgi?cmd=Retrieve&db=pubmed&dopt=Abstract&list_uids=25359883|6|J Cell Sci|127|Pt 24|Dec|2014|Journal Article,Research Support, Non-U.S. Gov't
eps8	actin	http://www.ncbi.nlm.nih.gov/entrez/query.fcgi?cmd=Retrieve&db=pubmed&dopt=Abstract&list_uids=25359883|6|J Cell Sci|127|Pt 24|Dec|2014|Journal Article,Research Support, Non-U.S. Gov't
cd4	cd8a	http://www.ncbi.nlm.nih.gov/entrez/query.fcgi?cmd=Retrieve&db=pubmed&dopt=Abstract&list_uids=25310899|6|Clin Exp Immunol|179|3|Mar|2015|Journal Article
cd4	1.13.11 .17	http://www.ncbi.nlm.nih.gov/entrez/query.fcgi?cmd=Retrieve&db=pubmed&dopt=Abstract&list_uids=25310899|6|Clin Exp Immunol|179|3|Mar|2015|Journal Article
cd8a	1.13.11 .17	http://www.ncbi.nlm.nih.gov/entrez/query.fcgi?cmd=Retrieve&db=pubmed&dopt=Abstract&list_uids=25310899|6|Clin Exp Immunol|179|3|Mar|2015|Journal Article
1.13.11 .17	cd274	http://www.ncbi.nlm.nih.gov/entrez/query.fcgi?cmd=Retrieve&db=pubmed&dopt=Abstract&list_uids=25310899|6|Clin Exp Immunol|179|3|Mar|2015|Journal Article
1.13.11 .17	icam1	http://www.ncbi.nlm.nih.gov/entrez/query.fcgi?cmd=Retrieve&db=pubmed&dopt=Abstract&list_uids=25310899|6|Clin Exp Immunol|179|3|Mar|2015|Journal Article
cd274	icam1	http://www.ncbi.nlm.nih.gov/entrez/query.fcgi?cmd=Retrieve&db=pubmed&dopt=Abstract&list_uids=25310899|6|Clin Exp Immunol|179|3|Mar|2015|Journal Article
nme1	nme2	http://www.ncbi.nlm.nih.gov/entrez/query.fcgi?cmd=Retrieve&db=pubmed&dopt=Abstract&list_uids=25249619|6|Nucleic Acids Res|42|18|Oct|2014|Journal Article,Research Support, N.I.H., Extramural,Research Support, Non-U.S. Gov't
snw1	actin	http://www.ncbi.nlm.nih.gov/entrez/query.fcgi?cmd=Retrieve&db=pubmed&dopt=Abstract&list_uids=25241900|6|Oncogene|34|28|Jul|2015|Journal Article,Research Support, Non-U.S. Gov't
snw1	pten	http://www.ncbi.nlm.nih.gov/entrez/query.fcgi?cmd=Retrieve&db=pubmed&dopt=Abstract&list_uids=25241900|6|Oncogene|34|28|Jul|2015|Journal Article,Research Support, Non-U.S. Gov't
cpeb4	fbn1	http://www.ncbi.nlm.nih.gov/entrez/query.fcgi?cmd=Retrieve&db=pubmed&dopt=Abstract&list_uids=25237887|6|PLoS One|9|9||2014|Journal Article,Research Support, Non-U.S. Gov't
jun	cpeb4	http://www.ncbi.nlm.nih.gov/entrez/query.fcgi?cmd=Retrieve&db=pubmed&dopt=Abstract&list_uids=25237887|6|PLoS One|9|9||2014|Journal Article,Research Support, Non-U.S. Gov't
jun	sorbs3	http://www.ncbi.nlm.nih.gov/entrez/query.fcgi?cmd=Retrieve&db=pubmed&dopt=Abstract&list_uids=25237887|6|PLoS One|9|9||2014|Journal Article,Research Support, Non-U.S. Gov't
mapk8	cpeb4	http://www.ncbi.nlm.nih.gov/entrez/query.fcgi?cmd=Retrieve&db=pubmed&dopt=Abstract&list_uids=25237887|6|PLoS One|9|9||2014|Journal Article,Research Support, Non-U.S. Gov't
mapk8	sorbs3	http://www.ncbi.nlm.nih.gov/entrez/query.fcgi?cmd=Retrieve&db=pubmed&dopt=Abstract&list_uids=25237887|6|PLoS One|9|9||2014|Journal Article,Research Support, Non-U.S. Gov't
cpeb4	sorbs3	http://www.ncbi.nlm.nih.gov/entrez/query.fcgi?cmd=Retrieve&db=pubmed&dopt=Abstract&list_uids=25237887|6|PLoS One|9|9||2014|Journal Article,Research Support, Non-U.S. Gov't
fat	cish	http://www.ncbi.nlm.nih.gov/entrez/query.fcgi?cmd=Retrieve&db=pubmed&dopt=Abstract&list_uids=25226367|6|PLoS One|9|9||2014|Journal Article,Research Support, Non-U.S. Gov't

arf6	arap2	http://www.ncbi.nlm.nih.gov/entrez/query.fcgi?cmd=Retrieve&db=pubmed&dopt=Abstract&list_uids=25225293|6|J Biol Chem|289|44|Oct|2014|Journal Article,Research Support, N.I.H., Intramural|http://www.ncbi.nlm.nih.gov/entrez/query.fcgi?cmd=Retrieve&db=pubmed&dopt=Abstract&list_uids=23295182|6|J Biol Chem|288|8|Feb|2013|Journal Article,Research Support, N.I.H., Intramural
arf6	acap1	http://www.ncbi.nlm.nih.gov/entrez/query.fcgi?cmd=Retrieve&db=pubmed&dopt=Abstract&list_uids=25225293|6|J Biol Chem|289|44|Oct|2014|Journal Article,Research Support, N.I.H., Intramural
arap2	acap1	http://www.ncbi.nlm.nih.gov/entrez/query.fcgi?cmd=Retrieve&db=pubmed&dopt=Abstract&list_uids=25225293|6|J Biol Chem|289|44|Oct|2014|Journal Article,Research Support, N.I.H., Intramural
arf6	rasa1	http://www.ncbi.nlm.nih.gov/entrez/query.fcgi?cmd=Retrieve&db=pubmed&dopt=Abstract&list_uids=25225293|6|J Biol Chem|289|44|Oct|2014|Journal Article,Research Support, N.I.H., Intramural|http://www.ncbi.nlm.nih.gov/entrez/query.fcgi?cmd=Retrieve&db=pubmed&dopt=Abstract&list_uids=23295182|6|J Biol Chem|288|8|Feb|2013|Journal Article,Research Support, N.I.H., Intramural
rasa1	acap1	http://www.ncbi.nlm.nih.gov/entrez/query.fcgi?cmd=Retrieve&db=pubmed&dopt=Abstract&list_uids=25225293|6|J Biol Chem|289|44|Oct|2014|Journal Article,Research Support, N.I.H., Intramural
ptbp1	appl1	http://www.ncbi.nlm.nih.gov/entrez/query.fcgi?cmd=Retrieve&db=pubmed&dopt=Abstract&list_uids=25225293|6|J Biol Chem|289|44|Oct|2014|Journal Article,Research Support, N.I.H., Intramural
ptbp1	eea1	http://www.ncbi.nlm.nih.gov/entrez/query.fcgi?cmd=Retrieve&db=pubmed&dopt=Abstract&list_uids=25225293|6|J Biol Chem|289|44|Oct|2014|Journal Article,Research Support, N.I.H., Intramural
ptbp1	arap2	http://www.ncbi.nlm.nih.gov/entrez/query.fcgi?cmd=Retrieve&db=pubmed&dopt=Abstract&list_uids=25225293|6|J Biol Chem|289|44|Oct|2014|Journal Article,Research Support, N.I.H., Intramural
ptbp1	rasa1	http://www.ncbi.nlm.nih.gov/entrez/query.fcgi?cmd=Retrieve&db=pubmed&dopt=Abstract&list_uids=25225293|6|J Biol Chem|289|44|Oct|2014|Journal Article,Research Support, N.I.H., Intramural
appl1	eea1	http://www.ncbi.nlm.nih.gov/entrez/query.fcgi?cmd=Retrieve&db=pubmed&dopt=Abstract&list_uids=25225293|6|J Biol Chem|289|44|Oct|2014|Journal Article,Research Support, N.I.H., Intramural
appl1	arap2	http://www.ncbi.nlm.nih.gov/entrez/query.fcgi?cmd=Retrieve&db=pubmed&dopt=Abstract&list_uids=25225293|6|J Biol Chem|289|44|Oct|2014|Journal Article,Research Support, N.I.H., Intramural
appl1	rasa1	http://www.ncbi.nlm.nih.gov/entrez/query.fcgi?cmd=Retrieve&db=pubmed&dopt=Abstract&list_uids=25225293|6|J Biol Chem|289|44|Oct|2014|Journal Article,Research Support, N.I.H., Intramural
eea1	arap2	http://www.ncbi.nlm.nih.gov/entrez/query.fcgi?cmd=Retrieve&db=pubmed&dopt=Abstract&list_uids=25225293|6|J Biol Chem|289|44|Oct|2014|Journal Article,Research Support, N.I.H., Intramural
eea1	rasa1	http://www.ncbi.nlm.nih.gov/entrez/query.fcgi?cmd=Retrieve&db=pubmed&dopt=Abstract&list_uids=25225293|6|J Biol Chem|289|44|Oct|2014|Journal Article,Research Support, N.I.H., Intramural
arap2	rasa1	http://www.ncbi.nlm.nih.gov/entrez/query.fcgi?cmd=Retrieve&db=pubmed&dopt=Abstract&list_uids=25225293|6|J Biol Chem|289|44|Oct|2014|Journal Article,Research Support, N.I.H., Intramural|http://www.ncbi.nlm.nih.gov/entrez/query.fcgi?cmd=Retrieve&db=pubmed&dopt=Abstract&list_uids=23295182|6|J Biol Chem|288|8|Feb|2013|Journal Article,Research Support, N.I.H., Intramural

arap2	fas	http://www.ncbi.nlm.nih.gov/entrez/query.fcgi?cmd=Retrieve&db=pubmed&dopt=Abstract&list_uids=25225293|6|J Biol Chem|289|44|Oct|2014|Journal Article,Research Support, N.I.H., Intramural|http://www.ncbi.nlm.nih.gov/entrez/query.fcgi?cmd=Retrieve&db=pubmed&dopt=Abstract&list_uids=23295182|6|J Biol Chem|288|8|Feb|2013|Journal Article,Research Support, N.I.H., Intramural
fas	acap1	http://www.ncbi.nlm.nih.gov/entrez/query.fcgi?cmd=Retrieve&db=pubmed&dopt=Abstract&list_uids=25225293|6|J Biol Chem|289|44|Oct|2014|Journal Article,Research Support, N.I.H., Intramural
map3k2	mapk8	http://www.ncbi.nlm.nih.gov/entrez/query.fcgi?cmd=Retrieve&db=pubmed&dopt=Abstract&list_uids=25190348|6|Biochem J|464|1|Nov|2014|Journal Article,Research Support, N.I.H., Extramural,Research Support, Non-U.S. Gov't
map3k2	jun	http://www.ncbi.nlm.nih.gov/entrez/query.fcgi?cmd=Retrieve&db=pubmed&dopt=Abstract&list_uids=25190348|6|Biochem J|464|1|Nov|2014|Journal Article,Research Support, N.I.H., Extramural,Research Support, Non-U.S. Gov't
mmrn1	pik3ca	http://www.ncbi.nlm.nih.gov/entrez/query.fcgi?cmd=Retrieve&db=pubmed&dopt=Abstract&list_uids=25183785|6|Cancer Res|74|17|Sep|2014|Journal Article,Research Support, N.I.H., Extramural,Research Support, Non-U.S. Gov't,Research Support, U.S. Gov't, P.H.S.
mmrn1	akt1	http://www.ncbi.nlm.nih.gov/entrez/query.fcgi?cmd=Retrieve&db=pubmed&dopt=Abstract&list_uids=25183785|6|Cancer Res|74|17|Sep|2014|Journal Article,Research Support, N.I.H., Extramural,Research Support, Non-U.S. Gov't,Research Support, U.S. Gov't, P.H.S.
akt1	pten	http://www.ncbi.nlm.nih.gov/entrez/query.fcgi?cmd=Retrieve&db=pubmed&dopt=Abstract&list_uids=25142071|6|Metallomics|6|12|Dec|2014|Journal Article,Research Support, Non-U.S. Gov't
akt1	mirn21	http://www.ncbi.nlm.nih.gov/entrez/query.fcgi?cmd=Retrieve&db=pubmed&dopt=Abstract&list_uids=25142071|6|Metallomics|6|12|Dec|2014|Journal Article,Research Support, Non-U.S. Gov't
pten	mirn21	http://www.ncbi.nlm.nih.gov/entrez/query.fcgi?cmd=Retrieve&db=pubmed&dopt=Abstract&list_uids=25142071|6|Metallomics|6|12|Dec|2014|Journal Article,Research Support, Non-U.S. Gov't
tlr4	ros1	http://www.ncbi.nlm.nih.gov/entrez/query.fcgi?cmd=Retrieve&db=pubmed&dopt=Abstract&list_uids=25130439|6|Int J Biochem Cell Biol|55||Oct|2014|Journal Article,Research Support, Non-U.S. Gov't
tlr4	pkc	http://www.ncbi.nlm.nih.gov/entrez/query.fcgi?cmd=Retrieve&db=pubmed&dopt=Abstract&list_uids=25130439|6|Int J Biochem Cell Biol|55||Oct|2014|Journal Article,Research Support, Non-U.S. Gov't
ros1	pkc	http://www.ncbi.nlm.nih.gov/entrez/query.fcgi?cmd=Retrieve&db=pubmed&dopt=Abstract&list_uids=25130439|6|Int J Biochem Cell Biol|55||Oct|2014|Journal Article,Research Support, Non-U.S. Gov't
trpm7	irf6	http://www.ncbi.nlm.nih.gov/entrez/query.fcgi?cmd=Retrieve&db=pubmed&dopt=Abstract&list_uids=25130439|6|Int J Biochem Cell Biol|55||Oct|2014|Journal Article,Research Support, Non-U.S. Gov't
pdlim7	pdlim5	http://www.ncbi.nlm.nih.gov/entrez/query.fcgi?cmd=Retrieve&db=pubmed&dopt=Abstract&list_uids=25124035|6|J Biol Chem|289|43|Oct|2014|Journal Article,Research Support, N.I.H., Extramural,Research Support, Non-U.S. Gov't
pdlim7	pkc	http://www.ncbi.nlm.nih.gov/entrez/query.fcgi?cmd=Retrieve&db=pubmed&dopt=Abstract&list_uids=25124035|6|J Biol Chem|289|43|Oct|2014|Journal Article,Research Support, N.I.H., Extramural,Research Support, Non-U.S. Gov't
pdlim5	pkc	http://www.ncbi.nlm.nih.gov/entrez/query.fcgi?cmd=Retrieve&db=pubmed&dopt=Abstract&list_uids=25124035|6|J Biol Chem|289|43|Oct|2014|Journal Article,Research Support, N.I.H., Extramural,Research Support, Non-U.S. Gov't

actin	msc	http://www.ncbi.nlm.nih.gov/entrez/query.fcgi?cmd=Retrieve&db=pubmed&dopt=Abstract&list_uids=25107365|6|J Cell Sci|127|Pt 19|Oct|2014|Journal Article,Research Support, Non-U.S. Gov't
arhgef2	msc	http://www.ncbi.nlm.nih.gov/entrez/query.fcgi?cmd=Retrieve&db=pubmed&dopt=Abstract&list_uids=25107365|6|J Cell Sci|127|Pt 19|Oct|2014|Journal Article,Research Support, Non-U.S. Gov't
fermt2	fermt3	http://www.ncbi.nlm.nih.gov/entrez/query.fcgi?cmd=Retrieve&db=pubmed&dopt=Abstract&list_uids=25086068|6|J Cell Sci|127|Pt 19|Oct|2014|Journal Article,Research Support, N.I.H., Extramural,Research Support, Non-U.S. Gov't
fermt2	fas	http://www.ncbi.nlm.nih.gov/entrez/query.fcgi?cmd=Retrieve&db=pubmed&dopt=Abstract&list_uids=25086068|6|J Cell Sci|127|Pt 19|Oct|2014|Journal Article,Research Support, N.I.H., Extramural,Research Support, Non-U.S. Gov't
fermt3	fas	http://www.ncbi.nlm.nih.gov/entrez/query.fcgi?cmd=Retrieve&db=pubmed&dopt=Abstract&list_uids=25086068|6|J Cell Sci|127|Pt 19|Oct|2014|Journal Article,Research Support, N.I.H., Extramural,Research Support, Non-U.S. Gov't
hps1	actin	http://www.ncbi.nlm.nih.gov/entrez/query.fcgi?cmd=Retrieve&db=pubmed&dopt=Abstract&list_uids=25050736|6|ACS Nano|8|8|Aug|2014|Journal Article,Research Support, Non-U.S. Gov't
mark1	dixdc1	http://www.ncbi.nlm.nih.gov/entrez/query.fcgi?cmd=Retrieve&db=pubmed&dopt=Abstract&list_uids=25042806|6|Mol Cell|55|3|Aug|2014|Journal Article,Research Support, N.I.H., Extramural,Research Support, Non-U.S. Gov't
prkaa1	stk11	http://www.ncbi.nlm.nih.gov/entrez/query.fcgi?cmd=Retrieve&db=pubmed&dopt=Abstract&list_uids=25042806|6|Mol Cell|55|3|Aug|2014|Journal Article,Research Support, N.I.H., Extramural,Research Support, Non-U.S. Gov't
bsg	hcc	http://www.ncbi.nlm.nih.gov/entrez/query.fcgi?cmd=Retrieve&db=pubmed&dopt=Abstract&list_uids=25033086|6|PLoS One|9|7||2014|Journal Article,Research Support, Non-U.S. Gov't
bsg	actr2	http://www.ncbi.nlm.nih.gov/entrez/query.fcgi?cmd=Retrieve&db=pubmed&dopt=Abstract&list_uids=25033086|6|PLoS One|9|7||2014|Journal Article,Research Support, Non-U.S. Gov't
fgfr2	fgf2	http://www.ncbi.nlm.nih.gov/entrez/query.fcgi?cmd=Retrieve&db=pubmed&dopt=Abstract&list_uids=25014166|6|Biochim Biophys Acta|1843|11|Nov|2014|Journal Article
ephb2	src	http://www.ncbi.nlm.nih.gov/entrez/query.fcgi?cmd=Retrieve&db=pubmed&dopt=Abstract&list_uids=25014166|6|Biochim Biophys Acta|1843|11|Nov|2014|Journal Article
ephb2	mapk14	http://www.ncbi.nlm.nih.gov/entrez/query.fcgi?cmd=Retrieve&db=pubmed&dopt=Abstract&list_uids=25014166|6|Biochim Biophys Acta|1843|11|Nov|2014|Journal Article|http://www.ncbi.nlm.nih.gov/entrez/query.fcgi?cmd=Retrieve&db=pubmed&dopt=Abstract&list_uids=24894398|6|Invest Ophthalmol Vis Sci|55|7|Jul|2014|Journal Article,Research Support, Non-U.S. Gov't
ephb2	rps6ka3	http://www.ncbi.nlm.nih.gov/entrez/query.fcgi?cmd=Retrieve&db=pubmed&dopt=Abstract&list_uids=25014166|6|Biochim Biophys Acta|1843|11|Nov|2014|Journal Article
src	mapk14	http://www.ncbi.nlm.nih.gov/entrez/query.fcgi?cmd=Retrieve&db=pubmed&dopt=Abstract&list_uids=25014166|6|Biochim Biophys Acta|1843|11|Nov|2014|Journal Article
src	rps6ka3	http://www.ncbi.nlm.nih.gov/entrez/query.fcgi?cmd=Retrieve&db=pubmed&dopt=Abstract&list_uids=25014166|6|Biochim Biophys Acta|1843|11|Nov|2014|Journal Article
mapk14	rps6ka3	http://www.ncbi.nlm.nih.gov/entrez/query.fcgi?cmd=Retrieve&db=pubmed&dopt=Abstract&list_uids=25014166|6|Biochim Biophys Acta|1843|11|Nov|2014|Journal Article

fgf2	mapk14	http://www.ncbi.nlm.nih.gov/entrez/query.fcgi?cmd=Retrieve&db=pubmed&dopt=Abstract&list_uids=25014166|6|Biochim Biophys Acta|1843|11|Nov|2014|Journal Article
fgf2	ephb2	http://www.ncbi.nlm.nih.gov/entrez/query.fcgi?cmd=Retrieve&db=pubmed&dopt=Abstract&list_uids=25014166|6|Biochim Biophys Acta|1843|11|Nov|2014|Journal Article|http://www.ncbi.nlm.nih.gov/entrez/query.fcgi?cmd=Retrieve&db=pubmed&dopt=Abstract&list_uids=24466271|6|PLoS One|9|1||2014|Journal Article,Research Support, N.I.H., Extramural,Research Support, Non-U.S. Gov't
fgf2	rps6ka3	http://www.ncbi.nlm.nih.gov/entrez/query.fcgi?cmd=Retrieve&db=pubmed&dopt=Abstract&list_uids=25014166|6|Biochim Biophys Acta|1843|11|Nov|2014|Journal Article
fgfr2	mapk14	http://www.ncbi.nlm.nih.gov/entrez/query.fcgi?cmd=Retrieve&db=pubmed&dopt=Abstract&list_uids=25014166|6|Biochim Biophys Acta|1843|11|Nov|2014|Journal Article
fgfr2	ephb2	http://www.ncbi.nlm.nih.gov/entrez/query.fcgi?cmd=Retrieve&db=pubmed&dopt=Abstract&list_uids=25014166|6|Biochim Biophys Acta|1843|11|Nov|2014|Journal Article
fgfr2	rps6ka3	http://www.ncbi.nlm.nih.gov/entrez/query.fcgi?cmd=Retrieve&db=pubmed&dopt=Abstract&list_uids=25014166|6|Biochim Biophys Acta|1843|11|Nov|2014|Journal Article
fgf2	egf	http://www.ncbi.nlm.nih.gov/entrez/query.fcgi?cmd=Retrieve&db=pubmed&dopt=Abstract&list_uids=25014166|6|Biochim Biophys Acta|1843|11|Nov|2014|Journal Article
fgf2	rps6ka1	http://www.ncbi.nlm.nih.gov/entrez/query.fcgi?cmd=Retrieve&db=pubmed&dopt=Abstract&list_uids=25014166|6|Biochim Biophys Acta|1843|11|Nov|2014|Journal Article
mapk14	rps6ka1	http://www.ncbi.nlm.nih.gov/entrez/query.fcgi?cmd=Retrieve&db=pubmed&dopt=Abstract&list_uids=25014166|6|Biochim Biophys Acta|1843|11|Nov|2014|Journal Article
egf	rps6ka1	http://www.ncbi.nlm.nih.gov/entrez/query.fcgi?cmd=Retrieve&db=pubmed&dopt=Abstract&list_uids=25014166|6|Biochim Biophys Acta|1843|11|Nov|2014|Journal Article
egf	fgfr2	http://www.ncbi.nlm.nih.gov/entrez/query.fcgi?cmd=Retrieve&db=pubmed&dopt=Abstract&list_uids=25014166|6|Biochim Biophys Acta|1843|11|Nov|2014|Journal Article
rps6ka1	fgfr2	http://www.ncbi.nlm.nih.gov/entrez/query.fcgi?cmd=Retrieve&db=pubmed&dopt=Abstract&list_uids=25014166|6|Biochim Biophys Acta|1843|11|Nov|2014|Journal Article
egfr	akt1	http://www.ncbi.nlm.nih.gov/entrez/query.fcgi?cmd=Retrieve&db=pubmed&dopt=Abstract&list_uids=24861878|6|Neuro Oncol|16|9|Sep|2014|Journal Article,Research Support, Non-U.S. Gov't|http://www.ncbi.nlm.nih.gov/entrez/query.fcgi?cmd=Retrieve&db=pubmed&dopt=Abstract&list_uids=25000176|6|PLoS One|9|7||2014|Journal Article,Research Support, N.I.H., Extramural,Research Support, U.S. Gov't, Non-P.H.S.
egfr	pi3	http://www.ncbi.nlm.nih.gov/entrez/query.fcgi?cmd=Retrieve&db=pubmed&dopt=Abstract&list_uids=25000176|6|PLoS One|9|7||2014|Journal Article,Research Support, N.I.H., Extramural,Research Support, U.S. Gov't, Non-P.H.S.
actr2	mmrn1	http://www.ncbi.nlm.nih.gov/entrez/query.fcgi?cmd=Retrieve&db=pubmed&dopt=Abstract&list_uids=24967897|6|PLoS One|9|6||2014|Journal Article,Research Support, Non-U.S. Gov't
actin	dstn	http://www.ncbi.nlm.nih.gov/entrez/query.fcgi?cmd=Retrieve&db=pubmed&dopt=Abstract&list_uids=24965068|6|Pflugers Arch|467|1|Jan|2015|Journal Article,Research Support, Non-U.S. Gov't,Review

isl1	pdlim5	http://www.ncbi.nlm.nih.gov/entrez/query.fcgi?cmd=Retrieve&db=pubmed&dopt=Abstract&list_uids=24933506|6|Trends Cell Biol|24|10|Oct|2014|Journal Article,Research Support, N.I.H., Extramural,Research Support, Non-U.S. Gov't,Review
bcar1	actin	http://www.ncbi.nlm.nih.gov/entrez/query.fcgi?cmd=Retrieve&db=pubmed&dopt=Abstract&list_uids=24928898|6|J Cell Sci|127|Pt 16|Aug|2014|Journal Article,Research Support, Non-U.S. Gov't
bcar1	3.6.4.1	http://www.ncbi.nlm.nih.gov/entrez/query.fcgi?cmd=Retrieve&db=pubmed&dopt=Abstract&list_uids=24928898|6|J Cell Sci|127|Pt 16|Aug|2014|Journal Article,Research Support, Non-U.S. Gov't
cse1l	actin	http://www.ncbi.nlm.nih.gov/entrez/query.fcgi?cmd=Retrieve&db=pubmed&dopt=Abstract&list_uids=24928898|6|J Cell Sci|127|Pt 16|Aug|2014|Journal Article,Research Support, Non-U.S. Gov't
cse1l	3.6.4.1	http://www.ncbi.nlm.nih.gov/entrez/query.fcgi?cmd=Retrieve&db=pubmed&dopt=Abstract&list_uids=24928898|6|J Cell Sci|127|Pt 16|Aug|2014|Journal Article,Research Support, Non-U.S. Gov't
actin	3.6.4.1	http://www.ncbi.nlm.nih.gov/entrez/query.fcgi?cmd=Retrieve&db=pubmed&dopt=Abstract&list_uids=24928898|6|J Cell Sci|127|Pt 16|Aug|2014|Journal Article,Research Support, Non-U.S. Gov't|http://www.ncbi.nlm.nih.gov/entrez/query.fcgi?cmd=Retrieve&db=pubmed&dopt=Abstract&list_uids=22524451|6|Cell Biol Int|36|7|Jul|2012|Journal Article,Research Support, Non-U.S. Gov't|http://www.ncbi.nlm.nih.gov/entrez/query.fcgi?cmd=Retrieve&db=pubmed&dopt=Abstract&list_uids=23378028|6|J Cell Sci|126|Pt 5|Mar|2013|Journal Article,Research Support, Non-U.S. Gov't|http://www.ncbi.nlm.nih.gov/entrez/query.fcgi?cmd=Retrieve&db=pubmed&dopt=Abstract&list_uids=24039980|6|PLoS One|8|9||2013|Journal Article,Research Support, N.I.H., Extramural,Research Support, Non-U.S. Gov't
frap1	cse1l	http://www.ncbi.nlm.nih.gov/entrez/query.fcgi?cmd=Retrieve&db=pubmed&dopt=Abstract&list_uids=24928898|6|J Cell Sci|127|Pt 16|Aug|2014|Journal Article,Research Support, Non-U.S. Gov't
frap1	src	http://www.ncbi.nlm.nih.gov/entrez/query.fcgi?cmd=Retrieve&db=pubmed&dopt=Abstract&list_uids=24928898|6|J Cell Sci|127|Pt 16|Aug|2014|Journal Article,Research Support, Non-U.S. Gov't
myh14	cse1l	http://www.ncbi.nlm.nih.gov/entrez/query.fcgi?cmd=Retrieve&db=pubmed&dopt=Abstract&list_uids=24928898|6|J Cell Sci|127|Pt 16|Aug|2014|Journal Article,Research Support, Non-U.S. Gov't|http://www.ncbi.nlm.nih.gov/entrez/query.fcgi?cmd=Retrieve&db=pubmed&dopt=Abstract&list_uids=22915102|6|J Neurosci|32|34|Aug|2012|Journal Article,Research Support, N.I.H., Extramural,Research Support, Non-U.S. Gov't,Validation Studies
3.6.4.1	src	http://www.ncbi.nlm.nih.gov/entrez/query.fcgi?cmd=Retrieve&db=pubmed&dopt=Abstract&list_uids=24928898|6|J Cell Sci|127|Pt 16|Aug|2014|Journal Article,Research Support, Non-U.S. Gov't|http://www.ncbi.nlm.nih.gov/entrez/query.fcgi?cmd=Retrieve&db=pubmed&dopt=Abstract&list_uids=23708000|6|Nat Cell Biol|15|6|Jun|2013|Journal Article,Research Support, Non-U.S. Gov't|http://www.ncbi.nlm.nih.gov/entrez/query.fcgi?cmd=Retrieve&db=pubmed&dopt=Abstract&list_uids=23209645|6|PLoS One|7|11||2012|Journal Article,Research Support, Non-U.S. Gov't
tm4sf5	stat3	http://www.ncbi.nlm.nih.gov/entrez/query.fcgi?cmd=Retrieve&db=pubmed&dopt=Abstract&list_uids=24912675|6|Mol Cell Biol|34|16|Aug|2014|Journal Article,Research Support, Non-U.S. Gov't
tm4sf5	ptk2	http://www.ncbi.nlm.nih.gov/entrez/query.fcgi?cmd=Retrieve&db=pubmed&dopt=Abstract&list_uids=24912675|6|Mol Cell

		Biol|34|16|Aug|2014|Journal Article,Research Support, Non-U.S. Gov't
il6	stat3	http://www.ncbi.nlm.nih.gov/entrez/query.fcgi?cmd=Retrieve&db=pubmed&dopt=Abstract&list_uids=24912675|6|Mol Cell Biol|34|16|Aug|2014|Journal Article,Research Support, Non-U.S. Gov't
il6	tm4sf5	http://www.ncbi.nlm.nih.gov/entrez/query.fcgi?cmd=Retrieve&db=pubmed&dopt=Abstract&list_uids=24912675|6|Mol Cell Biol|34|16|Aug|2014|Journal Article,Research Support, Non-U.S. Gov't
ptk2	stat3	http://www.ncbi.nlm.nih.gov/entrez/query.fcgi?cmd=Retrieve&db=pubmed&dopt=Abstract&list_uids=24861878|6|Neuro Oncol|16|9|Sep|2014|Journal Article,Research Support, Non-U.S. Gov't|http://www.ncbi.nlm.nih.gov/entrez/query.fcgi?cmd=Retrieve&db=pubmed&dopt=Abstract&list_uids=24912675|6|Mol Cell Biol|34|16|Aug|2014|Journal Article,Research Support, Non-U.S. Gov't|http://www.ncbi.nlm.nih.gov/entrez/query.fcgi?cmd=Retrieve&db=pubmed&dopt=Abstract&list_uids=24510996|6|Anticancer Res|34|2|Feb|2014|Journal Article
tm4sf5	il6r	http://www.ncbi.nlm.nih.gov/entrez/query.fcgi?cmd=Retrieve&db=pubmed&dopt=Abstract&list_uids=24912675|6|Mol Cell Biol|34|16|Aug|2014|Journal Article,Research Support, Non-U.S. Gov't
il6	il6r	http://www.ncbi.nlm.nih.gov/entrez/query.fcgi?cmd=Retrieve&db=pubmed&dopt=Abstract&list_uids=24912675|6|Mol Cell Biol|34|16|Aug|2014|Journal Article,Research Support, Non-U.S. Gov't
lpa	rhoa	http://www.ncbi.nlm.nih.gov/entrez/query.fcgi?cmd=Retrieve&db=pubmed&dopt=Abstract&list_uids=23689132|6|Mol Cell Biol|33|14|Jul|2013|Journal Article,Research Support, N.I.H., Extramural,Research Support, Non-U.S. Gov't|http://www.ncbi.nlm.nih.gov/entrez/query.fcgi?cmd=Retrieve&db=pubmed&dopt=Abstract&list_uids=24911199|6|FEBS Lett|588|17|Aug|2014|Journal Article,Research Support, Non-U.S. Gov't
rhoa	cdc42	http://www.ncbi.nlm.nih.gov/entrez/query.fcgi?cmd=Retrieve&db=pubmed&dopt=Abstract&list_uids=23615439|6|Mol Biol Cell|24|12|Jun|2013|Journal Article,Research Support, N.I.H., Extramural|http://www.ncbi.nlm.nih.gov/entrez/query.fcgi?cmd=Retrieve&db=pubmed&dopt=Abstract&list_uids=24333506|6|Exp Cell Res|321|2|Feb|2014|Journal Article,Research Support, N.I.H., Extramural,Research Support, Non-U.S. Gov't|http://www.ncbi.nlm.nih.gov/entrez/query.fcgi?cmd=Retrieve&db=pubmed&dopt=Abstract&list_uids=24903274|6|FASEB J|28|9|Sep|2014|Journal Article,Research Support, Non-U.S. Gov't
rhoa	vegfa	http://www.ncbi.nlm.nih.gov/entrez/query.fcgi?cmd=Retrieve&db=pubmed&dopt=Abstract&list_uids=24903274|6|FASEB J|28|9|Sep|2014|Journal Article,Research Support, Non-U.S. Gov't
rac1	cdc42	http://www.ncbi.nlm.nih.gov/entrez/query.fcgi?cmd=Retrieve&db=pubmed&dopt=Abstract&list_uids=23615439|6|Mol Biol Cell|24|12|Jun|2013|Journal Article,Research Support, N.I.H., Extramural|http://www.ncbi.nlm.nih.gov/entrez/query.fcgi?cmd=Retrieve&db=pubmed&dopt=Abstract&list_uids=24788532|6|Oncol Rep|31|6|Jun|2014|Journal Article,Research Support, Non-U.S. Gov't|http://www.ncbi.nlm.nih.gov/entrez/query.fcgi?cmd=Retrieve&db=pubmed&dopt=Abstract&list_uids=24903274|6|FASEB J|28|9|Sep|2014|Journal Article,Research Support, Non-U.S. Gov't|http://www.ncbi.nlm.nih.gov/entrez/query.fcgi?cmd=Retrieve&db=pubmed&dopt=Abstract&list_uids=24632816|6|PLoS One|9|3||2014|Journal Article,Research Support, Non-U.S. Gov't|http://www.ncbi.nlm.nih.gov/entrez/query.fcgi?cmd=Retrieve&db=pubmed&dopt=Abstract&list_uids=23826261|6|PL

		oS One|8|6||2013|Journal Article,Research Support, Non-U.S. Gov't|http://www.ncbi.nlm.nih.gov/entrez/query.fcgi?cmd=Retrieve&db=pubmed&dopt=Abstract&list_uids=23292068|6|Int J Oncol|42|3|Mar|2013|Journal Article,Research Support, Non-U.S. Gov't
cdc42	vegfa	http://www.ncbi.nlm.nih.gov/entrez/query.fcgi?cmd=Retrieve&db=pubmed&dopt=Abstract&list_uids=24903274|6|FASEB J|28|9|Sep|2014|Journal Article,Research Support, Non-U.S. Gov't
ephb2	jun	http://www.ncbi.nlm.nih.gov/entrez/query.fcgi?cmd=Retrieve&db=pubmed&dopt=Abstract&list_uids=24894398|6|Invest Ophthalmol Vis Sci|55|7|Jul|2014|Journal Article,Research Support, Non-U.S. Gov't
ephb2	mapk8	http://www.ncbi.nlm.nih.gov/entrez/query.fcgi?cmd=Retrieve&db=pubmed&dopt=Abstract&list_uids=24894398|6|Invest Ophthalmol Vis Sci|55|7|Jul|2014|Journal Article,Research Support, Non-U.S. Gov't
ephb2	smad2	http://www.ncbi.nlm.nih.gov/entrez/query.fcgi?cmd=Retrieve&db=pubmed&dopt=Abstract&list_uids=24894398|6|Invest Ophthalmol Vis Sci|55|7|Jul|2014|Journal Article,Research Support, Non-U.S. Gov't|http://www.ncbi.nlm.nih.gov/entrez/query.fcgi?cmd=Retrieve&db=pubmed&dopt=Abstract&list_uids=24466271|6|PLoS One|9|1||2014|Journal Article,Research Support, N.I.H., Extramural,Research Support, Non-U.S. Gov't
mapk14	jun	http://www.ncbi.nlm.nih.gov/entrez/query.fcgi?cmd=Retrieve&db=pubmed&dopt=Abstract&list_uids=24894398|6|Invest Ophthalmol Vis Sci|55|7|Jul|2014|Journal Article,Research Support, Non-U.S. Gov't
mapk14	mapk8	http://www.ncbi.nlm.nih.gov/entrez/query.fcgi?cmd=Retrieve&db=pubmed&dopt=Abstract&list_uids=24894398|6|Invest Ophthalmol Vis Sci|55|7|Jul|2014|Journal Article,Research Support, Non-U.S. Gov't
mapk14	smad2	http://www.ncbi.nlm.nih.gov/entrez/query.fcgi?cmd=Retrieve&db=pubmed&dopt=Abstract&list_uids=24894398|6|Invest Ophthalmol Vis Sci|55|7|Jul|2014|Journal Article,Research Support, Non-U.S. Gov't
jun	smad2	http://www.ncbi.nlm.nih.gov/entrez/query.fcgi?cmd=Retrieve&db=pubmed&dopt=Abstract&list_uids=24894398|6|Invest Ophthalmol Vis Sci|55|7|Jul|2014|Journal Article,Research Support, Non-U.S. Gov't
mapk8	smad2	http://www.ncbi.nlm.nih.gov/entrez/query.fcgi?cmd=Retrieve&db=pubmed&dopt=Abstract&list_uids=24894398|6|Invest Ophthalmol Vis Sci|55|7|Jul|2014|Journal Article,Research Support, Non-U.S. Gov't
ephb2	actin	http://www.ncbi.nlm.nih.gov/entrez/query.fcgi?cmd=Retrieve&db=pubmed&dopt=Abstract&list_uids=24894398|6|Invest Ophthalmol Vis Sci|55|7|Jul|2014|Journal Article,Research Support, Non-U.S. Gov't
mapk14	actin	http://www.ncbi.nlm.nih.gov/entrez/query.fcgi?cmd=Retrieve&db=pubmed&dopt=Abstract&list_uids=24894398|6|Invest Ophthalmol Vis Sci|55|7|Jul|2014|Journal Article,Research Support, Non-U.S. Gov't
mapk8	actin	http://www.ncbi.nlm.nih.gov/entrez/query.fcgi?cmd=Retrieve&db=pubmed&dopt=Abstract&list_uids=24894398|6|Invest Ophthalmol Vis Sci|55|7|Jul|2014|Journal Article,Research Support, Non-U.S. Gov't
actin	jun	http://www.ncbi.nlm.nih.gov/entrez/query.fcgi?cmd=Retrieve&db=pubmed&dopt=Abstract&list_uids=24894398|6|Invest Ophthalmol Vis Sci|55|7|Jul|2014|Journal Article,Research Support, Non-U.S. Gov't
jak2	stat3	http://www.ncbi.nlm.nih.gov/entrez/query.fcgi?cmd=Retrieve&db=pubmed&dopt=Abstract&list_uids=24861878|6|Neuro Oncol|16|9|Sep|2014|Journal Article,Research Support, Non-U.S. Gov't
jak2	egfr	http://www.ncbi.nlm.nih.gov/entrez/query.fcgi?cmd=Retrieve&db=pubmed&dopt=Abstract&list_uids=24861878|6|Neuro Oncol|16|9|Sep|2014|Journal Article,Research Support, Non-U.S. Gov't
jak2	ptk2	http://www.ncbi.nlm.nih.gov/entrez/query.fcgi?cmd=Retrieve&db=pubmed&dopt=Abstract&list_uids=24861878|6|Neuro

		Oncol|16|9|Sep|2014|Journal Article,Research Support, Non-U.S. Gov't
stat3	egfr	http://www.ncbi.nlm.nih.gov/entrez/query.fcgi?cmd=Retrieve&db=pubmed&dopt=Abstract&list_uids=24861878|6|Neuro Oncol|16|9|Sep|2014|Journal Article,Research Support, Non-U.S. Gov't
stat3	akt1	http://www.ncbi.nlm.nih.gov/entrez/query.fcgi?cmd=Retrieve&db=pubmed&dopt=Abstract&list_uids=24861878|6|Neuro Oncol|16|9|Sep|2014|Journal Article,Research Support, Non-U.S. Gov't
egfr	ptk2	http://www.ncbi.nlm.nih.gov/entrez/query.fcgi?cmd=Retrieve&db=pubmed&dopt=Abstract&list_uids=24861878|6|Neuro Oncol|16|9|Sep|2014|Journal Article,Research Support, Non-U.S. Gov't|http://www.ncbi.nlm.nih.gov/entrez/query.fcgi?cmd=Retrieve&db=pubmed&dopt=Abstract&list_uids=24091658|6|Cell Death Dis|4|||2013|Journal Article,Research Support, Non-U.S. Gov't
mmrn1	sfrs16	http://www.ncbi.nlm.nih.gov/entrez/query.fcgi?cmd=Retrieve&db=pubmed&dopt=Abstract&list_uids=24859005|6|Nat Cell Biol|16|6|Jun|2014|Journal Article,Research Support, N.I.H., Extramural,Research Support, Non-U.S. Gov't,Video-Audio Media
mmrn1	phldb2	http://www.ncbi.nlm.nih.gov/entrez/query.fcgi?cmd=Retrieve&db=pubmed&dopt=Abstract&list_uids=24859005|6|Nat Cell Biol|16|6|Jun|2014|Journal Article,Research Support, N.I.H., Extramural,Research Support, Non-U.S. Gov't,Video-Audio Media
sfrs16	phldb2	http://www.ncbi.nlm.nih.gov/entrez/query.fcgi?cmd=Retrieve&db=pubmed&dopt=Abstract&list_uids=24859005|6|Nat Cell Biol|16|6|Jun|2014|Journal Article,Research Support, N.I.H., Extramural,Research Support, Non-U.S. Gov't,Video-Audio Media
sfrs16	fas	http://www.ncbi.nlm.nih.gov/entrez/query.fcgi?cmd=Retrieve&db=pubmed&dopt=Abstract&list_uids=24859005|6|Nat Cell Biol|16|6|Jun|2014|Journal Article,Research Support, N.I.H., Extramural,Research Support, Non-U.S. Gov't,Video-Audio Media
arhgef2	vav2	http://www.ncbi.nlm.nih.gov/entrez/query.fcgi?cmd=Retrieve&db=pubmed&dopt=Abstract&list_uids=23615439|6|Mol Biol Cell|24|12|Jun|2013|Journal Article,Research Support, N.I.H., Extramural|http://www.ncbi.nlm.nih.gov/entrez/query.fcgi?cmd=Retrieve&db=pubmed&dopt=Abstract&list_uids=24858039|6|Oncogene|34|15|Apr|2015|Journal Article,Research Support, N.I.H., Extramural,Research Support, Non-U.S. Gov't
ptk2	vav2	http://www.ncbi.nlm.nih.gov/entrez/query.fcgi?cmd=Retrieve&db=pubmed&dopt=Abstract&list_uids=24858039|6|Oncogene|34|15|Apr|2015|Journal Article,Research Support, N.I.H., Extramural,Research Support, Non-U.S. Gov't
vav2	rac1	http://www.ncbi.nlm.nih.gov/entrez/query.fcgi?cmd=Retrieve&db=pubmed&dopt=Abstract&list_uids=23615439|6|Mol Biol Cell|24|12|Jun|2013|Journal Article,Research Support, N.I.H., Extramural|http://www.ncbi.nlm.nih.gov/entrez/query.fcgi?cmd=Retrieve&db=pubmed&dopt=Abstract&list_uids=24858039|6|Oncogene|34|15|Apr|2015|Journal Article,Research Support, N.I.H., Extramural,Research Support, Non-U.S. Gov't
clathrin	clc	http://www.ncbi.nlm.nih.gov/entrez/query.fcgi?cmd=Retrieve&db=pubmed&dopt=Abstract&list_uids=24852344|6|Nat Commun|5|||2014|Journal Article,Research Support, N.I.H., Extramural
clathrin	actin	http://www.ncbi.nlm.nih.gov/entrez/query.fcgi?cmd=Retrieve&db=pubmed&dopt=Abstract&list_uids=24852344|6|Nat Commun|5|||2014|Journal Article,Research Support, N.I.H., Extramural
clc	actin	http://www.ncbi.nlm.nih.gov/entrez/query.fcgi?cmd=Retrieve&db=pubmed&dopt=Abstract&list_uids=24852344|6|Nat

		Commun|5|||2014|Journal Article,Research Support, N.I.H., Extramural
abcb1	clathrin	http://www.ncbi.nlm.nih.gov/entrez/query.fcgi?cmd=Retrieve&db=pubmed&dopt=Abstract&list_uids=24852344|6|Nat Commun|5|||2014|Journal Article,Research Support, N.I.H., Extramural
ptk2	nf2	http://www.ncbi.nlm.nih.gov/entrez/query.fcgi?cmd=Retrieve&db=pubmed&dopt=Abstract&list_uids=24848258|6|Sci Transl Med|6|237|May|2014|Journal Article,Research Support, N.I.H., Extramural,Research Support, Non-U.S. Gov't
sema3d	sema3e	http://www.ncbi.nlm.nih.gov/entrez/query.fcgi?cmd=Retrieve&db=pubmed&dopt=Abstract&list_uids=24825896|6|J Biol Chem|289|26|Jun|2014|Journal Article,Research Support, N.I.H., Extramural,Research Support, Non-U.S. Gov't
nrp1	sema3d	http://www.ncbi.nlm.nih.gov/entrez/query.fcgi?cmd=Retrieve&db=pubmed&dopt=Abstract&list_uids=24825896|6|J Biol Chem|289|26|Jun|2014|Journal Article,Research Support, N.I.H., Extramural,Research Support, Non-U.S. Gov't
nrp1	sema3e	http://www.ncbi.nlm.nih.gov/entrez/query.fcgi?cmd=Retrieve&db=pubmed&dopt=Abstract&list_uids=24825896|6|J Biol Chem|289|26|Jun|2014|Journal Article,Research Support, N.I.H., Extramural,Research Support, Non-U.S. Gov't
plxnd1	sema3e	http://www.ncbi.nlm.nih.gov/entrez/query.fcgi?cmd=Retrieve&db=pubmed&dopt=Abstract&list_uids=24825896|6|J Biol Chem|289|26|Jun|2014|Journal Article,Research Support, N.I.H., Extramural,Research Support, Non-U.S. Gov't
plxnd1	sema3d	http://www.ncbi.nlm.nih.gov/entrez/query.fcgi?cmd=Retrieve&db=pubmed&dopt=Abstract&list_uids=24825896|6|J Biol Chem|289|26|Jun|2014|Journal Article,Research Support, N.I.H., Extramural,Research Support, Non-U.S. Gov't
sema3e	akt1	http://www.ncbi.nlm.nih.gov/entrez/query.fcgi?cmd=Retrieve&db=pubmed&dopt=Abstract&list_uids=24825896|6|J Biol Chem|289|26|Jun|2014|Journal Article,Research Support, N.I.H., Extramural,Research Support, Non-U.S. Gov't
sema3e	pik3ca	http://www.ncbi.nlm.nih.gov/entrez/query.fcgi?cmd=Retrieve&db=pubmed&dopt=Abstract&list_uids=24825896|6|J Biol Chem|289|26|Jun|2014|Journal Article,Research Support, N.I.H., Extramural,Research Support, Non-U.S. Gov't
sema3d	akt1	http://www.ncbi.nlm.nih.gov/entrez/query.fcgi?cmd=Retrieve&db=pubmed&dopt=Abstract&list_uids=24825896|6|J Biol Chem|289|26|Jun|2014|Journal Article,Research Support, N.I.H., Extramural,Research Support, Non-U.S. Gov't
sema3d	pik3ca	http://www.ncbi.nlm.nih.gov/entrez/query.fcgi?cmd=Retrieve&db=pubmed&dopt=Abstract&list_uids=24825896|6|J Biol Chem|289|26|Jun|2014|Journal Article,Research Support, N.I.H., Extramural,Research Support, Non-U.S. Gov't
fcn2	cdk5	http://www.ncbi.nlm.nih.gov/entrez/query.fcgi?cmd=Retrieve&db=pubmed&dopt=Abstract&list_uids=24793790|6|Oncogene|34|14|Apr|2015|Journal Article,Research Support, N.I.H., Extramural,Research Support, Non-U.S. Gov't,Research Support, U.S. Gov't, Non-P.H.S.
cdk5	axpc1	http://www.ncbi.nlm.nih.gov/entrez/query.fcgi?cmd=Retrieve&db=pubmed&dopt=Abstract&list_uids=24793790|6|Oncogene|34|14|Apr|2015|Journal Article,Research Support, N.I.H., Extramural,Research Support, Non-U.S. Gov't,Research Support, U.S. Gov't, Non-P.H.S.
rac1	asap1	http://www.ncbi.nlm.nih.gov/entrez/query.fcgi?cmd=Retrieve&db=pubmed&dopt=Abstract&list_uids=24788532|6|Oncol Rep|31|6|Jun|2014|Journal Article,Research Support, Non-U.S. Gov't
cdc42	asap1	http://www.ncbi.nlm.nih.gov/entrez/query.fcgi?cmd=Retrieve&db=pubmed&dopt=Abstract&list_uids=24788532|6|Oncol Rep|31|6|Jun|2014|Journal Article,Research Support, Non-U.S. Gov't
psmc6	arhgef2	http://www.ncbi.nlm.nih.gov/entrez/query.fcgi?cmd=Retrieve&db=pubmed&dopt=Abstract&list_uids=24706358|6|FASEB J|28|7|Jul|2014|Journal Article,Research Support, N.I.H., Extramural

capns1	mmp2	http://www.ncbi.nlm.nih.gov/entrez/query.fcgi?cmd=Retrieve&db=pubmed&dopt=Abstract&list_uids=24703594|6|Cancer Sci|105|6|Jun|2014|Journal Article,Research Support, Non-U.S. Gov't
capns1	rela	http://www.ncbi.nlm.nih.gov/entrez/query.fcgi?cmd=Retrieve&db=pubmed&dopt=Abstract&list_uids=24703594|6|Cancer Sci|105|6|Jun|2014|Journal Article,Research Support, Non-U.S. Gov't
mmp2	rela	http://www.ncbi.nlm.nih.gov/entrez/query.fcgi?cmd=Retrieve&db=pubmed&dopt=Abstract&list_uids=24703594|6|Cancer Sci|105|6|Jun|2014|Journal Article,Research Support, Non-U.S. Gov't
myl2	pf4	http://www.ncbi.nlm.nih.gov/entrez/query.fcgi?cmd=Retrieve&db=pubmed&dopt=Abstract&list_uids=24699667|6|PLoS One|9|4||2014|Journal Article,Research Support, Non-U.S. Gov't
myl2	vegfa	http://www.ncbi.nlm.nih.gov/entrez/query.fcgi?cmd=Retrieve&db=pubmed&dopt=Abstract&list_uids=24699667|6|PLoS One|9|4||2014|Journal Article,Research Support, Non-U.S. Gov't
pf4	vegfa	http://www.ncbi.nlm.nih.gov/entrez/query.fcgi?cmd=Retrieve&db=pubmed&dopt=Abstract&list_uids=24699667|6|PLoS One|9|4||2014|Journal Article,Research Support, Non-U.S. Gov't
pf4	kdr	http://www.ncbi.nlm.nih.gov/entrez/query.fcgi?cmd=Retrieve&db=pubmed&dopt=Abstract&list_uids=24699667|6|PLoS One|9|4||2014|Journal Article,Research Support, Non-U.S. Gov't
plagl2	dgcr14	http://www.ncbi.nlm.nih.gov/entrez/query.fcgi?cmd=Retrieve&db=pubmed&dopt=Abstract&list_uids=24675530|6|Carcinogenesis|35|9|Sep|2014|Journal Article,Research Support, Non-U.S. Gov't
plagl2	rhoa	http://www.ncbi.nlm.nih.gov/entrez/query.fcgi?cmd=Retrieve&db=pubmed&dopt=Abstract&list_uids=24675530|6|Carcinogenesis|35|9|Sep|2014|Journal Article,Research Support, Non-U.S. Gov't
plagl2	rac1	http://www.ncbi.nlm.nih.gov/entrez/query.fcgi?cmd=Retrieve&db=pubmed&dopt=Abstract&list_uids=24675530|6|Carcinogenesis|35|9|Sep|2014|Journal Article,Research Support, Non-U.S. Gov't
dgcr14	rhoa	http://www.ncbi.nlm.nih.gov/entrez/query.fcgi?cmd=Retrieve&db=pubmed&dopt=Abstract&list_uids=24675530|6|Carcinogenesis|35|9|Sep|2014|Journal Article,Research Support, Non-U.S. Gov't
dgcr14	rac1	http://www.ncbi.nlm.nih.gov/entrez/query.fcgi?cmd=Retrieve&db=pubmed&dopt=Abstract&list_uids=24675530|6|Carcinogenesis|35|9|Sep|2014|Journal Article,Research Support, Non-U.S. Gov't
actin	plagl2	http://www.ncbi.nlm.nih.gov/entrez/query.fcgi?cmd=Retrieve&db=pubmed&dopt=Abstract&list_uids=24675530|6|Carcinogenesis|35|9|Sep|2014|Journal Article,Research Support, Non-U.S. Gov't
chn1	rac1	http://www.ncbi.nlm.nih.gov/entrez/query.fcgi?cmd=Retrieve&db=pubmed&dopt=Abstract&list_uids=24675530|6|Carcinogenesis|35|9|Sep|2014|Journal Article,Research Support, Non-U.S. Gov't
chn1	plagl2	http://www.ncbi.nlm.nih.gov/entrez/query.fcgi?cmd=Retrieve&db=pubmed&dopt=Abstract&list_uids=24675530|6|Carcinogenesis|35|9|Sep|2014|Journal Article,Research Support, Non-U.S. Gov't
fyn	akt1	http://www.ncbi.nlm.nih.gov/entrez/query.fcgi?cmd=Retrieve&db=pubmed&dopt=Abstract&list_uids=24670416|6|PLoS One|9|3||2014|Journal Article,Research Support, Non-U.S. Gov't|http://www.ncbi.nlm.nih.gov/entrez/query.fcgi?cmd=Retrieve&db=pubmed&dopt=Abstract&list_uids=23836527|6|Stem Cells|31|11|Nov|2013|Journal Article,Research Support, N.I.H., Extramural
fyn	mapk1	http://www.ncbi.nlm.nih.gov/entrez/query.fcgi?cmd=Retrieve&db=pubmed&dopt=Abstract&list_uids=24670416|6|PLoS One|9|3||2014|Journal Article,Research Support, Non-U.S. Gov't

fyn	hdac1	http://www.ncbi.nlm.nih.gov/entrez/query.fcgi?cmd=Retrieve&db=pubmed&dopt=Abstract&list_uids=24670416|6|PLoS One|9|3||2014|Journal Article,Research Support, Non-U.S. Gov't
akt1	mapk1	http://www.ncbi.nlm.nih.gov/entrez/query.fcgi?cmd=Retrieve&db=pubmed&dopt=Abstract&list_uids=24670416|6|PLoS One|9|3||2014|Journal Article,Research Support, Non-U.S. Gov't
akt1	hdac1	http://www.ncbi.nlm.nih.gov/entrez/query.fcgi?cmd=Retrieve&db=pubmed&dopt=Abstract&list_uids=24670416|6|PLoS One|9|3||2014|Journal Article,Research Support, Non-U.S. Gov't
mapk1	hdac1	http://www.ncbi.nlm.nih.gov/entrez/query.fcgi?cmd=Retrieve&db=pubmed&dopt=Abstract&list_uids=24670416|6|PLoS One|9|3||2014|Journal Article,Research Support, Non-U.S. Gov't
fas	bcar1	http://www.ncbi.nlm.nih.gov/entrez/query.fcgi?cmd=Retrieve&db=pubmed&dopt=Abstract&list_uids=24642576|6|PLoS One|9|3||2014|Journal Article,Research Support, Non-U.S. Gov't|http://www.ncbi.nlm.nih.gov/entrez/query.fcgi?cmd=Retrieve&db=pubmed&dopt=Abstract&list_uids=24497348|6|Cell Biol Int|38|3|Mar|2014|Journal Article,Research Support, Non-U.S. Gov't,Review
rnf43	znrf3	http://www.ncbi.nlm.nih.gov/entrez/query.fcgi?cmd=Retrieve&db=pubmed&dopt=Abstract&list_uids=24639526|6|Proc Natl Acad Sci U S A|111|13|Apr|2014|Journal Article,Research Support, N.I.H., Extramural,Research Support, Non-U.S. Gov't
iqgap1	lgr4	http://www.ncbi.nlm.nih.gov/entrez/query.fcgi?cmd=Retrieve&db=pubmed&dopt=Abstract&list_uids=24639526|6|Proc Natl Acad Sci U S A|111|13|Apr|2014|Journal Article,Research Support, N.I.H., Extramural,Research Support, Non-U.S. Gov't
iqgap1	sgsm3	http://www.ncbi.nlm.nih.gov/entrez/query.fcgi?cmd=Retrieve&db=pubmed&dopt=Abstract&list_uids=24639526|6|Proc Natl Acad Sci U S A|111|13|Apr|2014|Journal Article,Research Support, N.I.H., Extramural,Research Support, Non-U.S. Gov't
map2k1	lrp5	http://www.ncbi.nlm.nih.gov/entrez/query.fcgi?cmd=Retrieve&db=pubmed&dopt=Abstract&list_uids=24639526|6|Proc Natl Acad Sci U S A|111|13|Apr|2014|Journal Article,Research Support, N.I.H., Extramural,Research Support, Non-U.S. Gov't
map2k1	actin	http://www.ncbi.nlm.nih.gov/entrez/query.fcgi?cmd=Retrieve&db=pubmed&dopt=Abstract&list_uids=24639526|6|Proc Natl Acad Sci U S A|111|13|Apr|2014|Journal Article,Research Support, N.I.H., Extramural,Research Support, Non-U.S. Gov't
lrp5	actin	http://www.ncbi.nlm.nih.gov/entrez/query.fcgi?cmd=Retrieve&db=pubmed&dopt=Abstract&list_uids=24639526|6|Proc Natl Acad Sci U S A|111|13|Apr|2014|Journal Article,Research Support, N.I.H., Extramural,Research Support, Non-U.S. Gov't
timp1	cd63	http://www.ncbi.nlm.nih.gov/entrez/query.fcgi?cmd=Retrieve&db=pubmed&dopt=Abstract&list_uids=24635319|6|Biochem J|459|3|May|2014|Journal Article,Research Support, Non-U.S. Gov't
timp1	akt1	http://www.ncbi.nlm.nih.gov/entrez/query.fcgi?cmd=Retrieve&db=pubmed&dopt=Abstract&list_uids=24635319|6|Biochem J|459|3|May|2014|Journal Article,Research Support, Non-U.S. Gov't
timp1	ptk2	http://www.ncbi.nlm.nih.gov/entrez/query.fcgi?cmd=Retrieve&db=pubmed&dopt=Abstract&list_uids=24635319|6|Biochem J|459|3|May|2014|Journal Article,Research Support, Non-U.S. Gov't
timp1	actin	http://www.ncbi.nlm.nih.gov/entrez/query.fcgi?cmd=Retrieve&db=pubmed&dopt=Abstract&list_uids=24635319|6|Biochem J|459|3|May|2014|Journal Article,Research Support, Non-U.S. Gov't
cd63	akt1	http://www.ncbi.nlm.nih.gov/entrez/query.fcgi?cmd=Retrieve&db=pubmed&dopt=Abstract&list_uids=24635319|6|Biochem J|459|3|May|2014|Journal Article,Research Support, Non-U.S. Gov't
cd63	ptk2	http://www.ncbi.nlm.nih.gov/entrez/query.fcgi?cmd=Retrieve&db=pubmed&dopt=Abstract&list_uids=24635319|6|Biochem J|459|3|May|2014|Journal Article,Research Support, Non-U.S. Gov't

cd63	actin	http://www.ncbi.nlm.nih.gov/entrez/query.fcgi?cmd=Retrieve&db=pubmed&dopt=Abstract&list_uids=24635319|6|Biochem J|459|3|May|2014|Journal Article,Research Support, Non-U.S. Gov't
pik3ca	timp1	http://www.ncbi.nlm.nih.gov/entrez/query.fcgi?cmd=Retrieve&db=pubmed&dopt=Abstract&list_uids=24635319|6|Biochem J|459|3|May|2014|Journal Article,Research Support, Non-U.S. Gov't
cdgap	rac1	http://www.ncbi.nlm.nih.gov/entrez/query.fcgi?cmd=Retrieve&db=pubmed&dopt=Abstract&list_uids=24632816|6|PLoS One|9|3||2014|Journal Article,Research Support, Non-U.S. Gov't
cdgap	cdc42	http://www.ncbi.nlm.nih.gov/entrez/query.fcgi?cmd=Retrieve&db=pubmed&dopt=Abstract&list_uids=24632816|6|PLoS One|9|3||2014|Journal Article,Research Support, Non-U.S. Gov't
tmeff2	rhoa	http://www.ncbi.nlm.nih.gov/entrez/query.fcgi?cmd=Retrieve&db=pubmed&dopt=Abstract&list_uids=24632071|6|Biochim Biophys Acta|1843|6|Jun|2014|Journal Article,Research Support, N.I.H., Extramural
3.6.4.1	rock2	http://www.ncbi.nlm.nih.gov/entrez/query.fcgi?cmd=Retrieve&db=pubmed&dopt=Abstract&list_uids=24610576|6|J Cell Biochem|115|9|Sep|2014|Journal Article,Research Support, Non-U.S. Gov't
ptger2	mlc1	http://www.ncbi.nlm.nih.gov/entrez/query.fcgi?cmd=Retrieve&db=pubmed&dopt=Abstract&list_uids=24610576|6|J Cell Biochem|115|9|Sep|2014|Journal Article,Research Support, Non-U.S. Gov't
fzd4	rock2	http://www.ncbi.nlm.nih.gov/entrez/query.fcgi?cmd=Retrieve&db=pubmed&dopt=Abstract&list_uids=24610576|6|J Cell Biochem|115|9|Sep|2014|Journal Article,Research Support, Non-U.S. Gov't
rock2	mlc1	http://www.ncbi.nlm.nih.gov/entrez/query.fcgi?cmd=Retrieve&db=pubmed&dopt=Abstract&list_uids=24610576|6|J Cell Biochem|115|9|Sep|2014|Journal Article,Research Support, Non-U.S. Gov't
ptk2	kcnk2	http://www.ncbi.nlm.nih.gov/entrez/query.fcgi?cmd=Retrieve&db=pubmed&dopt=Abstract&list_uids=24586773|6|PLoS One|9|2||2014|Journal Article,Research Support, N.I.H., Extramural,Research Support, Non-U.S. Gov't
tyr	kcnk2	http://www.ncbi.nlm.nih.gov/entrez/query.fcgi?cmd=Retrieve&db=pubmed&dopt=Abstract&list_uids=24586773|6|PLoS One|9|2||2014|Journal Article,Research Support, N.I.H., Extramural,Research Support, Non-U.S. Gov't
inppl1	tyr	http://www.ncbi.nlm.nih.gov/entrez/query.fcgi?cmd=Retrieve&db=pubmed&dopt=Abstract&list_uids=24561238|6|Cell Signal|26|6|Jun|2014|Journal Article,Research Support, Non-U.S. Gov't
arf5	arf6	http://www.ncbi.nlm.nih.gov/entrez/query.fcgi?cmd=Retrieve&db=pubmed&dopt=Abstract&list_uids=24522833|6|Basic Res Cardiol|109|2|Mar|2014|Journal Article,Research Support, N.I.H., Intramural,Research Support, Non-U.S. Gov't
egf	actin	http://www.ncbi.nlm.nih.gov/entrez/query.fcgi?cmd=Retrieve&db=pubmed&dopt=Abstract&list_uids=24505297|6|PLoS One|9|2||2014|Journal Article,Research Support, Non-U.S. Gov't|http://www.ncbi.nlm.nih.gov/entrez/query.fcgi?cmd=Retrieve&db=pubmed&dopt=Abstract&list_uids=23023326|6|Int J Oncol|41|6|Dec|2012|Journal Article,Research Support, Non-U.S. Gov't|http://www.ncbi.nlm.nih.gov/entrez/query.fcgi?cmd=Retrieve&db=pubmed&dopt=Abstract&list_uids=22357970|6|J Cell Sci|125|Pt 2|Jan|2012|Journal Article,Research Support, Non-U.S. Gov't
egfr	actin	http://www.ncbi.nlm.nih.gov/entrez/query.fcgi?cmd=Retrieve&db=pubmed&dopt=Abstract&list_uids=24505297|6|PLoS One|9|2||2014|Journal Article,Research Support, Non-U.S. Gov't
igfbp3	thbs1	http://www.ncbi.nlm.nih.gov/entrez/query.fcgi?cmd=Retrieve&db=pubmed&dopt=Abstract&list_uids=24497940|6|PLoS One|9|1||2014|Journal Article,Research Support, N.I.H., Extramural

duoxa1	bcar1	http://www.ncbi.nlm.nih.gov/entrez/query.fcgi?cmd=Retrieve&db=pubmed&dopt=Abstract&list_uids=24497348|6|Cell Biol Int|38|3|Mar|2014|Journal Article,Research Support, Non-U.S. Gov't,Review
duoxa1	fas	http://www.ncbi.nlm.nih.gov/entrez/query.fcgi?cmd=Retrieve&db=pubmed&dopt=Abstract&list_uids=24497348|6|Cell Biol Int|38|3|Mar|2014|Journal Article,Research Support, Non-U.S. Gov't,Review
map3k2	mapk7	http://www.ncbi.nlm.nih.gov/entrez/query.fcgi?cmd=Retrieve&db=pubmed&dopt=Abstract&list_uids=24491810|6|Biochim Biophys Acta|1843|5|May|2014|Journal Article,Research Support, N.I.H., Extramural,Research Support, Non-U.S. Gov't
map3k2	ptk2	http://www.ncbi.nlm.nih.gov/entrez/query.fcgi?cmd=Retrieve&db=pubmed&dopt=Abstract&list_uids=24491810|6|Biochim Biophys Acta|1843|5|May|2014|Journal Article,Research Support, N.I.H., Extramural,Research Support, Non-U.S. Gov't
mapk7	ptk2	http://www.ncbi.nlm.nih.gov/entrez/query.fcgi?cmd=Retrieve&db=pubmed&dopt=Abstract&list_uids=24491810|6|Biochim Biophys Acta|1843|5|May|2014|Journal Article,Research Support, N.I.H., Extramural,Research Support, Non-U.S. Gov't
ola1	ptk2	http://www.ncbi.nlm.nih.gov/entrez/query.fcgi?cmd=Retrieve&db=pubmed&dopt=Abstract&list_uids=24486488|6|Biochem Biophys Res Commun|444|4|Feb|2014|Journal Article,Research Support, N.I.H., Extramural,Research Support, Non-U.S. Gov't
ola1	actin	http://www.ncbi.nlm.nih.gov/entrez/query.fcgi?cmd=Retrieve&db=pubmed&dopt=Abstract&list_uids=24486488|6|Biochem Biophys Res Commun|444|4|Feb|2014|Journal Article,Research Support, N.I.H., Extramural,Research Support, Non-U.S. Gov't
cdc42bpa	tnfsf13	http://www.ncbi.nlm.nih.gov/entrez/query.fcgi?cmd=Retrieve&db=pubmed&dopt=Abstract&list_uids=24486483|6|Eur J Pharm Sci|55||May|2014|Journal Article,Research Support, Non-U.S. Gov't
dag1	pkc	http://www.ncbi.nlm.nih.gov/entrez/query.fcgi?cmd=Retrieve&db=pubmed&dopt=Abstract&list_uids=24486483|6|Eur J Pharm Sci|55||May|2014|Journal Article,Research Support, Non-U.S. Gov't
prkd1	cdc42	http://www.ncbi.nlm.nih.gov/entrez/query.fcgi?cmd=Retrieve&db=pubmed&dopt=Abstract&list_uids=24486483|6|Eur J Pharm Sci|55||May|2014|Journal Article,Research Support, Non-U.S. Gov't
prkd1	cdc42bpa	http://www.ncbi.nlm.nih.gov/entrez/query.fcgi?cmd=Retrieve&db=pubmed&dopt=Abstract&list_uids=24486483|6|Eur J Pharm Sci|55||May|2014|Journal Article,Research Support, Non-U.S. Gov't
cdc42	cdc42bpa	http://www.ncbi.nlm.nih.gov/entrez/query.fcgi?cmd=Retrieve&db=pubmed&dopt=Abstract&list_uids=24486483|6|Eur J Pharm Sci|55||May|2014|Journal Article,Research Support, Non-U.S. Gov't
src	micall1	http://www.ncbi.nlm.nih.gov/entrez/query.fcgi?cmd=Retrieve&db=pubmed&dopt=Abstract&list_uids=24481818|6|J Cell Sci|127|Pt 8|Apr|2014|Journal Article,Research Support, N.I.H., Extramural,Research Support, Non-U.S. Gov't
src	ehd1	http://www.ncbi.nlm.nih.gov/entrez/query.fcgi?cmd=Retrieve&db=pubmed&dopt=Abstract&list_uids=24481818|6|J Cell Sci|127|Pt 8|Apr|2014|Journal Article,Research Support, N.I.H., Extramural,Research Support, Non-U.S. Gov't
micall1	ehd1	http://www.ncbi.nlm.nih.gov/entrez/query.fcgi?cmd=Retrieve&db=pubmed&dopt=Abstract&list_uids=24481818|6|J Cell Sci|127|Pt 8|Apr|2014|Journal Article,Research Support, N.I.H., Extramural,Research Support, Non-U.S. Gov't
fbln7	actin	http://www.ncbi.nlm.nih.gov/entrez/query.fcgi?cmd=Retrieve&db=pubmed&dopt=Abstract&list_uids=24480309|6|Arch Biochem Biophys|545||Mar|2014|Journal Article,Research Support, Non-U.S. Gov't
rhoa	irs1	http://www.ncbi.nlm.nih.gov/entrez/query.fcgi?cmd=Retrieve&db=pubmed&dopt=Abstract&list_uids=24470116|6|Cell Mol Biol Lett|19|1|Mar|2014|Journal Article,Research Support, Non-U.S. Gov't

smad2	sgsm3	http://www.ncbi.nlm.nih.gov/entrez/query.fcgi?cmd=Retrieve&db=pubmed&dopt=Abstract&list_uids=24466271|6|PLoS One|9|1||2014|Journal Article,Research Support, N.I.H., Extramural,Research Support, Non-U.S. Gov't
smad2	fgf2	http://www.ncbi.nlm.nih.gov/entrez/query.fcgi?cmd=Retrieve&db=pubmed&dopt=Abstract&list_uids=24466271|6|PLoS One|9|1||2014|Journal Article,Research Support, N.I.H., Extramural,Research Support, Non-U.S. Gov't
sgsm3	fgf2	http://www.ncbi.nlm.nih.gov/entrez/query.fcgi?cmd=Retrieve&db=pubmed&dopt=Abstract&list_uids=24466271|6|PLoS One|9|1||2014|Journal Article,Research Support, N.I.H., Extramural,Research Support, Non-U.S. Gov't
ilk	pkb	http://www.ncbi.nlm.nih.gov/entrez/query.fcgi?cmd=Retrieve&db=pubmed&dopt=Abstract&list_uids=24466237|6|PLoS One|9|1||2014|Journal Article,Research Support, Non-U.S. Gov't
ilk	akt1	http://www.ncbi.nlm.nih.gov/entrez/query.fcgi?cmd=Retrieve&db=pubmed&dopt=Abstract&list_uids=24466237|6|PLoS One|9|1||2014|Journal Article,Research Support, Non-U.S. Gov't|http://www.ncbi.nlm.nih.gov/entrez/query.fcgi?cmd=Retrieve&db=pubmed&dopt=Abstract&list_uids=22767580|6|Mol Biol Cell|23|17|Sep|2012|Journal Article,Research Support, Non-U.S. Gov't
ilk	mmp9	http://www.ncbi.nlm.nih.gov/entrez/query.fcgi?cmd=Retrieve&db=pubmed&dopt=Abstract&list_uids=24466237|6|PLoS One|9|1||2014|Journal Article,Research Support, Non-U.S. Gov't
pkb	akt1	http://www.ncbi.nlm.nih.gov/entrez/query.fcgi?cmd=Retrieve&db=pubmed&dopt=Abstract&list_uids=24466237|6|PLoS One|9|1||2014|Journal Article,Research Support, Non-U.S. Gov't
pkb	mmp9	http://www.ncbi.nlm.nih.gov/entrez/query.fcgi?cmd=Retrieve&db=pubmed&dopt=Abstract&list_uids=24466237|6|PLoS One|9|1||2014|Journal Article,Research Support, Non-U.S. Gov't
akt1	mmp9	http://www.ncbi.nlm.nih.gov/entrez/query.fcgi?cmd=Retrieve&db=pubmed&dopt=Abstract&list_uids=24466237|6|PLoS One|9|1||2014|Journal Article,Research Support, Non-U.S. Gov't
mmp9	fos	http://www.ncbi.nlm.nih.gov/entrez/query.fcgi?cmd=Retrieve&db=pubmed&dopt=Abstract&list_uids=24466237|6|PLoS One|9|1||2014|Journal Article,Research Support, Non-U.S. Gov't
mapk3	akt1	http://www.ncbi.nlm.nih.gov/entrez/query.fcgi?cmd=Retrieve&db=pubmed&dopt=Abstract&list_uids=24463198|6|Chem Biol Interact|211||Mar|2014|Journal Article,Review|http://www.ncbi.nlm.nih.gov/entrez/query.fcgi?cmd=Retrieve&db=pubmed&dopt=Abstract&list_uids=23562553|6|Biochim Biophys Acta|1830|8|Aug|2013|Journal Article,Research Support, Non-U.S. Gov't
camk2g	actin	http://www.ncbi.nlm.nih.gov/entrez/query.fcgi?cmd=Retrieve&db=pubmed&dopt=Abstract&list_uids=24463198|6|Chem Biol Interact|211||Mar|2014|Journal Article,Review
camk2g	ptk2	http://www.ncbi.nlm.nih.gov/entrez/query.fcgi?cmd=Retrieve&db=pubmed&dopt=Abstract&list_uids=24463198|6|Chem Biol Interact|211||Mar|2014|Journal Article,Review
actin	capza1	http://www.ncbi.nlm.nih.gov/entrez/query.fcgi?cmd=Retrieve&db=pubmed&dopt=Abstract&list_uids=24430196|6|Mol Biosyst|10|3|Mar|2014|Journal Article,Research Support, Non-U.S. Gov't
smarcb1	ilf3	http://www.ncbi.nlm.nih.gov/entrez/query.fcgi?cmd=Retrieve&db=pubmed&dopt=Abstract&list_uids=24430196|6|Mol Biosyst|10|3|Mar|2014|Journal Article,Research Support, Non-U.S. Gov't
smarcb1	dpy30	http://www.ncbi.nlm.nih.gov/entrez/query.fcgi?cmd=Retrieve&db=pubmed&dopt=Abstract&list_uids=24430196|6|Mol Biosyst|10|3|Mar|2014|Journal Article,Research Support, Non-U.S. Gov't

ilf3	dpy30	http://www.ncbi.nlm.nih.gov/entrez/query.fcgi?cmd=Retrieve&db=pubmed&dopt=Abstract&list_uids=24430196|6|Mol Biosyst|10|3|Mar|2014|Journal Article,Research Support, Non-U.S. Gov't
grlf1	cntnap1	http://www.ncbi.nlm.nih.gov/entrez/query.fcgi?cmd=Retrieve&db=pubmed&dopt=Abstract&list_uids=24403595|6|J Virol|88|7|Apr|2014|Journal Article,Research Support, Non-U.S. Gov't
grlf1	rasa1	http://www.ncbi.nlm.nih.gov/entrez/query.fcgi?cmd=Retrieve&db=pubmed&dopt=Abstract&list_uids=24403595|6|J Virol|88|7|Apr|2014|Journal Article,Research Support, Non-U.S. Gov't
cntnap1	rasa1	http://www.ncbi.nlm.nih.gov/entrez/query.fcgi?cmd=Retrieve&db=pubmed&dopt=Abstract&list_uids=24403595|6|J Virol|88|7|Apr|2014|Journal Article,Research Support, Non-U.S. Gov't
cntnap1	rhoa	http://www.ncbi.nlm.nih.gov/entrez/query.fcgi?cmd=Retrieve&db=pubmed&dopt=Abstract&list_uids=24403595|6|J Virol|88|7|Apr|2014|Journal Article,Research Support, Non-U.S. Gov't
rasa1	rhoa	http://www.ncbi.nlm.nih.gov/entrez/query.fcgi?cmd=Retrieve&db=pubmed&dopt=Abstract&list_uids=24403595|6|J Virol|88|7|Apr|2014|Journal Article,Research Support, Non-U.S. Gov't|http://www.ncbi.nlm.nih.gov/entrez/query.fcgi?cmd=Retrieve&db=pubmed&dopt=Abstract&list_uids=24333506|6|Exp Cell Res|321|2|Feb|2014|Journal Article,Research Support, N.I.H., Extramural,Research Support, Non-U.S. Gov't
git1	egf	http://www.ncbi.nlm.nih.gov/entrez/query.fcgi?cmd=Retrieve&db=pubmed&dopt=Abstract&list_uids=24335959|6|Cancer Res|74|3|Feb|2014|Journal Article,Research Support, Non-U.S. Gov't
git1	egfr	http://www.ncbi.nlm.nih.gov/entrez/query.fcgi?cmd=Retrieve&db=pubmed&dopt=Abstract&list_uids=24335959|6|Cancer Res|74|3|Feb|2014|Journal Article,Research Support, Non-U.S. Gov't
git1	mapk3	http://www.ncbi.nlm.nih.gov/entrez/query.fcgi?cmd=Retrieve&db=pubmed&dopt=Abstract&list_uids=24335959|6|Cancer Res|74|3|Feb|2014|Journal Article,Research Support, Non-U.S. Gov't
git1	mmp2	http://www.ncbi.nlm.nih.gov/entrez/query.fcgi?cmd=Retrieve&db=pubmed&dopt=Abstract&list_uids=24335959|6|Cancer Res|74|3|Feb|2014|Journal Article,Research Support, Non-U.S. Gov't
egf	mmp2	http://www.ncbi.nlm.nih.gov/entrez/query.fcgi?cmd=Retrieve&db=pubmed&dopt=Abstract&list_uids=24335959|6|Cancer Res|74|3|Feb|2014|Journal Article,Research Support, Non-U.S. Gov't
egfr	mapk3	http://www.ncbi.nlm.nih.gov/entrez/query.fcgi?cmd=Retrieve&db=pubmed&dopt=Abstract&list_uids=24335959|6|Cancer Res|74|3|Feb|2014|Journal Article,Research Support, Non-U.S. Gov't
egfr	mmp2	http://www.ncbi.nlm.nih.gov/entrez/query.fcgi?cmd=Retrieve&db=pubmed&dopt=Abstract&list_uids=24335959|6|Cancer Res|74|3|Feb|2014|Journal Article,Research Support, Non-U.S. Gov't
mapk3	mmp2	http://www.ncbi.nlm.nih.gov/entrez/query.fcgi?cmd=Retrieve&db=pubmed&dopt=Abstract&list_uids=24335959|6|Cancer Res|74|3|Feb|2014|Journal Article,Research Support, Non-U.S. Gov't
stard13	rasa1	http://www.ncbi.nlm.nih.gov/entrez/query.fcgi?cmd=Retrieve&db=pubmed&dopt=Abstract&list_uids=24333506|6|Exp Cell Res|321|2|Feb|2014|Journal Article,Research Support, N.I.H., Extramural,Research Support, Non-U.S. Gov't
stard13	rhoa	http://www.ncbi.nlm.nih.gov/entrez/query.fcgi?cmd=Retrieve&db=pubmed&dopt=Abstract&list_uids=24333506|6|Exp Cell Res|321|2|Feb|2014|Journal Article,Research Support, N.I.H., Extramural,Research Support, Non-U.S. Gov't
stard13	cdc42	http://www.ncbi.nlm.nih.gov/entrez/query.fcgi?cmd=Retrieve&db=pubmed&dopt=Abstract&list_uids=24333506|6|Exp Cell Res|321|2|Feb|2014|Journal Article,Research Support, N.I.H., Extramural,Research Support, Non-U.S. Gov't

rasa1	cdc42	http://www.ncbi.nlm.nih.gov/entrez/query.fcgi?cmd=Retrieve&db=pubmed&dopt=Abstract&list_uids=24333506|6|Exp Cell Res|321|2|Feb|2014|Journal Article,Research Support, N.I.H., Extramural,Research Support, Non-U.S. Gov't
rhoa	akt1	http://www.ncbi.nlm.nih.gov/entrez/query.fcgi?cmd=Retrieve&db=pubmed&dopt=Abstract&list_uids=24333506|6|Exp Cell Res|321|2|Feb|2014|Journal Article,Research Support, N.I.H., Extramural,Research Support, Non-U.S. Gov't|http://www.ncbi.nlm.nih.gov/entrez/query.fcgi?cmd=Retrieve&db=pubmed&dopt=Abstract&list_uids=23836527|6|Stem Cells|31|11|Nov|2013|Journal Article,Research Support, N.I.H., Extramural
nsf	mmrn1	http://www.ncbi.nlm.nih.gov/entrez/query.fcgi?cmd=Retrieve&db=pubmed&dopt=Abstract&list_uids=24311785|6|J Biol Chem|289|4|Jan|2014|Journal Article,Research Support, N.I.H., Extramural,Research Support, Non-U.S. Gov't
pik3ca	ptk2	http://www.ncbi.nlm.nih.gov/entrez/query.fcgi?cmd=Retrieve&db=pubmed&dopt=Abstract&list_uids=24290759|6|Cell Rep|5|5|Dec|2013|Journal Article,Research Support, Non-U.S. Gov't
pik3ca	ptk2b	http://www.ncbi.nlm.nih.gov/entrez/query.fcgi?cmd=Retrieve&db=pubmed&dopt=Abstract&list_uids=24290759|6|Cell Rep|5|5|Dec|2013|Journal Article,Research Support, Non-U.S. Gov't
rhoa	arap3	http://www.ncbi.nlm.nih.gov/entrez/query.fcgi?cmd=Retrieve&db=pubmed&dopt=Abstract&list_uids=24290759|6|Cell Rep|5|5|Dec|2013|Journal Article,Research Support, Non-U.S. Gov't
mmrn1	mmp2	http://www.ncbi.nlm.nih.gov/entrez/query.fcgi?cmd=Retrieve&db=pubmed&dopt=Abstract&list_uids=24289792|6|Arthritis Res Ther|15|5||2013|Journal Article,Research Support, Non-U.S. Gov't
ptk2	mmp2	http://www.ncbi.nlm.nih.gov/entrez/query.fcgi?cmd=Retrieve&db=pubmed&dopt=Abstract&list_uids=24289792|6|Arthritis Res Ther|15|5||2013|Journal Article,Research Support, Non-U.S. Gov't
ephb2	ptk2	http://www.ncbi.nlm.nih.gov/entrez/query.fcgi?cmd=Retrieve&db=pubmed&dopt=Abstract&list_uids=24091658|6|Cell Death Dis|4|||2013|Journal Article,Research Support, Non-U.S. Gov't|http://www.ncbi.nlm.nih.gov/entrez/query.fcgi?cmd=Retrieve&db=pubmed&dopt=Abstract&list_uids=24252447|6|Acta Biomater|10|2|Feb|2014|Journal Article,Research Support, Non-U.S. Gov't
ptk2	pcsk1	http://www.ncbi.nlm.nih.gov/entrez/query.fcgi?cmd=Retrieve&db=pubmed&dopt=Abstract&list_uids=24194567|6|Mol Cancer Ther|13|1|Jan|2014|Journal Article,Research Support, Non-U.S. Gov't
tgm2	jun	http://www.ncbi.nlm.nih.gov/entrez/query.fcgi?cmd=Retrieve&db=pubmed&dopt=Abstract&list_uids=24193434|6|Cell Adh Migr|7|5||2013|Journal Article,Research Support, Non-U.S. Gov't
tgm2	mapk8	http://www.ncbi.nlm.nih.gov/entrez/query.fcgi?cmd=Retrieve&db=pubmed&dopt=Abstract&list_uids=24193434|6|Cell Adh Migr|7|5||2013|Journal Article,Research Support, Non-U.S. Gov't
dapk1	actin	http://www.ncbi.nlm.nih.gov/entrez/query.fcgi?cmd=Retrieve&db=pubmed&dopt=Abstract&list_uids=24166137|6|Apoptosis|19|2|Feb|2014|Journal Article,Research Support, Non-U.S. Gov't,Review
dapk1	stxbp1	http://www.ncbi.nlm.nih.gov/entrez/query.fcgi?cmd=Retrieve&db=pubmed&dopt=Abstract&list_uids=24166137|6|Apoptosis|19|2|Feb|2014|Journal Article,Research Support, Non-U.S. Gov't,Review
frap1	pdlim5	http://www.ncbi.nlm.nih.gov/entrez/query.fcgi?cmd=Retrieve&db=pubmed&dopt=Abstract&list_uids=24165133|6|J Biol Chem|288|49|Dec|2013|Journal Article,Research Support, N.I.H., Extramural,Research Support, Non-U.S. Gov't
rhoa	cyb5r3	http://www.ncbi.nlm.nih.gov/entrez/query.fcgi?cmd=Retrieve&db=pubmed&dopt=Abstract&list_uids=24126053|6|Mol Cell Biol|33|24|Dec|2013|Journal Article,Research Support, N.I.H., Extramural

cyb5r3	actin	http://www.ncbi.nlm.nih.gov/entrez/query.fcgi?cmd=Retrieve&db=pubmed&dopt=Abstract&list_uids=24126053|6|Mol Cell Biol|33|24|Dec|2013|Journal Article,Research Support, N.I.H., Extramural
actin	yap1	http://www.ncbi.nlm.nih.gov/entrez/query.fcgi?cmd=Retrieve&db=pubmed&dopt=Abstract&list_uids=24120037|6|Biomaterials|35|1|Jan|2014|Journal Article
map3k7	itk	http://www.ncbi.nlm.nih.gov/entrez/query.fcgi?cmd=Retrieve&db=pubmed&dopt=Abstract&list_uids=24113182|6|Cell Death Dis|4|||2013|Journal Article,Research Support, Non-U.S. Gov't
lats1	yap1	http://www.ncbi.nlm.nih.gov/entrez/query.fcgi?cmd=Retrieve&db=pubmed&dopt=Abstract&list_uids=22215811|6|Genes Dev|26|1|Jan|2012|Journal Article,Research Support, N.I.H., Extramural,Research Support, U.S. Gov't, Non-P.H.S.|http://www.ncbi.nlm.nih.gov/entrez/query.fcgi?cmd=Retrieve&db=pubmed&dopt=Abstract&list_uids=24106267|6|J Biol Chem|288|47|Nov|2013|Journal Article,Research Support, Non-U.S. Gov't
lats1	taz	http://www.ncbi.nlm.nih.gov/entrez/query.fcgi?cmd=Retrieve&db=pubmed&dopt=Abstract&list_uids=24106267|6|J Biol Chem|288|47|Nov|2013|Journal Article,Research Support, Non-U.S. Gov't
rbl2	lats1	http://www.ncbi.nlm.nih.gov/entrez/query.fcgi?cmd=Retrieve&db=pubmed&dopt=Abstract&list_uids=24106267|6|J Biol Chem|288|47|Nov|2013|Journal Article,Research Support, Non-U.S. Gov't
rbl2	fzd4	http://www.ncbi.nlm.nih.gov/entrez/query.fcgi?cmd=Retrieve&db=pubmed&dopt=Abstract&list_uids=24106267|6|J Biol Chem|288|47|Nov|2013|Journal Article,Research Support, Non-U.S. Gov't
lats1	fzd4	http://www.ncbi.nlm.nih.gov/entrez/query.fcgi?cmd=Retrieve&db=pubmed&dopt=Abstract&list_uids=24106267|6|J Biol Chem|288|47|Nov|2013|Journal Article,Research Support, Non-U.S. Gov't
amot	lats1	http://www.ncbi.nlm.nih.gov/entrez/query.fcgi?cmd=Retrieve&db=pubmed&dopt=Abstract&list_uids=24106267|6|J Biol Chem|288|47|Nov|2013|Journal Article,Research Support, Non-U.S. Gov't
egfr	ephb2	http://www.ncbi.nlm.nih.gov/entrez/query.fcgi?cmd=Retrieve&db=pubmed&dopt=Abstract&list_uids=24091658|6|Cell Death Dis|4|||2013|Journal Article,Research Support, Non-U.S. Gov't
egfr	cdk5	http://www.ncbi.nlm.nih.gov/entrez/query.fcgi?cmd=Retrieve&db=pubmed&dopt=Abstract&list_uids=24091658|6|Cell Death Dis|4|||2013|Journal Article,Research Support, Non-U.S. Gov't
ephb2	cdk5	http://www.ncbi.nlm.nih.gov/entrez/query.fcgi?cmd=Retrieve&db=pubmed&dopt=Abstract&list_uids=24091658|6|Cell Death Dis|4|||2013|Journal Article,Research Support, Non-U.S. Gov't
cdk5	ptk2	http://www.ncbi.nlm.nih.gov/entrez/query.fcgi?cmd=Retrieve&db=pubmed&dopt=Abstract&list_uids=24091658|6|Cell Death Dis|4|||2013|Journal Article,Research Support, Non-U.S. Gov't
egf	timm8a	http://www.ncbi.nlm.nih.gov/entrez/query.fcgi?cmd=Retrieve&db=pubmed&dopt=Abstract&list_uids=24091658|6|Cell Death Dis|4|||2013|Journal Article,Research Support, Non-U.S. Gov't
egfr	timm8a	http://www.ncbi.nlm.nih.gov/entrez/query.fcgi?cmd=Retrieve&db=pubmed&dopt=Abstract&list_uids=24091658|6|Cell Death Dis|4|||2013|Journal Article,Research Support, Non-U.S. Gov't
ptk2	2.7.7.48	http://www.ncbi.nlm.nih.gov/entrez/query.fcgi?cmd=Retrieve&db=pubmed&dopt=Abstract&list_uids=24061591|6|Dig Dis Sci|58|12|Dec|2013|Journal Article,Research Support, Non-U.S. Gov't
ptk2	ervk2	http://www.ncbi.nlm.nih.gov/entrez/query.fcgi?cmd=Retrieve&db=pubmed&dopt=Abstract&list_uids=24061591|6|Dig Dis Sci|58|12|Dec|2013|Journal Article,Research Support, Non-U.S. Gov't

lpa	2.7.7.48	http://www.ncbi.nlm.nih.gov/entrez/query.fcgi?cmd=Retrieve&db=pubmed&dopt=Abstract&list_uids=24061591|6|Dig Dis Sci|58|12|Dec|2013|Journal Article,Research Support, Non-U.S. Gov't
lpa	ervk2	http://www.ncbi.nlm.nih.gov/entrez/query.fcgi?cmd=Retrieve&db=pubmed&dopt=Abstract&list_uids=24061591|6|Dig Dis Sci|58|12|Dec|2013|Journal Article,Research Support, Non-U.S. Gov't
2.7.7.48	ervk2	http://www.ncbi.nlm.nih.gov/entrez/query.fcgi?cmd=Retrieve&db=pubmed&dopt=Abstract&list_uids=24061591|6|Dig Dis Sci|58|12|Dec|2013|Journal Article,Research Support, Non-U.S. Gov't
lpar1	lpar3	http://www.ncbi.nlm.nih.gov/entrez/query.fcgi?cmd=Retrieve&db=pubmed&dopt=Abstract&list_uids=24061591|6|Dig Dis Sci|58|12|Dec|2013|Journal Article,Research Support, Non-U.S. Gov't
lpar1	lpa	http://www.ncbi.nlm.nih.gov/entrez/query.fcgi?cmd=Retrieve&db=pubmed&dopt=Abstract&list_uids=24061591|6|Dig Dis Sci|58|12|Dec|2013|Journal Article,Research Support, Non-U.S. Gov't
lpar1	ptk2	http://www.ncbi.nlm.nih.gov/entrez/query.fcgi?cmd=Retrieve&db=pubmed&dopt=Abstract&list_uids=24061591|6|Dig Dis Sci|58|12|Dec|2013|Journal Article,Research Support, Non-U.S. Gov't
lpar3	lpa	http://www.ncbi.nlm.nih.gov/entrez/query.fcgi?cmd=Retrieve&db=pubmed&dopt=Abstract&list_uids=24061591|6|Dig Dis Sci|58|12|Dec|2013|Journal Article,Research Support, Non-U.S. Gov't
lpar3	ptk2	http://www.ncbi.nlm.nih.gov/entrez/query.fcgi?cmd=Retrieve&db=pubmed&dopt=Abstract&list_uids=24061591|6|Dig Dis Sci|58|12|Dec|2013|Journal Article,Research Support, Non-U.S. Gov't
marveld1	actin	http://www.ncbi.nlm.nih.gov/entrez/query.fcgi?cmd=Retrieve&db=pubmed&dopt=Abstract&list_uids=24055813|6|Int J Biochem Cell Biol|45|11|Nov|2013|Journal Article,Research Support, Non-U.S. Gov't
marveld1	sfrs2	http://www.ncbi.nlm.nih.gov/entrez/query.fcgi?cmd=Retrieve&db=pubmed&dopt=Abstract&list_uids=24055813|6|Int J Biochem Cell Biol|45|11|Nov|2013|Journal Article,Research Support, Non-U.S. Gov't
marveld1	ncbp2	http://www.ncbi.nlm.nih.gov/entrez/query.fcgi?cmd=Retrieve&db=pubmed&dopt=Abstract&list_uids=24055813|6|Int J Biochem Cell Biol|45|11|Nov|2013|Journal Article,Research Support, Non-U.S. Gov't
sfrs2	ncbp2	http://www.ncbi.nlm.nih.gov/entrez/query.fcgi?cmd=Retrieve&db=pubmed&dopt=Abstract&list_uids=24055813|6|Int J Biochem Cell Biol|45|11|Nov|2013|Journal Article,Research Support, Non-U.S. Gov't
marveld2	elmo1	http://www.ncbi.nlm.nih.gov/entrez/query.fcgi?cmd=Retrieve&db=pubmed&dopt=Abstract&list_uids=24050303|6|Fluids Barriers CNS|10|1||2013|Journal Article
3.6.4.1	fas	http://www.ncbi.nlm.nih.gov/entrez/query.fcgi?cmd=Retrieve&db=pubmed&dopt=Abstract&list_uids=23830911|6|Curr Opin Cell Biol|25|5|Oct|2013|Journal Article,Research Support, N.I.H., Intramural,Review|http://www.ncbi.nlm.nih.gov/entrez/query.fcgi?cmd=Retrieve&db=pubmed&dopt=Abstract&list_uids=22524451|6|Cell Biol Int|36|7|Jul|2012|Journal Article,Research Support, Non-U.S. Gov't|http://www.ncbi.nlm.nih.gov/entrez/query.fcgi?cmd=Retrieve&db=pubmed&dopt=Abstract&list_uids=24039980|6|PLoS One|8|9||2013|Journal Article,Research Support, N.I.H., Extramural,Research Support, Non-U.S. Gov't
actin	csrp1	http://www.ncbi.nlm.nih.gov/entrez/query.fcgi?cmd=Retrieve&db=pubmed&dopt=Abstract&list_uids=24036928|6|Cell Cycle|12|21|Nov|2013|Journal Article,Research Support, N.I.H., Extramural,Research Support, Non-U.S. Gov't
hras	ephb2	http://www.ncbi.nlm.nih.gov/entrez/query.fcgi?cmd=Retrieve&db=pubmed&dopt=Abstract&list_uids=23995792|6|Oncogene|33|28|Jul|2014|Journal Article,Research Support, Non-U.S. Gov't

hras	mapk3	http://www.ncbi.nlm.nih.gov/entrez/query.fcgi?cmd=Retrieve&db=pubmed&dopt=Abstract&list_uids=23995792|6|Oncogene|33|28|Jul|2014|Journal Article,Research Support, Non-U.S. Gov't
ephb2	pik3ca	http://www.ncbi.nlm.nih.gov/entrez/query.fcgi?cmd=Retrieve&db=pubmed&dopt=Abstract&list_uids=23995792|6|Oncogene|33|28|Jul|2014|Journal Article,Research Support, Non-U.S. Gov't
akt1	cdc42	http://www.ncbi.nlm.nih.gov/entrez/query.fcgi?cmd=Retrieve&db=pubmed&dopt=Abstract&list_uids=23344455|6|Toxins (Basel)|5|1|Jan|2013|Comparative Study,Journal Article,Research Support, Non-U.S. Gov't|http://www.ncbi.nlm.nih.gov/entrez/query.fcgi?cmd=Retrieve&db=pubmed&dopt=Abstract&list_uids=23974244|6|Cell Mol Life Sci|71|10|May|2014|Journal Article,Research Support, Non-U.S. Gov't
tnc	itk	http://www.ncbi.nlm.nih.gov/entrez/query.fcgi?cmd=Retrieve&db=pubmed&dopt=Abstract&list_uids=23958855|6|Oncogenesis|2|||2013|Journal Article
tnc	tnfsf13	http://www.ncbi.nlm.nih.gov/entrez/query.fcgi?cmd=Retrieve&db=pubmed&dopt=Abstract&list_uids=23958855|6|Oncogenesis|2|||2013|Journal Article
tnfsf13	itk	http://www.ncbi.nlm.nih.gov/entrez/query.fcgi?cmd=Retrieve&db=pubmed&dopt=Abstract&list_uids=23958855|6|Oncogenesis|2|||2013|Journal Article
mmp3	ephb2	http://www.ncbi.nlm.nih.gov/entrez/query.fcgi?cmd=Retrieve&db=pubmed&dopt=Abstract&list_uids=23940616|6|PLoS One|8|8||2013|Journal Article,Research Support, N.I.H., Extramural,Research Support, Non-U.S. Gov't|http://www.ncbi.nlm.nih.gov/entrez/query.fcgi?cmd=Retrieve&db=pubmed&dopt=Abstract&list_uids=22829527|6|FASEB J|26|11|Nov|2012|Journal Article,Research Support, N.I.H., Extramural,Research Support, Non-U.S. Gov't
plau	vegfa	http://www.ncbi.nlm.nih.gov/entrez/query.fcgi?cmd=Retrieve&db=pubmed&dopt=Abstract&list_uids=23939376|6|Mol Cancer Ther|12|10|Oct|2013|Journal Article,Research Support, Non-U.S. Gov't
plau	plaur	http://www.ncbi.nlm.nih.gov/entrez/query.fcgi?cmd=Retrieve&db=pubmed&dopt=Abstract&list_uids=23485561|6|Mol Biol Cell|24|9|May|2013|Journal Article,Research Support, Non-U.S. Gov't|http://www.ncbi.nlm.nih.gov/entrez/query.fcgi?cmd=Retrieve&db=pubmed&dopt=Abstract&list_uids=23939376|6|Mol Cancer Ther|12|10|Oct|2013|Journal Article,Research Support, Non-U.S. Gov't
btg2	ros1	http://www.ncbi.nlm.nih.gov/entrez/query.fcgi?cmd=Retrieve&db=pubmed&dopt=Abstract&list_uids=23907596|6|J Cancer Res Clin Oncol|139|10|Oct|2013|Journal Article,Research Support, Non-U.S. Gov't
btg2	actin	http://www.ncbi.nlm.nih.gov/entrez/query.fcgi?cmd=Retrieve&db=pubmed&dopt=Abstract&list_uids=23907596|6|J Cancer Res Clin Oncol|139|10|Oct|2013|Journal Article,Research Support, Non-U.S. Gov't
ros1	actin	http://www.ncbi.nlm.nih.gov/entrez/query.fcgi?cmd=Retrieve&db=pubmed&dopt=Abstract&list_uids=23907596|6|J Cancer Res Clin Oncol|139|10|Oct|2013|Journal Article,Research Support, Non-U.S. Gov't
nedd9	bcar1	http://www.ncbi.nlm.nih.gov/entrez/query.fcgi?cmd=Retrieve&db=pubmed&dopt=Abstract&list_uids=23874939|6|PLoS One|8|7||2013|Journal Article,Research Support, Non-U.S. Gov't
nedd9	cse1l	http://www.ncbi.nlm.nih.gov/entrez/query.fcgi?cmd=Retrieve&db=pubmed&dopt=Abstract&list_uids=23874939|6|PLoS One|8|7||2013|Journal Article,Research Support, Non-U.S. Gov't
pkd1	vasp	http://www.ncbi.nlm.nih.gov/entrez/query.fcgi?cmd=Retrieve&db=pubmed&dopt=Abstract&list_uids=23846685|6|J Biol Chem|288|34|Aug|2013|Journal Article,Research Support, N.I.H., Extramural,Research Support, Non-U.S. Gov't

rhoa	vasp	http://www.ncbi.nlm.nih.gov/entrez/query.fcgi?cmd=Retrieve&db=pubmed&dopt=Abstract&list_uids=23846685|6|J Biol Chem|288|34|Aug|2013|Journal Article,Research Support, N.I.H., Extramural,Research Support, Non-U.S. Gov't
fyn	rhoa	http://www.ncbi.nlm.nih.gov/entrez/query.fcgi?cmd=Retrieve&db=pubmed&dopt=Abstract&list_uids=23836527|6|Stem Cells|31|11|Nov|2013|Journal Article,Research Support, N.I.H., Extramural
fas	src	http://www.ncbi.nlm.nih.gov/entrez/query.fcgi?cmd=Retrieve&db=pubmed&dopt=Abstract&list_uids=23754369|6|Proc Natl Acad Sci U S A|110|26|Jun|2013|Journal Article,Research Support, N.I.H., Intramural,Research Support, Non-U.S. Gov't|http://www.ncbi.nlm.nih.gov/entrez/query.fcgi?cmd=Retrieve&db=pubmed&dopt=Abstract&list_uids=22291036|6|J Cell Biol|196|3|Feb|2012|Journal Article,Research Support, N.I.H., Extramural|http://www.ncbi.nlm.nih.gov/entrez/query.fcgi?cmd=Retrieve&db=pubmed&dopt=Abstract&list_uids=23836527|6|Stem Cells|31|11|Nov|2013|Journal Article,Research Support, N.I.H., Extramural
fyn	fas	http://www.ncbi.nlm.nih.gov/entrez/query.fcgi?cmd=Retrieve&db=pubmed&dopt=Abstract&list_uids=23836527|6|Stem Cells|31|11|Nov|2013|Journal Article,Research Support, N.I.H., Extramural
ptk2	fyn	http://www.ncbi.nlm.nih.gov/entrez/query.fcgi?cmd=Retrieve&db=pubmed&dopt=Abstract&list_uids=23836527|6|Stem Cells|31|11|Nov|2013|Journal Article,Research Support, N.I.H., Extramural
fyn	actin	http://www.ncbi.nlm.nih.gov/entrez/query.fcgi?cmd=Retrieve&db=pubmed&dopt=Abstract&list_uids=23836527|6|Stem Cells|31|11|Nov|2013|Journal Article,Research Support, N.I.H., Extramural
fyn	msc	http://www.ncbi.nlm.nih.gov/entrez/query.fcgi?cmd=Retrieve&db=pubmed&dopt=Abstract&list_uids=23836527|6|Stem Cells|31|11|Nov|2013|Journal Article,Research Support, N.I.H., Extramural
fyn	adipoq	http://www.ncbi.nlm.nih.gov/entrez/query.fcgi?cmd=Retrieve&db=pubmed&dopt=Abstract&list_uids=23836527|6|Stem Cells|31|11|Nov|2013|Journal Article,Research Support, N.I.H., Extramural
msc	src	http://www.ncbi.nlm.nih.gov/entrez/query.fcgi?cmd=Retrieve&db=pubmed&dopt=Abstract&list_uids=23836527|6|Stem Cells|31|11|Nov|2013|Journal Article,Research Support, N.I.H., Extramural
msc	adipoq	http://www.ncbi.nlm.nih.gov/entrez/query.fcgi?cmd=Retrieve&db=pubmed&dopt=Abstract&list_uids=23836527|6|Stem Cells|31|11|Nov|2013|Journal Article,Research Support, N.I.H., Extramural
src	adipoq	http://www.ncbi.nlm.nih.gov/entrez/query.fcgi?cmd=Retrieve&db=pubmed&dopt=Abstract&list_uids=23836527|6|Stem Cells|31|11|Nov|2013|Journal Article,Research Support, N.I.H., Extramural
3.6.4.1	mmrn1	http://www.ncbi.nlm.nih.gov/entrez/query.fcgi?cmd=Retrieve&db=pubmed&dopt=Abstract&list_uids=23830911|6|Curr Opin Cell Biol|25|5|Oct|2013|Journal Article,Research Support, N.I.H., Intramural,Review|http://www.ncbi.nlm.nih.gov/entrez/query.fcgi?cmd=Retrieve&db=pubmed&dopt=Abstract&list_uids=23708000|6|Nat Cell Biol|15|6|Jun|2013|Journal Article,Research Support, Non-U.S. Gov't
cdc42	actin	http://www.ncbi.nlm.nih.gov/entrez/query.fcgi?cmd=Retrieve&db=pubmed&dopt=Abstract&list_uids=23826261|6|PLoS One|8|6||2013|Journal Article,Research Support, Non-U.S. Gov't
mirn29a	cdc42	http://www.ncbi.nlm.nih.gov/entrez/query.fcgi?cmd=Retrieve&db=pubmed&dopt=Abstract&list_uids=23826261|6|PLoS One|8|6||2013|Journal Article,Research Support, Non-U.S. Gov't
net1	rhoa	http://www.ncbi.nlm.nih.gov/entrez/query.fcgi?cmd=Retrieve&db=pubmed&dopt=Abstract&list_uids=23792411|6|Cell Adh Migr|7|4||2013|Comment,Journal Article,Research Support, N.I.H., Extramural,Research Support, Non-U.S.

		Gov't|http://www.ncbi.nlm.nih.gov/entrez/query.fcgi?cmd=Retrieve&db=pubmed&dopt=Abstract&list_uids=23689132|6|Mol Cell Biol|33|14|Jul|2013|Journal Article,Research Support, N.I.H., Extramural,Research Support, Non-U.S. Gov't|http://www.ncbi.nlm.nih.gov/entrez/query.fcgi?cmd=Retrieve&db=pubmed&dopt=Abstract&list_uids=23184663|6|Mol Cell Biol|33|3|Feb|2013|Journal Article,Research Support, N.I.H., Extramural
rac1	net1	http://www.ncbi.nlm.nih.gov/entrez/query.fcgi?cmd=Retrieve&db=pubmed&dopt=Abstract&list_uids=23792411|6|Cell Adh Migr|7|4||2013|Comment,Journal Article,Research Support, N.I.H., Extramural,Research Support, Non-U.S. Gov't|http://www.ncbi.nlm.nih.gov/entrez/query.fcgi?cmd=Retrieve&db=pubmed&dopt=Abstract&list_uids=23184663|6|Mol Cell Biol|33|3|Feb|2013|Journal Article,Research Support, N.I.H., Extramural
net1	myh14	http://www.ncbi.nlm.nih.gov/entrez/query.fcgi?cmd=Retrieve&db=pubmed&dopt=Abstract&list_uids=23792411|6|Cell Adh Migr|7|4||2013|Comment,Journal Article,Research Support, N.I.H., Extramural,Research Support, Non-U.S. Gov't|http://www.ncbi.nlm.nih.gov/entrez/query.fcgi?cmd=Retrieve&db=pubmed&dopt=Abstract&list_uids=23689132|6|Mol Cell Biol|33|14|Jul|2013|Journal Article,Research Support, N.I.H., Extramural,Research Support, Non-U.S. Gov't|http://www.ncbi.nlm.nih.gov/entrez/query.fcgi?cmd=Retrieve&db=pubmed&dopt=Abstract&list_uids=23184663|6|Mol Cell Biol|33|3|Feb|2013|Journal Article,Research Support, N.I.H., Extramural
bcar3	actin	http://www.ncbi.nlm.nih.gov/entrez/query.fcgi?cmd=Retrieve&db=pubmed&dopt=Abstract&list_uids=23762409|6|PLoS One|8|6||2013|Journal Article,Research Support, N.I.H., Extramural,Research Support, Non-U.S. Gov't
bcar3	rhoa	http://www.ncbi.nlm.nih.gov/entrez/query.fcgi?cmd=Retrieve&db=pubmed&dopt=Abstract&list_uids=23762409|6|PLoS One|8|6||2013|Journal Article,Research Support, N.I.H., Extramural,Research Support, Non-U.S. Gov't
bcar3	myh14	http://www.ncbi.nlm.nih.gov/entrez/query.fcgi?cmd=Retrieve&db=pubmed&dopt=Abstract&list_uids=23762409|6|PLoS One|8|6||2013|Journal Article,Research Support, N.I.H., Extramural,Research Support, Non-U.S. Gov't
bbs4	rhoa	http://www.ncbi.nlm.nih.gov/entrez/query.fcgi?cmd=Retrieve&db=pubmed&dopt=Abstract&list_uids=23716571|6|Hum Mol Genet|22|19|Oct|2013|Journal Article,Research Support, Non-U.S. Gov't
bbs4	actin	http://www.ncbi.nlm.nih.gov/entrez/query.fcgi?cmd=Retrieve&db=pubmed&dopt=Abstract&list_uids=23716571|6|Hum Mol Genet|22|19|Oct|2013|Journal Article,Research Support, Non-U.S. Gov't
cox7a2l	mapre3	http://www.ncbi.nlm.nih.gov/entrez/query.fcgi?cmd=Retrieve&db=pubmed&dopt=Abstract&list_uids=23712260|6|J Cell Biol|201|5|May|2013|Journal Article,Research Support, Non-U.S. Gov't|http://www.ncbi.nlm.nih.gov/entrez/query.fcgi?cmd=Retrieve&db=pubmed&dopt=Abstract&list_uids=22949735|6|Sci Signal|5|240|Sep|2012|Comparative Study,Journal Article,Research Support, N.I.H., Extramural,Research Support, Non-U.S. Gov't
yap1	tbx1	http://www.ncbi.nlm.nih.gov/entrez/query.fcgi?cmd=Retrieve&db=pubmed&dopt=Abstract&list_uids=23708000|6|Nat Cell Biol|15|6|Jun|2013|Journal Article,Research Support, Non-U.S. Gov't
yap1	kat2b	http://www.ncbi.nlm.nih.gov/entrez/query.fcgi?cmd=Retrieve&db=pubmed&dopt=Abstract&list_uids=23708000|6|Nat Cell Biol|15|6|Jun|2013|Journal Article,Research Support, Non-U.S. Gov't
3.6.4.1	yap1	http://www.ncbi.nlm.nih.gov/entrez/query.fcgi?cmd=Retrieve&db=pubmed&dopt=Abstract&list_uids=23708000|6|Nat Cell Biol|15|6|Jun|2013|Journal Article,Research Support, Non-U.S. Gov't
src	yap1	http://www.ncbi.nlm.nih.gov/entrez/query.fcgi?cmd=Retrieve&db=pubmed&dopt=Abstract&list_uids=23708000|6|Nat Cell

		Biol|15|6|Jun|2013|Journal Article,Research Support, Non-U.S. Gov't
abl2	actin	http://www.ncbi.nlm.nih.gov/entrez/query.fcgi?cmd=Retrieve&db=pubmed&dopt=Abstract&list_uids=23707396|6|Exp Cell Res|319|13|Aug|2013|Journal Article
abl2	rhoa	http://www.ncbi.nlm.nih.gov/entrez/query.fcgi?cmd=Retrieve&db=pubmed&dopt=Abstract&list_uids=23707396|6|Exp Cell Res|319|13|Aug|2013|Journal Article
abl2	grlf1	http://www.ncbi.nlm.nih.gov/entrez/query.fcgi?cmd=Retrieve&db=pubmed&dopt=Abstract&list_uids=23707396|6|Exp Cell Res|319|13|Aug|2013|Journal Article
abl2	crk	http://www.ncbi.nlm.nih.gov/entrez/query.fcgi?cmd=Retrieve&db=pubmed&dopt=Abstract&list_uids=23707396|6|Exp Cell Res|319|13|Aug|2013|Journal Article
actin	grlf1	http://www.ncbi.nlm.nih.gov/entrez/query.fcgi?cmd=Retrieve&db=pubmed&dopt=Abstract&list_uids=23707396|6|Exp Cell Res|319|13|Aug|2013|Journal Article
actin	crk	http://www.ncbi.nlm.nih.gov/entrez/query.fcgi?cmd=Retrieve&db=pubmed&dopt=Abstract&list_uids=23707396|6|Exp Cell Res|319|13|Aug|2013|Journal Article
rhoa	crk	http://www.ncbi.nlm.nih.gov/entrez/query.fcgi?cmd=Retrieve&db=pubmed&dopt=Abstract&list_uids=23707396|6|Exp Cell Res|319|13|Aug|2013|Journal Article
grlf1	crk	http://www.ncbi.nlm.nih.gov/entrez/query.fcgi?cmd=Retrieve&db=pubmed&dopt=Abstract&list_uids=23707396|6|Exp Cell Res|319|13|Aug|2013|Journal Article
net1	lpa	http://www.ncbi.nlm.nih.gov/entrez/query.fcgi?cmd=Retrieve&db=pubmed&dopt=Abstract&list_uids=23689132|6|Mol Cell Biol|33|14|Jul|2013|Journal Article,Research Support, N.I.H., Extramural,Research Support, Non-U.S. Gov't
net1	ptk2	http://www.ncbi.nlm.nih.gov/entrez/query.fcgi?cmd=Retrieve&db=pubmed&dopt=Abstract&list_uids=23689132|6|Mol Cell Biol|33|14|Jul|2013|Journal Article,Research Support, N.I.H., Extramural,Research Support, Non-U.S. Gov't
net1	mmrn1	http://www.ncbi.nlm.nih.gov/entrez/query.fcgi?cmd=Retrieve&db=pubmed&dopt=Abstract&list_uids=23689132|6|Mol Cell Biol|33|14|Jul|2013|Journal Article,Research Support, N.I.H., Extramural,Research Support, Non-U.S. Gov't
zdhhc1	rab5a	http://www.ncbi.nlm.nih.gov/entrez/query.fcgi?cmd=Retrieve&db=pubmed&dopt=Abstract&list_uids=23687301|6|J Biol Chem|288|27|Jul|2013|Journal Article,Research Support, Non-U.S. Gov't
epha1	epha2	http://www.ncbi.nlm.nih.gov/entrez/query.fcgi?cmd=Retrieve&db=pubmed&dopt=Abstract&list_uids=23686814|6|Cell Biol Int|37|10|Oct|2013|Journal Article,Research Support, Non-U.S. Gov't
ptk2	tp53	http://www.ncbi.nlm.nih.gov/entrez/query.fcgi?cmd=Retrieve&db=pubmed&dopt=Abstract&list_uids=23686429|6|Mol Cells|36|1|Jul|2013|Journal Article,Research Support, Non-U.S. Gov't,Review
ptk2	gata4	http://www.ncbi.nlm.nih.gov/entrez/query.fcgi?cmd=Retrieve&db=pubmed&dopt=Abstract&list_uids=23686429|6|Mol Cells|36|1|Jul|2013|Journal Article,Research Support, Non-U.S. Gov't,Review
tp53	gata4	http://www.ncbi.nlm.nih.gov/entrez/query.fcgi?cmd=Retrieve&db=pubmed&dopt=Abstract&list_uids=23686429|6|Mol Cells|36|1|Jul|2013|Journal Article,Research Support, Non-U.S. Gov't,Review
pecam1	kdr	http://www.ncbi.nlm.nih.gov/entrez/query.fcgi?cmd=Retrieve&db=pubmed&dopt=Abstract&list_uids=23684974|6|Curr Biol|23|11|Jun|2013|Journal Article,Research Support, N.I.H., Extramural,Research Support, Non-U.S. Gov't

isl1	myh14	http://www.ncbi.nlm.nih.gov/entrez/query.fcgi?cmd=Retrieve&db=pubmed&dopt=Abstract&list_uids=23681438|6|EMBO Rep|14|6|Jun|2013|Journal Article,Research Support, Non-U.S. Gov't,Review
mat1a	myh14	http://www.ncbi.nlm.nih.gov/entrez/query.fcgi?cmd=Retrieve&db=pubmed&dopt=Abstract&list_uids=23676281|6|Cell Adh Migr|7|3||2013|Journal Article,Research Support, Non-U.S. Gov't
mat1a	dynamin	http://www.ncbi.nlm.nih.gov/entrez/query.fcgi?cmd=Retrieve&db=pubmed&dopt=Abstract&list_uids=23676281|6|Cell Adh Migr|7|3||2013|Journal Article,Research Support, Non-U.S. Gov't
myh14	dynamin	http://www.ncbi.nlm.nih.gov/entrez/query.fcgi?cmd=Retrieve&db=pubmed&dopt=Abstract&list_uids=23676281|6|Cell Adh Migr|7|3||2013|Journal Article,Research Support, Non-U.S. Gov't
stk11	ptk2	http://www.ncbi.nlm.nih.gov/entrez/query.fcgi?cmd=Retrieve&db=pubmed&dopt=Abstract&list_uids=23637231|6|J Biol Chem|288|24|Jun|2013|Journal Article,Research Support, N.I.H., Extramural,Research Support, Non-U.S. Gov't
stk11	tyr	http://www.ncbi.nlm.nih.gov/entrez/query.fcgi?cmd=Retrieve&db=pubmed&dopt=Abstract&list_uids=23637231|6|J Biol Chem|288|24|Jun|2013|Journal Article,Research Support, N.I.H., Extramural,Research Support, Non-U.S. Gov't
ppm1f	mapk1	http://www.ncbi.nlm.nih.gov/entrez/query.fcgi?cmd=Retrieve&db=pubmed&dopt=Abstract&list_uids=23621870|6|J Proteome Res|12|6|Jun|2013|Journal Article,Research Support, Non-U.S. Gov't
ankrd13a	mirn204	http://www.ncbi.nlm.nih.gov/entrez/query.fcgi?cmd=Retrieve&db=pubmed&dopt=Abstract&list_uids=23620728|6|PLoS One|8|4||2013|Journal Article,Research Support, Non-U.S. Gov't
vav2	rhoa	http://www.ncbi.nlm.nih.gov/entrez/query.fcgi?cmd=Retrieve&db=pubmed&dopt=Abstract&list_uids=23615439|6|Mol Biol Cell|24|12|Jun|2013|Journal Article,Research Support, N.I.H., Extramural
vav2	cdc42	http://www.ncbi.nlm.nih.gov/entrez/query.fcgi?cmd=Retrieve&db=pubmed&dopt=Abstract&list_uids=23615439|6|Mol Biol Cell|24|12|Jun|2013|Journal Article,Research Support, N.I.H., Extramural
vav2	pklr	http://www.ncbi.nlm.nih.gov/entrez/query.fcgi?cmd=Retrieve&db=pubmed&dopt=Abstract&list_uids=23615439|6|Mol Biol Cell|24|12|Jun|2013|Journal Article,Research Support, N.I.H., Extramural
vav2	git2	http://www.ncbi.nlm.nih.gov/entrez/query.fcgi?cmd=Retrieve&db=pubmed&dopt=Abstract&list_uids=23615439|6|Mol Biol Cell|24|12|Jun|2013|Journal Article,Research Support, N.I.H., Extramural
arhgef2	rhoa	http://www.ncbi.nlm.nih.gov/entrez/query.fcgi?cmd=Retrieve&db=pubmed&dopt=Abstract&list_uids=23615439|6|Mol Biol Cell|24|12|Jun|2013|Journal Article,Research Support, N.I.H., Extramural|http://www.ncbi.nlm.nih.gov/entrez/query.fcgi?cmd=Retrieve&db=pubmed&dopt=Abstract&list_uids=22949735|6|Sci Signal|5|240|Sep|2012|Comparative Study,Journal Article,Research Support, N.I.H., Extramural,Research Support, Non-U.S. Gov't
arhgef2	cdc42	http://www.ncbi.nlm.nih.gov/entrez/query.fcgi?cmd=Retrieve&db=pubmed&dopt=Abstract&list_uids=23615439|6|Mol Biol Cell|24|12|Jun|2013|Journal Article,Research Support, N.I.H., Extramural
arhgef2	pklr	http://www.ncbi.nlm.nih.gov/entrez/query.fcgi?cmd=Retrieve&db=pubmed&dopt=Abstract&list_uids=23615439|6|Mol Biol Cell|24|12|Jun|2013|Journal Article,Research Support, N.I.H., Extramural
arhgef2	git2	http://www.ncbi.nlm.nih.gov/entrez/query.fcgi?cmd=Retrieve&db=pubmed&dopt=Abstract&list_uids=23615439|6|Mol Biol Cell|24|12|Jun|2013|Journal Article,Research Support, N.I.H., Extramural
rac1	pklr	http://www.ncbi.nlm.nih.gov/entrez/query.fcgi?cmd=Retrieve&db=pubmed&dopt=Abstract&list_uids=23615439|6|Mol Biol

		Cell|24|12|Jun|2013|Journal Article,Research Support, N.I.H., Extramural
rac1	git2	http://www.ncbi.nlm.nih.gov/entrez/query.fcgi?cmd=Retrieve&db=pubmed&dopt=Abstract&list_uids=23615439|6|Mol Biol Cell|24|12|Jun|2013|Journal Article,Research Support, N.I.H., Extramural
rhoa	pklr	http://www.ncbi.nlm.nih.gov/entrez/query.fcgi?cmd=Retrieve&db=pubmed&dopt=Abstract&list_uids=23615439|6|Mol Biol Cell|24|12|Jun|2013|Journal Article,Research Support, N.I.H., Extramural
rhoa	git2	http://www.ncbi.nlm.nih.gov/entrez/query.fcgi?cmd=Retrieve&db=pubmed&dopt=Abstract&list_uids=23615439|6|Mol Biol Cell|24|12|Jun|2013|Journal Article,Research Support, N.I.H., Extramural
cdc42	pklr	http://www.ncbi.nlm.nih.gov/entrez/query.fcgi?cmd=Retrieve&db=pubmed&dopt=Abstract&list_uids=23615439|6|Mol Biol Cell|24|12|Jun|2013|Journal Article,Research Support, N.I.H., Extramural
cdc42	git2	http://www.ncbi.nlm.nih.gov/entrez/query.fcgi?cmd=Retrieve&db=pubmed&dopt=Abstract&list_uids=23615439|6|Mol Biol Cell|24|12|Jun|2013|Journal Article,Research Support, N.I.H., Extramural
pklr	git2	http://www.ncbi.nlm.nih.gov/entrez/query.fcgi?cmd=Retrieve&db=pubmed&dopt=Abstract&list_uids=23615439|6|Mol Biol Cell|24|12|Jun|2013|Journal Article,Research Support, N.I.H., Extramural
pklr	egf	http://www.ncbi.nlm.nih.gov/entrez/query.fcgi?cmd=Retrieve&db=pubmed&dopt=Abstract&list_uids=23615439|6|Mol Biol Cell|24|12|Jun|2013|Journal Article,Research Support, N.I.H., Extramural
vav2	egf	http://www.ncbi.nlm.nih.gov/entrez/query.fcgi?cmd=Retrieve&db=pubmed&dopt=Abstract&list_uids=23615439|6|Mol Biol Cell|24|12|Jun|2013|Journal Article,Research Support, N.I.H., Extramural
rac1	egf	http://www.ncbi.nlm.nih.gov/entrez/query.fcgi?cmd=Retrieve&db=pubmed&dopt=Abstract&list_uids=23615439|6|Mol Biol Cell|24|12|Jun|2013|Journal Article,Research Support, N.I.H., Extramural
arhgef2	egf	http://www.ncbi.nlm.nih.gov/entrez/query.fcgi?cmd=Retrieve&db=pubmed&dopt=Abstract&list_uids=23615439|6|Mol Biol Cell|24|12|Jun|2013|Journal Article,Research Support, N.I.H., Extramural
fos	mirn23b	http://www.ncbi.nlm.nih.gov/entrez/query.fcgi?cmd=Retrieve&db=pubmed&dopt=Abstract&list_uids=23580553|6|Nucleic Acids Res|41|10|May|2013|Journal Article,Research Support, Non-U.S. Gov't
mapk3	map2k1	http://www.ncbi.nlm.nih.gov/entrez/query.fcgi?cmd=Retrieve&db=pubmed&dopt=Abstract&list_uids=23549785|6|J Cell Sci|126|Pt 11|Jun|2013|Journal Article,Research Support, Non-U.S. Gov't|http://www.ncbi.nlm.nih.gov/entrez/query.fcgi?cmd=Retrieve&db=pubmed&dopt=Abstract&list_uids=23562553|6|Biochim Biophys Acta|1830|8|Aug|2013|Journal Article,Research Support, Non-U.S. Gov't
mapk1	mapk3	http://www.ncbi.nlm.nih.gov/entrez/query.fcgi?cmd=Retrieve&db=pubmed&dopt=Abstract&list_uids=23549785|6|J Cell Sci|126|Pt 11|Jun|2013|Journal Article,Research Support, Non-U.S. Gov't
mapk1	hgf	http://www.ncbi.nlm.nih.gov/entrez/query.fcgi?cmd=Retrieve&db=pubmed&dopt=Abstract&list_uids=23549785|6|J Cell Sci|126|Pt 11|Jun|2013|Journal Article,Research Support, Non-U.S. Gov't
mapk3	hgf	http://www.ncbi.nlm.nih.gov/entrez/query.fcgi?cmd=Retrieve&db=pubmed&dopt=Abstract&list_uids=23549785|6|J Cell Sci|126|Pt 11|Jun|2013|Journal Article,Research Support, Non-U.S. Gov't
hgf	ephb2	http://www.ncbi.nlm.nih.gov/entrez/query.fcgi?cmd=Retrieve&db=pubmed&dopt=Abstract&list_uids=23549785|6|J Cell Sci|126|Pt 11|Jun|2013|Journal Article,Research Support, Non-U.S. Gov't

hgf	map2k1	http://www.ncbi.nlm.nih.gov/entrez/query.fcgi?cmd=Retrieve&db=pubmed&dopt=Abstract&list_uids=23549785|6|J Cell Sci|126|Pt 11|Jun|2013|Journal Article,Research Support, Non-U.S. Gov't
mapk3	ephb2	http://www.ncbi.nlm.nih.gov/entrez/query.fcgi?cmd=Retrieve&db=pubmed&dopt=Abstract&list_uids=23549785|6|J Cell Sci|126|Pt 11|Jun|2013|Journal Article,Research Support, Non-U.S. Gov't
ephb2	map2k1	http://www.ncbi.nlm.nih.gov/entrez/query.fcgi?cmd=Retrieve&db=pubmed&dopt=Abstract&list_uids=23549785|6|J Cell Sci|126|Pt 11|Jun|2013|Journal Article,Research Support, Non-U.S. Gov't
tnfsf13	swap70	http://www.ncbi.nlm.nih.gov/entrez/query.fcgi?cmd=Retrieve&db=pubmed&dopt=Abstract&list_uids=23544157|6|PLoS One|8|3||2013|Journal Article,Research Support, Non-U.S. Gov't
tacstd2	pak4	http://www.ncbi.nlm.nih.gov/entrez/query.fcgi?cmd=Retrieve&db=pubmed&dopt=Abstract&list_uids=23536555|6|Cancer Res|73|10|May|2013|Journal Article,Research Support, N.I.H., Extramural,Research Support, Non-U.S. Gov't
ptpn11	egf	http://www.ncbi.nlm.nih.gov/entrez/query.fcgi?cmd=Retrieve&db=pubmed&dopt=Abstract&list_uids=23512980|6|Mol Cancer Res|11|6|Jun|2013|Journal Article,Research Support, N.I.H., Extramural,Research Support, U.S. Gov't, Non-P.H.S.
akt1	sla	http://www.ncbi.nlm.nih.gov/entrez/query.fcgi?cmd=Retrieve&db=pubmed&dopt=Abstract&list_uids=23505566|6|PLoS One|8|3||2013|Journal Article,Research Support, Non-U.S. Gov't
akt1	bmp2	http://www.ncbi.nlm.nih.gov/entrez/query.fcgi?cmd=Retrieve&db=pubmed&dopt=Abstract&list_uids=23505566|6|PLoS One|8|3||2013|Journal Article,Research Support, Non-U.S. Gov't
s100a6	rb1	http://www.ncbi.nlm.nih.gov/entrez/query.fcgi?cmd=Retrieve&db=pubmed&dopt=Abstract&list_uids=23485561|6|Mol Biol Cell|24|9|May|2013|Journal Article,Research Support, Non-U.S. Gov't
plau	ptk2	http://www.ncbi.nlm.nih.gov/entrez/query.fcgi?cmd=Retrieve&db=pubmed&dopt=Abstract&list_uids=23485561|6|Mol Biol Cell|24|9|May|2013|Journal Article,Research Support, Non-U.S. Gov't
plaur	ptk2	http://www.ncbi.nlm.nih.gov/entrez/query.fcgi?cmd=Retrieve&db=pubmed&dopt=Abstract&list_uids=23485561|6|Mol Biol Cell|24|9|May|2013|Journal Article,Research Support, Non-U.S. Gov't
rb1	ptk2	http://www.ncbi.nlm.nih.gov/entrez/query.fcgi?cmd=Retrieve&db=pubmed&dopt=Abstract&list_uids=23485561|6|Mol Biol Cell|24|9|May|2013|Journal Article,Research Support, Non-U.S. Gov't
s100a6	ptk2	http://www.ncbi.nlm.nih.gov/entrez/query.fcgi?cmd=Retrieve&db=pubmed&dopt=Abstract&list_uids=23485561|6|Mol Biol Cell|24|9|May|2013|Journal Article,Research Support, Non-U.S. Gov't
rap1a	apbb1ip	http://www.ncbi.nlm.nih.gov/entrez/query.fcgi?cmd=Retrieve&db=pubmed&dopt=Abstract&list_uids=23389036|6|J Biol Chem|288|12|Mar|2013|Journal Article,Research Support, N.I.H., Extramural,Research Support, Non-U.S. Gov't|http://www.ncbi.nlm.nih.gov/entrez/query.fcgi?cmd=Retrieve&db=pubmed&dopt=Abstract&list_uids=23468527|6|Mol Biol Cell|24|9|May|2013|Journal Article,Research Support, N.I.H., Extramural,Research Support, Non-U.S. Gov't
actin	actn4	http://www.ncbi.nlm.nih.gov/entrez/query.fcgi?cmd=Retrieve&db=pubmed&dopt=Abstract&list_uids=23466492|6|Int J Biochem Cell Biol|45|6|Jun|2013|Journal Article,Research Support, N.I.H., Extramural
ros1	rac1	http://www.ncbi.nlm.nih.gov/entrez/query.fcgi?cmd=Retrieve&db=pubmed&dopt=Abstract&list_uids=23465490|6|Biomaterials|34|15|May|2013|Journal Article,Research Support, N.I.H., Extramural,Research Support, Non-U.S. Gov't,Research Support, U.S. Gov't, Non-P.H.S.
rhoa	ros1	http://www.ncbi.nlm.nih.gov/entrez/query.fcgi?cmd=Retrieve&db=pubmed&dopt=Abstract&list_uids=23465490|6|Biomater

		ials|34|15|May|2013|Journal Article,Research Support, N.I.H., Extramural,Research Support, Non-U.S. Gov't,Research Support, U.S. Gov't, Non-P.H.S.
mirn145	klf4	http://www.ncbi.nlm.nih.gov/entrez/query.fcgi?cmd=Retrieve&db=pubmed&dopt=Abstract&list_uids=23457217|6|FASEB J|27|6|Jun|2013|Journal Article,Research Support, N.I.H., Extramural,Research Support, Non-U.S. Gov't
src	arf6	http://www.ncbi.nlm.nih.gov/entrez/query.fcgi?cmd=Retrieve&db=pubmed&dopt=Abstract&list_uids=23453597|6|Dev Cell|24|5|Mar|2013|Journal Article,Research Support, Non-U.S. Gov't
nck1	actin	http://www.ncbi.nlm.nih.gov/entrez/query.fcgi?cmd=Retrieve&db=pubmed&dopt=Abstract&list_uids=23444376|6|J Cell Sci|126|Pt 7|Apr|2013|Journal Article,Research Support, Non-U.S. Gov't,Research Support, U.S. Gov't, Non-P.H.S.
rhoa	nck1	http://www.ncbi.nlm.nih.gov/entrez/query.fcgi?cmd=Retrieve&db=pubmed&dopt=Abstract&list_uids=23444376|6|J Cell Sci|126|Pt 7|Apr|2013|Journal Article,Research Support, Non-U.S. Gov't,Research Support, U.S. Gov't, Non-P.H.S.
myh14	nck1	http://www.ncbi.nlm.nih.gov/entrez/query.fcgi?cmd=Retrieve&db=pubmed&dopt=Abstract&list_uids=23444376|6|J Cell Sci|126|Pt 7|Apr|2013|Journal Article,Research Support, Non-U.S. Gov't,Research Support, U.S. Gov't, Non-P.H.S.
hcc	hspg2	http://www.ncbi.nlm.nih.gov/entrez/query.fcgi?cmd=Retrieve&db=pubmed&dopt=Abstract&list_uids=23425030|6|J Gastroenterol Hepatol|28|6|Jun|2013|Journal Article,Research Support, Non-U.S. Gov't
hcc	hlf	http://www.ncbi.nlm.nih.gov/entrez/query.fcgi?cmd=Retrieve&db=pubmed&dopt=Abstract&list_uids=23425030|6|J Gastroenterol Hepatol|28|6|Jun|2013|Journal Article,Research Support, Non-U.S. Gov't
hspg2	hlf	http://www.ncbi.nlm.nih.gov/entrez/query.fcgi?cmd=Retrieve&db=pubmed&dopt=Abstract&list_uids=23425030|6|J Gastroenterol Hepatol|28|6|Jun|2013|Journal Article,Research Support, Non-U.S. Gov't
cdkn1a	pak2	http://www.ncbi.nlm.nih.gov/entrez/query.fcgi?cmd=Retrieve&db=pubmed&dopt=Abstract&list_uids=23425030|6|J Gastroenterol Hepatol|28|6|Jun|2013|Journal Article,Research Support, Non-U.S. Gov't
pak2	hcc	http://www.ncbi.nlm.nih.gov/entrez/query.fcgi?cmd=Retrieve&db=pubmed&dopt=Abstract&list_uids=23425030|6|J Gastroenterol Hepatol|28|6|Jun|2013|Journal Article,Research Support, Non-U.S. Gov't
pak2	akt1	http://www.ncbi.nlm.nih.gov/entrez/query.fcgi?cmd=Retrieve&db=pubmed&dopt=Abstract&list_uids=23425030|6|J Gastroenterol Hepatol|28|6|Jun|2013|Journal Article,Research Support, Non-U.S. Gov't
hcc	akt1	http://www.ncbi.nlm.nih.gov/entrez/query.fcgi?cmd=Retrieve&db=pubmed&dopt=Abstract&list_uids=23425030|6|J Gastroenterol Hepatol|28|6|Jun|2013|Journal Article,Research Support, Non-U.S. Gov't
abcb6	gba	http://www.ncbi.nlm.nih.gov/entrez/query.fcgi?cmd=Retrieve&db=pubmed&dopt=Abstract&list_uids=23424639|6|PLoS One|8|2||2013|Comparative Study,Journal Article,Research Support, Non-U.S. Gov't
il1b	il8	http://www.ncbi.nlm.nih.gov/entrez/query.fcgi?cmd=Retrieve&db=pubmed&dopt=Abstract&list_uids=23399841|6|Cell Immunol|280|2|Dec|2012|Journal Article,Research Support, N.I.H., Extramural,Research Support, Non-U.S. Gov't
il1b	ccl2	http://www.ncbi.nlm.nih.gov/entrez/query.fcgi?cmd=Retrieve&db=pubmed&dopt=Abstract&list_uids=23399841|6|Cell Immunol|280|2|Dec|2012|Journal Article,Research Support, N.I.H., Extramural,Research Support, Non-U.S. Gov't
il8	ccl2	http://www.ncbi.nlm.nih.gov/entrez/query.fcgi?cmd=Retrieve&db=pubmed&dopt=Abstract&list_uids=23399841|6|Cell Immunol|280|2|Dec|2012|Journal Article,Research Support, N.I.H., Extramural,Research Support, Non-U.S. Gov't
il1b	mapk8	http://www.ncbi.nlm.nih.gov/entrez/query.fcgi?cmd=Retrieve&db=pubmed&dopt=Abstract&list_uids=23399841|6|Cell Immunol|280|2|Dec|2012|Journal Article,Research Support, N.I.H., Extramural,Research Support, Non-U.S. Gov't

il1b	fos	http://www.ncbi.nlm.nih.gov/entrez/query.fcgi?cmd=Retrieve&db=pubmed&dopt=Abstract&list_uids=23399841|6|Cell Immunol|280|2|Dec|2012|Journal Article,Research Support, N.I.H., Extramural,Research Support, Non-U.S. Gov't
mmp14	mmrn1	http://www.ncbi.nlm.nih.gov/entrez/query.fcgi?cmd=Retrieve&db=pubmed&dopt=Abstract&list_uids=22291036|6|J Cell Biol|196|3|Feb|2012|Journal Article,Research Support, N.I.H., Extramural|http://www.ncbi.nlm.nih.gov/entrez/query.fcgi?cmd=Retrieve&db=pubmed&dopt=Abstract&list_uids=23382803|6|PLoS One|8|1||2013|Journal Article,Research Support, Non-U.S. Gov't
usp7	tp53	http://www.ncbi.nlm.nih.gov/entrez/query.fcgi?cmd=Retrieve&db=pubmed&dopt=Abstract&list_uids=23382381|6|J Biol Chem|288|11|Mar|2013|Journal Article,Research Support, N.I.H., Extramural,Research Support, Non-U.S. Gov't
myh14	stim1	http://www.ncbi.nlm.nih.gov/entrez/query.fcgi?cmd=Retrieve&db=pubmed&dopt=Abstract&list_uids=23378028|6|J Cell Sci|126|Pt 5|Mar|2013|Journal Article,Research Support, Non-U.S. Gov't
actin	stim1	http://www.ncbi.nlm.nih.gov/entrez/query.fcgi?cmd=Retrieve&db=pubmed&dopt=Abstract&list_uids=23378028|6|J Cell Sci|126|Pt 5|Mar|2013|Journal Article,Research Support, Non-U.S. Gov't
3.6.4.1	stim1	http://www.ncbi.nlm.nih.gov/entrez/query.fcgi?cmd=Retrieve&db=pubmed&dopt=Abstract&list_uids=23378028|6|J Cell Sci|126|Pt 5|Mar|2013|Journal Article,Research Support, Non-U.S. Gov't
fas	cdkn1a	http://www.ncbi.nlm.nih.gov/entrez/query.fcgi?cmd=Retrieve&db=pubmed&dopt=Abstract&list_uids=23344455|6|Toxins (Basel)|5|1|Jan|2013|Comparative Study,Journal Article,Research Support, Non-U.S. Gov't
nanog	sox2	http://www.ncbi.nlm.nih.gov/entrez/query.fcgi?cmd=Retrieve&db=pubmed&dopt=Abstract&list_uids=23329853|6|J Mol Cell Biol|5|4|Aug|2013|Journal Article,Research Support, Non-U.S. Gov't
nanog	pou5f1	http://www.ncbi.nlm.nih.gov/entrez/query.fcgi?cmd=Retrieve&db=pubmed&dopt=Abstract&list_uids=23329853|6|J Mol Cell Biol|5|4|Aug|2013|Journal Article,Research Support, Non-U.S. Gov't
sox2	pou5f1	http://www.ncbi.nlm.nih.gov/entrez/query.fcgi?cmd=Retrieve&db=pubmed&dopt=Abstract&list_uids=23329853|6|J Mol Cell Biol|5|4|Aug|2013|Journal Article,Research Support, Non-U.S. Gov't
ctnnd1	rac1	http://www.ncbi.nlm.nih.gov/entrez/query.fcgi?cmd=Retrieve&db=pubmed&dopt=Abstract&list_uids=23325790|6|Mol Biol Cell|24|6|Mar|2013|Journal Article,Research Support, N.I.H., Extramural,Research Support, U.S. Gov't, Non-P.H.S.
frap1	gal	http://www.ncbi.nlm.nih.gov/entrez/query.fcgi?cmd=Retrieve&db=pubmed&dopt=Abstract&list_uids=23296673|6|Methods Mol Biol|965|||2013|Journal Article,Research Support, Non-U.S. Gov't
frap1	tp53	http://www.ncbi.nlm.nih.gov/entrez/query.fcgi?cmd=Retrieve&db=pubmed&dopt=Abstract&list_uids=23296673|6|Methods Mol Biol|965|||2013|Journal Article,Research Support, Non-U.S. Gov't
frap1	cdkn1a	http://www.ncbi.nlm.nih.gov/entrez/query.fcgi?cmd=Retrieve&db=pubmed&dopt=Abstract&list_uids=23296673|6|Methods Mol Biol|965|||2013|Journal Article,Research Support, Non-U.S. Gov't
frap1	cdkn2a	http://www.ncbi.nlm.nih.gov/entrez/query.fcgi?cmd=Retrieve&db=pubmed&dopt=Abstract&list_uids=23296673|6|Methods Mol Biol|965|||2013|Journal Article,Research Support, Non-U.S. Gov't
frap1	asprv1	http://www.ncbi.nlm.nih.gov/entrez/query.fcgi?cmd=Retrieve&db=pubmed&dopt=Abstract&list_uids=23296673|6|Methods Mol Biol|965|||2013|Journal Article,Research Support, Non-U.S. Gov't
gal	tp53	http://www.ncbi.nlm.nih.gov/entrez/query.fcgi?cmd=Retrieve&db=pubmed&dopt=Abstract&list_uids=23296673|6|Methods Mol Biol|965|||2013|Journal Article,Research Support, Non-U.S. Gov't

gal	cdkn1a	http://www.ncbi.nlm.nih.gov/entrez/query.fcgi?cmd=Retrieve&db=pubmed&dopt=Abstract&list_uids=23296673|6|Methods Mol Biol|965|||2013|Journal Article,Research Support, Non-U.S. Gov't
gal	cdkn2a	http://www.ncbi.nlm.nih.gov/entrez/query.fcgi?cmd=Retrieve&db=pubmed&dopt=Abstract&list_uids=23296673|6|Methods Mol Biol|965|||2013|Journal Article,Research Support, Non-U.S. Gov't
gal	asprv1	http://www.ncbi.nlm.nih.gov/entrez/query.fcgi?cmd=Retrieve&db=pubmed&dopt=Abstract&list_uids=23296673|6|Methods Mol Biol|965|||2013|Journal Article,Research Support, Non-U.S. Gov't
tp53	cdkn1a	http://www.ncbi.nlm.nih.gov/entrez/query.fcgi?cmd=Retrieve&db=pubmed&dopt=Abstract&list_uids=23296673|6|Methods Mol Biol|965|||2013|Journal Article,Research Support, Non-U.S. Gov't
tp53	cdkn2a	http://www.ncbi.nlm.nih.gov/entrez/query.fcgi?cmd=Retrieve&db=pubmed&dopt=Abstract&list_uids=23296673|6|Methods Mol Biol|965|||2013|Journal Article,Research Support, Non-U.S. Gov't
tp53	asprv1	http://www.ncbi.nlm.nih.gov/entrez/query.fcgi?cmd=Retrieve&db=pubmed&dopt=Abstract&list_uids=23296673|6|Methods Mol Biol|965|||2013|Journal Article,Research Support, Non-U.S. Gov't
cdkn1a	cdkn2a	http://www.ncbi.nlm.nih.gov/entrez/query.fcgi?cmd=Retrieve&db=pubmed&dopt=Abstract&list_uids=23296673|6|Methods Mol Biol|965|||2013|Journal Article,Research Support, Non-U.S. Gov't
cdkn1a	asprv1	http://www.ncbi.nlm.nih.gov/entrez/query.fcgi?cmd=Retrieve&db=pubmed&dopt=Abstract&list_uids=23296673|6|Methods Mol Biol|965|||2013|Journal Article,Research Support, Non-U.S. Gov't
cdkn2a	asprv1	http://www.ncbi.nlm.nih.gov/entrez/query.fcgi?cmd=Retrieve&db=pubmed&dopt=Abstract&list_uids=23296673|6|Methods Mol Biol|965|||2013|Journal Article,Research Support, Non-U.S. Gov't
cdkn2a	arap2	http://www.ncbi.nlm.nih.gov/entrez/query.fcgi?cmd=Retrieve&db=pubmed&dopt=Abstract&list_uids=23295182|6|J Biol Chem|288|8|Feb|2013|Journal Article,Research Support, N.I.H., Intramural
cdkn2a	arf6	http://www.ncbi.nlm.nih.gov/entrez/query.fcgi?cmd=Retrieve&db=pubmed&dopt=Abstract&list_uids=23295182|6|J Biol Chem|288|8|Feb|2013|Journal Article,Research Support, N.I.H., Intramural
cdkn2a	rasa1	http://www.ncbi.nlm.nih.gov/entrez/query.fcgi?cmd=Retrieve&db=pubmed&dopt=Abstract&list_uids=23295182|6|J Biol Chem|288|8|Feb|2013|Journal Article,Research Support, N.I.H., Intramural
cdkn2a	fas	http://www.ncbi.nlm.nih.gov/entrez/query.fcgi?cmd=Retrieve&db=pubmed&dopt=Abstract&list_uids=23295182|6|J Biol Chem|288|8|Feb|2013|Journal Article,Research Support, N.I.H., Intramural
arf6	fas	http://www.ncbi.nlm.nih.gov/entrez/query.fcgi?cmd=Retrieve&db=pubmed&dopt=Abstract&list_uids=23295182|6|J Biol Chem|288|8|Feb|2013|Journal Article,Research Support, N.I.H., Intramural
rasa1	fas	http://www.ncbi.nlm.nih.gov/entrez/query.fcgi?cmd=Retrieve&db=pubmed&dopt=Abstract&list_uids=23295182|6|J Biol Chem|288|8|Feb|2013|Journal Article,Research Support, N.I.H., Intramural
arap2	mtg1	http://www.ncbi.nlm.nih.gov/entrez/query.fcgi?cmd=Retrieve&db=pubmed&dopt=Abstract&list_uids=23295182|6|J Biol Chem|288|8|Feb|2013|Journal Article,Research Support, N.I.H., Intramural
arap2	rac1	http://www.ncbi.nlm.nih.gov/entrez/query.fcgi?cmd=Retrieve&db=pubmed&dopt=Abstract&list_uids=23295182|6|J Biol Chem|288|8|Feb|2013|Journal Article,Research Support, N.I.H., Intramural
fas	mtg1	http://www.ncbi.nlm.nih.gov/entrez/query.fcgi?cmd=Retrieve&db=pubmed&dopt=Abstract&list_uids=23295182|6|J Biol Chem|288|8|Feb|2013|Journal Article,Research Support, N.I.H., Intramural

mtg1	rac1	http://www.ncbi.nlm.nih.gov/entrez/query.fcgi?cmd=Retrieve&db=pubmed&dopt=Abstract&list_uids=23295182|6|J Biol Chem|288|8|Feb|2013|Journal Article,Research Support, N.I.H., Intramural|http://www.ncbi.nlm.nih.gov/entrez/query.fcgi?cmd=Retrieve&db=pubmed&dopt=Abstract&list_uids=22721990|6|Cardiovasc Res|95|3|Aug|2012|Journal Article,Research Support, Non-U.S. Gov't
arf6	rac1	http://www.ncbi.nlm.nih.gov/entrez/query.fcgi?cmd=Retrieve&db=pubmed&dopt=Abstract&list_uids=23295182|6|J Biol Chem|288|8|Feb|2013|Journal Article,Research Support, N.I.H., Intramural
vegfa	nrp1	http://www.ncbi.nlm.nih.gov/entrez/query.fcgi?cmd=Retrieve&db=pubmed&dopt=Abstract&list_uids=23288161|6|Arterioscler Thromb Vasc Biol|33|3|Mar|2013|Journal Article,Research Support, Non-U.S. Gov't
actin	nrp1	http://www.ncbi.nlm.nih.gov/entrez/query.fcgi?cmd=Retrieve&db=pubmed&dopt=Abstract&list_uids=23288161|6|Arterioscler Thromb Vasc Biol|33|3|Mar|2013|Journal Article,Research Support, Non-U.S. Gov't
gdf15	ctgf	http://www.ncbi.nlm.nih.gov/entrez/query.fcgi?cmd=Retrieve&db=pubmed&dopt=Abstract&list_uids=23280549|6|J Cell Biochem|114|6|Jun|2013|Journal Article,Research Support, Non-U.S. Gov't
gdf15	ptk2	http://www.ncbi.nlm.nih.gov/entrez/query.fcgi?cmd=Retrieve&db=pubmed&dopt=Abstract&list_uids=23280549|6|J Cell Biochem|114|6|Jun|2013|Journal Article,Research Support, Non-U.S. Gov't
wasf1	wasl	http://www.ncbi.nlm.nih.gov/entrez/query.fcgi?cmd=Retrieve&db=pubmed&dopt=Abstract&list_uids=23273897|6|Curr Biol|23|2|Jan|2013|Journal Article,Research Support, Non-U.S. Gov't
wasf1	ptk2	http://www.ncbi.nlm.nih.gov/entrez/query.fcgi?cmd=Retrieve&db=pubmed&dopt=Abstract&list_uids=23273897|6|Curr Biol|23|2|Jan|2013|Journal Article,Research Support, Non-U.S. Gov't
wasl	ptk2	http://www.ncbi.nlm.nih.gov/entrez/query.fcgi?cmd=Retrieve&db=pubmed&dopt=Abstract&list_uids=23273897|6|Curr Biol|23|2|Jan|2013|Journal Article,Research Support, Non-U.S. Gov't
wasf1	actr2	http://www.ncbi.nlm.nih.gov/entrez/query.fcgi?cmd=Retrieve&db=pubmed&dopt=Abstract&list_uids=23273897|6|Curr Biol|23|2|Jan|2013|Journal Article,Research Support, Non-U.S. Gov't
ptk2	actr2	http://www.ncbi.nlm.nih.gov/entrez/query.fcgi?cmd=Retrieve&db=pubmed&dopt=Abstract&list_uids=23273897|6|Curr Biol|23|2|Jan|2013|Journal Article,Research Support, Non-U.S. Gov't
actr2	actin	http://www.ncbi.nlm.nih.gov/entrez/query.fcgi?cmd=Retrieve&db=pubmed&dopt=Abstract&list_uids=23273897|6|Curr Biol|23|2|Jan|2013|Journal Article,Research Support, Non-U.S. Gov't
col2a1	bmp2	http://www.ncbi.nlm.nih.gov/entrez/query.fcgi?cmd=Retrieve&db=pubmed&dopt=Abstract&list_uids=23270543|6|Tissue Eng Part C Methods|19|7|Jul|2013|Journal Article,Research Support, Non-U.S. Gov't
col2a1	sox9	http://www.ncbi.nlm.nih.gov/entrez/query.fcgi?cmd=Retrieve&db=pubmed&dopt=Abstract&list_uids=23270543|6|Tissue Eng Part C Methods|19|7|Jul|2013|Journal Article,Research Support, Non-U.S. Gov't
col2a1	sox5	http://www.ncbi.nlm.nih.gov/entrez/query.fcgi?cmd=Retrieve&db=pubmed&dopt=Abstract&list_uids=23270543|6|Tissue Eng Part C Methods|19|7|Jul|2013|Journal Article,Research Support, Non-U.S. Gov't
col2a1	sox6	http://www.ncbi.nlm.nih.gov/entrez/query.fcgi?cmd=Retrieve&db=pubmed&dopt=Abstract&list_uids=23270543|6|Tissue Eng Part C Methods|19|7|Jul|2013|Journal Article,Research Support, Non-U.S. Gov't
col2a1	sp1	http://www.ncbi.nlm.nih.gov/entrez/query.fcgi?cmd=Retrieve&db=pubmed&dopt=Abstract&list_uids=23270543|6|Tissue Eng Part C Methods|19|7|Jul|2013|Journal Article,Research Support, Non-U.S. Gov't

col2a1	zbtb7b	http://www.ncbi.nlm.nih.gov/entrez/query.fcgi?cmd=Retrieve&db=pubmed&dopt=Abstract&list_uids=23270543|6|Tissue Eng Part C Methods|19|7|Jul|2013|Journal Article,Research Support, Non-U.S. Gov't
col2a1	rela	http://www.ncbi.nlm.nih.gov/entrez/query.fcgi?cmd=Retrieve&db=pubmed&dopt=Abstract&list_uids=23270543|6|Tissue Eng Part C Methods|19|7|Jul|2013|Journal Article,Research Support, Non-U.S. Gov't
col2a1	nf-kappab	http://www.ncbi.nlm.nih.gov/entrez/query.fcgi?cmd=Retrieve&db=pubmed&dopt=Abstract&list_uids=23270543|6|Tissue Eng Part C Methods|19|7|Jul|2013|Journal Article,Research Support, Non-U.S. Gov't
bmp2	sox9	http://www.ncbi.nlm.nih.gov/entrez/query.fcgi?cmd=Retrieve&db=pubmed&dopt=Abstract&list_uids=23270543|6|Tissue Eng Part C Methods|19|7|Jul|2013|Journal Article,Research Support, Non-U.S. Gov't
bmp2	sox5	http://www.ncbi.nlm.nih.gov/entrez/query.fcgi?cmd=Retrieve&db=pubmed&dopt=Abstract&list_uids=23270543|6|Tissue Eng Part C Methods|19|7|Jul|2013|Journal Article,Research Support, Non-U.S. Gov't
bmp2	sox6	http://www.ncbi.nlm.nih.gov/entrez/query.fcgi?cmd=Retrieve&db=pubmed&dopt=Abstract&list_uids=23270543|6|Tissue Eng Part C Methods|19|7|Jul|2013|Journal Article,Research Support, Non-U.S. Gov't
bmp2	sp1	http://www.ncbi.nlm.nih.gov/entrez/query.fcgi?cmd=Retrieve&db=pubmed&dopt=Abstract&list_uids=23270543|6|Tissue Eng Part C Methods|19|7|Jul|2013|Journal Article,Research Support, Non-U.S. Gov't
bmp2	zbtb7b	http://www.ncbi.nlm.nih.gov/entrez/query.fcgi?cmd=Retrieve&db=pubmed&dopt=Abstract&list_uids=23270543|6|Tissue Eng Part C Methods|19|7|Jul|2013|Journal Article,Research Support, Non-U.S. Gov't
bmp2	rela	http://www.ncbi.nlm.nih.gov/entrez/query.fcgi?cmd=Retrieve&db=pubmed&dopt=Abstract&list_uids=23270543|6|Tissue Eng Part C Methods|19|7|Jul|2013|Journal Article,Research Support, Non-U.S. Gov't
bmp2	nf-kappab	http://www.ncbi.nlm.nih.gov/entrez/query.fcgi?cmd=Retrieve&db=pubmed&dopt=Abstract&list_uids=23270543|6|Tissue Eng Part C Methods|19|7|Jul|2013|Journal Article,Research Support, Non-U.S. Gov't
sox9	sox5	http://www.ncbi.nlm.nih.gov/entrez/query.fcgi?cmd=Retrieve&db=pubmed&dopt=Abstract&list_uids=23270543|6|Tissue Eng Part C Methods|19|7|Jul|2013|Journal Article,Research Support, Non-U.S. Gov't
sox9	sox6	http://www.ncbi.nlm.nih.gov/entrez/query.fcgi?cmd=Retrieve&db=pubmed&dopt=Abstract&list_uids=23270543|6|Tissue Eng Part C Methods|19|7|Jul|2013|Journal Article,Research Support, Non-U.S. Gov't
sox9	sp1	http://www.ncbi.nlm.nih.gov/entrez/query.fcgi?cmd=Retrieve&db=pubmed&dopt=Abstract&list_uids=23270543|6|Tissue Eng Part C Methods|19|7|Jul|2013|Journal Article,Research Support, Non-U.S. Gov't
sox9	zbtb7b	http://www.ncbi.nlm.nih.gov/entrez/query.fcgi?cmd=Retrieve&db=pubmed&dopt=Abstract&list_uids=23270543|6|Tissue Eng Part C Methods|19|7|Jul|2013|Journal Article,Research Support, Non-U.S. Gov't
sox9	rela	http://www.ncbi.nlm.nih.gov/entrez/query.fcgi?cmd=Retrieve&db=pubmed&dopt=Abstract&list_uids=23270543|6|Tissue Eng Part C Methods|19|7|Jul|2013|Journal Article,Research Support, Non-U.S. Gov't
sox9	nf-kappab	http://www.ncbi.nlm.nih.gov/entrez/query.fcgi?cmd=Retrieve&db=pubmed&dopt=Abstract&list_uids=23270543|6|Tissue Eng Part C Methods|19|7|Jul|2013|Journal Article,Research Support, Non-U.S. Gov't
sox5	sox6	http://www.ncbi.nlm.nih.gov/entrez/query.fcgi?cmd=Retrieve&db=pubmed&dopt=Abstract&list_uids=23270543|6|Tissue Eng Part C Methods|19|7|Jul|2013|Journal Article,Research Support, Non-U.S. Gov't
sox5	sp1	http://www.ncbi.nlm.nih.gov/entrez/query.fcgi?cmd=Retrieve&db=pubmed&dopt=Abstract&list_uids=23270543|6|Tissue Eng Part C Methods|19|7|Jul|2013|Journal Article,Research Support, Non-U.S. Gov't

sox5	zbtb7b	http://www.ncbi.nlm.nih.gov/entrez/query.fcgi?cmd=Retrieve&db=pubmed&dopt=Abstract&list_uids=23270543|6|Tissue Eng Part C Methods|19|7|Jul|2013|Journal Article,Research Support, Non-U.S. Gov't
sox5	rela	http://www.ncbi.nlm.nih.gov/entrez/query.fcgi?cmd=Retrieve&db=pubmed&dopt=Abstract&list_uids=23270543|6|Tissue Eng Part C Methods|19|7|Jul|2013|Journal Article,Research Support, Non-U.S. Gov't
sox5	nf-kappab	http://www.ncbi.nlm.nih.gov/entrez/query.fcgi?cmd=Retrieve&db=pubmed&dopt=Abstract&list_uids=23270543|6|Tissue Eng Part C Methods|19|7|Jul|2013|Journal Article,Research Support, Non-U.S. Gov't
sox6	sp1	http://www.ncbi.nlm.nih.gov/entrez/query.fcgi?cmd=Retrieve&db=pubmed&dopt=Abstract&list_uids=23270543|6|Tissue Eng Part C Methods|19|7|Jul|2013|Journal Article,Research Support, Non-U.S. Gov't
sox6	zbtb7b	http://www.ncbi.nlm.nih.gov/entrez/query.fcgi?cmd=Retrieve&db=pubmed&dopt=Abstract&list_uids=23270543|6|Tissue Eng Part C Methods|19|7|Jul|2013|Journal Article,Research Support, Non-U.S. Gov't
sox6	rela	http://www.ncbi.nlm.nih.gov/entrez/query.fcgi?cmd=Retrieve&db=pubmed&dopt=Abstract&list_uids=23270543|6|Tissue Eng Part C Methods|19|7|Jul|2013|Journal Article,Research Support, Non-U.S. Gov't
sox6	nf-kappab	http://www.ncbi.nlm.nih.gov/entrez/query.fcgi?cmd=Retrieve&db=pubmed&dopt=Abstract&list_uids=23270543|6|Tissue Eng Part C Methods|19|7|Jul|2013|Journal Article,Research Support, Non-U.S. Gov't
sp1	zbtb7b	http://www.ncbi.nlm.nih.gov/entrez/query.fcgi?cmd=Retrieve&db=pubmed&dopt=Abstract&list_uids=23270543|6|Tissue Eng Part C Methods|19|7|Jul|2013|Journal Article,Research Support, Non-U.S. Gov't
sp1	rela	http://www.ncbi.nlm.nih.gov/entrez/query.fcgi?cmd=Retrieve&db=pubmed&dopt=Abstract&list_uids=23270543|6|Tissue Eng Part C Methods|19|7|Jul|2013|Journal Article,Research Support, Non-U.S. Gov't
sp1	nf-kappab	http://www.ncbi.nlm.nih.gov/entrez/query.fcgi?cmd=Retrieve&db=pubmed&dopt=Abstract&list_uids=23270543|6|Tissue Eng Part C Methods|19|7|Jul|2013|Journal Article,Research Support, Non-U.S. Gov't
zbtb7b	rela	http://www.ncbi.nlm.nih.gov/entrez/query.fcgi?cmd=Retrieve&db=pubmed&dopt=Abstract&list_uids=23270543|6|Tissue Eng Part C Methods|19|7|Jul|2013|Journal Article,Research Support, Non-U.S. Gov't
zbtb7b	nf-kappab	http://www.ncbi.nlm.nih.gov/entrez/query.fcgi?cmd=Retrieve&db=pubmed&dopt=Abstract&list_uids=23270543|6|Tissue Eng Part C Methods|19|7|Jul|2013|Journal Article,Research Support, Non-U.S. Gov't
rela	nf-kappab	http://www.ncbi.nlm.nih.gov/entrez/query.fcgi?cmd=Retrieve&db=pubmed&dopt=Abstract&list_uids=23270543|6|Tissue Eng Part C Methods|19|7|Jul|2013|Journal Article,Research Support, Non-U.S. Gov't
myh14	kif1c	http://www.ncbi.nlm.nih.gov/entrez/query.fcgi?cmd=Retrieve&db=pubmed&dopt=Abstract&list_uids=23237952|6|Dev Cell|23|6|Dec|2012|Journal Article,Research Support, Non-U.S. Gov't
kat5	gtse1	http://www.ncbi.nlm.nih.gov/entrez/query.fcgi?cmd=Retrieve&db=pubmed&dopt=Abstract&list_uids=23236459|6|PLoS One|7|12||2012|Journal Article,Research Support, Non-U.S. Gov't
epor	jak2	http://www.ncbi.nlm.nih.gov/entrez/query.fcgi?cmd=Retrieve&db=pubmed&dopt=Abstract&list_uids=23221524|6|J Microbiol Biotechnol|22|12|Dec|2012|Journal Article,Research Support, Non-U.S. Gov't
epor	stat5a	http://www.ncbi.nlm.nih.gov/entrez/query.fcgi?cmd=Retrieve&db=pubmed&dopt=Abstract&list_uids=23221524|6|J Microbiol Biotechnol|22|12|Dec|2012|Journal Article,Research Support, Non-U.S. Gov't
jak2	stat5a	http://www.ncbi.nlm.nih.gov/entrez/query.fcgi?cmd=Retrieve&db=pubmed&dopt=Abstract&list_uids=23221524|6|J Microbiol Biotechnol|22|12|Dec|2012|Journal Article,Research Support, Non-U.S. Gov't

stim1	hcc	http://www.ncbi.nlm.nih.gov/entrez/query.fcgi?cmd=Retrieve&db=pubmed&dopt=Abstract&list_uids=23211538|6|Cancer Lett|330|2|Apr|2013|Journal Article,Research Support, Non-U.S. Gov't
rap1a	rapgef3	http://www.ncbi.nlm.nih.gov/entrez/query.fcgi?cmd=Retrieve&db=pubmed&dopt=Abstract&list_uids=23209645|6|PLoS One|7|11||2012|Journal Article,Research Support, Non-U.S. Gov't
src	rap1a	http://www.ncbi.nlm.nih.gov/entrez/query.fcgi?cmd=Retrieve&db=pubmed&dopt=Abstract&list_uids=22499769|6|Mol Cell Proteomics|11|8|Aug|2012|Journal Article,Research Support, N.I.H., Extramural,Research Support, Non-U.S. Gov't|http://www.ncbi.nlm.nih.gov/entrez/query.fcgi?cmd=Retrieve&db=pubmed&dopt=Abstract&list_uids=23209645|6|PLoS One|7|11||2012|Journal Article,Research Support, Non-U.S. Gov't
ptk2	rap1a	http://www.ncbi.nlm.nih.gov/entrez/query.fcgi?cmd=Retrieve&db=pubmed&dopt=Abstract&list_uids=23209645|6|PLoS One|7|11||2012|Journal Article,Research Support, Non-U.S. Gov't
ptk2	3.6.4.1	http://www.ncbi.nlm.nih.gov/entrez/query.fcgi?cmd=Retrieve&db=pubmed&dopt=Abstract&list_uids=23209645|6|PLoS One|7|11||2012|Journal Article,Research Support, Non-U.S. Gov't
rap1a	3.6.4.1	http://www.ncbi.nlm.nih.gov/entrez/query.fcgi?cmd=Retrieve&db=pubmed&dopt=Abstract&list_uids=23209645|6|PLoS One|7|11||2012|Journal Article,Research Support, Non-U.S. Gov't
ctsh	pc-3	http://www.ncbi.nlm.nih.gov/entrez/query.fcgi?cmd=Retrieve&db=pubmed&dopt=Abstract&list_uids=23204516|6|J Biol Chem|288|4|Jan|2013|Journal Article,Research Support, Non-U.S. Gov't
gnrh1	gnrhr	http://www.ncbi.nlm.nih.gov/entrez/query.fcgi?cmd=Retrieve&db=pubmed&dopt=Abstract&list_uids=23176180|6|BMC Cancer|12|||2012|Journal Article,Research Support, Non-U.S. Gov't
actin	gnrh1	http://www.ncbi.nlm.nih.gov/entrez/query.fcgi?cmd=Retrieve&db=pubmed&dopt=Abstract&list_uids=23176180|6|BMC Cancer|12|||2012|Journal Article,Research Support, Non-U.S. Gov't
gnrh1	rhoa	http://www.ncbi.nlm.nih.gov/entrez/query.fcgi?cmd=Retrieve&db=pubmed&dopt=Abstract&list_uids=23176180|6|BMC Cancer|12|||2012|Journal Article,Research Support, Non-U.S. Gov't
itgal	orai1	http://www.ncbi.nlm.nih.gov/entrez/query.fcgi?cmd=Retrieve&db=pubmed&dopt=Abstract&list_uids=23144497|6|J Immunol|189|12|Dec|2012|Journal Article
il8	itgal	http://www.ncbi.nlm.nih.gov/entrez/query.fcgi?cmd=Retrieve&db=pubmed&dopt=Abstract&list_uids=23144497|6|J Immunol|189|12|Dec|2012|Journal Article
il8	icam1	http://www.ncbi.nlm.nih.gov/entrez/query.fcgi?cmd=Retrieve&db=pubmed&dopt=Abstract&list_uids=23144497|6|J Immunol|189|12|Dec|2012|Journal Article
itgal	icam1	http://www.ncbi.nlm.nih.gov/entrez/query.fcgi?cmd=Retrieve&db=pubmed&dopt=Abstract&list_uids=23144497|6|J Immunol|189|12|Dec|2012|Journal Article
myh14	3.6.1.8	http://www.ncbi.nlm.nih.gov/entrez/query.fcgi?cmd=Retrieve&db=pubmed&dopt=Abstract&list_uids=23132927|6|J Cell Sci|126|Pt 1|Jan|2013|Journal Article,Research Support, Non-U.S. Gov't
slk	ptk2	http://www.ncbi.nlm.nih.gov/entrez/query.fcgi?cmd=Retrieve&db=pubmed&dopt=Abstract&list_uids=23128389|6|Oncogene|32|39|Sep|2013|Journal Article,Research Support, Non-U.S. Gov't
rps6ka3	zhx2	http://www.ncbi.nlm.nih.gov/entrez/query.fcgi?cmd=Retrieve&db=pubmed&dopt=Abstract&list_uids=23118220|6|J Biol Chem|287|52|Dec|2012|Journal Article,Research Support, N.I.H., Extramural,Research Support, Non-U.S. Gov't,Research

		Support, U.S. Gov't, Non-P.H.S.
cav1	src	http://www.ncbi.nlm.nih.gov/entrez/query.fcgi?cmd=Retrieve&db=pubmed&dopt=Abstract&list_uids=23091071|6|J Cell Biol|199|3|Oct|2012|Journal Article,Research Support, Non-U.S. Gov't
cav1	rbm8a	http://www.ncbi.nlm.nih.gov/entrez/query.fcgi?cmd=Retrieve&db=pubmed&dopt=Abstract&list_uids=23091071|6|J Cell Biol|199|3|Oct|2012|Journal Article,Research Support, Non-U.S. Gov't
src	rbm8a	http://www.ncbi.nlm.nih.gov/entrez/query.fcgi?cmd=Retrieve&db=pubmed&dopt=Abstract&list_uids=23091071|6|J Cell Biol|199|3|Oct|2012|Journal Article,Research Support, Non-U.S. Gov't
cav1	actin	http://www.ncbi.nlm.nih.gov/entrez/query.fcgi?cmd=Retrieve&db=pubmed&dopt=Abstract&list_uids=23091071|6|J Cell Biol|199|3|Oct|2012|Journal Article,Research Support, Non-U.S. Gov't
cav1	egr1	http://www.ncbi.nlm.nih.gov/entrez/query.fcgi?cmd=Retrieve&db=pubmed&dopt=Abstract&list_uids=23091071|6|J Cell Biol|199|3|Oct|2012|Journal Article,Research Support, Non-U.S. Gov't
actin	egr1	http://www.ncbi.nlm.nih.gov/entrez/query.fcgi?cmd=Retrieve&db=pubmed&dopt=Abstract&list_uids=23091071|6|J Cell Biol|199|3|Oct|2012|Journal Article,Research Support, Non-U.S. Gov't
cav1	pkc	http://www.ncbi.nlm.nih.gov/entrez/query.fcgi?cmd=Retrieve&db=pubmed&dopt=Abstract&list_uids=23091071|6|J Cell Biol|199|3|Oct|2012|Journal Article,Research Support, Non-U.S. Gov't
egr1	src	http://www.ncbi.nlm.nih.gov/entrez/query.fcgi?cmd=Retrieve&db=pubmed&dopt=Abstract&list_uids=23091071|6|J Cell Biol|199|3|Oct|2012|Journal Article,Research Support, Non-U.S. Gov't
egr1	pkc	http://www.ncbi.nlm.nih.gov/entrez/query.fcgi?cmd=Retrieve&db=pubmed&dopt=Abstract&list_uids=23091071|6|J Cell Biol|199|3|Oct|2012|Journal Article,Research Support, Non-U.S. Gov't
actin	pkc	http://www.ncbi.nlm.nih.gov/entrez/query.fcgi?cmd=Retrieve&db=pubmed&dopt=Abstract&list_uids=23091071|6|J Cell Biol|199|3|Oct|2012|Journal Article,Research Support, Non-U.S. Gov't
src	pkc	http://www.ncbi.nlm.nih.gov/entrez/query.fcgi?cmd=Retrieve&db=pubmed&dopt=Abstract&list_uids=23091071|6|J Cell Biol|199|3|Oct|2012|Journal Article,Research Support, Non-U.S. Gov't
tfap2a	clathrin	http://www.ncbi.nlm.nih.gov/entrez/query.fcgi?cmd=Retrieve&db=pubmed&dopt=Abstract&list_uids=23056266|6|PLoS One|7|10||2012|Journal Article,Research Support, N.I.H., Extramural
fas	orai1	http://www.ncbi.nlm.nih.gov/entrez/query.fcgi?cmd=Retrieve&db=pubmed&dopt=Abstract&list_uids=23043102|6|J Biol Chem|287|48|Nov|2012|Journal Article,Research Support, N.I.H., Extramural,Research Support, Non-U.S. Gov't
fas	pla2g6	http://www.ncbi.nlm.nih.gov/entrez/query.fcgi?cmd=Retrieve&db=pubmed&dopt=Abstract&list_uids=23043102|6|J Biol Chem|287|48|Nov|2012|Journal Article,Research Support, N.I.H., Extramural,Research Support, Non-U.S. Gov't
fas	stim1	http://www.ncbi.nlm.nih.gov/entrez/query.fcgi?cmd=Retrieve&db=pubmed&dopt=Abstract&list_uids=23043102|6|J Biol Chem|287|48|Nov|2012|Journal Article,Research Support, N.I.H., Extramural,Research Support, Non-U.S. Gov't
orai1	pla2g6	http://www.ncbi.nlm.nih.gov/entrez/query.fcgi?cmd=Retrieve&db=pubmed&dopt=Abstract&list_uids=23043102|6|J Biol Chem|287|48|Nov|2012|Journal Article,Research Support, N.I.H., Extramural,Research Support, Non-U.S. Gov't
orai1	stim1	http://www.ncbi.nlm.nih.gov/entrez/query.fcgi?cmd=Retrieve&db=pubmed&dopt=Abstract&list_uids=23043102|6|J Biol Chem|287|48|Nov|2012|Journal Article,Research Support, N.I.H., Extramural,Research Support, Non-U.S. Gov't

pla2g6	stim1	http://www.ncbi.nlm.nih.gov/entrez/query.fcgi?cmd=Retrieve&db=pubmed&dopt=Abstract&list_uids=23043102|6|J Biol Chem|287|48|Nov|2012|Journal Article,Research Support, N.I.H., Extramural,Research Support, Non-U.S. Gov't
frizzled	sfrp1	http://www.ncbi.nlm.nih.gov/entrez/query.fcgi?cmd=Retrieve&db=pubmed&dopt=Abstract&list_uids=23028669|6|PLoS One|7|9||2012|Journal Article,Research Support, N.I.H., Extramural
myoc	top	http://www.ncbi.nlm.nih.gov/entrez/query.fcgi?cmd=Retrieve&db=pubmed&dopt=Abstract&list_uids=23028669|6|PLoS One|7|9||2012|Journal Article,Research Support, N.I.H., Extramural
myoc	fop	http://www.ncbi.nlm.nih.gov/entrez/query.fcgi?cmd=Retrieve&db=pubmed&dopt=Abstract&list_uids=23028669|6|PLoS One|7|9||2012|Journal Article,Research Support, N.I.H., Extramural
top	fop	http://www.ncbi.nlm.nih.gov/entrez/query.fcgi?cmd=Retrieve&db=pubmed&dopt=Abstract&list_uids=23028669|6|PLoS One|7|9||2012|Journal Article,Research Support, N.I.H., Extramural
myoc	actin	http://www.ncbi.nlm.nih.gov/entrez/query.fcgi?cmd=Retrieve&db=pubmed&dopt=Abstract&list_uids=23028669|6|PLoS One|7|9||2012|Journal Article,Research Support, N.I.H., Extramural
myoc	rhoa	http://www.ncbi.nlm.nih.gov/entrez/query.fcgi?cmd=Retrieve&db=pubmed&dopt=Abstract&list_uids=23028669|6|PLoS One|7|9||2012|Journal Article,Research Support, N.I.H., Extramural
sfrp1	myoc	http://www.ncbi.nlm.nih.gov/entrez/query.fcgi?cmd=Retrieve&db=pubmed&dopt=Abstract&list_uids=23028669|6|PLoS One|7|9||2012|Journal Article,Research Support, N.I.H., Extramural
igf1	egf	http://www.ncbi.nlm.nih.gov/entrez/query.fcgi?cmd=Retrieve&db=pubmed&dopt=Abstract&list_uids=23023326|6|Int J Oncol|41|6|Dec|2012|Journal Article,Research Support, Non-U.S. Gov't
apc	akt1	http://www.ncbi.nlm.nih.gov/entrez/query.fcgi?cmd=Retrieve&db=pubmed&dopt=Abstract&list_uids=23011394|6|Circ Res|111|12|Dec|2012|Journal Article,Research Support, N.I.H., Extramural,Research Support, Non-U.S. Gov't
pik3ca	src	http://www.ncbi.nlm.nih.gov/entrez/query.fcgi?cmd=Retrieve&db=pubmed&dopt=Abstract&list_uids=22969158|6|Sci Signal|5|241||2012|Journal Article,Research Support, N.I.H., Extramural,Research Support, Non-U.S. Gov't
pik3ca	inppl1	http://www.ncbi.nlm.nih.gov/entrez/query.fcgi?cmd=Retrieve&db=pubmed&dopt=Abstract&list_uids=22969158|6|Sci Signal|5|241||2012|Journal Article,Research Support, N.I.H., Extramural,Research Support, Non-U.S. Gov't
src	inppl1	http://www.ncbi.nlm.nih.gov/entrez/query.fcgi?cmd=Retrieve&db=pubmed&dopt=Abstract&list_uids=22969158|6|Sci Signal|5|241||2012|Journal Article,Research Support, N.I.H., Extramural,Research Support, Non-U.S. Gov't
pik3ca	pten	http://www.ncbi.nlm.nih.gov/entrez/query.fcgi?cmd=Retrieve&db=pubmed&dopt=Abstract&list_uids=22969158|6|Sci Signal|5|241||2012|Journal Article,Research Support, N.I.H., Extramural,Research Support, Non-U.S. Gov't
mapk3	mapk9	http://www.ncbi.nlm.nih.gov/entrez/query.fcgi?cmd=Retrieve&db=pubmed&dopt=Abstract&list_uids=22966240|6|Clin Dev Immunol|2012|||2012|Journal Article,Research Support, Non-U.S. Gov't
mapk3	mapk8	http://www.ncbi.nlm.nih.gov/entrez/query.fcgi?cmd=Retrieve&db=pubmed&dopt=Abstract&list_uids=22966240|6|Clin Dev Immunol|2012|||2012|Journal Article,Research Support, Non-U.S. Gov't
mapk9	mapk8	http://www.ncbi.nlm.nih.gov/entrez/query.fcgi?cmd=Retrieve&db=pubmed&dopt=Abstract&list_uids=22966240|6|Clin Dev Immunol|2012|||2012|Journal Article,Research Support, Non-U.S. Gov't
flna	actin	http://www.ncbi.nlm.nih.gov/entrez/query.fcgi?cmd=Retrieve&db=pubmed&dopt=Abstract&list_uids=22956851|6|J Neurosci|32|36|Sep|2012|Journal Article,Research Support, N.I.H., Extramural,Research Support, Non-U.S. Gov't,Research

		Support, U.S. Gov't, Non-P.H.S.
arfgef2	actin	http://www.ncbi.nlm.nih.gov/entrez/query.fcgi?cmd=Retrieve&db=pubmed&dopt=Abstract&list_uids=22956851|6|J Neurosci|32|36|Sep|2012|Journal Article,Research Support, N.I.H., Extramural,Research Support, Non-U.S. Gov't,Research Support, U.S. Gov't, Non-P.H.S.
fam123a	arhgef2	http://www.ncbi.nlm.nih.gov/entrez/query.fcgi?cmd=Retrieve&db=pubmed&dopt=Abstract&list_uids=22949735|6|Sci Signal|5|240|Sep|2012|Comparative Study,Journal Article,Research Support, N.I.H., Extramural,Research Support, Non-U.S. Gov't
fam123a	3.6.4.1	http://www.ncbi.nlm.nih.gov/entrez/query.fcgi?cmd=Retrieve&db=pubmed&dopt=Abstract&list_uids=22949735|6|Sci Signal|5|240|Sep|2012|Comparative Study,Journal Article,Research Support, N.I.H., Extramural,Research Support, Non-U.S. Gov't
arhgef2	3.6.4.1	http://www.ncbi.nlm.nih.gov/entrez/query.fcgi?cmd=Retrieve&db=pubmed&dopt=Abstract&list_uids=22949735|6|Sci Signal|5|240|Sep|2012|Comparative Study,Journal Article,Research Support, N.I.H., Extramural,Research Support, Non-U.S. Gov't
snw1	fam123a	http://www.ncbi.nlm.nih.gov/entrez/query.fcgi?cmd=Retrieve&db=pubmed&dopt=Abstract&list_uids=22949735|6|Sci Signal|5|240|Sep|2012|Comparative Study,Journal Article,Research Support, N.I.H., Extramural,Research Support, Non-U.S. Gov't
snw1	cox7a2l	http://www.ncbi.nlm.nih.gov/entrez/query.fcgi?cmd=Retrieve&db=pubmed&dopt=Abstract&list_uids=22949735|6|Sci Signal|5|240|Sep|2012|Comparative Study,Journal Article,Research Support, N.I.H., Extramural,Research Support, Non-U.S. Gov't
snw1	mapre3	http://www.ncbi.nlm.nih.gov/entrez/query.fcgi?cmd=Retrieve&db=pubmed&dopt=Abstract&list_uids=22949735|6|Sci Signal|5|240|Sep|2012|Comparative Study,Journal Article,Research Support, N.I.H., Extramural,Research Support, Non-U.S. Gov't
fam123a	cox7a2l	http://www.ncbi.nlm.nih.gov/entrez/query.fcgi?cmd=Retrieve&db=pubmed&dopt=Abstract&list_uids=22949735|6|Sci Signal|5|240|Sep|2012|Comparative Study,Journal Article,Research Support, N.I.H., Extramural,Research Support, Non-U.S. Gov't
fam123a	mapre3	http://www.ncbi.nlm.nih.gov/entrez/query.fcgi?cmd=Retrieve&db=pubmed&dopt=Abstract&list_uids=22949735|6|Sci Signal|5|240|Sep|2012|Comparative Study,Journal Article,Research Support, N.I.H., Extramural,Research Support, Non-U.S. Gov't
fam123a	rhoa	http://www.ncbi.nlm.nih.gov/entrez/query.fcgi?cmd=Retrieve&db=pubmed&dopt=Abstract&list_uids=22949735|6|Sci Signal|5|240|Sep|2012|Comparative Study,Journal Article,Research Support, N.I.H., Extramural,Research Support, Non-U.S. Gov't
snw1	3.6.4.1	http://www.ncbi.nlm.nih.gov/entrez/query.fcgi?cmd=Retrieve&db=pubmed&dopt=Abstract&list_uids=22949735|6|Sci Signal|5|240|Sep|2012|Comparative Study,Journal Article,Research Support, N.I.H., Extramural,Research Support, Non-U.S. Gov't
apbb1ip	mapk3	http://www.ncbi.nlm.nih.gov/entrez/query.fcgi?cmd=Retrieve&db=pubmed&dopt=Abstract&list_uids=22946047|6|J Cell Sci|125|Pt 22|Nov|2012|Journal Article,Research Support, Non-U.S. Gov't

apbb1ip	rhoa	http://www.ncbi.nlm.nih.gov/entrez/query.fcgi?cmd=Retrieve&db=pubmed&dopt=Abstract&list_uids=22946047|6|J Cell Sci|125|Pt 22|Nov|2012|Journal Article,Research Support, Non-U.S. Gov't
uchl1	ptk2	http://www.ncbi.nlm.nih.gov/entrez/query.fcgi?cmd=Retrieve&db=pubmed&dopt=Abstract&list_uids=22932395|6|FASEB J|26|12|Dec|2012|Journal Article,Research Support, Non-U.S. Gov't
uchl1	akt1	http://www.ncbi.nlm.nih.gov/entrez/query.fcgi?cmd=Retrieve&db=pubmed&dopt=Abstract&list_uids=22932395|6|FASEB J|26|12|Dec|2012|Journal Article,Research Support, Non-U.S. Gov't
itk	akt1	http://www.ncbi.nlm.nih.gov/entrez/query.fcgi?cmd=Retrieve&db=pubmed&dopt=Abstract&list_uids=22918955|6|Mol Biol Cell|23|20|Oct|2012|Journal Article,Research Support, N.I.H., Extramural,Research Support, Non-U.S. Gov't
decr1	itk	http://www.ncbi.nlm.nih.gov/entrez/query.fcgi?cmd=Retrieve&db=pubmed&dopt=Abstract&list_uids=22918955|6|Mol Biol Cell|23|20|Oct|2012|Journal Article,Research Support, N.I.H., Extramural,Research Support, Non-U.S. Gov't
ptk2	crk	http://www.ncbi.nlm.nih.gov/entrez/query.fcgi?cmd=Retrieve&db=pubmed&dopt=Abstract&list_uids=22915102|6|J Neurosci|32|34|Aug|2012|Journal Article,Research Support, N.I.H., Extramural,Research Support, Non-U.S. Gov't,Validation Studies
ptk2	cse1l	http://www.ncbi.nlm.nih.gov/entrez/query.fcgi?cmd=Retrieve&db=pubmed&dopt=Abstract&list_uids=22287273|6|J Cell Physiol|227|11|Nov|2012|Journal Article,Research Support, N.I.H., Extramural|http://www.ncbi.nlm.nih.gov/entrez/query.fcgi?cmd=Retrieve&db=pubmed&dopt=Abstract&list_uids=22915102|6|J Neurosci|32|34|Aug|2012|Journal Article,Research Support, N.I.H., Extramural,Research Support, Non-U.S. Gov't,Validation Studies
crk	cse1l	http://www.ncbi.nlm.nih.gov/entrez/query.fcgi?cmd=Retrieve&db=pubmed&dopt=Abstract&list_uids=22915102|6|J Neurosci|32|34|Aug|2012|Journal Article,Research Support, N.I.H., Extramural,Research Support, Non-U.S. Gov't,Validation Studies
fas	clathrin	http://www.ncbi.nlm.nih.gov/entrez/query.fcgi?cmd=Retrieve&db=pubmed&dopt=Abstract&list_uids=22908306|6|J Cell Biol|198|4|Aug|2012|Journal Article,Research Support, N.I.H., Extramural,Research Support, Non-U.S. Gov't,Review
git1	pkd3	http://www.ncbi.nlm.nih.gov/entrez/query.fcgi?cmd=Retrieve&db=pubmed&dopt=Abstract&list_uids=22893698|6|J Biol Chem|287|41|Oct|2012|Journal Article,Research Support, Non-U.S. Gov't
atp8a2	pip	http://www.ncbi.nlm.nih.gov/entrez/query.fcgi?cmd=Retrieve&db=pubmed&dopt=Abstract&list_uids=22879066|6|Adv Exp Med Biol|986|||2013|Journal Article,Review
atp8a2	actin	http://www.ncbi.nlm.nih.gov/entrez/query.fcgi?cmd=Retrieve&db=pubmed&dopt=Abstract&list_uids=22879066|6|Adv Exp Med Biol|986|||2013|Journal Article,Review
atp8a2	rac1	http://www.ncbi.nlm.nih.gov/entrez/query.fcgi?cmd=Retrieve&db=pubmed&dopt=Abstract&list_uids=22879066|6|Adv Exp Med Biol|986|||2013|Journal Article,Review
atp8a2	rhoa	http://www.ncbi.nlm.nih.gov/entrez/query.fcgi?cmd=Retrieve&db=pubmed&dopt=Abstract&list_uids=22879066|6|Adv Exp Med Biol|986|||2013|Journal Article,Review
pip	actin	http://www.ncbi.nlm.nih.gov/entrez/query.fcgi?cmd=Retrieve&db=pubmed&dopt=Abstract&list_uids=22879066|6|Adv Exp Med Biol|986|||2013|Journal Article,Review
pip	rac1	http://www.ncbi.nlm.nih.gov/entrez/query.fcgi?cmd=Retrieve&db=pubmed&dopt=Abstract&list_uids=22879066|6|Adv Exp

		Med Biol|986|||2013|Journal Article,Review
pip	rhoa	http://www.ncbi.nlm.nih.gov/entrez/query.fcgi?cmd=Retrieve&db=pubmed&dopt=Abstract&list_uids=22879066|6|Adv Exp Med Biol|986|||2013|Journal Article,Review
igf1	akt1	http://www.ncbi.nlm.nih.gov/entrez/query.fcgi?cmd=Retrieve&db=pubmed&dopt=Abstract&list_uids=22859931|6|PLoS One|7|6||2012|Journal Article,Research Support, N.I.H., Extramural,Research Support, Non-U.S. Gov't
ptk2	igf1r	http://www.ncbi.nlm.nih.gov/entrez/query.fcgi?cmd=Retrieve&db=pubmed&dopt=Abstract&list_uids=22859931|6|PLoS One|7|6||2012|Journal Article,Research Support, N.I.H., Extramural,Research Support, Non-U.S. Gov't
ptk2b	igf1r	http://www.ncbi.nlm.nih.gov/entrez/query.fcgi?cmd=Retrieve&db=pubmed&dopt=Abstract&list_uids=22859931|6|PLoS One|7|6||2012|Journal Article,Research Support, N.I.H., Extramural,Research Support, Non-U.S. Gov't
ptk2b	igf1	http://www.ncbi.nlm.nih.gov/entrez/query.fcgi?cmd=Retrieve&db=pubmed&dopt=Abstract&list_uids=22859931|6|PLoS One|7|6||2012|Journal Article,Research Support, N.I.H., Extramural,Research Support, Non-U.S. Gov't
ptk2b	akt1	http://www.ncbi.nlm.nih.gov/entrez/query.fcgi?cmd=Retrieve&db=pubmed&dopt=Abstract&list_uids=22859931|6|PLoS One|7|6||2012|Journal Article,Research Support, N.I.H., Extramural,Research Support, Non-U.S. Gov't
igf1r	akt1	http://www.ncbi.nlm.nih.gov/entrez/query.fcgi?cmd=Retrieve&db=pubmed&dopt=Abstract&list_uids=22859931|6|PLoS One|7|6||2012|Journal Article,Research Support, N.I.H., Extramural,Research Support, Non-U.S. Gov't
agxt	plekhh2	http://www.ncbi.nlm.nih.gov/entrez/query.fcgi?cmd=Retrieve&db=pubmed&dopt=Abstract&list_uids=22832517|6|Kidney Int|82|10|Nov|2012|Journal Article,Research Support, Non-U.S. Gov't,Video-Audio Media
nell1	st2	http://www.ncbi.nlm.nih.gov/entrez/query.fcgi?cmd=Retrieve&db=pubmed&dopt=Abstract&list_uids=22807400|6|J Cell Biochem|113|12|Dec|2012|Journal Article,Research Support, N.I.H., Extramural,Research Support, Non-U.S. Gov't
nox1	decr1	http://www.ncbi.nlm.nih.gov/entrez/query.fcgi?cmd=Retrieve&db=pubmed&dopt=Abstract&list_uids=22773830|6|J Biol Chem|287|35|Aug|2012|Journal Article
p4hb	nox1	http://www.ncbi.nlm.nih.gov/entrez/query.fcgi?cmd=Retrieve&db=pubmed&dopt=Abstract&list_uids=22773830|6|J Biol Chem|287|35|Aug|2012|Journal Article
p4hb	nox4	http://www.ncbi.nlm.nih.gov/entrez/query.fcgi?cmd=Retrieve&db=pubmed&dopt=Abstract&list_uids=22773830|6|J Biol Chem|287|35|Aug|2012|Journal Article
nox1	nox4	http://www.ncbi.nlm.nih.gov/entrez/query.fcgi?cmd=Retrieve&db=pubmed&dopt=Abstract&list_uids=22773830|6|J Biol Chem|287|35|Aug|2012|Journal Article
p4hb	arhgdia	http://www.ncbi.nlm.nih.gov/entrez/query.fcgi?cmd=Retrieve&db=pubmed&dopt=Abstract&list_uids=22773830|6|J Biol Chem|287|35|Aug|2012|Journal Article
ilk	pik3ca	http://www.ncbi.nlm.nih.gov/entrez/query.fcgi?cmd=Retrieve&db=pubmed&dopt=Abstract&list_uids=22767580|6|Mol Biol Cell|23|17|Sep|2012|Journal Article,Research Support, Non-U.S. Gov't
ilk	mmrn1	http://www.ncbi.nlm.nih.gov/entrez/query.fcgi?cmd=Retrieve&db=pubmed&dopt=Abstract&list_uids=22763012|6|Curr Opin Cell Biol|24|5|Oct|2012|Journal Article,Research Support, N.I.H., Extramural,Review
tns4	nt5c3	http://www.ncbi.nlm.nih.gov/entrez/query.fcgi?cmd=Retrieve&db=pubmed&dopt=Abstract&list_uids=22750970|6|Pancreas|42|1|Jan|2013|Journal Article,Research Support, Non-U.S. Gov't

tns4	ilk	http://www.ncbi.nlm.nih.gov/entrez/query.fcgi?cmd=Retrieve&db=pubmed&dopt=Abstract&list_uids=22750970|6|Pancreas|42|1|Jan|2013|Journal Article,Research Support, Non-U.S. Gov't
ros1	decr1	http://www.ncbi.nlm.nih.gov/entrez/query.fcgi?cmd=Retrieve&db=pubmed&dopt=Abstract&list_uids=22735335|6|Mol Syst Biol|8|||2012|Journal Article,Research Support, N.I.H., Extramural,Research Support, Non-U.S. Gov't
src	ret	http://www.ncbi.nlm.nih.gov/entrez/query.fcgi?cmd=Retrieve&db=pubmed&dopt=Abstract&list_uids=22732841|6|EMBO Rep|13|8|Aug|2012|Journal Article,Research Support, Non-U.S. Gov't
ret	ptk2	http://www.ncbi.nlm.nih.gov/entrez/query.fcgi?cmd=Retrieve&db=pubmed&dopt=Abstract&list_uids=22732841|6|EMBO Rep|13|8|Aug|2012|Journal Article,Research Support, Non-U.S. Gov't
lhx1	lhx3	http://www.ncbi.nlm.nih.gov/entrez/query.fcgi?cmd=Retrieve&db=pubmed&dopt=Abstract&list_uids=22728435|6|FEBS Lett|586|16|Jul|2012|Journal Article,Research Support, N.I.H., Extramural
lhx1	akt1	http://www.ncbi.nlm.nih.gov/entrez/query.fcgi?cmd=Retrieve&db=pubmed&dopt=Abstract&list_uids=22728435|6|FEBS Lett|586|16|Jul|2012|Journal Article,Research Support, N.I.H., Extramural
lhx3	akt1	http://www.ncbi.nlm.nih.gov/entrez/query.fcgi?cmd=Retrieve&db=pubmed&dopt=Abstract&list_uids=22728435|6|FEBS Lett|586|16|Jul|2012|Journal Article,Research Support, N.I.H., Extramural
rhob	akt1	http://www.ncbi.nlm.nih.gov/entrez/query.fcgi?cmd=Retrieve&db=pubmed&dopt=Abstract&list_uids=22724071|6|Open Biol|2|5|May|2012|Journal Article,Research Support, Non-U.S. Gov't
dym	pvrl1	http://www.ncbi.nlm.nih.gov/entrez/query.fcgi?cmd=Retrieve&db=pubmed&dopt=Abstract&list_uids=22721990|6|Cardiovasc Res|95|3|Aug|2012|Journal Article,Research Support, Non-U.S. Gov't
dym	rac1	http://www.ncbi.nlm.nih.gov/entrez/query.fcgi?cmd=Retrieve&db=pubmed&dopt=Abstract&list_uids=22721990|6|Cardiovasc Res|95|3|Aug|2012|Journal Article,Research Support, Non-U.S. Gov't
dym	rhoa	http://www.ncbi.nlm.nih.gov/entrez/query.fcgi?cmd=Retrieve&db=pubmed&dopt=Abstract&list_uids=22721990|6|Cardiovasc Res|95|3|Aug|2012|Journal Article,Research Support, Non-U.S. Gov't
dym	mtg1	http://www.ncbi.nlm.nih.gov/entrez/query.fcgi?cmd=Retrieve&db=pubmed&dopt=Abstract&list_uids=22721990|6|Cardiovasc Res|95|3|Aug|2012|Journal Article,Research Support, Non-U.S. Gov't
rac1	pvrl1	http://www.ncbi.nlm.nih.gov/entrez/query.fcgi?cmd=Retrieve&db=pubmed&dopt=Abstract&list_uids=22721990|6|Cardiovasc Res|95|3|Aug|2012|Journal Article,Research Support, Non-U.S. Gov't
rhoa	mtg1	http://www.ncbi.nlm.nih.gov/entrez/query.fcgi?cmd=Retrieve&db=pubmed&dopt=Abstract&list_uids=22348083|6|PLoS One|7|2||2012|Journal Article,Research Support, Non-U.S. Gov't|http://www.ncbi.nlm.nih.gov/entrez/query.fcgi?cmd=Retrieve&db=pubmed&dopt=Abstract&list_uids=22721990|6|Cardiovasc Res|95|3|Aug|2012|Journal Article,Research Support, Non-U.S. Gov't
rhoa	pvrl1	http://www.ncbi.nlm.nih.gov/entrez/query.fcgi?cmd=Retrieve&db=pubmed&dopt=Abstract&list_uids=22721990|6|Cardiovasc Res|95|3|Aug|2012|Journal Article,Research Support, Non-U.S. Gov't
mtg1	pvrl1	http://www.ncbi.nlm.nih.gov/entrez/query.fcgi?cmd=Retrieve&db=pubmed&dopt=Abstract&list_uids=22721990|6|Cardiovasc Res|95|3|Aug|2012|Journal Article,Research Support, Non-U.S. Gov't
ptk2	dym	http://www.ncbi.nlm.nih.gov/entrez/query.fcgi?cmd=Retrieve&db=pubmed&dopt=Abstract&list_uids=22721990|6|Cardiovasc Res|95|3|Aug|2012|Journal Article,Research Support, Non-U.S. Gov't

ptk2	pvrl1	http://www.ncbi.nlm.nih.gov/entrez/query.fcgi?cmd=Retrieve&db=pubmed&dopt=Abstract&list_uids=22721990|6|Cardiovasc Res|95|3|Aug|2012|Journal Article,Research Support, Non-U.S. Gov't
map3k11	tyr	http://www.ncbi.nlm.nih.gov/entrez/query.fcgi?cmd=Retrieve&db=pubmed&dopt=Abstract&list_uids=22700880|6|Cancer Res|72|16|Aug|2012|Journal Article,Research Support, Non-U.S. Gov't,Research Support, U.S. Gov't, Non-P.H.S.
map3k11	mapk8	http://www.ncbi.nlm.nih.gov/entrez/query.fcgi?cmd=Retrieve&db=pubmed&dopt=Abstract&list_uids=22700880|6|Cancer Res|72|16|Aug|2012|Journal Article,Research Support, Non-U.S. Gov't,Research Support, U.S. Gov't, Non-P.H.S.
ahr	src	http://www.ncbi.nlm.nih.gov/entrez/query.fcgi?cmd=Retrieve&db=pubmed&dopt=Abstract&list_uids=22665056|6|Oncogene|32|14|Apr|2013|Journal Article,Research Support, Non-U.S. Gov't
ahr	ptk2	http://www.ncbi.nlm.nih.gov/entrez/query.fcgi?cmd=Retrieve&db=pubmed&dopt=Abstract&list_uids=22665056|6|Oncogene|32|14|Apr|2013|Journal Article,Research Support, Non-U.S. Gov't
ahr	nedd9	http://www.ncbi.nlm.nih.gov/entrez/query.fcgi?cmd=Retrieve&db=pubmed&dopt=Abstract&list_uids=22665056|6|Oncogene|32|14|Apr|2013|Journal Article,Research Support, Non-U.S. Gov't
ptk2	nedd9	http://www.ncbi.nlm.nih.gov/entrez/query.fcgi?cmd=Retrieve&db=pubmed&dopt=Abstract&list_uids=22665056|6|Oncogene|32|14|Apr|2013|Journal Article,Research Support, Non-U.S. Gov't
iqgap1	rac1	http://www.ncbi.nlm.nih.gov/entrez/query.fcgi?cmd=Retrieve&db=pubmed&dopt=Abstract&list_uids=22634185|6|Cell Signal|24|9|Sep|2012|Journal Article,Research Support, Non-U.S. Gov't
iqgap1	rhoa	http://www.ncbi.nlm.nih.gov/entrez/query.fcgi?cmd=Retrieve&db=pubmed&dopt=Abstract&list_uids=22634185|6|Cell Signal|24|9|Sep|2012|Journal Article,Research Support, Non-U.S. Gov't
itk	mmrn1	http://www.ncbi.nlm.nih.gov/entrez/query.fcgi?cmd=Retrieve&db=pubmed&dopt=Abstract&list_uids=22605996|6|Int J Mol Sci|13|4||2012|Journal Article,Research Support, Non-U.S. Gov't,Review
dspp	actin	http://www.ncbi.nlm.nih.gov/entrez/query.fcgi?cmd=Retrieve&db=pubmed&dopt=Abstract&list_uids=22588498|6|Cell Adh Migr|6|4||2012|Journal Article,Research Support, N.I.H., Extramural,Research Support, Non-U.S. Gov't
egf	slc12a2	http://www.ncbi.nlm.nih.gov/entrez/query.fcgi?cmd=Retrieve&db=pubmed&dopt=Abstract&list_uids=22570591|6|PLoS Biol|10|5||2012|Journal Article,Research Support, N.I.H., Extramural,Research Support, Non-U.S. Gov't
tax1bp3	bcatenin	http://www.ncbi.nlm.nih.gov/entrez/query.fcgi?cmd=Retrieve&db=pubmed&dopt=Abstract&list_uids=22564736|6|Biochem Biophys Res Commun|422|1|May|2012|Journal Article,Research Support, N.I.H., Extramural
dync1i2	pkc	http://www.ncbi.nlm.nih.gov/entrez/query.fcgi?cmd=Retrieve&db=pubmed&dopt=Abstract&list_uids=22553211|6|J Cell Sci|125|Pt 16|Aug|2012|Journal Article,Research Support, Non-U.S. Gov't
actin	ptgs2	http://www.ncbi.nlm.nih.gov/entrez/query.fcgi?cmd=Retrieve&db=pubmed&dopt=Abstract&list_uids=22542712|6|Exp Mol Pathol|93|1|Aug|2012|Journal Article,Research Support, N.I.H., Extramural
ptk2	ptgs2	http://www.ncbi.nlm.nih.gov/entrez/query.fcgi?cmd=Retrieve&db=pubmed&dopt=Abstract&list_uids=22542712|6|Exp Mol Pathol|93|1|Aug|2012|Journal Article,Research Support, N.I.H., Extramural
ptk2	mylk	http://www.ncbi.nlm.nih.gov/entrez/query.fcgi?cmd=Retrieve&db=pubmed&dopt=Abstract&list_uids=22542712|6|Exp Mol Pathol|93|1|Aug|2012|Journal Article,Research Support, N.I.H., Extramural
ptk2	edn1	http://www.ncbi.nlm.nih.gov/entrez/query.fcgi?cmd=Retrieve&db=pubmed&dopt=Abstract&list_uids=22542712|6|Exp Mol Pathol|93|1|Aug|2012|Journal Article,Research Support, N.I.H., Extramural

mylk	edn1	http://www.ncbi.nlm.nih.gov/entrez/query.fcgi?cmd=Retrieve&db=pubmed&dopt=Abstract&list_uids=22542712|6|Exp Mol Pathol|93|1|Aug|2012|Journal Article,Research Support, N.I.H., Extramural
lasp1	actin	http://www.ncbi.nlm.nih.gov/entrez/query.fcgi?cmd=Retrieve&db=pubmed&dopt=Abstract&list_uids=22514729|6|PLoS One|7|4||2012|Journal Article,Research Support, Non-U.S. Gov't
lgals3	rhoa	http://www.ncbi.nlm.nih.gov/entrez/query.fcgi?cmd=Retrieve&db=pubmed&dopt=Abstract&list_uids=22513979|6|J Pathol|228|1|Sep|2012|Journal Article,Research Support, Non-U.S. Gov't
lgals3	atm	http://www.ncbi.nlm.nih.gov/entrez/query.fcgi?cmd=Retrieve&db=pubmed&dopt=Abstract&list_uids=22513979|6|J Pathol|228|1|Sep|2012|Journal Article,Research Support, Non-U.S. Gov't
lgals3	traf3ip2	http://www.ncbi.nlm.nih.gov/entrez/query.fcgi?cmd=Retrieve&db=pubmed&dopt=Abstract&list_uids=22513979|6|J Pathol|228|1|Sep|2012|Journal Article,Research Support, Non-U.S. Gov't
cav1	rhoa	http://www.ncbi.nlm.nih.gov/entrez/query.fcgi?cmd=Retrieve&db=pubmed&dopt=Abstract&list_uids=22513979|6|J Pathol|228|1|Sep|2012|Journal Article,Research Support, Non-U.S. Gov't
cav1	atm	http://www.ncbi.nlm.nih.gov/entrez/query.fcgi?cmd=Retrieve&db=pubmed&dopt=Abstract&list_uids=22513979|6|J Pathol|228|1|Sep|2012|Journal Article,Research Support, Non-U.S. Gov't
cav1	traf3ip2	http://www.ncbi.nlm.nih.gov/entrez/query.fcgi?cmd=Retrieve&db=pubmed&dopt=Abstract&list_uids=22513979|6|J Pathol|228|1|Sep|2012|Journal Article,Research Support, Non-U.S. Gov't
rhoa	atm	http://www.ncbi.nlm.nih.gov/entrez/query.fcgi?cmd=Retrieve&db=pubmed&dopt=Abstract&list_uids=22513979|6|J Pathol|228|1|Sep|2012|Journal Article,Research Support, Non-U.S. Gov't
rhoa	traf3ip2	http://www.ncbi.nlm.nih.gov/entrez/query.fcgi?cmd=Retrieve&db=pubmed&dopt=Abstract&list_uids=22513979|6|J Pathol|228|1|Sep|2012|Journal Article,Research Support, Non-U.S. Gov't
ptk2	atm	http://www.ncbi.nlm.nih.gov/entrez/query.fcgi?cmd=Retrieve&db=pubmed&dopt=Abstract&list_uids=22513979|6|J Pathol|228|1|Sep|2012|Journal Article,Research Support, Non-U.S. Gov't
ptk2	traf3ip2	http://www.ncbi.nlm.nih.gov/entrez/query.fcgi?cmd=Retrieve&db=pubmed&dopt=Abstract&list_uids=22513979|6|J Pathol|228|1|Sep|2012|Journal Article,Research Support, Non-U.S. Gov't
atm	traf3ip2	http://www.ncbi.nlm.nih.gov/entrez/query.fcgi?cmd=Retrieve&db=pubmed&dopt=Abstract&list_uids=22513979|6|J Pathol|228|1|Sep|2012|Journal Article,Research Support, Non-U.S. Gov't
myh11	myh14	http://www.ncbi.nlm.nih.gov/entrez/query.fcgi?cmd=Retrieve&db=pubmed&dopt=Abstract&list_uids=22511748|6|Circ Res|110|11|May|2012|Journal Article,Research Support, N.I.H., Extramural,Research Support, Non-U.S. Gov't
myh11	dym	http://www.ncbi.nlm.nih.gov/entrez/query.fcgi?cmd=Retrieve&db=pubmed&dopt=Abstract&list_uids=22511748|6|Circ Res|110|11|May|2012|Journal Article,Research Support, N.I.H., Extramural,Research Support, Non-U.S. Gov't
myh14	dym	http://www.ncbi.nlm.nih.gov/entrez/query.fcgi?cmd=Retrieve&db=pubmed&dopt=Abstract&list_uids=22511748|6|Circ Res|110|11|May|2012|Journal Article,Research Support, N.I.H., Extramural,Research Support, Non-U.S. Gov't
tbx1	ddr1	http://www.ncbi.nlm.nih.gov/entrez/query.fcgi?cmd=Retrieve&db=pubmed&dopt=Abstract&list_uids=22500976|6|Radiat Oncol|7|||2012|Journal Article,Research Support, Non-U.S. Gov't
air	mmp3	http://www.ncbi.nlm.nih.gov/entrez/query.fcgi?cmd=Retrieve&db=pubmed&dopt=Abstract&list_uids=22500976|6|Radiat Oncol|7|||2012|Journal Article,Research Support, Non-U.S. Gov't

air	mmp1	http://www.ncbi.nlm.nih.gov/entrez/query.fcgi?cmd=Retrieve&db=pubmed&dopt=Abstract&list_uids=22500976|6|Radiat Oncol|7|||2012|Journal Article,Research Support, Non-U.S. Gov't
mmp3	mmp1	http://www.ncbi.nlm.nih.gov/entrez/query.fcgi?cmd=Retrieve&db=pubmed&dopt=Abstract&list_uids=22500976|6|Radiat Oncol|7|||2012|Journal Article,Research Support, Non-U.S. Gov't
rhoa	rieg2	http://www.ncbi.nlm.nih.gov/entrez/query.fcgi?cmd=Retrieve&db=pubmed&dopt=Abstract&list_uids=22500016|6|J Biol Chem|287|23|Jun|2012|Journal Article
src	rapgef1	http://www.ncbi.nlm.nih.gov/entrez/query.fcgi?cmd=Retrieve&db=pubmed&dopt=Abstract&list_uids=22499769|6|Mol Cell Proteomics|11|8|Aug|2012|Journal Article,Research Support, N.I.H., Extramural,Research Support, Non-U.S. Gov't
rap1a	rapgef1	http://www.ncbi.nlm.nih.gov/entrez/query.fcgi?cmd=Retrieve&db=pubmed&dopt=Abstract&list_uids=22499769|6|Mol Cell Proteomics|11|8|Aug|2012|Journal Article,Research Support, N.I.H., Extramural,Research Support, Non-U.S. Gov't
cox8a	src	http://www.ncbi.nlm.nih.gov/entrez/query.fcgi?cmd=Retrieve&db=pubmed&dopt=Abstract&list_uids=22488171|6|J Cancer Res Clin Oncol|138|8|Aug|2012|Journal Article
cox8a	ptk2	http://www.ncbi.nlm.nih.gov/entrez/query.fcgi?cmd=Retrieve&db=pubmed&dopt=Abstract&list_uids=22488171|6|J Cancer Res Clin Oncol|138|8|Aug|2012|Journal Article
cox8a	serpinb 3	http://www.ncbi.nlm.nih.gov/entrez/query.fcgi?cmd=Retrieve&db=pubmed&dopt=Abstract&list_uids=22488171|6|J Cancer Res Clin Oncol|138|8|Aug|2012|Journal Article
src	serpinb 3	http://www.ncbi.nlm.nih.gov/entrez/query.fcgi?cmd=Retrieve&db=pubmed&dopt=Abstract&list_uids=22488171|6|J Cancer Res Clin Oncol|138|8|Aug|2012|Journal Article
ptk2	serpinb 3	http://www.ncbi.nlm.nih.gov/entrez/query.fcgi?cmd=Retrieve&db=pubmed&dopt=Abstract&list_uids=22488171|6|J Cancer Res Clin Oncol|138|8|Aug|2012|Journal Article
bcar1	enah	http://www.ncbi.nlm.nih.gov/entrez/query.fcgi?cmd=Retrieve&db=pubmed&dopt=Abstract&list_uids=22456508|6|Mol Biol Cell|23|10|May|2012|Journal Article,Research Support, N.I.H., Extramural,Research Support, Non-U.S. Gov't
bcar1	vasp	http://www.ncbi.nlm.nih.gov/entrez/query.fcgi?cmd=Retrieve&db=pubmed&dopt=Abstract&list_uids=22456508|6|Mol Biol Cell|23|10|May|2012|Journal Article,Research Support, N.I.H., Extramural,Research Support, Non-U.S. Gov't
sh2b3	akt1	http://www.ncbi.nlm.nih.gov/entrez/query.fcgi?cmd=Retrieve&db=pubmed&dopt=Abstract&list_uids=22441983|6|FASEB J|26|6|Jun|2012|Journal Article,Research Support, N.I.H., Extramural,Research Support, Non-U.S. Gov't
sorbs3	sorbs2	http://www.ncbi.nlm.nih.gov/entrez/query.fcgi?cmd=Retrieve&db=pubmed&dopt=Abstract&list_uids=22431180|6|Med Mol Morphol|45|1|Dec|2012|Journal Article,Research Support, Non-U.S. Gov't
ptk2	s100p	http://www.ncbi.nlm.nih.gov/entrez/query.fcgi?cmd=Retrieve&db=pubmed&dopt=Abstract&list_uids=22399300|6|J Biol Chem|287|19|May|2012|Journal Article,Research Support, Non-U.S. Gov't
fas	s100p	http://www.ncbi.nlm.nih.gov/entrez/query.fcgi?cmd=Retrieve&db=pubmed&dopt=Abstract&list_uids=22399300|6|J Biol Chem|287|19|May|2012|Journal Article,Research Support, Non-U.S. Gov't
junb	tpm1	http://www.ncbi.nlm.nih.gov/entrez/query.fcgi?cmd=Retrieve&db=pubmed&dopt=Abstract&list_uids=22391036|6|J Cell Biol|196|5|Mar|2012|Journal Article,Research Support, N.I.H., Extramural,Research Support, Non-U.S. Gov't,Research Support, U.S. Gov't, P.H.S.
junb	actin	http://www.ncbi.nlm.nih.gov/entrez/query.fcgi?cmd=Retrieve&db=pubmed&dopt=Abstract&list_uids=22391036|6|J Cell

		Biol|196|5|Mar|2012|Journal Article,Research Support, N.I.H., Extramural,Research Support, Non-U.S. Gov't,Research Support, U.S. Gov't, P.H.S.
tpm1	actin	http://www.ncbi.nlm.nih.gov/entrez/query.fcgi?cmd=Retrieve&db=pubmed&dopt=Abstract&list_uids=22391036|6|J Cell Biol|196|5|Mar|2012|Journal Article,Research Support, N.I.H., Extramural,Research Support, Non-U.S. Gov't,Research Support, U.S. Gov't, P.H.S.
tnfsf13	ptk2	http://www.ncbi.nlm.nih.gov/entrez/query.fcgi?cmd=Retrieve&db=pubmed&dopt=Abstract&list_uids=22364697|6|Biomaterials|33|15|May|2012|Journal Article,Research Support, Non-U.S. Gov't
tat	angptl6	http://www.ncbi.nlm.nih.gov/entrez/query.fcgi?cmd=Retrieve&db=pubmed&dopt=Abstract&list_uids=22362758|6|Arterioscler Thromb Vasc Biol|32|5|May|2012|Comparative Study,Journal Article,Research Support, Non-U.S. Gov't
tat	actin	http://www.ncbi.nlm.nih.gov/entrez/query.fcgi?cmd=Retrieve&db=pubmed&dopt=Abstract&list_uids=22362758|6|Arterioscler Thromb Vasc Biol|32|5|May|2012|Comparative Study,Journal Article,Research Support, Non-U.S. Gov't
tat	kdr	http://www.ncbi.nlm.nih.gov/entrez/query.fcgi?cmd=Retrieve&db=pubmed&dopt=Abstract&list_uids=22362758|6|Arterioscler Thromb Vasc Biol|32|5|May|2012|Comparative Study,Journal Article,Research Support, Non-U.S. Gov't
angptl6	actin	http://www.ncbi.nlm.nih.gov/entrez/query.fcgi?cmd=Retrieve&db=pubmed&dopt=Abstract&list_uids=22362758|6|Arterioscler Thromb Vasc Biol|32|5|May|2012|Comparative Study,Journal Article,Research Support, Non-U.S. Gov't
angptl6	kdr	http://www.ncbi.nlm.nih.gov/entrez/query.fcgi?cmd=Retrieve&db=pubmed&dopt=Abstract&list_uids=22362758|6|Arterioscler Thromb Vasc Biol|32|5|May|2012|Comparative Study,Journal Article,Research Support, Non-U.S. Gov't
actin	kdr	http://www.ncbi.nlm.nih.gov/entrez/query.fcgi?cmd=Retrieve&db=pubmed&dopt=Abstract&list_uids=22362758|6|Arterioscler Thromb Vasc Biol|32|5|May|2012|Comparative Study,Journal Article,Research Support, Non-U.S. Gov't
kdr	ephb2	http://www.ncbi.nlm.nih.gov/entrez/query.fcgi?cmd=Retrieve&db=pubmed&dopt=Abstract&list_uids=22362758|6|Arterioscler Thromb Vasc Biol|32|5|May|2012|Comparative Study,Journal Article,Research Support, Non-U.S. Gov't
ephb2	tat	http://www.ncbi.nlm.nih.gov/entrez/query.fcgi?cmd=Retrieve&db=pubmed&dopt=Abstract&list_uids=22362758|6|Arterioscler Thromb Vasc Biol|32|5|May|2012|Comparative Study,Journal Article,Research Support, Non-U.S. Gov't
src	ilk	http://www.ncbi.nlm.nih.gov/entrez/query.fcgi?cmd=Retrieve&db=pubmed&dopt=Abstract&list_uids=22357970|6|J Cell Sci|125|Pt 2|Jan|2012|Journal Article,Research Support, Non-U.S. Gov't
egf	ilk	http://www.ncbi.nlm.nih.gov/entrez/query.fcgi?cmd=Retrieve&db=pubmed&dopt=Abstract&list_uids=22357970|6|J Cell Sci|125|Pt 2|Jan|2012|Journal Article,Research Support, Non-U.S. Gov't
egf	cyld	http://www.ncbi.nlm.nih.gov/entrez/query.fcgi?cmd=Retrieve&db=pubmed&dopt=Abstract&list_uids=22357970|6|J Cell Sci|125|Pt 2|Jan|2012|Journal Article,Research Support, Non-U.S. Gov't
cyld	src	http://www.ncbi.nlm.nih.gov/entrez/query.fcgi?cmd=Retrieve&db=pubmed&dopt=Abstract&list_uids=22357970|6|J Cell Sci|125|Pt 2|Jan|2012|Journal Article,Research Support, Non-U.S. Gov't
cyld	actin	http://www.ncbi.nlm.nih.gov/entrez/query.fcgi?cmd=Retrieve&db=pubmed&dopt=Abstract&list_uids=22357970|6|J Cell Sci|125|Pt 2|Jan|2012|Journal Article,Research Support, Non-U.S. Gov't
angpt1	tnfsf13	http://www.ncbi.nlm.nih.gov/entrez/query.fcgi?cmd=Retrieve&db=pubmed&dopt=Abstract&list_uids=22357955|6|J Cell Sci|125|Pt 9|May|2012|Journal Article,Research Support, Non-U.S. Gov't
angpt1	tek	http://www.ncbi.nlm.nih.gov/entrez/query.fcgi?cmd=Retrieve&db=pubmed&dopt=Abstract&list_uids=22357955|6|J Cell

		Sci|125|Pt 9|May|2012|Journal Article,Research Support, Non-U.S. Gov't
angpt1	angpt2	http://www.ncbi.nlm.nih.gov/entrez/query.fcgi?cmd=Retrieve&db=pubmed&dopt=Abstract&list_uids=22357955|6|J Cell Sci|125|Pt 9|May|2012|Journal Article,Research Support, Non-U.S. Gov't
tnfsf13	tek	http://www.ncbi.nlm.nih.gov/entrez/query.fcgi?cmd=Retrieve&db=pubmed&dopt=Abstract&list_uids=22357955|6|J Cell Sci|125|Pt 9|May|2012|Journal Article,Research Support, Non-U.S. Gov't
tnfsf13	angpt2	http://www.ncbi.nlm.nih.gov/entrez/query.fcgi?cmd=Retrieve&db=pubmed&dopt=Abstract&list_uids=22357955|6|J Cell Sci|125|Pt 9|May|2012|Journal Article,Research Support, Non-U.S. Gov't
tek	angpt2	http://www.ncbi.nlm.nih.gov/entrez/query.fcgi?cmd=Retrieve&db=pubmed&dopt=Abstract&list_uids=22357955|6|J Cell Sci|125|Pt 9|May|2012|Journal Article,Research Support, Non-U.S. Gov't
hnrnpc	rhoa	http://www.ncbi.nlm.nih.gov/entrez/query.fcgi?cmd=Retrieve&db=pubmed&dopt=Abstract&list_uids=22357624|6|Mol Biol Cell|23|8|Apr|2012|Journal Article,Research Support, N.I.H., Extramural,Research Support, Non-U.S. Gov't
rock1	rock2	http://www.ncbi.nlm.nih.gov/entrez/query.fcgi?cmd=Retrieve&db=pubmed&dopt=Abstract&list_uids=22348083|6|PLoS One|7|2||2012|Journal Article,Research Support, Non-U.S. Gov't
rock1	mtg1	http://www.ncbi.nlm.nih.gov/entrez/query.fcgi?cmd=Retrieve&db=pubmed&dopt=Abstract&list_uids=22348083|6|PLoS One|7|2||2012|Journal Article,Research Support, Non-U.S. Gov't
rock1	rhoa	http://www.ncbi.nlm.nih.gov/entrez/query.fcgi?cmd=Retrieve&db=pubmed&dopt=Abstract&list_uids=22348083|6|PLoS One|7|2||2012|Journal Article,Research Support, Non-U.S. Gov't
rock2	mtg1	http://www.ncbi.nlm.nih.gov/entrez/query.fcgi?cmd=Retrieve&db=pubmed&dopt=Abstract&list_uids=22348083|6|PLoS One|7|2||2012|Journal Article,Research Support, Non-U.S. Gov't
rock2	rhoa	http://www.ncbi.nlm.nih.gov/entrez/query.fcgi?cmd=Retrieve&db=pubmed&dopt=Abstract&list_uids=22348083|6|PLoS One|7|2||2012|Journal Article,Research Support, Non-U.S. Gov't
dlc1	tns4	http://www.ncbi.nlm.nih.gov/entrez/query.fcgi?cmd=Retrieve&db=pubmed&dopt=Abstract&list_uids=22307599|6|Proc Natl Acad Sci U S A|109|5|Jan|2012|Journal Article,Research Support, Non-U.S. Gov't
tns4	egf	http://www.ncbi.nlm.nih.gov/entrez/query.fcgi?cmd=Retrieve&db=pubmed&dopt=Abstract&list_uids=22307599|6|Proc Natl Acad Sci U S A|109|5|Jan|2012|Journal Article,Research Support, Non-U.S. Gov't
actin	tns4	http://www.ncbi.nlm.nih.gov/entrez/query.fcgi?cmd=Retrieve&db=pubmed&dopt=Abstract&list_uids=22307599|6|Proc Natl Acad Sci U S A|109|5|Jan|2012|Journal Article,Research Support, Non-U.S. Gov't
tns4	rhoa	http://www.ncbi.nlm.nih.gov/entrez/query.fcgi?cmd=Retrieve&db=pubmed&dopt=Abstract&list_uids=22307599|6|Proc Natl Acad Sci U S A|109|5|Jan|2012|Journal Article,Research Support, Non-U.S. Gov't
nrp2	ptk2	http://www.ncbi.nlm.nih.gov/entrez/query.fcgi?cmd=Retrieve&db=pubmed&dopt=Abstract&list_uids=22302985|6|J Cell Sci|125|Pt 2|Jan|2012|Journal Article,Research Support, N.I.H., Extramural
nrp2	src	http://www.ncbi.nlm.nih.gov/entrez/query.fcgi?cmd=Retrieve&db=pubmed&dopt=Abstract&list_uids=22302985|6|J Cell Sci|125|Pt 2|Jan|2012|Journal Article,Research Support, N.I.H., Extramural
nrp2	pkc	http://www.ncbi.nlm.nih.gov/entrez/query.fcgi?cmd=Retrieve&db=pubmed&dopt=Abstract&list_uids=22302985|6|J Cell Sci|125|Pt 2|Jan|2012|Journal Article,Research Support, N.I.H., Extramural

tgm2	mmrn1	http://www.ncbi.nlm.nih.gov/entrez/query.fcgi?cmd=Retrieve&db=pubmed&dopt=Abstract&list_uids=22298777|6|J Biol Chem|287|16|Apr|2012|Journal Article,Research Support, Non-U.S. Gov't
tgm2	mapk3	http://www.ncbi.nlm.nih.gov/entrez/query.fcgi?cmd=Retrieve&db=pubmed&dopt=Abstract&list_uids=22298777|6|J Biol Chem|287|16|Apr|2012|Journal Article,Research Support, Non-U.S. Gov't
tgm2	actin	http://www.ncbi.nlm.nih.gov/entrez/query.fcgi?cmd=Retrieve&db=pubmed&dopt=Abstract&list_uids=22298777|6|J Biol Chem|287|16|Apr|2012|Journal Article,Research Support, Non-U.S. Gov't
mapk3	actin	http://www.ncbi.nlm.nih.gov/entrez/query.fcgi?cmd=Retrieve&db=pubmed&dopt=Abstract&list_uids=22298777|6|J Biol Chem|287|16|Apr|2012|Journal Article,Research Support, Non-U.S. Gov't
mmp14	fas	http://www.ncbi.nlm.nih.gov/entrez/query.fcgi?cmd=Retrieve&db=pubmed&dopt=Abstract&list_uids=22291036|6|J Cell Biol|196|3|Feb|2012|Journal Article,Research Support, N.I.H., Extramural
mmp14	src	http://www.ncbi.nlm.nih.gov/entrez/query.fcgi?cmd=Retrieve&db=pubmed&dopt=Abstract&list_uids=22291036|6|J Cell Biol|196|3|Feb|2012|Journal Article,Research Support, N.I.H., Extramural
mmp14	tyr	http://www.ncbi.nlm.nih.gov/entrez/query.fcgi?cmd=Retrieve&db=pubmed&dopt=Abstract&list_uids=22291036|6|J Cell Biol|196|3|Feb|2012|Journal Article,Research Support, N.I.H., Extramural
mmp14	mt1a	http://www.ncbi.nlm.nih.gov/entrez/query.fcgi?cmd=Retrieve&db=pubmed&dopt=Abstract&list_uids=22291036|6|J Cell Biol|196|3|Feb|2012|Journal Article,Research Support, N.I.H., Extramural
fas	tyr	http://www.ncbi.nlm.nih.gov/entrez/query.fcgi?cmd=Retrieve&db=pubmed&dopt=Abstract&list_uids=22291036|6|J Cell Biol|196|3|Feb|2012|Journal Article,Research Support, N.I.H., Extramural
fas	mt1a	http://www.ncbi.nlm.nih.gov/entrez/query.fcgi?cmd=Retrieve&db=pubmed&dopt=Abstract&list_uids=22291036|6|J Cell Biol|196|3|Feb|2012|Journal Article,Research Support, N.I.H., Extramural
src	tyr	http://www.ncbi.nlm.nih.gov/entrez/query.fcgi?cmd=Retrieve&db=pubmed&dopt=Abstract&list_uids=22291036|6|J Cell Biol|196|3|Feb|2012|Journal Article,Research Support, N.I.H., Extramural
src	mt1a	http://www.ncbi.nlm.nih.gov/entrez/query.fcgi?cmd=Retrieve&db=pubmed&dopt=Abstract&list_uids=22291036|6|J Cell Biol|196|3|Feb|2012|Journal Article,Research Support, N.I.H., Extramural
tyr	mt1a	http://www.ncbi.nlm.nih.gov/entrez/query.fcgi?cmd=Retrieve&db=pubmed&dopt=Abstract&list_uids=22291036|6|J Cell Biol|196|3|Feb|2012|Journal Article,Research Support, N.I.H., Extramural
src	mapk3	http://www.ncbi.nlm.nih.gov/entrez/query.fcgi?cmd=Retrieve&db=pubmed&dopt=Abstract&list_uids=22287273|6|J Cell Physiol|227|11|Nov|2012|Journal Article,Research Support, N.I.H., Extramural
cse1l	mapk3	http://www.ncbi.nlm.nih.gov/entrez/query.fcgi?cmd=Retrieve&db=pubmed&dopt=Abstract&list_uids=22287273|6|J Cell Physiol|227|11|Nov|2012|Journal Article,Research Support, N.I.H., Extramural
igf2bp1	zbp1	http://www.ncbi.nlm.nih.gov/entrez/query.fcgi?cmd=Retrieve&db=pubmed&dopt=Abstract&list_uids=22266909|6|J Cell Sci|125|Pt 1|Jan|2012|Journal Article,Research Support, N.I.H., Extramural,Research Support, Non-U.S. Gov't
zbp1	actr2	http://www.ncbi.nlm.nih.gov/entrez/query.fcgi?cmd=Retrieve&db=pubmed&dopt=Abstract&list_uids=22266909|6|J Cell Sci|125|Pt 1|Jan|2012|Journal Article,Research Support, N.I.H., Extramural,Research Support, Non-U.S. Gov't
src	myh14	http://www.ncbi.nlm.nih.gov/entrez/query.fcgi?cmd=Retrieve&db=pubmed&dopt=Abstract&list_uids=22266905|6|J Cell Sci|125|Pt 1|Jan|2012|Journal Article,Research Support, Non-U.S. Gov't

src	mylk	http://www.ncbi.nlm.nih.gov/entrez/query.fcgi?cmd=Retrieve&db=pubmed&dopt=Abstract&list_uids=22266905|6|J Cell Sci|125|Pt 1|Jan|2012|Journal Article,Research Support, Non-U.S. Gov't
myh14	mylk	http://www.ncbi.nlm.nih.gov/entrez/query.fcgi?cmd=Retrieve&db=pubmed&dopt=Abstract&list_uids=22266905|6|J Cell Sci|125|Pt 1|Jan|2012|Journal Article,Research Support, Non-U.S. Gov't
pip	exoc7	http://www.ncbi.nlm.nih.gov/entrez/query.fcgi?cmd=Retrieve&db=pubmed&dopt=Abstract&list_uids=22264730|6|Dev Cell|22|1|Jan|2012|Journal Article,Research Support, N.I.H., Extramural,Research Support, Non-U.S. Gov't
mbp	actin	http://www.ncbi.nlm.nih.gov/entrez/query.fcgi?cmd=Retrieve&db=pubmed&dopt=Abstract&list_uids=22249765|6|Neurochem Res|37|6|Jun|2012|Journal Article,Research Support, Non-U.S. Gov't
mbp	tjp1	http://www.ncbi.nlm.nih.gov/entrez/query.fcgi?cmd=Retrieve&db=pubmed&dopt=Abstract&list_uids=22249765|6|Neurochem Res|37|6|Jun|2012|Journal Article,Research Support, Non-U.S. Gov't
actin	tjp1	http://www.ncbi.nlm.nih.gov/entrez/query.fcgi?cmd=Retrieve&db=pubmed&dopt=Abstract&list_uids=22249765|6|Neurochem Res|37|6|Jun|2012|Journal Article,Research Support, Non-U.S. Gov't
actin	mlc1	http://www.ncbi.nlm.nih.gov/entrez/query.fcgi?cmd=Retrieve&db=pubmed&dopt=Abstract&list_uids=22237407|6|Am J Physiol Cell Physiol|302|7|Apr|2012|Journal Article,Research Support, N.I.H., Extramural,Research Support, N.I.H., Intramural
ranbp9	app	http://www.ncbi.nlm.nih.gov/entrez/query.fcgi?cmd=Retrieve&db=pubmed&dopt=Abstract&list_uids=22223749|6|FASEB J|26|4|Apr|2012|Journal Article,Research Support, N.I.H., Extramural,Research Support, Non-U.S. Gov't
ranbp9	bace1	http://www.ncbi.nlm.nih.gov/entrez/query.fcgi?cmd=Retrieve&db=pubmed&dopt=Abstract&list_uids=22223749|6|FASEB J|26|4|Apr|2012|Journal Article,Research Support, N.I.H., Extramural,Research Support, Non-U.S. Gov't
ranbp9	rpsa	http://www.ncbi.nlm.nih.gov/entrez/query.fcgi?cmd=Retrieve&db=pubmed&dopt=Abstract&list_uids=22223749|6|FASEB J|26|4|Apr|2012|Journal Article,Research Support, N.I.H., Extramural,Research Support, Non-U.S. Gov't
app	bace1	http://www.ncbi.nlm.nih.gov/entrez/query.fcgi?cmd=Retrieve&db=pubmed&dopt=Abstract&list_uids=22223749|6|FASEB J|26|4|Apr|2012|Journal Article,Research Support, N.I.H., Extramural,Research Support, Non-U.S. Gov't
app	rpsa	http://www.ncbi.nlm.nih.gov/entrez/query.fcgi?cmd=Retrieve&db=pubmed&dopt=Abstract&list_uids=22223749|6|FASEB J|26|4|Apr|2012|Journal Article,Research Support, N.I.H., Extramural,Research Support, Non-U.S. Gov't
bace1	rpsa	http://www.ncbi.nlm.nih.gov/entrez/query.fcgi?cmd=Retrieve&db=pubmed&dopt=Abstract&list_uids=22223749|6|FASEB J|26|4|Apr|2012|Journal Article,Research Support, N.I.H., Extramural,Research Support, Non-U.S. Gov't
wnt5a	tnfsf13	http://www.ncbi.nlm.nih.gov/entrez/query.fcgi?cmd=Retrieve&db=pubmed&dopt=Abstract&list_uids=22218904|6|Methods Mol Biol|839|||2012|Journal Article,Research Support, Non-U.S. Gov't

List of invadopodia-associated proteins identified using GLAD4U

**Rank**	**Gene ID**	**Gene symbol**	**Specie**	**Score**
1	2017	cttn	Homo sapiens	40.23392
2	9644	sh3pxd2a	Homo sapiens	36.58909
3	285590	sh3pxd2b	Homo sapiens	25.29619
4	50807	asap1	Homo sapiens	17.6154
5	6714	src	Homo sapiens	14.65431
6	4323	mmp14	Homo sapiens	13.44519
7	7454	was	Homo sapiens	10.61229
8	2191	fap	Homo sapiens	8.895094
9	8871	synj2	Homo sapiens	8.603794
10	7456	wipf1	Homo sapiens	7.730623
11	4318	mmp9	Homo sapiens	7.723941
12	6548	slc9a1	Homo sapiens	7.306912
13	23022	palld	Homo sapiens	7.204015
14	5829	pxn	Homo sapiens	6.450688
15	10749	kif1c	Homo sapiens	6.352221
16	8976	wasl	Homo sapiens	6.349799
17	10811	noxa1	Homo sapiens	6.259372
18	30011	sh3kbp1	Homo sapiens	6.18967
19	998	cdc42	Homo sapiens	5.156586
20	134265	afap1l1	Homo sapiens	4.921854
21	10642	igf2bp1	Homo sapiens	4.547113
22	5747	ptk2	Homo sapiens	4.485162
23	1759	dnm1	Homo sapiens	4.181252
24	6624	fscn1	Homo sapiens	4.155068
25	1839	hbegf	Homo sapiens	4.039629
26	55763	exoc1	Homo sapiens	4.032285
27	8038	adam12	Homo sapiens	4.008513
28	90102	phldb2	Homo sapiens	3.939549

29	8853	asap2	Homo sapiens	3.910733
30	1445	csk	Homo sapiens	3.697452
31	23048	fnbp1	Homo sapiens	3.665857
32	2245	fgd1	Homo sapiens	3.644816
33	9051	pstpip1	Homo sapiens	3.62428
34	10810	wasf3	Homo sapiens	3.604225
35	9322	trip10	Homo sapiens	3.546736
36	867	cbl	Homo sapiens	3.537081
37	6840	svil	Homo sapiens	3.510451
38	60412	exoc4	Homo sapiens	3.410022
39	64866	cdcp1	Homo sapiens	3.363921
40	25	abl1	Homo sapiens	3.290002
41	3936	lcp1	Homo sapiens	3.23894
42	387	rhoa	Homo sapiens	3.201915
43	800	cald1	Homo sapiens	3.176709
44	6845	vamp7	Homo sapiens	3.176709
45	1803	dpp4	Homo sapiens	3.110556
46	10163	wasf2	Homo sapiens	3.013505
47	27	abl2	Homo sapiens	3.013505
48	8773	snap23	Homo sapiens	2.993946
49	5879	rac1	Homo sapiens	2.915688
50	7430	ezr	Homo sapiens	2.892122
51	85360	syde1	Homo sapiens	2.809182
52	3690	itgb3	Homo sapiens	2.756912
53	868	cblb	Homo sapiens	2.752045
54	3688	itgb1	Homo sapiens	2.673773
55	3685	itgav	Homo sapiens	2.649293
56	10643	igf2bp3	Homo sapiens	2.530753
57	10630	pdpn	Homo sapiens	2.508854
58	89782	lmln	Homo sapiens	2.508487

59	7414	vcl	Homo sapiens	2.492805
60	27035	nox1	Homo sapiens	2.492805
61	90378	samd1	Homo sapiens	2.441653
62	57648	kiaa1522	Homo sapiens	2.441653
63	1785	dnm2	Homo sapiens	2.397854
64	4478	msn	Homo sapiens	2.397854
65	64147	kif9	Homo sapiens	2.383773
66	382	arf6	Homo sapiens	2.360953
67	2934	gsn	Homo sapiens	2.312799
68	3567	il5	Homo sapiens	2.295972
69	51411	bin2	Homo sapiens	2.287086
70	3055	hck	Homo sapiens	2.259345
71	57513	caskin2	Homo sapiens	2.245805
72	54873	palmd	Homo sapiens	2.208129
73	79649	map7d3	Homo sapiens	2.173478
74	10013	hdac6	Homo sapiens	2.148037
75	10966	rab40b	Homo sapiens	2.141406
76	644815	fam83g	Homo sapiens	2.141406
77	79038	zfyve21	Homo sapiens	2.141406
78	22902	rufy3	Homo sapiens	2.141406
79	4322	mmp13	Homo sapiens	2.137711
80	7474	wnt5a	Homo sapiens	2.134298
81	79073	tmem109	Homo sapiens	2.111554
82	9936	cd302	Homo sapiens	2.083637
83	151011	42623	Homo sapiens	2.083637
84	55616	asap3	Homo sapiens	2.083637
85	221496	lemd2	Homo sapiens	2.05742
86	26145	irf2bp1	Homo sapiens	2.05742
87	10634	gas2l1	Homo sapiens	2.05742
88	91748	elmsan1	Homo sapiens	2.032708

89	51133	kctd3	Homo sapiens	2.032708
90	93343	mvb12a	Homo sapiens	2.032708
91	65979	phactr4	Homo sapiens	2.032708
92	92799	shkbp1	Homo sapiens	2.009339
93	11173	adamts7	Homo sapiens	2.009339
