# Supplementary material for: Hepatitis C Virus Enhances the Invasiveness of Hepatocellular Carcinoma via EGFR-Mediated Invadopodia Formation and Activation
Source: Cells. 2019 Nov 5;8(11):1395. doi: 10.3390/cells8111395 (PMC6912298; doi:10.3390/cells8111395)
Supplement: Supplementary file 1 [file cells-08-01395-s001.zip › supplementry/Supp Table 3-min.pdf]

**Supplementary Table 3. Classification of overexpressed invasion-related genes.**

**Invasion - related genes classified by manual curation from GeneCards and UniProt**

| **Gene** | **mRNA logFC** | **mRNA adj.p.value** | **Description** | **Classification** | **Comments** |
|---|---|---|---|---|---|
| PIGR | 4.55 | 1.1E-05 | Polymeric Immunoglobulin Receptor | Cell surface receptor | Member of the immunoglobulin superfamily |
| CXCL5 | 3.77 | 0.0E+00 | C-X-C Motif Chemokine Ligand 5 | Cytokine | Chemokine; neutrophil recruitment and activation; thought to play a role in cancer cell proliferation, migration, and invasion |
| BIRC7 | 3.68 | 2.1E-03 | Baculoviral IAP Repeat Containing 7 | Apoptosis | Inhibitor of apoptosis |
| LCN2 | 3.47 | 5.9E-04 | Lipocalin 2 | Transporter | |
| IL11 | 3.29 | 2.1E-13 | Interleukin 11 | Cytokine | |
| FOSB | 2.50 | 0.0E+00 | FBJ Murine Osteosarcoma Viral Oncogene Homolog B | Transcription | Transcription factor |
| KLF4 | 2.45 | 9.6E-107 | Kruppel-Like Factor 4 (Gut) | Transcription | Transcription factor |
| JUNB | 2.23 | 2.9E-272 | Jun B Proto-Oncogene | Transcription | Transcription factor |
| PDGFB | 2.17 | 3.5E-72 | Platelet Derived Growth Factor Subunit B | Growth factor | |
| SOCS2 | 2.07 | 9.6E-86 | Suppressor Of Cytokine Signaling 2 | Signal transduction | Cytokine-inducible negative regulators of cytokine receptor signaling |
| ICAM1 | 1.91 | 2.1E-304 | Intercellular Adhesion Molecule 1 | Cell-cell adhesion | Leukocyte transendothelial migration, integrin ligand |
| RELB | 1.90 | 4.4E-158 | RELB Proto-Oncogene, NF-KB Subunit | Transcription | |
| FOSL2 | 1.81 | 6.3E-225 | FOS Like Antigen 2 | Transcription | |
| RBM24 | 1.62 | 1.9E-18 | RNA Binding Motif Protein 24 | mRNA regulation | mRNA stability; myogenic differentiation |

| | | | | | |
|---|---|---|---|---|---|
| MAP3K14 | 1.43 | 9.8E-30 | Mitogen-Activated Protein Kinase Kinase Kinase 14 | Signal transduction | Cytokine signaling |
| EGR1 | 1.37 | 1.2E-147 | Early Growth Response 1 | Transcription | Transcription regulation |
| TLR2 | 1.34 | 4.0E-02 | Toll Like Receptor 2 | Cell surface receptor | Plays a fundamental role in pathogen recognition and activation of innate immunity |
| GLI1 | 1.27 | 1.4E-07 | GLI Family Zinc Finger 1 | Transcription | Transcription factor |
| ITGB6 | 1.22 | 5.1E-04 | Integrin Subunit Beta 6 | Cell adhesion | |
| BDNF | 1.10 | 2.6E-02 | Brain-Derived Neurotrophic Factor | Growth factor | Member of the nerve growth factor family of proteins; neuronal |
| CYR61 | 1.09 | 4.0E-158 | Cysteine Rich Angiogenic Inducer 61 | Growth factor | Promotes cell proliferation, chemotaxis, angiogenesis and cell adhesion; role in wound healing; ligand for integrins |
| HBEGF | 1.04 | 2.5E-24 | Heparin Binding EGF Like Growth Factor | Growth factor | Promotes smooth muscle and fibroblast proliferation |
| ETS2 | 1.03 | 5.0E-114 | ETS Proto-Oncogene 2, Transcription Factor | Transcription | Transcription factor; development and apoptosis genes transcription |
| S100P | 0.97 | 6.9E-33 | S100 Calcium Binding Protein P | Cell cycle | Calcium binding protein |
| VIM | 0.92 | 2.0E-150 | Vimentin | Cytoskeleton scaffold protein | Intermediate filament |
| SLC9A1 | 0.91 | 1.2E-54 | Solute Carrier Family 9 Member A1 | Transporter | also known as NHE1; pH regulation |
| THBS1 | 0.89 | 1.1E-285 | Thrombospondin 1 | Cell adhesion | Can bind to fibrinogen, fibronectin, laminin, type V collagen and integrins alpha-V/beta-1 |
| STC2 | 0.88 | 1.5E-89 | Stanniocalcin 2 | Metabolism | Secreted protein; calcium and phosphate homeostasis |
| MUC1 | 0.88 | 1.3E-02 | Mucin 1, Cell Surface Associated | Signal transduction | Mucin; adhesion |
| JAK2 | 0.83 | 1.5E-08 | Janus Kinase 2 | Signal transduction | NRTK; cytokine receptor signaling |
| AHR | 0.83 | 2.2E-57 | Aryl Hydrocarbon Receptor | Transcription | Ligand-activated transcriptional activator; |

| | | | | | |
|---|---|---|---|---|---|
| TNFRSF12A | 0.83 | 4.6E-46 | Tumor Necrosis Factor Receptor Superfamily Member 12A | Cell surface receptor | Aapoptosis; angiogenesis and proliferation of endothelial cells; cell adhesion |
| ITGB8 | 0.80 | 1.3E-04 | Integrin Subunit Beta 8 | Cell adhesion | |
| MFI2 | 0.79 | 3.4E-11 | Melanotransferrin | Metabolism | Involved in iron cellular uptake |
| COL1A1 | 0.79 | 1.3E-02 | Collagen Type I Alpha 1 | Extracellular matrix protein | Type I collagen is found in most connective tissues and is abundant in bone, cornea, dermis and tendon |
| JUN | 0.78 | 1.3E-55 | Jun Proto-Oncogene | Transcription | Transcription factor |
| CDKN2B | 0.78 | 2.3E-63 | Cyclin-Dependent Kinase Inhibitor 2B | Cell cycle | CDK inhibitor |
| RND3 | 0.77 | 1.0E-49 | Rho Family GTPase 3 | Cytoskeletal signaling | Small G protein |
| SERPINE1 | 0.73 | 2.1E-128 | Serpin Family E Member 1 | Protease inhibitor | Inhibitor of fibrinolysis; inhibitor of tissue plasminogen activator (tPA) and urokinase (uPA) |
| CDKN1A | 0.73 | 1.6E-04 | Cyclin-Dependent Kinase Inhibitor 1A | Cell cycle | CDK2, 4 inhibitor; cell cycle inhibitor |
| BCAR1 | 0.72 | 1.9E-35 | BCAR1, Cas Family Scaffolding Protein | Cytoskeletal signaling | p130Cas; adaptor protein; Src substrate, cytoskeleton |
| RHOB | 0.71 | 2.2E-64 | Ras Homolog Family Member B | Cytoskeletal signaling | Small G protein |
| WEE1 | 0.69 | 1.1E-53 | WEE1 G2 Checkpoint Kinase | Cell cycle | Nuclear serine/threonine kinase |
| KLF5 | 0.66 | 5.9E-22 | Kruppel-Like Factor 5 (Intestinal) | Transcription | Transcriptional activator |
| STAT5A | 0.63 | 6.1E-09 | Signal Transducer And Activator Of Transcription 5A | Transcription | Transcriptional activator; growth factor and cytokine signaling |
| DUSP4 | 0.63 | 1.7E-22 | Dual Specificity Phosphatase 4 | Signal transduction | Phosphatase; works in MAPK pathway |
| NES | 0.60 | 2.6E-51 | Nestin | Cytoskeleton scaffold protein | Neuronal intermediate filament family |
| CAV1 | 0.59 | 2.0E-14 | Caveolin 1 | Vesicular transport | Receptor-independent endocytosis |

| | | | | | |
|---|---|---|---|---|---|
| VEGFA | 0.58 | 2.0E-50 | Vascular Endothelial Growth Factor A | Growth factor | Angiogenesis |
| ZFP36 | 0.58 | 4.7E-20 | ZFP36 Ring Finger Protein | mRNA regulation | Binds ARE-containing mRNAs and promots their degradation |
| SRF | 0.58 | 3.1E-30 | Serum Response Factor | Transcription | Transcription factor; proliferation and differentiation |
| MYC | 0.51 | 4.1E-24 | V-Myc Avian Myelocytomatosis Viral Oncogene Homolog | Transcription | Transcription factor |
| RASSF1 | 0.50 | 6.5E-16 | Ras Association Domain Family Member 1 | Apoptosis | Tumor suppressor |
| SIRT1 | 0.50 | 3.0E-17 | Sirtuin 1 | Epigenetic regulation | NAD-dependent protein deacetylase that links transcriptional regulation directly to intracellular energetics |
| NFATC2 | 0.50 | 8.6E-30 | Nuclear Factor Of Activated T-Cells 2 | Transcription | Transcription activator; promotes invasive migration through activation of GPC6 expression and WNT5A signaling pathway |
| NUAK1 | 0.49 | 1.1E-28 | NUAK Family Kinase 1 | Cytoskeletal signaling | |
| TES | 0.47 | 7.0E-28 | Testin LIM Domain Protein | Cytoskeletal signaling | Scaffold protein |
| PAK7 | 0.45 | 2.3E-03 | P21 Protein (Cdc42/Rac)-Activated Kinase 7 | Cytoskeletal signaling | Serine/threonine kinase |
| ENAH | 0.42 | 6.7E-29 | Enabled Homolog (Drosophila) | Cytoskeletal signaling | Ena/VASP proteins |
| AKAP13 | 0.41 | 1.1E-19 | A-Kinase Anchoring Protein 13 | Signal transduction | Adaptor protein |
| LPAR2 | 0.41 | 2.7E-02 | Lysophosphatidic Acid Receptor 2 | Signal transduction | |
| SMAD3 | 0.41 | 1.6E-21 | SMAD Family Member 3 | Transcription | Transcription factor; activated by TGFb signaling pathway |
| SHH | 0.39 | 3.2E-08 | Sonic Hedgehog | Embryonic development | Embryo patterning |
| CREB1 | 0.38 | 7.3E-06 | CAMP Responsive Element Binding Protein 1 | Transcription | Activated by hormonal stimulation of cAMP pathway |
| PTP4A3 | 0.37 | 4.5E-09 | Protein Tyrosine Phosphatase Type IVA, Member 3 | Signal transduction/cell | Cell proliferation; metastasis |

| | | | | | |
|---|---|---|---|---|---|
| | | | | cycle | |
| ANXA4 | 0.35 | 3.0E-18 | Annexin A4 | Vesicular transport | |
| JAK1 | 0.35 | 5.7E-17 | Janus Kinase 1 | Signal transduction | NRTK; cytokine receptor signaling |
| CTNND1 | 0.35 | 1.0E-37 | Catenin Delta 1 | Cell-cell adhesion/signal transduction | Wnt signaling pathway |
| GPR160 | 0.35 | 1.2E-02 | G Protein-Coupled Receptor 160 | Cell surface receptor | |
| LATS1 | 0.34 | 1.3E-06 | Large Tumor Suppressor Kinase 1 | Cell cycle | Serine/threonine kinase |
| SOX9 | 0.34 | 1.3E-13 | SRY-Box 9 | Transcription | Transcription factor; chondrocyte differentiation; skeletal development |
| FMNL2 | 0.32 | 6.9E-08 | Formin Like 2 | Actin remodeling | Formin-like protein |
| BMP1 | 0.32 | 6.0E-07 | Bone Morphogenetic Protein 1 | Growth factor | Involved in cartilage and bone formation; cleaves pro-collagen; peptidase |
| CMTM3 | 0.32 | 7.2E-11 | CKLF Like MARVEL Transmembrane Domain Containing 3 | Chemokine | Chemokine-like factor; B-cell signaling |
| SPAG9 | 0.32 | 1.6E-12 | Sperm Associated Antigen 9 | Signal transduction | Scaffold protein |
| CIC | 0.30 | 2.3E-07 | Capicua Transcriptional Repressor | Transcription | Transcriptional repressor which may play a role in development of the central nervous system |
| RHOC | 0.30 | 2.7E-05 | Ras Homolog Family Member C | Cytoskeletal signaling | Small G protein; overexpressed in cancer |
| MCAM | 0.30 | 3.0E-04 | Melanoma Cell Adhesion Molecule | Cell adhesion | |
| MMP14 | 0.28 | 1.0E-08 | Matrix Metallopeptidase 14 | Matrix metalloproteinase | Membranal MMP; activates MMP2; endopeptidase |
| MAMLD1 | 0.28 | 2.9E-04 | Mastermind Like Domain Containing 1 | Transcription | Transcriptional co-activator |
| PTPN11 | 0.27 | 2.5E-08 | Protein Tyrosine Phosphatase, Non-Receptor Type 11 | Signal transduction | Shp2; tyrosine phosphatase |

| | | | | | |
|---|---|---|---|---|---|
| CLDN6 | 0.27 | 1.6E-05 | Claudin 6 | Cytoskeleton scaffold protein | The protein is an integral membrane protein and is one of the entry cofactors for hepatitis C virus |
| MAPK7 | 0.26 | 5.1E-04 | Mitogen-Activated Protein Kinase 7 | Signal transduction | Proliferation, differentiation and cell survival |
| INSR | 0.25 | 1.3E-08 | Insulin Receptor | Metabolism | RTK |
| JUND | 0.25 | 1.4E-04 | Jun D Proto-Oncogene | Transcription | Component of transcription factor complex (AP1) |
| MAP3K2 | 0.24 | 3.8E-04 | Mitogen-Activated Protein Kinase Kinase Kinase 2 | Signal transduction | Serine/threonine kinase |
| FERMT2 | 0.24 | 2.6E-04 | Fermitin Family Member 2 | Cytoskeletal signaling | Scaffold protein |
| FOXO1 | 0.22 | 6.5E-05 | Forkhead Box O1 | Transcription | Transcription factor; triggers apoptosis genes |
| PXN | 0.22 | 3.0E-05 | Paxillin | Cytoskeletal signaling | Cell migration; focal adhesions |
| SETD2 | 0.21 | 7.6E-07 | SET Domain Containing 2 | Epigenetic regulation | Histone methyltransferase |
| ZDHHC7 | 0.21 | 1.1E-05 | Zinc Finger DHHC-Type Containing 7 | Metabolism | |
| ARF6 | 0.21 | 1.0E-05 | ADP Ribosylation Factor 6 | Vesicular transport | Small G protein |
| FUT8 | 0.20 | 2.0E-03 | Fucosyltransferase 8 | Metabolism | |
| IL6R | 0.20 | 2.7E-04 | Interleukin 6 Receptor | Cell surface receptor | Immune cell signaling; cytokine signaling |
| ROCK1 | 0.20 | 1.5E-03 | Rho Associated Coiled-Coil Containing Protein Kinase 1 | Cytoskeletal signaling | Serine/threonine kinase of Rho |
| BCL2L1 | 0.18 | 1.0E-04 | BCL2 Like 1 | Apoptosis | Inhibitor of apoptosis |
| CD9 | 0.17 | 4.0E-02 | CD9 Molecule | Cell adhesion | |
| TJP1 | 0.16 | 4.3E-03 | Tight Junction Protein 1 | Cell-cell adhesion | Plays a role in the regulation of cell migration by targeting CDC42BPB to the leading edge of migrating cells |

| | | | | | |
|---|---|---|---|---|---|
| LGALS3 | 0.16 | 2.5E-02 | Lectin, Galactoside Binding Soluble 3 | Cell adhesion | Lectin; may mediate with the alpha-3, beta-1 integrin the stimulation by CSPG4 of endothelial cells migration |
| RAP1GAP | 0.15 | 1.6E-02 | RAP1 GTPase Activating Protein | Signal transduction | GAP of Rap1 |
| ABL1 | 0.15 | 3.8E-03 | ABL Proto-Oncogene 1, Non-Receptor Tyrosine Kinase | Cytoskeletal signaling | NRTK |
| NOTCH1 | 0.14 | 4.3E-02 | Notch 1 | Signal transduction | Differentiation, proliferation and apoptotic signaling |
| PTGER4 | 0.14 | 4.0E-02 | Prostaglandin E Receptor 4 | Metabolism | GPCR |
| SRC | 0.14 | 3.1E-02 | SRC Proto-Oncogene, Non-Receptor Tyrosine Kinase | Signal transduction | NRTK |
| NDRG1 | 0.13 | 3.6E-02 | N-Myc Downstream Regulated 1 | Signal transduction | Involved in stress responses, hormone responses, cell growth, and differentiation |
| HIF1A | 0.13 | 1.3E-03 | Hypoxia Inducible Factor 1 Alpha Subunit | Transcription | Transcription regulation; adaptation to hypoxia; angiogenesis related |
| BRAF | 0.13 | 3.6E-02 | B-Raf Proto-Oncogene, Serine/Threonine Kinase | Signal transduction | Serine/treonine kinase, MAPK pathway; proliferation and differentiation |
| BAMBI | 0.13 | 2.3E-02 | BMP And Activin Membrane-Bound Inhibitor | Signal transduction | Negatively regulates TGF-beta signaling |
| TP53 | 0.13 | 5.7E-03 | Tumor Protein P53 | Cell cycle | Tumor suppressor; induces growth arrest or apoptosis depending on the physiological circumstances and cell type |
| CRK | 0.12 | 1.3E-02 | V-Crk Avian Sarcoma Virus CT10 Oncogene Homolog | cytoskeletal signaling | Adaptor protein |
| CDKN1B | 0.12 | 1.2E-02 | Cyclin-Dependent Kinase Inhibitor 1B | Cell cycle | Cyclin-dependent kinase inhibitor |
| ADAM17 | 0.12 | 3.1E-02 | ADAM Metallopeptidase Domain 17 | Matrix metalloproteinase | |
| KEAP1 | 0.10 | 4.6E-02 | Kelch Like ECH Associated Protein 1 | Protein ubiquitination | E3 ligase |
| SNX9 | 0.09 | 4.9E-02 | Sorting Nexin 9 | Vesicular transport/actin remodeling | Works with dynamin 1 among others |

| | | | | | |
|---|---|---|---|---|---|
| SOX4 | 0.08 | 3.3E-03 | SRY-Box 4 | Transcription | Transcriptional activator; regulation of embryonic development and in the determination of the cell fate |
