# Supplementary material for: Hepatitis C Virus Enhances the Invasiveness of Hepatocellular Carcinoma via EGFR-Mediated Invadopodia Formation and Activation
Source: Cells. 2019 Nov 5;8(11):1395. doi: 10.3390/cells8111395 (PMC6912298; doi:10.3390/cells8111395)
Supplement: Supplementary file 1 [file cells-08-01395-s001.zip › supplementry/Supp Table 4-min.pdf]

Supplementary Table 4. Classification of overexpressed invadopodia-related genes.

Invadopodia - related genes classified by manual curation from GeneCards and UniProt

Gene	mRNA logFC	mRNA adj.p.value	Description	Classification	Comments
PDGFB	2.17	3.5E-72	Platelet Derived Growth Factor Subunit B	Growth factor	
ICAM1	1.91	2.1E-304	Intercellular Adhesion Molecule 1	Cell-cell adhesion	Leukocyte transendothelial migration, integrin ligand
CSF1	1.52	5.6E-168	Colony Stimulating Factor 1	Growth factors and cytokines	Signals to macrophages
NRG1	1.24	6.0E-07	Neuregulin 1	Growth factors and cytokines	Ligand of ERBB3, ERBB4; cell-cell interaction
SVIL	1.12	2.0E-28	Supervillin	Actin remodeling	Links between actin-membrane
HBEGF	1.04	2.5E-24	Heparin Binding EGF Like Growth Factor	Growth factors and cytokines	Promotes smooth muscle and fibroblast proliferation
S100P	0.97	6.9E-33	S100 Calcium Binding Protein P	Cell cycle	Calcium binding protein
SLC9A1	0.91	1.2E-54	Solute Carrier Family 9 Member A1	Ion transporter	also known as NHE1; pH regulation
FAM83G	0.91	2.0E-92	Family With Sequence Similarity 83 Member G	Signal transduction	BMP signaling pathway
GRASP	0.90	1.8E-07	GRP1 (General Receptor For Phosphoinositides 1)-Associated Scaffold Protein	Signal transduction	Adaptor protein; neuronal
MUC1	0.88	1.3E-02	Mucin 1, Cell Surface Associated	Signal transduction	Mucin; adhesion
JAK2	0.83	1.5E-08	Janus Kinase 2	Signal transduction	NRTK; cytokine receptor signaling
JUN	0.78	1.3E-55	Jun Proto-Oncogene	Transcription	Transcription factor; triggers proliferation genes
FOXO3	0.78	2.2E-52	Forkhead Box O3	Transcription	Transcription factor; triggers apoptosis genes
CDKN1A	0.73	1.6E-04	Cyclin-Dependent Kinase	Cell cycle	CDK inhibitor

			Inhibitor 1A		
BCAR1	0.72	1.9E-35	BCAR1, Cas Family Scaffolding Protein	Cytoskeletal signaling	p130Cas; adaptor protein; Src substrate, cytoskeleton
RHOU	0.67	1.3E-47	Ras Homolog Family Member U	Cytoskeletal signaling	Small G protein
STAT5A	0.63	6.1E-09	Signal Transducer And Activator Of Transcription 5A	Transcription	Transcription factor; cytokine signaling
CAV1	0.59	2.0E-14	Caveolin 1	Vesicular transport	Receptor-independent endocytosis
SYNJ2	0.59	1.5E-57	Synaptojanin 2	Signal transduction	Inositol 5-phosphatase; membrane trafficking
VEGFA	0.58	2.0E-50	Vascular Endothelial Growth Factor A	Growth factor	Angiogenesis
TGM2	0.51	1.4E-28	Transglutaminase 2	Metabolism	Induces resistance to proteolytic cleavage
DNM1	0.45	1.9E-04	Dynamin 1	Cytoskeletal signaling	GTP-binding protein; receptor mediated endocytosis; microtubule binding and regulation
SMAD3	0.41	1.6E-21	SMAD Family Member 3	Transcription	Transcription factor; activated by TGFb signaling pathway
RHOQ	0.40	4.6E-11	Ras Homolog Family Member Q	Cytoskeletal signaling	Small G protein; epithelial cell polarization; filopodia formation
VCL	0.39	7.3E-25	Vinculin	Cytoskeletal signaling	Cell migration; actin binding
CREB1	0.38	7.3E-06	CAMP Responsive Element Binding Protein 1	Transcription	Activated by hormonal stimulation of cAMP pathway
BRAP	0.36	5.2E-07	BRCA1 Associated Protein	Signal transduction	Negative regulation of Ras-MAPK pathway; cytoplasmic retention regulation
EPS8	0.33	2.0E-17	Epidermal Growth Factor Receptor Pathway Substrate 8	Cytoskeletal signaling	actin remodeling; filopodia
BMP1	0.32	6.0E-07	Bone Morphogenetic Protein 1	Growth factors and cytokines	Involved in cartilage and bone formation; cleaves pro-collagen; peptidase
KIAA1522	0.32	3.7E-09	KIAA1522	Unknown	Mutated in cancer; prognostic marker
CASKIN2	0.30	3.4E-06	CASK Interacting Protein 2	Signal transduction	
RHOC	0.30	2.7E-05	Ras Homolog Family Member C	Cytoskeletal signaling	Small G protein; overexpressed in cancer
MMP14	0.28	1.0E-08	Matrix Metallopeptidase 14	Matrix metalloproteinase	Membranal MMP; activates MMP2; endopeptidase

PTPN11	0.27	2.5E-08	Protein Tyrosine Phosphatase, Non-Receptor Type 11	Signal transduction	Shp2; tyrosine phosphatase
MAPK7	0.26	5.1E-04	Mitogen-Activated Protein Kinase 7	Signal transduction	Proliferation, differentiation and cell survival signaling
FERMT2	0.24	2.6E-04	Fermitin Family Member 2	Cytoskeletal signaling	Mediates integrin signaling
KIF5B	0.23	2.8E-05	Kinesin Family Member 5B	Motor protein	Microtubule-dependent motor required for normal distribution of mitochondria and lysosomes
CDK5RAP1	0.23	4.9E-06	CDK5 Regulatory Subunit Associated Protein 1	Cell cycle	Regulator of cyclin-dependent kinase 5 activity
PXN	0.22	3.0E-05	Paxillin	Cytoskeletal signaling	Cell migration; focal adhesions
ARF6	0.21	1.0E-05	ADP Ribosylation Factor 6	Vesicular transport	Small G protein
ROCK1	0.20	1.5E-03	Rho Associated Coiled-Coil Containing Protein Kinase 1	Cytoskeletal signaling	Serine/threonine kinase of Rho
TRIP10	0.19	1.6E-02	Thyroid Hormone Receptor Interactor 10	Vesicular transport	Regulation of actin reorganization in endocytosis and tubulation
RUFY3	0.18	1.2E-02	RUN And FYVE Domain Containing 3	Actin remodeling	Axon formation and development; regulates F-actin-enriched protrusive structures at the cell periphery
SOS1	0.17	8.0E-03	SOS Ras/Rac Guanine Nucleotide Exchange Factor 1	Signal transduction	Ras GEF
TJP1	0.16	4.3E-03	Tight Junction Protein 1	Cell-cell adhesion	Cell-cell junctions
ACTN4	0.16	4.9E-05	Actinin Alpha 4	Actin remodeling	Cytoskeletal scaffold protein
LASP1	0.15	2.7E-04	LIM And SH3 Protein 1	Actin remodeling	Regulation of dynamic actin-based, cytoskeletal activities; actin-membrane connection
ABL1	0.15	3.8E-03	ABL Proto-Oncogene 1, Non-Receptor Tyrosine Kinase	Cytoskeletal signaling	NRTK
PTGER4	0.14	4.0E-02	Prostaglandin E Receptor 4	Metabolism	GPCR; receptor for prostaglandin E2 (PGE2)
SRC	0.14	3.1E-02	SRC Proto-Oncogene, Non-Receptor Tyrosine Kinase	Signal transduction	NRTK
BRAF	0.13	3.6E-02	B-Raf Proto-Oncogene, Serine/Threonine Kinase	Signal transduction	Serine/treonine kinase, MAPK pathway; proliferation and differentiation
TP53	0.13	5.7E-03	Tumor Protein P53	Cell cycle	Tumor suppressor; induces growth arrest

					or apoptosis depending on the physiological circumstances and cell type
CDKN1B	0.12	1.2E-02	Cyclin-Dependent Kinase Inhibitor 1B	Cell cycle	Cyclin-dependent kinase inhibitor
ADAMTS1	0.12	3.3E-02	ADAM Metallopeptidase With Thrombospondin Type 1 Motif 1	Matrix metalloproteinase	
SNX9	0.09	4.9E-02	Sorting Nexin 9	Vesicular transport/actin remodeling	Works with dynamin 1 among others
