# Supplementary material for: Hepatitis C Virus Enhances the Invasiveness of Hepatocellular Carcinoma via EGFR-Mediated Invadopodia Formation and Activation
Source: Cells. 2019 Nov 5;8(11):1395. doi: 10.3390/cells8111395 (PMC6912298; doi:10.3390/cells8111395)
Supplement: Supplementary file 1 [file cells-08-01395-s001.zip › supplementry/Supp Table 5-min.pdf]

Supplementary Table 5. Receptor-ligand correlation.

RTKs activated in PamGene	Official gene name	Comments	Receptor overexpressed/ downregulated in mRNAseq?	Receptor involved in invasion/ invadopodia?	Ligands	Ligand overexpressed/ downregulated in mRNAseq?	Ligand involved in invasion/ invadopodia?
AXL	AXL	Also acts as a receptor for several viruses		Yes (invasion)	GAS6		
CCK4 (PTK7)	PTK7	Inactive tyrosine kinase involved in Wnt signaling pathway			??		
FMS	CSF1R	Colony stimulating factor 1 receptor			CSF1, IL34	Overexpressed	
KIT	KIT	SCFR			KITLG/SCF		
LMR2 (KIAA1079)	LMTK2	Serine/threonine/tyrosine kinase receptor			??		
MER	MERTK	ECM receptor	Downregulated		LGALS3, TUB, TULP1, GAS6	Overexpressed	Yes (invasion)
MET	MET	HGF receptor		Yes (invasion)	HGF, VEGFA	Overexpressed	Yes (invasion)
PDGFRB	PDGFRB				PDGFA, PDGFB, PDGFD	Overexpressed	
RET	RET				GDNF	Overexpressed	
RON	MST1R	MST1R, macrophage stimulating receptor, ECM receptor			MST1		

ROS1	ROS1				Orphan receptor (no ligand)		
RYK	RYK	Neuronal receptor, co-receptor with FZD8 for WNTs			WNT1, WNT3, WNT3A, WNT5A		
TNK1	TNK1	Negative regulation of cell growth by down regulation of Ras-MAPK pathway			??		
TYRO3	TYRO3	ECM receptor, acts as a receptor for several viruses	Downregulated		TULP1, GAS6		
ROR1	ROR1	RTK-like orphan receptor (no known ligand)	Downregulated		WNT5A (GeneCards)		
ROR2	ROR2	RTK-like orphan receptor (no known ligand)			WNT5A (GeneCards)		
DDR1	DDR1	Collagen receptor, regulates ECM remodelling by up-regulation of MMP2, MMP7, MMP9 (facilitates invasion)		Yes (invasion)	**COL2A1**, COL3A1, COL4A1, COL5A1 (STRING)	Downregulated	
DDR2	DDR2	collagen receptor, regulates ECM remodelling by up-regulation of MMP1, MMP2, MMP13		Yes (invasion)	COL1A1, COL2A1, COL3A1, COL4A1, COL5A1	Downregulated/ overexpressed	

		(facilitates invasion)			(STRING)		
EPHA1	EPHA1	Ephrin receptor family,neuronal/cancer, juxtacrine signaling			EFNA1, EFNA3, EFNA4 (Uniprot)	Downregulated	
EPHA10	EPHA10	Ephrin receptor family,neuronal/cancer, juxtacrine signaling			EFNB1, EFNB2 (STRING via GeneCards)		
EPHA2	EPHA2	Ephrin receptor family,neuronal/cancer, juxtacrine signaling		Yes (invasion)	EFNA1, EFNA2, EFNA5	Downregulated	
EPHA3	EPHA3	Ephrin receptor family,neuronal/cancer, juxtacrine signaling			EFNA5, EFNA1 (Uniprot)	Downregulated	
EPHA4	EPHA4	Ephrin receptor family,neuronal/cancer, juxtacrine signaling			EFNA1, EFNB3 (Uniprot)		
EPHA5	EPHA5	Ephrin receptor family,neuronal/cancer, juxtacrine signaling			EFNA5, EFNA1, EFNA4 (STRING via GeneCards)	Downregulated	
EPHA6	EPHA6	Ephrin receptor family,neuronal/cancer, juxtacrine signaling	Overexpressed		EFNA5 (BioGrid), EFNA1 (STRING via GeneCards)	Downregulated	
EPHA7	EPHA7	Ephrin receptor family,neuronal/cancer, juxtacrine signaling			EFNA5 (Uniprot), EFNA1, EFNA4 (STRING via	Downregulated	

					GeneCards)		
EPHA8	EPHA8	Ephrin receptor family,neuronal/cancer, juxtacrine signaling			EFNA2, EFNA3, EFNA5 (Uniprot)	Downregulated	
EPHB1	EPHB1	Ephrin receptor family,neuronal/cancer, juxtacrine signaling			EFNB1, EFNB2, EFNB3 (Uniprot)		
EPHB2	EPHB2	Ephrin receptor family,neuronal/cancer, juxtacrine signaling		Yes (invasion)	EFNB1, EFNB2 (Uniprot)		
EPHB3	EPHB3	Ephrin receptor family,neuronal/cancer, juxtacrine signaling	Downregulated		EFNB2 (Uniprot), EFNB3 (STRING via GeneCards)		
EPHB4	EPHB4	Ephrin receptor family,neuronal/cancer, juxtacrine signaling	Downregulated	Yes (invasion)	EFNB2 (Uniprot)		
EGFR	EGFR	EGFR family		Yes (invasion)	EGF, TGFA, amphiregulin (AREG), EPGN, BTC, EREG, HBEGF	Overexpressed	Yes (invasion)
ERBB2	ERBB2	EGFR family		Yes (invasion)	No ligand, HB-EGF can bind to heterodimer of ErbB2 with other Erbs	Overexpressed	Yes (invasion)
ERBB3	ERBB3	EGFR family	Downregulated	Yes (invasion)	NRG1, CSPG5	Overexpressed	

ERBB4	ERBB4	EGFR family			NRG1, NRG2, NRG3, NRG4, BTC, EREG, HBEGF	Overexpressed	Yes (invasion)
FGFR1	FGFR1	FGFR family	Downregulated		FGF1, FGF2, FGF3, FGF6, FGF7 (STRING via GeneCards)		
FGFR2	FGFR2	FGFR family			FGF1, FGF3, FGF6, FGF7, FGF8 (STRING via GeneCards)		
FGFR3	FGFR3	FGFR family	Downregulated		FGF3, FGF4, FGF5, FGF6, FGF7 (STRING via GeneCards)		
FGFR4	FGFR4	FGFR family	Downregulated		FGF19, FGF1, FGF3, FGF4, FGF5, FGF9 (STRING via GeneCards)	Overexpressed	
FLT1	FLT1	VEGFR1 (angiogenesis)			VEGFA, VEGFB, PGF	Downregulated/ overexpressed	Yes (invasion)
FLT3	FLT3	Regulates hematopoiesis			FLT3LG		
FLT4	FLT4	VEGFR3 (angiogenesis)			VEGFC, VEGFD		
KDR	KDR	VEGFR2 (angiogenesis)		Yes (invasion)	VEGFA, VEGFC, VEGFD, FGF2	Overexpressed	Yes (invasion)

ALK	ALK	Neuronal receptor, insulin receptor superfamily			PTN, MDK	Downregulated	
INSR	INSR	Insulin receptor	Overexpressed		Insulin, IGF1, IGF2	Downregulated	
IGF1R	IGF1R	Insulin-like growth factor receptor		Yes (invasion)	Insulin, IGF1, IGF2	Downregulated	
IRR (INSRR)	INSRR	Insulin receptor related receptor, functions as a pH sensing receptor which is activated by increased extracellular pH			?? Extracellular pH?		
LTK	LTK	Leukocyte receptor tyrosine kinase, member of ROS/insulin receptor family, exact function unknown			??		
TRKA (NTRK1)	NTRK1	Neuronal receptor			NGF, NTF3/ neurotrophin3		
TRKB	NTRK2	Neuronal receptor			BDNF, NTF4, NTF3		
TRKC	NTRK3	Neuronal receptor			NTF3, NTF4, BDNF, NGF		
