# Supplementary material for: Hepatitis C Virus Enhances the Invasiveness of Hepatocellular Carcinoma via EGFR-Mediated Invadopodia Formation and Activation
Source: Cells. 2019 Nov 5;8(11):1395. doi: 10.3390/cells8111395 (PMC6912298; doi:10.3390/cells8111395)
Supplement: Supplementary file 1 [file cells-08-01395-s001.zip › supplementry/Supplementary Information_revision2.docx]

**Supplementary Information**

**Supplementary Figures**

**Figure S1. Immunostaining to validate 100% infection of HCV in Huh7.5 cells.** HCV-infected or non-infected cells were stained with HCV-positive serum and anti-human 488 Alexa fluor as secondary antibody and visualized by fluorescence microscopy. Scale bars: 50μm.

**Figure S2.** **HCV infection does not affect cell migration**. (A) Non-infected or HCV-infected cells were plated on the upper chamber of Transwells and allowed to migrate for 24 hours. Filters were stained with crystal violet and the cells that migrated into the lower side of the filters were counted. The violin plot present the number of migrated cell in HCV-infected compared to non-infected cells. n = 10 fields from three independent experiments (P=0.0641, Student’s *t*-test). (B) Fold change of number of migrated or invaded cells in HCV infected relative to non-infected cells.

**Figure S3.** **HCV infection increases the expression of MMPs.** Evaluation of MMP2, MMP9 and MMP14 genes expression by qRT-PCR from non-infected and HCV-infected Huh7.5 HCC cells. * P<0.05; *** P<0.001, Student’s *t*-test. Values are presented as relative quantity (RQ) compared to non-infected cells. Shown are results from three independent experiments.

**Figure S4. HCV infection induces activation of receptor and non-receptor tyrosine kinases involved in invasion and invadopodia formation.** Overlap of activated tyrosine kinases upon HCV infection (Pamgene) with invasion (A) and with invadopodia (B) associated genes obtained by literature mining using GLAD4U and ALS. Receptor tyrosine kinases are shown in green and non-receptor tyrosine kinases in cyan in the close-up views of the networks.

**Figure S5. Human-specific qRT-PCR.** (A) **qRT-**PCR for human or mouse genomic DNA was performed with human and mouse specific primers to confirm the specificity of the primers. (B) Standard curve of qRT-PCR for human and mouse DNA standards. Y-axis: - Log (2) Amplification ratio of the results of the qRT-PCR determined by the average of triplicate samples. X-axis: percentage of human cells. (C) RNA from mouse and human cells were extracted and reverse transcribed into cDNA. To validate the specificity of the primers to human genes, PCR was performed with primers for the genes *ROCK, SLC9A1, CTTN* and *GAPDH*. (D) Non-infected or HCV-infected Huh7.5 cells were injected into the left lobe of the liver of NSG male mice. Two weeks following injection, mouse liver left lobe and right lobes were harvested, RNA was extracted and reverse transcribed into cDNA. Expression level of the invadopodia-associated gene *CTTN* (expression cortactin), was quantified in the right and left lobes by qRT-PCR. n = 3/6 mice per group.

**Supplementary Tables**

**Table S1. Quantitative real-time PCR primers.** Shown are the sequences of the gene-specific primers used for qRT-PCR experiments of Figures 1 and 3. Human *GAPDH* was used as a normalization control. Primers were designed using the Primer3 PCR Primer Design Tool. (A) or purchased as custom validated PrimeTime® qPCR probes from IDT (B).

**Table S2. Literature mining of invasion- and invadopodia-related genes.** Shown are all invasion- and invadopodia-associated genes from literature mining using ALS and GLAD4U as described in Materials and Methods.

**Table S3. Classification of overexpressed invasion-related genes.** Shown is a list of invasion-associated genes from the overlap of RNA-sequencing genes and literature mining using the term “invasion”, with their mRNA log fold change values and adjusted p values. Genes were classified by manual curation using GeneCards and UniProt.

**Table S4. Classification of overexpressed invadopodia-related genes.** Shown is a list of invadopodia-associated genes from the overlap of RNA-sequencing genes and literature mining using the term “invasion”, with their mRNA logarithmic fold-change values and adjusted p values. Genes were classified by manual curation using GeneCards and UniProt.

**Table S5. Receptor-ligand correlation.** Shown is a list of activated receptor tyrosine kinases (RTKs) from Pamgene analysis. Receptor tyrosine kinase correlation with their corresponding ligands was performed by manual curation using GeneCards and UniProt. Ligands that were overexpressed in RNA sequencing are marked in red, ligands that were down regulated in RNA sequencing are marked in green.
