# Supplementary figures and images for: Hepatitis C Virus Enhances the Invasiveness of Hepatocellular Carcinoma via EGFR-Mediated Invadopodia Formation and Activation
Source: Cells. 2019 Nov 5;8(11):1395. doi: 10.3390/cells8111395 (PMC6912298; doi:10.3390/cells8111395)

# Supplementary Figure 1

Non-infected

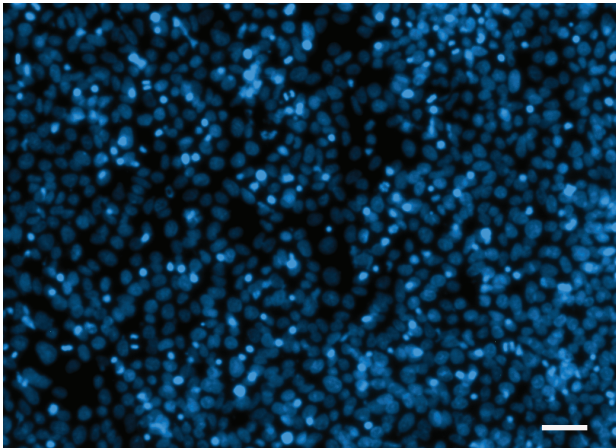

HCV-infected

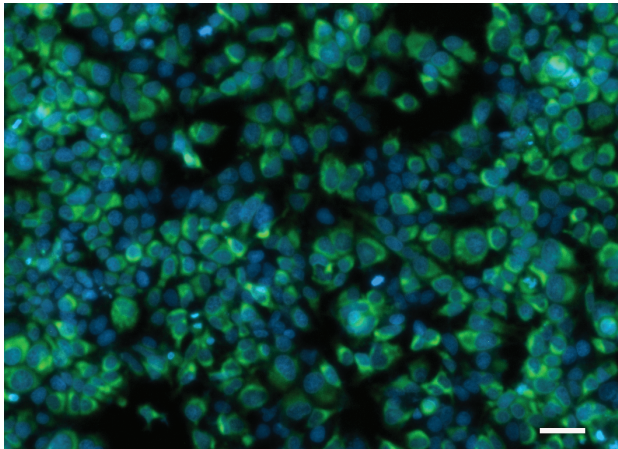

Supplement: Supplementary file 1 [file cells-08-01395-s001.zip › supplementry/Supp FIG1_rev.pdf]

# Supplementary Figure 2

**A**

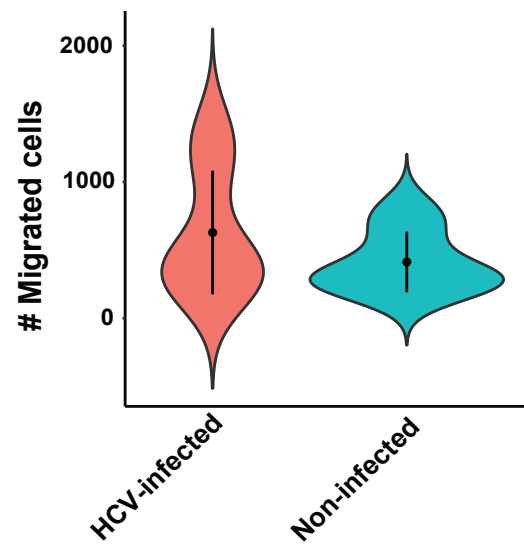

**B**

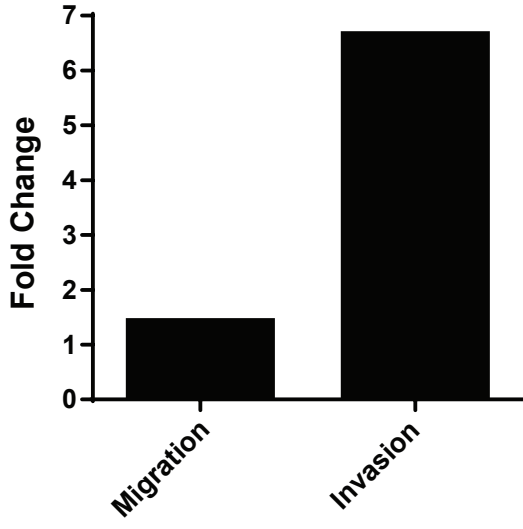

Supplement: Supplementary file 1 [file cells-08-01395-s001.zip › supplementry/Supp FIG2_rev2.pdf]

# Supplementary Figure 3

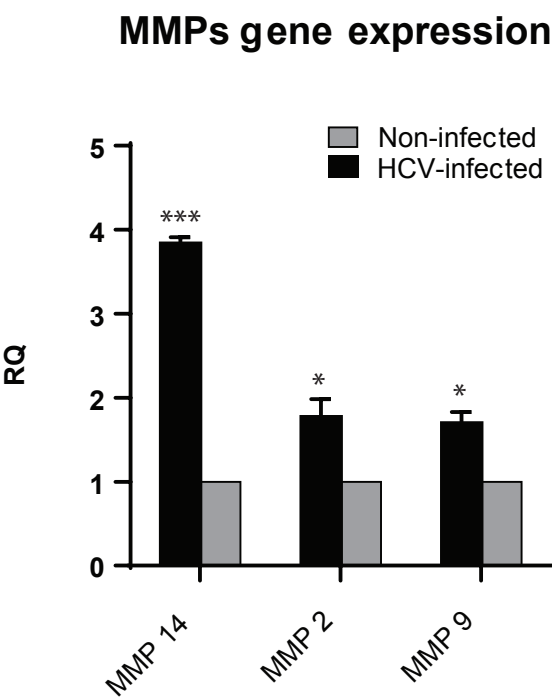

Supplement: Supplementary file 1 [file cells-08-01395-s001.zip › supplementry/Supp FIG3_rev.pdf]

# Supplementary Figure 4

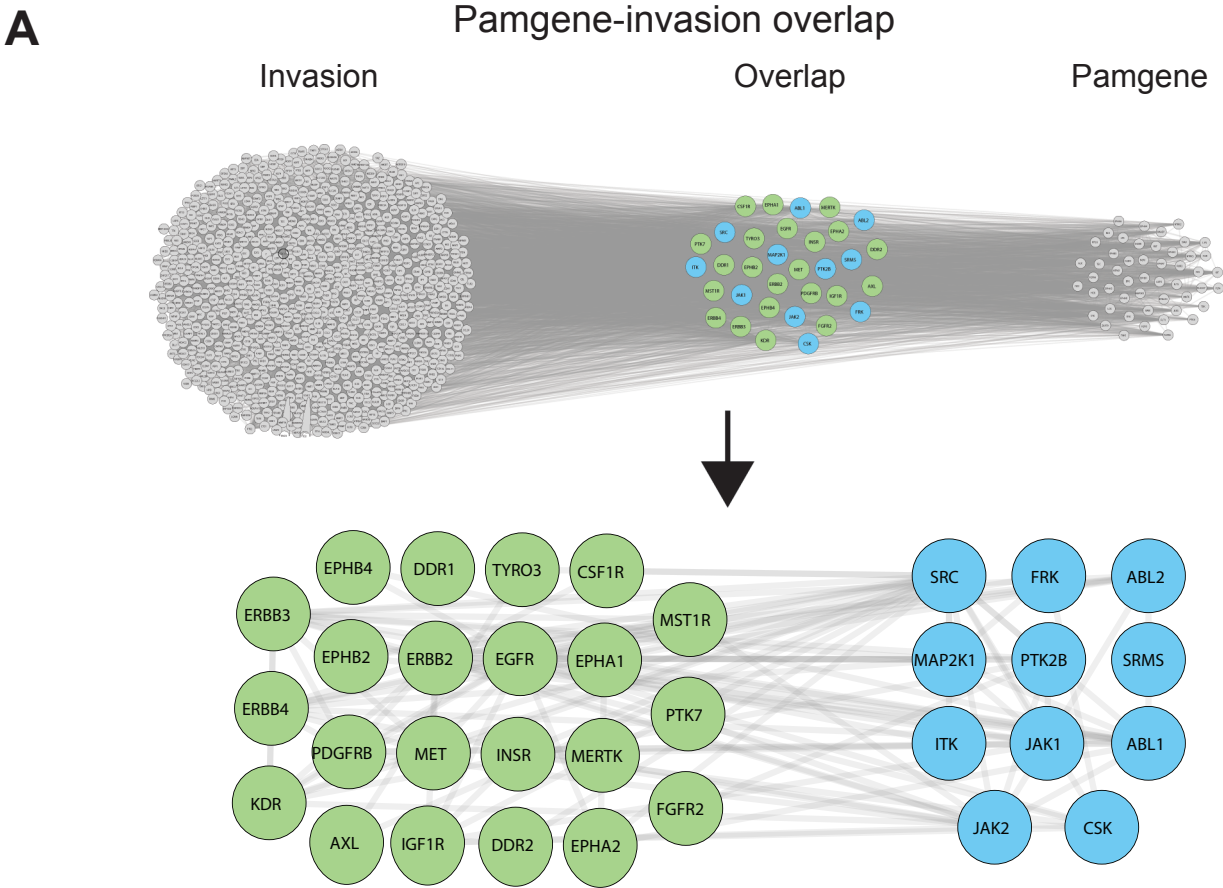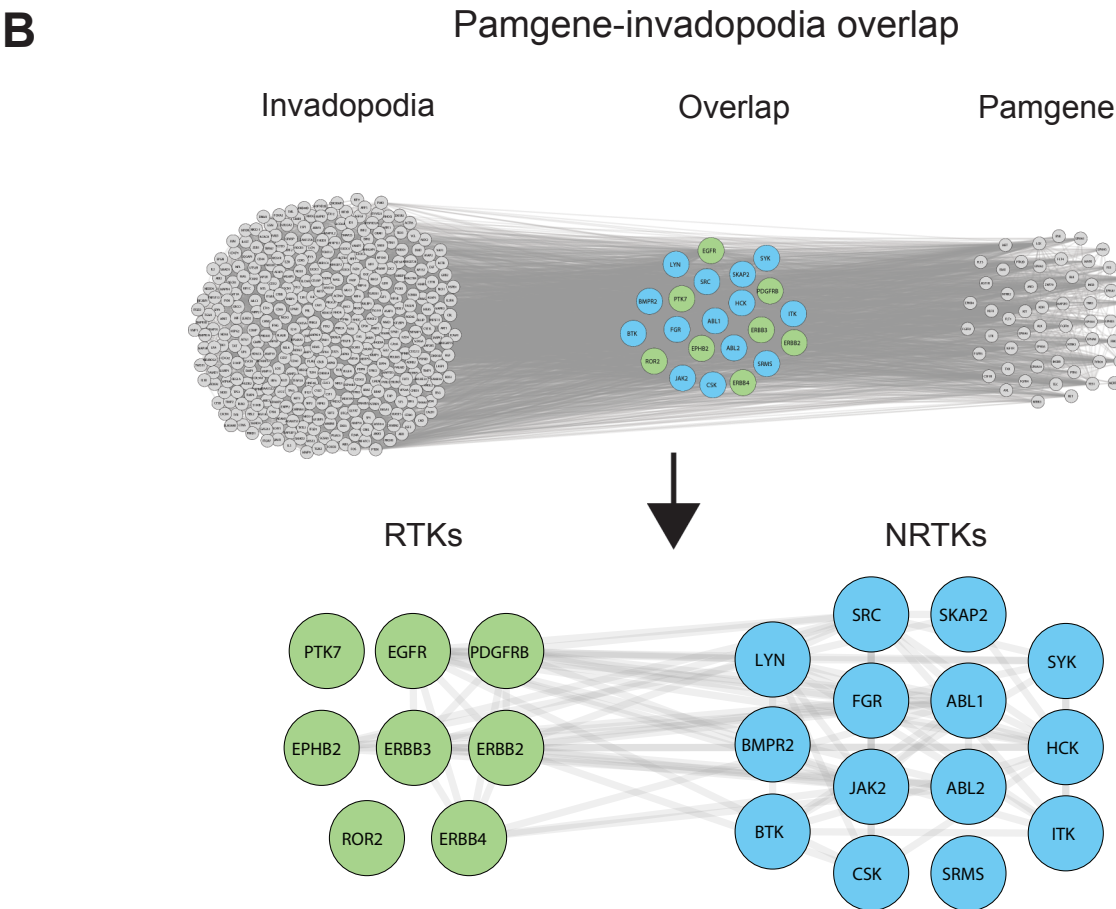

Supplement: Supplementary file 1 [file cells-08-01395-s001.zip › supplementry/Supp FIG4_rev.pdf]

**A**

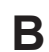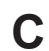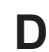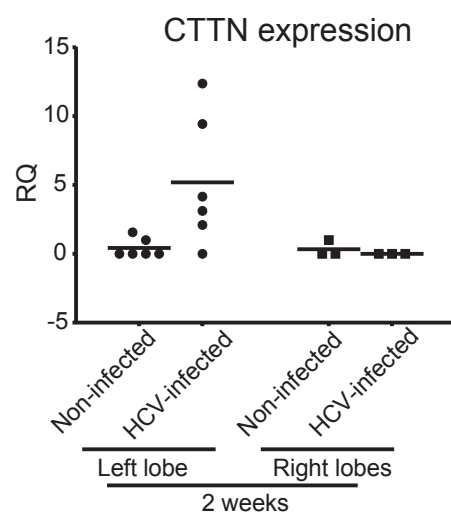

Supplement: Supplementary file 1 [file cells-08-01395-s001.zip › supplementry/Supp FIG5_rev.pdf]
